# Integration of Transcriptome and Epigenome to Identify and Develop Prognostic Markers for Ovarian Cancer

**DOI:** 10.1155/2022/3744466

**Published:** 2022-08-30

**Authors:** Can Xu, Wei Cao

**Affiliations:** Department of Pathology, Shengjing Hospital of China Medical University, Shenyang, Liaoning, China

## Abstract

DNA methylation is a widely researched epigenetic modification. It is associated with the occurrence and development of cancer and has helped evaluate patients' prognoses. However, most existing DNA methylation prognosis models have not simultaneously considered the changes of the downstream transcriptome. *Methods*. The RNA-Sequencing data and DNA methylation omics data of ovarian cancer patients were downloaded from The Cancer Genome Atlas (TCGA) database. The Consensus Cluster Plus algorithm was used to construct the methylated molecular subtypes of the ovary. Lasso regression was employed to build a multi-gene signature. An independent data set was applied to verify the prognostic value of the signature. The Gene Set Variation Analysis (GSVA) was used to carry out the enrichment analysis of the pathways linked to the gene signature. The IMvigor 210 cohort was used to explore the predictive efficacy of the gene signature for immunotherapy response. *Results*. We distinguished ovarian cancer samples into two subtypes with different prognosis, based on the omics data of DNA methylation. Differentially expressed genes and enrichment analysis among subtypes indicated that DNA methylation was related to fatty acid metabolism and the extracellular matrix (ECM)-receptor. Furthermore, we constructed an 8-gene signature, which proved to be efficient and stable in predicting prognostics in ovarian cancer patients with different data sets and distinctive pathological characteristics. Finally, the 8-gene signature could predict patients' responses to immunotherapy. The polymerase chain reaction experiment was further used to verify the expression of 8 genes. *Conclusion*. We analyzed the prognostic value of the related genes of methylation in ovarian cancer. The 8-gene signature predicted the prognosis and immunotherapy response of ovarian cancer patients well and is expected to be valuable in clinical application.

## 1. Introduction

Ovarian cancer is the most common and fatal [1] malignant tumor in the gynecological system and can occur at any age. The early clinical symptoms are atypical or inexistent, making it impossible to diagnose and treat the patient on time. Consequently, most patients are already at an advanced stage when diagnosed with the disease and present obvious clinical symptoms. Therefore, they miss the best treatment opportunities and lack effective treatment measures, resulting in an unsatisfactory prognosis [2, 3]. Given this, we have to seek more accurate and effective prognostic markers for the stratification of patients and the formulation of subsequent clinical treatment schemes. With the development of bioinformatics and sequencing technology, an increasing number of researchers studied the potential prognostic evaluation scheme for ovarian cancer patients [4–6]. However, most of the analysis parameters in these studies come from a genome or transcriptome and lack an analysis of multi-omics. Thus, these models cannot effectively display ovarian cancer's features.

Gene expression is strictly and complexly regulated in living organisms. Abnormal gene expression occurs when a tumor develops. The key role of the epigenetic mechanism in regulating the genetic transcription expression has drawn more and more attention. Epigenetics involves the regulation of gene expression without changing the genome sequence [7]. DNA methylation is a widely studied epigenetic modification that regulates gene expression and chromatin conformation, along with histone modification. DNA methylation bears the maintenance of normal cell function, the stability of genome structure, embryonic development, and the occurrence and development of tumors [8, 9]. Several studies [10, 11] have constructed prognostic models of DNA methylation in multiple ovarian cancer patients, which could revolutionize clinical application. However, they only analyzed the data of DNA methylation and omitted the subsequent transcriptomics. Based on multi-platform data, it is more conducive to integrate and analyze data from multiple sources—including transcriptome, DNA methylation, and clinical results—to explore specific events of carcinogenesis and determine potential prognostic models related to patients.

In this study, we identified the molecular subtypes of ovarian cancer and evaluated their relationship with patients' prognosis and clinical features by analyzing the DNA methylation and transcriptome of ovarian cancer patients. The 8-gene signature constructed based on Differentially Methylated Genes (DMEG) genes could efficiently and stably predict ovarian cancer patients' prognoses and immunotherapy responses.

## 2. Methodology

### 2.1. Data Download and Preprocessing

The expression data, including methylation data (27K), SNV/Indel data, CNV data, and clinical follow-up information, of ovarian cancer (OV) patients were downloaded from TCGA. The GEO data were downloaded from Gene Expression Omnibus (GEO) and GSE17260 with overall survival (OS) was selected.

The following were performed on methylation data (27K) gathered from TCGA of OV patients:

1. The KNN function in the *R* package impute was used to complement the NA values.
2. The cross-reactive sites in CpG tagged by the discovery of cross-reactive probes and polymorphic CpGs in the Illumina Infinium Human Methylation 450 microarray were removed.
3. Unstable genomic DNA methylation sites were removed.
4. Solid tumor samples were retained.

On the RNA-Seq data from TCGA of OV patients:

1. Using log2 transformation, expression profile FPKM data were converted to TPM data.
2. Ensemble was converted to gene symbol.
3. The median value was taken in the case of expression with multiple gene symbols.

The following steps performed on the data set of GSE17260 were as follows:

1. Samples without OS and survival state were removed.
2. Probes were converted to gene symbols.
3. The probe was removed when it corresponded to more than one gene.
4. The median value was taken in the case of expression with multiple gene symbols.
5. Only the expression profile data of OV were retained.

After preprocessing the three sets of data, a total of 567 samples of methylation data were available in TCGA-OV-366 samples had expression profile data. The GSE17260 cohort contained 110 samples.

### 2.2. Identification of 74Molecular Subtype of Methylation Data

Univariate cox analysis through coxph function in *R* was used to preprocess the TCGA methylation data (beta values) and the results were used to obtain the methylation sites that correlated with OV prognosis (*P* < 0.05). Following the extraction of data of the methylation sites, the TCGA samples were clustered consistently using ConsensusClusterPlus (V1.48.0; parameters: reps = 100, pItem = 0.8, pFeature = 1, distance = “euclidean”). *D*2 and Euclidean distance were used as clustering algorithms and the distance metric was used to select and analyze the optimal grouped subtype, depending on survival prognosis, clinical correlation, and mutation and amplification between different subtypes.

### 2.3. Identification of Differentially Methylated Genes (DMGs) and Differentially Expressed Genes (DEGs) between Molecular Subtypes

The minfi package (V1.30.0) was used to calculate differentially methylated sites between molecular subtypes.

Match files between CpG sites and genes were first downloaded from the Illumina website (https://www.illumina.com/) and the average *β*-values of genes within different regions (TSS1500, TSS200, 5′-UTR—5′-untranslated region, first exon, gene body, 3′-UTR—3′-untranslated region, and intergenic region, TSS—transcriptional start site) were calculated according to the correspondence. In addition, the *R* software package clusterProfiler (v3.14.0) was used to perform KEGG pathway analysis of Hypermethylation genes and Hypomethylation genes for OV grouped subtype. Then, the DEGs between molecular subtypes were calculated using the limma package, and KEGG pathway analysis was performed on the upregulated and downregulated genes of OV grouped subtype by the *R* package clusterProfiler (v3.14.0). The Venn diagrams of DMGs with DEGs were plotted as the last step.

### 2.4. Construction of a Polygenic Prognostic Model

#### 2.4.1. Random Grouping

The 366 samples with expression profiles in the TCGA cohort were first divided into a training set and a validation set to circumvent the random assignment bias affecting the stability of subsequent modeling. All samples were randomly grouped 100 times with replacement in advance, where group sampling was performed in the ratio of training set: validation set = 1 : 1. The most suitable training and validation sets were selected based on the following conditions: (1) The two groups had similarities in age distribution, gender, follow-up time, and percentage of patient deaths. (2) There was a close association in the number of dichotomous samples after gene expression profile clustering amongst the two randomly grouped cohorts.

#### 2.4.2. Univariate and Lasso Regression Analysis in Training Set

Using the training set data, a univariate cox proportional hazard regression model was constructed for molecular subtypes of DMGs, where the identified *P* < 0.05 was selected as a threshold for filtering and prognosticating genes.

The Lasso method (a shrinkage estimate) obtains a refined model by constructing a penalty function to shrink some coefficients while setting some coefficients to zero, thereby having the advantage of subset shrinkage and biased estimation dealing with multicollinearity data that can select variables to better solve the problem of multicollinearity in regression analysis. The *R* package glmnet for Lasso Cox regression was performed to shrink genes and to reduce the number of genes in the risk model.

### 2.5. Gene Set Variation Analysis

Gene Set Variation Analysis (GSVA) is a nonparametric, unsupervised method for estimating variation of gene set enrichment through the samples of an expression data set. GSVA performs a change in coordinate systems, transforming the data from a gene by sample matrix to a gene-set by sample matrix, thereby allowing the evaluation of pathway enrichment for each sample.

In this research, the relationship between RiskScores and biological functions of different samples were observed through the selection of gene expression profiles for single-sample GSEA (ssGSEA) analysis using the *R* package GSVA.

### 2.6. Clinical Tissues and Real-Time PCR

Ovarian cancer tissues (*n* = 20) and paired normal ovarian tissues (*n* = 20) were obtained from the Shengjing Hospital of China Medical University. The ovarian cancer sample information was presented in Table 1. RNA was extracted from frozen tissues using TRIzol reagent (15596018, Invitrogen). RNA was reverse-transcribed into cDNA and quantified to real-time PCR analyses, and the levels were normalized to GAPDH levels. The primers were as follows, the forward primer of TCF15: 5′-CAGCTGCTTGAAAGTGAGGG-3′; the reverse primer of TCF15: 5′-TCCTCCGGTCCTTACACAAC-3′; the forward primer of TCIRG1: 5′-CTGGATGATGAAGAGGAGGCCGA-3′; the reverse primer of TCIRG1: 5′-CCCTAGTCATCTGTGGCAGCGAA-3'; the forward primer of TMPRSS3: 5'-AGTGGGGTAGACGGAGACCT-3′; the reverse primer of TMPRSS3: 5′-CACTGAACCCTTCCTGGTTT-3′; the forward primer of DMC1: 5′-AATGGCACTTTTTCG AGTGG-3′; the reverse primer of DMC1: 5′-CAGGCATCTCAGGACTGTCA-3′; the forward primer of HLADOB: 5′-ATCTGACCCGACTGGATTCCT-3′; the reverse primer of HLADOB: 5′-GCACCTTTTCTGTCCCGTTG-3′; the forward primer of NPY: 5′-TCACCAGGCAGA GATATGGA-3′; the reverse primer of NPY: 5′-GCAAGTCTCATTTCCCATCA-3′; the forward primer of ARPC1B: 5′-CAAGGACCGCACCCAGATT-3′; the reverse primer of ARPC1B: 5′-TGCCGCAGGTCACAATACG-3′; the forward primer of ACSS3: 5′-CCGGTCGTGACCTTGATTGG-3′; the reverse primer of ACSS3: 5′-CGTTGTGCCAGATGTGTAAAGA-3′.

### 2.7. In Vitro Experiments

#### 2.7.1. Cell Culture and Transfection

Ovarian cancer cells, OVCAR3, CAOV3, and SKOV3 were purchased from the Chinese Academy of Medical Sciences and the CAMS & PUMC Medical College (Beijing, China). The normal ovarian cell line (IOSE-80) was obtained from Shanghai Yaji Biotechnology Co., Ltd. The cell lines were cultured in 1640 medium supplemented with 10% fetal bovine serum and 100 units/ml penicillin at 37°C in a humidified 5% CO_2_ incubator. Lipofectamine 2000 siRNA (small interfering RNA) sequence transfection protocol (Invitrogen, Shanghai, China) was performed in this study.

#### 2.7.2. Cell Viability Assay

Cells (1500/well) were added to a 96-well culture plate and transfected with NC-siRNA, TCIRG1-siRNA. After 0, 24, 48, or 72 h, and cultured with 20 *μ*l of CCK8 solution for another 2 h, cell viability is expressed as an optical density (OD) value at 450 nm.

#### 2.7.3. Colony Formation Assay

To explore the effects of TCIRG1 expression on cell proliferation, cells (1000/well) transfected with NC-siRNA or siRNA were added into each well of 6-well culture plates for two weeks. The number of colonies in each well was counted.

#### 2.7.4. Western Blotting

Total protein was extracted from cells using RIPA buffer. Proteins (30 *μ*g/lane) were separated via SDS-PAGE and subsequently transferred to PVDF membranes. Following blocking with 5% skimmed milk at room temperature, the membranes were incubated at 4°C overnight with primary antibodies. Subsequently, the membranes were incubated with HRP-conjugated secondary antibodies at room temperature for 30 min. Protein bands were visualized using Pierce™ ECL Western Blotting Substrate (Pierce; Thermo Fisher Scientific, Inc.).

## 3. Results

### 3.1. Identification of Molecular Subtype of Methylation Data

The 1053 (S1_Table) methylation data from DNA methylation sites were extracted through Univariate Cox analysis and correlated with OV prognosis (*P* < 0.05). The TCGA samples were also clustered using *ConsensusClusterPlus*.

**Figure 1:**
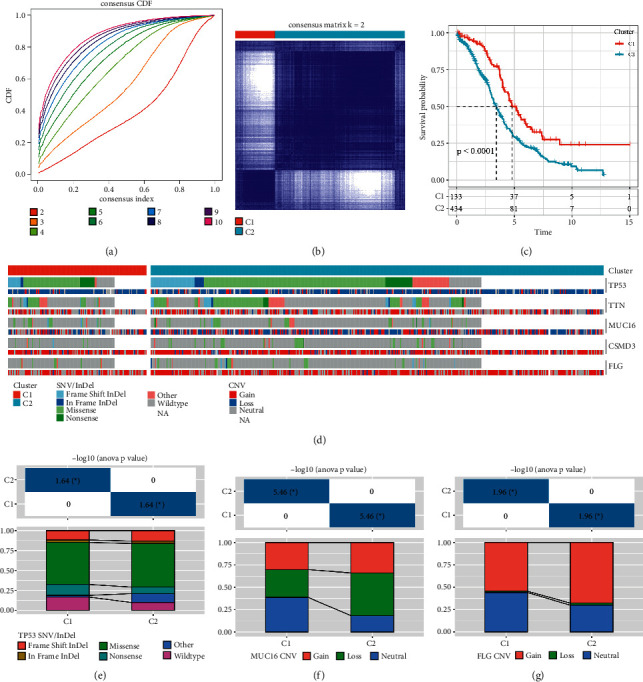
(a) The cumulative distribution function; (b) sample clustering for consensus cluster plus clustering (*k* = 2), the red means cluster 1, and the blue means the cluster 2; (c) OS prognostic survival curves for OV molecular subtypes; (d) distribution of mutations between OV molecular subtypes; (e) distribution of TP53 molecular mutations between molecular subtypes; (f) distribution of CNV in MUC16 between molecular subtypes; (g) distribution of CNV in FLG between molecular subtypes ^*∗*^*P* < 0.05.

The results showed that the samples were clustered into two major groups at *k* = 2 (Figures 1(a) and 1(b), S2_Table) and the prognostic relationship between the two groups showed a difference between *C*1 and *C*2 (Figure 1(c), log rank *P* < 0.0001), with the *C*2 subtype having a worse prognosis.

A molecular event, such as TP53 mutation, is a driver of metabolic reprogramming in OVs. To determine the carcinogenic factors among molecular subtypes, the distribution of genes frequently mutated in OV between SNV/InDel and CNV affected metabolic subtypes were investigated (Figure 1(d)). The difference between *C*1 and *C*2 in terms of MUC16 amplification and deletion, FLG amplification, and mutation frequency of TP53 was that it was higher in the *C*2 group than in the *C*1 group (Figures 1(e)–1(g)).

### 3.2. Identification of Differentially Methylated Genes (DMGs) between Molecular Subtypes

The minfi package (V1.30.0) was used to calculate the differentially methylated sites between *C*2 and *C*1 molecular subtypes and a total of 2728 differentially methylated sites were found after using the threshold FDR <0.05 and |FC| >1.2. The *C*2 molecular subtypes were subdivided into 1350 sites in hypermethylation state and 1378 in hypomethylation state, as shown in S3_Table.

A total of 1877 CpGb were matched to the corresponding genes (S4_Table), where 742 genes were Hypermethylated, and 921 genes were Hypomethylated (S5_Table). The distribution of CpG sites and methylation state within different regions were shown in Supplementary Figures 1(a) and 1(b).

The regions of differential methylation between *C*2 and *C*1 subtypes concentrated in decreasing order are as follows: the TSS1500 region, TSS200 region, and Body region. The Hypermethylation sites in different regions compared to Hypomethylation sites were not much different in terms of number. (The proportion of Hypomethylation sites in the TSS1500 region was higher than that of Hypermethylation sites.)

KEGG pathway analysis was conducted by the *R* software package *clusterProfiler* (v3.14.0) on 742 Hypermethylation genes in the OV subtype group. The top 10 annotations depicted in Supplementary Figure 1(c), showed significant pathways: NOD-like receptor signaling pathway, Rap1 signaling pathway, and other pathways.

The KEGG pathway analysis was then performed on 921 Hypomethylation genes in the OV subtype group by the *R* software package clusterProfiler (v3.14.0). Some of the annotation results were significant such as PI3K-Akt signaling pathway, ECM-receptor interaction, and cAMP signaling pathway (Supplementary Figure 1(d)).

### 3.3. Identification of Differentially Expressed Genes (DEGs) between Molecular Subtypes

**Figure 2:**
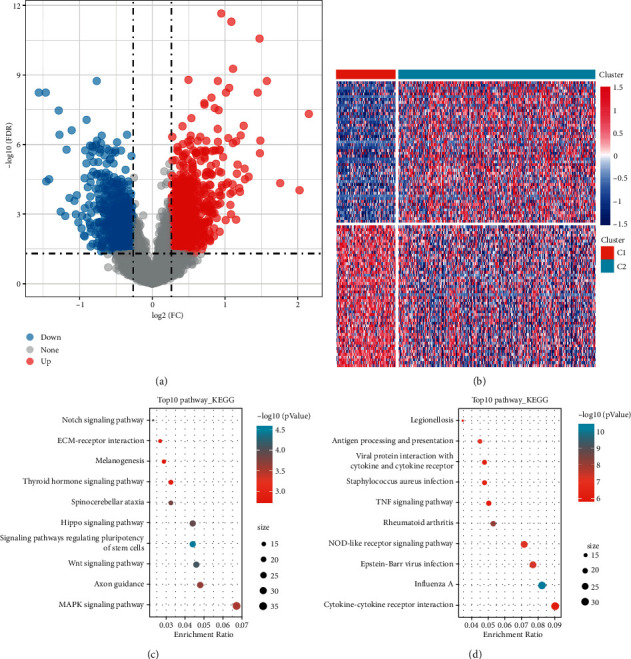
(a) Volcano plot of differentially expressed genes in *C*1 and *C*2 groups; (b) heat map of differentially expressed genes in *C*1 and *C*2 groups; (c) KEGG annotation plot of differentially upregulated expressed genes in molecular subtypes; (d) KEGG annotation plot of differentially downregulated expressed genes in molecular subtypes.

The limma package used to calculate the DEGs between *C*2 and *C*1 molecular subtypes used filtering according to the threshold FDR <0.05 and |FC| >1.2 d and obtained a total of 2213 DEGs (S6_Table), 1399 of which were upregulated and 814 were downregulated. The volcano plot illustrates the upregulation and downregulation of DEGs as shown in Figure 2(a). The heat map of the 100 most differentially upregulated and downregulated genes was plotted, as shown in Figure 2(b). Furthermore, the KEGG pathway analysis of 1399 differentially upregulated genes in the OV subtype group was performed by the *R* software package clusterProfiler (v3.14.0) and annotated 37 significantly enriched pathways (Figure 2(c), *P* < 0.05), with genes significantly enriched in MAPK signaling pathway, Wnt signaling pathway, Notch signaling pathway, and other pathways. The KEGG pathway analysis was also conducted on 814 differentially downregulated genes in OV patients, and 74 significantly enriched pathways were annotated (Figure 2(d), *P* < 0.05), with genes significantly enriched in the TNF signaling pathway, NOD-like receptor signaling pathway, and other pathways.

### 3.4. Identification of Differentially Methylated Expression Genes (DMEGs) and Functional Analysis

**Figure 3:**
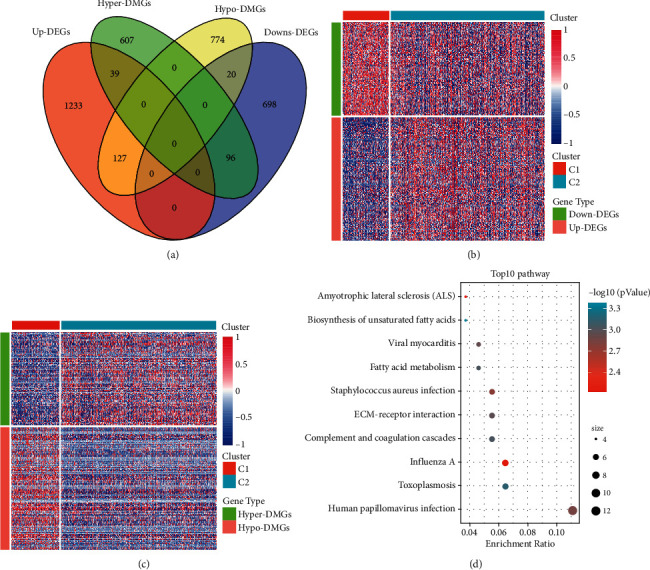
(a) Venn diagram of DMGs and DEGs; (b) heat map of DMEGs in molecular subtypes on expression profile data; (c) heat map of DMEGs in molecular subtypes on methylation data; (d) KEGG annotation map of DMEG in molecular subtypes.

The relationship between the degree of gene methylation and gene expression are inversely proportional where the higher the methylation, the lower is the gene expression, and vice versa. Venn diagrams of DMGs and DEGs in Figure 3(a) showed that 96 genes were hypermethylated and down regulated, 127 genes were hypomethylated and upregulated, totaling 223 genes that were defined as DMEGs. The mapping of these DMEGs also revealed low expression levels of hypermethylated genes and high expression levels of hypomethylated genes (Figures 3(a)–3(c)).

Both the KEGG pathway analysis of the 223 DMEGs in the OV subtype group showed enrichment of genes in *Fatty acid metabolism*, *ECM-receptor interaction*, *Human papillomavirus infection*, and other pathways (Figure 3(d)).

### 3.5. Construction of a Prognostic Hazard Model Based on Differentially Methylated Expression Genes (DMEGs)

#### 3.5.1. Random Grouping of Training Set Samples

It was written in the methodology section that 183 samples had both the final training set data and test set data. Table 2 is the detailed training set and validation set sample information that was tested using the chi-square test and results showed no preference in groupings and no significant difference between the groups (*P* > 0.05).

#### 3.5.2. Construction and Evaluation of Risk Model

Univariate Cox proportional hazard regression models were constructed using the training set data for each *C*1 and *C*2 molecular subtype of DMEGs (223 in total) and survival data were made using the *R* package survival coxph function with a threshold for filtering of *P* < 0.05 selected. The results of univariate cox analysis are shown in S7_Table. A total of 10 prognostic DMEGs were identified, yet many genes make it nonconductive to clinical testing and therefore there is still the need to further narrow down the number of genes.

**Figure 4:**
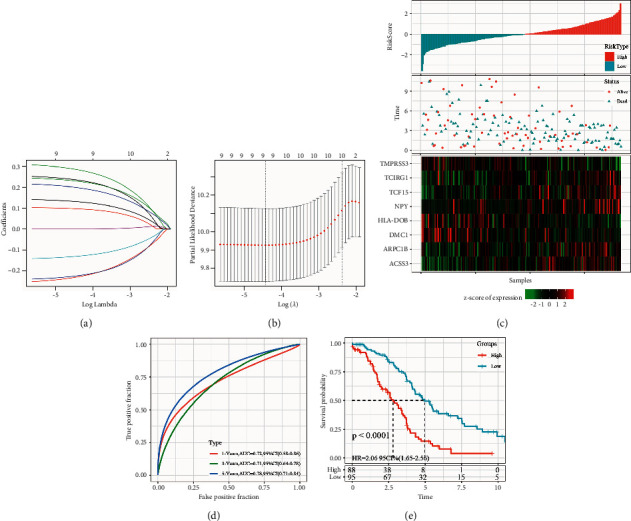
(a) Trajectory of each independent variable where the horizontal axis represents the log value of the independent variable lambda, while the vertical axis indicates the coefficient of the independent variable; (b) confidence interval under each lambda. (c) riskScore, OS, survival status, and expression of 8-gene in TCGA training set; (d) ROC curve of 8-gene signature classification and AUC; (e) KM survival curve distribution of 8-gene signature in training set.

The *R* package *glmnet* was used to perform Lasso Cox regression. The first step was the analysis of the trajectory of each independent variable shown in Figure 4(a), where it is seen that as the lambda gradually increases, the number of independent variable coefficients tending to zero also gradually increases. The model was then constructed using 5-foldcross-validation, and the confidence intervals under each lambda were analyzed as shown in Figure 4(b). When the 9 genes are at lambda = 0.01195682, the model has reached optimum and has become a target gene for further analysis.

The Akaike Information Criterion (AIC) method in the MASS package started with the most complex model and sequentially removed one variable to reduce the AIC. The smaller value indicates a better and sufficient model having fewer parameters. Using this algorithm, the 9 genes were finally reduced to 8 genes, namely: ARPC1B, DMC1, TCIRG1, TMPRSS3, HLA-DOB, NPY, TCF15, and ACSS3. The formula of the final 8-gene signature is as follows: RiskScore = 0.207 *∗* ARPC1B − 0.284 *∗* DMC1 + 0.318 *∗* TCIRG1 − 0.271 *∗* TMPRSS3 − 0.180 *∗* (HLA-DOB) + 0.152 *∗* NPY + 0.262 *∗* TCF15 + 0.253 *∗* ACSS3.

The RiskScore of each sample calculated showed that samples with high RiskScores have a worse prognosis, while the RiskScore distributions were plotted and are shown in Figure 4(c). The changes in expression of 8 different signature genes were analyzed, where high expression of ARPC1B, TCIRG1, NPY, TCF15, and ACSS3 correlated with a higher risk while high expression of TMPRSS3, HLA-DOB, and DMC1 correlated with a lower risk. A ROC analysis of the prognostic classification of RiskScores was then performed using the *R* package time ROC, and the prognostic classification efficiency at one year, three years, and five years was analyzed, respectively, as shown in Figure 4(d). Finally, the zscore of RiskScore was done, and samples with RiskScores greater than zero were classified as a high-risk group, while those with less than zero were classified as a low-risk group, on the basis of KM curves plotted in Figure 4(e). The 88 samples classified as a high-risk group and 95 samples classified as a low-risk group had a significant difference of *P* < 0.0001.

### 3.6. Validation of the Risk Model

#### 3.6.1. Robustness of 8-Gene Signature Verified by Internal Cohort

The robustness of the model was determined by the training set used to calculate the RiskScore for each sample in the entire TCGA cohort.

The distribution of RiskScore for the entire TCGA cohort in Supplementary Figure 2(a) shows that the samples with high RiskScores have a worse prognosis than in the low RiskScore group. The ROC analysis of RiskScore was performed and the prognostic classification efficiency at one year, three years, and five years were analyzed, respectively (Supplementary Figure 2(b)). Finally, the zscore of RiskScore was performed and 181 samples with RiskScore greater than zero were classified as a high-risk group while 185 samples with less than zero were classified as a low-risk group, based on the KM curves plotted as shown in Supplementary Figure 2(c). There was a highly significant difference of *P* < 0.0001.

#### 3.6.2. Robustness of 8-Gene Signature Verified by External Cohort

The same models were used in the external validation set GSE17260 as in the training set. The distribution of RiskScore for the independent validation cohort in Supplementary Figure 2(d) shows that high RiskScores samples have a worse prognosis than low RiskScores samples, which is consistent with the performance on the training set. The prognostic classification efficiency at one year, three years and five years was analyzed respectively, as shown in Supplementary Figure 2(e) and the KM curves between high and low RiskScore groups were plotted as shown in Supplementary Figure 2(f). A significant difference (*P* < 0.001) between 50 samples classified as a high-risk group and 60 samples classified as a low-risk group was observed.

### 3.7. Risk Model and Prognostic Analysis of Clinical Features

**Figure 5:**
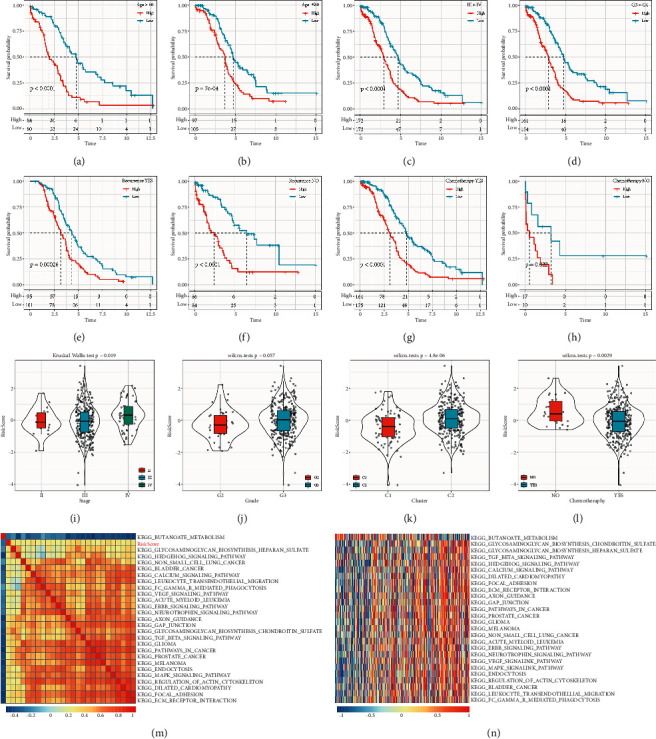
Performance of the risk model on different clinical features. (a)–(h) Based on the expression of RiskScore, analyze the overall survival of different clinical subgroups; (i) comparison of RiskScores between groups in terms of stage; (j) comparison of RiskScores between groups in terms of grade 2 + 3; (k) comparison of RiskScores between groups in terms of cluster; (l) comparison of RiskScores between groups in terms of chemotherapy conditions; (m) correlation coefficient clustering between KEGG pathways with correlations greater than 0.25; (n) relationship between ssGSEA scores of KEGG pathways with correlation greater than 0.25 and increasing RiskScores in each sample, where the horizontal axis represents the samples with increasing RiskScores from left to right.

The performance of RiskScore analysis on the 8-gene signature found that the expression of RiskScore, Age, Stage III + IV, recurrence, and chemotherapy can be categorized into two groups either having high-risk prognosis or low-risk prognosis (Figures 5(a)–5(h), *P* < 0.05), and is suggestive that our model has good predictive ability across different clinical features.

The distribution of RiskScores between clinical feature groups was compared and resulted in a significant difference (*P* < 0.05) in terms of Stage, Cluster, and Chemotherapy group. The RiskScore was marginally significant (*P* ≈ 0.05) between Grade 2 + 3 groups proving that the more advanced the stage is, the higher the RiskScore would be. The RiskScore of the nonchemotherapy sample was significantly higher than the chemotherapy sample and the higher RiskScore in the *C*2 group correlates to a worse prognosis compared to the *C*1 cluster group (Figures 5(i)–5(l)).

The relationship between RiskScores and biological functions of different samples were observed through the selection of gene expression profiles for single-sample GSEA (ssGSEA) analysis using the *R* package GSVA, thus resulting in the ssGSEA scores of each sample. The correlation between these functions and the RiskScore was further calculated, Figure 5(m) indicates that 25 types of functions have a positive correlation greater than 0.25 with the RiskScore of the sample, and only 1 showed a negative correlation with the RiskScore. The top 26 most relevant KEGG pathways were selected and clustered according to their enrichment scores as shown in Figure 5(n), which had increasing scores. The 26 KEGG pathways are KEGG_NON_SMALL_CELL_LUNG_CANCER, KEGG_MAPK_SIGNALING_PATHWAY, KEGG_BLADDER_CANCER, KEGG_FOCAL_ADHESION, KEGG_TGF_BETA_SIGNALING_PATHWAY and other tumor-related pathways.

### 3.8. Univariate and Multivariate Analysis of 8-Gene Signature

**Figure 6:**
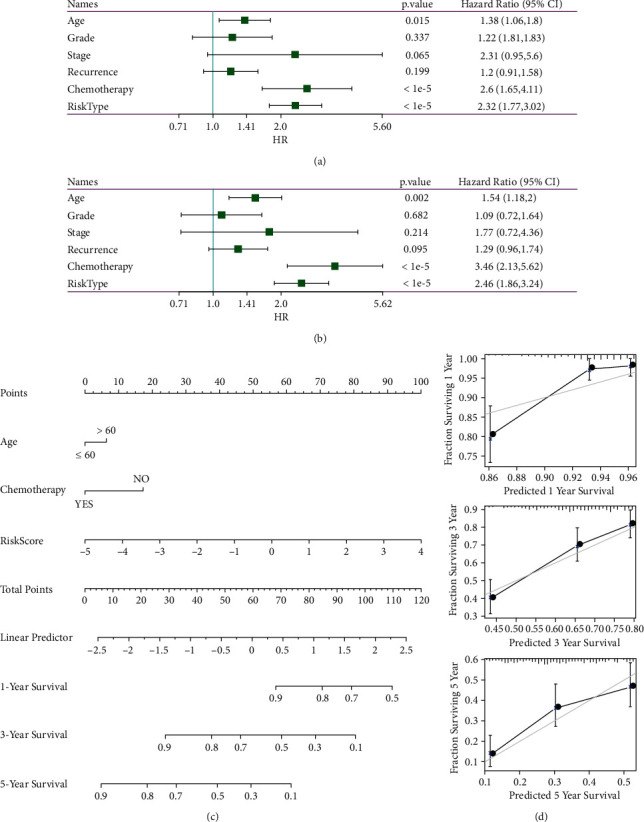
(a) Univariate analysis results of clinical features with RiskScore; (b) multivariate analysis results of clinical features with RiskScore; (c) nomogram constructed by Age + Chemotherapy + RiskScore; (d) calibration plot of nomogram survival rate.

The independence of the 8-gene signature model in clinical applications was identified by performing both univariate and multivariate COX regression analyses on the RiskScore and clinical variables. The data showed that in the TCGA cohort, both the univariate COX regression analysis (Figure 6(a)) and multivariate COX regression analysis found that the RiskType (HR = 2.46, 95% CI = 1.86–3.24, *P* < 1*e* − 5) (Figure 6(b)) were correlated with survival, thus concluding the independent predictive performance of the 8-gene signature in terms of clinical application value.

Nomogram is a visual way to present the results of risk models and make predictions of outcomes. The multivariate significant clinical features, such as Age, Chemotherapy, and RiskScore, were combined to construct the nomogram model (Figure 6(c)). The model results showed the RiskScore feature had the greatest impact on survival prediction and the risk model based on 8-gene can better predict the prognosis. Calibration of nomogram data for 1, 3, and 5 years was also done to visualize the good performance of the nomogram (Figure 6(d)).

### 3.9. Comparison of Risk Models with Others and Prediction of Immunotherapy

**Figure 7:**
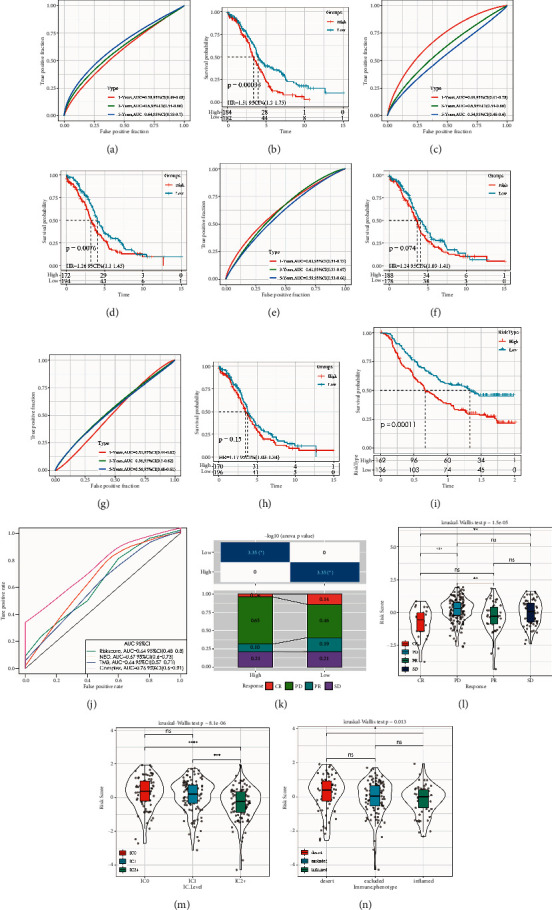
(a) (b) ROC and OVKM curves of high-risk and low-risk groups for the 10-gene signature (Wang) risk model; (c) (d) ROC and OVKM curves of high-risk and low-risk groups for the 7-gene signature (Sabatier) risk model; (e) (f) ROC and OVKM curves of high-risk and low-risk groups for the 5-gene signature (Wang) risk model; (g) (h) ROC and OVKM curves of high-risk and low-risk groups for the 8-gene signature (Yue) risk model. (i) KM curves of IMvigor 210 cohort; (j) ROC curves of IMvigor 210 cohort; (k) corresponding stacked plots of immunotherapy between different groups of IMvigor 210 cohort; (l) differences in RiskScore between the effectiveness groups of immunotherapy; (m) differences in RiskScore between immune cell groups; (n) differences in RiskScore between immune phenotype groups. (IC: immune cell; TC: tumor cell; IP: immune phenotype); ^*∗*^*P* < 0.05.

The literature review selected five prognosis-related risk models, namely, the: 10-gene signature (Figures 7(a) and 7(b)) [12],7-gene signature (Figures 7(c) and 7(d)) (Sabatier et al. 2011), 5-gene signature (Figures 7(e) and 7(f)) [13], and 8-gene signature (Figures 7(g) and 7(h)) [14], and the models were used as a comparison with our 8-gene model.

**Figure 8:**
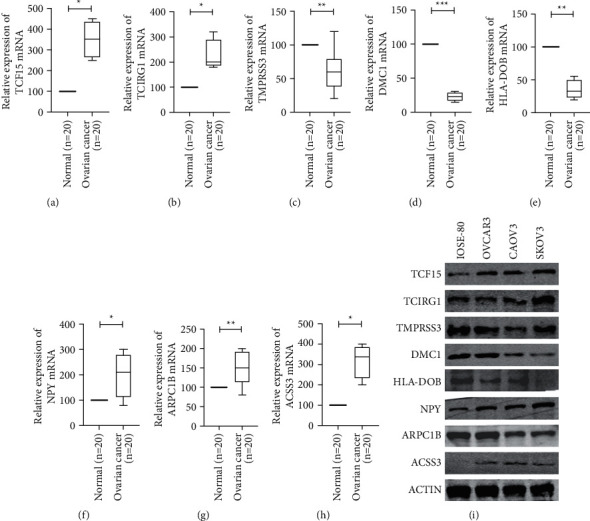
The mRNA expression of 8 genes in ovarian cancer tissues (*n* = 20) and paired normal ovarian tissues (*n* = 20). (a) TCF15; (b) TCIRG1; (c) TMPRSS3; (d) DMC1; (e) HLA-DOB; (f) NPY; (g) ARPC1B; (h) ACSS3; (i) protein expression of 8 genes in ovarian cancer cell lines (OVCAR3, CAOV3, and SKOV3) and normal ovarian cell line (IOSE-80) ^*∗*^*P* < 0.05, ^*∗∗*^*P* < 0.01 and ^*∗∗*^*P* < 0.001.

To make the models comparable, the RiskScore of each OV sample in TCGA was calculated according to the corresponding genes in these five models using the same method. The Zscore method revealed that samples greater than zero were classified as the high-risk group, while samples less than zero were classified as the low-risk group, based on the calculated prognostic difference. The ROC and KM curves of the five models in Figures 7(a)–7(h) show that the AUC values of these five models at 1, 3, and 5 years are lower than those of our model. The prognosis of OV in the high-risk and low-risk groups with 5-gene signature (Figure 7(f)) and 8-gene signature (Figure 7(h)) in these five models was not statistically different (log rank *P* > 0.05).

### 3.10. Prediction of Immunotherapy by Risk Models

Effective predictive markers for immunotherapy are currently limited and the identification of novel predictive markers is critical to further advance precision in immunotherapy. In this research, an immunotherapy cohort (Imvigor210) containing transcriptomic data was retrieved to explore whether the 8-gene model could predict the benefit of immunotherapy. Imvigor 210 recorded expression data in human mUC samples from patients who responded or failed to respond to anti-PD-L1 immunotherapy. Kaplan–Meier curves showed that the higher RiskScore values, the poorer survival in mUC patients treated with immunotherapy (Figure 7(i)), and the ROC curves indicated that RiskScore has a higher AUC value (Figure 7(j)). There was a significant difference between immunotherapy response and nonresponse between the high-risk and low-risk groups, highlighting a smaller proportion of the high-risk subgroup responding to immunotherapy (Figure 7(k)).

In addition, a comparison of the differences between RiskScores of different groups was done and showed that RiskScore was significantly different from the: immunotherapy effectiveness group, immune cell group, and immune phenotype group (Figures 7(l)–7(n)), in which patients in the desert group had higher RiskScore expression than inflamed immune inflammation group. Patients having a higher tumor immune infiltration and belonging in the low RiskScore group would mean a better prognosis than those in the high RiskScore group.

### 3.11. Protein and mRNA Expression of 8 Genes in Ovarian Cancer

To clarify the role of 8-gene signature in the occurrence and development of ovarian cancer, we analyzed the mRNA expression levels of 8 genes in ovarian cancer tissues and normal ovarian tissues based on the PCR analysis. In Figures 8(a)–8(h), the expression levels of *TCF15, TCIRG1, NPY, ARPC1B,* and *ACSS3* in ovarian cancer tissues are significantly higher than that of normal ovarian tissues. And the expression levels of *TMPRSS3, DMC1,* and *HLA-DO* in ovarian cancer tissue are significantly lower than those in normal ovarian tissue.

Furthermore, the protein expression levels of 8 genes in ovarian cancer cell lines and normal ovarian cell lines were analyzed based on the western blot analysis. In Figure 8(i), the protein expression levels of TCF15, TCIRG1, NPY, ARPC1B, and ACSS3 in OVCAR3, CAOV3, and SKOV3 cells are significantly higher than that of IOSE-80 cells. And the expression levels of TMPRSS3, DMC1, and HLA-DOB in OVCAR3, CAOV3, and SKOV3 are significantly lower than those in IOSE-80 cells.

### 3.12. Function Analysis of TCIRG1 in Ovarian Cancer

**Figure 9:**
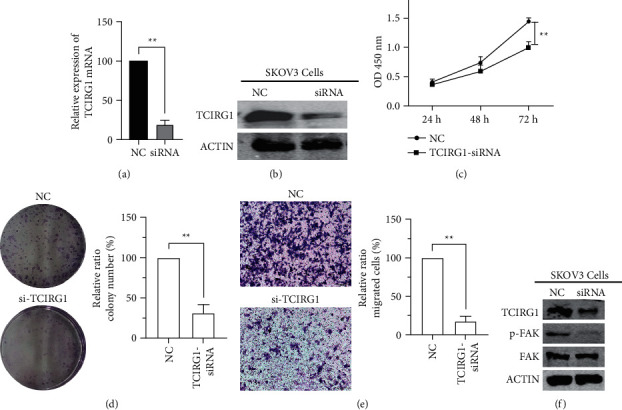
Function analysis of TCIRG1 in SKOV3 cells. TCIRG1 expression levels were detected by qRT-PCR (a) and western blotting (b) analyses after transfection with negative control (NC) or TCIRG1-siRNA; (c) Cell viability assays showed that TCIRG1 knockdown decreased SKOV3cell proliferation; (d) the number of colonies formed by SKOV3 cells transfected with TCIRG1‐siRNA was lower than that for cells transfected with NC; (e) transwell assay showed that the TCIRG1-siRNA-transfected group had significantly fewer migrated cells than the NC-siRNA-transfected group; (f) western blot analysis showed the expression of p-FAK was significantly decreased after transfected with TCIRG1-siRNA^*∗*^*P* < 0.05, ^*∗∗*^*P* < 0.01 and ^*∗∗*^*P* < 0.001.

TCIRG1 was selected as high priority markers for next study because it showed the higher coefficient in genetic models, and there are currently few studies in ovarian cancer. SKOV3 cell line was chosen for experiments as it has the highest TCIRG1 expression. Knockdown of TCIRG1 expression was performed using siRNA. Transfection efficiency was measured using qRT–PCR and western blot analyses (Figures 9(a) and 9(b)). CCK-8 assays revealed that knockdown of TCIRG1 significantly decreased the proliferation of SKOV3 cells (Figure 9(c)). Moreover, the TCIRG1-siRNA-transfected group had significantly fewer colonies than the NC-siRNA-transfected group (Figure 9(d)). Transwell assay showed that the TCIRG1-siRNA-transfected group had significantly fewer migrated cells than the NC-siRNA-transfected group (Figure 9(e)). The results in Figure 5(n) showed that RiskScore is significantly positively correlated with the KEGG_FOCAL_ADHESION pathway. Therefore, we tried to explore whether TCIRG1 regulates the expression of FAK. In Figure 9(f), the expression of p-FAK was significantly deceased after being transfected with TCIRG1-siRNA. Taken together, these results demonstrated that TCIRG1 knockdown could decrease the proliferation ability of ovarian cancer cells in vitro.

## 4. Discussion

Due to the lack of early detection means, 70% of patients with ovarian cancer (OV) are in advanced stages by the time they get diagnosed [15]. Distant metastasis is the leading cause of death in patients with OV [16, 17] and conventional clinical staging still fails to predict the survival rate of individual patients. The rapid development of omics sequencing technology has allowed researchers to analyze the mechanism of OV progression through large-scale gene expression data and clinical information. Aberrant DNA methylation is the most widely studied deregulatory epigenetic mechanism in tumors [18], making it valuable in evaluating OV patients' prognoses.

In this study, we divided OV samples into two subtypes, *C*1 and *C*2, based on methylation data from The Cancer Genome Atlas Ovarian Cancer (TCGA-OV). The subtypes showed differences in prognosis. We also noticed contrasts in the mutation and copy number variation (CNV) distribution of molecular subtypes. The immune score of the *C*1 subtype was higher than that of *C*2, which may help explain the accurate prognosis of *C*1. Based on the further screening of 223 DMEG genes, we noted a correlation between these genes and fatty acid metabolism and ECM-receptor interactions. The fatty acid metabolism and ECM-receptor interaction corresponded with the malignant progression of OV, and the activation of both promoted the proliferation and invasive capacity of OV cells [19–22]. These results suggest the involvement of a regulated metabolism of DNA methylation and an extracellular matrix in the progression of OV. Using these 223 DMEGs, we created an 8-gene signature that predicted efficiently and consistently across different platform cohorts (The International Cancer Genome Consortium, GSE17260, and The Cancer Genome Atlas). More importantly, the constructed gene models performed well on the immunotherapy cohort, IMvigor 210. In summary, the fabricated 8-gene signature determines the prognosis of patients with OV accurately and contributes to the precise application of immunotherapy.

The 8-gene signature screened and established in this paper includes Actin-Related Protein 2/3 Complex Subunit 1*B* (ARPC1B), Meiotic recombination protein DMC1, *T* Cell Immune Regulator 1 (TCIRG1), Transmembrane Protease, Serine 3 (TMPRSS3), HLA-DOB, Neuropeptide Y (NPY), Transcription Factor 15 (TCF15), and Acyl-CoA Synthetase Short Chain Family Member 3 (ACSS3). The protein encoded by ARPC1B is one of the subunits of the Arp2/3 protein complex, while the deletion and mutation of the ARPC1B gene disrupt the development of platelets and *T*-lymphocytes, [23] and leads to a combined immune deficiency encompassing severe infections, inflammations, and allergies [24]. In terms of tumors, ARPC1B was identified as a prognostic marker in oral squamous cell carcinoma [25] and melanoma [26]. The protein encoded by DMC1, a member of the Recombinases superfamily, is essential to repair double-stranded DNA breaks during mitosis and meiosis [27, 28]. It is also strongly correlated with the prognosis of patients with endometrial cancer [29] and is downregulated in OV tissues [30]. The protein encoded by TCIRG1 is a subunit of H+-ATPase, and a prognostic marker and immune infiltration marker in patients with glioma [31]. According to hepatocellular carcinoma studies, however, TCIRG1 promotes the proliferation and epithelial-mesenchymal transition (EMT) of hepatocellular carcinoma cells and is a poor prognostic factor for patients with hepatocellular carcinoma. The protein encoded by TMPRSS belongs to the serine protease family, a predominant factor in cancer progression and metastasis [32]. With regard to clinical significance, TMPRSS3 acts as a pro-oncogene in breast cancer [33, 34], pancreatic cancer [35], glioma [36], gastric cancer [37], and nasopharyngeal cancer [38]. In the case of OV, initial studies revealed that TMPRSS3 expression was upregulated in OV tissues compared to normal tissues [39], while further research indicated that this gene promotes the proliferation, invasion, and metastatic ability of OV cells through the regulation of the ERK pathway [34]. Recent studies have confirmed the results of our analysis by showing that the high expression of TMPRSS3 in OV tissues is caused by a deficiency of methylation in the promoter region [40]. HLA-DOB was originally identified in the class II region of the human major histocompatibility complex, [41] whose polymorphisms are tied with hematologic tumorigenesis [42, 43]. Furthermore, bioinformatics studies focusing on OV have identified HLA-DOB as a prognostic marker in OV patients [44]. Widely expressed in the central nervous system, the neuropeptide encoded by NPY affects many physiological processes, such as the patient's cortical excitability, stress response, food intake, circadian rhythm, and cardiovascular function through *G*protein-coupled receptors. The high expression of NPY matches up with poor prognosis in patients with neuroblastoma [45] and prostate cancer [46]. DNA methylation sequencing analysis for gastric cancer [47] and myelodysplastic syndrome [48] further showed that NPY is regulated by its DNA methylation level. Transcription factors encoded by TCF15 are essential in embryonic development, although their roles in tumors have not been clarified yet. ACSS3 is a metabolic enzyme in charge of catalyzing the synthesis of acetyl coenzyme A from short-chain fatty acids, which corresponds with the progression of gastric [49], hepatocellular [50], bladder [51], and prostate cancers [52]. As far as this research is concerned, ARPC1B, TCIRG1, NPY, TCF15, and ACSS3 are reported for the first time for their prognostic value in OV, of which the deeper mechanisms of action still require further exploration.

The fabricated 8-gene signature accurately predicted the training set (TCGA), validation set (International Cancer Genome Consortium (ICGC) and GSE17260), and prognosis of patients with OV with different subtypes. After incorporating clinical characteristics, the univariate and multivariate cox analysis revealed that the 8-gene signature is an independent prognostic factor for patients with OV. This finding further validates the reliability of the model. In addition, the 8-gene signature performed better than other published prognostic models for OV [12–14]. Therefore, this paper further analyzed the mechanism of this 8-gene signature in OV. Gene Set Variation Analysis (GSVA) found that the Mitogen-Activated Protein Kinase (MAPK), FOCAL_ADHESION, and Transforming Growth Factor Beta (TGF_BETA) pathways all showed increasing RiskScore. MAPK pathway is an essential signal transmitter from the cell surface to the interior of the nucleus. And MAPK activation, the final step in the intracellular phosphorylation cascade reaction, is involved in the regulation of various biological behaviors, such as cell proliferation, differentiation, transformation, and apoptosis [53, 54]. The MAPK pathway is determined in the progression of OV [55]. Focal Adhesion Kinase (FAK) is a class of cytoplasmic nonreceptor protein tyrosine kinases and a hub for intra- and extracellular signaling in and out, thus mediating multiple signaling pathways [56, 57]. In OV, the upregulation of FAK activity promotes the malignant biological behavior of tumor cells [58, 59]. Moreover, VS-6063, a specific inhibitor of FAK, can reverse taxol resistance in OV, thus exhibiting great potential for clinical application [60]. The TGF_BETA pathway determines the specific outcome of cells during cell proliferation, embryogenesis, differentiation, and death [61–63]. The results of this study suggest that methylation-related genes in OV may function through the MAPK, FOCAL_ADHESION, and TGF_BETA pathways.

At the time of the initial diagnosis, OV is often advanced due to its insidious location, a lack of better screening tools, and obvious symptoms. Worse still, this kind of tumor is primary or secondary resistant to chemotherapy, giving OV patients a 5-year survival rate of 30% to 45% [64]. Currently, immunotherapy, especially Immune Checkpoint Blockade (ICB), is very effective in treating melanoma and nonsmall cell lung cancer, while the therapeutic value of immunotherapy in OV is still in the research stage. The approval of monoclonal antibodies to Programmed cell death protein 1 (PD-1) by the U.S. Food and Drug Administration (FDA) is a notable development for the treatment of solid tumors with high microsatellite instability and mismatch repair defects, including OV [65, 66]. However, the objective response rate of PD-1 monoclonal antibodies alone is not promising. Therefore, we must investigate molecular markers that can predict the efficacy of immunotherapy in OV, so as to screen for appropriate immunotherapy populations. An immunotherapy cohort (IMvigor 210) was retrieved to explore whether the 8-gene model could predict the benefit of immunotherapy. The results showed that higher RiskScore values corresponded with poorer survival rates in mUC patients treated with immunotherapy, while the ROC curves indicated that RiskScore predicted patients' response to immunotherapy more efficiently. These results suggest that an 8-gene signature can predict patient response to immunotherapy, demonstrating its potential for clinical application in predicting the efficacy of PD-1 monoclonal antibodies in OV patients.

The fabricated 8-gene signature shows good prognostic efficacy across multiple platforms and patient subgroups. However, the TCGA database is mostly limited to Caucasian and African populations and lacks data from the Asian population. In addition, all subjects in the study are retrospective samples, and we still need the validation of prospective samples.

In conclusion, we analyzed the prognostic value of methylation-related genes in OV by combining methylation omics and transcriptomic data. Besides, the constructed 8-gene signature predicted OV patients' prognosis and immunotherapy response well, and thus, could be applied clinically.

## Data Availability

The data and materials can be obtained from the corresponding author upon request.

## Abbreviations

TCGA: The cancer genome atlas
GSVA: Gene set variation analysis
DMEG: Differentially methylated genes
OV: Ovarian cancer
GEO: Gene expression omnibus
OS: Overall survival
ARPC1B: Actin related protein 2/3 complex subunit 1B
TCF15: Transcription factor 15
TCIRG1: T CELL IMMUNE REGUlator 1
NPY: Neuropeptide Y
ACSS3: Acyl-CoA synthetase short chain family member 3
EMT: Epithelial-mesenchymal transition
ICGC: International cancer genome consortium
ICB: Immune Checkpoint Blockade.

## Supplementary Materials

Supplementary Materials Supplementary Figure 1. (a) Distribution of DMSs in different CpG regions; (b) Proportion of hypermethylation and hypomethylation in different CpG regions; (c) KEGG annotation plots of differential Hypermethylation genes in molecular subtypes; (d) KEGG annotation plots of differential Hypomethylation genes in molecular subtypes. Supplementary Figure 2. (a) RiskScore, OS, survival status, and expression of 8-gene in full TCGA cohort; (b) ROC curve of 8-gene signature classification and AUC; (c) KM survival curve distribution of 8-gene signature in full TCGA cohort. (d) RiskScore, OS, survival status, and expression of 8-gene in the independent validation cohort; (e) ROC curve of 8-gene signature classification and AUC; (f) KM survival curve distribution of 8-gene signature in the independent validation cohort. S1_Table. The 1053 methylation data from DNA methylation sites. S2_Table. The samples were clustered into two major groups. S3_Table. The C2 molecular subtypes were subdivided into 1350 sites in hypermethylation state and 1378 in hypomethylation state. S4_Table. A total of 1877 CpGb matched to the corresponding genes. S5_Table. 742 Hypermethylated genes and 921 Hypomethylated genes. S6_Table. Identification of DEGs between *C*2 and *C*1 molecular subtypes. S7_Table. The results of univariate cox analysis.
