# Supplementary material for: Integration of Transcriptome and Epigenome to Identify and Develop Prognostic Markers for Ovarian Cancer
Source: J Oncol. 2022 Aug 30;2022:3744466. doi: 10.1155/2022/3744466 (PMC9448543; doi:10.1155/2022/3744466)
Supplement: Supplementary Materials — Supplementary Figure 1. (a) Distribution of DMSs in different CpG regions; (b) Proportion of hypermethylation and hypomethylation in different CpG regions; (c) KEGG annotation plots of differential Hypermethylation genes in molecular subtypes; (d) KEGG annotation plots of differential Hypomethylation genes in molecular subtypes. Supplementary Figure 2. (a) RiskScore, OS, survival status, and expression of 8-gene in full TCGA cohort; (b) ROC curve of 8-gene signature classification and AUC; (c) KM survival curve distribution of 8-gene signature in full TCGA cohort. (d) RiskScore, OS, survival status, and expression of 8-gene in the independent validation cohort; (e) ROC curve of 8-gene signature classification and AUC; (f) KM survival curve distribution of 8-gene signature in the independent validation cohort. S1_Table. The 1053 methylation data from DNA methylation sites. S2_Table. The samples were clustered into two major groups. S3_Table. The C2 molecular subtypes were subdivided into 1350 sites in hypermethylation state and 1378 in hypomethylation state. S4_Table. A total of 1877 CpGb matched to the corresponding genes. S5_Table. 742 Hypermethylated genes and 921 Hypomethylated genes. S6_Table. Identification of DEGs between C2 and C1 molecular subtypes. S7_Table. The results of univariate cox analysis. [file 3744466.f1.pdf]

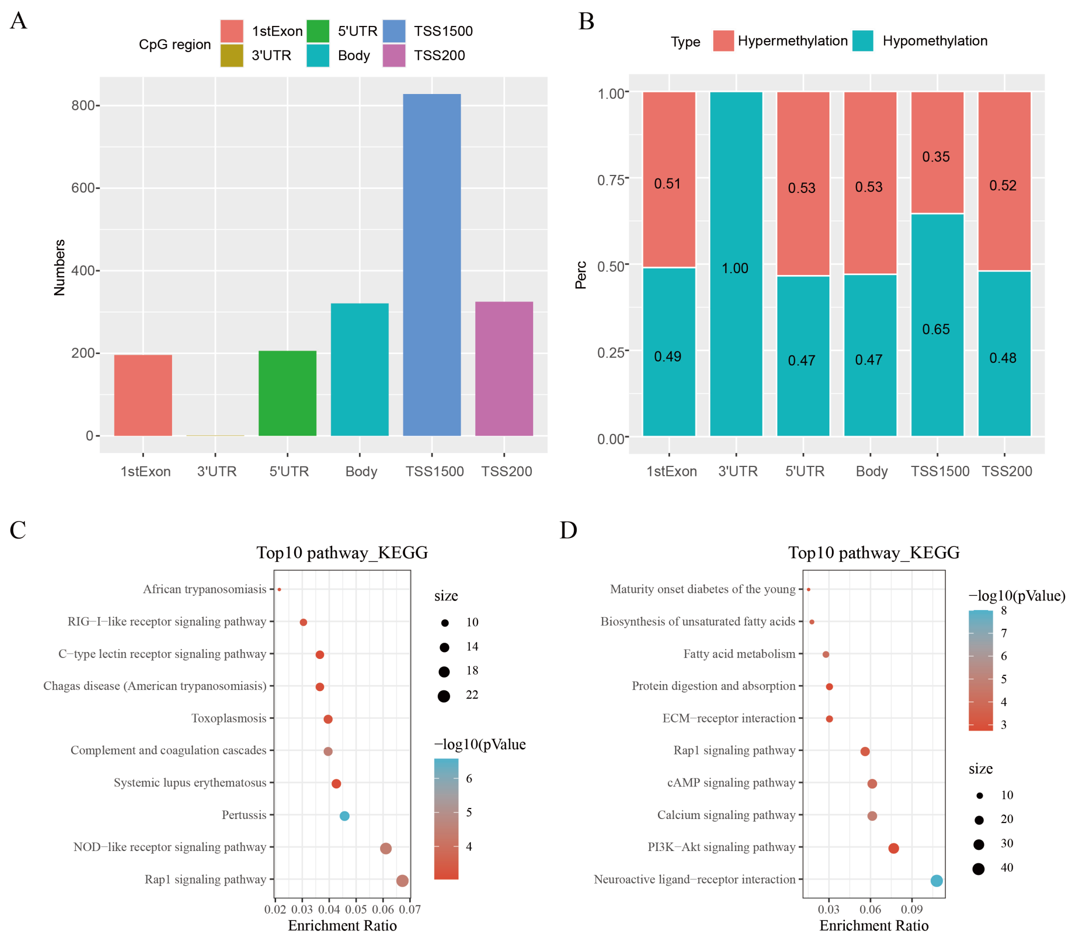

Supplement Fig1. A: Distribution of DMSs in different CpG regions; B: Proportion of hypermethylation and hypomethylation in different CpG regions; C: KEGG annotation plots of differential Hypermethylation genes in molecular subtypes; D. KEGG annotation plots of differential Hypomethylation genes in molecular subtypes.

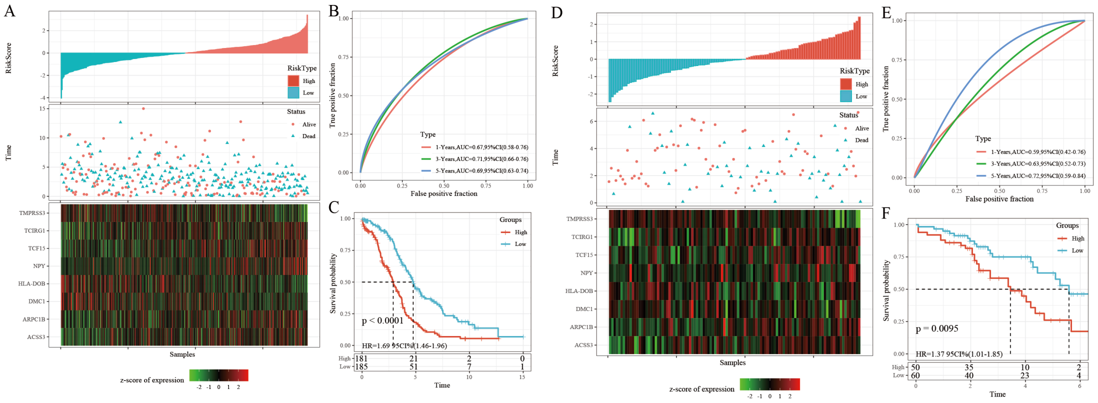

Supplement Fig2. A: RiskScore, OS, survival status, and expression of 8-gene in full TCGA cohort; B: ROC curve of 8-gene signature classification and AUC; C: KM survival curve distribution of 8-gene signature in full TCGA cohort. D: RiskScore, OS, survival status, and expression of 8-gene in the independent validation cohort; E: ROC curve of 8-gene signature classification and AUC; F: KM survival curve distribution of 8-gene signature in the independent validation cohort.

Supplementary table 1. The 1053 methylation data from methylation sites

	p.value	HR	Low 95%CI	High 95%CI
cg09001953	0.012690975	0.340530945	0.145963625	0.794453584
cg06466797	0.025888157	98.42357756	1.736546706	5578.427914
cg07471052	0.045710569	11.70672525	1.048019414	130.7680127
cg25927124	0.046771852	5.98E-08	4.54E-15	0.788576287
cg08123074	0.044473165	0.203108904	0.042909933	0.961391089
cg04786857	0.043401076	0.628145354	0.400041989	0.986312929
cg00278366	0.041493781	0.57838588	0.341667119	0.97911156
cg18755783	0.007843196	11.4291328	1.897017432	68.85813187
cg05316065	0.00704836	3.127241016	1.364553537	7.166912917
cg16114640	0.015010323	0.105189983	0.017132452	0.645846389
cg23668631	0.036153738	0.462695134	0.225005296	0.951474436
cg18382305	0.022051468	0.555077775	0.335352741	0.918767909
cg02668984	0.038700011	1.44E-05	3.72E-10	0.560466049
cg22171829	0.026378022	0.046490773	0.003098472	0.697567082
cg18043455	0.021346298	0.001817915	8.44E-06	0.391761361
cg15749322	0.048399748	233.7593289	1.039092837	52587.6244
cg10213812	0.027565726	0.221418871	0.05791278	0.846554354
cg10769011	0.011477006	0.284669703	0.107463823	0.754084842
cg23751724	0.004624467	21.52146341	2.573064364	180.0084731
cg17884373	0.016850926	0.364823242	0.159570182	0.83409066
cg12790134	0.0152438	36.10375749	1.992892725	654.0649623
cg11389172	0.045609531	3.582039109	1.025222132	12.51534061
cg00066816	0.034431508	4.334080472	1.113477381	16.86990132
cg07296772	0.010815382	0.126813961	0.025910321	0.620670824
cg23873703	0.039842648	0.63507704	0.411913298	0.979145
cg16547450	0.04581836	0.005434084	3.25E-05	0.907666133
cg16384137	0.044447936	0.592106249	0.355179259	0.987078498
cg17820591	0.006241294	131.4855654	3.984240677	4339.209226
cg17384214	0.042356328	0.269672346	0.076086888	0.955791159
cg24335149	0.022838985	0.243774778	0.072297784	0.821963538

cg12071073	0.032962384	0.399144385	0.171608518	0.928370231
cg04613791	0.018887686	544667.6086	8.86006601	33483136982
cg22925006	0.043874585	0.132811322	0.018643991	0.946087542
cg00630583	0.048873318	6.11E-07	4.01E-13	0.9317529
cg08831701	0.016159476	20.0561964	1.742230652	230.8827557
cg08880153	0.033755236	0.157625723	0.028632992	0.867735671
cg12893143	0.015817887	2.14E-06	5.32E-11	0.086184596
cg01464985	0.028144998	0.420734684	0.194230174	0.911380915
cg18611281	0.040996638	9.884189682	1.098251936	88.95700743
cg22573917	0.03285018	4.505191188	1.1305439	17.95308227
cg16747567	0.048887132	0.006558017	4.41E-05	0.97576837
cg16606638	0.047582623	41.46351412	1.040495338	1652.312069
cg05679613	0.021080041	4.74708118	1.263689701	17.83252623
cg03064400	0.031835748	3.49E-05	2.97E-09	0.409955759
cg07850221	0.004644091	0.051776265	0.006665185	0.402206648
cg02674804	0.016984059	0.567644894	0.356577367	0.903648844
cg17675882	0.004826357	29718.82422	23.03609231	38340205.5
cg26413355	0.019775712	2.53E-06	4.98E-11	0.128852113
cg00432979	0.010159084	3.31E-06	2.20E-10	0.0499614
cg04032226	0.037008647	0.719227173	0.527665808	0.980332096
cg05010623	0.001118677	716.4360651	13.74009255	37356.4176
cg24341944	0.000646172	44.12618495	5.009313494	388.7000086
cg00327483	0.029143117	0.000497792	5.37E-07	0.461866734
cg24727568	0.014637931	383.2705049	3.230737559	45468.34189
cg05469695	0.034641322	668.4455682	1.599633981	279326.0728
cg08338368	0.012903595	3.678438942	1.317556343	10.26970355
cg23580000	0.01051111	0.001044802	5.44E-06	0.200720505
cg08460026	0.042649437	1.524516824	1.014069101	2.291906484
cg23111544	0.030118396	0.582239517	0.357104658	0.949309531
cg00579393	0.049894179	0.534081468	0.285325698	0.999710212
cg07841014	0.017458829	0.573033247	0.362057633	0.906947047
cg12099357	0.008640346	3.12E-06	2.43E-10	0.040149225
cg27626899	0.041946055	0.004030636	1.99E-05	0.818041104

cg04815626	0.024447545	0.002380789	1.23E-05	0.45901204
cg14391855	0.03986649	0.31612517	0.105409041	0.948069744
cg05064181	0.041597079	0.35303762	0.129674792	0.961139471
cg16913124	0.024326377	0.015132631	0.000394284	0.580790762
cg11730100	0.024238014	0.003925397	3.17E-05	0.486113217
cg14947494	0.00828572	2.47E-08	5.55E-14	0.010961608
cg12780286	0.034927543	1.23E-09	6.47E-18	0.234207384
cg20098478	0.006583937	0.472466756	0.275098628	0.811435656
cg03338959	0.042844064	0.012747228	0.000187018	0.868854359
cg09607282	0.040220097	0.063798272	0.004601885	0.884467919
cg24459023	0.030820116	4557.68242	2.17716796	9541050.312
cg05973262	0.048725815	0.400072727	0.160880049	0.994891457
cg16829154	0.040731652	4.03E-05	2.49E-09	0.652686993
cg13382694	0.045209977	0.124706252	0.016258015	0.956552796
cg12542281	0.036920903	0.219460927	0.052807875	0.912043871
cg15779381	0.046990717	0.004376507	2.06E-05	0.930306032
cg20044189	0.008638802	136.8177968	3.481842107	5376.208614
cg18488623	0.047931736	57883092918	1.253116573	2.67E+21
cg10057295	0.033034407	0.089311092	0.009689761	0.823185585
cg07911673	0.014095324	0.237222481	0.075209408	0.748237579
cg04230869	0.026817884	1.84E-08	2.61E-15	0.129395641
cg16534233	0.004653388	23113.3066	21.96341323	24323402.57
cg22199779	0.04396062	271.779692	1.163747178	63471.00335
cg20903926	0.039307038	0.605783303	0.376098678	0.975737039
cg02537838	0.013839009	2.994806802	1.250397587	7.172812773
cg13925920	0.019970101	0.012251726	0.000300524	0.499477137
cg18034859	0.026357515	0.031206325	0.001463526	0.66540329
cg26680127	0.025966863	0.238296479	0.067429812	0.842138078
cg19237753	0.003388177	917896.5692	94.25060437	8939296649
cg07260017	0.000753049	0.359374564	0.198161341	0.651742044
cg03159785	0.005943047	56.06678373	3.182653631	987.6928508
cg21303011	0.018283962	3.508047385	1.236964259	9.948869873
cg03156547	0.027245585	0.50735655	0.277797282	0.926613345

cg03770548	0.001314189	0.389317888	0.21896517	0.692203321
cg03801144	0.009147849	87.03986648	3.027823323	2502.10714
cg22468803	0.030490967	2888.444664	2.117345419	3940364.431
cg08424423	0.032042672	0.640964286	0.426813889	0.962562901
cg24735489	0.005631786	0.503565632	0.309831054	0.818440703
cg11939496	0.002993997	0.382014115	0.202363441	0.721151919
cg10978355	0.006153119	0.067354843	0.009775357	0.464093006
cg03821311	0.041988575	0.492623011	0.248982141	0.974678063
cg10056627	0.046855159	0.024164705	0.000614963	0.949540958
cg24101359	0.048528717	0.004096125	1.74E-05	0.965104276
cg23239039	0.007787355	62.66764209	2.975118097	1320.026042
cg04732193	0.028550831	0.378587545	0.158700593	0.903137954
cg01110312	0.016413029	0.496412191	0.280162613	0.879578686
cg18641050	0.002587696	0.205452409	0.073385864	0.575188328
cg16396417	0.021149677	49.04300637	1.791641381	1342.46535
cg06425556	0.037133733	3376.289903	1.623563797	7021179.907
cg17111669	0.036307642	5.38E-07	7.26E-13	0.398290046
cg16551326	0.038137383	47258.42436	1.801724617	1239567163
cg06911113	0.02048948	6.492382713	1.334333186	31.58958628
cg01091565	0.016598747	0.462820378	0.246408177	0.869300301
cg01294695	0.017353872	0.076384134	0.009179126	0.635630878
cg16949889	0.007482273	0.000259799	6.13E-07	0.110162237
cg24169822	0.04576042	0.598483054	0.361662779	0.990375527
cg15275890	0.045997671	0.58396122	0.34428528	0.990488779
cg16179125	0.025516897	2.721396128	1.130437236	6.551444563
cg14377791	0.00164652	0.106661738	0.026469882	0.429798908
cg08013810	0.014101683	0.315723565	0.12575371	0.7926714
cg05103623	0.018444844	0.292352777	0.105123602	0.813044309
cg00626466	0.016385126	0.546888647	0.334097651	0.895208903
cg24556026	0.005442895	0.435187797	0.242045571	0.782449429
cg06038133	0.003917384	3.712772989	1.522758447	9.052442489
cg26388152	0.024955489	101.0903774	1.787544551	5716.928506
cg06353318	0.038211945	0.171403979	0.032334046	0.908618868

cg01367992	0.005944543	0.539312673	0.347357711	0.837344761
cg07854244	0.048811849	1.71E-05	3.08E-10	0.944394731
cg08563994	0.035863538	5.97156E+12	6.970619762	5.12E+24
cg06598256	0.003601232	0.00516455	0.000149038	0.178965308
cg14654385	0.036081021	0.102295236	0.012133321	0.862444466
cg23090046	0.000930735	0.184162972	0.067638089	0.501433449
cg21666675	0.045882045	19.47599634	1.055803736	359.2660458
cg11177693	0.039157278	0.660703803	0.445619701	0.979601022
cg27472032	0.035198138	3.25E-07	2.97E-13	0.35469292
cg12413566	0.029273044	0.181498977	0.03912811	0.84189802
cg22307649	0.017658498	7558782.488	15.72265498	3.63394E+12
cg19483159	0.010816163	0.024751473	0.00143951	0.425585985
cg16243646	0.020016831	0.002841634	2.03E-05	0.397416142
cg13002506	0.03646309	0.646621051	0.429761168	0.972909641
cg03814826	0.028301508	0.000365768	3.10E-07	0.431201025
cg19324172	0.029276326	0.001748622	5.80E-06	0.527210142
cg04457051	0.042342368	0.381498787	0.150470987	0.967238451
cg06611744	0.035314387	27.89577476	1.257271693	618.9388131
cg23207527	0.025952903	1.635254251	1.060726849	2.520966137
cg15448599	0.011569553	0.016406645	0.000675276	0.39861949
cg02809263	0.046578967	650211.6219	1.224762282	3.4519E+11
cg21076470	0.046533242	0.071617812	0.005340977	0.96033205
cg06391660	0.013952783	0.000694925	2.11E-06	0.228891813
cg16094520	0.003667131	77.7728904	4.123958979	1466.702873
cg08724563	0.009424986	10.14163925	1.764303473	58.29657327
cg22375610	0.023586304	0.524481527	0.299969443	0.917029648
cg03693099	0.004210769	2.421806018	1.32149308	4.438270982
cg05657090	0.026369846	0.616378392	0.402134142	0.944765148
cg09475757	0.026567975	0.02920861	0.001286537	0.663131382
cg26323655	0.000536575	2.490216322	1.485593333	4.174209182
cg20456055	0.013099595	22.70658689	1.926760598	267.5937472
cg09657538	0.035494632	0.021021307	0.000574326	0.769415116
cg20972543	0.04062071	4.04E-08	3.38E-15	0.483427859

cg18589858	0.030386032	0.255228474	0.074136195	0.878674357
cg13269407	0.013270826	0.037736016	0.002820511	0.504875572
cg17543123	0.014827548	6.69E-06	4.60E-10	0.097239937
cg08605641	0.026030108	678446.4604	4.970427489	92605636149
cg06494782	0.048800389	0.612956569	0.376684643	0.997427857
cg00917893	0.037382114	6.45E-08	1.10E-14	0.380154242
cg03144357	0.042827674	0.338946389	0.118970536	0.965656356
cg21226234	0.026758629	0.085301751	0.009660837	0.753184062
cg12582959	0.008200159	0.321178243	0.138376976	0.745466957
cg04785227	0.04848566	0.080490463	0.006588199	0.983381741
cg25484904	0.049363203	0.526378435	0.277570203	0.998213258
cg15942562	0.021300779	0.394723882	0.17892107	0.870813832
cg22809047	0.044131375	0.625020627	0.395503662	0.987729879
cg08826839	0.044058718	0.613587854	0.381414159	0.987089877
cg04172043	0.04579703	75.21429126	1.08401815	5218.722222
cg12421458	0.019207019	60.51029176	1.95134048	1876.400067
cg12924262	0.040913639	0.243186134	0.062696056	0.943272977
cg24797830	0.020137846	0.000252116	2.33E-07	0.273300532
cg13756879	0.021788357	3.176770513	1.183321051	8.528430119
cg13791131	0.000179034	2.518207194	1.55340493	4.082237252
cg25574024	5.74E-05	2.230469064	1.508931187	3.297030567
cg02604290	0.041471084	0.022091147	0.000565527	0.862944791
cg23910243	0.012342039	0.473333926	0.26346853	0.850367233
cg22325715	0.005836666	0.544569721	0.353508921	0.838893061
cg01420388	0.031087236	0.654017848	0.444573228	0.962134735
cg15746187	0.035752765	0.622497827	0.399924928	0.968940713
cg06538003	0.040229047	256146.696	1.741979161	37664704230
cg04848452	0.007092384	0.073130845	0.01089513	0.490872574
cg04985146	0.010990616	37.85800131	2.300510848	623.0043491
cg05028467	0.045520778	0.024758873	0.000659882	0.928956127
cg15895197	0.029946713	0.532847017	0.301826805	0.94069161
cg20516209	0.005639958	0.245802694	0.091007091	0.663892933
cg10218959	0.045059338	0.073483129	0.00571933	0.944126418

cg00053292	0.030183537	8.57E-07	2.80E-12	0.262213556
cg01413314	0.025659665	15.53463452	1.395978839	172.8714382
cg16298282	0.025767421	0.235460186	0.066041434	0.839495685
cg18194038	0.012054953	0.000700669	2.41E-06	0.203334398
cg11277126	0.023092977	0.257391578	0.079820338	0.829994291
cg15643724	0.014374678	0.000296831	4.45E-07	0.19816668
cg04968426	0.013332192	0.21289212	0.062528304	0.724840622
cg08558340	0.040769451	0.67118903	0.458099946	0.983398313
cg10575841	0.034159475	3.13E-07	3.00E-13	0.326690338
cg16293656	0.044494846	3.145552061	1.028594651	9.619433421
cg26491425	0.040991265	0.07225578	0.005813753	0.89802534
cg00830029	0.003676838	0.454601302	0.267060449	0.773841072
cg05194726	0.011292517	0.452049142	0.244586327	0.835485896
cg04743650	0.046064536	0.551385337	0.307203166	0.989657084
cg08942875	0.004551229	9986.8975	17.24164828	5784720.815
cg16116363	0.04164903	3.55E-11	3.13E-21	0.402494958
cg10045881	0.012308364	0.561171485	0.356976713	0.882168008
cg04970994	0.018628828	0.036353705	0.00229866	0.574940146
cg19525717	0.043955302	0.136780138	0.019744332	0.947553259
cg10786622	0.046142895	2.15E-08	6.23E-16	0.739250105
cg26272237	0.019357761	24.21955632	1.675293491	350.1397884
cg04432009	0.009297455	0.425390863	0.223385766	0.810066773
cg07952391	0.000547896	3.050922991	1.620746246	5.743114396
cg07080946	0.047566771	0.680998565	0.465674445	0.995886829
cg15811235	0.002607384	258.8248594	6.951595014	9636.681613
cg14031452	0.045627434	0.146072007	0.02215011	0.963292347
cg02198044	0.041808652	0.008003019	7.66E-05	0.836124107
cg18910313	0.022306584	0.444032105	0.221308115	0.890905021
cg17285325	0.030605489	0.370772716	0.150838284	0.911389356
cg05318777	0.021348675	0.258221307	0.081526639	0.81787062
cg05562817	0.000221821	4441.842161	51.47486244	383293.1424
cg25903497	0.016173816	5.486685391	1.370246943	21.96955573
cg25040783	0.006313173	6.33E-05	6.15E-08	0.065259508

cg08923379	0.039885747	1.17E-13	5.43E-26	0.252569978
cg15075170	0.024105811	4.46E-07	1.35E-12	0.147259652
cg04313552	0.037824337	0.077431985	0.006923473	0.865997819
cg07246225	0.048762603	0.222234847	0.049793205	0.991868804
cg00480356	0.005390928	0.177810697	0.052683335	0.600126086
cg04979725	0.049914382	5.25E-06	2.77E-11	0.995465716
cg02533993	0.044502471	1.59E-05	3.30E-10	0.762222253
cg02788090	0.02494619	7.31E-05	1.77E-08	0.30150107
cg11554937	0.003929593	0.013080932	0.000686437	0.249273662
cg10458876	0.04899378	641.7851556	1.028917848	400312.0238
cg24167928	0.008176558	7.166729116	1.665129098	30.84566012
cg05227963	0.0317766	31189098.09	4.503064593	2.16022E+14
cg19317638	0.023058411	0.198020636	0.048993089	0.800361304
cg25203561	0.001962172	0.051749182	0.007937732	0.337373183
cg16928795	0.006197642	114.886301	3.845788921	3432.029793
cg15911500	0.033975229	3.56E-18	2.65E-34	0.047867468
cg02125271	0.036065728	0.589131466	0.359217354	0.966200214
cg18506672	0.023149118	0.569730686	0.350589911	0.925848246
cg02082571	0.042520679	0.213758049	0.048133646	0.949284062
cg22493172	0.013538768	41.19533254	2.153162055	788.1689254
cg02750754	0.011046178	9078.620547	8.041549031	10249437.11
cg12920798	0.009507784	4.31E-07	6.66E-12	0.027893497
cg07413497	0.037227223	7.77E-08	1.59E-14	0.379509675
cg12080675	0.0314434	0.028478544	0.001113182	0.728567013
cg24060938	0.049402885	3.64738823	1.00338652	13.25854058
cg13424229	0.025502744	1.585740728	1.058127965	2.376436253
cg26391080	0.025129061	0.504967711	0.277687271	0.918271796
cg00168082	0.049894489	0.014539893	0.000211821	0.998052567
cg20897667	0.029668282	0.510852271	0.278849023	0.935882935
cg06792598	0.002136381	21.54374044	3.035699358	152.8915408
cg24206694	0.00137474	988.8429832	14.47404089	67556.14776
cg00540544	0.037572245	15.03694667	1.168589363	193.4894945
cg12026956	0.00447747	9.44E-07	6.61E-11	0.013473721

cg05449607	0.004719178	0.000296705	1.06E-06	0.083038145
cg20188282	0.048497859	80986.57474	1.077410179	6087584296
cg21049762	0.00400651	0.050901775	0.006697148	0.386879728
cg23749163	0.000786682	20.98048728	3.54962468	124.0077153
cg00415993	0.013251601	0.392303508	0.187094547	0.822589675
cg08241785	0.003115764	0.112388765	0.026383081	0.478762673
cg11876012	0.045468976	0.161535213	0.027070467	0.963914834
cg17445987	0.045996489	5.77E-05	3.95E-09	0.840744511
cg16717225	0.041301226	0.461297721	0.219393516	0.96992651
cg00669623	0.032013757	276525.2886	2.935350699	26050119070
cg09155001	0.006442392	0.219333328	0.073634781	0.653320458
cg02506908	0.044008107	0.52983658	0.285553189	0.983098114
cg07031532	0.027218242	334.2033174	1.922711604	58090.80109
cg04703844	0.045958477	980.2355916	1.131550399	849155.1202
cg26358246	0.033785167	399627729.5	4.56212879	3.50061E+16
cg18888520	0.013552341	18.09553637	1.81652047	180.2613523
cg13665593	0.003694824	162.1590781	5.22337084	5034.213999
cg23109897	0.032768916	0.310589897	0.106166152	0.908633146
cg06840042	0.013771697	57.1369989	2.285181712	1428.611399
cg03365354	0.043951468	4.52E-12	4.14E-23	0.492687138
cg14149007	0.03062005	2.28E-05	1.41E-09	0.368337378
cg14386312	0.001744819	2.832081967	1.475885142	5.434493538
cg06583518	0.005946861	0.005982359	0.00015589	0.229575464
cg07281688	0.045696872	5206851.187	1.344205598	2.0169E+13
cg09553448	0.023942138	0.411063602	0.190014622	0.889264644
cg23799276	0.000804583	56386.77937	93.85158124	33877627.26
cg02581667	0.037441208	0.002799796	1.10E-05	0.710576496
cg02675896	0.030050313	9.27E-05	2.11E-08	0.407954079
cg27541374	0.048952303	0.01107371	0.00012519	0.979531034
cg15819853	0.013419294	5.67E-05	2.44E-08	0.131752107
cg16852892	0.040488414	6.18E-07	7.10E-13	0.538240843
cg02178898	0.036228543	0.103507959	0.012392359	0.864556717
cg22691736	0.029039377	0.044020495	0.002665999	0.726858587

cg27625732	0.002673786	0.451630572	0.268823538	0.758751166
cg24124977	0.031998541	73228.0444	2.620969903	2045939742
cg24323726	0.049145766	2.1241553	1.00282279	4.49933506
cg02170525	0.042781699	4.603685353	1.050899519	20.1674075
cg01289103	0.018392554	0.441369438	0.223622264	0.871143047
cg17767184	0.036370461	0.014055835	0.000258966	0.762906236
cg13739417	0.047355447	1.767462603	1.006668855	3.103229068
cg01738359	0.038811821	8.85E-09	2.03E-16	0.385508391
cg17055959	0.018074941	0.048118364	0.003889263	0.595325409
cg02037013	0.048991612	0.594665272	0.354440038	0.997705528
cg06307169	0.045642966	0.051998919	0.002863261	0.944338425
cg01299496	0.04483382	0.015253195	0.000256211	0.908079008
cg13920529	0.004465462	0.004178507	9.57E-05	0.18236394
cg19862344	0.01721805	0.610980537	0.407349469	0.91640531
cg00864867	0.020518185	24119.10937	4.730113444	122984669.1
cg11795262	0.033584667	0.067106574	0.005554694	0.81071834
cg05373457	0.026351292	1.620193583	1.058352085	2.480296758
cg06291334	0.025796519	0.124970661	0.020078108	0.777845524
cg18942631	0.020969275	0.526196626	0.305070942	0.907601646
cg09034896	0.024556882	0.454718878	0.228759705	0.903870976
cg15571154	0.029940437	0.316426591	0.111968886	0.894228668
cg10940099	0.017281997	3.88E-09	4.61E-16	0.032627569
cg18414381	0.045721542	1.871630946	1.011990068	3.461498792
cg08835688	0.010935534	0.474213171	0.266937143	0.84243852
cg02144298	0.013366915	15686.45437	7.438968847	33077817.05
cg02661879	0.026598845	12.30796421	1.338286269	113.1940053
cg25664034	0.010652523	0.050724584	0.005146952	0.499904272
cg11868900	0.022706349	4.401044369	1.230072234	15.74638546
cg06426831	0.015265033	0.129085775	0.024692568	0.674823982
cg05294095	0.026258211	2.77E-18	9.06E-34	0.008449439
cg07883333	0.022492878	4.76E-05	9.22E-09	0.245522173
cg05689121	0.04757142	1.59E-05	2.84E-10	0.888405445
cg10447080	0.04681854	0.646604545	0.420670619	0.993883145

cg07422345	0.044530664	1.83E-06	4.62E-12	0.724074674
cg01033160	0.002653801	254.3516435	6.86511412	9423.697471
cg15046693	0.018728948	0.605024635	0.397955186	0.919839271
cg12568776	0.025701143	2.87E-09	8.94E-17	0.091875536
cg25004981	0.02933222	0.0071695	8.44E-05	0.608747832
cg23828212	0.018694325	66.93818082	2.013860352	2224.940794
cg26608667	0.029905153	0.25198254	0.072613519	0.874426716
cg14672994	0.010857668	0.377884297	0.178714619	0.799019926
cg26091981	0.030566653	3.650053727	1.129025586	11.80034569
cg08697665	0.049254446	0.491407195	0.242041698	0.997683595
cg13652336	0.036003569	4.792337361	1.107741886	20.73271551
cg16873684	0.00864837	83198.11248	17.67688134	391580719.8
cg00158308	0.003710704	65.32252557	3.882380984	1099.076151
cg27040030	0.04839087	2.91010441	1.007580761	8.404991443
cg12091331	0.02888396	0.374427397	0.155126623	0.903751224
cg05869585	0.030726957	0.270332898	0.082518286	0.885620375
cg21245372	0.018685208	0.502391851	0.283067336	0.891652055
cg21743649	0.016845764	0.15601259	0.034002507	0.715827451
cg22396353	0.027687332	6.61E-15	1.57E-27	0.027744378
cg23977670	0.017990379	0.39907466	0.186443329	0.854203713
cg27508002	0.034098142	0.290651801	0.092682961	0.911477883
cg22381955	0.009884148	0.125613985	0.025975305	0.607456717
cg21618713	0.015080773	34819538.08	28.83389157	4.20477E+13
cg23841186	0.003638632	0.526134418	0.341281072	0.811112743
cg17718515	0.001339833	478.9910343	11.02548719	20809.27645
cg04032566	0.022908418	0.138577084	0.025247924	0.760601484
cg02741744	0.044222779	0.002491064	7.25E-06	0.856440365
cg02533173	0.001871664	0.214237174	0.081133237	0.565706095
cg08044694	0.013512597	0.509394228	0.298260618	0.869985723
cg00209066	9.85E-05	0.0001172	1.23E-06	0.011153307
cg19692710	0.044242596	1.612467714	1.012382697	2.568250263
cg09892390	0.009467938	110.4436592	3.160796812	3859.09079
cg00672638	0.02423027	52473.02953	4.118438352	668558951.9

cg20346096	0.033596573	0.18098091	0.037400921	0.875756249
cg23486067	0.018564387	9.182780258	1.449600414	58.17013605
cg08278554	0.008123101	0.258004162	0.09461336	0.703559709
cg20543571	0.040066484	0.155730656	0.02638585	0.91913041
cg13179915	0.004251676	0.223366175	0.079931807	0.624187665
cg24743283	0.025705698	0.000470215	5.60E-07	0.394652991
cg20865068	0.030150013	2.04E-06	1.46E-11	0.28421871
cg06461769	0.047952368	2.06E-07	4.87E-14	0.870429478
cg21643045	0.048959581	3.127127345	1.005213111	9.728211186
cg15783800	0.00124282	0.256871389	0.112565543	0.586173255
cg21639968	0.014999691	84.93591107	2.369581429	3044.465533
cg10661591	0.025759122	0.018807767	0.000572163	0.618237033
cg00601486	0.036459626	0.372227802	0.147455355	0.939630416
cg19491035	0.032693676	0.035501702	0.001659086	0.759677726
cg08368934	0.00607453	0.329308952	0.14893857	0.728114857
cg12125117	0.005105084	0.403109285	0.213430315	0.761359019
cg04513422	0.006465795	0.36499248	0.176703155	0.75391699
cg00575744	0.030565831	14.34529896	1.283578653	160.3233287
cg21559783	0.020804918	1.80E-05	1.71E-09	0.189847392
cg19211800	0.011484953	14.1117625	1.812018226	109.9005728
cg20202438	0.015601368	0.469236567	0.254122581	0.866443881
cg01776246	0.042767158	0.048785409	0.002626151	0.906275539
cg19685976	0.047037776	0.000723451	5.75E-07	0.90968312
cg25226247	0.023422554	0.56466152	0.344463657	0.925620528
cg19298821	0.027460847	0.385476984	0.165191531	0.89951648
cg10864501	0.030566491	0.083859847	0.008871629	0.792692554
cg12100791	0.014668894	0.594331186	0.391333837	0.902629738
cg15468095	0.041206655	0.487262648	0.244336653	0.971712126
cg07864632	0.003681039	282.6350427	6.266206486	12748.15433
cg03929796	0.013993078	0.537488933	0.327583671	0.881894854
cg04371779	0.00959605	99503951.69	88.23911784	1.12207E+14
cg12228229	0.002382498	0.16333122	0.050741229	0.525747757
cg10564498	0.045649621	0.217530898	0.048736545	0.970928318

cg01275830	0.016839305	0.141443296	0.028448492	0.703243109
cg02040734	0.020280728	0.063823143	0.006250195	0.651722608
cg19724470	0.029289601	0.561448805	0.334101653	0.943499554
cg20001829	0.039740251	0.346525569	0.126201115	0.951496905
cg15757271	0.035290305	2.67E-06	1.72E-11	0.412963947
cg07156669	0.042366848	0.574589012	0.336515692	0.981091049
cg23733753	0.030622137	1.86E-05	9.55E-10	0.361404532
cg14580567	0.013009866	11.49048701	1.673112392	78.91358181
cg00034039	0.007653398	137078.4521	23.0050809	816797911.9
cg02280309	0.026350532	0.261441517	0.08002525	0.85412625
cg11631518	0.021047274	5.088737671	1.277405383	20.27175666
cg17838026	0.009596375	6.64623939	1.585287333	27.86403267
cg01683883	0.038053771	0.731561454	0.544480865	0.98292189
cg13813391	0.010974728	0.530442519	0.325425076	0.864620729
cg03560090	0.048110481	2.43103E+13	1.291990678	4.57E+26
cg19339848	0.012787253	0.131214994	0.026522702	0.649156128
cg05671018	0.043068424	0.417585083	0.179190812	0.973137519
cg09496393	0.035375253	0.000176311	5.62E-08	0.553307083
cg22916109	0.008574093	928754267.2	191.0396049	4.51521E+15
cg03429034	0.046135631	7.04E-07	6.32E-13	0.78438758
cg20122491	0.015887948	9.84E-08	1.99E-13	0.048760556
cg22534509	0.020028949	10.95265808	1.457178806	82.32395263
cg20684973	0.001373041	129.3350073	6.582918804	2541.052778
cg09599653	0.040814304	4.510492696	1.064952791	19.10370537
cg26477793	0.043193377	12411.95213	1.334374557	115452258
cg09099177	0.007662798	0.042522534	0.004174831	0.43311111
cg19342782	0.015748964	0.41880616	0.206636129	0.848828327
cg06059810	0.016400293	0.097778167	0.014641353	0.652984057
cg10938486	0.038742585	0.405603643	0.172377817	0.954382172
cg09983885	0.028638475	0.042285281	0.002487706	0.718752486
cg20030243	0.021883277	2.66E-11	2.42E-20	0.029279224
cg24991452	0.01480644	86.16216078	2.392673249	3102.771327
cg21974239	0.01485586	3.639713769	1.287119181	10.29237736

cg05859264	0.025248578	2.02333064	1.091373538	3.751114295
cg24115571	0.015910776	0.013679008	0.000417616	0.448055268
cg00893242	0.019840056	0.1184536	0.019679558	0.712986311
cg07015079	0.039051347	17.8564218	1.155826149	275.8648432
cg23036025	0.027584077	0.120689151	0.018395154	0.791831986
cg21279955	0.049323924	0.048417786	0.002365381	0.991080221
cg18783796	0.023054683	6.36E-06	2.10E-10	0.192861399
cg05348272	0.047223481	89.75072123	1.05667938	7623.118342
cg27248887	0.04170441	2.608440422	1.036669144	6.563291167
cg06688396	0.046814161	4.015193329	1.019781563	15.80904975
cg18641937	0.047095928	0.391119572	0.154828047	0.988028475
cg22936016	0.022673842	0.165784871	0.035343676	0.777639072
cg04660234	0.039803711	3824.671658	1.468852566	9958871.046
cg25788012	0.013453008	93.57481239	2.558632082	3422.237052
cg21252483	0.01875191	0.398279371	0.184839196	0.858186255
cg09783309	0.048079065	488.7829353	1.053721576	226728.5431
cg17384145	0.031873113	29.98548327	1.342863467	669.5611495
cg04925864	0.031015361	0.126056946	0.01919761	0.827725622
cg07499372	0.024941992	0.361142696	0.148269528	0.879641614
cg16547341	0.002889442	0.516646889	0.334589954	0.797764563
cg23657252	0.019545455	9.32E-05	3.85E-08	0.22525315
cg26112353	0.031800611	0.074581096	0.006973076	0.797688119
cg21373526	0.04360201	0.003885082	1.77E-05	0.852693344
cg14211646	0.005773963	13982.8962	15.92387354	12278506.58
cg14093936	0.042059221	0.577466108	0.340104769	0.980483475
cg07387199	0.002665536	358832.8067	85.2039337	1511209373
cg10049535	0.026088528	0.171477397	0.036274385	0.810613261
cg12467090	0.032513049	0.114733082	0.015765128	0.834987228
cg20791593	0.030683345	0.590066639	0.365704189	0.952077248
cg09305224	0.045664098	0.3411237	0.118802881	0.979482795
cg18760752	0.032463249	1823.88696	1.872718542	1776328.673
cg19324791	0.030656852	4.61E-05	5.40E-09	0.394213333
cg14047667	0.012705918	6.422674707	1.487379499	27.7338436

cg06178072	0.049494601	0.032534118	0.001066514	0.992456516
cg06335889	0.036910964	1.23E-07	3.98E-14	0.380327098
cg21523528	0.029199563	1.45E-12	3.33E-23	0.063290955
cg16003913	0.032942118	0.547457506	0.3147044	0.952353132
cg10331779	0.042406408	0.480421518	0.236676325	0.975191901
cg23260026	0.029856211	1.656443732	1.050499643	2.61190554
cg03954587	0.036665147	1.498361629	1.025380439	2.189516675
cg02992632	0.011199281	2.560182828	1.238192524	5.293632442
cg10222534	0.022068235	0.173708604	0.038812373	0.777449997
cg04741821	0.024323803	0.002295221	1.16E-05	0.454758006
cg21517055	0.018831309	0.574971077	0.362315055	0.912442736
cg03060925	0.01087775	10682.76798	8.473282505	13468396.88
cg13436343	0.044003991	9.18E-11	1.57E-20	0.537590047
cg24887211	0.042474516	0.43832798	0.197587233	0.972387814
cg04341806	0.018748567	3.34636114	1.222240143	9.161974382
cg14885742	0.003683437	0.243104219	0.093597143	0.631425911
cg11792664	0.002009037	3146.214231	18.98331982	521440.0896
cg15914863	0.032456845	0.621603472	0.402056261	0.961036835
cg22025233	0.034005107	0.483562807	0.247017401	0.946625572
cg04533291	0.014124474	0.608099573	0.408738074	0.904699401
cg12400881	0.048588865	12.2992596	1.015668164	148.9381986
cg24983959	0.011214158	3.01E-09	7.80E-16	0.011600622
cg16256610	0.004737722	2084812984	711.3287064	6.11032E+15
cg11879577	0.046673125	0.390247786	0.154418076	0.986240329
cg24888049	0.049181729	0.461490374	0.213564361	0.997232705
cg03568064	0.023251158	0.001087786	3.00E-06	0.394512695
cg13271963	0.010644041	0.13304187	0.028299197	0.625464359
cg12785689	0.00797549	0.061974928	0.007942802	0.483568861
cg25027167	0.009276572	0.159958104	0.040211491	0.636300585
cg02949544	0.047871455	0.052849549	0.002871118	0.97281794
cg13278334	0.043072261	20.00431929	1.097862491	364.5017417
cg25658980	0.003563211	48.1204424	3.556012489	651.1723408
cg03940966	0.000394798	18796.39767	81.29117255	4346161.512

cg17274742	0.019040617	0.5999092	0.391369696	0.919567999
cg13782957	0.005671715	266369614.3	285.5272449	2.48497E+14
cg05000446	0.046290754	0.558542662	0.314972111	0.990468344
cg21120249	0.041502169	0.571799569	0.334070114	0.978700979
cg18700516	0.014323566	0.070331577	0.008404809	0.588535763
cg00223950	0.023734281	5643580255	19.94611246	1.5968E+18
cg10850580	0.024743228	0.003794091	2.92E-05	0.492341613
cg21548788	0.040795447	0.000325725	1.48E-07	0.714514034
cg06095560	0.004676344	0.011788701	0.000543412	0.25574217
cg09441152	0.011883979	1.55E-09	2.13E-16	0.011337
cg00646492	0.04834418	0.206382141	0.043085497	0.988582968
cg13904493	0.030365738	5.58E-08	1.52E-14	0.205212442
cg14992253	0.025186166	1.08E-05	4.88E-10	0.241081497
cg20807701	0.007327692	3.446735266	1.395134306	8.515297732
cg05140736	0.036484873	0.531789599	0.29425639	0.961067245
cg26780404	0.02422586	20011.50834	3.633476016	110214148.8
cg26159933	0.027409656	0.398893901	0.176267573	0.902697763
cg05131524	0.013350512	0.024491731	0.001296896	0.462523496
cg02233558	0.00072245	53.93418641	5.344141832	544.3149816
cg26755793	0.025088237	2.848789074	1.139848662	7.119891842
cg14378057	0.012284945	0.219539298	0.066997957	0.719387657
cg15679095	0.011655945	0.285857296	0.108034712	0.756371655
cg06850526	0.030997002	2.55891474	1.089667226	6.009215006
cg05959508	0.041414891	0.355264328	0.13140122	0.960514239
cg20018806	0.047883026	0.686232319	0.472570514	0.996496357
cg03712038	0.032767662	5.38966E+13	13.34408067	2.18E+26
cg08902132	0.033316944	1.95E-07	1.29E-13	0.294734201
cg06200339	0.025333177	0.574585949	0.353544925	0.933824782
cg14047008	0.024582131	0.239699168	0.068991232	0.832796995
cg17497271	0.048496754	6.930406292	1.012862836	47.42056838
cg02902770	0.046969268	0.625555734	0.393786975	0.993735196
cg04215480	0.016157513	6412.543525	5.068883886	8112380.436
cg22658985	0.010643151	2210.499506	5.99928452	814481.8021

cg08810582	0.003332401	0.022844048	0.001831829	0.284879556
cg23862908	0.047150005	1.08E-06	1.38E-12	0.841163496
cg11415932	0.01076973	2.92411E+12	766.966652	1.11E+22
cg26701826	0.048854132	0.405928478	0.165526135	0.995479838
cg02321871	0.026571503	0.004004409	3.05E-05	0.526404281
cg15534084	0.017779476	0.292570589	0.105886994	0.808385869
cg05575043	0.019901025	7.31E-06	3.47E-10	0.154054553
cg05874450	0.049459804	3.385060984	1.002885596	11.425668
cg21589280	0.036057615	418.3748099	1.480712344	118211.6718
cg12419067	0.01097158	554355.9321	20.79375001	14778984036
cg18412984	0.033134159	0.406420642	0.177518366	0.93048253
cg20884362	0.03687517	0.625205729	0.402230667	0.971786182
cg00769470	0.026166476	0.57140914	0.348918573	0.9357725
cg26025891	0.049429659	0.507847658	0.258345659	0.998310727
cg03812679	0.031647429	0.017694885	0.000446376	0.701446842
cg09872934	0.026702221	1.32E-05	6.37E-10	0.273205339
cg17188169	0.035754912	0.528411694	0.291327588	0.958436239
cg06236061	0.048851542	0.022270121	0.000505553	0.981021429
cg06351503	0.036303098	0.350080699	0.131043455	0.935235536
cg15727249	0.008638797	0.280198399	0.108411559	0.724195315
cg21022435	0.004233508	0.029231717	0.002597652	0.328948332
cg20999934	0.002733701	70.33648684	4.353576949	1136.357859
cg22967284	0.030089636	0.003740118	2.40E-05	0.583688214
cg22605643	0.045394057	0.64381678	0.418258707	0.99101355
cg19764399	0.041533775	3.25E-06	1.72E-11	0.615821641
cg01116966	0.047629154	5.85E-09	4.18E-17	0.820290592
cg21092324	0.026969418	2.101047157	1.08826158	4.056376922
cg24612198	0.011455927	6.195891725	1.506951674	25.47465517
cg16326979	0.03958542	4.03E-11	5.09E-21	0.31960811
cg11754676	0.004566335	1234.87098	9.017740422	169100.7132
cg18896687	0.019869554	254.487792	2.404429392	26935.30385
cg15800421	0.044024986	0.000972826	1.14E-06	0.830620373
cg10885645	0.049520608	40.59959715	1.007795357	1635.57738

cg03907174	0.031471084	0.515735869	0.282099724	0.942870426
cg09357097	0.038388877	291.9364915	1.35460632	62916.37194
cg07705908	0.027660042	1.880014891	1.071872424	3.297459577
cg06270401	0.042182686	0.435048569	0.194912827	0.971035416
cg22784047	0.032137334	0.177880524	0.036661277	0.86307633
cg14127336	0.049784023	0.625165582	0.391005266	0.999556883
cg10370591	0.029274891	0.668523843	0.465445128	0.960207988
cg16016036	0.019438602	0.527113132	0.308088134	0.901846656
cg07684353	0.017237807	1184.639014	3.500580315	400896.2706
cg24723331	0.048883589	2.684019936	1.004844808	7.169229474
cg00294382	0.049951803	0.561794165	0.31565098	0.999878677
cg15822411	0.049009586	0.512892218	0.263821902	0.997106098
cg15492104	0.014286006	24.20448012	1.891451081	309.7393655
cg03811411	0.005872974	0.47456373	0.279245554	0.806497116
cg25620220	0.001042493	0.412355434	0.242824915	0.700245295
cg14812492	0.014048345	0.01799957	0.000729326	0.44422486
cg17067942	0.023756221	0.050392419	0.003780294	0.671745544
cg22197830	0.000860005	0.483054257	0.31488713	0.741031923
cg06606198	0.015440475	106.9020035	2.437700636	4688.040109
cg16509658	0.033384544	0.002296277	8.51E-06	0.619824509
cg08946332	0.000269709	0.368944314	0.215758599	0.630889836
cg22150335	0.040132624	2.223052455	1.036627916	4.76734433
cg20576597	0.049806298	3.660629818	1.001098596	13.3855054
cg19192120	0.04516544	0.001126205	1.47E-06	0.863958151
cg25149927	0.045623092	0.024444933	0.000642311	0.930320517
cg12845249	0.007820185	84.39917084	3.21230271	2217.480942
cg07115304	0.03368377	0.002340514	8.74E-06	0.626640928
cg26829131	0.007055185	8.539208144	1.794078202	40.64375546
cg14218343	0.011023332	115.2022934	2.964708852	4476.516609
cg05633152	0.026592375	0.056619679	0.00447443	0.716468523
cg08797194	0.031495436	0.000700584	9.35E-07	0.525003999
cg25169784	0.036425896	0.000512779	4.24E-07	0.619641181
cg17777592	0.046651931	1.722628249	1.00809217	2.943627748

cg07786760	0.018016956	0.000302384	3.67E-07	0.249413067
cg16465769	0.007941054	0.189605507	0.055547131	0.647202609
cg08504049	0.03481634	0.011368959	0.000177873	0.726658344
cg14156381	0.028357728	3.354650155	1.136861393	9.898900366
cg26394380	0.002938764	0.202538841	0.070710755	0.580137803
cg24478387	0.049607888	0.001349055	1.84E-06	0.988735629
cg01309153	0.037284111	0.408501509	0.175909582	0.948632138
cg15605888	0.046989781	0.000419528	1.95E-07	0.901710082
cg18042079	0.04130024	0.023558041	0.000643473	0.862477838
cg20701850	0.040262706	2.27E-05	8.28E-10	0.621976673
cg04171565	0.00651794	0.001446317	1.30E-05	0.160736959
cg26347745	0.033087586	29369289.62	3.975949369	2.16943E+14
cg03391568	0.011406701	0.41759388	0.212302346	0.821397651
cg13156411	0.022631872	0.005493954	6.26E-05	0.482061605
cg11653466	0.018945559	0.445993022	0.227213649	0.875430577
cg05836145	0.011765737	0.449996473	0.241763521	0.837582217
cg27210136	0.049109928	1.548073208	1.001705417	2.392450533
cg04467034	0.044680221	4.49E-07	2.86E-13	0.706681999
cg11094938	0.005916041	0.063029496	0.008804555	0.451211601
cg00216361	0.026487012	4.415753316	1.189306676	16.39516345
cg22799850	0.047339058	0.001865201	3.75E-06	0.928857597
cg21835622	0.018677806	39.26271843	1.843449337	836.2372795
cg03000846	0.016783216	0.535272393	0.320715551	0.893366514
cg27485235	0.028512692	0.000387656	3.43E-07	0.437956495
cg26756862	0.008300295	0.281325515	0.109707115	0.721412149
cg23226134	0.027221771	2.63E-06	2.93E-11	0.235701462
cg01297972	0.018446463	10.12062898	1.476253837	69.38314289
cg17403875	0.000206322	3590.418434	47.59597395	270844.432
cg00292662	0.007232795	56.72266424	2.978615009	1080.186808
cg16731016	0.02612957	0.323366734	0.119578958	0.874451879
cg09989134	0.011058556	85.6440588	2.765965673	2651.842312
cg05254747	0.01228163	0.450165342	0.241017816	0.84080438
cg07242414	0.019141563	25.06887972	1.692829996	371.2414902

cg06980053	0.034771376	0.524568464	0.288172844	0.954885514
cg17558126	0.040598179	1.956236787	1.029139122	3.718508301
cg02323334	0.002938906	310975.0682	74.62901488	1295816288
cg10872212	0.022453125	6.47E-05	1.64E-08	0.255761315
cg08871016	0.006619932	16.7335573	2.189786476	127.8718007
cg15298323	0.027937877	0.528336334	0.299120746	0.933199335
cg24092253	0.047815384	0.433721693	0.189632583	0.991994646
cg17398613	0.005785997	0.093567776	0.017394127	0.50332671
cg07748017	0.020370076	21.56266947	1.609579783	288.8634163
cg25513133	0.030268829	1.01E-05	3.05E-10	0.333896564
cg13180098	0.047291202	0.479927328	0.232360952	0.991260529
cg17255302	0.006988708	0.298284593	0.123847872	0.718411199
cg10092957	0.004374165	3.041150051	1.415241296	6.534994182
cg20641580	0.025217714	587174542.2	12.29011875	2.80529E+16
cg20278498	0.002816738	27.99759572	3.144781407	249.2590945
cg16129988	0.005359689	2609045.173	79.46153578	85665556920
cg23101680	0.003784152	0.384863299	0.201656259	0.734516051
cg07123548	0.010951577	0.394656146	0.19282561	0.807742675
cg21466821	0.031615768	0.345253534	0.130903077	0.910597405
cg23146358	0.012767605	500.2464274	3.755980517	66626.14117
cg07426848	0.000242964	0.296313912	0.154741814	0.567409238
cg01870826	0.035204663	0.366115146	0.143712548	0.932697262
cg04336379	0.046880552	0.37505729	0.14258377	0.986563694
cg00129774	0.015511142	9.07E-06	7.49E-10	0.109924716
cg11473104	0.004322216	0.188053924	0.059681166	0.592553405
cg10957680	0.048243557	0.000108641	1.27E-08	0.931943706
cg22407458	0.045023995	0.645271052	0.42044098	0.990328608
cg12154803	0.031630271	0.003393846	1.90E-05	0.606364299
cg23033845	0.038154896	0.560283798	0.324008426	0.968857316
cg26091679	0.001913143	22.69705215	3.159369215	163.056655
cg10107186	0.02463279	0.114700813	0.017350304	0.758273555
cg05714479	0.036427133	61.17734252	1.296767147	2886.15211
cg18829411	0.035723836	0.366599425	0.143699724	0.935249801

cg12287813	0.0129756	56357.21705	10.06800393	315468283.1
cg27215108	0.041583814	21.19180552	1.123485938	399.7314127
cg02144933	0.000631377	22.66385974	3.785288944	135.6965204
cg12627583	0.00171425	26.35291273	3.409977524	203.6599961
cg15341340	0.043064908	2070.309137	1.268976248	3377667.57
cg03196745	0.02092895	3.30E-09	2.09E-16	0.052122027
cg08590939	0.006331404	19.1391983	2.299432936	159.3040204
cg25759381	0.049875199	0.225868944	0.051058165	0.999189451
cg23118151	0.004096734	25.84455813	2.805749679	238.061574
cg05443740	0.000761642	21.27392058	3.587153025	126.1668219
cg25123470	0.001611998	3.008010547	1.517191704	5.963733804
cg15140807	0.033766596	5.769497869	1.143934992	29.09877387
cg24499411	0.032331589	0.402334498	0.174778802	0.926159506
cg09618028	0.019172529	3.73E-05	7.35E-09	0.189422922
cg17691309	0.01873635	23056128.67	16.74724031	3.17417E+13
cg27022827	0.003836116	0.416003786	0.229558101	0.753879516
cg17240836	0.048724046	9.03E-09	9.04E-17	0.901496404
cg16545079	0.044737712	1.841034914	1.014440235	3.341162387
cg06491924	0.045157042	0.001229425	1.75E-06	0.865369878
cg03293882	0.018796004	0.124001163	0.021735722	0.70742017
cg13652556	0.023816205	0.107427389	0.015519894	0.743603255
cg26553682	0.017745254	59.20405009	2.028556906	1727.888203
cg19370284	0.022823251	0.553119949	0.332190117	0.920983685
cg20319405	0.034859918	105.5245186	1.392703506	7995.545341
cg27467734	0.037102908	7239.153266	1.701447987	30800436.1
cg00725635	0.048720776	0.356443098	0.127789461	0.994226604
cg11223252	0.009043895	0.009852324	0.000306919	0.316266759
cg25195673	0.01756279	0.457034724	0.239490652	0.87218744
cg21237418	0.008081352	0.069403302	0.009638695	0.499737598
cg17808849	0.009760865	0.000675168	2.66E-06	0.171446351
cg19747852	0.034650139	3057.166996	1.784618036	5237126.292
cg23797100	0.048071227	0.48203241	0.233798563	0.993826657
cg00834958	0.016345185	0.000433882	7.81E-07	0.24109714

cg02433671	0.024702045	3.046266362	1.152431984	8.052309265
cg09009042	0.034664208	2.24E-05	1.08E-09	0.462094147
cg02303361	0.030177309	0.001976995	7.10E-06	0.550563232
cg18338296	0.009510071	0.616439984	0.427647006	0.888579246
cg13620808	0.044791419	0.000183857	4.13E-08	0.818779684
cg21053748	0.032368288	0.005281429	4.33E-05	0.643547263
cg09282338	0.044592097	0.000248534	7.55E-08	0.818323321
cg14023451	0.01741352	0.310707838	0.118555159	0.814299115
cg05621401	0.0375303	5.072919296	1.098205377	23.43324001
cg16826286	0.021562518	0.446931829	0.224869444	0.88828458
cg08872550	0.040906408	0.000217363	6.70E-08	0.705648959
cg17465827	0.015451777	0.062253839	0.006579744	0.589010827
cg14974772	0.031185161	0.019687339	0.000552767	0.701183607
cg04999691	0.002725731	0.253349596	0.103223659	0.621814985
cg12556325	0.005519344	0.422570021	0.229973639	0.776460394
cg25750404	0.004695108	201847755.5	352.879106	1.15457E+14
cg21546057	0.028550942	548.6214647	1.937845436	155319.6689
cg14992108	0.012284748	0.522007928	0.313820286	0.868306764
cg05656364	0.006605963	10.40358598	1.919415419	56.389357
cg19310430	0.011633468	63.44692395	2.5250391	1594.237554
cg08108311	0.037836216	0.045332539	0.002445121	0.840465271
cg20630567	0.021904334	6.74E-10	9.68E-18	0.046913189
cg08785155	0.01171339	27594.22626	9.723457584	78309728.44
cg05724065	0.040620491	0.464119566	0.222584016	0.967755796
cg19759064	0.028577013	0.291433292	0.096641482	0.878849975
cg27318281	0.007144216	0.51125192	0.313578852	0.833533653
cg21487067	0.02963439	6.39748E+13	23.15444526	1.77E+26
cg01184449	0.049553364	0.579641046	0.336341783	0.998935484
cg01414934	0.015774284	0.174309702	0.042204952	0.719912494
cg18677965	0.001323259	1338965.481	243.7339158	7355679463
cg05125838	0.037742697	0.546679112	0.30925689	0.966374755
cg26159905	0.029645881	2.297880429	1.085655201	4.863656954
cg23141855	0.014972431	84.22612703	2.368011558	2995.779497

cg23686014	0.000532068	418.770205	13.75520531	12749.24515
cg13804316	0.000230635	66671.23113	180.5953661	24613328.45
cg25634666	0.001105646	0.367777985	0.20164261	0.670793968
cg05922591	0.022961271	0.570040403	0.351178243	0.925302372
cg21944455	0.011314743	0.507086666	0.299824651	0.857624236
cg10917602	0.030965751	0.323339228	0.115934848	0.901784564
cg13727946	0.023720123	0.536711747	0.312990212	0.920346671
cg05380982	0.038477922	2.679608966	1.053663007	6.814611658
cg19469297	0.000251003	149751.6853	254.0073852	88287067.79
cg00615377	0.013264484	0.008574863	0.000198422	0.370565154
cg07331806	0.045448731	0.492799749	0.246357032	0.985770897
cg18925884	0.04396836	0.143329066	0.021650312	0.948864894
cg01731685	0.024160311	0.056527165	0.004651124	0.686999679
cg22820108	0.033654764	0.351570757	0.134005646	0.922364101
cg04256470	0.034460222	0.449825413	0.214519576	0.943237469
cg18006085	0.044124482	0.000305679	1.16E-07	0.808271701
cg20345446	0.028051046	0.357386553	0.142695722	0.895087437
cg03431064	0.010526735	62440066.55	66.46185973	5.86616E+13
cg08390209	0.018327552	0.346220921	0.143422192	0.835776703
cg15146752	0.016060898	0.207281003	0.057566796	0.746357577
cg02376703	0.04948986	54.02879558	1.008941568	2893.240645
cg09283635	0.041097957	9.48E-06	1.43E-10	0.626499425
cg10114327	0.041906829	1.548341626	1.016133922	2.359297077
cg13707560	0.040149495	3.312898085	1.055316077	10.40000618
cg11599505	0.012777366	0.4955607	0.285160255	0.86120139
cg02314308	0.04098368	0.374128874	0.145725637	0.960520175
cg10737625	0.004990995	676957.3458	57.58537196	7958119092
cg14817848	0.036932076	42.88722015	1.256038905	1464.376338
cg04595372	0.027100925	0.52880502	0.300541074	0.930437712
cg08942800	0.04654697	0.66425813	0.444007609	0.993764194
cg11093356	0.045837496	6.88E-07	6.15E-13	0.769225975
cg09757277	0.028029552	0.166780775	0.033742322	0.824360175
cg22973042	0.014485228	0.190051037	0.050210809	0.719355007

cg20000468	0.008097791	0.063480061	0.008248529	0.488537805
cg01313949	0.030171663	10.08094586	1.248021847	81.42923912
cg11456838	0.007086717	0.498700281	0.300542807	0.82750931
cg16721845	0.032854158	6.24E-08	1.51E-14	0.258740692
cg06614002	0.013080119	0.187042823	0.049764156	0.703016402
cg13098960	0.01595632	0.57831967	0.370459708	0.902807061
cg01561916	0.01364309	1.513150301	1.088787716	2.102911156
cg18632631	0.030910193	0.140735163	0.023715715	0.835158727
cg13473383	0.022936065	7.21E-05	1.94E-08	0.267433281
cg03565782	0.012714563	137.6324448	2.860132198	6623.011998
cg17351385	0.027389038	0.016024577	0.000407138	0.630712134
cg26177629	0.023190045	3.341679593	1.179289946	9.469106846
cg01990225	0.020172882	0.067906404	0.00702132	0.656753964
cg16075384	0.020430499	0.000307215	3.30E-07	0.286424363
cg12864853	0.032562683	0.006290361	6.03E-05	0.656485143
cg04237003	0.014415173	0.521104313	0.309148786	0.878378688
cg22627427	0.02359275	0.611285274	0.399179966	0.936093288
cg09303642	0.000867921	0.041951756	0.006488937	0.271223148
cg23355725	0.038550894	10254459.77	2.340505758	4.49279E+13
cg11245384	0.038163081	1.610702026	1.026354534	2.527743515
cg05950276	0.012081635	20.03289625	1.928237152	208.1263354
cg08785534	0.04601518	4.9457774	1.028676753	23.77881488
cg26036443	0.007327962	2.287968431	1.249471888	4.189609699
cg01866521	0.02621435	21739106.86	7.383123994	6.40093E+13
cg02334775	0.042004796	0.611440962	0.380567938	0.982374006
cg09298484	0.043171233	0.598553151	0.363956385	0.984364857
cg20016845	0.040634514	0.002143804	5.97E-06	0.769529779
cg02725014	0.016593137	147370.7202	8.707014394	2494325631
cg05955301	0.035229644	2.629355715	1.069201437	6.466051429
cg19530885	0.004183354	0.450840236	0.261369173	0.777662172
cg23047271	0.043348388	0.556934255	0.31564868	0.982661371
cg15822346	0.01273172	27.97135945	2.034690673	384.5286951
cg27488807	0.037108077	0.613332324	0.387331272	0.971201055

cg02218324	0.002354266	0.547804634	0.371700006	0.807344399
cg27118825	0.010516858	0.520197993	0.31530046	0.858247882
cg06797533	0.001263047	3.168571725	1.571799703	6.38748484
cg03198372	0.044126372	0.225355709	0.052814358	0.961579337
cg23680936	0.023429998	40.38545172	1.648708548	989.249866
cg25366404	0.017906538	0.509343434	0.291380543	0.890350232
cg20773127	0.04022602	0.683896742	0.47570479	0.983203793
cg04007936	0.038686488	0.462123209	0.222307091	0.960643494
cg06528936	0.040470214	1.40E-10	5.23E-20	0.37338812
cg10294836	0.00639848	20.44026524	2.336031775	178.852209
cg09290866	0.03147044	0.64740979	0.435646303	0.962109477
cg17518825	0.04485268	0.666657174	0.448589672	0.990731207
cg05788638	0.016629633	0.513693726	0.297808429	0.886077149
cg12503243	0.037354052	0.069573011	0.005658031	0.855492611
cg23571857	0.034077488	0.539555584	0.304931066	0.954708328
cg15398448	0.007426588	7.455318047	1.712816188	32.45051487
cg13277385	0.006259594	5.25E-14	1.59E-23	0.00017412
cg26974738	0.044565883	6.26E-07	5.53E-13	0.706959153
cg02126424	0.005914687	0.014329444	0.000697154	0.294530161
cg22572159	0.009388673	9.760818024	1.749367166	54.46173356
cg24691461	0.013524516	2.007627617	1.154696222	3.490587892
cg18105675	0.009947264	0.396433653	0.19617012	0.801139548
cg04637372	0.048353274	1.433361538	1.002608659	2.049179688
cg00676660	0.021256848	7.32E-05	2.22E-08	0.241774143
cg03750606	0.000305583	30.74519643	4.787846819	197.4305235
cg21584983	0.01432454	118.4087739	2.594440336	5404.10876
cg06186861	0.0206863	0.224103195	0.063124124	0.795610918
cg08084415	0.005831471	2548.571343	9.656982068	672592.7259
cg07611334	0.005091463	19.29951878	2.432554402	153.1194635
cg16178491	0.03523767	2.385981235	1.061999969	5.360552373
cg22043361	0.004178263	881634440.7	667.4792494	1.1645E+15
cg21291896	0.002718328	6501039.538	228.4658558	1.84988E+11
cg00336605	0.049157203	0.027286602	0.000754523	0.986794206

cg20520365	0.0112756	0.003290788	3.95E-05	0.273876736
cg27431396	0.020503286	0.027363257	0.001303657	0.574344041
cg21281799	0.004159283	0.387879266	0.202956079	0.74129499
cg01861509	0.044819283	0.626088853	0.396253673	0.989233107
cg02401978	0.04659528	0.635209104	0.406260177	0.993182766
cg01804429	0.004109451	0.383243196	0.199061414	0.737839366
cg24333473	0.031703025	0.644157673	0.431237145	0.962206323
cg19166626	0.018824019	2.33E-10	2.14E-18	0.025388836
cg15240064	0.015242731	0.120468617	0.021801374	0.665677662
cg07986525	0.032456042	251.213646	1.58718278	39761.20253
cg00782174	0.020682798	0.001940965	9.79E-06	0.384898728
cg16415646	0.007898989	0.46757884	0.266846112	0.819311062
cg12598198	0.002462685	0.329643709	0.160721274	0.676108225
cg03243946	0.047822565	0.028459648	0.000838075	0.966443083
cg17860186	0.020380296	624361.7997	7.903962083	49320537329
cg06607866	0.029837389	0.057978961	0.004440153	0.757081963
cg21747271	0.04632166	5.739656801	1.028900682	32.01830921
cg27546682	0.00043421	117.5499078	8.259834076	1672.91264
cg04711324	0.049836858	0.713063585	0.508582219	0.99975905
cg03041841	0.032187781	0.029432819	0.001169055	0.741017834
cg02545106	0.020169645	0.001027204	3.09E-06	0.3410301
cg20918903	0.024183494	0.000192234	1.13E-07	0.327291013
cg19279346	0.024498588	0.604276345	0.389587301	0.937273624
cg15475323	0.016538075	0.351443251	0.149446222	0.826466915
cg02838877	0.040456688	0.425471141	0.187878328	0.963526202
cg07352600	0.019159599	7.10E-05	2.40E-08	0.210220518
cg06499565	0.026445448	0.003501779	2.38E-05	0.516105029
cg18149919	0.0059585	0.490709819	0.295444347	0.815030406
cg04491443	0.045806113	0.580605065	0.340534439	0.989921146
cg06585027	0.01653812	80.14203097	2.223339256	2888.783217
cg24793470	0.035858283	0.37616732	0.150938448	0.937480507
cg23446109	0.032699612	0.001568457	4.19E-06	0.587714716
cg21289015	0.047051824	0.131461364	0.017744987	0.973913962

cg13263114	0.010863841	0.092042789	0.014681678	0.577037255
cg22778981	0.025110368	0.249367742	0.073963024	0.84074809
cg15811427	0.030355981	0.607735556	0.387213078	0.953848223
cg23032316	0.036255507	0.230966486	0.058593733	0.910430426
cg19182048	0.046395257	0.000139007	2.23E-08	0.867743009
cg06567342	0.030114784	0.11270567	0.015670856	0.810585483
cg21032203	0.040954295	0.05991584	0.004030311	0.890727353
cg05949660	0.049855031	0.616092106	0.379685902	0.999693379
cg24735937	0.047512391	0.550136304	0.304640566	0.993465702
cg08176694	0.034179229	0.334133932	0.121158513	0.92148279
cg20051033	0.041858312	0.005472096	3.63E-05	0.825427015
cg24240626	0.013670029	0.59139739	0.389544475	0.897845805
cg27342801	0.004797401	0.564573813	0.379473375	0.839962989
cg23159337	0.045081793	0.172989419	0.031098694	0.962269951
cg00095674	0.037020957	0.00226215	7.38E-06	0.692974179
cg13581475	0.043856822	3.00E-07	1.36E-13	0.661331091
cg01656216	0.000310987	0.394761248	0.238193052	0.654244283
cg08764758	0.032471429	6.46E-07	1.37E-12	0.304143978
cg19566405	0.014096768	0.269409364	0.094545306	0.76768915
cg24712395	0.003670968	0.251160338	0.098883714	0.637936348
cg20870559	0.027081214	1.850887329	1.072230103	3.195008141
cg06206626	0.010789103	0.403095083	0.200472021	0.810515329
cg01438829	0.001188985	282914.5262	143.0001707	559724011.1
cg25683185	0.020103494	0.675420022	0.485142293	0.940326607
cg22660544	0.026978856	8.94E-06	3.00E-10	0.266134136
cg26490839	0.034049335	418138.6176	2.649037441	66001295720
cg00262415	0.032494205	3.60E-07	4.47E-13	0.290167662
cg05362669	0.010035606	30625.01603	11.77508646	79650507.07
cg12148919	0.011663153	38.88021132	2.261320875	668.4901948
cg25044651	0.012899758	1.628345122	1.108767914	2.391400221
cg10857774	0.007204736	0.498014187	0.299516844	0.82806071
cg20908204	0.00649225	0.598566298	0.413630563	0.866187474
cg19746675	0.028904773	0.001301044	3.35E-06	0.504724551

cg07845392	0.020355515	0.520890191	0.300207841	0.903795817
cg03883519	0.038153772	12.01556874	1.145444282	126.0418288
cg01606998	0.014544273	20604.48705	7.140374082	59456953.07
cg26426582	0.028151878	184.1742834	1.748664774	19397.7526
cg21900416	0.030404658	5.67E-13	4.63E-24	0.069396734
cg14870461	0.025083847	0.314647439	0.114406272	0.865363495
cg27153400	0.047578605	173.5634513	1.056591009	28510.81581
cg11806389	0.01189091	53504789526	233.8116197	1.22439E+19
cg13129046	0.041152546	13.44496026	1.110027443	162.8490876
cg23621689	0.007968888	0.002452391	2.89E-05	0.20787854
cg24098951	0.026450561	61.14651672	1.617748685	2311.172645
cg21742836	0.049396464	0.54989196	0.302859093	0.998421952
cg21452766	0.021581976	26.39561099	1.617783349	430.6684698
cg04838107	0.023437516	0.001336749	4.37E-06	0.408891807
cg15802323	0.036634263	0.031246265	0.001210942	0.806255753
cg11736869	0.031630672	0.004309805	3.00E-05	0.619253831
cg07566050	0.026665021	0.239083384	0.067452329	0.84742611
cg07186707	0.00737597	8.60E-10	2.01E-16	0.003681044
cg03794550	0.023378428	8.239965702	1.330815326	51.01912597
cg10467022	0.007521151	3.799066469	1.427600443	10.10990583
cg00047050	0.018052327	1.46E-05	1.43E-09	0.148764405
cg14338062	0.016496593	0.051317086	0.004529	0.581462437
cg14576824	0.003926522	0.293209552	0.127371854	0.674967339
cg18996334	0.040732874	8.85E-05	1.16E-08	0.674690139
cg22006386	9.71E-07	1625.863608	84.32855917	31346.82363
cg02863947	0.037209468	0.314042656	0.105631303	0.933651171
cg17740645	0.00253172	0.145703808	0.04173327	0.508697251
cg06765947	0.011688343	0.190483156	0.052489202	0.691262805
cg14456683	0.004652649	0.59728189	0.418000402	0.853457688
cg07967308	0.017043119	0.042941003	0.003234065	0.570158589
cg10021735	0.030512469	0.000416858	3.61E-07	0.481057611
cg14056306	0.02068819	3.68E-05	6.45E-09	0.209990906
cg13433272	0.047850877	2.97E-05	9.76E-10	0.906064356

cg12228611	0.031647234	94.5598218	1.49161446	5994.551635
cg09526693	0.036794061	1.700021619	1.033084356	2.797519378
cg17296078	0.003328388	0.015425585	0.000952005	0.249944688
cg25752527	0.043989253	1.78E-10	5.79E-20	0.546380734
cg04663487	0.025954693	0.588075719	0.36855582	0.938346466
cg00520708	0.00365524	0.30125155	0.134137004	0.676565702
cg03112869	0.019911367	0.047199397	0.003610093	0.617098569
cg06285340	0.023591762	0.188911322	0.044630218	0.799626119
cg00344358	0.048042852	0.132256947	0.017799305	0.982729364
cg15932716	0.018140834	55.8503897	1.985225332	1571.240292
cg13501507	0.038339595	0.000127638	2.64E-08	0.617814021
cg03986640	0.041589255	0.361257457	0.13566975	0.961945831
cg05955224	0.02875863	0.033004754	0.001551701	0.702012655
cg10409799	0.004035959	14.43213115	2.339250851	89.03979219
cg09737668	0.030107069	0.119290043	0.017461299	0.814951652
cg07837085	0.011716994	0.591428526	0.393129144	0.889752661
cg15076659	0.018204917	0.24317618	0.075203853	0.786324797
cg15488402	0.037638044	0.121635936	0.016688467	0.886558408
cg00655307	0.008456997	1.26E-05	2.85E-09	0.055893612
cg19459675	0.021803831	965807.2248	7.429507216	1.25551E+11
cg07403350	0.047074908	0.367654053	0.136928816	0.987151623
cg21142158	0.037060514	0.000953947	1.38E-06	0.658767719
cg26266098	0.017418053	0.035972021	0.00232093	0.557529153
cg14824983	0.044660265	0.53133121	0.286602009	0.985034458
cg24816455	0.022624459	1.627296669	1.070681608	2.473279104
cg08448751	0.003624068	0.09467769	0.019342938	0.463417964
cg10534672	0.025077598	0.012331118	0.00026352	0.5770209
cg17171485	0.0099234	1386.188776	5.670939878	338836.1301
cg00708598	0.002700202	2.281948458	1.331108383	3.911994568
cg09015232	0.033543383	0.585600765	0.357523452	0.959176957
cg18854666	0.032882409	0.659226253	0.449562147	0.96667225
cg25457331	0.011552489	30.68541703	2.152690139	437.4037865
cg17071957	0.022399703	0.311974045	0.114793251	0.847853022

cg22495124	0.014083734	0.410036899	0.201248657	0.83543543
cg00469635	0.017035401	50.70946198	2.015678582	1275.723996
cg18711066	0.031738764	0.463709971	0.22996675	0.9350349
cg16026550	0.006017041	22.12225152	2.428016877	201.5612071
cg15558658	0.029370382	115872.5618	3.220691559	4168809817
cg03799530	0.044945306	0.0320483	0.001109924	0.92537276
cg24652919	0.013816565	0.385178349	0.18022403	0.823210757
cg06290096	0.001204634	0.292995093	0.139356946	0.616016114
cg11102794	0.043322852	0.071090234	0.005471003	0.923746719
cg01044662	0.018032461	0.022495476	0.00096914	0.522160374
cg27394566	0.021322208	0.025626567	0.001132286	0.579995388
cg01843018	0.022663467	3.285323439	1.181210508	9.137533088
cg16812893	0.0378529	1.550392301	1.024903555	2.345309738
cg14271690	0.030835548	0.397795285	0.172289162	0.918462236
cg18710692	0.014296623	1.12E-05	1.23E-09	0.102423992
cg07510080	0.023480334	0.08048963	0.009100566	0.711887671
cg19007602	0.044911059	4.69E-08	3.23E-15	0.68178097
cg04312209	0.026403958	0.449565343	0.221965415	0.910542739
cg13474734	0.006740991	0.397905735	0.204296526	0.774995918
cg12213910	0.037935598	0.133876991	0.020046799	0.894060403
cg22016649	0.007551107	0.109687421	0.021675232	0.555072734
cg24427660	0.022227197	0.308899111	0.112845878	0.845566207
cg21248774	0.037722919	54543.24612	1.856706211	1602281330
cg15227982	0.041696126	0.186816537	0.037172887	0.93886758
cg00692549	0.047835716	0.05088032	0.002663349	0.972011945
cg26393983	0.024557182	2.57341E+13	52.46114756	1.26E+25
cg10920427	0.011638318	0.003406572	4.12E-05	0.281426907
cg22041658	0.038625184	1.92E-10	1.18E-19	0.310201133
cg08397758	0.009664109	0.000162487	2.19E-07	0.120473114
cg22436229	0.043881008	0.00119369	1.71E-06	0.831461717
cg06638451	0.037098214	0.110497166	0.013929467	0.876532015
cg12958813	0.012623323	1.664266546	1.115284387	2.483477012
cg10791955	0.042916184	3.46E-13	2.99E-25	0.400518653

cg13650156	0.047161948	0.310876128	0.098069712	0.985461926
cg21721155	0.022099651	0.000878298	2.12E-06	0.363963802
cg07519011	0.010736767	0.00164502	1.20E-05	0.226408845
cg01610488	0.009201933	1.896647347	1.171629022	3.070315852
cg24682149	0.041578083	5.70E-06	5.15E-11	0.63080574
cg00168785	0.024692814	0.000315914	2.79E-07	0.358071729
cg02704907	0.021327721	0.475383307	0.252399874	0.895362128
cg07846220	0.010342784	7.571406847	1.611237863	35.57898122
cg04809136	0.013855024	123.7203042	2.666865718	5739.589197
cg05960024	0.008531147	0.378985645	0.183921815	0.780930302
cg03046445	0.04835319	85.55100181	1.032721105	7087.076919
cg22283058	0.003927086	0.269388721	0.110474074	0.656898771
cg21023770	0.044496722	0.375829768	0.144688729	0.976219887
cg02782630	0.034010922	0.042108784	0.002251931	0.7873909
cg01459453	0.036862538	0.619984725	0.395754845	0.971260527
cg04113075	0.004312967	0.000201937	5.86E-07	0.069550708
cg01278291	0.000865557	0.14879988	0.048501047	0.456513941
cg02357714	0.003397158	0.307995324	0.140066885	0.677255866
cg17960516	0.047829913	9.570049895	1.021820918	89.63004513
cg00503458	0.040540815	0.022432678	0.000592656	0.84910134
cg24063470	0.040227326	4.652889371	1.07093781	20.21534705
cg27662379	0.043076583	99.09715732	1.153890919	8510.550198
cg03534410	0.027273237	0.426321333	0.199988878	0.908799936
cg12259537	0.005868472	2.179843025	1.252178179	3.79475996
cg13307384	0.036559455	0.498304031	0.259357221	0.95739346
cg00766889	0.026571874	133720842.6	8.801781777	2.03155E+15
cg16173067	0.036735864	0.561335619	0.326512893	0.965039003
cg07576541	0.011545699	0.51868552	0.311652291	0.86325266
cg11694641	0.032190743	2974.989509	1.973520574	4484656.859
cg17356733	0.011903527	0.657407046	0.474100497	0.911587368
cg18325289	0.000265066	136283.2348	237.2937209	78270592.36
cg02608292	0.017173429	1241.33535	3.541657294	435082.5965
cg10693071	0.041494423	0.592983958	0.358786132	0.98005453

cg11593656	0.029774905	0.124056853	0.018885477	0.814917359
cg20880234	0.003676125	3.009223845	1.430914657	6.328419455
cg03752087	0.02185939	0.616924898	0.408240286	0.932285084
cg03679305	0.040883988	0.572289912	0.33517956	0.97713519
cg05610379	0.021349438	84.36066146	1.932011724	3683.580752
cg06495347	0.0095868	0.007610825	0.000189872	0.305072748
cg07172280	0.028484408	0.007841546	0.000102435	0.600283985
cg00431050	0.004493414	0.410685964	0.222280531	0.758784226
cg18390025	0.025086383	0.660547223	0.459544501	0.949467643
cg25322008	0.015396234	0.534231867	0.321723958	0.88710735
cg17389295	0.031329466	0.000170992	6.36E-08	0.459695885
cg15983538	0.043965763	2.193350879	1.021458502	4.709724448
cg05322019	0.00303259	0.214176588	0.077321924	0.593254908
cg14990333	0.049082748	0.018146246	0.000334635	0.984017178
cg22992570	0.044258243	1.12E-08	2.02E-16	0.624896189
cg08151470	0.025964707	0.3079944	0.10923478	0.86840977
cg26792080	0.030759953	0.049032785	0.003179337	0.75619978
cg04310489	0.029519284	99.45905148	1.580252941	6259.822503
cg09382492	0.02938008	0.409956499	0.183780546	0.914483794
cg04431054	0.014554806	0.155654656	0.035004494	0.69215032
cg09092161	0.014190026	1.03E-10	1.07E-18	0.009875557
cg06744574	0.001305601	22.3175582	3.360506378	148.2137952
cg04916911	0.039909311	0.431100873	0.193198589	0.961953004
cg13015534	0.039899527	2.992702962	1.051887295	8.514477797
cg15503752	0.022081518	2.654131929	1.150618938	6.12228433
cg22800191	0.047323153	4.77E-05	2.56E-09	0.889094771
cg04315264	0.011428227	16.25828482	1.873344066	141.1015895
cg04536922	0.041611053	0.137891808	0.020501062	0.927471523
cg27305303	0.00902759	1.974682232	1.184898005	3.290890779
cg10289190	0.001473839	2.4146E+12	56270.08441	1.04E+20
cg14163776	0.033832622	0.372294675	0.149469344	0.927302687
cg26044825	0.036862692	92.93119969	1.318413438	6550.454986
cg14070162	0.018228464	0.085449208	0.011088013	0.65851

Supplementary table 2. The samples were clustered into two major groups at k=2

Samples	Cluster
TCGA-61-1910-01	C1
TCGA-31-1951-01	C1
TCGA-13-1477-01	C1
TCGA-13-0924-01	C1
TCGA-23-1021-01	C1
TCGA-23-2084-01	C1
TCGA-04-1516-01	C1
TCGA-24-1556-01	C1
TCGA-09-1666-01	C1
TCGA-25-2409-01	C1
TCGA-09-1662-01	C1
TCGA-57-1584-01	C1
TCGA-13-1485-01	C1
TCGA-29-1688-01	C1
TCGA-13-0804-01	C1
TCGA-36-2542-01	C1
TCGA-13-0903-01	C1
TCGA-20-1687-01	C1
TCGA-24-2038-01	C1
TCGA-23-2081-01	C1

TCGA-20-0996-01	C1
TCGA-29-2436-01	C1
TCGA-13-1404-01	C1
TCGA-04-1335-01	C1
TCGA-13-0766-01	C1
TCGA-29-1770-01	C1
TCGA-24-1845-01	C1
TCGA-30-1860-01	C1
TCGA-13-2071-01	C1
TCGA-13-2065-01	C1
TCGA-24-1466-01	C1
TCGA-61-1995-01	C1
TCGA-24-1847-01	C1
TCGA-09-1670-01	C1
TCGA-13-0884-01	C1
TCGA-13-1497-01	C1
TCGA-13-0913-01	C1
TCGA-24-1471-01	C1
TCGA-61-2097-01	C1
TCGA-13-2059-01	C1
TCGA-09-1667-01	C1
TCGA-29-1776-01	C1

TCGA-36-2532-01	C1
TCGA-04-1353-01	C1
TCGA-24-1435-01	C1
TCGA-36-2540-01	C1
TCGA-29-1690-01	C1
TCGA-36-2544-01	C1
TCGA-09-1672-01	C1
TCGA-09-1675-01	C1
TCGA-13-0906-01	C1
TCGA-61-1903-01	C1
TCGA-24-1463-01	C1
TCGA-23-1028-01	C1
TCGA-25-2408-01	C1
TCGA-04-1649-01	C1
TCGA-61-1913-01	C1
TCGA-24-1417-01	C1
TCGA-24-1614-01	C1
TCGA-24-1470-01	C1
TCGA-13-0916-01	C1
TCGA-42-2589-01	C1
TCGA-61-1901-01	C1
TCGA-61-2018-01	C1

TCGA-10-0937-01	C1
TCGA-04-1367-01	C1
TCGA-29-1781-01	C1
TCGA-24-1616-01	C1
TCGA-61-1737-01	C1
TCGA-13-1482-01	C1
TCGA-31-1956-01	C1
TCGA-23-2645-01	C1
TCGA-13-0801-01	C1
TCGA-29-1764-01	C1
TCGA-29-1696-01	C1
TCGA-04-1654-01	C1
TCGA-04-1536-01	C1
TCGA-23-1027-01	C1
TCGA-04-1651-01	C1
TCGA-24-1563-01	C1
TCGA-61-1725-01	C1
TCGA-36-2549-01	C1
TCGA-24-0980-01	C1
TCGA-13-1511-01	C1
TCGA-24-1846-01	C1
TCGA-09-2045-01	C1

TCGA-59-2348-01	C1
TCGA-36-1570-01	C1
TCGA-61-2095-01	C1
TCGA-13-0897-01	C1
TCGA-24-1103-01	C1
TCGA-24-2298-01	C1
TCGA-25-1328-01	C1
TCGA-29-1768-01	C1
TCGA-13-0900-01	C1
TCGA-24-1428-01	C1
TCGA-13-0720-01	C1
TCGA-24-2254-01	C1
TCGA-09-1668-01	C1
TCGA-25-1322-01	C1
TCGA-23-2079-01	C1
TCGA-24-2271-01	C1
TCGA-61-1914-01	C1
TCGA-24-2036-01	C1
TCGA-13-0797-01	C1
TCGA-61-2087-01	C1
TCGA-13-0887-01	C1
TCGA-13-1483-01	C1

TCGA-13-0799-01	C1
TCGA-24-1604-01	C1
TCGA-23-1026-01	C1
TCGA-61-2017-01	C1
TCGA-29-1711-01	C1
TCGA-24-1927-01	C1
TCGA-13-1817-01	C1
TCGA-25-2391-01	C1
TCGA-10-0925-01	C1
TCGA-61-2110-01	C1
TCGA-36-1574-01	C1
TCGA-25-2401-01	C1
TCGA-20-1682-01	C1
TCGA-13-0905-01	C1
TCGA-04-1331-01	C1
TCGA-13-1405-01	C1
TCGA-09-2043-01	C1
TCGA-13-0725-01	C1
TCGA-24-1104-01	C1
TCGA-23-2649-01	C1
TCGA-29-1771-01	C1
TCGA-29-1707-01	C1

TCGA-23-2078-01	C1
TCGA-24-1545-01	C1
TCGA-23-1118-01	C1
TCGA-61-1728-01	C2
TCGA-31-1953-01	C2
TCGA-13-1819-01	C2
TCGA-25-2392-01	C2
TCGA-24-1552-01	C2
TCGA-30-1862-01	C2
TCGA-29-1698-01	C2
TCGA-25-1326-01	C2
TCGA-09-1659-01	C2
TCGA-09-2051-01	C2
TCGA-13-1495-01	C2
TCGA-24-2281-01	C2
TCGA-61-1918-01	C2
TCGA-24-0982-01	C2
TCGA-23-1109-01	C2
TCGA-24-1924-01	C2
TCGA-61-1724-01	C2
TCGA-24-1426-01	C2
TCGA-23-1116-01	C2

TCGA-25-2398-01	C2
TCGA-29-1778-01	C2
TCGA-04-1343-01	C2
TCGA-13-0791-01	C2
TCGA-24-1423-01	C2
TCGA-42-2582-01	C2
TCGA-13-1496-01	C2
TCGA-25-1626-01	C2
TCGA-20-0987-01	C2
TCGA-24-2024-01	C2
TCGA-04-1644-01	C2
TCGA-10-0931-01	C2
TCGA-25-1623-01	C2
TCGA-29-1774-01	C2
TCGA-61-2016-01	C2
TCGA-13-0795-01	C2
TCGA-61-2113-01	C2
TCGA-13-1492-01	C2
TCGA-24-1565-01	C2
TCGA-24-2260-01	C2
TCGA-13-2057-01	C2
TCGA-36-2552-01	C2

TCGA-23-1024-01	C2
TCGA-09-0365-01	C2
TCGA-13-0885-01	C2
TCGA-24-1928-01	C2
TCGA-25-1315-01	C2
TCGA-04-1371-01	C2
TCGA-31-1955-01	C2
TCGA-36-1571-01	C2
TCGA-13-1489-01	C2
TCGA-29-1694-01	C2
TCGA-30-1892-01	C2
TCGA-25-1319-01	C2
TCGA-04-1349-01	C2
TCGA-61-1722-01	C2
TCGA-20-1683-01	C2
TCGA-23-1031-01	C2
TCGA-13-0755-01	C2
TCGA-04-1652-01	C2
TCGA-36-1575-01	C2
TCGA-36-1568-01	C2
TCGA-31-1959-01	C2
TCGA-57-1993-01	C2

TCGA-09-0369-01	C2
TCGA-09-2053-01	C2
TCGA-04-1341-01	C2
TCGA-25-1323-01	C2
TCGA-13-1512-01	C2
TCGA-13-0802-01	C2
TCGA-29-1761-01	C2
TCGA-61-1738-01	C2
TCGA-25-2400-01	C2
TCGA-29-2434-01	C2
TCGA-13-0751-01	C2
TCGA-61-1899-01	C2
TCGA-59-2351-01	C2
TCGA-13-0894-01	C2
TCGA-04-1356-01	C2
TCGA-61-1734-01	C2
TCGA-24-1548-01	C2
TCGA-25-1632-01	C2
TCGA-13-1408-01	C2
TCGA-25-1877-01	C2
TCGA-29-1691-01	C2
TCGA-61-2101-01	C2

TCGA-04-1365-01	C2
TCGA-59-2355-01	C2
TCGA-25-1634-01	C2
TCGA-10-0927-01	C2
TCGA-13-0890-01	C2
TCGA-24-1544-01	C2
TCGA-23-1122-01	C2
TCGA-24-1849-01	C2
TCGA-13-1410-01	C2
TCGA-13-0920-01	C2
TCGA-13-0714-01	C2
TCGA-24-1422-01	C2
TCGA-24-0968-01	C2
TCGA-61-2102-01	C2
TCGA-24-1553-01	C2
TCGA-24-1603-01	C2
TCGA-36-1581-01	C2
TCGA-61-2613-01	C2
TCGA-13-0727-01	C2
TCGA-24-1557-01	C2
TCGA-04-1369-01	C2
TCGA-61-2000-01	C2

TCGA-23-1111-01	C2
TCGA-04-1337-01	C2
TCGA-30-1859-01	C2
TCGA-61-2002-01	C2
TCGA-25-2404-01	C2
TCGA-29-1702-01	C2
TCGA-04-1348-01	C2
TCGA-13-0919-01	C2
TCGA-61-2009-01	C2
TCGA-10-0933-01	C2
TCGA-24-1427-01	C2
TCGA-30-1855-01	C2
TCGA-04-1648-01	C2
TCGA-09-2056-01	C2
TCGA-24-2293-01	C2
TCGA-04-1361-01	C2
TCGA-13-0794-01	C2
TCGA-24-1551-01	C2
TCGA-25-2393-01	C2
TCGA-09-2044-01	C2
TCGA-24-2035-01	C2
TCGA-57-1585-01	C2

TCGA-24-1105-01	C2
TCGA-20-1685-01	C2
TCGA-10-0936-01	C2
TCGA-24-1923-01	C2
TCGA-13-1484-01	C2
TCGA-23-1114-01	C2
TCGA-04-1530-01	C2
TCGA-42-2588-01	C2
TCGA-61-2088-01	C2
TCGA-61-1740-01	C2
TCGA-25-2042-01	C2
TCGA-24-1920-01	C2
TCGA-61-2092-01	C2
TCGA-61-1916-01	C2
TCGA-61-2096-01	C2
TCGA-36-1569-01	C2
TCGA-24-2029-01	C2
TCGA-36-2548-01	C2
TCGA-29-2431-01	C2
TCGA-09-1669-01	C2
TCGA-25-1871-01	C2
TCGA-61-1730-01	C2

TCGA-24-2020-01	C2
TCGA-04-1655-01	C2
TCGA-25-1313-01	C2
TCGA-23-2643-01	C2
TCGA-13-0899-01	C2
TCGA-13-0768-01	C2
TCGA-59-2350-01	C2
TCGA-29-2428-01	C2
TCGA-23-1123-01	C2
TCGA-13-0889-01	C2
TCGA-59-2354-01	C2
TCGA-13-0803-01	C2
TCGA-24-1413-01	C2
TCGA-61-2109-01	C2
TCGA-04-1514-01	C2
TCGA-04-1338-01	C2
TCGA-24-1555-01	C2
TCGA-61-2098-01	C2
TCGA-29-1769-01	C2
TCGA-25-1325-01	C2
TCGA-24-1567-01	C2
TCGA-24-2023-01	C2

TCGA-24-1474-01	C2
TCGA-23-1032-01	C2
TCGA-42-2590-01	C2
TCGA-59-2352-01	C2
TCGA-36-1577-01	C2
TCGA-24-0979-01	C2
TCGA-13-1500-01	C2
TCGA-13-2060-01	C2
TCGA-13-0726-01	C2
TCGA-24-1467-01	C2
TCGA-04-1332-01	C2
TCGA-13-0792-01	C2
TCGA-13-1510-01	C2
TCGA-13-1407-01	C2
TCGA-24-0970-01	C2
TCGA-25-1320-01	C2
TCGA-24-1424-01	C2
TCGA-09-0367-01	C2
TCGA-24-0975-01	C2
TCGA-13-0908-01	C2
TCGA-59-2349-01	C2
TCGA-25-1317-01	C2

TCGA-25-1635-01	C2
TCGA-13-0762-01	C2
TCGA-29-1763-01	C2
TCGA-25-1329-01	C2
TCGA-61-2008-01	C2
TCGA-24-1546-01	C2
TCGA-25-1630-01	C2
TCGA-20-0991-01	C2
TCGA-13-1505-01	C2
TCGA-09-2048-01	C2
TCGA-24-1560-01	C2
TCGA-30-1861-01	C2
TCGA-30-1856-01	C2
TCGA-04-1525-01	C2
TCGA-59-2372-01	C2
TCGA-23-1121-01	C2
TCGA-36-2529-01	C2
TCGA-23-1023-01	C2
TCGA-13-2061-01	C2
TCGA-36-2551-01	C2
TCGA-30-1891-01	C2
TCGA-29-2414-01	C2

TCGA-13-0912-01	C2
TCGA-24-1843-01	C2
TCGA-36-1580-01	C2
TCGA-30-1869-01	C2
TCGA-20-1684-01	C2
TCGA-13-1488-01	C2
TCGA-09-2050-01	C2
TCGA-30-1880-01	C2
TCGA-30-1867-01	C2
TCGA-29-1710-01	C2
TCGA-24-1436-01	C2
TCGA-24-2295-01	C2
TCGA-57-1992-01	C2
TCGA-04-1351-01	C2
TCGA-61-1736-01	C2
TCGA-09-1665-01	C2
TCGA-36-2538-01	C2
TCGA-24-1852-01	C2
TCGA-23-2641-01	C2
TCGA-61-1900-01	C2
TCGA-13-1499-01	C2
TCGA-61-1915-01	C2

TCGA-29-1704-01	C2
TCGA-61-1727-01	C2
TCGA-29-2427-01	C2
TCGA-29-2425-01	C2
TCGA-57-1583-01	C2
TCGA-24-1550-01	C2
TCGA-13-0886-01	C2
TCGA-25-1625-01	C2
TCGA-24-2288-01	C2
TCGA-25-1312-01	C2
TCGA-09-2055-01	C2
TCGA-10-0926-01	C2
TCGA-13-0807-01	C2
TCGA-13-0730-01	C2
TCGA-04-1519-01	C2
TCGA-25-1870-01	C2
TCGA-61-2104-01	C2
TCGA-23-2647-01	C2
TCGA-13-0888-01	C2
TCGA-24-2033-01	C2
TCGA-24-2290-01	C2
TCGA-10-0934-01	C2

TCGA-61-1906-01	C2
TCGA-04-1346-01	C2
TCGA-57-1994-01	C2
TCGA-25-2399-01	C2
TCGA-25-2396-01	C2
TCGA-13-0717-01	C2
TCGA-36-1576-01	C2
TCGA-61-2612-01	C2
TCGA-13-1403-01	C2
TCGA-24-1431-01	C2
TCGA-23-1110-01	C2
TCGA-24-2262-01	C2
TCGA-04-1342-01	C2
TCGA-04-1336-01	C2
TCGA-29-1784-01	C2
TCGA-42-2591-01	C2
TCGA-29-1777-01	C2
TCGA-09-1661-01	C2
TCGA-29-2429-01	C2
TCGA-61-2111-01	C2
TCGA-23-2072-01	C2
TCGA-23-1119-01	C2

TCGA-24-1425-01	C2
TCGA-13-0764-01	C2
TCGA-29-1701-01	C2
TCGA-24-2027-01	C2
TCGA-09-0366-01	C2
TCGA-24-1850-01	C2
TCGA-25-1316-01	C2
TCGA-61-1733-01	C2
TCGA-20-0990-01	C2
TCGA-36-2534-01	C2
TCGA-13-0793-01	C2
TCGA-13-1498-01	C2
TCGA-25-1631-01	C2
TCGA-36-2537-01	C2
TCGA-24-2019-01	C2
TCGA-13-0893-01	C2
TCGA-09-1673-01	C2
TCGA-09-2049-01	C2
TCGA-13-0910-01	C2
TCGA-31-1946-01	C2
TCGA-23-1124-01	C2
TCGA-25-1321-01	C2

TCGA-29-1692-01	C2
TCGA-30-1857-01	C2
TCGA-24-1416-01	C2
TCGA-25-1324-01	C2
TCGA-23-1107-01	C2
TCGA-04-1364-01	C2
TCGA-23-1022-01	C2
TCGA-13-0923-01	C2
TCGA-61-2614-01	C2
TCGA-23-1120-01	C2
TCGA-23-1113-01	C2
TCGA-42-2587-01	C2
TCGA-13-1504-01	C2
TCGA-13-0904-01	C2
TCGA-29-1783-01	C2
TCGA-13-1506-01	C2
TCGA-13-1501-01	C2
TCGA-13-1507-01	C2
TCGA-09-1674-01	C2
TCGA-13-0724-01	C2
TCGA-24-1469-01	C2
TCGA-61-1917-01	C2

TCGA-24-2297-01	C2
TCGA-09-1664-01	C2
TCGA-13-0758-01	C2
TCGA-13-0883-01	C2
TCGA-13-1412-01	C2
TCGA-61-1741-01	C2
TCGA-04-1646-01	C2
TCGA-24-1434-01	C2
TCGA-25-1633-01	C2
TCGA-13-0760-01	C2
TCGA-29-1705-01	C2
TCGA-13-2066-01	C2
TCGA-04-1362-01	C2
TCGA-13-1509-01	C2
TCGA-13-1494-01	C2
TCGA-04-1350-01	C2
TCGA-57-1582-01	C2
TCGA-25-1627-01	C2
TCGA-29-1785-01	C2
TCGA-61-1911-01	C2
TCGA-61-2012-01	C2
TCGA-36-2545-01	C2

TCGA-29-1699-01	C2
TCGA-24-1930-01	C2
TCGA-31-1950-01	C2
TCGA-57-1586-01	C2
TCGA-13-1491-01	C2
TCGA-24-2267-01	C2
TCGA-04-1517-01	C2
TCGA-61-1904-01	C2
TCGA-10-0938-01	C2
TCGA-30-1718-01	C2
TCGA-13-0921-01	C2
TCGA-24-2280-01	C2
TCGA-13-1487-01	C2
TCGA-29-1695-01	C2
TCGA-29-1697-01	C2
TCGA-25-1318-01	C2
TCGA-24-1562-01	C2
TCGA-13-1411-01	C2
TCGA-30-1887-01	C2
TCGA-30-1714-01	C2
TCGA-13-0757-01	C2
TCGA-09-2054-01	C2

TCGA-61-1919-01	C2
TCGA-24-1558-01	C2
TCGA-23-1117-01	C2
TCGA-61-2003-01	C2
TCGA-24-1418-01	C2
TCGA-23-2077-01	C2
TCGA-24-1430-01	C2
TCGA-61-1895-01	C2
TCGA-04-1347-01	C2
TCGA-09-0364-01	C2
TCGA-10-0930-01	C2
TCGA-29-1775-01	C2
TCGA-24-0966-01	C2
TCGA-24-2261-01	C2
TCGA-13-0800-01	C2
TCGA-25-1314-01	C2
TCGA-13-0765-01	C2
TCGA-13-1409-01	C2
TCGA-29-1762-01	C2
TCGA-24-2026-01	C2
TCGA-10-0928-01	C2
TCGA-13-0805-01	C2

TCGA-61-1998-01	C2
TCGA-23-1029-01	C2
TCGA-61-1721-01	C2
TCGA-36-2543-01	C2
TCGA-20-1686-01	C2
TCGA-24-1549-01	C2
TCGA-25-2397-01	C2
TCGA-29-1766-01	C2
TCGA-59-2363-01	C2
TCGA-25-1878-01	C2
TCGA-23-1030-01	C2
TCGA-29-1693-01	C2
TCGA-13-0901-01	C2
TCGA-30-1866-01	C2
TCGA-61-1907-01	C2
TCGA-24-2030-01	C2
TCGA-29-2432-01	C2
TCGA-24-1564-01	C2
TCGA-30-1853-01	C2
TCGA-13-0911-01	C2
TCGA-36-1578-01	C2
TCGA-24-1842-01	C2

TCGA-23-1809-01	C2
TCGA-24-1464-01	C2
TCGA-29-1703-01	C2
TCGA-61-1743-01	C2
TCGA-42-2593-01	C2
TCGA-31-1944-01	C2
TCGA-04-1542-01	C2
TCGA-61-2094-01	C2
TCGA-25-1628-01	C2
TCGA-24-1844-01	C2
TCGA-24-1419-01	C2
TCGA-13-0891-01	C2
TCGA-24-2289-01	C2
TCGA-13-1481-01	C2
TCGA-13-0761-01	C2
TCGA-10-0935-01	C2
TCGA-04-1638-01	C2
TCGA-36-2547-01	C2
TCGA-13-0723-01	C2

Supplementary table 3. The C2 molecular subtypes were subdivided into 1350 sites in

hypermethylation state and 1378 in hypomethylation state

	intercept	f	pval	qval	beta_FC
cg00514407	0.294649338	210.8574767	7.89E-41	7.95E-37	-1.786724397
cg16979445	0.58288168	176.1876051	3.42E-35	1.72E-31	-1.541009416
cg00626466	0.43892309	164.8149525	2.77E-33	9.30E-30	-1.55370265
cg01531431	0.802402777	153.530607	2.32E-31	5.85E-28	-1.365422184
cg17641252	0.671028025	151.7110283	4.78E-31	9.62E-28	-1.416976258
cg16858125	0.383547144	141.3835289	2.96E-29	4.27E-26	-1.570797779
cg09816471	0.311208068	138.0423689	1.14E-28	1.44E-25	-1.622213847
cg18236734	0.454620104	133.6003652	6.93E-28	6.98E-25	-1.533029916
cg15046693	0.497174023	132.2597422	1.20E-27	1.10E-24	-1.488431441
cg06044899	0.462887364	131.4232471	1.68E-27	1.41E-24	1.260788917
cg09971811	0.700133802	130.9992005	2.00E-27	1.55E-24	-1.442983422
cg11793332	0.362260717	126.3348036	1.36E-26	8.56E-24	1.534208532
cg09492887	0.571124772	125.9711983	1.58E-26	9.35E-24	-1.536313802

cg26174752	0.368265412	123.9059832	3.70E-26	1.97E-23	-1.556295849
cg17240454	0.444100784	123.8994286	3.71E-26	1.97E-23	1.4109434
cg21184174	0.448471606	121.7151711	9.17E-26	4.43E-23	-1.484892867
cg24926276	0.189953415	121.6977224	9.23E-26	4.43E-23	1.860191913
cg17644208	0.314806388	121.5059065	1.00E-25	4.58E-23	1.360777018
cg16379513	0.960458469	119.8368449	2.00E-25	8.10E-23	-1.211342021
cg18390025	0.67685427	119.825042	2.01E-25	8.10E-23	-1.400993591
cg22809047	0.375833181	118.0060298	4.29E-25	1.58E-22	-1.631199745
cg20892287	0.317823303	117.9436673	4.40E-25	1.58E-22	1.438744616
cg03866607	0.533123817	113.381158	2.97E-24	9.98E-22	1.208019205
cg21541083	0.717194866	112.0797433	5.13E-24	1.57E-21	-1.314031051
cg21762589	0.493782645	110.8845024	8.49E-24	2.52E-21	-1.467508469
cg20422318	0.323335109	110.3089736	1.08E-23	3.12E-21	1.344139892
cg17356733	0.743163546	110.0527138	1.21E-23	3.38E-21	-1.393968588
cg03294491	0.379084236	106.0537123	6.56E-23	1.79E-20	-1.516679215
cg18486150	0.434313916	105.4549367	8.47E-23	2.24E-20	-1.456850502
cg07080946	0.597414369	104.8551557	1.09E-22	2.82E-20	-

1.422790528

cg02477931 0.579746375 103.8570203 1.67E-22 4.21E-20 -

1.441366986

cg19728382 0.862107457 103.0291361 2.38E-22 5.85E-20 -

1.295930072

cg18674980 0.458189181 101.86811843.91E-22 9.16E-20 -1.540436179

cg25645462 0.315992399 100.4990879 7.02E-22 1.61E-19 1.38314101

cg02889982 0.39314101 100.0242692 8.61E-22 1.93E-19 1.272644992

cg03387723 0.732813076 99.90137944 9.08E-22 1.99E-19 -

1.341433862

cg08465774 0.595760008 99.70828327 9.86E-22 2.11E-19

1.254818744

cg05046097 0.291385054 98.87190619 1.41E-21 2.90E-19

1.400635179

cg12461141 0.37655123 98.032611482.03E-21 4.08E-19 1.418528077

cg13320683 0.844484343 96.70489101 3.59E-21 6.82E-19 -

1.248337765

cg22584138 0.614380691 95.97250629 4.92E-21 9.19E-19 -

1.357458158

cg07715201 0.144262069 95.46924372 6.12E-21 1.12E-18 -

1.662706888

cg13474734 0.446717173 95.38986653 6.33E-21 1.14E-18 1.33209996

cg26453588 0.541222704 92.50348408 2.21E-20 3.91E-18 -

1.344546257

cg05113558 0.767329965 91.30671332 3.72E-20 6.35E-18 -

1.273180882

cg11147193 0.539726233 91.17871778 3.93E-20 6.60E-18 -

1.363389845

cg18741908 0.347192912 89.09802821 9.74E-20 1.49E-17 -

1.498854421

cg18493147 0.874098065 88.76174774 1.13E-19 1.68E-17 -

1.247618408

cg16302441 0.261841923 88.74949205 1.13E-19 1.68E-17 -

1.495845493

cg04049033 0.500827556 88.63391706 1.19E-19 1.74E-17

1.235504958

cg00509616 0.130186669 87.82543943 1.70E-19 2.44E-17 -

1.575456938

cg21794225 0.454657163 87.27995056 2.16E-19 3.06E-17 -

1.371615317

cg00328227 0.364945761 84.811665886.37E-19 8.23E-17 -1.483595371

cg10057065 0.250139642 84.63760641 6.88E-19 8.77E-17 -

1.527024385

cg09676390 0.346088062 84.37816232 7.71E-19 9.59E-17

1.251358461

cg00347904 0.23477673 83.88890729 9.56E-19 1.13E-16 -1.625904143

cg06550629 0.659709582 83.54574103 1.11E-18 1.29E-16 -

1.399993294

cg15210999 0.606502424 82.26571033 1.96E-18 2.21E-16 -

1.323067662

cg20395892 0.444372928 82.20693817 2.01E-18 2.22E-16 -

1.424306024

cg16003238 0.506709462 81.97588174 2.22E-18 2.43E-16 -

1.319493808

cg19562969 0.438313402 81.92055073 2.28E-18 2.47E-16 -

1.403855107

cg21960110 0.708857747 81.78949751 2.41E-18 2.59E-16 -

1.341478621

cg26316946 0.402691268 81.61683197 2.61E-18 2.76E-16

1.549327283

cg22705225 0.45105112881.29946839 3.00E-18 3.11E-16 -1.304546169

cg22919728 0.250841891 80.85198983 3.65E-18 3.68E-16 -

1.504598793

cg22449114 0.51168147580.40805442 4.45E-18 4.44E-16 -1.402667577

cg13603171 0.494656164 79.54185699 6.53E-18 6.39E-16 -

1.485592419

cg15101633	0.445708221	78.96932246	8.42E-18	8.08E-16	1.250527333
cg15679651	0.389537927	78.73875144	9.33E-18	8.87E-16	-1.525085302
cg08996748	0.1958679	78.66001943	9.66E-18	9.10E-16	-1.530814675
cg13678049	0.532086074	78.25233368	1.16E-17	1.07E-15	-1.301435801
cg10709021	0.237638433	78.21205165	1.18E-17	1.08E-15	-1.505615402
cg24831427	0.496528318	78.02654272	1.28E-17	1.15E-15	-1.387434783
cg17349199	0.729018219	77.88208985	1.37E-17	1.22E-15	-1.284291735
cg23679724	0.274808876	77.41578264	1.68E-17	1.47E-15	1.591565513
cg00016968	0.255193679	77.27724243	1.79E-17	1.53E-15	1.34850417
cg21096399	0.42295765	76.8992084	2.12E-17	1.79E-15	-1.363130475
cg18149919	0.676605249	76.83418555	2.18E-17	1.83E-15	-1.237477704
cg03699904	0.261569499	76.65731962	2.36E-17	1.96E-15	-1.508778806
cg26527984	0.40119013776.58964021		2.43E-17	2.01E-15	1.208480432

cg13277939	0.279538674	76.0214803	3.13E-17	2.52E-15	1.666991185
cg09205751	0.744302291	75.92849113	3.26E-17	2.61E-15	-1.251615597
cg12770741	0.756949118	74.97444655	4.99E-17	3.90E-15	-1.30787753
cg05094216	0.399190606	74.61623106	5.86E-17	4.54E-15	1.317173153
cg27416067	0.079643878	74.5943747	5.92E-17	4.55E-15	-1.594875202
cg22844623	0.609095312	74.56002784	6.01E-17	4.59E-15	1.237319059
cg00461841	0.43834526	74.52744073	6.10E-17	4.62E-15	1.238027662
cg25549459	0.150200106	73.43102523	9.97E-17	7.28E-15	-1.63280551
cg09134726	0.538077795	73.42982057	9.97E-17	7.28E-15	-1.351178019
cg07586911	0.798990525	73.06554989	1.17E-16	8.51E-15	-1.25065436
cg20624391	0.344852746	72.94925937	1.24E-16	8.90E-15	-1.380396951
cg07773116	0.424221246	72.70006476	1.38E-16	9.82E-15	-1.426974697
cg05293216	0.567975262	72.19878976	1.73E-16	1.20E-14	-1.380571195
cg20648149	0.60762707	72.03726946	1.86E-16	1.27E-14	-1.337295749

cg01693350	0.201542321	71.8887335	1.99E-16	1.34E-14	1.98065577
cg04513422	0.227758741	71.8561528	2.02E-16	1.35E-14	-1.471536869
cg15284635	0.611952867	71.83303164	2.04E-16	1.35E-14	-1.272350233
cg05868799	0.408603907	71.68727498	2.18E-16	1.44E-14	-1.485229279
cg24621042	0.235330823	71.33624577	2.55E-16	1.66E-14	1.536709505
cg25201363	0.517747269	71.06781295	2.88E-16	1.84E-14	-1.256374847
cg22424746	0.324701582	70.9102345	3.09E-16	1.96E-14	1.487807188
cg15937958	0.370406752	70.76412869	3.30E-16	2.08E-14	1.441128871
cg19067730	0.16508432	70.52921268	3.67E-16	2.28E-14	-1.630336363
cg09143663	0.506514274	70.4706831	3.77E-16	2.33E-14	-1.33649504
cg06142324	0.66437547	70.17330603	4.31E-16	2.65E-14	-1.294456578
cg05445326	0.669978763	69.96239352	4.74E-16	2.88E-14	-1.355814248
cg21745164	0.635372183	69.93071353	4.81E-16	2.89E-14	-1.291452619
cg03513363	0.195172106	69.61146351	5.56E-16	3.29E-14	-1.442265745
cg18758482	0.34839222	69.39769659	6.12E-16	3.61E-14	1.215518491
cg21275690	0.208517246	69.36087118	6.22E-16	3.65E-14	1.489912397

cg15479752	0.851757922	69.32330825	6.33E-16	3.69E-14	-1.2242205
cg10612997	0.512562848	69.07721252	7.07E-16	4.10E-14	1.356016134
cg06469542	0.399855072	69.01375777	7.28E-16	4.19E-14	-1.333764047
cg18396533	0.257231547	68.91441247	7.61E-16	4.36E-14	-1.415451432
cg13689073	0.279721053	68.85404533	7.82E-16	4.45E-14	1.577648342
cg06154570	0.438730076	68.3759621	9.71E-16	5.47E-14	-1.34794218
cg07156669	0.344981022	68.00825615	1.15E-15	6.38E-14	-1.397125532
cg20790056	0.352142407	67.80166341	1.26E-15	6.97E-14	1.300148535
cg00462994	0.255829399	67.46672884	1.47E-15	7.98E-14	-1.602098396
cg04590978	0.160063157	67.34013144	1.55E-15	8.36E-14	-1.577176991
cg22335340	0.254483383	67.27096095	1.60E-15	8.58E-14	1.51092684
cg17259265	0.4435474	66.98963281	1.82E-15	9.66E-14	1.366311474
cg18738906	0.253424265	66.98754503	1.82E-15	9.66E-14	1.560180746

cg01892689 0.26893114466.71220171 2.06E-15 1.09E-13 -1.366632815

cg24080529 0.454791962 66.6576767 2.12E-15 1.11E-13 -1.296158441

cg17714030 0.784465155 66.61703264 2.15E-15 1.12E-13 -

1.252724928

cg15160445 0.46525216 66.593061132.18E-15 1.13E-13 1.266511661

cg03599338 0.365597565 66.58691523 2.18E-15 1.13E-13

1.261340013

cg25195673 0.325948733 66.26935653 2.52E-15 1.29E-13 -

1.360120602

cg04711324 0.82145325 66.110701842.71E-15 1.38E-13 -1.274332175

cg24127989 0.159314622 66.00254013 2.85E-15 1.44E-13 -

1.484978962

cg12955127 0.25602372 65.94801964 2.92E-15 1.47E-13 1.645099775

cg23239444 0.40056787 65.60674934 3.41E-15 1.71E-13 -1.409308975

cg11277230 0.137216273 65.54407207 3.51E-15 1.75E-13 -

1.532360019

cg25683185 0.614418463 65.32366074 3.88E-15 1.90E-13 -

1.399682698

cg18429742 0.397225154 65.31960217 3.88E-15 1.90E-13 -

1.447892259

cg04747322 0.275996124 65.25064814 4.01E-15 1.95E-13 -

1.548553847

cg09276363	0.553787239	64.76969509	4.99E-15	2.39E-13	-1.26536154
cg02170525	0.086369414	64.73874989	5.06E-15	2.40E-13	-1.490909254
cg23555120	0.605631516	64.66300288	5.24E-15	2.48E-13	-1.420384388
cg18401406	0.856331953	64.52932522	5.57E-15	2.62E-13	-1.200232877
cg22233974	0.67527574	64.19959192	6.47E-15	3.02E-13	-1.239119434
cg11590700	0.076228971	64.13398959	6.66E-15	3.09E-13	1.326042196
cg01356829	0.631574767	64.06774365	6.87E-15	3.17E-13	-1.288668219
cg04457481	0.42239001	63.92028979	7.35E-15	3.32E-13	1.292447156
cg19317715	0.489566712	63.72939561	8.02E-15	3.61E-13	1.295495019
cg06100324	0.305078467	63.54832983	8.71E-15	3.87E-13	1.345337209
cg21615663	0.358363605	63.54715415	8.71E-15	3.87E-13	1.32505834
cg19279346	0.542352736	63.18260444	1.03E-14	4.51E-13	-1.315824576
cg06048973	0.71813488	62.5845636	1.35E-14	5.82E-13	-1.213012629

cg04001668	0.350771734	62.1153235	1.68E-14	7.09E-13	1.358672907
cg27625732	0.579271835	62.08835454	1.70E-14	7.15E-13	-1.222445763
cg09462826	0.083596347	62.01910999	1.75E-14	7.35E-13	-1.566759318
cg17518825	0.70912801	61.41805757	2.31E-14	9.48E-13	-1.244656173
cg19433435	0.665766771	61.404525	2.32E-14	9.50E-13	-1.245966543
cg14417329	0.440760137	61.38844347	2.34E-14	9.53E-13	-1.364102569
cg06874144	0.730275017	61.24941372	2.49E-14	1.01E-12	-1.245147401
cg04089739	0.503531568	61.15983125	2.60E-14	1.05E-12	1.316701607
cg15988792	0.218306803	61.07064769	2.70E-14	1.09E-12	-1.504164301
cg14011639	0.11587142	61.04237387	2.74E-14	1.10E-12	2.862522695
cg05600717	0.129972969	60.96068977	2.84E-14	1.14E-12	-1.55626365
cg13042487	0.047601327	60.9265891	2.89E-14	1.15E-12	-1.553918382
cg05768141	0.095042935	60.55982249	3.42E-14	1.34E-12	-1.5339791
cg18793806	0.370511955	60.38997443	3.69E-14	1.44E-12	-1.344061098
cg23502772	0.175111856	60.20546872	4.02E-14	1.55E-12	-1.554593327

cg01777397	0.186709343	60.18822705	4.05E-14	1.56E-12	-1.498960158
cg14170423	0.347881919	60.17149158	4.08E-14	1.56E-12	-1.329828575
cg15988232	0.857189003	60.07770243	4.26E-14	1.62E-12	-1.206150805
cg08260891	0.158609816	60.04036971	4.34E-14	1.64E-12	-1.598187357
cg01420388	0.704124466	60.02692984	4.36E-14	1.65E-12	-1.265897993
cg15901783	0.518242172	59.96361884	4.49E-14	1.69E-12	-1.293753513
cg05125838	0.422849552	59.91529483	4.59E-14	1.71E-12	1.311536335
cg02335804	0.156926803	59.74720912	4.96E-14	1.84E-12	2.561572331
cg20083676	0.41430494	59.69047705	5.09E-14	1.88E-12	-1.427381022
cg14925024	0.539516209	59.62055067	5.26E-14	1.93E-12	-1.376036629
cg10631471	0.177168177	59.58039573	5.36E-14	1.96E-12	1.518170205
cg12058490	0.521537263	59.57928212	5.36E-14	1.96E-12	

					1.276221977
cg03693099	0.37397748	59.38759785	5.85E-14	2.13E-12	1.354085328
cg18967533	0.245994858	59.30812524	6.07E-14	2.19E-12	1.446820461
cg19591881	0.090757949	58.83776263	7.54E-14	2.69E-12	-1.56684777
cg16907566	0.704096401	58.79510799	7.69E-14	2.74E-12	-1.31916601
cg02831604	0.206559088	58.51773064	8.74E-14	3.10E-12	-1.508089309
cg22289837	0.640330994	58.50348357	8.79E-14	3.11E-12	-1.283245589
cg07785936	0.226397542	58.33776033	9.49E-14	3.32E-12	1.299930478
cg13144783	0.532622387	58.11014382	1.05E-13	3.65E-12	1.230626133
cg06607866	0.065068582	58.03508523	1.09E-13	3.75E-12	-1.564014291
cg19740375	0.503235412	57.88476716	1.17E-13	4.01E-12	-1.319098327
cg05406101	0.809360709	57.80740247	1.21E-13	4.14E-12	-1.235571153
cg13701109	0.102227972	57.79368371	1.22E-13	4.15E-12	-

1.562162927

cg18168989 0.924456986 57.75374826 1.24E-13 4.20E-12 -

1.214207753

cg17177660 0.467799192 57.693862111.28E-13 4.27E-12 -1.284375955

cg00573606 0.426671043 57.590117511.34E-13 4.47E-12 -1.388897494

cg03552103 0.385273244 57.43364135 1.44E-13 4.77E-12 -

1.421721114

cg07408740 0.343987289 57.35509341 1.49E-13 4.93E-12 -

1.343123924

cg10362475 0.855634756 57.33744028 1.51E-13 4.96E-12 -

1.249451634

cg12788467 0.236978373 57.17256467 1.62E-13 5.32E-12 1.97602632

cg20367961 0.316703727 57.15563999 1.64E-13 5.32E-12

1.296965106

cg13641903 0.2562363 57.13325102 1.65E-13 5.36E-12 1.583479756

cg03952109 0.39661142257.06343769 1.71E-13 5.50E-12 1.239540577

cg16393207 0.559104434 57.0232267 1.74E-13 5.57E-12 -1.311194587

cg03202897 0.497578667 57.001112931.76E-13 5.61E-12 1.215782302

cg03870261 0.81218134 56.97464281 1.78E-13 5.66E-12 -1.207363839

cg15534366 0.731393724 56.63139527 2.09E-13 6.53E-12 -

1.259749077

cg21623671 0.108918231 56.41859776 2.30E-13 7.16E-12 -

1.536334197

cg08952029 0.360025693 56.33993545 2.39E-13 7.38E-12 -

1.378135239

cg03213216 0.083974528 56.26292224 2.47E-13 7.62E-12 -

1.562900731

cg00221494 0.22884117455.96847183 2.83E-13 8.65E-12 -1.482705752

cg10225525 0.57337611855.3555677 3.76E-13 1.14E-11 1.216926756

cg09458237 0.756321902 55.20681032 4.03E-13 1.21E-11 -

1.214090771

cg26847866 0.149741738 55.18340883 4.08E-13 1.22E-11

1.592272448

cg14719352 0.324314522 55.15320339 4.13E-13 1.24E-11

1.295251885

cg09486093 0.196468101 54.89222556 4.67E-13 1.38E-11 -

1.483236263

cg08221207 0.079656516 54.77180361 4.93E-13 1.45E-11 -

1.502198398

cg11935638 0.12934408 54.57694798 5.40E-13 1.58E-11 -1.436619031

cg13523557 0.20891994 54.45291628 5.72E-13 1.67E-11 -1.511383555

cg01566404 0.536998047 54.072085 6.83E-13 1.95E-11 -1.341509687

cg10523671 0.48355118253.99275883 7.08E-13 2.02E-11 1.395640263

cg16869108 0.435668093 53.93201448 7.29E-13 2.07E-11 -

1.409116957

cg23640701 0.643152418 53.911085247.36E-13 2.08E-11 -1.204907019

cg21459867 0.375817969 53.82746952 7.65E-13 2.16E-11 1.25875481

cg18137704 0.120999592 53.72073957 8.04E-13 2.25E-11 -

1.457044909

cg01269795 0.182226808 53.68924431 8.16E-13 2.27E-11 1.4828571

cg25040733 0.101895 53.50016958 8.91E-13 2.46E-11 1.285227768

cg05023691 0.52346056 53.28921333 9.82E-13 2.70E-11 1.216676498

cg16501028 0.249306749 53.17025067 1.04E-12 2.84E-11

1.678253546

cg27285720 0.212403154 52.87551287 1.19E-12 3.24E-11

1.909408368

cg03032025 0.510641026 52.86205093 1.20E-12 3.25E-11

1.268215139

cg05564266 0.698187071 52.59805972 1.36E-12 3.64E-11 -

1.269577937

cg18081258 0.33684112652.50126379 1.42E-12 3.79E-11 -1.371579255

cg20939319 0.462336054 52.13742152 1.68E-12 4.45E-11

1.248894118

cg02250594 0.253564813 51.97205286 1.81E-12 4.78E-11 -

1.539798603

cg10954182 0.11481196 51.96797667 1.82E-12 4.78E-11 -1.410838969

cg07027513	0.355593526	51.62607691	2.13E-12	5.54E-11	-1.459934112
cg24670151	0.308412819	51.54901685	2.21E-12	5.72E-11	1.300778767
cg19420968	0.76180344	51.52644731	2.23E-12	5.77E-11	-1.200922912
cg15544036	0.277050275	51.46048679	2.30E-12	5.94E-11	1.305797535
cg04230060	0.641039582	51.3300494	2.45E-12	6.29E-11	-1.219399982
cg08088989	0.533647091	51.32421596	2.45E-12	6.29E-11	-1.304771765
cg15329642	0.7102647	51.30894312	2.47E-12	6.32E-11	-1.205055469
cg24087944	0.443858036	51.25292661	2.54E-12	6.47E-11	-1.255565287
cg17655614	0.156155879	50.90455964	2.99E-12	7.52E-11	1.296956224
cg01718139	0.404436012	50.82978578	3.09E-12	7.77E-11	-1.353256454
cg21301440	0.358865479	50.80014043	3.14E-12	7.84E-11	-1.313809485
cg26203861	0.423945902	50.73630313	3.23E-12	8.02E-11	-1.28081241
cg09259772	0.175448742	50.65522109	3.36E-12	8.27E-11	-

1.408270588

cg15446391 0.172658871 50.65485017 3.36E-12 8.27E-11

1.783486776

cg01888601 0.431463593 50.52373965 3.57E-12 8.73E-11 -

1.331604829

cg02930996 0.349075591 50.3834245 3.81E-12 9.25E-11 -1.446674362

cg12629244 0.614447636 50.34780288 3.87E-12 9.38E-11 -

1.312777585

cg11695358 0.481284757 50.33335125 3.90E-12 9.42E-11 -

1.257829978

cg20001829 0.135870568 50.29483585 3.97E-12 9.55E-11 -

1.528937024

cg06821120 0.369084544 50.2492728 4.06E-12 9.73E-11 -1.373803605

cg04563996 0.362828709 50.1294458 4.29E-12 1.02E-10 1.326320224

cg20340596 0.623395773 49.96781268 4.63E-12 1.10E-10 -

1.289579245

cg23851011 0.357444291 49.84831005 4.89E-12 1.15E-10

1.521220816

cg26453670 0.177613597 49.79320397 5.02E-12 1.18E-10 -

1.51568183

cg00135056 0.069319449 49.77259077 5.07E-12 1.19E-10

1.597057977

cg11204562	0.219668861	49.72124375	5.19E-12	1.21E-10	1.594977031
cg20324165	0.136193571	49.68135198	5.29E-12	1.23E-10	1.447676548
cg12554476	0.679636975	49.43307282	5.95E-12	1.37E-10	-1.26051843
cg20955688	0.287446689	49.3763559	6.11E-12	1.40E-10	1.527141705
cg24833277	0.21433071	49.08931057	6.99E-12	1.59E-10	1.437766604
cg02275294	0.169500115	49.03243079	7.17E-12	1.63E-10	-1.475695462
cg01414934	0.10977681	49.01393144	7.24E-12	1.64E-10	-1.457044972
cg08145177	0.235244709	48.95270599	7.45E-12	1.68E-10	-1.45488029
cg15241708	0.644009281	48.92430045	7.55E-12	1.70E-10	-1.288938866
cg12855988	0.240200984	48.67446542	8.49E-12	1.90E-10	1.365936829
cg03381111	0.462818569	48.62218773	8.70E-12	1.94E-10	1.310569609
cg02947253	0.654421933	48.50286126	9.20E-12	2.04E-10	-1.232967578
cg25151806	0.846424664	48.4708821	9.34E-12	2.06E-10	-1.213952437
cg01033938	0.560573865	48.45104053	9.43E-12	2.08E-10	-

1.298376435

cg01129459 0.20549422 48.29930479 1.01E-11 2.21E-10 -1.469284534

cg06781209 0.347232652 48.22812915 1.05E-11 2.28E-10 -

1.438961423

cg15821095 0.675330155 48.16410047 1.08E-11 2.34E-10 -

1.244884712

cg08268099 0.548535665 48.05697737 1.13E-11 2.45E-10 -

1.314341176

cg21614638 0.229575029 48.0531925 1.14E-11 2.45E-10 1.727777277

cg00489401 0.561365035 48.039776 1.14E-11 2.45E-10 -1.322000835

cg00186701 0.502106458 48.03880471 1.14E-11 2.45E-10 -

1.411612501

cg17191178 0.12831173348.0226179 1.15E-11 2.47E-10 -1.619899998

cg07799947 0.175916881 48.00754856 1.16E-11 2.47E-10

1.296737242

cg18099844 0.801730484 47.67903301 1.35E-11 2.87E-10 -

1.212437514

cg01573562 0.047862935 47.62192236 1.39E-11 2.94E-10 -

1.38251708

cg06488678 0.594191828 47.60045408 1.41E-11 2.96E-10 -

1.258133679

cg11296937 0.225967539 47.511815271.47E-11 3.08E-10 1.292181324

cg04032226	0.543687019	47.45582701	1.50E-11	3.14E-10	-1.397892752
cg01281904	0.503967177	47.3662173	1.57E-11	3.27E-10	-1.318988951
cg24459209	0.492211574	47.22682694	1.68E-11	3.48E-10	-1.314429219
cg07297178	0.76044224	47.19021529	1.71E-11	3.54E-10	-1.229907846
cg00231644	0.441287968	47.14641215	1.74E-11	3.60E-10	-1.28125963
cg06851207	0.27836631	47.07188742	1.80E-11	3.72E-10	-1.358493127
cg07294734	0.274528454	46.97890394	1.88E-11	3.87E-10	1.338769357
cg12554573	0.283653041	46.85174943	2.00E-11	4.08E-10	1.328830333
cg03152385	0.203624156	46.72155857	2.13E-11	4.33E-10	-1.335570237
cg14566624	0.139741346	46.6929854	2.16E-11	4.36E-10	-1.56008064
cg04451770	0.658920804	46.6241114	2.23E-11	4.49E-10	-1.328146335
cg17612991	0.311890701	46.5831513	2.27E-11	4.56E-10	1.559868476
cg26683023	0.729476071	46.57307654	2.28E-11	4.58E-10	-1.240210229
cg18170080	0.09613927	46.44017279	2.43E-11	4.84E-10	-1.446114256
cg12971694	0.557383186	46.36569226	2.51E-11	4.99E-10	-1.223602033

cg20437604	0.229963302	46.12835544	2.81E-11	5.56E-10	1.390502198
cg15512851	0.119366911	46.11092784	2.84E-11	5.59E-10	2.005106915
cg00116838	0.087590439	46.02540239	2.95E-11	5.81E-10	-1.443016467
cg19005210	0.572579145	45.99309968	3.00E-11	5.87E-10	-1.210204478
cg21111471	0.6253407	45.98730663	3.01E-11	5.87E-10	-1.274652762
cg21129531	0.824273658	45.9095532	3.12E-11	6.07E-10	-1.201465482
cg18952647	0.446213379	45.83095658	3.24E-11	6.27E-10	-1.428786321
cg07185695	0.224905607	45.81134135	3.27E-11	6.32E-10	-1.49879912
cg27494383	0.142173901	45.78388226	3.31E-11	6.38E-10	-1.602907589
cg10885338	0.453327854	45.72728082	3.40E-11	6.54E-10	-1.330518261
cg06096336	0.148910621	45.71779084	3.41E-11	6.54E-10	1.836631189
cg19186356	0.15319238	45.65243393	3.52E-11	6.73E-10	-1.415331591
cg09945801	0.422358139	45.58774895	3.63E-11	6.93E-10	-1.343172031
cg16408970	0.304737378	45.55050691	3.70E-11	7.04E-10	-

1.398281258

cg15741583 0.479162269 45.49864205 3.79E-11 7.20E-10

1.200379053

cg26251865 0.450553463 45.43072509 3.91E-11 7.42E-10 -

1.278360175

cg27462398 0.684868879 45.38770606 3.99E-11 7.54E-10 -

1.258123577

cg05556202 0.736153327 45.34638292 4.07E-11 7.66E-10 -

1.254372972

cg07730329 0.463379344 45.13301473 4.50E-11 8.43E-10

1.389061882

cg10149836 0.221861228 45.03472548 4.72E-11 8.81E-10 -

1.367890676

cg07705908 0.186665347 44.94498393 4.92E-11 9.15E-10

1.652414726

cg00174901 0.254656472 44.86827965 5.10E-11 9.41E-10 -

1.353937131

cg17692403 0.22401474 44.8380865 5.18E-11 9.51E-10 -1.41086373

cg07950803 0.559769883 44.52245108 6.01E-11 1.09E-09 -

1.320271258

cg16173067 0.501780635 44.439118636.25E-11 1.13E-09 1.246079935

cg10693071 0.35463777 44.26748291 6.78E-11 1.21E-09 -1.376233295

cg02166532	0.144721425	44.23683364	6.88E-11	1.22E-09	1.34556629
cg16615211	0.398073307	44.21708628	6.94E-11	1.23E-09	-1.381379833
cg18732541	0.192584216	44.19494342	7.02E-11	1.24E-09	-1.457251753
cg21057494	0.191685917	44.18089011	7.06E-11	1.25E-09	1.50385601
cg14859417	0.226755444	44.05755082	7.49E-11	1.32E-09	1.393986827
cg11647681	0.52973396	44.03878711	7.56E-11	1.33E-09	1.288001345
cg23900225	0.474719957	43.97309456	7.79E-11	1.36E-09	-1.235214733
cg14597908	0.376469807	43.89950163	8.07E-11	1.40E-09	1.264915284
cg20125091	0.377637185	43.85907685	8.23E-11	1.42E-09	-1.333909781
cg18414381	0.085812128	43.82551596	8.36E-11	1.44E-09	2.282127455
cg19026260	0.12011539	43.79329414	8.49E-11	1.46E-09	-1.475234716
cg04863713	0.048799069	43.77364478	8.57E-11	1.47E-09	-1.401913278
cg05421688	0.155650417	43.71832937	8.80E-11	1.51E-09	-1.535424423

cg03826976	0.181028063	43.69083699	8.91E-11	1.52E-09	-1.527327096
cg11877382	0.141813273	43.6418436	9.12E-11	1.56E-09	-1.405738751
cg04473302	0.094671626	43.56922437	9.44E-11	1.61E-09	-1.620553483
cg21019522	0.29641908	43.43292875	1.01E-10	1.71E-09	1.409460825
cg25722142	0.123572442	43.34709664	1.05E-10	1.78E-09	1.266854527
cg24603941	0.556185894	43.33865355	1.05E-10	1.78E-09	1.24179084
cg27016307	0.451385816	43.28660598	1.08E-10	1.82E-09	-1.28090431
cg17982102	0.323785528	43.27666166	1.08E-10	1.82E-09	-1.430398555
cg16386080	0.113265286	43.19038237	1.13E-10	1.89E-09	-1.485301953
cg16992787	0.409051769	43.11823776	1.17E-10	1.95E-09	1.288388302
cg06305609	0.517094524	43.01502401	1.23E-10	2.05E-09	-1.291115201
cg00448720	0.644528057	42.99409574	1.24E-10	2.07E-09	-1.228076759
cg19862344	0.624975891	42.85140886	1.33E-10	2.19E-09	-1.241748847
cg21092324	0.485263998	42.81802854	1.35E-10	2.22E-09	

1.203745247

cg25268451 0.45107076 42.72957993 1.41E-10 2.32E-09 1.234546121

cg16862361 0.248993551 42.72156807 1.41E-10 2.32E-09

1.570814799

cg15201877 0.183672809 42.65528272 1.46E-10 2.38E-09 -

1.459589073

cg22876908 0.122355074 42.56785015 1.52E-10 2.48E-09

1.409652753

cg11277126 0.11984580442.53479389 1.54E-10 2.51E-09 -1.470560797

cg13064571 0.507579164 42.36229238 1.67E-10 2.71E-09 1.24575757

cg09467501 0.377167881 42.0781117 1.92E-10 3.08E-09 -1.474415459

cg15312298 0.194165925 42.03539318 1.96E-10 3.13E-09

1.258195006

cg01485645 0.086789712 41.97495312 2.01E-10 3.21E-09

1.302841165

cg17675150 0.712723095 41.97438125 2.01E-10 3.21E-09 -

1.228438986

cg08810582 0.08326616 41.95430308 2.03E-10 3.23E-09 -1.401755059

cg18495563 0.208976134 41.93622644 2.05E-10 3.25E-09 -

1.40568996

cg21032583 0.443442558 41.81888399 2.17E-10 3.43E-09 -

1.368034392

cg18055007 0.27964311541.67460689 2.32E-10 3.64E-09 -1.518542687

cg18437633 0.17371108241.64552603 2.36E-10 3.68E-09 1.586075718

cg11719297 0.144982786 41.61400181 2.39E-10 3.73E-09

1.338579846

cg07378350 0.446979913 41.58754861 2.42E-10 3.77E-09 -

1.346260083

cg06339657 0.577589984 41.57465665 2.44E-10 3.78E-09 -

1.221135798

cg20103550 0.509147224 41.43538796 2.60E-10 4.04E-09

1.209567452

cg27176536 0.11644750241.293311422.79E-10 4.29E-09 1.271083916

cg20584011 0.546635344 41.29281383 2.79E-10 4.29E-09 -

1.26722255

cg25484904 0.281560183 41.2795637 2.80E-10 4.31E-09 -1.341770616

cg19525717 0.096800713 41.244686112.85E-10 4.36E-09 -1.381318197

cg21513385 0.207087802 41.2237815 2.88E-10 4.40E-09 -1.35474361

cg02593766 0.492566846 41.13493439 3.00E-10 4.57E-09

1.238224082

cg07115558 0.093303081 41.131141013.01E-10 4.57E-09 1.263541317

cg04456238 0.17304115940.85269475 3.44E-10 5.19E-09 1.892497497

cg06310844 0.23860035 40.73547765 3.64E-10 5.46E-09 1.265617456

cg15127733 0.672459719 40.72998492 3.65E-10 5.47E-09 -

1.224015184

cg19255783 0.4381784 40.52962179 4.01E-10 5.98E-09 1.205555968

cg22461018 0.547306102 40.52494209 4.02E-10 5.98E-09

1.239083998

cg09276451 0.240403813 40.41526344 4.24E-10 6.28E-09

1.316392939

cg07965823 0.11999675540.39233885 4.28E-10 6.34E-09 -1.597717933

cg01993576 0.24026734 40.35090544 4.37E-10 6.44E-09 -1.355543259

cg20225915 0.181964743 40.22896045 4.63E-10 6.80E-09

1.278347407

cg17704839 0.313735676 40.22715819 4.63E-10 6.80E-09 1.39031513

cg13577076 0.247286495 40.02636254 5.10E-10 7.45E-09 -

1.494810248

cg16853982 0.487515389 39.89231798 5.44E-10 7.90E-09 -

1.355522279

cg05778847 0.198221294 39.84271812 5.57E-10 8.06E-09 -1.3721489

cg18854666 0.752638218 39.82466791 5.62E-10 8.12E-09 -

1.201726505

cg00311768 0.308492157 39.781138095.74E-10 8.26E-09 1.37525169

cg25259754 0.499449236 39.69091334 5.99E-10 8.56E-09 -

1.345122184

cg19514469 0.283758379 39.67170943 6.04E-10 8.61E-09

1.322830505

cg19224278 0.136564966 39.50419895 6.55E-10 9.27E-09 -

1.432564796

cg22421699 0.108431668 39.42980305 6.79E-10 9.58E-09

1.241287616

cg19535609 0.124552066 39.30805158 7.19E-10 1.01E-08 -

1.323287648

cg09298623 0.268682964 39.27526907 7.31E-10 1.03E-08 -

1.235956229

cg22396755 0.492718855 39.18489201 7.63E-10 1.07E-08 -

1.371959896

cg27478659 0.299294758 39.16624744 7.70E-10 1.08E-08

1.328839884

cg04925864 0.071690374 39.12154839 7.87E-10 1.10E-08 -

1.469150434

cg11536940 0.392795692 39.118154527.88E-10 1.10E-08 -1.413020581

cg08575537 0.544021489 39.07181531 8.05E-10 1.12E-08 -

1.310629287

cg04369341 0.334510555 39.04732072 8.15E-10 1.13E-08 -

1.384386294

cg09191232 0.477919016 39.02659086 8.23E-10 1.14E-08 -1.3182713

cg13471990 0.48763315 39.0143925 8.28E-10 1.15E-08 -1.286824158

cg19418958	0.350200931	38.85924777	8.92E-10	1.23E-08	1.297729071
cg11154879	0.409381453	38.85191626	8.95E-10	1.23E-08	1.205338056
cg13631259	0.085051501	38.81099338	9.13E-10	1.25E-08	1.620941354
cg03977657	0.229371887	38.78820096	9.23E-10	1.27E-08	1.28896572
cg26796190	0.336462578	38.78533842	9.24E-10	1.27E-08	-1.507268697
cg27634151	0.093275372	38.78214917	9.25E-10	1.27E-08	-1.584223692
cg25027167	0.114205208	38.69801405	9.64E-10	1.31E-08	-1.440614708
cg00850538	0.597896585	38.63682823	9.92E-10	1.35E-08	-1.233751172
cg22780475	0.268980159	38.62699565	9.97E-10	1.35E-08	1.380127107
cg22377998	0.030518616	38.48862614	1.07E-09	1.43E-08	-1.433622339
cg20019546	0.310098575	38.35593687	1.14E-09	1.52E-08	-1.396874749
cg22740783	0.083656453	38.34525261	1.14E-09	1.53E-08	-1.434614493

cg24800810	0.106221649	38.2700133	1.18E-09	1.58E-08	-1.542997551
cg04001333	0.297124322	38.21821828	1.21E-09	1.62E-08	-1.39599162
cg05222924	0.151214716	38.21653462	1.21E-09	1.62E-08	2.17443824
cg07408456	0.161840279	38.00725865	1.34E-09	1.77E-08	1.312504503
cg00208967	0.117977892	38.00068745	1.35E-09	1.77E-08	-1.451537641
cg20289911	0.353577965	37.98928292	1.35E-09	1.78E-08	1.28287265
cg07548313	0.741654896	37.90219573	1.41E-09	1.85E-08	-1.231855458
cg21073927	0.251436658	37.89157138	1.42E-09	1.86E-08	-1.494738026
cg16986720	0.62654222	37.85033058	1.45E-09	1.89E-08	-1.250366079
cg15783027	0.086015551	37.79798443	1.48E-09	1.93E-08	-1.540799692
cg18705301	0.467194677	37.78157611	1.50E-09	1.94E-08	-1.285347176
cg18997129	0.143216106	37.75180265	1.52E-09	1.97E-08	1.434015837
cg09328024	0.162677772	37.64254883	1.60E-09	2.07E-08	-1.429824054
cg03389133	0.305927138	37.62531475	1.61E-09	2.08E-08	1.250678929

cg25974617	0.30149294	37.61435844	1.62E-09	2.09E-08	-1.292995841
cg26116551	0.448465413	37.61190147	1.62E-09	2.09E-08	-1.30832947
cg00090147	0.271237353	37.54980129	1.67E-09	2.15E-08	-1.476005299
cg06734812	0.265063941	37.44889562	1.76E-09	2.25E-08	1.272628599
cg02789485	0.392815399	37.44886691	1.76E-09	2.25E-08	1.338622982
cg05292376	0.35205602	37.4386202	1.76E-09	2.26E-08	1.328482875
cg26563737	0.317115409	37.38531269	1.81E-09	2.31E-08	1.231901062
cg03991512	0.240072411	37.1966374	1.98E-09	2.51E-08	1.540982275
cg15415507	0.11867044	37.14453211	2.03E-09	2.57E-08	1.222744748
cg05538432	0.230915296	37.10567635	2.07E-09	2.61E-08	1.518421841
cg22909609	0.652163293	37.0821035	2.09E-09	2.64E-08	-1.245575243
cg07123069	0.262541356	37.08112583	2.09E-09	2.64E-08	-1.458302801
cg22215728	0.098430307	37.07574222	2.10E-09	2.64E-08	-1.518667423
cg16944093	0.17456627	37.04994844	2.13E-09	2.67E-08	1.310428227
cg21624282	0.588292439	36.93555291	2.25E-09	2.81E-08	-1.232166695
cg15652212	0.188693892	36.90070212	2.28E-09	2.85E-08	

1.427150016

cg06690548 0.237031017 36.7769352 2.43E-09 3.01E-08 1.557157988

cg11438428 0.239062728 36.71957408 2.49E-09 3.09E-08 -

1.490870714

cg24364574 0.216677495 36.57969727 2.67E-09 3.30E-08 -

1.347638574

cg06921282 0.148962032 36.53214574 2.73E-09 3.36E-08 -

1.393211328

cg12781568 0.104342292 36.53105462 2.73E-09 3.36E-08

2.261191574

cg24977027 0.690820921 36.51929345 2.75E-09 3.37E-08 -

1.227950073

cg06595693 0.320718424 36.49897721 2.77E-09 3.40E-08 -

1.337404691

cg09949775 0.292330694 36.4833464 2.79E-09 3.42E-08 -1.418153329

cg24505341 0.53016979 36.3447141 2.99E-09 3.64E-08 1.228742659

cg20973210 0.41441463 36.31789995 3.03E-09 3.68E-08 -1.360180191

cg03752885 0.239652213 36.28780844 3.07E-09 3.71E-08

1.509925301

cg22262140 0.527164373 36.26967296 3.10E-09 3.74E-08 -

1.24027484

cg03625911 0.156156313 36.22720488 3.16E-09 3.81E-08

					1.333018114
cg26525091	0.268581014	36.18167769	3.23E-09	3.89E-08	-1.358943329
cg07327468	0.271207385	35.88259156	3.73E-09	4.46E-08	-1.395702263
cg27167601	0.066185298	35.86074618	3.77E-09	4.50E-08	-1.495450396
cg01656853	0.126294308	35.85752419	3.78E-09	4.50E-08	1.346400178
cg16377872	0.621657309	35.77951423	3.92E-09	4.67E-08	-1.247379189
cg09997082	0.520526245	35.72112563	4.03E-09	4.78E-08	-1.206438325
cg16028753	0.178704188	35.69521436	4.09E-09	4.84E-08	-1.374616101
cg25782229	0.201503521	35.60208437	4.27E-09	5.04E-08	1.762104535
cg18986273	0.074644372	35.47013046	4.55E-09	5.34E-08	1.326304004
cg16983159	0.101069086	35.41819434	4.67E-09	5.47E-08	2.146226732
cg04582938	0.394062397	35.40773297	4.69E-09	5.49E-08	-1.274670638

cg23873703	0.44243331	35.36097271	4.80E-09	5.61E-08	-1.316702586
cg11378484	0.111582748	35.30671484	4.93E-09	5.75E-08	1.637957842
cg02105856	0.314312219	35.11598108	5.40E-09	6.24E-08	1.391788768
cg02876062	0.706353333	35.07463335	5.51E-09	6.35E-08	-1.218531097
cg14851685	0.431108425	35.01770265	5.67E-09	6.51E-08	-1.282094115
cg11004890	0.623759279	34.91601637	5.95E-09	6.82E-08	-1.249669934
cg22774472	0.219930959	34.81739876	6.24E-09	7.15E-08	-1.448959834
cg05382123	0.113025512	34.78199664	6.35E-09	7.26E-08	-1.598562188
cg18139769	0.424408793	34.75050212	6.45E-09	7.35E-08	1.212640039
cg10525372	0.272565114	34.71591213	6.56E-09	7.46E-08	1.300605599
cg11566244	0.116802572	34.62598172	6.85E-09	7.78E-08	1.295222107
cg01484156	0.258498469	34.56345565	7.06E-09	8.00E-08	1.481827707
cg18540325	0.266020353	34.52708084	7.19E-09	8.14E-08	1.280494654
cg13573276	0.069915515	34.4795091	7.35E-09	8.31E-08	-1.617187608
cg02956093	0.085717389	34.47408735	7.37E-09	8.32E-08	1.301018651

cg20270599	0.21027789	34.46948164	7.39E-09	8.32E-08	-1.368168297
cg03642518	0.269771677	34.38548033	7.70E-09	8.62E-08	-1.391638033
cg11368643	0.381059985	34.38174082	7.71E-09	8.62E-08	1.451666581
cg10878307	0.33316924	34.23403326	8.28E-09	9.25E-08	1.316286388
cg12387247	0.389043422	34.16268348	8.57E-09	9.56E-08	-1.316924802
cg26644395	0.21237159	34.09805816	8.85E-09	9.82E-08	-1.377664602
cg21105318	0.194265586	34.03090009	9.14E-09	1.01E-07	1.471759771
cg12815142	0.565843598	33.94776489	9.51E-09	1.05E-07	-1.264734273
cg22892904	0.240778062	33.84257652	1.00E-08	1.10E-07	-1.320207719
cg09107315	0.477507517	33.75040299	1.05E-08	1.15E-07	-1.30682552
cg22748452	0.262504495	33.74917635	1.05E-08	1.15E-07	-1.27905733
cg25341653	0.384898249	33.73601768	1.05E-08	1.16E-07	1.290114699
cg21517055	0.286700522	33.71265363	1.07E-08	1.17E-07	-

1.440449778

cg17836145 0.454700451 33.70719545 1.07E-08 1.17E-07

1.279616393

cg03914397 0.522656213 33.62789412 1.11E-08 1.21E-07 -

1.263751099

cg09173897 0.49824466 33.56294533 1.15E-08 1.25E-07 -1.241052198

cg10670077 0.255256019 33.537116271.16E-08 1.27E-07 1.291554074

cg26385743 0.767967367 33.52753885 1.17E-08 1.27E-07 -

1.226287369

cg11809091 0.190683029 33.51685692 1.17E-08 1.27E-07

1.749837034

cg03973663 0.66863978 33.462112111.20E-08 1.30E-07 -1.230675202

cg15967525 0.159914074 33.40592802 1.24E-08 1.33E-07

1.551453648

cg12085660 0.064537656 33.15391559 1.40E-08 1.49E-07

1.277079021

cg19692710 0.267992636 33.15313937 1.40E-08 1.49E-07

1.446785054

cg00498305 0.454583739 33.14675083 1.40E-08 1.50E-07 -

1.289994388

cg01835489 0.207796103 33.127013 1.42E-08 1.51E-07 1.363307117

cg16616769 0.315185188 33.10010459 1.44E-08 1.52E-07

					1.472417432
cg17777592	0.304192451	33.08687719	1.44E-08	1.53E-07	1.362752625
cg18133957	0.424417973	33.0517702	1.47E-08	1.56E-07	-1.303437677
cg11136562	0.392862546	32.99036105	1.51E-08	1.60E-07	1.212586571
cg19601328	0.344083857	32.98265028	1.52E-08	1.60E-07	1.34511105
cg05924583	0.228286102	32.91873055	1.57E-08	1.65E-07	-1.414090862
cg04743872	0.166962945	32.88458542	1.59E-08	1.67E-07	-1.442882659
cg04586023	0.387420532	32.8494421	1.62E-08	1.69E-07	1.217397519
cg27159719	0.377467642	32.83968654	1.63E-08	1.70E-07	1.42189348
cg16376234	0.068259471	32.78457936	1.67E-08	1.74E-07	1.265753132
cg04637372	0.62695756	32.73887991	1.71E-08	1.78E-07	-1.245493548
cg15916628	0.150758286	32.73201983	1.72E-08	1.78E-07	1.613179955
cg05208878	0.215009619	32.7260423	1.72E-08	1.78E-07	1.28726826
cg17127823	0.141667797	32.66160282	1.78E-08	1.84E-07	-1.300190134
cg10044101	0.506666421	32.61157163	1.82E-08	1.88E-07	1.231694575

cg17786776	0.576313244	32.6105021	1.82E-08	1.88E-07	-1.223795254
cg04837071	0.138064292	32.58076691	1.85E-08	1.91E-07	1.396510166
cg11484576	0.363491847	32.48805083	1.93E-08	1.99E-07	1.312687883
cg18771300	0.676220916	32.39562923	2.02E-08	2.07E-07	-1.239435954
cg26929536	0.314367977	32.38878373	2.03E-08	2.08E-07	-1.392355989
cg13765785	0.086326066	32.3017239	2.12E-08	2.16E-07	1.240465712
cg13053608	0.453063304	32.20241115	2.22E-08	2.26E-07	1.20433387
cg20330472	0.0669154	32.180294	2.25E-08	2.28E-07	-1.458984562
cg24805239	0.043521603	32.13475896	2.30E-08	2.33E-07	-1.600655656
cg00893242	0.071921157	32.11085067	2.32E-08	2.35E-07	-1.521252151
cg17803965	0.36336741	32.05107897	2.39E-08	2.41E-07	1.343805525
cg02633817	0.196260854	32.04597792	2.40E-08	2.41E-07	1.546501363
cg23539753	0.043707694	32.01548812	2.43E-08	2.44E-07	2.947580959
cg25763788	0.233482965	31.97364246	2.48E-08	2.49E-07	1.563768465

cg01119135 0.203580484 31.97223726 2.49E-08 2.49E-07

1.538558889

cg15776355 0.152810018 31.87820072 2.60E-08 2.60E-07

1.569671295

cg10305797 0.587260308 31.80269253 2.70E-08 2.69E-07 -1.2228685

cg05647859 0.261467343 31.74966671 2.77E-08 2.75E-07 -

1.453366462

cg04431054 0.107287663 31.74283804 2.78E-08 2.76E-07 -

1.368794443

cg00756887 0.30154117831.73198569 2.79E-08 2.77E-07 1.264949501

cg24273512 0.085449951 31.703723112.83E-08 2.80E-07 -1.369780818

cg23950724 0.51158630331.64628274 2.91E-08 2.88E-07 -1.214657712

cg10757144 0.31962166 31.57327551 3.02E-08 2.98E-07 1.513541556

cg17279839 0.307966293 31.56341771 3.03E-08 2.99E-07 -

1.408856573

cg19853703 0.026294999 31.55625974 3.04E-08 2.99E-07 3.57910508

cg11300809 0.143204978 31.55464153 3.05E-08 2.99E-07

1.339661345

cg14371590 0.601690457 31.54585005 3.06E-08 3.00E-07 -

1.213083479

cg19917856 0.17720599 31.531132513.08E-08 3.02E-07 -1.329613257

cg13015534 0.092014792 31.5262941 3.09E-08 3.03E-07 1.535856027

cg22721827	0.349887148	31.51434083	3.11E-08	3.04E-07	-1.258624041
cg19778698	0.084409054	31.51342938	3.11E-08	3.04E-07	-1.280015019
cg05719902	0.177366654	31.50882606	3.11E-08	3.04E-07	1.315412941
cg13530039	0.174688753	31.38146056	3.31E-08	3.23E-07	1.282587348
cg19896198	0.14860955	31.36584994	3.34E-08	3.25E-07	-1.362035896
cg16848873	0.432365844	31.35285674	3.36E-08	3.27E-07	-1.324512846
cg24664957	0.660060242	31.32680229	3.40E-08	3.30E-07	-1.219397437
cg21301148	0.410307507	31.29888376	3.45E-08	3.34E-07	1.27739671
cg08527127	0.48736617	31.24552866	3.54E-08	3.42E-07	-1.254855182
cg13470920	0.349558175	31.18546361	3.65E-08	3.52E-07	1.29617481
cg00229387	0.106329272	31.03844347	3.92E-08	3.75E-07	1.42891699
cg15798455	0.113750615	30.98606136	4.02E-08	3.83E-07	-1.542522351
cg02983451	0.23947734	30.98152473	4.03E-08	3.84E-07	-1.502806209
cg03562120	0.326173717	30.97771999	4.04E-08	3.84E-07	1.426402846
cg15443822	0.115621528	30.94908568	4.09E-08	3.89E-07	-1.524167849

cg10398682	0.297918637	30.93883563	4.11E-08	3.90E-07	-1.34154344
cg13795840	0.697132572	30.91360202	4.17E-08	3.95E-07	-1.221960977
cg17166812	0.187454258	30.85512611	4.29E-08	4.05E-07	1.460976065
cg26410550	0.431941265	30.8426242	4.31E-08	4.06E-07	1.25094173
cg08924430	0.628970301	30.83630091	4.33E-08	4.07E-07	-1.238428317
cg20368904	0.479915949	30.82500617	4.35E-08	4.09E-07	1.250788888
cg01426743	0.527848911	30.75723457	4.50E-08	4.21E-07	-1.247319377
cg21475402	0.252971897	30.7544242	4.50E-08	4.21E-07	-1.526973735
cg04716261	0.185969048	30.73931926	4.54E-08	4.24E-07	-1.279792625
cg23349242	0.315600871	30.70915592	4.60E-08	4.29E-07	1.253026259
cg19946699	0.221508342	30.69013594	4.65E-08	4.32E-07	1.28380868
cg16495265	0.06074756	30.67737597	4.67E-08	4.35E-07	3.176405377
cg11473104	0.308876947	30.63385238	4.78E-08	4.43E-07	1.212338968
cg19008097	0.326062017	30.6071056	4.84E-08	4.48E-07	-1.301114786
cg25494227	0.218388556	30.57517817	4.91E-08	4.55E-07	-

1.297101502

cg17465631 0.157047815 30.51599855 5.06E-08 4.66E-07

1.252740676

cg07546360 0.262491701 30.46776078 5.18E-08 4.77E-07

1.422449869

cg11618577 0.281648429 30.45204324 5.22E-08 4.80E-07

1.265591598

cg12188860 0.215897578 30.32693969 5.55E-08 5.09E-07

1.396968678

cg19764436 0.086854664 30.26547482 5.72E-08 5.23E-07 -

1.463077027

cg14679202 0.216178389 30.26343425 5.72E-08 5.23E-07 -

1.404071546

cg09649610 0.303943869 30.25199981 5.76E-08 5.25E-07 -

1.397657861

cg12285118 0.45944426 30.22580019 5.83E-08 5.31E-07 -1.242509256

cg19139729 0.561717722 30.212511655.87E-08 5.34E-07 -1.234953022

cg24421410 0.409343126 30.19656301 5.91E-08 5.38E-07

1.292857823

cg25552492 0.085203904 30.193811975.92E-08 5.38E-07 -1.370729321

cg22797169 0.14710466 30.161186026.02E-08 5.46E-07 1.393310567

cg19884658 0.578170803 30.13025358 6.11E-08 5.54E-07 -

1.273912992

cg06817269 0.237309995 30.10098618 6.20E-08 5.61E-07 1.35001895

cg06539804 0.033760523 30.07230175 6.28E-08 5.67E-07 -

1.356025393

cg19145398 0.376005726 30.02757067 6.42E-08 5.78E-07

1.225267461

cg08634464 0.192780468 29.91909728 6.77E-08 6.09E-07

1.482354374

cg10458876 0.072887005 29.91216656 6.80E-08 6.10E-07

1.256929249

cg03702236 0.226426533 29.87695003 6.92E-08 6.20E-07 -

1.498178536

cg07388493 0.429088804 29.80730771 7.16E-08 6.40E-07 -

1.219418859

cg01870826 0.121560654 29.75682802 7.34E-08 6.55E-07 -

1.553427241

cg22402007 0.176289535 29.73918914 7.40E-08 6.60E-07 -

1.452006573

cg13439730 0.25002311929.70640362 7.52E-08 6.69E-07 1.297000293

cg10694914 0.434061022 29.69882286 7.55E-08 6.71E-07 -

1.293526097

cg14759043 0.327125703 29.67538468 7.63E-08 6.78E-07 -

1.282322993

cg05500015 0.11502431429.67316848 7.64E-08 6.78E-07 -1.546147638

cg01081263 0.306746349 29.66322312 7.68E-08 6.81E-07 -

1.329360831

cg17675882 0.066585922 29.6222008 7.84E-08 6.93E-07 1.276077878

cg00888561 0.023496912 29.59730907 7.93E-08 7.00E-07 -

1.210603197

cg07730301 0.097243268 29.59189348 7.95E-08 7.02E-07

1.559728366

cg08446111 0.131961973 29.57259419 8.03E-08 7.08E-07

1.479498733

cg10919204 0.41123595929.54393678 8.14E-08 7.17E-07 -1.335693364

cg26267310 0.408923415 29.50776724 8.29E-08 7.29E-07 -

1.359964106

cg27256309 0.204108522 29.45401603 8.51E-08 7.47E-07

1.276002211

cg20916523 0.404138614 29.42854433 8.62E-08 7.54E-07 -

1.383037936

cg02237119 0.173402685 29.4017099 8.73E-08 7.63E-07 1.338363169

cg16268429 0.089093734 29.39945576 8.74E-08 7.63E-07

1.250172676

cg16536450 0.514157804 29.38012188 8.82E-08 7.69E-07 -

1.200900787

cg06424894 0.154204631 29.37053959 8.86E-08 7.72E-07 -

1.366031347

cg21907579 0.479526444 29.27992467 9.27E-08 8.04E-07

1.283150567

cg09001777 0.167471383 29.25616734 9.38E-08 8.12E-07

1.482267645

cg12508624 0.38311347729.221131529.54E-08 8.24E-07 -1.399705381

cg13797282 0.29111373229.19712136 9.65E-08 8.33E-07 1.274584123

cg03424436 0.030859901 29.18503616 9.71E-08 8.36E-07 -

1.201568926

cg04329382 0.174174758 29.12497072 1.00E-07 8.60E-07

1.700154858

cg06540941 0.075367086 29.12141019 1.00E-07 8.60E-07

1.239331389

cg03317245 0.433946963 29.098381121.01E-07 8.69E-07 -1.20924046

cg15503752 0.13872834 29.04043358 1.04E-07 8.92E-07 1.438268303

cg10162464 0.094509488 29.03974348 1.04E-07 8.92E-07

1.232748638

cg27238470 0.231457869 28.97606675 1.08E-07 9.18E-07

1.267945439

cg06800962 0.076981378 28.95809089 1.09E-07 9.25E-07 1.82754199

cg09313705	0.23965989	28.8850351	1.12E-07	9.55E-07	1.462971233
cg18760752	0.066783519	28.83350738	1.15E-07	9.76E-07	1.269885248
cg24855780	0.162514295	28.82632893	1.16E-07	9.79E-07	1.388013978
cg00273068	0.436999705	28.74982702	1.20E-07	1.01E-06	-1.263494316
cg16779976	0.125029388	28.7388633	1.21E-07	1.02E-06	1.656756744
cg27243140	0.223462841	28.67776	1.25E-07	1.05E-06	-1.362780735
cg17063929	0.24070907	28.66499989	1.25E-07	1.05E-06	-1.369486804
cg12438037	0.227235302	28.61456577	1.28E-07	1.08E-06	1.429385175
cg19335327	0.444634002	28.49474837	1.36E-07	1.14E-06	1.244804332
cg00687686	0.040951699	28.47339444	1.38E-07	1.15E-06	-1.445924811
cg05657090	0.588625586	28.43122385	1.41E-07	1.18E-06	-1.210121829
cg27109971	0.123195646	28.39116591	1.43E-07	1.20E-06	1.241753315
cg06577005	0.033616296	28.36501545	1.45E-07	1.21E-06	-1.215837057
cg00995520	0.459840688	28.35114868	1.46E-07	1.22E-06	-1.299824473

cg10414058 0.250682025 28.34361076 1.47E-07 1.22E-06 -1.324257426

cg15228639 0.375183353 28.28699242 1.51E-07 1.26E-06 1.270780889

cg12220493 0.065848518 28.28474047 1.51E-07 1.26E-06 -1.558446451

cg02537838 0.208474641 28.2723442 1.52E-07 1.26E-06 1.298773128

cg24476569 0.140798135 28.18057956 1.59E-07 1.32E-06 -1.329987509

cg20857455 0.05486409 28.17141388 1.60E-07 1.32E-06 2.602110683

cg17891123 0.11827398528.1516191 1.61E-07 1.33E-06 1.255788596

cg19580810 0.370666245 28.08957507 1.66E-07 1.37E-06 1.33779322

cg00411097 0.17804861128.06138301 1.69E-07 1.39E-06 1.292574732

cg05660795 0.086051723 28.03870599 1.71E-07 1.40E-06 -1.384396909

cg05590982 0.200820529 28.02394678 1.72E-07 1.41E-06 1.574677591

cg24743310 0.272075084 27.99020973 1.75E-07 1.43E-06 1.338940119

cg18938204 0.064439235 27.96557381 1.77E-07 1.45E-06 -1.437196315

cg22620680 0.057247031 27.94384137 1.79E-07 1.46E-06 1.22631523

cg20786074	0.348634797	27.87243322	1.85E-07	1.51E-06	-1.301192517
cg11874272	0.381147993	27.82889691	1.89E-07	1.54E-06	1.227874587
cg13210534	0.223552521	27.79038231	1.93E-07	1.57E-06	1.305759574
cg25691167	0.322797423	27.75997685	1.96E-07	1.59E-06	1.467742636
cg08831744	0.113568105	27.75211241	1.96E-07	1.60E-06	-1.438330181
cg07533529	0.075331563	27.7293306	1.99E-07	1.61E-06	1.239275818
cg09381003	0.112131565	27.71587494	2.00E-07	1.62E-06	-1.407891129
cg00430287	0.442354397	27.65345535	2.06E-07	1.67E-06	1.268312752
cg17877656	0.020367391	27.64945302	2.07E-07	1.67E-06	-1.353269985
cg23092823	0.125688786	27.55161053	2.17E-07	1.75E-06	-1.559953925
cg05266781	0.175094937	27.51465812	2.21E-07	1.78E-06	-1.46492668
cg14785479	0.226929204	27.50822519	2.21E-07	1.78E-06	-1.446181335
cg15403517	0.275170998	27.45031019	2.28E-07	1.83E-06	-1.408118726

cg23499956 0.18094729 27.43224841 2.30E-07 1.84E-06 1.459207455

cg13749822 0.189448712 27.42750462 2.30E-07 1.85E-06 -

1.376358372

cg05429895 0.301963345 27.36607791 2.38E-07 1.90E-06 -

1.348075285

cg10705800 0.11876663927.3554462 2.39E-07 1.90E-06 1.561127503

cg20284673 0.128851246 27.3427549 2.40E-07 1.91E-06 1.213312447

cg01427567 0.100831883 27.33357658 2.41E-07 1.92E-06

1.209780053

cg16361890 0.219406278 27.32412972 2.43E-07 1.93E-06

1.461960815

cg02330106 0.347580151 27.32282698 2.43E-07 1.93E-06 -

1.263991912

cg21435336 0.046225617 27.31269094 2.44E-07 1.94E-06 -

1.550888684

cg26862286 0.501752656 27.30663893 2.45E-07 1.94E-06 -

1.215905452

cg21692936 0.040298057 27.30484309 2.45E-07 1.94E-06 -

1.393593828

cg03543593 0.312412076 27.29779079 2.46E-07 1.94E-06 -

1.328560372

cg10667970 0.129155106 27.2944286 2.46E-07 1.94E-06 1.5737188

cg18342900	0.153470152	27.24470682	2.52E-07	1.99E-06	1.208416353
cg08314660	0.425651538	27.1999134	2.58E-07	2.03E-06	1.218829253
cg09837648	0.232824897	27.1930134	2.59E-07	2.04E-06	1.359668816
cg26980692	0.064736405	27.16025772	2.63E-07	2.06E-06	2.206841611
cg12875426	0.316009206	27.14313524	2.65E-07	2.08E-06	-1.285455659
cg26565975	0.671134248	27.11864948	2.68E-07	2.09E-06	-1.207093183
cg16396417	0.080624999	27.11723462	2.69E-07	2.09E-06	1.285986927
cg25949363	0.142514573	27.05935745	2.76E-07	2.15E-06	1.441220004
cg15585987	0.26535632	27.04420701	2.78E-07	2.16E-06	-1.310221447
cg24166628	0.337735173	27.02189483	2.82E-07	2.18E-06	1.210216761
cg09937039	0.531115749	27.0182796	2.82E-07	2.18E-06	-1.241949759
cg19246110	0.102805889	27.00221317	2.84E-07	2.20E-06	2.653175438
cg19853760	0.079944536	26.99473636	2.85E-07	2.21E-06	1.751706826
cg15164103	0.084000866	26.98808618	2.86E-07	2.21E-06	1.416965312

cg11213150 0.344933038 26.9871244 2.86E-07 2.21E-06 -1.280705978

cg15417244 0.55853532 26.98620603 2.87E-07 2.21E-06 -1.228014958

cg14211646 0.049404309 26.96526513 2.90E-07 2.23E-06

1.267968103

cg21052164 0.19560991 26.94363846 2.93E-07 2.25E-06 -1.310883702

cg12105450 0.094912603 26.9223297 2.96E-07 2.27E-06 1.585008826

cg00910067 0.080831284 26.92202191 2.96E-07 2.27E-06 -

1.357974096

cg11554507 0.629954717 26.84650507 3.07E-07 2.36E-06 -

1.220001602

cg11783497 0.17463811326.80966866 3.13E-07 2.40E-06 1.522017731

cg12936747 0.14032065 26.80050634 3.14E-07 2.40E-06 -1.38972927

cg16426459 0.046016067 26.78928355 3.16E-07 2.41E-06

1.951067098

cg06258834 0.101341932 26.78891843 3.16E-07 2.41E-06

1.455730973

cg19998328 0.374469271 26.76188921 3.20E-07 2.44E-06

1.249533865

cg08205865 0.484914834 26.75941872 3.21E-07 2.44E-06 -

1.226225873

cg06636463 0.061483522 26.73885018 3.24E-07 2.47E-06 -

1.492722479

cg12914657 0.215750761 26.711216913.28E-07 2.50E-06 -1.394425139

cg06456031 0.083471079 26.68365925 3.33E-07 2.53E-06

2.306010644

cg22190114 0.540736134 26.63264252 3.41E-07 2.59E-06 -

1.246108047

cg19384697 0.133392699 26.63097502 3.42E-07 2.59E-06

1.273725754

cg20798152 0.452749834 26.62802655 3.42E-07 2.59E-06

1.221317533

cg03490200 0.487606923 26.60204544 3.46E-07 2.62E-06

1.235280168

cg04576021 0.297244981 26.58179835 3.50E-07 2.64E-06

1.437665412

cg11832722 0.178323347 26.54356604 3.57E-07 2.69E-06 -

1.478732953

cg25432696 0.253499478 26.49894684 3.65E-07 2.74E-06 -

1.33208655

cg25983380 0.34311341826.46806984 3.70E-07 2.78E-06 -1.327870975

cg09906488 0.093274536 26.45022407 3.73E-07 2.80E-06 -

1.562408518

cg24385322 0.045368579 26.44636764 3.74E-07 2.80E-06 -

1.477920749

cg09630437	0.078768772	26.4413583	3.75E-07	2.81E-06	-1.333153249
cg09214254	0.203704084	26.43995651	3.75E-07	2.81E-06	-1.392031418
cg25140571	0.121817956	26.42244833	3.79E-07	2.83E-06	1.236200993
cg06993413	0.023671763	26.39661082	3.83E-07	2.86E-06	-1.328471514
cg26911787	0.080867256	26.35256505	3.92E-07	2.91E-06	-1.372454651
cg08555612	0.073598618	26.33924628	3.94E-07	2.92E-06	-1.610309762
cg21296676	0.58176729	26.32789649	3.97E-07	2.94E-06	-1.232694896
cg27154163	0.039441074	26.31739038	3.99E-07	2.95E-06	-1.563932602
cg24715245	0.09889746	26.28909589	4.04E-07	2.99E-06	-1.506096887
cg22362636	0.139864459	26.27400348	4.07E-07	3.01E-06	-1.400608934
cg26073060	0.306948581	26.23698349	4.15E-07	3.06E-06	1.219508456
cg12910797	0.305187945	26.18043753	4.27E-07	3.14E-06	1.519372136
cg20970875	0.026254438	26.11522342	4.41E-07	3.24E-06	-1.363830753

cg00840516	0.248674777	26.11504605	4.41E-07	3.24E-06	1.301999597
cg00884529	0.071147122	26.09601236	4.45E-07	3.26E-06	1.24840714
cg22190705	0.078243561	26.09460195	4.45E-07	3.26E-06	-1.402181303
cg16425577	0.193938814	26.08658957	4.47E-07	3.27E-06	-1.358863242
cg27496506	0.198617215	26.0677695	4.51E-07	3.30E-06	1.349540924
cg20664201	0.512726256	26.04975027	4.55E-07	3.33E-06	-1.202519354
cg03064067	0.142080204	26.04771186	4.56E-07	3.33E-06	-1.394989309
cg17243643	0.137256339	26.02981681	4.60E-07	3.35E-06	1.527690672
cg00638514	0.065046833	26.02634151	4.61E-07	3.35E-06	-1.590485311
cg09432154	0.13948746	25.97883403	4.72E-07	3.43E-06	1.588296636
cg08450982	0.422011499	25.94918782	4.79E-07	3.47E-06	-1.269586177
cg05316065	0.066355505	25.93947107	4.81E-07	3.49E-06	1.958462361
cg11976790	0.122026421	25.91275252	4.87E-07	3.53E-06	1.251540037
cg03148461	0.081169457	25.87747272	4.96E-07	3.59E-06	1.275600353
cg03017653	0.194501944	25.8112009	5.12E-07	3.69E-06	1.378483969

cg20050113	0.167855117	25.77271496	5.22E-07	3.75E-06	-1.362711518
cg08749917	0.35737138	25.76096685	5.25E-07	3.77E-06	-1.243197639
cg00673191	0.273416385	25.69498213	5.43E-07	3.89E-06	1.324732894
cg22054191	0.342089667	25.69041533	5.44E-07	3.90E-06	-1.253170147
cg10710439	0.517470339	25.65221475	5.54E-07	3.96E-06	-1.210042874
cg07766612	0.102925975	25.62774593	5.61E-07	4.01E-06	-1.606079176
cg03561565	0.335809406	25.59640824	5.70E-07	4.05E-06	-1.28983394
cg16864658	0.258739905	25.52857597	5.89E-07	4.18E-06	-1.41240086
cg03167883	0.436752709	25.52200343	5.91E-07	4.19E-06	-1.271853556
cg10092957	0.156664414	25.50848345	5.95E-07	4.21E-06	-1.354016949
cg01772980	0.162224737	25.49322099	6.00E-07	4.24E-06	1.291034821
cg13765621	0.378304838	25.47181883	6.06E-07	4.28E-06	-1.258631855

cg08137040	0.131064898	25.4616016	6.09E-07	4.30E-06	-1.312947495
cg11105610	0.199913706	25.44592012	6.14E-07	4.33E-06	1.312997223
cg06554069	0.128866052	25.43915898	6.16E-07	4.34E-06	1.274036569
cg25372103	0.097458254	25.36137475	6.40E-07	4.50E-06	1.24297584
cg24122922	0.466450516	25.33488933	6.49E-07	4.55E-06	-1.255442283
cg00386408	0.129662378	25.28923086	6.64E-07	4.64E-06	-1.422729998
cg26815021	0.160708641	25.27555857	6.68E-07	4.67E-06	1.329782909
cg12360736	0.41971994	25.25790012	6.74E-07	4.71E-06	-1.279577382
cg23067535	0.083800639	25.22049276	6.87E-07	4.79E-06	1.871828505
cg03682823	0.371971603	25.2076038	6.91E-07	4.82E-06	1.206481346
cg04636557	0.489917724	25.20472941	6.92E-07	4.82E-06	-1.220599003
cg25042226	0.333584959	25.18727039	6.98E-07	4.86E-06	1.2568225
cg10273210	0.036754959	25.14262	7.14E-07	4.95E-06	3.467538975
cg17729667	0.202435135	25.14256171	7.14E-07	4.95E-06	-1.380607289

cg06650786 0.184949415 25.114831797.24E-07 5.02E-06 -1.263843199

cg09196959 0.464790788 25.08350719 7.35E-07 5.09E-06 -

1.214456116

cg23447996 0.073655257 25.06019195 7.44E-07 5.15E-06

1.413183411

cg04597449 0.226061556 24.98878543 7.70E-07 5.31E-06 -

1.368571875

cg20291049 0.313318134 24.97366633 7.76E-07 5.34E-06 -

1.348101024

cg10780112 0.04764174 24.9232942 7.96E-07 5.47E-06 1.214731489

cg07215749 0.041758722 24.91884434 7.98E-07 5.48E-06 -

1.410529173

cg10236239 0.506780323 24.911259988.01E-07 5.50E-06 -1.261412359

cg23239828 0.078157019 24.88466036 8.11E-07 5.56E-06 -

1.321411025

cg25577842 0.068766976 24.86010712 8.21E-07 5.62E-06 2.59802542

cg26607785 0.327754396 24.84256487 8.28E-07 5.67E-06 -

1.284810305

cg26489108 0.435988816 24.81441883 8.40E-07 5.72E-06

1.286168479

cg09522147 0.167135748 24.79601565 8.48E-07 5.77E-06

1.437513283

cg18750960	0.145453035	24.75492115	8.65E-07	5.88E-06	1.94313684
cg00711916	0.030942592	24.74455525	8.70E-07	5.91E-06	1.261432143
cg21750589	0.260276659	24.6998617	8.89E-07	6.03E-06	1.351361918
cg09432376	0.402512741	24.6983208	8.90E-07	6.03E-06	1.276263308
cg01405107	0.175998539	24.69356127	8.92E-07	6.04E-06	1.657324586
cg19324627	0.233112656	24.6807328	8.98E-07	6.07E-06	1.325015466
cg15784332	0.155436432	24.67148343	9.02E-07	6.09E-06	-1.452293051
cg25554036	0.052171868	24.65412361	9.10E-07	6.14E-06	-1.321536189
cg14832904	0.414321375	24.61204954	9.29E-07	6.26E-06	1.337162868
cg07443748	0.356200841	24.53102665	9.67E-07	6.50E-06	1.405845963
cg26293512	0.1013255	24.48328047	9.90E-07	6.65E-06	1.592302431
cg03923277	0.369668084	24.46835672	9.98E-07	6.70E-06	1.256632079
cg21197871	0.106512262	24.42302773	1.02E-06	6.84E-06	-1.279137238
cg06521280	0.064132254	24.40125521	1.03E-06	6.90E-06	-

1.428615469

cg11394785 0.283046319 24.38450728 1.04E-06 6.96E-06

1.429512569

cg25664034 0.092943477 24.35548826 1.06E-06 7.05E-06

1.279670024

cg07072643 0.539322896 24.33835554 1.06E-06 7.09E-06 -

1.242576952

cg10016608 0.21213571124.29221248 1.09E-06 7.24E-06 1.300712524

cg15147516 0.063083851 24.24106091 1.12E-06 7.41E-06 -

1.501569967

cg03139377 0.105082459 24.21049128 1.13E-06 7.52E-06

1.504286356

cg04052038 0.479484054 24.20232339 1.14E-06 7.54E-06 -

1.214465439

cg24331162 0.23640276 24.14453914 1.17E-06 7.75E-06 1.245901365

cg24210717 0.20416316 24.13314544 1.18E-06 7.77E-06 1.245004655

cg15777781 0.555797033 24.114282631.19E-06 7.84E-06 -1.210786614

cg07899016 0.350862149 24.111534371.19E-06 7.85E-06 1.358806097

cg06946880 0.305051381 24.110662011.19E-06 7.85E-06 1.228835792

cg26530341 0.514472245 24.09353297 1.20E-06 7.90E-06 -

1.250407454

cg12759554 0.457155092 24.06782515 1.22E-06 7.99E-06

1.250430165

cg24981018 0.272783368 24.06535452 1.22E-06 8.00E-06

1.215863744

cg17483510 0.225644652 24.0526586 1.23E-06 8.05E-06 -1.38667722

cg00412772 0.21319721 24.02801832 1.24E-06 8.13E-06 1.327949874

cg01656216 0.46583446 23.99755154 1.26E-06 8.25E-06 1.205389249

cg08261841 0.165222128 23.99276572 1.26E-06 8.26E-06

1.367458365

cg01657207 0.14740911 23.95648368 1.29E-06 8.41E-06 -1.348055187

cg26096837 0.383141841 23.94797254 1.29E-06 8.44E-06 -

1.267215632

cg16215361 0.199366059 23.9274694 1.31E-06 8.51E-06 1.388218988

cg13614181 0.322279703 23.9077826 1.32E-06 8.59E-06 -1.302211217

cg05889321 0.100440776 23.90603205 1.32E-06 8.59E-06

1.239189858

cg14963371 0.140789441 23.89763339 1.33E-06 8.62E-06 -

1.265972002

cg22189019 0.435950777 23.88185132 1.34E-06 8.68E-06 -

1.227391515

cg12880658 0.273203666 23.83081065 1.37E-06 8.90E-06 1.55792686

cg13591701 0.107037865 23.81907847 1.38E-06 8.94E-06

1.207739309

cg21505886 0.284445547 23.81395181 1.38E-06 8.96E-06

1.323656165

cg05065690 0.03110603923.75202089 1.43E-06 9.22E-06 1.210421946

cg05958352 0.40385211623.74456987 1.43E-06 9.25E-06 1.255670399

cg18746357 0.291717998 23.71972057 1.45E-06 9.36E-06

1.218647427

cg22536398 0.015126015 23.68829323 1.47E-06 9.49E-06 -

1.27564056

cg24924779 0.055746851 23.63764791 1.51E-06 9.71E-06 -

1.537154373

cg03630088 0.179879335 23.57826608 1.55E-06 1.00E-05 -

1.383615851

cg06589885 0.160176133 23.56684239 1.56E-06 1.00E-05 1.43119861

cg04301614 0.08671142223.54631524 1.58E-06 1.01E-05 1.684042391

cg25303383 0.250100429 23.54320786 1.58E-06 1.01E-05

1.293485286

cg08005849 0.185281264 23.54305618 1.58E-06 1.01E-05 -

1.35512274

cg17861230 0.41631163323.54047156 1.58E-06 1.01E-05 -1.334070058

cg01612158 0.474617502 23.53041227 1.59E-06 1.02E-05 -

1.226693246

cg12072001 0.127647971 23.51586255 1.60E-06 1.03E-05

1.637662741

cg02506426 0.052444584 23.51248872 1.61E-06 1.03E-05

1.252330127

cg11814446 0.087163302 23.47544698 1.64E-06 1.04E-05 -

1.456796355

cg08017606 0.055313221 23.44992754 1.66E-06 1.06E-05

1.239973175

cg15321211 0.21837489 23.44299605 1.66E-06 1.06E-05 -1.375297637

cg12144803 0.151221423 23.34240466 1.75E-06 1.11E-05

1.439263882

cg07758904 0.077744316 23.32729827 1.76E-06 1.12E-05 -

1.422353671

cg13968061 0.524716074 23.30633739 1.78E-06 1.13E-05 -

1.230933985

cg26482939 0.355303042 23.29789518 1.79E-06 1.13E-05 -

1.277342383

cg12970724 0.11767595 23.27718785 1.81E-06 1.14E-05 1.202221017

cg12195135 0.076387047 23.27104937 1.81E-06 1.14E-05 -

1.341415647

cg15840985 0.11963950423.24512009 1.84E-06 1.16E-05 1.290152096

cg21245372 0.249319057 23.23722229 1.84E-06 1.16E-05 -

1.350263202

cg09340639	0.220866023	23.22505395	1.85E-06	1.17E-05	-1.323826252
cg18959422	0.344208795	23.21931144	1.86E-06	1.17E-05	1.266770973
cg18442986	0.045993012	23.21721441	1.86E-06	1.17E-05	1.216448931
cg13121699	0.113127768	23.17111675	1.90E-06	1.20E-05	-1.48734319
cg04682845	0.060838027	23.13801348	1.94E-06	1.22E-05	-1.365301076
cg14345882	0.434050075	23.13762709	1.94E-06	1.22E-05	1.249194575
cg23663332	0.370031704	23.12434532	1.95E-06	1.22E-05	-1.224339153
cg19205533	0.277809672	23.112435	1.96E-06	1.23E-05	1.328912694
cg06554928	0.036372534	23.10783965	1.97E-06	1.23E-05	1.227956845
cg27091787	0.216687343	23.10025898	1.97E-06	1.23E-05	1.412003212
cg10869069	0.045284946	23.09173345	1.98E-06	1.24E-05	1.220152943
cg25682936	0.086844072	23.08009968	1.99E-06	1.24E-05	1.253656767
cg08876932	0.162544903	23.04752614	2.03E-06	1.26E-05	-

1.495855716

cg18192417 0.192214031 23.0312298 2.04E-06 1.27E-05 -1.3379005

cg10692870 0.306701697 23.00950627 2.07E-06 1.28E-05 -

1.32695542

cg18790143 0.544036992 22.99217531 2.08E-06 1.29E-05 -

1.215414972

cg17264618 0.082580867 22.99073351 2.08E-06 1.29E-05 -

1.407558228

cg18661868 0.163087065 22.97365874 2.10E-06 1.30E-05

1.254810991

cg05786809 0.090467519 22.96685783 2.11E-06 1.30E-05 -

1.567937824

cg03705396 0.04306126 22.95461021 2.12E-06 1.31E-05 -1.398701051

cg24516901 0.54381137722.94592451 2.13E-06 1.31E-05 -1.237080519

cg22705929 0.190551505 22.93628432 2.14E-06 1.32E-05 -

1.268068168

cg11154542 0.104161367 22.92655886 2.15E-06 1.32E-05 -

1.317875634

cg17342283 0.371932663 22.91842851 2.16E-06 1.33E-05

1.271250972

cg07027685 0.048654063 22.90096103 2.18E-06 1.34E-05

1.221663648

cg00282347 0.142956586 22.89730413 2.18E-06 1.34E-05 -

1.453559972

cg10917602 0.131603772 22.89083568 2.19E-06 1.34E-05

1.412181227

cg20579480 0.346066529 22.89039995 2.19E-06 1.34E-05 -

1.21037588

cg14592099 0.066083551 22.86765306 2.22E-06 1.36E-05

1.998576988

cg14614211 0.11660368122.84742845 2.24E-06 1.37E-05 -1.467985132

cg17200465 0.170318435 22.8390743 2.25E-06 1.37E-05 -1.360144996

cg15286905 0.04331794 22.82122356 2.27E-06 1.38E-05 -1.293156806

cg15736165 0.50348711422.78833154 2.31E-06 1.41E-05 -1.200235567

cg25014318 0.088363585 22.77705606 2.32E-06 1.41E-05 -

1.391921624

cg20910746 0.106630189 22.75227903 2.35E-06 1.43E-05

1.567654346

cg07703401 0.323219265 22.74636923 2.36E-06 1.43E-05 -

1.404587756

cg19242268 0.16856911422.72792095 2.38E-06 1.44E-05 1.574488322

cg01857260 0.214390602 22.70061004 2.41E-06 1.46E-05 -

1.348077625

cg18216249 0.216424649 22.70055133 2.41E-06 1.46E-05

					1.339820957
cg09640202	0.612018153	22.69565373	2.42E-06	1.46E-05	-1.210499016
cg06110728	0.044142933	22.67715567	2.44E-06	1.48E-05	-1.365169259
cg10150530	0.454189612	22.6583917	2.46E-06	1.49E-05	1.251790696
cg25149155	0.096014026	22.65039013	2.47E-06	1.49E-05	1.205424151
cg10080004	0.054721284	22.62953671	2.50E-06	1.51E-05	-1.40227801
cg17199658	0.155248368	22.59209689	2.54E-06	1.53E-05	-1.276685919
cg13044277	0.202814797	22.59099237	2.55E-06	1.53E-05	1.323292989
cg15448245	0.312819069	22.58281187	2.56E-06	1.54E-05	1.263435809
cg21132577	0.360912927	22.5808382	2.56E-06	1.54E-05	-1.26400473
cg24323726	0.169828496	22.58006096	2.56E-06	1.54E-05	1.328464388
cg21210789	0.118111189	22.57010241	2.57E-06	1.54E-05	-1.348067399
cg13870866	0.384433974	22.56821228	2.58E-06	1.54E-05	-1.296202565
cg14060828	0.174751526	22.56794662	2.58E-06	1.54E-05	

1.293028865

cg24585690 0.534344232 22.53930839 2.61E-06 1.56E-05 -

1.215654306

cg16957313 0.092853779 22.491056 2.68E-06 1.60E-05 -1.414669507

cg16752583 0.11848911222.39033349 2.81E-06 1.68E-05 1.415164298

cg06516124 0.109195686 22.34854735 2.87E-06 1.71E-05

2.046873295

cg03699566 0.216283008 22.33791406 2.89E-06 1.72E-05

1.361661861

cg06291867 0.276673541 22.311513642.93E-06 1.74E-05 -1.360176979

cg26571739 0.186735291 22.31023187 2.93E-06 1.74E-05

1.346895464

cg22459146 0.371220258 22.291152612.96E-06 1.75E-05 1.229273007

cg25608949 0.099830737 22.24181816 3.03E-06 1.79E-05

1.385430437

cg25604883 0.02946281122.23268981 3.05E-06 1.80E-05 -1.425361986

cg17215680 0.14436313 22.21343123 3.08E-06 1.82E-05 -1.372783287

cg22632663 0.084447774 22.20868348 3.08E-06 1.82E-05

1.245492143

cg24497819 0.276236159 22.18738379 3.12E-06 1.84E-05

1.278432613

cg06204948 0.0708717 22.18564466 3.12E-06 1.84E-05 2.249086581

cg03513163 0.056959901 22.09475608 3.26E-06 1.92E-05

1.332605013

cg07337598 0.240832792 22.068911143.31E-06 1.94E-05 1.275281677

cg26033681 0.218039213 22.06630615 3.31E-06 1.94E-05

1.394835544

cg14209518 0.38541148922.011139043.40E-06 1.99E-05 1.254275448

cg12251804 0.053543344 21.98929813 3.44E-06 2.01E-05 -

1.543916805

cg23338195 0.506093958 21.9676205 3.48E-06 2.03E-05 1.243672315

cg10052840 0.17400528 21.9618834 3.49E-06 2.03E-05 1.32353756

cg08912400 0.078218692 21.91488541 3.57E-06 2.07E-05 -

1.389290262

cg05072008 0.539530551 21.91230315 3.58E-06 2.07E-05 -

1.255835858

cg02887841 0.131465758 21.89272564 3.61E-06 2.09E-05 -

1.455675866

cg17351116 0.042638536 21.83284989 3.72E-06 2.15E-05

1.260224834

cg19308222 0.472734627 21.829761153.73E-06 2.15E-05 -1.254655583

cg17741572 0.184252864 21.82670968 3.73E-06 2.15E-05

1.499078383

cg24968336 0.08795226 21.79897384 3.79E-06 2.18E-05 -1.372848447

cg21816539 0.20555817 21.79312003 3.80E-06 2.19E-05 -1.443484712

cg25406518 0.11500940321.70555509 3.97E-06 2.27E-05 1.453220931

cg02549424 0.224733742 21.68676179 4.01E-06 2.29E-05

1.265002058

cg07059360 0.150776595 21.67983355 4.02E-06 2.30E-05 -

1.259052043

cg05535113 0.15454121121.67344435 4.03E-06 2.30E-05 1.33366352

cg25859012 0.064713195 21.67228768 4.03E-06 2.30E-05

1.271246511

cg14823162 0.072088573 21.62302489 4.14E-06 2.36E-05 -

1.551387891

cg17606785 0.157990522 21.6162702 4.15E-06 2.36E-05 -1.333073977

cg12288726 0.364418258 21.61584401 4.15E-06 2.36E-05 -

1.243891621

cg07423149 0.105161963 21.59495875 4.19E-06 2.38E-05

1.487035511

cg06148264 0.075448441 21.57585763 4.24E-06 2.40E-05

1.727856452

cg11027330 0.092973169 21.54029263 4.31E-06 2.45E-05 -

1.262096415

cg08263647 0.208667496 21.53788808 4.32E-06 2.45E-05

1.449610381

cg02181506 0.069547186 21.50223703 4.39E-06 2.49E-05

1.329242855

cg18403361 0.29087383 21.47894434 4.45E-06 2.51E-05 -1.297730929

cg03019000 0.510668163 21.46107635 4.49E-06 2.53E-05 -

1.218052841

cg03442064 0.099182683 21.45649366 4.50E-06 2.54E-05

1.222573249

cg25778479 0.11247987421.441611874.53E-06 2.55E-05 1.488263886

cg01880569 0.458964523 21.39913779 4.63E-06 2.61E-05 -

1.214070745

cg24489015 0.335308723 21.34790146 4.75E-06 2.67E-05

1.314905223

cg02016419 0.179636241 21.34786364 4.75E-06 2.67E-05 -

1.285430419

cg11098259 0.240141804 21.31251906 4.83E-06 2.71E-05

1.419478259

cg02046532 0.425364786 21.30574653 4.85E-06 2.72E-05 -

1.200742791

cg22881914 0.51354255 21.27694677 4.92E-06 2.75E-05 -1.297689757

cg09595479 0.275876181 21.25393604 4.98E-06 2.78E-05 -

1.343857271

cg06051311 0.216449892 21.24203252 5.01E-06 2.79E-05 -

1.281194845

cg11970458 0.137744767 21.23627302 5.02E-06 2.80E-05 1.22649011

cg14944269 0.197995908 21.23218833 5.03E-06 2.80E-05 -

1.24064156

cg25481253 0.260872017 21.22182992 5.06E-06 2.81E-05

1.232572176

cg25802871 0.0952686 21.20202126 5.11E-06 2.84E-05 1.206032736

cg16009558 0.22779251 21.18092007 5.16E-06 2.87E-05 1.556253154

cg13460409 0.273708266 21.15688149 5.23E-06 2.90E-05 -

1.342103824

cg21700166 0.071496603 21.12860598 5.30E-06 2.94E-05 -

1.369894224

cg26885858 0.048747978 21.12338106 5.32E-06 2.94E-05 -

1.374157587

cg12638745 0.26011818921.10069372 5.38E-06 2.97E-05 1.262361981

cg10234985 0.566700681 21.10065347 5.38E-06 2.97E-05 -

1.210410737

cg04329454 0.11293527521.08508012 5.42E-06 2.98E-05 1.331971447

cg00347729 0.19945917 21.04319703 5.54E-06 3.04E-05 1.260530048

cg19130550 0.043820785 21.0404266 5.54E-06 3.05E-05 -1.275407557

cg16449464 0.093681928 21.01738926 5.61E-06 3.08E-05 -

1.29734676

cg23547429	0.056214566	20.99614861	5.67E-06	3.11E-05	1.900215696
cg14967066	0.038413511	20.98760276	5.69E-06	3.12E-05	2.370153213
cg16250754	0.198347548	20.96862781	5.75E-06	3.15E-05	1.300932857
cg14008883	0.189054994	20.92964457	5.86E-06	3.20E-05	-1.379501388
cg21410991	0.361220766	20.92158491	5.88E-06	3.22E-05	-1.305612617
cg17207590	0.379269468	20.88173422	6.00E-06	3.28E-05	-1.249707072
cg12072973	0.023104575	20.85552083	6.08E-06	3.31E-05	-1.382065792
cg19005368	0.051601554	20.80587695	6.24E-06	3.39E-05	1.618262874
cg09119967	0.393656191	20.79860995	6.26E-06	3.40E-05	-1.232622913
cg14982472	0.178026916	20.79822395	6.26E-06	3.40E-05	1.458573625
cg23741330	0.403636618	20.77092793	6.35E-06	3.44E-05	-1.218512835
cg18573383	0.091886755	20.74997334	6.42E-06	3.47E-05	-

1.451031085

cg23325242 0.041467862 20.74371201 6.44E-06 3.48E-05

1.480133355

cg24620905 0.10943338 20.72207833 6.51E-06 3.51E-05 1.239974858

cg22406518 0.054673642 20.69535883 6.59E-06 3.55E-05 1.22611974

cg10107725 0.097590718 20.64133979 6.78E-06 3.64E-05

1.430649216

cg16077929 0.29116718920.64076549 6.78E-06 3.64E-05 1.340628583

cg13705284 0.451358952 20.62278771 6.84E-06 3.66E-05

1.220464989

cg23642747 0.244425891 20.6030147 6.91E-06 3.70E-05 -1.30950556

cg25565479 0.090994535 20.59884437 6.92E-06 3.70E-05

1.250791459

cg24868525 0.064592134 20.57741281 7.00E-06 3.74E-05

1.209917424

cg14870461 0.121547732 20.57732774 7.00E-06 3.74E-05 -

1.432078739

cg23131007 0.128991313 20.55894426 7.06E-06 3.77E-05 -

1.428407094

cg24341800 0.148762414 20.55101041 7.09E-06 3.78E-05 1.23438639

cg18441959 0.141651297 20.53954702 7.13E-06 3.80E-05

1.573586761

cg13112336 0.043355818 20.53807656 7.14E-06 3.81E-05 1.229285631

cg00003994 0.101927379 20.53413245 7.15E-06 3.81E-05 -1.390777198

cg08909157 0.157985585 20.52164191 7.20E-06 3.83E-05 -1.338318523

cg11507178 0.108702963 20.5134521 7.23E-06 3.84E-05 1.213560167

cg04311964 0.423607202 20.50806304 7.25E-06 3.85E-05 1.205818403

cg22510943 0.11588626220.49870975 7.28E-06 3.87E-05 1.270046736

cg13120814 0.105038593 20.49650081 7.29E-06 3.87E-05 1.43146604

cg11196870 0.057432087 20.48679057 7.33E-06 3.88E-05 1.216281451

cg21664828 0.045267485 20.47315029 7.38E-06 3.91E-05 1.220468385

cg16864895 0.047886476 20.45532508 7.44E-06 3.94E-05 1.205587539

cg11822659 0.04119888620.45482431 7.44E-06 3.94E-05 1.226563662

cg26912636 0.317638644 20.4377812 7.51E-06 3.97E-05 -1.221186922

cg19686152 0.259172004 20.42808621 7.55E-06 3.98E-05 1.237481617

cg05260966 0.24871194220.38323484 7.72E-06 4.07E-05 -1.248806769

cg24043307 0.109921736 20.3769515 7.74E-06 4.08E-05 1.202453841

cg25661884 0.273317219 20.36817737 7.78E-06 4.09E-05 -

1.268932423

cg20959866 0.315072187 20.35858828 7.81E-06 4.11E-05 -

1.350229375

cg27248887 0.11515093320.33800003 7.90E-06 4.15E-05 1.43657959

cg17860186 0.025420866 20.33127122 7.92E-06 4.16E-05

1.230598967

cg04574507 0.584193074 20.328761127.93E-06 4.17E-05 -1.218385143

cg26125600 0.280127389 20.31632069 7.98E-06 4.19E-05 -

1.289139758

cg25315362 0.251005036 20.30688797 8.02E-06 4.20E-05 -

1.290315541

cg21330703 0.100194019 20.29535655 8.07E-06 4.22E-05 -

1.311795221

cg08752459 0.16861182920.27636919 8.15E-06 4.26E-05 1.399045204

cg14950072 0.062422768 20.22695409 8.35E-06 4.36E-05 -

1.397812804

cg15149645 0.275373964 20.22194903 8.37E-06 4.37E-05

1.492268787

cg12845808 0.150279252 20.20547319 8.44E-06 4.40E-05

1.345122539

cg18618334	0.449453209	20.19414805	8.49E-06	4.43E-05	-1.244079458
cg08504583	0.411708881	20.19323588	8.49E-06	4.43E-05	1.282277878
cg12730381	0.102348928	20.13849422	8.73E-06	4.54E-05	1.246737251
cg15095327	0.14005467	20.1303543	8.77E-06	4.56E-05	1.305244518
cg09298484	0.420125695	20.08283435	8.98E-06	4.66E-05	1.211437996
cg22123464	0.147101399	20.06822239	9.05E-06	4.69E-05	-1.335956883
cg22438810	0.16798396	20.04335699	9.16E-06	4.74E-05	1.310275565
cg07705835	0.089056214	20.04013182	9.18E-06	4.75E-05	1.362604201
cg05252264	0.405610423	20.02236231	9.26E-06	4.78E-05	-1.217407038
cg12624641	0.126238346	20.01919605	9.27E-06	4.79E-05	1.251677462
cg24877842	0.277413419	19.96336191	9.54E-06	4.91E-05	1.40389643
cg03792653	0.069848613	19.9199019	9.75E-06	5.01E-05	-1.429987696
cg24921089	0.194907608	19.87595275	9.97E-06	5.11E-05	1.320560708
cg01293647	0.065685166	19.87228944	9.99E-06	5.12E-05	-1.437609

cg12351433	0.223638104	19.86914081	1.00E-05	5.13E-05	-1.353364345
cg07008386	0.063807999	19.80766976	1.03E-05	5.28E-05	2.22461176
cg27583102	0.372540483	19.76869362	1.05E-05	5.37E-05	1.269336931
cg10073091	0.038284945	19.76838842	1.05E-05	5.37E-05	1.270542421
cg06154597	0.197235508	19.73156481	1.07E-05	5.47E-05	1.256662363
cg05886626	0.109747779	19.70522376	1.09E-05	5.54E-05	1.342635177
cg01892727	0.11258688	19.70267646	1.09E-05	5.54E-05	1.27544235
cg17950095	0.398015149	19.70155355	1.09E-05	5.54E-05	-1.254121559
cg15952487	0.377953322	19.68911691	1.10E-05	5.55E-05	-1.306493725
cg18022496	0.06943836	19.67372539	1.10E-05	5.55E-05	1.215625005
cg26620959	0.126082469	19.66260481	1.11E-05	5.55E-05	-1.392107453
cg09635067	0.032064531	19.63613307	1.13E-05	5.55E-05	-1.352868692
cg07220939	0.33666456	19.63342266	1.13E-05	5.55E-05	-1.280805137
cg06172871	0.371418076	19.59929911	1.15E-05	5.55E-05	1.245165457

cg13548361	0.053525255	19.5962087	1.15E-05	5.55E-05	-1.497177274
cg05859264	0.24543687	19.5904965	1.15E-05	5.55E-05	1.303555332
cg25524473	0.159037199	19.58727736	1.15E-05	5.55E-05	-1.241121775
cg22492020	0.06475755	19.53417632	1.19E-05	5.55E-05	1.629984948
cg24315815	0.158919946	19.49818976	1.21E-05	5.55E-05	1.489748346
cg06378617	0.427257536	19.47536021	1.22E-05	5.55E-05	1.225691004
cg10925082	0.164603834	19.45783975	1.23E-05	5.55E-05	1.583887783
cg09325101	0.385149421	19.45091272	1.24E-05	5.55E-05	-1.243721552
cg20229788	0.107082599	19.43803453	1.24E-05	5.55E-05	-1.241650673
cg09044743	0.043673085	19.43331002	1.25E-05	5.55E-05	-1.358886652
cg18815943	0.134141091	19.40448134	1.27E-05	5.55E-05	-1.527074724
cg03425110	0.091963562	19.38325957	1.28E-05	5.55E-05	-1.517612829
cg23129478	0.389338042	19.37461076	1.28E-05	5.55E-05	-

1.31246139

cg00096922 0.031704984 19.3333961 1.31E-05 5.55E-05 -1.339911023

cg20318748 0.132267719 19.33092332 1.31E-05 5.55E-05 -

1.317476102

cg17992056 0.059375764 19.32463019 1.32E-05 5.55E-05 -

1.498887713

cg08961832 0.241674916 19.32379443 1.32E-05 5.55E-05 -

1.210149627

cg03483626 0.356241952 19.29229755 1.34E-05 5.55E-05 -

1.303439327

cg02588309 0.089653862 19.28504801 1.34E-05 5.55E-05 -

1.321889443

cg27124774 0.086089905 19.25746266 1.36E-05 5.55E-05

1.689670666

cg01346718 0.223950558 19.24601545 1.37E-05 5.55E-05

1.214658506

cg26620157 0.318669323 19.22194652 1.39E-05 5.55E-05 -

1.326039036

cg27185377 0.04835133 19.2204307 1.39E-05 5.55E-05 1.301057982

cg13488201 0.252199256 19.17496894 1.42E-05 5.55E-05 -

1.373602768

cg19826026 0.083972812 19.15015168 1.44E-05 5.55E-05 1.82520388

cg08578023 0.161799412 19.14663869 1.44E-05 5.55E-05

1.331852437

cg23320056 0.056105125 19.122593 1.46E-05 5.55E-05 1.252432346

cg25124030 0.228353904 19.08622985 1.49E-05 5.55E-05

1.270910278

cg26331247 0.329687419 19.08352866 1.49E-05 5.55E-05 -

1.215518565

cg17469978 0.045963057 19.05933643 1.51E-05 5.55E-05 -

1.344375965

cg13727946 0.386628 19.03427772 1.53E-05 5.55E-05 -1.204120844

cg10334385 0.04018933 19.02455353 1.53E-05 5.55E-05 1.870623229

cg18677965 0.02645002 19.01916364 1.54E-05 5.55E-05 1.227133769

cg06751597 0.080394805 19.00282171 1.55E-05 5.55E-05 1.23941401

cg17456704 0.08113348818.99361931 1.56E-05 5.55E-05 1.219491734

cg04123507 0.55610904 18.98864951 1.56E-05 5.55E-05 -1.210907215

cg11801011 0.297084968 18.96147307 1.58E-05 5.55E-05 1.22898993

cg12716838 0.042220871 18.95833691 1.59E-05 5.55E-05 -

1.596418791

cg00698688 0.221806478 18.95563796 1.59E-05 5.55E-05

1.270411071

cg13798289 0.261330485 18.94177224 1.60E-05 5.55E-05

1.247998862

cg09300114 0.090871344 18.90622855 1.63E-05 5.55E-05

1.803053781

cg16933388 0.027336075 18.86397144 1.66E-05 5.55E-05 -

1.304732379

cg00231140 0.06073261118.85037012 1.68E-05 5.55E-05 -1.513213003

cg02089348 0.195299041 18.84874487 1.68E-05 5.55E-05

1.269788039

cg19664945 0.320584325 18.844110341.68E-05 5.55E-05 1.368137716

cg10106388 0.412627191 18.75868249 1.76E-05 5.55E-05 -

1.202743012

cg21168622 0.174202328 18.74053257 1.77E-05 5.55E-05

1.255673699

cg21929875 0.48716079 18.73859563 1.77E-05 5.55E-05 1.220647636

cg08999895 0.11892298 18.72736238 1.78E-05 5.55E-05 1.350996966

cg26431343 0.34019110118.71570371 1.79E-05 5.55E-05 -1.226742615

cg25230532 0.11155253718.69829256 1.81E-05 5.55E-05 1.214417289

cg17866455 0.277410932 18.66348682 1.84E-05 5.55E-05 -

1.235419391

cg13351583 0.158028931 18.65641416 1.85E-05 5.55E-05 -

1.300218302

cg04797323 0.552880875 18.65461737 1.85E-05 5.55E-05 -

1.276885257

cg05989054	0.487254291	18.63851554	1.87E-05	5.55E-05	-1.213100046
cg25465406	0.285922505	18.63735571	1.87E-05	5.55E-05	-1.344595855
cg23854009	0.031816663	18.62096136	1.88E-05	5.55E-05	-1.207937791
cg17998964	0.067242736	18.59488348	1.91E-05	5.55E-05	1.96701708
cg07889201	0.060234465	18.55277079	1.95E-05	5.55E-05	-1.278084237
cg10525488	0.217846334	18.55112761	1.95E-05	5.55E-05	-1.213049504
cg00308665	0.483555194	18.5362102	1.97E-05	5.55E-05	-1.208324752
cg10214058	0.036373597	18.52782733	1.97E-05	5.55E-05	1.206661219
cg05026186	0.425825621	18.51883939	1.98E-05	5.55E-05	-1.230548683
cg14221171	0.362017376	18.48699405	2.02E-05	5.55E-05	-1.292520045
cg26112639	0.338412013	18.48666736	2.02E-05	5.55E-05	-1.211309532
cg01765473	0.011843937	18.44880814	2.05E-05	5.55E-05	-1.240702174
cg00302793	0.015683743	18.41412962	2.09E-05	5.55E-05	1.218552564

cg20773127	0.31999924	18.4094379	2.10E-05	5.55E-05	1.384012177
cg22464186	0.044896098	18.38526558	2.12E-05	5.55E-05	1.245727666
cg08694544	0.024987488	18.37620569	2.13E-05	5.55E-05	-1.436781161
cg19226099	0.381587201	18.37123605	2.14E-05	5.55E-05	-1.207449655
cg14188232	0.040868931	18.35807857	2.15E-05	5.55E-05	-1.412844714
cg14696870	0.385301021	18.34395636	2.17E-05	5.55E-05	-1.283293309
cg20029201	0.127398418	18.33469599	2.18E-05	5.55E-05	1.357456232
cg15819333	0.219138593	18.32595911	2.19E-05	5.55E-05	1.220911636
cg01348086	0.348519291	18.31201269	2.20E-05	5.55E-05	1.262700608
cg00678539	0.13371122	18.31112592	2.20E-05	5.55E-05	-1.322380468
cg03643709	0.096391051	18.29309608	2.22E-05	5.55E-05	1.257397724
cg13673514	0.372412749	18.26730621	2.25E-05	5.55E-05	-1.271281541
cg23877385	0.161673941	18.26545502	2.26E-05	5.55E-05	1.32065393

cg24292612	0.121546571	18.24650475	2.28E-05	5.55E-05	1.317944299
cg26043257	0.16244614	18.22166579	2.31E-05	5.55E-05	-1.310639009
cg03775422	0.11146081	18.2003556	2.33E-05	5.55E-05	-1.440394569
cg15926585	0.451358412	18.19359206	2.34E-05	5.55E-05	-1.200005397
cg08088390	0.346248373	18.18602014	2.35E-05	5.55E-05	-1.227776746
cg10140638	0.174831823	18.17861317	2.36E-05	5.55E-05	-1.399454492
cg27196745	0.140073052	18.15879701	2.38E-05	5.55E-05	-1.442451863
cg03046445	0.021363743	18.15342593	2.39E-05	5.55E-05	1.311538693
cg17791651	0.330495035	18.13494861	2.41E-05	5.55E-05	-1.308781537
cg17356252	0.281629745	18.1334174	2.41E-05	5.55E-05	1.241904067
cg13526007	0.11135461	218.12865462	2.42E-05	5.55E-05	-1.377147216
cg23547073	0.074593769	18.04702135	2.52E-05	5.74E-05	1.314537508
cg16924616	0.195460689	18.02383662	2.55E-05	5.80E-05	-1.414134162

cg25221625	0.274680766	17.99672246	2.59E-05	5.87E-05	1.276937715
cg10430690	0.192732383	17.99667962	2.59E-05	5.87E-05	1.343864289
cg23799313	0.107530536	17.96145112	2.63E-05	5.97E-05	1.395257593
cg25242557	0.076991029	17.95410866	2.64E-05	5.99E-05	-1.358647925
cg06476337	0.177424268	17.91327033	2.70E-05	6.12E-05	1.303700329
cg10303842	0.287850901	17.91035685	2.70E-05	6.12E-05	-1.306437354
cg27015047	0.259964084	17.89207595	2.73E-05	6.17E-05	1.227130504
cg09686308	0.429598804	17.89149031	2.73E-05	6.18E-05	1.217155698
cg06456512	0.063401327	17.8655875	2.76E-05	6.25E-05	1.212583186
cg17803089	0.054357533	17.86467635	2.77E-05	6.26E-05	-1.447046931
cg03724463	0.091438159	17.8542065	2.78E-05	6.29E-05	-1.301599491
cg13620770	0.110655909	17.85408575	2.78E-05	6.29E-05	1.200796507
cg20935106	0.053427775	17.82498358	2.82E-05	6.38E-05	1.564005848

cg06637893	0.107077374	17.80268567	2.85E-05	6.44E-05	-1.494560278
cg06778853	0.08335039	17.79846671	2.86E-05	6.46E-05	-1.315582092
cg19636861	0.296263645	17.7940872	2.87E-05	6.47E-05	1.250025382
cg02755525	0.073780883	17.78199882	2.88E-05	6.51E-05	-1.615437428
cg14455487	0.076738588	17.76003732	2.92E-05	6.58E-05	1.269199065
cg22533573	0.057527691	17.7454279	2.94E-05	6.62E-05	2.497788143
cg00233307	0.137660731	17.73096531	2.96E-05	6.67E-05	-1.471817867
cg26143719	0.231266385	17.72740123	2.97E-05	6.68E-05	1.22839529
cg25856383	0.024874091	17.72227234	2.97E-05	6.69E-05	3.741395502
cg09755102	0.05605225	17.71489825	2.98E-05	6.72E-05	1.218597325
cg03742003	0.055330562	17.69756571	3.01E-05	6.77E-05	-1.264564843
cg04517429	0.33291234	17.67566014	3.05E-05	6.85E-05	-1.228382408
cg02775617	0.095848838	17.67333565	3.05E-05	6.85E-05	1.212081848
cg23538064	0.08969376	17.67056452	3.05E-05	6.86E-05	1.588055162
cg01430807	0.030503568	17.66584362	3.06E-05	6.87E-05	

1.211737383

cg02712878 0.162653043 17.65963184 3.07E-05 6.89E-05

1.276052212

cg24794531 0.10480671117.63006957 3.12E-05 6.99E-05 -1.487394137

cg14386312 0.305212203 17.62390656 3.13E-05 7.01E-05

1.220769657

cg27281093 0.028472241 17.61846571 3.14E-05 7.03E-05

2.379045137

cg06263495 0.21498387 17.61804683 3.14E-05 7.03E-05 -1.424084273

cg21271753 0.40757933 17.61520622 3.14E-05 7.04E-05 -1.210291903

cg19461621 0.083679641 17.6115723 3.15E-05 7.05E-05 -1.354085941

cg07826255 0.22620312 17.6076836 3.15E-05 7.06E-05 1.27744709

cg09243900 0.275707266 17.59914901 3.17E-05 7.09E-05

1.352882714

cg10734665 0.30493251 17.56350858 3.22E-05 7.22E-05 1.293449993

cg00987015 0.129371657 17.56254225 3.23E-05 7.22E-05

1.281509374

cg19831575 0.166792524 17.55097762 3.24E-05 7.26E-05 -

1.441445316

cg17439694 0.128682761 17.52873464 3.28E-05 7.33E-05

1.417101094

cg03848675 0.106878419 17.52139064 3.29E-05 7.36E-05 -

1.366657197

cg19486673 0.421535999 17.50853885 3.32E-05 7.41E-05 -

1.237935792

cg16639185 0.189282624 17.441157953.43E-05 7.66E-05 -1.315860082

cg20209009 0.160344509 17.4345036 3.44E-05 7.68E-05 -1.453873559

cg01283289 0.076358183 17.43066468 3.45E-05 7.70E-05 -

1.456402217

cg20557202 0.034302109 17.42563075 3.46E-05 7.71E-05 -

1.490385937

cg21604856 0.372034825 17.39977043 3.51E-05 7.81E-05 -

1.221025571

cg03962522 0.18586031117.38295359 3.54E-05 7.88E-05 1.361536874

cg17108819 0.044756249 17.36123575 3.57E-05 7.96E-05 -

1.562306385

cg07534467 0.034646682 17.35341843 3.59E-05 7.99E-05

1.222639311

cg13271951 0.283378433 17.3532175 3.59E-05 7.99E-05 -1.241237963

cg24454143 0.140486183 17.33972396 3.61E-05 8.04E-05 -

1.265346244

cg20134215 0.307746909 17.33231042 3.63E-05 8.07E-05 -

1.291050389

cg19657082 0.048040976 17.31938923 3.65E-05 8.12E-05

1.200517795

cg18829411 0.140023682 17.3145765 3.66E-05 8.13E-05 -1.314723044

cg15853125 0.064074752 17.31023963 3.67E-05 8.15E-05 -

1.455473592

cg15784615 0.11327728 17.30318854 3.68E-05 8.18E-05 1.44175716

cg10143146 0.397102282 17.30218213 3.68E-05 8.18E-05 -

1.235046631

cg06896207 0.428512083 17.26128096 3.76E-05 8.34E-05 -

1.201326749

cg21740204 0.034537038 17.24369919 3.80E-05 8.41E-05

1.225847019

cg03266453 0.200474016 17.2317838 3.82E-05 8.46E-05 -1.325717028

cg11504740 0.29897971117.22338652 3.84E-05 8.49E-05 1.28898923

cg12998491 0.020630549 17.20456252 3.87E-05 8.57E-05 -

1.586259698

cg16907514 0.070122707 17.19903977 3.88E-05 8.59E-05 -

1.342925831

cg02314308 0.176966666 17.184511373.91E-05 8.65E-05 -1.253040898

cg24695828 0.03215061 17.1842717 3.91E-05 8.65E-05 1.26493039

cg12827555 0.32815934 17.16682048 3.95E-05 8.73E-05 -1.239416539

cg22506059 0.232917068 17.16125562 3.96E-05 8.74E-05

1.247707451

cg22083639	0.074480077	17.14694993	3.99E-05	8.81E-05	-1.378122586
cg23440155	0.092567284	17.14251773	4.00E-05	8.82E-05	1.385495036
cg22830895	0.167792374	17.12041627	4.04E-05	8.91E-05	-1.254746172
cg22737001	0.09911587	17.07888486	4.13E-05	9.10E-05	-1.508639113
cg02938601	0.38914025	17.06320384	4.16E-05	9.17E-05	-1.257152096
cg18565355	0.080454886	17.05931314	4.17E-05	9.18E-05	1.256835997
cg14662379	0.064744491	17.04217833	4.21E-05	9.26E-05	1.240687906
cg24698622	0.063058955	17.03591153	4.22E-05	9.29E-05	1.872169135
cg09503974	0.107874081	17.00679398	4.28E-05	9.42E-05	-1.32709937
cg11812218	0.226287592	17.00068614	4.30E-05	9.44E-05	1.445216589
cg06638433	0.374774618	16.98963335	4.32E-05	9.50E-05	-1.291141657
cg02512226	0.357236345	16.98237712	4.34E-05	9.53E-05	-1.204921667
cg11442717	0.028667439	16.95374884	4.40E-05	9.66E-05	-

1.343706738

cg14951292 0.401820388 16.94179098 4.43E-05 9.72E-05 -

1.220300805

cg22585988 0.218556689 16.93036944 4.46E-05 9.77E-05

1.284154938

cg07807709 0.05721199816.88555844 4.56E-05 9.99E-05 1.512635922

cg21126943 0.4015075 16.85173824 4.64E-05 0.000101475 -

1.21738326

cg12865837 0.379933869 16.8324744 4.68E-05 0.000102369

1.248642762

cg10957151 0.168401273 16.82652738 4.70E-05 0.000102659 -

1.385880022

cg23037133 0.040464355 16.80620019 4.75E-05 0.000103664

1.234708382

cg16678925 0.185882537 16.79501314 4.77E-05 0.000104214

1.473375674

cg24278423 0.081478894 16.76923731 4.84E-05 0.000105575 -

1.527120934

cg17321617 0.094846124 16.76476242 4.85E-05 0.000105771

1.364850673

cg22199080 0.277373418 16.73903251 4.91E-05 0.000107081 -

1.247421142

cg03330058	0.115255858	16.70998803	4.99E-05	0.000108614	1.327010233
cg13629753	0.146409027	16.70885182	4.99E-05	0.000108654	1.274224147
cg01333011	0.421255818	16.70779518	4.99E-05	0.000108689	-1.223866741
cg26531804	0.131938203	16.69210267	5.03E-05	0.000109542	1.235592957
cg07675169	0.18047621	16.67474151	5.08E-05	0.000110448	-1.293912115
cg18602811	0.035255211	16.66716752	5.10E-05	0.000110848	1.288023493
cg21610516	0.0841181	16.66683484	5.10E-05	0.000110848	1.204445289
cg15983538	0.159988461	16.62391763	5.21E-05	0.000113214	1.350706511
cg24901042	0.365657634	16.55603911	5.40E-05	0.000117118	-1.250240441
cg04457979	0.134372048	16.54249218	5.43E-05	0.000117882	-1.445227074
cg22234962	0.497765003	16.53953529	5.44E-05	0.000118036	-1.203682927
cg26160573	0.085865769	16.53788327	5.45E-05	0.00011811	1.571947697
cg16509045	0.125421631	16.53597823	5.45E-05	0.0001182	1.295247317

cg27544190	0.027978503	16.50130722	5.55E-05	0.000120241	1.97721586
cg08748415	0.063350215	16.49474672	5.57E-05	0.00012062	1.229921813
cg23610841	0.095849615	16.48222688	5.60E-05	0.000121274	1.200028022
cg08872742	0.132997962	16.48209791	5.60E-05	0.000121274	1.303803432
cg13517305	0.022191906	16.46235959	5.66E-05	0.000122428	-1.255532767
cg03192551	0.095332046	16.45720549	5.68E-05	0.000122725	-1.365973678
cg26727372	0.13424944	16.43784855	5.73E-05	0.000123869	1.264006889
cg18501026	0.106396151	16.41976474	5.79E-05	0.000124915	-1.3438085
cg18946226	0.321988074	16.41409771	5.80E-05	0.000125252	-1.263065482
cg16158681	0.148960746	16.41287975	5.81E-05	0.000125303	-1.4249653
cg14196790	0.057542673	16.39974473	5.85E-05	0.000126015	1.383279508

cg12069042 0.175623919 16.36989269 5.94E-05 0.000127872

1.308453544

cg19718882 0.034982517 16.36872906 5.94E-05 0.000127898

2.557016329

cg07363637 0.266918357 16.3662909 5.95E-05 0.00012803 1.238120494

cg05592398 0.086308415 16.36241327 5.96E-05 0.000128203 -

1.278378354

cg00128877 0.062167265 16.36168093 5.96E-05 0.000128224

1.200565155

cg12809314 0.067025794 16.360790115.96E-05 0.000128255

1.311090608

cg13565157 0.086938161 16.35640394 5.98E-05 0.000128461 -

1.260207431

cg12555334 0.092805485 16.34577393 6.01E-05 0.000129108

1.357695637

cg22023770 0.053717938 16.33388674 6.05E-05 0.00012987

1.200744276

cg19030554 0.129324292 16.30288856 6.14E-05 0.000131838

1.429860418

cg16377880 0.1110046891 6.30078569 6.15E-05 0.000131928 1.30368604

cg10159529 0.298425412 16.29167639 6.18E-05 0.000132514

1.223058609

cg15746719 0.302189372 16.267291 6.26E-05 0.000134125 -

1.355168096

cg11199399 0.071861234 16.26481269 6.26E-05 0.000134267

1.204995396

cg06497752 0.022844274 16.12670612 6.72E-05 0.000143638 -

1.492164419

cg04375036 0.436135965 16.094417 6.84E-05 0.000145927 -

1.20175655

cg00077877 0.430600786 16.09424271 6.84E-05 0.000145927 -

1.202833842

cg22736323 0.19112105216.08289938 6.88E-05 0.000146686 -

1.286065518

cg25119415 0.49613827 16.08217359 6.88E-05 0.00014671 -1.250095819

cg11170179 0.032875249 16.06592202 6.94E-05 0.000147877

1.480045003

cg13234848 0.069888196 16.06229822 6.95E-05 0.000148121

1.215163143

cg14833385 0.036757513 16.05092877 6.99E-05 0.000148956

2.370935504

cg24908499 0.023760323 16.04569796 7.01E-05 0.000149325 -

1.312497711

cg21926138 0.053666847 16.03510153 7.05E-05 0.000150077 -

1.28597867

cg26055770 0.025989731 16.03033822 7.07E-05 0.000150412

1.418182377

cg06722216 0.037997263 16.01973761 7.10E-05 0.000151138-

1.510761703

cg27398499 0.158570959 16.01804001 7.11E-05 0.000151206

1.273965215

cg09906458 0.224502838 16.00808225 7.15E-05 0.000151917

1.216765137

cg14141399 0.373322544 15.97784931 7.26E-05 0.000154131 -

1.243824065

cg13663218 0.195158526 15.97026308 7.29E-05 0.000154701 -

1.380490984

cg04478795 0.08307018 15.969063117.29E-05 0.000154763 -1.369742857

cg00563926 0.138151391 15.94984702 7.36E-05 0.000156184 -

1.326745547

cg22631938 0.427107332 15.94963631 7.36E-05 0.000156184 -

1.218618772

cg13494498 0.08300208 15.93482402 7.42E-05 0.000157311-1.372112169

cg16175792 0.474142269 15.92879856 7.44E-05 0.000157765

1.204865595

cg02516189 0.12970819 15.9249189 7.46E-05 0.000158013 1.482677697

cg02205739	0.072659393	15.91471091	7.50E-05	0.000158778	-1.350454083
cg08097755	0.08361114	215.90889938	7.52E-05	0.000159219	-1.398064548
cg08085267	0.208040337	15.88420099	7.62E-05	0.000161016	-1.308088703
cg25433648	0.187762442	15.85586041	7.73E-05	0.000163207	1.28339067
cg09158314	0.047076373	15.83544638	7.81E-05	0.000164859	1.93489527
cg05698069	0.070820788	15.83504264	7.81E-05	0.000164859	-1.352602831
cg03945800	0.244560258	15.76127973	8.11E-05	0.000170943	-1.280067026
cg07613278	0.162121898	15.74260363	8.19E-05	0.000172521	1.217593394
cg02096656	0.082947623	15.73352744	8.23E-05	0.000173292	1.305219295
cg08101264	0.45495216	15.72577623	8.26E-05	0.000173947	-1.241163458
cg20322862	0.530181042	15.69557455	8.39E-05	0.000176597	1.201933203

cg20789620 0.247670325 15.6902616 8.41E-05 0.000177018 1.204750219

cg14399060 0.51213702 15.69012445 8.42E-05 0.000177018 1.223408333

cg14467840 0.295970596 15.68528075 8.44E-05 0.000177423 1.259101394

cg04438497 0.124878547 15.676321188.48E-05 0.000178175 1.322869044

cg21663431 0.305167266 15.6762423 8.48E-05 0.000178175 1.230873481

cg02033116 0.031664469 15.66574371 8.52E-05 0.000179065 1.222338036

cg06207460 0.134785629 15.63198267 8.67E-05 0.000182051 1.242332504

cg15540820 0.442374904 15.60812159 8.78E-05 0.000184108 1.238596326

cg05345286 0.11728210515.59212769 8.85E-05 0.000185552 -1.432104338

cg01804429 0.284947685 15.5492262 9.05E-05 0.000189497 -1.202644807

cg13818573 0.076868993 15.54717716 9.06E-05 0.000189618 -1.406799265

cg11263296 0.171076554 15.54221683 9.08E-05 0.000189985 -

1.31343146

cg25853078 0.473389284 15.523911449.17E-05 0.000191743 -

1.200050819

cg27566805 0.438820418 15.49564944 9.30E-05 0.000194432 -

1.218688964

cg14056644 0.44900729 15.49475845 9.31E-05 0.000194481 -

1.212964773

cg10240853 0.039473764 15.48724015 9.34E-05 0.000195195 -

1.341118273

cg20664247 0.195363642 15.48359164 9.36E-05 0.000195522

1.225300366

cg00546897 0.127166086 15.47596463 9.40E-05 0.00019625

1.337493211

cg26599006 0.150617056 15.47327639 9.41E-05 0.000196481 -

1.443904595

cg09001953 0.173522848 15.47134366 9.42E-05 0.000196636 -

1.27663458

cg03805684 0.198919506 15.45628057 9.49E-05 0.000198044

1.309710337

cg13509147 0.09559817 15.45489604 9.50E-05 0.000198144 -

1.308258167

cg23813257	0.079614762	15.4265816	9.64E-05	0.000200805	1.493876245
cg25358289	0.296392734	15.41732964	9.68E-05	0.000201598	-1.25529196
cg09936839	0.254168758	15.411364	9.71E-05	0.000202176	1.258439403
cg07214572	0.025325611	15.39936746	9.77E-05	0.000203303	-1.329419125
cg17412351	0.101330418	15.39280746	9.81E-05	0.000203949	1.252328034
cg20780953	0.027387549	15.38618073	9.84E-05	0.000204605	1.251627402
cg00392257	0.188263086	15.36081005	9.97E-05	0.000207083	1.372337855
cg04447890	0.080684906	15.35921206	9.98E-05	0.00020721	1.257180754
cg18671950	0.039655842	15.34893157	0.000100314	0.000208225	-1.518120748
cg20870559	0.067839348	15.33690882	0.000100938	0.000209476	1.972239471
cg06836849	0.200502762	15.31027315	0.000102333	0.000212203	-1.308470091

cg23187653 0.165268019 15.30981591 0.000102357 0.000212203 -

1.37254237

cg23061578 0.147710235 15.30980794 0.000102357 0.000212203 -

1.316645448

cg23412850 0.442962123 15.237226110.000106259 0.00022002 -

1.266591469

cg27367952 0.085042753 15.17458532 0.000109747 0.000226962

1.206589583

cg06638012 0.043155948 15.17184692 0.000109902 0.000227236 -

1.255603153

cg08026144 0.031970001 15.17018246 0.000109996 0.000227384

1.21613596

cg17204557 0.235252182 15.16479516 0.0001103020.00022797

1.233257071

cg16604516 0.178286097 15.15861015 0.0001106540.000228652 -

1.267580516

cg18886444 0.091950742 15.14391714 0.0001114960.000230249 -

1.420422422

cg16516400 0.252299882 15.13262879 0.0001121470.000231546 -

1.24835235

cg02388150 0.186471386 15.12305919 0.0001127020.000232644 -

1.285809357

cg26580095 0.070870471 15.10614013 0.00011369 0.000234587

1.263412461

cg25214346 0.292072381 15.10447825 0.0001137870.00023474

1.220689935

cg26029902 0.184738743 15.09929801 0.0001140920.00023532

1.480071863

cg11646887 0.01639173 15.0988588 0.0001141180.000235325 1.337054353

cg08843492 0.16317127 15.091119510.0001145740.000236025 1.290322641

cg16761041 0.049637245 15.08316988 0.0001150450.000236898

1.206135862

cg05724065 0.13800661115.07276435 0.0001156640.000238125

1.393193685

cg06493994 0.063306486 15.02462355 0.0001185730.000243716 -

1.413700792

cg13461600 0.039450931 15.00742598 0.0001196310.000245838 -

1.379637793

cg12441928 0.055928894 14.99606956 0.000120334 0.000247233

1.76885724

cg26610808 0.075083434 14.98259321 0.0001211740.00024884 -

1.294516377

cg21472642 0.39406306 14.98233448 0.00012119 0.00024884 -1.214123785

cg14333454 0.153796508 14.96603038 0.000122214 0.00025079

					1.375098457
cg06675478	0.394852399	14.93080035	0.000124457	0.000255237	1.235184191
cg22074858	0.056757178	14.92672033	0.00012472	0.000255723	1.425905688
cg11380624	0.053017044	14.9153276	0.000125456	0.000257075	1.413199065
cg11164400	0.173277054	14.90234993	0.000126299	0.0002586	-1.201894672
cg10515956	0.044648774	14.8935785	0.000126873	0.000259608	-1.208880971
cg17471102	0.118584543	14.88666232	0.000127326	0.000260484	1.237885436
cg25836326	0.164317118	14.88344188	0.000127538	0.000260812	-1.268261086
cg14473924	0.038021601	14.85987536	0.0001291	0.000263792	1.242398315
cg02506908	0.307577376	14.84875061	0.000129844	0.000265151	1.2173842
cg02654291	0.133749965	14.84703355	0.000129959	0.000265332	1.343297451
cg19596755	0.154887645	14.8448294	0.000130107	0.000265581	-

1.324877564

cg06397837 0.056620514 14.84159579 0.000130325 0.000265917 -

1.428619763

cg20264732 0.128188104 14.82862288 0.000131201 0.000267596

1.26582862

cg03332271 0.197137192 14.82085425 0.000131729 0.000268618

1.421730298

cg06504820 0.351388626 14.7936553 0.000133593 0.000272309

1.208498888

cg26960719 0.241941028 14.77834532 0.000134654 0.000274305

1.22479205

cg19751300 0.16337334 14.76336568 0.0001357 0.00027634 -1.355223052

cg10848367 0.086076425 14.74619771 0.00013691 0.000278562

1.297864236

cg11804789 0.31144171814.73520435 0.00013769 0.000280036 -

1.243928643

cg01152019 0.539095916 14.73323181 0.00013783 0.000280209

1.205874577

cg01817029 0.167457722 14.73089279 0.000137997 0.000280378 -

1.361535093

cg22750254 0.027342575 14.72954132 0.000138093 0.000280518 -

1.324081961

cg19014419	0.084529534	14.72613979	0.000138336	0.000280955	1.625782189
cg24989962	0.357182036	14.7247739	0.000138434	0.000281096	-1.292527323
cg16052901	0.16558243	14.71671783	0.000139012	0.000282212	1.390514386
cg16929104	0.088743173	14.71295055	0.000139283	0.000282705	1.23872133
cg22285621	0.048916732	14.71251531	0.000139314	0.000282712	1.919891367
cg16248277	0.18268542	14.70713654	0.000139702	0.000283442	1.277547684
cg02412050	0.032065773	14.69052826	0.000140906	0.000285484	1.223447971
cg15376097	0.052570023	14.67701365	0.000141894	0.000287312	1.23485041
cg23290344	0.482719634	14.63338771	0.000145131	0.00029363	1.227770167
cg20053158	0.049442107	14.59760803	0.000147842	0.000298754	-1.321970864
cg05130485	0.085993654	14.58682316	0.000148669	0.000300185	1.405507209

cg26896762 0.090769131 14.57787293 0.000149359 0.000301517 -

1.386202054

cg16352283 0.278621098 14.57025955 0.000149948 0.000302646 -

1.241723137

cg13958614 0.21467154 14.54388732 0.000152008 0.000306558 -

1.331171727

cg14386691 0.036170786 14.53909064 0.000152385 0.000307197

1.428602793

cg18420965 0.221446147 14.53775498 0.000152491 0.000307348 -

1.374498239

cg09082287 0.21465163 14.50730545 0.000154912 0.000312103 -

1.337912823

cg23114866 0.024563983 14.49869142 0.000155604 0.000313342 -

1.381658947

cg00466249 0.108432945 14.49709664 0.000155733 0.000313506

1.338094785

cg21048669 0.289366322 14.49131608 0.000156199 0.000314319 -

1.221528769

cg09619786 0.049286048 14.48765523 0.000156496 0.000314853 -

1.393206763

cg21905630 0.025724329 14.47394971 0.00015761 0.000316967 -

1.350220977

cg15765212	0.038772789	14.47172852	0.000157791	0.000317268	1.253073308
cg14869028	0.066592764	14.43873541	0.000160509	0.000322347	1.215634028
cg02992632	0.025925593	14.41362178	0.000162609	0.000326435	3.120535295
cg13901526	0.365891426	14.40675558	0.000163188	0.000327532	1.269598689
cg15385623	0.070918833	14.37493685	0.000165899	0.000332841	1.203613648
cg08256781	0.375678679	14.32876748	0.000169914	0.000340556	-1.234075539
cg22880820	0.06601934	14.31704732	0.000170948	0.000342493	1.21379095
cg18769818	0.023261799	14.31219657	0.000171378	0.000343287	3.127749438
cg14672994	0.227344848	14.26537337	0.000175586	0.000351367	-1.237405698
cg19254558	0.033854509	14.26003206	0.000176073	0.000352271	1.224080242
cg19093820	0.070812767	14.25505588	0.000176528	0.00035304	1.30828862

cg13297865 0.041394513 14.24694334 0.000177271 0.000354386 -

1.623487743

cg10908369 0.039073197 14.22871428 0.000178954 0.000357608 -

1.452595067

cg09872616 0.159879516 14.2114325 0.000180564 0.000360396

1.41650724

cg21057046 0.166149962 14.21039147 0.000180661 0.00036052 -

1.212759617

cg20673481 0.034687475 14.20193238 0.000181455 0.00036196 -

1.33004048

cg19292008 0.083300172 14.18838009 0.000182734 0.000364176 -

1.4424634

cg05602212 0.031849807 14.17943951 0.000183583 0.000365626

1.210110212

cg14865868 0.11257775514.17768414 0.00018375 0.000365887

1.514011724

cg14313310 0.13145466 14.17387097 0.0001841140.000366538 -

1.252060656

cg21137823 0.044886991 14.17273209 0.000184222 0.00036661 -

1.463621236

cg18459342 0.12921598 14.16049255 0.000185395 0.000368725

1.269912687

cg18219951 0.075322838 14.14833297 0.000186568 0.000370983

1.555837998

cg01580681 0.443755768 14.13928391 0.000187445 0.000372581

1.213280003

cg16792632 0.031413932 14.13691412 0.000187675 0.000372966 -

1.546728887

cg09014354 0.138491894 14.13558037 0.000187805 0.00037315 -

1.266625886

cg04837898 0.194075339 14.1320931 0.000188145 0.000373752

1.266292011

cg20847746 0.161524872 14.13016522 0.000188333 0.000374052

1.281813097

cg09276883 0.053661852 14.12369005 0.000188967 0.000375113-

1.283872534

cg12340144 0.097460131 14.116885350.000189635 0.000376191 -

1.288202836

cg09424308 0.167693082 14.115710780.00018975 0.000376346

1.271411705

cg04956511 0.130454941 14.115171660.000189803 0.000376378

1.298621377

cg01200177 0.106203473 14.10936618 0.000190376 0.000377438 -

1.23295684

cg21187265	0.055163333	14.09340091	0.000191958	0.000380202	1.23839568
cg23886551	0.083119372	14.05836122	0.000195479	0.000387023	-1.465212893
cg03837750	0.110083581	14.04469352	0.00019687	0.000389551	1.21703181
cg20595215	0.036736776	14.04263671	0.00019708	0.000389886	1.223249281
cg25938646	0.107521279	14.0416856	0.000197177	0.000390002	-1.447382896
cg02175308	0.066996996	14.01996773	0.000199411	0.000394267	1.227975919
cg25993718	0.234049087	13.99857196	0.000201638	0.000398355	1.329886005
cg11750183	0.056791952	13.99434401	0.00020208	0.000399132	1.288520547
cg27223047	0.169627123	13.9940612	0.00020211	0.000399132	-1.324849858
cg02214188	0.337127934	13.99024441	0.000202511	0.000399767	1.205071576
cg19789466	0.028551051	13.98872347	0.000202671	0.000400004	1.926333336
cg22968401	0.090717235	13.97762748	0.000203841	0.000402235	

1.243857586

cg27177839 0.27280181113.97596958 0.000204017 0.000402424 -

1.284236987

cg00554702 0.11499133213.96862338 0.000204796 0.000403803

1.221637418

cg01603095 0.281284132 13.95597842 0.000206144 0.000406223 -

1.235327389

cg06339706 0.227541034 13.95050648 0.000206731 0.000407299

1.227445211

cg00547018 0.131064603 13.9474938 0.000207054 0.000407777

1.256022165

cg02553486 0.19035458 13.94397561 0.000207433 0.000408443 -

1.250067529

cg11484872 0.205661345 13.91262817 0.000210836 0.0004149

1.327307091

cg07654934 0.045968262 13.89970074 0.000212255 0.000417467

2.478872029

cg13619408 0.143139927 13.89572738 0.000212694 0.00041823 -

1.273720892

cg10883303 0.31482981113.89470497 0.000212807 0.00041837 -

1.334388224

cg03732545 0.152772869 13.86832191 0.000215742 0.000423893 -

1.203968404

cg05270634 0.124319424 13.856898 0.000217026 0.000426332 -

1.36887786

cg17826679 0.245578125 13.80892774 0.000222501 0.000436322

1.228977998

cg13812587 0.016702752 13.799611670.00022358 0.000438353 -

1.253846222

cg15387123 0.05770679 13.79890439 0.000223662 0.000438429

1.240031109

cg01062029 0.11095250613.795113170.000224103 0.000439208 -

1.342839059

cg02932669 0.172042838 13.76595095 0.000227525 0.000445307 -

1.279998756

cg09747578 0.090796051 13.75786954 0.000228482 0.000447007

2.079880874

cg05898102 0.037943912 13.74059454 0.000230542 0.000450862

2.457110055

cg23967169 0.11702052613.72216658 0.000232761 0.000454672 -

1.416094671

cg23748737 0.026622814 13.71398882 0.000233752 0.00045652 -

1.304658304

cg09827833 0.315866355 13.67281531 0.000238809 0.000465819 -

1.221119473

cg06983551 0.100754194 13.6256329 0.00024474 0.000476593

1.226550259

cg03292149 0.22052621 13.61298222 0.000246355 0.000479385

1.234724896

cg01351032 0.131790243 13.61291397 0.000246364 0.000479385

1.283445791

cg26514492 0.291856973 13.603273 0.000247602 0.000481608

1.2717676

cg03103192 0.235657107 13.60151342 0.000247829 0.000481956

1.250294396

cg20070090 0.12884311713.59750234 0.000248346 0.000482776

1.296971091

cg14289461 0.140000874 13.58820084 0.000249551 0.00048484

1.283379304

cg04809787 0.061268984 13.56068998 0.000253147 0.000491446 -

1.304868621

cg17901463 0.206536793 13.54827064 0.000254788 0.000494536

1.420561091

cg26667975 0.02490795 13.5444174 0.000255299 0.000495051 -

1.285932113

cg06533629 0.126192622 13.54399735 0.000255355 0.000495064 -

1.363193716

cg14634738 0.037770882 13.54141749 0.000255698 0.000495443 -

1.237160999

cg09089053 0.250881917 13.53399368 0.000256688 0.000497169

1.228532871

cg03565323 0.079736559 13.50090929 0.0002611450.000505413 -

1.31927629

cg11063110 0.363931365 13.49314725 0.000262202 0.000507264 -

1.20197755

cg23214764 0.043121298 13.48200631 0.000263726 0.000510018 -

1.2613969

cg05937453 0.067922584 13.46204741 0.00026648 0.000514948 -

1.502809798

cg26894575 0.068376609 13.44001505 0.000269554 0.000520289

1.647394893

cg08558340 0.448082368 13.43625108 0.000270083 0.00052121 -

1.218575258

cg15050111 0.02423213 13.425068 0.00027166 0.000524052 1.208128281

cg11885098 0.21795991 13.3947811 0.000275977 0.000532076 -

1.215365362

cg24860534 0.166467576 13.37220678 0.000279241 0.000537956 -

1.219089927

cg12024906 0.108295015 13.36738357 0.000279943 0.000539206

1.51461601

cg01479232 0.136994599 13.36143471 0.000280812 0.000540775

1.342666743

cg11330108 0.079964625 13.3587854 0.0002811990.000541419

1.234225585

cg17389519 0.28711883613.35133945 0.000282292 0.000543418

1.215281467

cg25112191 0.156532471 13.35007096 0.000282479 0.000543674

1.248836321

cg02310296 0.173365842 13.32416457 0.000286316 0.000550744 -

1.270662811

cg05671350 0.072556883 13.31299333 0.000287987 0.00055343 -

1.283509956

cg16080552 0.065590728 13.30765944 0.000288789 0.000554865

1.374930937

cg06200697 0.182396291 13.28654973 0.000291982 0.00056068

1.423350616

cg07845392 0.277001585 13.25820348 0.000296327 0.000568157 -

1.251173745

cg06425556 0.021381595 13.24773222 0.000297949 0.000570831

1.205932552

cg12476968 0.127875656 13.24436314 0.000298472 0.000571726

1.246528279

cg06908778 0.454544196 13.23973708 0.000299193 0.000572888

1.200672766

cg14370448 0.11815751213.231106210.000300542 0.000575252

1.446094879

cg27352992 0.08553957 13.216811830.000302789 0.000579444 -

1.429340381

cg03945021 0.076916739 13.2050288 0.000304655 0.000582793

1.500336344

cg07633499 0.11759762513.19780176 0.000305805 0.00058466

1.287150529

cg02780849 0.235642629 13.18074358 0.000308537 0.000589771 -

1.243368035

cg27105123 0.051965128 13.17526219 0.00030942 0.000591234

1.211726049

cg26366091 0.231401283 13.15807105 0.000312206 0.000595992

1.331591418

cg15639045 0.020148948 13.15003464 0.000313517 0.000598269 -

1.20566644

cg14163776 0.153607721 13.1410255 0.000314993 0.000600858 -

1.239882187

cg09022993 0.137752225 13.12988849 0.000316828 0.00060413

1.635828225

cg22791453 0.083909166 13.12017909 0.000318436 0.000607082

1.303859965

cg21576698 0.213802021 13.114347780.000319406 0.000608816

1.558425361

cg06953304 0.081491039 13.09786136 0.000322165 0.000613725

1.214595721

cg21604803 0.258061003 13.09376489 0.000322854 0.000614922 -

1.374598884

cg20895877 0.11536187513.03359056 0.000333151 0.000633695

1.259102043

cg25167643 0.089148314 13.0200623 0.0003355110.000637824 -

1.516638044

cg05881135 0.068540592 13.00565191 0.000338044 0.000642033 -

1.461410749

cg16990174 0.155120985 12.99859561 0.000339291 0.000644281

1.214625927

cg21360828 0.062161928 12.99740843 0.000339502 0.000644437 -

1.409321

cg19674669 0.065797816 12.99408514 0.000340091 0.000645434 -

1.373119283

cg23656386	0.125898034	12.98618558	0.000341496	0.000647979	1.291269633
cg09721427	0.1286214	12.98210921	0.000342224	0.000649237	-1.253378136
cg27319898	0.05699489	12.97817008	0.000342928	0.000650451	-1.37437744
cg09017174	0.068827064	12.95825175	0.000346513	0.000657003	1.395203853
cg16431978	0.277883032	12.94202875	0.00034946	0.000662466	1.265112537
cg02748539	0.129378024	12.93976107	0.000349874	0.000663127	-1.359867843
cg05098732	0.077302203	12.92591403	0.000352413	0.000667687	-1.288951745
cg14384532	0.121261422	12.90868874	0.000355597	0.000673341	-1.415474062
cg17031727	0.191673733	12.90335261	0.00035659	0.000675093	-1.330057029
cg11896923	0.213920006	12.90161389	0.000356914	0.000675452	1.210082576
cg22947000	0.118333112	12.89500249	0.000358148	0.000677661	1.214419011

cg06537829 0.11793215312.88625219 0.000359789 0.00068051

1.772333449

cg23904249 0.343405934 12.88220169 0.000360551 0.000681695 -

1.206320329

cg24686358 0.036999067 12.87970598 0.000361021 0.0006822 -

1.396282428

cg22821324 0.070362294 12.87649068 0.000361628 0.000683091 -

1.479110333

cg05158615 0.257049543 12.87388568 0.00036212 0.000683893 -

1.323578038

cg07713361 0.11632277512.86780905 0.000363272 0.000685938

1.310391068

cg23704082 0.01469931112.85816874 0.000365106 0.000689272

4.589912315

cg13877915 0.063538581 12.84963847 0.000366736 0.000692091

2.20439613

cg17651821 0.25958183 12.84769514 0.000367109 0.000692665 -

1.347526653

cg03929796 0.330068231 12.84398416 0.000367821 0.000693879 -

1.213853307

cg18818531 0.321945204 12.816117250.000373216 0.000703529 -

1.216253456

cg20451680 0.338675373 12.80650702 0.000375095 0.000706807 -
1.200159611

cg01125463 0.070476468 12.79324261 0.000377704 0.000711324
1.440493452

cg17808849 0.033437476 12.79234655 0.000377881 0.000711524-
1.203356955

cg15979932 0.171817984 12.76890279 0.00038254 0.000719758
1.335119686

cg02376163 0.039364381 12.76454557 0.000383412 0.000721102
1.229612038

cg15582789 0.053729422 12.73891522 0.000388584 0.000730216
1.220955773

cg12374431 0.047547899 12.738691190.000388629 0.000730216 -
1.382358869

cg24829483 0.214109476 12.721146740.00039221 0.000736578
1.228627488

cg18992688 0.435350275 12.71807642 0.00039284 0.000737624 -
1.220227166

cg06742978 0.024814236 12.7165765 0.000393149 0.000737927 -
1.238351078

cg07104706 0.26177103 12.711698320.000394153 0.000739674 -
1.333280735

cg17084151 0.126397338 12.69932953 0.00039671 0.000743643

1.283411844

cg19914607 0.053875592 12.69149129 0.00039834 0.000746419 -

1.222415998

cg22244122 0.15571074 12.68834669 0.000398995 0.00074737 -

1.234317115

cg24625388 0.205833744 12.68367174 0.000399972 0.00074906 -

1.219866234

cg08804892 0.170748616 12.68158196 0.000400409 0.00074974

1.448804116

cg09072120 0.016867313 12.68017736 0.000400704 0.000750151 -

1.252837536

cg06269753 0.160951494 12.67272481 0.000402268 0.000752941 -

1.301635048

cg10725344 0.16756354 12.66210354 0.000404509 0.000756573

1.321376504

cg03082060 0.30589119212.63440621 0.000410413 0.000767072

1.20085822

cg05483509 0.068662783 12.61922155 0.000413686 0.000773019 -

1.397250421

cg20401945 0.318761852 12.61237038 0.000415171 0.000775651

1.232157676

cg20275133	0.071788282	12.5992162	0.000418039	0.000780429	1.390655867
cg07642638	0.099859817	12.57202497	0.00042403	0.000790735	-1.347682085
cg15242570	0.04953956	12.54661432	0.000429707	0.000800432	1.278890927
cg12024292	0.111014322	12.53309406	0.000432759	0.00080567	-1.369896731
cg07747970	0.052375	12.52721924	0.000434092	0.000808003	-1.367363014
cg22325703	0.052750913	12.51627904	0.000436585	0.000812494	-1.44387704
cg25999015	0.044509484	12.51431597	0.000437034	0.000813179	-1.369437797
cg08460435	0.151377376	12.49953234	0.00044043	0.000819195	-1.327857832
cg00834796	0.132161082	12.49420278	0.000441661	0.000821181	-1.251441236
cg26209676	0.048621008	12.48923094	0.000442812	0.000822645	1.437234327
cg20876010	0.190264163	12.48911791	0.000442838	0.000822645	1.233150934

cg18515587 0.129979909 12.48496976 0.000443801 0.000823944

1.32280366

cg19639622 0.021690431 12.48375944 0.000444082 0.000824315 -

1.272254309

cg24596472 0.070742975 12.47505765 0.000446111 0.000827775

1.333278942

cg26672426 0.139890036 12.47002709 0.000447287 0.000829805

1.297599135

cg18636558 0.162774384 12.46276914 0.000448991 0.000832812

1.235401651

cg04270799 0.123860743 12.46174751 0.000449231 0.000833104 -

1.355168828

cg19744952 0.094675615 12.46066541 0.000449486 0.000833423

1.201432236

cg12894126 0.13228282 12.45421302 0.000451007 0.000836091

1.206063639

cg26304237 0.12244041112.42275056 0.000458501 0.000849358 -

1.400377027

cg19008809 0.046607716 12.39640129 0.000464875 0.000860374 -

1.473443833

cg06953768 0.137772913 12.38873779 0.000466745 0.000863201

1.25518393

cg01888566 0.137312912 12.36818992 0.000471798 0.000871905

1.394910093

cg05249393 0.04970679 12.34681783 0.0004771120.000880822

1.732629617

cg17966192 0.372955385 12.34667517 0.000477147 0.000880822 -

1.209424432

cg25084878 0.16518402 12.3364493 0.000479712 0.00088507 1.214666733

cg04528819 0.328513927 12.32122018 0.000483556 0.000891745 -

1.308451393

cg07027075 0.061480931 12.32106683 0.000483595 0.000891745

1.210864111

cg26884581 0.324896357 12.30150862 0.000488579 0.000900599 -

1.226433464

cg16632715 0.076226635 12.29602675 0.000489985 0.000902867 -

1.339234

cg22077101 0.121788029 12.29220465 0.000490968 0.000904513

1.290999538

cg14896516 0.157577281 12.281138180.000493825 0.000909444 -

1.385737927

cg21949305 0.192307243 12.27767181 0.000494723 0.000910932

1.265969637

cg18110483 0.06882199 12.25836576 0.000499757 0.000919193 -

1.415689746

cg18804206 0.11674782812.24185487 0.000504104 0.000926848

1.223205999

cg24719984 0.172572653 12.20596934 0.000513682 0.000943599 -

1.301473031

cg04330084 0.100819548 12.20187392 0.000514787 0.000945456

1.262782333

cg26133068 0.095369258 12.20034825 0.000515199 0.000945868 -

1.2610603

cg22753768 0.058497154 12.19718396 0.000516055 0.000947095

1.315037274

cg18464137 0.247847749 12.16552377 0.000524699 0.000962258 -

1.286936704

cg23557926 0.186686656 12.16333754 0.000525302 0.000963187

1.241978682

cg22264436 0.179428846 12.16034582 0.000526127 0.000964525

1.23907079

cg23918047 0.0687434 12.15309746 0.000528132 0.000967848

1.482546416

cg06834875 0.133721208 12.13165524 0.000534109 0.000977912 -

1.236711961

cg03544320 0.093151745 12.12410901 0.000536229 0.000981258

1.625247689

cg08367838 0.129477923 12.12115369 0.000537061 0.000982603

1.260230372

cg04273431 0.277734382 12.11865147 0.000537767 0.000983716 -

1.256486042

cg08124030 0.095121758 12.1159902 0.000538519 0.000984733

1.550472328

cg04645342 0.066594571 12.09572414 0.000544278 0.000994363 -

1.358666437

cg18508125 0.061963435 12.06929349 0.000551884 0.00100771

1.209161216

cg26557658 0.30844561 12.06840091 0.000552143 0.001008 -

1.211663922

cg05257610 0.198946345 12.06597343 0.000552847 0.001009102

1.202586417

cg00520708 0.230964812 12.0532145 0.000556563 0.001015165

1.211619117

cg11354906 0.100686828 12.03320083 0.000562444 0.001025691 -

1.41810638

cg18847227 0.299048364 12.02155674 0.000565894 0.001031796 -

1.239630312

cg08287471 0.061864076 12.01147453 0.000568899 0.001037087

1.212092028

cg04095468 0.11457715711.981769550.000577847 0.001051876

1.314699042

cg08596000 0.076726026 11.965655070.00058276 0.001060628 -

1.254796779

cg03190825 0.088400949 11.957454120.000585277 0.001064134

1.315200477

cg05369166 0.059707639 11.955154520.000585984 0.001064802 -

1.281278656

cg25085950 0.077969658 11.953962160.000586352 0.001065241

1.223458383

cg20587394 0.11336518 11.952728360.000586732 0.001065739 -

1.362210243

cg08242493 0.017128803 11.934005420.000592534 0.001074534

1.211213954

cg12800028 0.11727986411.917066940.000597832 0.001082974 -

1.273870373

cg02196730 0.033952099 11.905445610.000601496 0.001089218 -

1.275948601

cg22282672 0.087572938 11.894062360.000605106 0.001095361

1.222346146

cg08598221 0.169937836 11.885723980.000607764 0.001099779 -

1.222795329

cg06171787 0.122558287 11.869254090.00061305 0.001108348-1.298306703

cg26798786 0.03221617 11.8590672 0.000616342 0.0011141011.221251185

cg25882366 0.102513712 11.845512480.000620751 0.001121618

1.271374102

cg26781575 0.035045284 11.845255340.000620835 0.001121618-

1.288085921

cg15059932 0.034006634 11.840300630.000622455 0.00112394

1.913008421

cg05920090 0.035248361 11.827957750.000626509 0.00113045

2.411423313

cg10183248 0.023329178 11.823065130.000628123 0.001133159

1.774143192

cg02192520 0.086332422 11.822066330.000628453 0.001133467

1.496939311

cg12876594 0.23250301 11.821868990.000628518 0.0011334671.283371205

cg25370441 0.260047733 11.8029877 0.000634792 0.001143143

1.260526081

cg09871043 0.196212339 11.764666990.00064772 0.0011649661.250332523

cg18107072 0.142454937 11.759971630.000649322 0.001167431-

1.284905839

cg06459327 0.067627384 11.757675690.000650107 0.001168634

1.307405758

cg03763616 0.260453997 11.739248040.000656442 0.001179601-

1.293080115

cg11816577 0.039414052 11.725979780.000661042 0.001187232

1.247039279

cg03557733 0.07211993411.723606370.000661868 0.00118808 -1.413219925

cg10482024 0.035026564 11.720599450.000662916 0.001189616-1.2349315

cg15982419 0.089426534 11.701556850.000669595 0.001200666 -

1.272481752

cg20163288 0.017224019 11.672374990.000679961 0.001218171 -

1.229679883

cg22585269 0.085468544 11.669917410.000680842 0.001219315

1.209745743

cg13084525 0.08702116311.669219170.000681092 0.001219546

1.342890834

cg21238818 0.425558308 11.657427630.000685334 0.00122627 -

1.23732953

cg11919694 0.029378739 11.655527450.00068602 0.00122728 1.524731687

cg11393848 0.094925634 11.629034590.000695659 0.001243198

1.33163025

cg23303408 0.156433391 11.624440310.000697344 0.001245768 -

1.342224625

cg26385222 0.204633014 11.623796870.00069758 0.00124597 -1.261465803

cg05221167 0.261214853 11.590940350.000709759 0.001265927

1.407227702

cg21573601 0.01166552611.586594090.0007113860.001268604 -1.214276736

cg05358404 0.464839202 11.575082310.000715714 0.001275645 -

1.214658008

cg03538436 0.098125143 11.565113160.000719483 0.001282136 -

1.371514969

cg15227982 0.050051788 11.5547853 0.000723409 0.001288677

1.45543193

cg02189785 0.03892226 11.541658230.000728431 0.001296935

1.211655744

cg13704548 0.095261327 11.538537380.00072963 0.0012986111.271726424

cg14419187 0.19493982 11.5224527 0.000735842 0.001308973 -

1.343457104

cg09495977 0.086996586 11.518038770.000737556 0.00131179 -

1.403068899

cg01311051 0.148265388 11.503819350.000743105 0.001320494

1.228231631

cg14671488 0.075533003 11.483840010.000750973 0.001333345

1.247854683

cg02071074 0.09911478711.4834429 0.0007511310.001333345 -1.242947921

cg04084157	0.094711328	11.4793684	0.000752746	0.001335977	-1.251582182
cg08578641	0.33303055	11.46914702	0.000756814	0.00134296	-1.210885296
cg26954174	0.132646997	11.45570219	0.000762198	0.001351827	1.416475748
cg27234090	0.100148926	11.44967418	0.000764625	0.001355389	-1.250096847
cg02361557	0.029052472	11.41084	0.000780448	0.00138125	-1.452749648
cg09558502	0.074332601	11.39756305	0.000785933	0.001390469	1.332801386
cg04893119	0.390656262	11.37861772	0.000793829	0.00140348	1.254534903
cg26256793	0.311884957	11.36290109	0.000800439	0.001413899	-1.236736116
cg15543551	0.059021975	11.35340981	0.000804459	0.001420251	2.046125937
cg24206256	0.087484093	11.33706998	0.000811426	0.00143205	1.516113549
cg12000587	0.056856984	11.32305756	0.000817449	0.001441923	-1.220710608
cg04689061	0.27942779	11.3034659	0.000825947	0.001455384	-1.213620152

cg08411435 0.123591285 11.29498918 0.000829652 0.001461656 1.207076121

cg03188118 0.190936215 11.291484 0.000831189 0.001464108 1.20453844

cg08186362 0.040468411 11.28338096 0.000834753 0.001469614 -1.524859734

cg03214212 0.047644391 11.27844005 0.000836933 0.001473196 1.20096429

cg20948472 0.055414088 11.27556367 0.000838205 0.001475178 1.316591038

cg14870271 0.060086497 11.26732839 0.000841858 0.001481089 1.224235349

cg22994720 0.064454252 11.25839609 0.000845839 0.001487572 -1.360902206

cg24662718 0.071218376 11.25606362 0.000846881 0.001489146 -1.413626848

cg10604333 0.132477382 11.25305826 0.000848226 0.001490991 -1.359826357

cg03506489 0.076140012 11.23218787 0.000857628 0.001506466 -1.424712994

cg22970435 0.296296719 11.21424418 0.000865796 0.001520018 -1.231448735

cg05874450 0.085062222 11.20356271 0.000870695 0.001527023 -

1.258596898

cg08548888 0.064805745 11.191489280.000876267 0.00153575

1.526783362

cg22319147 0.153865644 11.187330410.000878195 0.001538836

1.316817618

cg00431549 0.244018756 11.174499260.000884169 0.001548766

1.298114732

cg01441777 0.091707726 11.167394750.000887495 0.001554051

1.476537577

cg09988116 0.204967771 11.167037840.000887662 0.001554075

1.225450501

cg02589695 0.21447586 11.165500050.000888384 0.001555068

1.211357679

cg02064106 0.052243694 11.151008310.000895214 0.001565663 -

1.261418521

cg01214847 0.11026067511.149076910.000896128 0.001566913

1.284733105

cg01986577 0.070609855 11.129573380.000905414 0.001582404 -

1.257072444

cg02838492 0.207143568 11.1085514 0.000915533 0.001598979

1.224759585

cg19776201 0.060731206 11.103018090.000918215 0.00160283

1.910563186

cg25985103 0.109505 11.083622850.00092768 0.0016185111.253923895

cg21604042 0.05995871111.083160940.000927906 0.001618626 -

1.454472976

cg04335339 0.095156173 11.042616060.000948019 0.001651309

1.20388664

cg20131596 0.230997363 11.042418090.0009481180.001651309

1.217252389

cg16120811 0.056906098 11.041571190.000948543 0.001651763 -

1.500511617

cg19103704 0.20090635 11.038948690.000949859 0.001653771

1.295564219

cg10539507 0.084762407 11.036438720.0009511210.001655682 -

1.363693984

cg15319457 0.020408692 11.0172967 0.000960802 0.001671667 -

1.281576241

cg09954385 0.027540147 11.015791650.000961567 0.001672421

1.213137624

cg23983173 0.136672951 11.014467620.000962241 0.001673243

1.216784276

cg27305895 0.05905691 11.014210560.000962372 0.001673243 1.24798919

cg12603560 0.144990945 11.008667660.000965198 0.001677867

1.217456542

cg02630207 0.108525548 11.005489820.000966823 0.001680401

1.378875587

cg13218435 0.02792458 11.002159910.000968528 0.001682784 -

1.253763453

cg03954587 0.195844415 10.98455432 0.000977593 0.001697363

1.449490401

cg24499839 0.032831977 10.979928 0.000979989 0.001700937 -

1.239028368

cg05436231 0.064257772 10.95131549 0.000994943 0.001725703

1.407343382

cg06379754 0.029542652 10.94991277 0.000995682 0.001726687 -

1.280995177

cg18722841 0.247423221 10.94073669 0.00100053 0.001734498 -

1.314238145

cg17251713 0.242898795 10.9303396 0.001006052 0.001743696

1.339180007

cg03243946 0.05110534510.89313445 0.001026067 0.00177663 -

1.230739401

cg25023994 0.29213116710.88336951 0.001031387 0.001785227

1.302495201

cg00970325 0.093190987 10.88301062 0.001031583 0.00178526 -

1.396633848

cg04880063 0.054385091 10.8745587 0.0010362110.001792961 -

1.382097823

cg12613383 0.218634684 10.87221689 0.001037497 0.00179457 -

1.248088864

cg20492933 0.061259133 10.856117040.001046383 0.001808295

1.28901518

cg13745870 0.058171717 10.85588998 0.001046509 0.001808295

1.220005276

cg27138584 0.12521947 10.84500382 0.001052561 0.001817506 -

1.265261949

cg16188243 0.088557575 10.84298667 0.001053686 0.001818514

1.257080681

cg03876618 0.068586576 10.82852554 0.00106179 0.001831246 -

1.319178563

cg09309269 0.101632709 10.80860912 0.001073055 0.001849724 -

1.251716026

cg26898166 0.091850871 10.77728755 0.001091016 0.001878617 -

1.24707622

cg08911391 0.090329805 10.77710444 0.0010911220.001878617 -

1.253529226

cg03633120 0.052975081 10.76897598 0.001095833 0.001886084

1.4254833

cg04748704 0.102031526 10.76553924 0.001097831 0.001888903

1.418100114

cg00211661 0.016655827 10.75824169 0.0011020870.001895552

2.856309933

cg06656924 0.176585694 10.75348235 0.0011048710.001899692

1.201702496

cg08392591 0.062487646 10.74783422 0.0011081840.001904414

1.265440084

cg14039952 0.038789746 10.72934567 0.0011191010.001921863

1.303668574

cg23090824 0.167426615 10.71396933 0.0011282630.001935948

1.252846337

cg19995014 0.021427083 10.7006048 0.0011362880.001949054 -

1.270229845

cg02867079 0.060950636 10.69715447 0.0011383690.001952292 -

1.330606336

cg13258700 0.049350261 10.68347375 0.00114666 0.001964503 -

1.33754923

cg24697184 0.027653264 10.67674183 0.0011507620.001971196

1.900290603

cg04785227 0.045365087 10.66717645 0.0011566160.001979877 -

1.364170883

cg00029826 0.107548505 10.63839858 0.001174411 0.002008974

1.242009444

cg18592174 0.170052391 10.63376921 0.0011773 0.002012207 -

1.286715519

cg19579782 0.175688207 10.62666375 0.001181747 0.002019123

1.227908945

cg23003881 0.08059089 10.59920118 0.001199098 0.002046686 1.356431224

cg05389335 0.126407197 10.5865187 0.001207197 0.00205807 -

1.343218752

cg10784030 0.292701697 10.57695018 0.001213345 0.002067502 -

1.283527763

cg27329371 0.173204249 10.57137291 0.001216943 0.002072581

1.201268461

cg20977864 0.119280136 10.57050065 0.001217507 0.002073191

1.261400222

cg22807823 0.076863075 10.56604417 0.001220391 0.0020774

1.259032346

cg09134747 0.146676906 10.56301789 0.001222353 0.002080389

1.216529588

cg21838334 0.097494292 10.53789411 0.00123877 0.002105839 -

1.381006884

cg25422943	0.014022184	10.53030936	0.00124377	0.002113269	-1.428930781
cg05855071	0.075286812	10.52971375	0.001244163	0.002113581	1.307088261
cg05158538	0.175224896	10.50657285	0.00125955	0.002137557	1.21746265
cg09214551	0.25464034	10.50567466	0.001260151	0.002138217	1.207615786
cg14844130	0.208118439	10.4905155	0.00127034	0.002154687	1.305962102
cg03682712	0.06788755	10.46704574	0.00128628	0.002180349	1.318018674
cg25854162	0.043001252	10.46597974	0.001287009	0.002181218	-1.305047515
cg01919208	0.124082976	10.46388355	0.001288443	0.002182914	1.26876283
cg09109450	0.081118838	10.44969645	0.001298193	0.002197586	1.662256852
cg07621046	0.213766684	10.44626657	0.001300562	0.002201225	-1.262227856
cg08009622	0.178249865	10.43934091	0.001305358	0.002208972	-1.335059469
cg25372693	0.056785427	10.43248201	0.001310125	0.002216295	

1.353284217

cg27420236 0.073518955 10.42990614 0.00131192 0.002218958 -

1.46236406

cg21869532 0.067047908 10.42382951 0.001316164 0.00222539

1.210125548

cg00888007 0.058824234 10.41727224 0.00132076 0.002232411-

1.420269662

cg02581667 0.022712732 10.41679506 0.001321095 0.002232603 -

1.218334615

cg00410898 0.160390333 10.41594073 0.001321695 0.002233243

1.242455964

cg06567342 0.08353455 10.41029847 0.001325665 0.0022392

1.200086519

cg08301503 0.057069276 10.40682784 0.0013281140.00224219

1.219034733

cg21908259 0.096518771 10.40236282 0.00133127 0.002246407

1.909700006

cg03663715 0.169732461 10.37844017 0.0013483110.002271739 -

1.312925748

cg03285457 0.083977707 10.36628992 0.001357051 0.002284936 -

1.394836294

cg04949741 0.120006894 10.3649463 0.001358021 0.002286188

1.266867504

cg04065065 0.140009524 10.36448978 0.001358351 0.002286361

1.266886478

cg25511807 0.091336403 10.3638979 0.001358779 0.002286698

1.253260667

cg20134151 0.047596347 10.35175043 0.001367586 0.002299599 -

1.240990088

cg08775230 0.122656922 10.35006529 0.001368812 0.002301277

1.23206176

cg07115304 0.026291257 10.34806786 0.001370267 0.002302955 -

1.210547354

cg27554782 0.073800258 10.33310037 0.00138122 0.002319815

1.209015315

cg22084611 0.104626612 10.33000938 0.001383493 0.002323245

1.202519332

cg09542111 0.11586993310.27393841 0.001425391 0.002388031 -

1.375168997

cg06491116 0.039653535 10.26250534 0.001434091 0.00240101

1.208748198

cg05065037 0.030959539 10.25122274 0.001442729 0.002414269

1.240751788

cg26806924 0.089795639 10.23775228 0.0014531120.002428417

1.351031196

cg10481740 0.027240196 10.21594265 0.001470084 0.002452306

1.307554007

cg04561804 0.073252559 10.20592728 0.001477945 0.002464603 -

1.217084185

cg00775197 0.055324416 10.20375629 0.001479655 0.002467046 -

1.331873955

cg12420104 0.092543124 10.171110560.001505607 0.002507826 -

1.380042548

cg15536490 0.036178029 10.16772964 0.001508321 0.002511932

1.231076734

cg07505695 0.05015293 10.16685428 0.001509024 0.002512574

1.20619197

cg18221862 0.068746931 10.165888110.001509801 0.002513151 -

1.348006705

cg12513379 0.13012308 10.15239356 0.001520694 0.002530029 -

1.244992409

cg25720804 0.278391677 10.14863406 0.001523743 0.002534683 -

1.32098063

cg26045434 0.071244855 10.13066726 0.0015384 0.002557375 -

1.224102629

cg11882252 0.257821493 10.10007973 0.001563683 0.002595332

1.411100513

cg16141690 0.069180181 10.097927110.001565478 0.002597674

1.289467089

cg01425670 0.015636415 10.08409231 0.001577066 0.002613888 -

1.415449223

cg24713204 0.086944074 10.06705471 0.001591455 0.002634272

1.366691993

cg20507276 0.206559587 10.05905487 0.001598258 0.002644312

1.41622356

cg11500727 0.170652816 10.04088365 0.001613819 0.002666909 -

1.249838151

cg16319578 0.101447817 10.03416777 0.00161961 0.002675161

1.806776236

cg17307280 0.055245507 10.03057251 0.001622718 0.002678539 -

1.436270016

cg03752628 0.049897688 10.011849080.001639004 0.002702323

1.273104413

cg17272843 0.072983217 10.00831323 0.001642099 0.002706981

1.574159725

cg01027739 0.05867893 10.0051742 0.00164485 0.002711074-1.290230142

cg00480115 0.155576932 9.998255105 0.001650933 0.002720654

1.314118675

cg16504798 0.14252717 9.996983796 0.001652053 0.002722055

1.27197454

cg16750914 0.01318526 9.994697813 0.001654069 0.002724931 -

1.265360768

cg02913089 0.031652867 9.98815932 0.001659849 0.002734006 -

1.366531427

cg13885201 0.039461487 9.983556297 0.00166393 0.002738937 -

1.36992725

cg26486702 0.062624224 9.981965739 0.001665343 0.002740367

1.242739107

cg00105253 0.044092828 9.980465455 0.001666676 0.002741666

1.372597879

cg26624134 0.057535151 9.976395646 0.0016703 0.002746729

1.265037604

cg26833602 0.01502426 9.9651168530.001680382 0.002761958

2.023938271

cg27090216 0.304276903 9.959543027 0.001685388 0.00276883 -

1.325034209

cg17471928 0.045466746 9.956015716 0.001688563 0.002773595 -

1.267643197

cg01530101 0.1142255579.950733984 0.00169333 0.002780517

1.432501498

cg24130043	0.039998807	9.941518023	0.001701679	0.002792862	-1.412499025
cg12515638	0.087598549	9.929309039	0.001712805	0.002809749	-1.411767956
cg10078415	0.065292858	9.924083444	0.001717589	0.00281668	1.548366239
cg19343464	0.186254405	9.914922816	0.001726009	0.002829107	-1.354628779
cg17565490	0.038289563	9.903563203	0.001736509	0.002844003	2.41980632
cg06214007	0.143868314	9.89177448	0.001747474	0.002861496	1.326627785
cg00485296	0.02940155	9.881981854	0.001756636	0.002876031	2.923469883
cg09226684	0.085028597	9.876662052	0.001761633	0.002883745	1.312777457
cg24652919	0.209717309	9.87343727	0.00176467	0.002887308	1.22662857
cg26186727	0.363895587	9.869767596	0.001768131	0.00289168	1.222623243
cg20330296	0.040827932	9.856589246	0.001780621	0.002910381	1.208175761
cg15205507	0.124260127	9.856065324	0.001781119	0.002910441	

1.218412313

cg11241627 0.243186897 9.850108161 0.001786796 0.002919244

1.316699089

cg18008766 0.085923368 9.84936052 0.001787509 0.002919936 -

1.369375448

cg12435792 0.127933789 9.847436209 0.001789348 0.002922465

1.271318413

cg27546237 0.031512813 9.845694058 0.001791014 0.002924712

1.22406246

cg09396217 0.270415688 9.831413008 0.00180473 0.002946156 -

1.224128497

cg08647446 0.032257027 9.8195760110.00181618 0.002963407 1.20068276

cg26486663 0.065403196 9.801089289 0.00183421 0.002991857 -

1.344063514

cg12339802 0.040238862 9.783860295 0.0018511770.00301514 -

1.268913061

cg08612037 0.03733004 9.776286085 0.001858687 0.003025904 -

1.309783963

cg21201572 0.1141953039.773369224 0.001861587 0.003029646

1.269363211

cg20287640 0.056470088 9.769359553 0.001865581 0.003035166

1.28469482

cg02121427	0.241446795	9.765632214	0.001869302	0.003040238	1.297153899
cg19362572	0.182819621	9.757160055	0.001877787	0.003052454	1.205426172
cg09712066	0.219304257	9.753065339	0.001881903	0.00305777	1.230425749
cg26764244	0.043202423	9.751841653	0.001883134	0.00305879	1.638580249
cg06509239	0.112113052	9.751838553	0.001883137	0.00305879	-1.333927793
cg01692572	0.024591832	9.746724386	0.001888293	0.003066176	1.322179392
cg11639651	0.126944244	9.74519682	0.001889836	0.003067693	1.250726266
cg04270835	0.081720109	9.737488119	0.001897642	0.003079371	-1.241893449
cg05517572	0.085518603	9.699819251	0.001936258	0.003138496	1.212293308
cg25300386	0.104285145	9.668942726	0.001968506	0.003186667	-1.421972582
cg11990309	0.132917846	9.659686339	0.00197828	0.00320146	-1.266172955

cg08090640	0.120763815	9.644975401	0.001993915	0.003224844	1.385735768
cg23702568	0.028525112	9.639781532	0.001999465	0.003233147	1.210153404
cg09600829	0.036862751	9.638517172	0.002000818	0.003234816	1.223767965
cg10414946	0.170949239	9.633078758	0.00200665	0.003242684	1.344733143
cg06423920	0.086608317	9.621766856	0.002018837	0.003261331	1.526991533
cg22764338	0.038596861	9.605463588	0.002036533	0.003286814	1.268285499
cg01242619	0.037573138	9.602362761	0.002039916	0.003290389	1.418597847
cg26805528	0.225793534	9.602203617	0.00204009	0.003290389	1.313906018
cg06349174	0.023319351	9.586197629	0.002057648	0.003315523	1.787095014
cg24453664	0.059209613	9.583213316	0.002060939	0.003319764	1.325421472
cg23881725	0.103292449	9.57732708	0.002067445	0.00332918	-1.335865272

cg24030630 0.026064975 9.575596019 0.002069362 0.003331735

2.936556237

cg20498685 0.071798477 9.567430831 0.002078431 0.003344197 -

1.394135338

cg08395365 0.11076629 9.559352799 0.002087442 0.00335816

1.207503409

cg05078019 0.14800693 9.539827019 0.002109388 0.003390759

1.278916643

cg24745738 0.147652886 9.532799698 0.0021173440.003401377 -

1.218900536

cg12758687 0.275798167 9.526501278 0.0021245 0.003412329 -

1.200938699

cg09874127 0.027489871 9.51017483 0.002143166 0.003439991

1.333638212

cg19721889 0.053589934 9.505161268 0.002148931 0.003447173 -

1.367157682

cg08008403 0.053556669 9.497157803 0.002158167 0.003459786 -

1.259271046

cg15864184 0.074023131 9.489507967 0.002167033 0.003472342

1.214389632

cg04794268 0.122233295 9.487014408 0.002169931 0.003475879

1.202441445

cg08886154	0.262548823	9.484355202	0.002173026	0.003479002	1.200541187
cg27294629	0.114499158	9.473772088	0.002185387	0.003496629	1.261696901
cg19769182	0.119235722	9.473537984	0.002185661	0.003496629	-1.254941358
cg09423836	0.025894303	9.438212072	0.002227448	0.00355614	-1.249174703
cg18822544	0.274089157	9.41941944	0.002250008	0.003589882	1.218965988
cg02260587	0.158812133	9.408285576	0.002263484	0.003607383	1.474832056
cg24433189	0.168547396	9.400256553	0.002273252	0.003622379	1.254145673
cg25841987	0.079182605	9.387201765	0.002289227	0.003644952	1.237558321
cg26500816	0.014753141	9.360818333	0.002321862	0.003692246	1.386460603
cg03588357	0.063026953	9.358426638	0.002324843	0.00369582	-1.271394652
cg22139878	0.024100836	9.347727964	0.002338229	0.003715927	-1.206205013

cg05091653	0.035726329	9.347066028	0.00233906	0.003716661	1.830421646
cg20792062	0.188542754	9.341513931	0.00234604	0.003725989	-1.256446549
cg10377764	0.020568943	9.330482692	0.002359971	0.003746343	1.600456074
cg20398399	0.313857455	9.327176991	0.002364162	0.003751814	1.201837181
cg13325529	0.022315428	9.318077035	0.002375738	0.003768998	1.22694648
cg06849477	0.061758012	9.315101633	0.002379536	0.003774428	1.275201631
cg06653796	0.130587449	9.314461883	0.002380353	0.00377513	1.452816026
cg13140267	0.069567509	9.30991136	0.002386175	0.003783169	-1.365758993
cg27239157	0.081261849	9.309620244	0.002386548	0.003783169	-1.271507287
cg11762346	0.128595832	9.297844596	0.002401684	0.003805964	1.246090982
cg09227563	0.046579459	9.287674928	0.002414834	0.003825543	-1.36253927

cg19586576	0.102132507	9.287409672	0.002415178	0.003825543	-1.313845503
cg01236137	0.074147268	9.282542298	0.002421498	0.003833747	1.205832643
cg26036443	0.077905435	9.273272985	0.002433582	0.003849853	1.625967643
cg17001035	0.048186251	9.263368758	0.002446561	0.00386917	-1.34804046
cg09102409	0.064734207	9.257275403	0.002454581	0.003880636	1.275672306
cg01968793	0.174231472	9.249614541	0.002464702	0.003893935	-1.257594228
cg10059959	0.034339657	9.247346868	0.002467706	0.003897717	-1.235377
cg10896774	0.068507961	9.233710628	0.00248585	0.003923299	1.448873674
cg17285325	0.085307651	9.229653799	0.002491274	0.003930628	-1.420380293
cg18454685	0.111099094	9.228207605	0.00249321	0.003933067	-1.338692535
cg06638966	0.247705891	9.225914451	0.002496284	0.003936683	-1.260778994

cg25511429	0.289597839	9.220576145	0.002503454	0.003947373	-1.231757635
cg26556719	0.386009645	9.215958724	0.002509673	0.003955941	1.20755374
cg20263942	0.169628007	9.203144981	0.002527013	0.003980783	-1.250500728
cg09529667	0.02456674	9.201999538	0.002528569	0.003982611	-1.389867678
cg24650501	0.042663604	9.199763115	0.00253161	0.003986778	-1.323497742
cg09980522	0.270770786	9.186925187	0.002549138	0.004012499	1.203529213
cg20312687	0.311505828	9.181319736	0.00255683	0.00402335	-1.208865768
cg11668844	0.112141231	9.172092234	0.002569544	0.004042093	1.351440912
cg26401870	0.109234551	9.163638315	0.002581248	0.004059237	1.339778702
cg06277481	0.019692519	9.161931965	0.002583617	0.004062329	-1.259199926
cg17963840	0.314319024	9.158169993	0.002588848	0.004069918	1.215805915
cg05697249	0.085358547	9.15347089	0.002595397	0.004079577	

1.233952285

cg06121469 0.029839972 9.143987336 0.002608665 0.004097237 -

1.294473885

cg02019333 0.090677182 9.141694733 0.0026118830.004101652

1.265270831

cg15683488 0.044375302 9.137230784 0.00261816 0.004110869

1.244888229

cg24835159 0.120079161 9.128982144 0.0026298 0.004126943

1.248515487

cg18275051 0.05112732 9.124766299 0.002635769 0.004134548 -

1.387097992

cg06609049 0.138412027 9.123227369 0.002637952 0.004134856

1.231989631

cg26222229 0.020300629 9.122739985 0.002638643 0.004135296

1.218148839

cg03909500 0.073595986 9.1186283660.002644485 0.004143807 -

1.39518992

cg21602520 0.15326407 9.1157292440.0026486110.004149628 -1.280159431

cg16025584 0.053198463 9.102598419 0.002667385 0.004175145

1.225619467

cg14284643 0.039759567 9.090124103 0.002685345 0.004202605

1.241436315

cg15147435	0.050070344	9.064419967	0.002722743	0.004254525	2.164550583
cg04598121	0.351638967	9.063556601	0.002724009	0.004255842	1.218871354
cg19523029	0.069607577	9.060319108	0.002728759	0.004261941	1.250680402
cg24428760	0.017261546	9.04745236	0.00274772	0.004287569	1.204952614
cg25523753	0.059699677	9.044804051	0.00275164	0.004292356	1.262243308
cg02844545	0.270050109	9.044443039	0.002752174	0.004292525	-1.249486619
cg05010967	0.267017347	9.017211706	0.002792816	0.004353351	-1.249250184
cg10742801	0.161207702	9.004034467	0.002812702	0.004380825	-1.22523728
cg17775235	0.048331114	8.993324853	0.00282897	0.004404733	-1.370992678
cg16427670	0.064104125	8.988465678	0.002836382	0.004413614	-1.335407375
cg05628549	0.067216607	8.98395212	0.002843285	0.004422248	-1.369852426

cg03565081	0.187515265	8.983690475	0.002843686	0.004422248	-1.228263692
cg20308679	0.166519379	8.974715199	0.002857466	0.004439566	-1.348345066
cg07537523	0.084317063	8.969793967	0.00286505	0.004449977	-1.273668858
cg01407797	0.029907948	8.956926319	0.002884979	0.004478169	1.278074772
cg15435730	0.066453951	8.947744156	0.002899286	0.004496912	-1.290380301
cg02008154	0.227550938	8.93721594	0.002915779	0.004518319	-1.289213102
cg16970828	0.025536177	8.934469779	0.002920097	0.004522925	1.499002691
cg18089852	0.026128182	8.926703692	0.002932343	0.004540496	-1.432927323
cg04101379	0.069178369	8.916450692	0.00294859	0.004562849	-1.363509339
cg24907852	0.055797879	8.914771296	0.00295126	0.00456628	1.233094881
cg01404615	0.058593304	8.910561737	0.002957964	0.004575844	-1.34103467

cg15379887	0.10029201	8.910034486	0.002958804	0.004575844	1.226677062
cg11577097	0.163051257	8.893031884	0.002986046	0.004612464	1.214665054
cg01543654	0.190601505	8.873941274	0.003016938	0.004655031	-1.275932982
cg19006008	0.028709159	8.849616166	0.003056772	0.004709283	1.77940763
cg18438777	0.082670273	8.842589969	0.003068378	0.004725717	-1.343776324
cg08530414	0.031061429	8.822399664	0.003101978	0.004773227	-1.280958722
cg15972294	0.035954486	8.822345691	0.003102068	0.004773227	1.473875409
cg09038885	0.021178414	8.821922689	0.003102776	0.004773587	-1.203430524
cg24199834	0.239398669	8.818600828	0.003108342	0.004781419	-1.313626067
cg11961618	0.02011124	8.809375192	0.003123851	0.004803484	1.256511263
cg27433088	0.04744598	8.806961319	0.003127923	0.00480787	1.252202139

cg14800883 0.092503525 8.777517933 0.003178018 0.004875202
1.239572314

cg24022301 0.05979428 8.772815755 0.003186094 0.004886847 -
1.328900247

cg19761273 0.046568236 8.764666792 0.003200139 0.004904656
1.270999484

cg27020690 0.064158392 8.7624116860.003204037 0.004909137
1.983005595

cg22747092 0.092477004 8.760129244 0.003207987 0.004914442
1.342710587

cg21296230 0.340595204 8.757056208 0.003213313 0.004921853 -
1.220240915

cg17190608 0.049390563 8.746832337 0.003231098 0.004943833 -
1.451490971

cg06043042 0.023961725 8.738879176 0.003245002 0.004962847 -
1.239309324

cg01520924 0.059810456 8.73339437 0.003254627 0.004976811-
1.280555244

cg04527363 0.127722683 8.728051795 0.00326403 0.004988918
1.367713947

cg26018901 0.056141393 8.726189791 0.003267313 0.004992422 -
1.323651062

cg23123362	0.092805059	8.715543252	0.003286153	0.005018925	1.261732692
cg24446548	0.063149398	8.71045556	0.003295195	0.005029859	-1.342235284
cg18053505	0.070288828	8.710391608	0.003295309	0.005029859	1.305117653
cg17387870	0.165882916	8.704651524	0.003305541	0.005043482	-1.273546141
cg21091679	0.043605261	8.704542817	0.003305736	0.005043482	-1.335481347
cg11612345	0.082342937	8.69933851	0.003315041	0.005054618	1.626436196
cg17602451	0.042732716	8.69604755	0.00332094	0.005062079	-1.42404368
cg18142353	0.062482332	8.687058308	0.003337106	0.005085182	1.248645123
cg09593286	0.028146648	8.679468088	0.003350818	0.005103762	1.213675308
cg20420433	0.071350948	8.669866662	0.003368246	0.005125056	-1.316192032
cg18931421	0.031258189	8.657979372	0.003389952	0.005153241	-1.229572768

cg10891879	0.032438703	8.649498453	0.003405525	0.005175352	1.458224342
cg06521852	0.04097651	8.632128153	0.003437649	0.005220234	1.252020327
cg01389761	0.070448565	8.618258279	0.003463521	0.005255562	1.812038414
cg07141002	0.138390695	8.597563469	0.003502494	0.005307505	1.305570043
cg07499072	0.070004893	8.586864318	0.003522817	0.005335895	-1.31809024
cg12645220	0.036032063	8.577081604	0.003541506	0.005360978	-1.301289902
cg10685945	0.025078673	8.572319569	0.003550639	0.005372793	-1.219865703
cg02409351	0.104122416	8.555631855	0.003582837	0.005417846	-1.392157839
cg02883230	0.132330413	8.554954012	0.003584151	0.005418407	1.228688242
cg14254380	0.028265694	8.554885711	0.003584283	0.005418407	-1.353156713
cg13174197	0.126732931	8.533745135	0.003625517	0.00547253	1.208331331

cg06856528	0.082161022	8.515129581	0.003662227	0.00552215	-1.340639795
cg00259755	0.132801914	8.508970963	0.003674455	0.005538101	-1.228561965
cg02624129	0.074186951	8.507064821	0.003678248	0.005542989	1.299721423
cg25388528	0.245135319	8.499481829	0.003693377	0.005563292	1.213054348
cg15223781	0.066436004	8.498861914	0.003694617	0.005564327	1.220948493
cg19147390	0.256682446	8.497528331	0.003697285	0.005566682	1.206870882
cg15736338	0.041296276	8.496835406	0.003698672	0.005567938	-1.31172814
cg01794265	0.023163873	8.491906892	0.003708554	0.00558198	-1.227710796
cg10893007	0.15780486	8.488468197	0.003715464	0.005591546	-1.368787775
cg17125623	0.137120323	8.468872338	0.003755096	0.005647815	1.217738828
cg19035457	0.108652343	8.457339038	0.003778623	0.005677268	1.217086094

cg22815110	0.424117908	8.455675728	0.003782028	0.005681537	-1.205477795
cg10331779	0.195885409	8.454330112	0.003784786	0.005684832	-1.216756769
cg21416022	0.103733378	8.453620282	0.003786241	0.00568617	1.280117813
cg13846866	0.066120707	8.452095495	0.003789369	0.00569002	1.206677261
cg01267315	0.044861786	8.439321038	0.00381568	0.005726967	-1.382095785
cg16293105	0.077480441	8.415664053	0.003864898	0.005793071	-1.298992473
cg04490714	0.211545141	8.397335232	0.003903475	0.005845678	-1.289982473
cg20537992	0.121532406	8.381420878	0.00393729	0.005892814	-1.243108739
cg03005261	0.084446578	8.374956216	0.003951111	0.005911743	-1.330693056
cg07671976	0.03233553	8.371210706	0.003959142	0.005922	-1.34539326
cg03160135	0.103401138	8.359625463	0.003984087	0.005955775	-1.244675083

cg20530056	0.099193791	8.359040981	0.003985349	0.005956779	1.221963626
cg03684977	0.163895194	8.353507756	0.003997323	0.005972904	1.22244802
cg23051598	0.220545647	8.350101436	0.004004713	0.005983059	1.20941451
cg10708793	0.017260609	8.346761115	0.004011973	0.00599124	2.522086054
cg13633560	0.089733028	8.313668237	0.00408463	0.006089812	-1.222588437
cg11516377	0.115853234	8.313341408	0.004085354	0.006089991	-1.319367832
cg20300246	0.107180588	8.312058697	0.004088198	0.006091526	-1.222846662
cg13640200	0.037395533	8.306023181	0.004101605	0.0061106	1.627680293
cg21870662	0.074213925	8.302727812	0.004108944	0.006119724	-1.245919391
cg09715672	0.056882574	8.30136644	0.00411198	0.006123341	1.942147089
cg27202708	0.155473672	8.285789026	0.004146881	0.006168021	-1.288687448
cg20449692	0.088674546	8.283902447	0.004151129	0.006173427	

1.678734743

cg21902544 0.100575874 8.266370427 0.004190813 0.00622554 -

1.381036499

cg01704534 0.046150895 8.266192324 0.004191218 0.00622554

1.253746771

cg26372517 0.1166707068.26596656 0.004191731 0.00622554 1.307915344

cg26119740 0.125797486 8.264284017 0.004195561 0.006228473

1.203292701

cg07665060 0.1188917288.257357442 0.0042113640.006250221

1.285273324

cg17530977 0.039880209 8.253577526 0.004220014 0.006262007 -

1.289530492

cg23402444 0.032264506 8.224543432 0.004287061 0.006346541 -

1.249679765

cg14265075 0.157608174 8.219267061 0.004299362 0.006361947 -

1.237846563

cg15966757 0.146822263 8.208778496 0.004323921 0.006394531

1.218965583

cg20630207 0.1189995498.204878363 0.004333089 0.00640715

1.213707091

cg25599242 0.045606866 8.201736959 0.004340489 0.006413386

1.335827507

cg10194829	0.069218474	8.186530649	0.004376489	0.006461841	1.243609733
cg00333528	0.120277632	8.183273086	0.004384241	0.006469911	1.446676297
cg25982743	0.132447531	8.172390579	0.004410239	0.006504048	1.312176374
cg12164282	0.05336714	8.170200968	0.004415489	0.006510837	1.686697779
cg05337441	0.071514523	8.163294022	0.004432092	0.006531495	1.50303108
cg14934766	0.023228881	8.148612048	0.004467595	0.006579004	-1.246103507
cg14072120	0.026193499	8.146828728	0.004471927	0.006584421	1.396787385
cg25298754	0.07368302	8.138405638	0.004492447	0.006613668	1.342076896
cg20387341	0.188066654	8.135123807	0.004500468	0.006623541	-1.237602993
cg04770504	0.017828271	8.12823186	0.00451736	0.00664646	-1.223770595
cg10073723	0.015137727	8.089470854	0.004613577	0.006777138	-1.218996442
cg14036856	0.094875839	8.0818895	0.004632639	0.006804148	

1.228983892

cg01971122 0.090345019 8.081297373 0.004634132 0.006804356

1.50022812

cg20483374 0.167245602 8.071891535 0.004657901 0.006834278 -

1.250007183

cg11308840 0.088823867 8.069555141 0.004663825 0.006840977 -

1.305221534

cg07629017 0.048068897 8.06039411 0.004687126 0.006873155 -

1.368549167

cg22983529 0.035018549 8.053088503 0.004705793 0.006896513

1.229462296

cg00842351 0.229152064 8.044757315 0.004727174 0.006918789

1.292486304

cg08792314 0.029210833 8.037104449 0.004746901 0.006944636

1.822856729

cg15674432 0.10695349 8.033252941 0.004756861 0.006955168

1.303202828

cg07651242 0.105564729 8.028722074 0.004768605 0.006971328 -

1.361576908

cg07935264 0.105109685 8.025543459 0.004776862 0.006981372

1.384933368

cg08572611 0.121433169 8.01429265 0.004806204 0.007016692 -

1.338186762

cg24056567 0.215170175 8.0112218130.004814245 0.007025817 -

1.240327

cg07328579 0.044041648 7.991622988 0.004865888 0.007093991 -

1.267924931

cg02331561 0.023551804 7.976045666 0.004907339 0.007147183 -

1.248598749

cg25558099 0.077988149 7.962106315 0.004944736 0.007196448 -

1.39966225

cg13549845 0.135097197 7.952342499 0.0049711050.007228559 -

1.219993623

cg26239233 0.128622108 7.937741672 0.005010805 0.007279984

1.221054989

cg00056767 0.045499653 7.933862344 0.005021407 0.007292507 -

1.331411304

cg10970251 0.033836358 7.933793474 0.005021595 0.007292507

1.903160628

cg12300353 0.242189199 7.929955898 0.005032107 0.007306718 -

1.300288092

cg26578617 0.271997059 7.9191135510.005061926 0.007341552 -

1.205201736

cg21264055 0.089299081 7.884558477 0.005158172 0.007471463 -

1.331462265

cg22660578 0.133599885 7.878563027 0.00517506 0.007492694 -

1.327923302

cg20120491 0.087799238 7.877272691 0.005178703 0.00749689

1.544789102

cg24653181 0.044163853 7.871527749 0.00519495 0.007516091 -

1.387470858

cg22129364 0.0735113267.864950895 0.005213615 0.007538765 -

1.344669627

cg17329249 0.049232403 7.856262252 0.005238377 0.007571311

1.651499304

cg14667273 0.043778048 7.854328217 0.005243906 0.007578215

1.504004225

cg21215336 0.026386732 7.849061348 0.005258991 0.007598925

1.306529707

cg26829131 0.044730701 7.845608802 0.005268904 0.007609975 -

1.333796597

cg05656364 0.054903766 7.841217073 0.005281541 0.007627133

1.334668636

cg20372689 0.1108816327.834929473 0.005299687 0.007649746

1.228403735

cg26626089 0.148787977 7.809627902 0.005373356 0.007744185

1.312150152

cg10521852 0.059280024 7.807322957 0.0053801190.007752823

1.226637843

cg19616230 0.014299316 7.801625383 0.005396873 0.007773631

2.204928975

cg10530281 0.081940743 7.80110284 0.005398413 0.007774737 -

1.382860864

cg13228642 0.098997435 7.788456958 0.005435802 0.007820759 -

1.255830396

cg00739120 0.1131943467.785070061 0.00544586 0.007832994

1.294274328

cg00564163 0.072776782 7.773936328 0.005479061 0.007878497 -

1.25681806

cg21311175 0.073580792 7.772853359 0.005482301 0.007882032

1.228701944

cg22335801 0.081336555 7.770663581 0.005488859 0.007889209 -

1.372690546

cg26705553 0.068857539 7.763915642 0.0055091190.007913813 -

1.356931917

cg25263140 0.149140204 7.763028701 0.0055117870.007916518 -

1.335536535

cg21835643 0.256958272 7.762638623 0.005512961 0.007917075

1.24423102

cg06377278 0.052446394 7.760037285 0.005520798 0.007926069 -

1.524914429

cg06337239 0.096002604 7.755981655 0.005533037 0.007941378

1.227550089

cg07536920 0.0178331187.75488768 0.005536344 0.007943861 -

1.258582226

cg08097882 0.131639233 7.745228472 0.005565624 0.007980192 -

1.418930484

cg11984608 0.174762751 7.740050008 0.005581388 0.008000517

1.238499201

cg18884137 0.07191473 7.719501213 0.005644389 0.008080479 -

1.273082403

cg12686915 0.039228318 7.712345559 0.005666498 0.008110978

1.232125315

cg11847808 0.068496093 7.699171627 0.005707435 0.008167253 -

1.231748227

cg21968580 0.070060526 7.697914202 0.0057113580.008171706

1.25581637

cg13022174 0.024189094 7.697478714 0.005712717 0.008172491

1.229913731

cg03085312 0.071613737 7.694252285 0.005722798 0.008185751

1.37956206

cg24429836 0.150395438 7.685793947 0.005749314 0.00821901

1.246503763

cg13102585 0.104052056 7.647942067 0.005869522 0.008374221 -

1.213003341

cg01284306 0.1100144117.625252002 0.00594281 0.008465597

1.215464425

cg21428681 0.267462477 7.622062281 0.005953187 0.008477982 -

1.210385986

cg03030757 0.0552011477.614973853 0.005976315 0.008509716 -

1.268806904

cg14471615 0.02754287 7.614251895 0.005978676 0.008511875-

1.305461334

cg23043245 0.070806569 7.6119348910.005986259 0.008521466

1.261168526

cg02293044 0.0748081147.598638831 0.006029964 0.0085752

1.243401675

cg19194454 0.031970063 7.575473344 0.006106892 0.008674804

1.686074254

cg15940569 0.1125070877.5748113110.006109105 0.008676724 -

1.233341023

cg27138018 0.10619242 7.572032473 0.0061184040.008687482 -1.2588918

cg21922574	0.08717472	7.571541449	0.006120048	0.008688593	1.589348531
cg01246254	0.070876766	7.564210729	0.006144655	0.008717385	1.228087448
cg23582408	0.049487247	7.555663259	0.006173474	0.008752109	-1.384730409
cg05154390	0.039016942	7.554916049	0.006176	0.008754458	1.258806364
cg14483391	0.118744343	7.551462347	0.006187688	0.008769793	1.238768041
cg15043057	0.234794975	7.543631993	0.006214273	0.008801281	-1.219308148
cg00685836	0.021405258	7.533556834	0.006248652	0.008844998	-1.397706863
cg10104451	0.037180819	7.533022742	0.00625048	0.008846343	1.236230165
cg18445047	0.034838001	7.528358699	0.006266465	0.008866476	1.233668941
cg17178336	0.060773021	7.525980953	0.00627463	0.008876783	-1.377065102
cg00818693	0.02591388	7.515555834	0.00631056	0.008920098	1.719216556

cg23256150	0.206051971	7.506717169	0.006341188	0.008955852	-1.208395909
cg19528976	0.103258632	7.505234816	0.006346339	0.008961017	-1.333812377
cg01998146	0.048910757	7.505152996	0.006346624	0.008961017	1.209844891
cg05389183	0.05848987	7.503929828	0.006350878	0.008964606	1.213282834
cg01254505	0.028505995	7.503082977	0.006353825	0.008967387	1.732931691
cg12317456	0.093544858	7.498911617	0.006368362	0.008983962	-1.251305687
cg20761322	0.046048666	7.49869545	0.006369116	0.008983962	-1.216835169
cg21053529	0.258717599	7.493994848	0.00638554	0.009004608	-1.228045105
cg16184943	0.128076295	7.493353334	0.006387785	0.009006513	-1.284369769
cg13794888	0.027582281	7.483727263	0.006421566	0.009047812	1.364780596
cg15835825	0.043920269	7.478180393	0.006441115	0.009072818	-1.471537478

cg00792849	0.25283692	7.462300945	0.006497417	0.009145731	1.271683885
cg18264687	0.065634794	7.459697768	0.006506694	0.009157511	1.262744793
cg27403635	0.070250745	7.438584555	0.006582445	0.009254765	-1.37028519
cg01966465	0.017601736	7.435063986	0.006595164	0.009267783	-1.363420962
cg09188980	0.313360644	7.432333977	0.006605044	0.009280373	-1.205808461
cg02919422	0.042384917	7.427036797	0.006624258	0.009302181	1.332827483
cg03382304	0.048911917	7.413905065	0.006672138	0.009355073	-1.273381123
cg22915732	0.087068381	7.401425039	0.006717969	0.009414094	-1.213119813
cg27140220	0.030808164	7.375059615	0.006815857	0.009535352	-1.293751655
cg09955730	0.082528652	7.357255272	0.006882785	0.0096183	1.219734804
cg07525077	0.351730742	7.356716957	0.006884819	0.009619809	1.202360451

cg20312228 0.050327916 7.352124974 0.006902195 0.009638653

1.262755252

cg06352750 0.095282163 7.341249694 0.006943526 0.009687466 -

1.319649164

cg01555431 0.040832748 7.337880651 0.006956381 0.009699622

1.221866323

cg03262773 0.071272677 7.3351144160.006966955 0.009713021

1.376381322

cg01626227 0.078421589 7.33286989 0.006975546 0.009723654

1.61270164

cg19329389 0.064573439 7.302859209 0.007091467 0.009867771

1.312043692

cg14147105 0.032271778 7.294279599 0.007124968 0.009910006 -

1.287958924

cg19766460 0.07258193 7.288356219 0.007148193 0.009937202

1.505520008

cg23559331 0.07098798 7.286790156 0.007154346 0.009944003 -

1.327097293

cg02620769 0.062610782 7.284704139 0.00716255 0.009954033

1.898769808

cg25186143 0.104573618 7.277443045 0.0071911840.009986939 -

1.325515734

cg12640109	0.094243871	7.274511074	0.007202779	0.00999753	1.204372199
cg04389838	0.175964543	7.269050254	0.007224427	0.010020674	1.230659837
cg06027949	0.041145685	7.263285278	0.007247352	0.010046941	1.21221025
cg15647515	0.026403772	7.246158394	0.007315901	0.010132212	-1.263520769
cg05362516	0.028152873	7.235884689	0.00735734	0.010181205	-1.248958285
cg01635061	0.054674619	7.23497418	0.007361024	0.010184904	-1.227448909
cg23889010	0.116208963	7.228037807	0.007389152	0.010219613	1.27643752
cg19759064	0.097783393	7.227033024	0.007393235	0.010222574	1.244008889
cg16797831	0.175778103	7.223490666	0.007407651	0.010238286	1.278278769
cg16270890	0.063149577	7.223220844	0.00740875	0.010238286	1.286771116
cg08806153	0.018348673	7.216505442	0.007436161	0.010274756	-1.329590263

cg14586939 0.142594302 7.214965362 0.007442462 0.010282053 -

1.304493713

cg16232126 0.165398975 7.207639294 0.0074725110.010320319 -

1.304000777

cg04245402 0.099586066 7.204816231 0.007484123 0.010332525

1.240030704

cg00512279 0.1100792787.19901973 0.007508024 0.010359844 -

1.358784712

cg15494980 0.055421701 7.197603668 0.007513875 0.010365078

1.21797066

cg11905589 0.0657730117.194798269 0.00752548 0.010376825

1.208517003

cg25884854 0.021479817 7.193204277 0.007532082 0.010381666

1.297352622

cg14262937 0.180965891 7.186910851 0.007558206 0.010414824

1.352307579

cg25416372 0.067484787 7.183633874 0.007571845 0.010432192 -

1.261804743

cg21672276 0.063859375 7.182097813 0.007578248 0.010439585

1.607770023

cg07379574 0.12530524 7.14606112 0.007730048 0.01062291 -1.224632582

cg25908985 0.15286551 7.146001088 0.007730304 0.01062291 -

1.31492247

cg07212894	0.03605724	7.141463745	0.007749637	0.010645123	-1.372928167
cg22936016	0.089180169	7.140308657	0.007754567	0.010648991	1.213774654
cg21092462	0.094461559	7.134661337	0.007778715	0.010673424	-1.238173633
cg02665570	0.303723607	7.129005952	0.007802975	0.010705254	1.247267184
cg24873414	0.068905033	7.126393932	0.007814206	0.010716285	1.225641818
cg26021627	0.111186465	7.122778985	0.007829777	0.010730336	-1.265591997
cg04784672	0.101579582	7.121531068	0.00783516	0.010736252	-1.350695492
cg18997990	0.055439905	7.112744991	0.007873164	0.010780996	1.201173269
cg18267374	0.323330238	7.101427009	0.007922398	0.010845467	-1.233795873
cg11591325	0.055455733	7.085284853	0.007993165	0.010924533	-1.358356686
cg13060154	0.051658175	7.083621916	0.008000492	0.010931581	

1.720807779

cg14958635 0.367796774 7.080135397 0.008015877 0.010949632 -

1.206740701

cg19996355 0.026325549 7.067665432 0.0080711490.011020652-

1.344017318

cg09297361 0.03239266 7.065860573 0.008079182 0.011030125

1.234241427

cg24939733 0.215317073 7.04725314 0.008162472 0.0111242411.2093019

cg22176895 0.109356425 7.045239122 0.008171539 0.011135093-

1.284701629

cg04062391 0.2049922117.04400147 0.0081771170.01113968 1.34265411

cg04759439 0.095651909 7.038134341 0.00820361 0.011166713

1.207199156

cg26728422 0.059754824 7.036909082 0.008209154 0.011172749

1.572687162

cg09405612 0.035316976 7.033487705 0.008224655 0.011190823-

1.203377015

cg12089439 0.047794987 7.024521856 0.008265418 0.011238698

1.200549308

cg26952662 0.042106496 7.009718968 0.008333173 0.011320133-

1.398004747

cg18511007 0.132963352 7.00872322 0.008337751 0.011324313-

1.335386289

cg22165175 0.104127131 7.006078699 0.008349922 0.011338299-

1.210970199

cg18676237 0.0413001136.994605064 0.00840294 0.01140281 2.185390294

cg22341104 0.1121287736.950163584 0.0086115790.0116606 -1.347458737

cg16541031 0.052642077 6.948554042 0.008619234 0.01166783

1.905130077

cg06392241 0.09730245 6.946060034 0.00863111 0.011682336-1.270833983

cg19319069 0.072499927 6.944423432 0.008638912 0.011691326

1.435955552

cg08268266 0.058583672 6.935524397 0.008681464 0.011742604

1.232975783

cg13847070 0.141337807 6.922239775 0.008745386 0.01181796

1.212806412

cg05454446 0.159975731 6.907887393 0.00881499 0.011900845-

1.217854965

cg23473904 0.0453439 6.8981167480.008862699 0.011959276-1.330279253

cg06493386 0.21348943 6.886792799 0.008918325 0.012027461 -

1.260695769

cg18766912 0.061808774 6.88636291 0.008920443 0.012028708

1.245588052

cg12359315 0.11392782 6.881828328 0.008942824 0.012054047

1.212603868

cg21387302 0.046606861 6.876244654 0.008970462 0.012083218

1.211312611

cg01636591 0.152261958 6.874380086 0.00897971 0.012094059

1.204876371

cg09085198 0.027006681 6.8731164380.008985984 0.012100891 -

1.283645571

cg14070647 0.061682374 6.869331055 0.009004804 0.012122994 -

1.30735989

cg14289511 0.045162509 6.86575155 0.009022638 0.012143658

1.795763167

cg22708914 0.0332491156.865524794 0.009023769 0.012143658

1.269368015

cg08169325 0.025039246 6.85969625 0.009052889 0.012176341 -

1.256125212

cg23414387 0.088534986 6.855808996 0.009072363 0.012199278

1.296958399

cg25332298 0.076486857 6.852731867 0.00908781 0.012218417 -

1.283928565

cg01366419 0.030469479 6.848573915 0.009108725 0.012241637

1.484325458

cg00317680 0.108535145 6.84396618 0.009131961 0.012267955

1.213904444

cg13043862 0.080304865 6.827056037 0.009217756 0.012368371 -

1.222562734

cg21696393 0.147551048 6.822633572 0.00924033 0.01239536 -

1.21377422

cg05275231 0.059454424 6.811187771 0.009299019 0.012459158

1.228021702

cg25887294 0.03302916 6.810024833 0.009305003 0.012463861

1.265572868

cg15747595 0.294395885 6.809397262 0.009308234 0.012466532 -

1.228707863

cg02497700 0.114338842 6.791549167 0.009400609 0.012578546 -

1.205914214

cg15414833 0.030637788 6.785283358 0.009433262 0.01261721 -

1.409925709

cg00445824 0.076127617 6.783530954 0.009442415 0.0126261

1.300334237

cg23526055 0.032440727 6.779805399 0.009461904 0.012648804

1.246009005

cg26864028 0.04513018 6.778879757 0.009466753 0.012653606 -

1.4165564

cg04575343 0.015087856 6.778384892 0.009469346 0.012655394 -

1.271161349

cg19531713 0.161538586 6.777499732 0.009473987 0.012659916 -

1.29657431

cg24562819 0.088976859 6.772220342 0.009501712 0.012695281 -

1.36289998

cg13959523 0.031649973 6.768726105 0.009520108 0.012714802 -

1.336124225

cg09879797 0.029690962 6.768004841 0.009523909 0.012718193

1.205156687

cg06055013 0.06788761 6.740745466 0.009668743 0.012883136 -

1.201514945

cg17119387 0.024377753 6.739276283 0.009676613 0.012891358

1.893396512

cg25616762 0.019107745 6.729690301 0.009728124 0.012954844 -

1.233929714

cg13576290 0.228625629 6.728575768 0.009734131 0.01296113

1.200999261

cg22131172 0.056514423 6.722892975 0.009764821 0.012998558

1.204519862

cg10057295 0.060880004 6.722520397 0.009766836 0.012999524

1.280356916

cg08958015 0.059787998 6.712997352 0.009818499 0.013056213

1.788935684

cg25942450	0.175284612	6.701353339	0.00988205	0.013132054	-	1.302319011
cg22334665	0.050832978	6.69954939	0.009891933	0.013141722	-	1.409218046
cg01918706	0.023053259	6.693581171	0.009924704	0.013181782	-	1.238468227
cg19843036	0.026755351	6.68738077	0.009958868	0.013225415		1.488308413
cg26872475	0.025667353	6.670610105	0.010051881	0.013330222		2.032905076
cg00243313	0.046996775	6.665915313	0.010078079	0.013360834	-	1.388586237
cg24250393	0.209672101	6.650972901	0.010161928	0.013457829	-	1.206679677
cg15452573	0.029982982	6.643071502	0.010206556	0.013506281	-	1.289517708
cg13918811	0.117028288	6.640369715	0.010221863	0.013523867	-	1.209139708
cg19890858	0.030948335	6.634663639	0.010254267	0.013555173		1.224227161
cg25097436	0.043800253	6.633096201	0.010263186	0.013565185	-	

1.283299824

cg17682828 0.036858948 6.623449089 0.010318261 0.013634401

1.390895379

cg14645481 0.065356467 6.617780272 0.010350765 0.013673765

1.229818103

cg27650434 0.022717162 6.608027574 0.010406933 0.013731761 -

1.21865525

cg10844844 0.019474369 6.601224279 0.010446299 0.013778291

1.32598494

cg14116122 0.075450851 6.584841096 0.01054173 0.013880539 -

1.385844926

cg07080358 0.05594969 6.571047076 0.010622773 0.013976292 -

1.41555514

cg06226384 0.207033225 6.563514063 0.010667302 0.014029382

1.272253946

cg12456510 0.187416095 6.548866808 0.010754435 0.014127381

1.214255252

cg25123470 0.078277541 6.542013748 0.010795454 0.014175719

1.391158255

cg04543012 0.034189291 6.524076515 0.010903579 0.01429772

1.729832495

cg07850604 0.04440298 6.523515415 0.010906979 0.014297941 -

1.401160714

cg02309273 0.259166283 6.509676868 0.0109911860.014395217 -

1.222602805

cg26450866 0.030332883 6.497572081 0.0110653910.01447359 -

1.292370108

cg05590257 0.066002748 6.496085606 0.0110745390.014481796 -

1.295828276

cg09547224 0.094696032 6.489575458 0.011114695 0.014530535

1.233556616

cg16158807 0.044164979 6.475219595 0.0112037760.014629908

1.24227859

cg27194921 0.030541464 6.471858889 0.0112247350.01465158

2.109987086

cg14885742 0.144987896 6.439149904 0.0114308510.014895536

1.216472269

cg08331313 0.1126782756.437892621 0.0114388520.014904034 -

1.317139211

cg18731789 0.035836534 6.401614265 0.0116721920.015180577

1.235131833

cg14307212 0.085739216 6.39596161 0.0117089880.015226468

1.201598616

cg06933965 0.131933925 6.38678479 0.01176898 0.015292639 -

1.252136328

cg19713196 0.023661897 6.384606906 0.0117832640.015309226

1.256617411

cg23696949 0.058989204 6.365782841 0.0119074710.015460632

1.30193495

cg11840540 0.026333952 6.356549648 0.0119688870.015532367 -

1.219331548

cg09084200 0.091739162 6.35264788 0.0119949380.015555687

1.245483433

cg17183546 0.036226819 6.351601575 0.012001934 0.015561223 -

1.224443263

cg21487207 0.108586343 6.345497852 0.012042828 0.01560822 -

1.30353192

cg25266232 0.12363599 6.344057479 0.0120525 0.015617254

1.362068605

cg11628034 0.033091955 6.33993688 0.012080212 0.015648619

1.406522749

cg00534274 0.055243497 6.333355243 0.0121246110.015702096

1.34262192

cg13191049 0.069669016 6.322505289 0.012198171 0.015791271 -

1.332612986

cg21200703 0.051486008 6.31798712 0.012228939 0.015820938

1.237016016

cg06038133 0.103656348 6.317641496 0.012231295 0.015821956

1.263084176

cg18988110 0.093861405 6.3168569 0.012236648 0.015826847

1.200607781

cg16179125 0.029025889 6.310597796 0.01227943 0.015876068

2.02116411

cg18611245 0.040178437 6.306705844 0.012306111 0.01590444

1.354543895

cg19629292 0.147407068 6.30053853 0.012348512 0.015951055 -

1.294955475

cg16741710 0.050038702 6.293421949 0.012397626 0.01600629 -

1.293817905

cg11311499 0.02529935 6.29234002 0.01240511 0.0160123 1.209355905

cg16388829 0.034173655 6.28647179 0.012445786 0.016060236 -

1.226534111

cg22040627 0.021276309 6.28463025 0.012458579 0.016073955 -

1.220994515

cg14576824 0.13441246 6.284482254 0.012459608 0.016073955 -

1.265936598

cg15044041 0.30646509 6.275976636 0.012518876 0.016144215

1.201432595

cg03469054	0.147699507	6.274853161	0.012526727	0.016152271	-1.268639878
cg05861567	0.158970177	6.27216992	0.012545496	0.016172334	1.205559213
cg20855565	0.026236494	6.271925075	0.012547211	0.016172475	-1.476944621
cg06117855	0.066030206	6.245016411	0.012737078	0.016396225	1.467369821
cg11171719	0.012376744	6.228635662	0.012854108	0.016529981	1.338083975
cg26704579	0.130284502	6.223330113	0.01289225	0.0165748	1.231029487
cg13105904	0.067345656	6.216693794	0.012940124	0.016622341	1.554079735
cg25418748	0.028081766	6.180664007	0.013203255	0.016903048	1.423395832
cg00973677	0.020043349	6.176701016	0.013232533	0.016932339	2.243724352
cg25913233	0.151262463	6.175551445	0.013241038	0.016941071	-1.2624706
cg16519321	0.10255903	6.172348966	0.013264761	0.016953889	-1.22131704
cg25095814	0.058785275	6.171824843	0.013268648	0.016953889	

1.298053624

cg24496666 0.2111704536.155450446 0.013390671 0.017091281 -

1.2513546

cg06346081 0.125018535 6.142062477 0.013491298 0.017210997 -

1.305757675

cg01837719 0.097296386 6.125106513 0.013619865 0.017359624 -

1.286209499

cg21426387 0.014921731 6.1182341420.013672333 0.017417686 -

1.251505192

cg07200280 0.03969485 6.099597133 0.013815672 0.017582506

1.400048859

cg01726775 0.0154231126.088674108 0.013900402 0.017679172

1.48425061

cg10983208 0.046255882 6.086949551 0.013913828 0.017691782 -

1.329159752

cg08977371 0.191494978 6.083583646 0.013940072 0.017720679 -

1.224771334

cg16063112 0.05436451 6.078296608 0.013981397 0.017768728 -

1.242252548

cg18239753 0.210667123 6.060160025 0.014124122 0.017918473

1.295711751

cg06816106 0.0163526 6.058905233 0.014134052 0.017923926

2.432335271

cg26509022 0.070856474 6.052848848 0.014182082 0.01797842 -

1.299329816

cg14614901 0.049065747 6.05087647 0.01419776 0.017993768 -

1.299939145

cg03310469 0.00869923 6.042500073 0.014264542 0.018060231

1.254206047

cg06668300 0.020263912 6.038127641 0.01429953 0.018097707 -

1.234871822

cg11375622 0.026218242 6.032674615 0.01434329 0.018148531

1.248379527

cg04498679 0.105236297 6.02998908 0.014364892 0.0181713

1.299551732

cg03013422 0.025572469 6.023105495 0.014420416 0.018230092

1.348750889

cg05497616 0.022103658 6.013040343 0.014502001 0.018321737 -

1.388354495

cg13351161 0.140819695 6.0061147840.014558414 0.018381485

1.208905033

cg21265783 0.030879093 6.000877829 0.014601223 0.018426299 -

1.2589808

cg10741760 0.217620608 5.991504095 0.014678173 0.018516449 -

1.23206659

cg02672493 0.030773163 5.989130889 0.014697721 0.018536468 -

1.314119669

cg02982690 0.038798443 5.984954456 0.014732187 0.018577611-

1.352250124

cg20523861 0.2862291185.983393559 0.01474509 0.018591555 -

1.207170901

cg19779211 0.1116985755.972090433 0.014838875 0.018693429 -

1.227569563

cg07693270 0.13999469 5.955309264 0.01497925 0.018846704 -

1.230430788

cg24068708 0.035323756 5.951551649 0.01501087 0.018879415 -

1.210373695

cg15013019 0.216259043 5.949651449 0.015026886 0.0188972

1.216640671

cg14213992 0.105708819 5.948866598 0.015033507 0.018903167 -

1.24940402

cg15146752 0.095002031 5.938036929 0.015125167 0.01900182

1.217797108

cg21697134 0.062063687 5.9334911280.015163813 0.019045622

1.540002406

cg02433671 0.021902437 5.927156497 0.015217837 0.019103951

2.11817647

cg13492340 0.152283357 5.924271091 0.0152425110.019128869

1.275753937

cg07684809 0.059250949 5.924033742 0.015244543 0.019128869

1.504037252

cg16606638 0.0220593 5.923947539 0.015245281 0.019128869

1.336319192

cg22740835 0.316018492 5.918631498 0.015290856 0.019164575

1.249808134

cg01182697 0.14280278 5.916329156 0.015310639 0.01918221

1.218546656

cg19536127 0.0186071145.915432932 0.015318346 0.019184981

1.342341508

cg00756058 0.01263561 5.910136213 0.015363981 0.019227523 -

1.308404711

cg03621406 0.155244967 5.905480645 0.015404208 0.019272981

1.22089847

cg26775866 0.041642275 5.90071955 0.015445459 0.019310302

1.223057258

cg25766046 0.082562369 5.893679692 0.015506663 0.019367875 -

1.234378447

cg25282780 0.0641126345.892879368 0.015513637 0.019371503

1.230989522

cg04283938 0.08798206 5.892600418 0.015516068 0.019372138 -

1.214412002

cg14042128 0.029930247 5.88468017 0.01558527 0.019441673

1.780638651

cg26344532 0.035950792 5.878237351 0.015641797 0.019498937 -

1.333898834

cg04658354 0.1711373955.878124889 0.015642786 0.019498937 -

1.320721056

cg06688396 0.054207153 5.8664411860.015745844 0.019606692

1.284168618

cg04583874 0.1042116675.861602841 0.015788727 0.019641973

1.220842252

cg26446827 0.024268299 5.859218456 0.015809904 0.019661036

1.465615694

cg17162024 0.087201316 5.85779905 0.015822525 0.019671875 -

1.336096741

cg22236626 0.054260313 5.839351534 0.015987501 0.019845153

1.280900619

cg24194539 0.290973659 5.837248778 0.016006419 0.019861295 -

1.239937585

cg24304714 0.127801289 5.795289343 0.016388788 0.020268356

1.209863493

cg11695266 0.088498224 5.794977381 0.016391666 0.020269427 -

1.364745701

cg10257049 0.064370144 5.785781921 0.016476732 0.020347149

1.475122499

cg01769037 0.091770539 5.778256088 0.016546693 0.020420042

1.202347472

cg19695867 0.1147102715.770751525 0.016616762 0.020497461

1.205166933

cg14603345 0.049181553 5.7684125110.016638663 0.020521965

1.397964538

cg11299964 0.054131885 5.761777032 0.016700958 0.020591237

1.32818843

cg20640433 0.065026826 5.761472428 0.016703824 0.02059225 -

1.209026454

cg23777956 0.16038243 5.756674671 0.016749024 0.020640396 -

1.200408052

cg02286642 0.06571514 5.753884542 0.016775368 0.020663128

1.448192281

cg08191854 0.022905763 5.751864008 0.016794473 0.020683755

1.387340705

cg01589580 0.104090634 5.742025034 0.016887825 0.020788561 -

1.232529835

cg20807701 0.036039123 5.730152369 0.0170011870.020894922

1.78276548

cg08900043 0.054928669 5.728737707 0.017014747 0.020907504 -

1.30754222

cg25964007 0.047935447 5.727441937 0.017027177 0.020919209

1.8467088

cg20587543 0.022335583 5.726222926 0.01703888 0.020928052 -

1.203481458

cg19280968 0.140825893 5.720102422 0.017097762 0.020975235 -

1.222345148

cg17194182 0.034022085 5.705498424 0.0172391110.02113063 -

1.200710916

cg01683883 0.375379554 5.6846511420.017442986 0.021348868 -

1.223548364

cg25947945 0.072445587 5.6801134950.017487691 0.02139628

1.227989861

cg23828595 0.041242942 5.677494693 0.017513546 0.021416118-

1.436558025

cg15139737 0.013527006 5.677310231 0.017515368 0.021416118

1.336083734

cg03554552 0.029255549 5.670062594 0.017587137 0.021497069

1.255587686

cg06850526 0.083892296 5.668510957 0.017602541 0.021510678

1.284101603

cg08465510 0.042024264 5.667333971 0.017614235 0.021519749

1.21509961

cg10172318 0.024382943 5.666758873 0.017619952 0.021524123

1.241022297

cg23739862 0.056949147 5.658788446 0.017699382 0.021602819 -

1.375763224

cg26219051 0.15132391 5.651869188 0.017768637 0.02167422 -

1.221626557

cg16378421 0.027838594 5.639136926 0.017896809 0.021806804 -

1.262362857

cg11572744 0.026628477 5.6388416 0.017899793 0.021807803

1.221710956

cg16340268 0.043731753 5.629394253 0.017995532 0.021913845

1.438387049

cg27201297 0.074172631 5.621836629 0.018072502 0.021999437

1.211313614

cg15937081 0.052337832 5.589336872 0.018407399 0.022366733

1.286321064

cg04868764 0.030570967 5.583084966 0.018472557 0.022435083

1.323583804

cg08896053 0.09527291 5.580469619 0.018499885 0.02246015 -

1.295818861

cg16076328 0.1138321085.552308007 0.018796815 0.022771255

1.233937448

cg23428445 0.021734887 5.549398184 0.018827776 0.022797798 -

1.306885787

cg11368578 0.040623818 5.54182766 0.018908574 0.022881884

1.343985796

cg15207619 0.13658487 5.538171316 0.018947727 0.022917678

1.258674293

cg02271621 0.034537145 5.51792796 0.019166018 0.023154491 -

1.312687242

cg23207990 0.1370181165.514596239 0.019202194 0.023195415 -

1.289522455

cg23009046 0.046581647 5.501339361 0.019346841 0.023333775

1.300149128

cg20052718 0.0418556 5.491523772 0.019454668 0.023452593 -

1.379374103

cg13801416 0.064489176 5.480137835 0.019580526 0.023590202 -

1.454855831

cg24154474 0.0221127745.477610173 0.019608581 0.023618353

1.257564072

cg13997435 0.036347357 5.470386088 0.019688991 0.023692548

1.33567789

cg26796283 0.09449465 5.467852951 0.019717268 0.023717693

1.370052657

cg01615704 0.0668524115.457601575 0.019832133 0.023842017

1.256098088

cg09009111 0.035777276 5.457018137 0.019838691 0.023844211-

1.255622552

cg07172280 0.0261125425.455401668 0.019856873 0.023860371 -

1.272206392

cg15796978 0.0511885065.446983554 0.019951838 0.023945925 -

1.293781445

cg13975625 0.095603698 5.446752572 0.019954451 0.023946208 -

1.249722552

cg21402071 0.041952528 5.443898081 0.019986764 0.023982129 -

1.217726894

cg18265887 0.049354503 5.443143675 0.019995313 0.02398953

1.26757187

cg12167564 0.045072234 5.428373719 0.020163453 0.024150899

1.396081116

cg20339650 0.126670773 5.425704154 0.020193999 0.024170343 -

1.205208165

cg18145505 0.1148418955.4219811170.02023668 0.02420705 -1.242568648

cg15160742 0.0169250115.421603487 0.020241014 0.02420936 -

1.202863784

cg08793459 0.037079971 5.420344568 0.020255471 0.024223776

1.94177662

cg10837843 0.04272597 5.415923495 0.020306324 0.024275948 -

1.254772724

cg21146268 0.199071872 5.4118161680.020353687 0.024326797

1.329751017

cg27637521 0.019009894 5.41117641 0.020361075 0.024332153

2.36009787

cg09131135 0.028145347 5.4110099110.020362998 0.024332153

1.250121226

cg23213217 0.096249878 5.407255896 0.020406408 0.024375352

1.242428781

cg18906795 0.04573885 5.4021129910.020466035 0.024434988

1.230207717

cg17525406 0.179507593 5.382722458 0.020692478 0.024687792 -

1.282228589

cg16092786 0.016863248 5.381285147 0.020709366 0.024705016

1.76174411

cg12457773 0.138544665 5.375810334 0.020773825 0.024756548 -

1.217138825

cg17671157 0.062980675 5.375738091 0.020774677 0.024756548 -

1.36020838

cg01120308 0.218903607 5.3721139140.020817464 0.024798736

1.224610046

cg15972617 0.070749049 5.363967098 0.020913978 0.024901932

1.261309966

cg10451401 0.022891824 5.363101475 0.020924261 0.024911231

1.235220528

cg18416881 0.055369692 5.36259163 0.020930319 0.0249155 -

1.432494729

cg07888234 0.032998964 5.351637299 0.021060933 0.025043459 -

1.216071481

cg08274234 0.0951138485.351492276 0.021062667 0.025043459 -

1.299293971

cg25990647 0.055285505 5.336147967 0.021247064 0.025221039 -

1.438482669

cg25753817 0.02648737 5.332803366 0.02128748 0.025260085

1.323541488

cg12343777 0.05884367 5.325387627 0.021377374 0.025351827 -

1.21322204

cg08532057	0.20710756	5.324075958	0.021393315	0.025364761	-1.2253023
cg10146929	0.160293091	5.323452986	0.021400891	0.025370757	1.330091486
cg05626013	0.126412369	5.318022822	0.021467041	0.025443191	1.208163916
cg17475456	0.0725524	5.316403409	0.02148681	0.025460631	1.325020245
cg00582628	0.053576679	5.290438843	0.021806364	0.025787727	1.223577908
cg22892110	0.025906978	5.279853333	0.021938056	0.025928248	1.284408193
cg19455368	0.022811898	5.275964579	0.021986643	0.025959037	1.999533646
cg24879335	0.120111207	5.270197282	0.022058906	0.026034431	-1.284131308
cg23300372	0.088594145	5.269464017	0.022068111	0.026042245	-1.351768897
cg04504095	0.020903007	5.258937241	0.022200704	0.026185459	-1.25297035
cg20404387	0.090565856	5.233164828	0.022528833	0.026517607	-1.28286079

cg15796941 0.055187203 5.224149852 0.022644796 0.026627181 -
1.238601662
cg10729531 0.033515342 5.214178946 0.022773779 0.026759031 -
1.209955229
cg27444994 0.096091877 5.204334103 0.02290188 0.0268876 -
1.304039919
cg02605634 0.080866913 5.200047388 0.022957893 0.026941017
1.282429561
cg09470640 0.068520732 5.193348933 0.023045704 0.027021819 -
1.297211766
cg26162582 0.187727062 5.153772287 0.023571699 0.027526447 -
1.268272369
cg08388746 0.08762584 5.153326877 0.023577689 0.027530251
1.201771903
cg08560942 0.0268115985.14163191 0.023735541 0.02768569 -1.232843728
cg18230771 0.038271372 5.136145488 0.023809973 0.027762866 -
1.297249349
cg07054641 0.273090061 5.135617772 0.023817145 0.027768015
1.237639296
cg10887021 0.123561037 5.125283664 0.023958046 0.027909683
1.384653722
cg25905812 0.032121027 5.120992326 0.0240168110.027971673 -

1.343782656

cg20273774 0.0582119085.1190208510.024043859 0.027996702

1.215209477

cg08431931 0.266383492 5.104684639 0.024241499 0.028188019

1.227616141

cg22083798 0.168478794 5.095806049 0.024364747 0.028306448 -

1.223529368

cg12966875 0.038277441 5.092300764 0.024413585 0.02835512

1.487934122

cg25229172 0.1124865615.091392247 0.02442626 0.028366571 -

1.205306611

cg04518808 0.099133666 5.078953879 0.024600477 0.028526149 -

1.21882248

cg16363586 0.21249227 5.077534765 0.024620436 0.028542723

1.311273905

cg17749443 0.029264409 5.0604980110.024861366 0.028795529 -

1.260421536

cg02497758 0.095584394 5.055180279 0.02493707 0.028866621 -

1.27190519

cg01344452 0.053868945 5.0372022 0.02519479 0.029121458 -

1.466240935

cg01663295 0.047260918 5.030385242 0.025293237 0.029221838

1.201323272

cg00240432 0.027815205 5.01045949 0.025583297 0.029519718 -
1.317938609

cg14019317 0.04259435 5.006270541 0.025644716 0.029566884 -
1.301534289

cg22796458 0.024825974 5.004042545 0.025677445 0.029601232
1.498125513

cg11428724 0.171085693 5.002426632 0.02570121 0.02962185 -
1.288634355

cg26388152 0.019191447 4.9990611090.02575078 0.029656156 1.2274752

cg13694867 0.024980924 4.999006947 0.025751579 0.029656156 -
1.263026236

cg18864581 0.07383506 4.990884368 0.025871629 0.02976719
1.217503527

cg21504624 0.07422185 4.990151384 0.025882491 0.029776287
1.205955032

cg23496260 0.076435479 4.98760918 0.025920201 0.029816265 -
1.311599347

cg01294695 0.062042631 4.98061967 0.026024174 0.029918788 -
1.219456

cg18678185 0.062980317 4.976040555 0.026092527 0.029982417
1.344066164

cg14137939	0.060152459	4.974871944	0.026110001	0.029996923	1.346764011
cg04425624	0.196700744	4.961731233	0.026307332	0.030202967	1.205444443
cg25742201	0.039903903	4.954193785	0.026421221	0.030309545	1.44101109
cg17561435	0.037908675	4.947184136	0.026527597	0.030417722	-1.225089577
cg19965810	0.051087683	4.937575046	0.026674146	0.030557941	-1.379445786
cg08233173	0.053003886	4.935159187	0.026711123	0.030589868	1.226522003
cg24497877	0.021317102	4.930642358	0.026780402	0.030648305	1.218601959
cg23959705	0.042542262	4.919552441	0.026951292	0.030819373	-1.237189846
cg21988041	0.034560109	4.908644353	0.02712049	0.03099175	-1.247160409
cg20632573	0.022227601	4.902164808	0.02722152	0.031093096	-1.228765025
cg04461705	0.052900838	4.895044308	0.027332997	0.031192138	1.264353593

cg24719601	0.130172664	4.89170211	0.027385486	0.031230053	1.299822445
cg03969906	0.061285623	4.891547384	0.027387918	0.031230053	1.276084462
cg19162158	0.021081028	4.891233427	0.027392855	0.031232148	-1.380322904
cg00002426	0.137128234	4.882473146	0.027530972	0.031371873	1.309367657
cg11304234	0.079223099	4.867800501	0.027763935	0.031619457	1.312658977
cg00265490	0.044350821	4.867538827	0.027768109	0.031619882	1.200447076
cg05056120	0.088974689	4.856202848	0.027949531	0.031802068	-1.210620734
cg02774439	0.112536603	4.848764272	0.02806925	0.031895066	-1.321030312
cg03734874	0.156503791	4.834914244	0.028293586	0.03211738	-1.244891853
cg19430897	0.131374588	4.827811726	0.028409355	0.032230638	1.253705907
cg10098888	0.031671084	4.805703521	0.028772886	0.032599019	1.752141798

cg24530795 0.060325342 4.803806464 0.028804305 0.032623611-
1.276720757

cg25234611 0.0196501114.796371032 0.028927797 0.032752433
1.234670628

cg24322623 0.101856967 4.78576981 0.029104826 0.03292327 -
1.250996186

cg02387679 0.257504364 4.782960554 0.029151927 0.032966911-
1.207808233

cg13378388 0.02630961 4.767042696 0.029420318 0.033220478 -
1.233733872

cg15379633 0.1113927134.766665763 0.029426705 0.033223966
1.240399706

cg25725843 0.076577658 4.766348275 0.029432085 0.033226318 -
1.204226303

cg02131967 0.025109698 4.765897323 0.029439729 0.033231224 -
1.379597443

cg27601582 0.1308774 4.754129018 0.029639952 0.033427273 -
1.315466459

cg11285843 0.05238465 4.753817165 0.029645277 0.033429537
1.208376086

cg02119229 0.018291959 4.752746509 0.029663567 0.033446418
1.268677062

cg08376864 0.036972584 4.748586068 0.02973475 0.033522928

1.321852118

cg25092328 0.027622615 4.748049844 0.029743938 0.033525784

1.948869626

cg09548179 0.02117479 4.738149328 0.029914106 0.033702507

1.858637482

cg02104644 0.094496182 4.736413771 0.029944041 0.033732461 -

1.216263778

cg08128768 0.046316223 4.701460321 0.030553634 0.034315586

1.688606011

cg10708675 0.037279265 4.695078992 0.030666318 0.034426795

1.263642553

cg09191327 0.045550795 4.693774151 0.030689413 0.034448883 -

1.370447582

cg17146877 0.037664163 4.689564587 0.030764044 0.034519854

1.213470044

cg05871607 0.02358013 4.688057032 0.030790818 0.034535777

1.261444749

cg12078929 0.026187224 4.686430176 0.030819737 0.034560519

1.884633089

cg09149294 0.012394205 4.682308127 0.030893139 0.034625568 -

1.307925486

cg19044674	0.034834198	4.682207798	0.030894928	0.034625568		1.270693552
cg19257550	0.198716396	4.678561887	0.030960008	0.034690789		1.210190369
cg01599709	0.048710995	4.67263088	0.031066183	0.034790413		1.206397966
cg26090652	0.053909884	4.66940259	0.031124135	0.034828287		-1.32806362
cg20616414	0.049014588	4.660113665	0.031291515	0.034988313		1.515245176
cg25302419	0.050205165	4.659639037	0.031300093	0.034994021		-1.319389612
cg22026853	0.03712395	4.656014954	0.03136567	0.035055668		-1.348337988
cg21842478	0.10388545	4.636618887	0.031719091	0.035389007		-1.32808303
cg12513481	0.091176211	4.636562782	0.031720119	0.035389007		-1.273748748
cg06797533	0.029635903	4.634839073	0.03175173	0.03541251		1.906042945
cg21421701	0.022725024	4.633429588	0.031777602	0.035437443		-1.210237366

cg17223189 0.016855494 4.632650542 0.0317919110.035446868

1.948260852

cg09047884 0.056583186 4.628368196 0.03187069 0.035517665

1.282145867

cg14013300 0.023367004 4.6201197060.032023007 0.035671629 -

1.230363866

cg13216303 0.012806914 4.6097514 0.03221555 0.035834606 2.02620339

cg13079099 0.034602462 4.596356401 0.032466095 0.036093374

1.754747733

cg12249575 0.02300309 4.595825925 0.032476059 0.036100468 -

1.211993478

cg18006568 0.1127550644.585643967 0.032667932 0.036289732

1.256940019

cg23432345 0.178793247 4.575598415 0.032858398 0.036475238 -

1.273792282

cg07175883 0.104883623 4.575500261 0.032860265 0.036475238 -

1.308359364

cg12448933 0.09378984 4.56748115 0.033013153 0.036624771 -

1.305552136

cg07314414 0.105953033 4.563668894 0.033086097 0.036701653

1.245468153

cg12998614 0.023086309 4.5551156410.033250369 0.036859529

1.216706835

cg25957124 0.093338845 4.549669214 0.033355418 0.036943462

1.285659472

cg16042149 0.130413439 4.539137925 0.033559528 0.037116488-

1.246899376

cg03891319 0.032131414 4.536999662 0.03360113 0.037154342

1.290331689

cg21303011 0.025550051 4.528985988 0.033757525 0.037307947

1.674746516

cg01803059 0.1122018944.528633274 0.033764426 0.037310339

1.223908035

cg11670211 0.055702293 4.516366128 0.034005363 0.0375313 -

1.329007384

cg10549973 0.021738707 4.510652532 0.0341181970.037635221

1.233582326

cg02973416 0.0200112214.507568796 0.034179259 0.037690198

1.64450253

cg09238598 0.026958121 4.497034561 0.034388715 0.03789214

1.319427126

cg02248486 0.154571537 4.492502731 0.034479237 0.037983515

1.223423477

cg14696396 0.039290758 4.486207239 0.034605401 0.038105898 -

1.289866738

cg12091331 0.1142879224.479403614 0.034742294 0.038223219

1.232333276

cg03169527 0.027604157 4.478732091 0.034755836 0.038233943

1.816060807

cg16041611 0.043460369 4.477887026 0.034772886 0.038244348

1.253973468

cg00343092 0.014363695 4.47361306 0.034859251 0.038305883

1.44376444

cg01143454 0.050133545 4.470166009 0.034929069 0.038365868

1.238772989

cg01401376 0.03914875 4.468857824 0.034955605 0.038390829 -

1.310184023

cg16090392 0.033325531 4.463352227 0.035067512 0.038496949

1.218302368

cg01988129 0.095087991 4.456209524 0.035213256 0.03861576

1.45053707

cg06385087 0.014616677 4.447857814 0.035384475 0.038773091

2.169166822

cg17740399 0.027402252 4.438987513 0.035567282 0.038931066 -

1.372061775

cg17277529 0.025409279 4.438228391 0.035582973 0.03894401

1.2208585

cg09688546 0.026028301 4.43797147 0.035588285 0.038945594

1.343873648

cg18436172 0.052205248 4.434512606 0.035659881 0.039019706

1.312280661

cg03405173 0.02303427 4.431269335 0.035727152 0.039084826

1.201581806

cg06268694 0.122917163 4.430333184 0.035746594 0.039101849

1.308794983

cg12109455 0.092902179 4.421684548 0.035926736 0.039269057

1.319806361

cg01031400 0.05527835 4.42002895 0.035961329 0.039302604 -

1.337128084

cg05832051 0.03190262 4.418013351 0.036003491 0.039340149 -

1.215226789

cg15334028 0.050897912 4.416745235 0.036030044 0.039360626

1.249237615

cg01126560 0.098168401 4.4149101180.036068506 0.039392841

1.287042513

cg12532500 0.032356449 4.414337669 0.036080512 0.039398672

1.413725296

cg24801210 0.062609639 4.412961387 0.036109396 0.039425939

1.271896592

cg05820087 0.123845335 4.399452044 0.036394202 0.039672417

1.209118471

cg27286999 0.026921245 4.3945110480.036498958 0.039778001

1.463189827

cg24517609 0.037303998 4.379696905 0.036814938 0.040061702

1.217483112

cg12045002 0.080780963 4.376997953 0.036872814 0.040103486 -

1.255312609

cg24794433 0.0475031174.376978255 0.036873237 0.040103486

1.672570413

cg12622986 0.095996463 4.375014342 0.0369154110.040136357

1.30080322

cg27092035 0.04080551 4.373697795 0.036943712 0.040158461 -

1.310997155

cg09088508 0.017135574 4.3711846150.0369978 0.040195439

1.990250971

cg08965235 0.096009132 4.3661182560.037107089 0.040279561

1.202175154

cg26091679 0.033917277 4.365862563 0.0371126130.040281218

1.251422499

cg20802051 0.0525061134.362990364 0.037174731 0.040329508

1.282557336

cg02829654 0.083412458 4.3540511750.037368761 0.040498159

1.272637146

cg27096144 0.077667974 4.353521233 0.037380297 0.040506304

1.435308996

cg22674717 0.013285602 4.347632232 0.037508744 0.040619281

1.201839602

cg01557989 0.019593849 4.346419481 0.037535254 0.040639253 -

1.247308371

cg11673092 0.052695987 4.327293377 0.03795594 0.041015395 -

1.302087057

cg18689253 0.036827861 4.326786544 0.037967155 0.041023114-

1.28045897

cg14225544 0.023242224 4.324891092 0.038009129 0.041046457

1.298598044

cg12696750 0.046834741 4.315710446 0.0382131180.041231394

1.247739046

cg20159072 0.009762091 4.294063916 0.038698663 0.041670503

2.661028555

cg27146152 0.020412465 4.292558763 0.038732665 0.041700349

1.431474376

cg07265300 0.028552979 4.289266321 0.038807152 0.041765005

1.648746616

cg22131691 0.147347244 4.2856219 0.038889777 0.041836053 -

1.239195381

cg18292394 0.120941447 4.2805721 0.039004571 0.041937156

1.28365605

cg10409680 0.058466578 4.278058233 0.03906185 0.04199426

1.301527954

cg04265576 0.126330078 4.266905798 0.039317029 0.042237048 -

1.26209312

cg13801381 0.051753396 4.264461976 0.03937318 0.042288352 -

1.337269462

cg13697387 0.099848034 4.255462793 0.03958068 0.04248162

1.291120901

cg18197795 0.017937505 4.241816707 0.039897525 0.042766928

1.422285888

cg17371081 0.06050402 4.241735742 0.039899413 0.042766928 -

1.326139115

cg07322981 0.018259213 4.230235477 0.04016852 0.043014192

1.211353072

cg16474684 0.058502341 4.225494778 0.040280009 0.043113034-

1.296920765

cg07935568 0.132935057 4.223561725 0.040325562 0.043149747 -

1.223842341

cg08687825 0.087347271 4.216891014 0.040483179 0.043273696 -

1.277807218

cg23596123 0.223154048 4.21239379 0.040589806 0.04336393

1.201368308

cg18244915 0.025837329 4.2113984560.040613444 0.043375555 -

1.219982843

cg10523019 0.137223965 4.2113918260.040613602 0.043375555 -

1.252298249

cg04315264 0.02160445 4.1918654 0.041080293 0.04379642 1.484159456

cg03409548 0.050622097 4.1899811020.0411256260.0438341181.228333867

cg08398233 0.165558121 4.18779757 0.0411782250.043876256

1.223077341

cg19904653 0.126902171 4.185530163 0.041232919 0.0439206

1.21305801

cg20647888 0.123692489 4.177433809 0.041428843 0.044110641

1.202446121

cg14449051 0.030982262 4.172321286 0.041553066 0.044210203 -

1.328963471

cg10723020 0.06409405 4.169021723 0.041633447 0.044253666 -

1.217828132

cg16703647 0.038607178 4.167933784 0.041659986 0.04426882 -

1.340528303

cg19615059 0.0374071184.162588449 0.041790639 0.044383293 -

1.2341658

cg16112129 0.137309028 4.154968313 0.041977641 0.044544334 -

1.206171544

cg21644826 0.04525779 4.1540101140.042001218 0.044559966

1.224903181

cg10217449 0.052572567 4.146268558 0.042192216 0.044729631

1.243713702

cg09019938 0.036929029 4.127432173 0.042660777 0.045136136 -

1.36029113

cg19428336 0.022668024 4.125736745 0.042703219 0.045169624

1.472918357

cg25823578 0.057367396 4.125630616 0.042705877 0.045169624 -

1.314043525

cg08996521 0.04785905 4.1139788770.042998788 0.045436506 -

1.213022108

cg03506799 0.012469404 4.1130484570.043022269 0.045456551 -

1.200200879

cg21516384 0.049339318 4.1122547090.0430423110.045468191 -

1.207625746

cg11846236 0.093557005 4.1114429260.043062819 0.045483116-

1.266528141

cg20047732 0.02388236 4.1101229620.043096187 0.045496599 1.34385331

cg01513661 0.098166555 4.108006167 0.043149756 0.045538721

1.228485133

cg05654164 0.1153413854.10549168 0.043213479 0.045588251

1.211521698

cg03879902 0.0911870264.105440167 0.043214786 0.045588251 -

1.204049986

cg17491456 0.1162951134.103096648 0.043274268 0.045641443

1.204979341

cg16127845 0.026705981 4.094873422 0.043483671 0.045828719

1.663074193

cg24884084 0.133846238 4.076528469 0.043954684 0.046242902

1.206600649

cg18342279 0.021395047 4.074314522 0.0440118910.046284798

1.729976424

cg07758574 0.050438928 4.071667807 0.044080383 0.046326772

1.417012489

cg08558873 0.028254715 4.061775222 0.044337384 0.046538618 -

1.379492354

cg05705583 0.02655138 4.061083347 0.044355418 0.046552697

1.28078728

cg11467738 0.024256255 4.055057974 0.044512794 0.046688689

1.213355022

cg21178548 0.103300396 4.045213266 0.0447711960.046925528

1.227848041

cg09929612 0.01190186 4.039841212 0.044912869 0.047064226

1.830617199

cg05970790 0.026690003 4.02418752 0.045328397 0.047421024

1.331928011

cg09553358 0.09701501 4.022036693 0.045385807 0.047451245 -

1.293061481

cg05899618 0.149074733 4.021289454 0.04540577 0.047467191 -

1.235259997

cg05307923 0.044024954 4.0024401190.045912434 0.047892506 -

1.316311731

cg11398517 0.156022856 3.999452784 0.04599328 0.047966906

1.205534579

cg04420907 0.031309539 3.998837836 0.04600994 0.047979315

1.802638379

cg21599792 0.016944866 3.993938616 0.046142903 0.048098057

1.232859421

cg08949296 0.0223011743.989844996 0.046254314 0.048179537 -

1.250590699

cg13274713 0.01865712 3.989048326 0.046276029 0.048196934 -

1.282339773

cg15839448 0.088849565 3.985078876 0.046384386 0.048279844 -

1.274542422

cg19714749 0.019648001 3.983199588 0.046435779 0.048318363 -

1.277875299

cg08993267 0.128287959 3.982777426 0.046447333 0.048325394 -

1.231794034

cg00297584 0.060456307 3.979638966 0.046533318 0.048399863 -

1.222022314

cg16638540 0.064220172 3.963241647 0.046985307 0.048799455

1.391996409

cg08260959 0.1159729743.953467476 0.047256936 0.049031029

1.403805977

cg05449414 0.029371838 3.946388592 0.047454698 0.049226078

1.406005124

cg11724134 0.014755542 3.93361576 0.047813751 0.049527146

1.475476458

cg22224704 0.087781844 3.928230312 0.047965999 0.049654222

1.212435291

cg26538116 0.019747709 3.927835443 0.047977183 0.049660697

1.605273614

cg25432997	0.019995273	3.924307995	0.048077208	0.049733576	1.230296733
cg18925884	0.037328712	3.923481422	0.048100678	0.04974764	1.321577628

Supplementary table 4. 1877 CpGb were matched to the corresponding genes

	chr	Name	Probe_rs	Islands_Name	Relation_to_Island	Methyl27_Loci	UCSC_RefGene_Name	UCSC_RefGene_Group
cg00514407	chr2	cg00514407	rs115531623	chr2:224903221-224904735	Island	TRUE	SERPINE2	TSS1500
cg16979445	chr6	cg16979445	NA		OpenSea	TRUE	NRM	TSS1500
cg00626466	chr12	cg00626466	NA	chr12:65152869-65153489	S_Shore	TRUE	GNS	TSS1500
cg01531431	chr20	cg01531431	NA	chr20:62151045-62152847	N_Shore	TRUE	PPDPF	TSS1500
cg17641252	chr14	cg17641252	NA	chr14:67878534-67879167	Island	TRUE	PLEK2	Body
cg16858125	chr1	cg16858125	NA	chr1:43832814-43833073	S_Shore	TRUE	ELOVL1	TSS1500
cg09816471	chr16	cg09816471	NA	chr16:11761859-11762127	Island	TRUE	SNN	TSS1500
cg18236734	chr3	cg18236734	rs34611203		OpenSea	TRUE	HTR3E	TSS200
cg15046693	chr19	cg15046693	NA	chr19:33864176-33865010	N_Shore	TRUE	CEBPG	TSS1500
cg09492887	chr7	cg09492887	rs77333559	chr7:103085710-103086132	Island	TRUE	SLC26A5	5'UTR
cg26174752	chr6	cg26174752	NA	chr6:97284830-97285782	S_Shore	TRUE	GPR63	TSS1500
cg17240454	chr6	cg17240454	NA		OpenSea	TRUE	SPDEF	TSS200

cg21184174 chr1 cg21184174 rs11466067 chr1:115880167-115881332 Island TRUE NGF TSS1500
cg24926276 chr19 cg24926276 NA chr19:4543407-4544578 N_Shelf TRUE LRG1 Body
cg17644208 chr2 cg17644208 NA chr2:25142472-25143689 N_Shore TRUE ADCY3 TSS1500
cg18390025 chr10 cg18390025 NA chr10:103985852-103986246 S_Shore TRUE ELOVL3 Body
cg22809047 chr2 cg22809047 NA chr2:101618247-101618658 Island TRUE RPL31 TSS1500
cg03866607 chr17 cg03866607 rs3760368 chr17:59531723-59535254 Island TRUE TBX4 TSS1500
cg21541083 chr19 cg21541083 NA chr19:7701661-7702336 N_Shore TRUE STXBP2 TSS1500
cg21762589 chr10 cg21762589 NA chr10:133794898-133796598 Island TRUE BNIP3 TSS1500
cg20422318 chr9 cg20422318 NA chr9:134954960-134955335 Island TRUE MED27 1stExon
cg17356733 chr21 cg17356733 NA chr21:34775437-34776493 N_Shore TRUE IFNGR2 TSS1500
cg03294491 chr18 cg03294491 NA chr18:45456484-45458024 S_Shore TRUE SMAD2 TSS1500
cg18486150 chr1 cg18486150 NA chr1:21043832-21044771 S_Shore TRUE KIF17 TSS1500
cg07080946 chr16 cg07080946 NA chr16:281088-281316 N_Shore TRUE LUC7L TSS1500
cg02477931 chr11 cg02477931 NA OpenSea TRUE NTM Body
cg19728382 chr5 cg19728382 NA chr5:172754056-172757098 S_Shore TRUE STC2 TSS1500
cg18674980 chr8 cg18674980 NA chr8:86350765-86351196 N_Shore TRUE CA3 TSS1500
cg25645462 chr16 cg25645462 NA OpenSea TRUE GPR56 TSS1500
cg02889982 chr17 cg02889982 rs138437542 chr17:16945384-16946317 Island

TRUE MPRIP 1stExon
cg03387723 chr1 cg03387723 NA chr1:41706957-41708327 S_Shore TRUE
SCMH1 TSS1500
cg08465774 chr8 cg08465774 NA OpenSea TRUE IDO1 Body
cg12461141 chr11 cg12461141 NA OpenSea TRUE TRIM22
TSS1500
cg13320683 chr10 cg13320683 NA chr10:62761163-62761595 S_Shore TRUE
RHOBTB1 TSS1500
cg22584138 chr17 cg22584138 NA chr17:28562387-28563186 N_Shore
TRUE SLC6A4 5'UTR
cg07715201 chr5 cg07715201 NA chr5:37836747-37840726 S_Shore TRUE
GDNF TSS1500
cg13474734 chr15 cg13474734 NA OpenSea TRUE C15orf52 Body
cg26453588 chr22 cg26453588 NA chr22:43505951-43506167 Island TRUE
BIKTSS1500
cg05113558 chr8 cg05113558 NA chr8:145703038-145703902 N_Shore
TRUE FOXH1 TSS1500
cg11147193 chr2 cg11147193 NA chr2:10442308-10444509 N_Shore TRUE
HPCAL1 TSS1500
cg18741908 chr3 cg18741908 rs2287483 chr3:169755594-169756847 Island
TRUE GPR1605'UTR
cg18493147 chr19 cg18493147 NA OpenSea TRUE ODF3L2 Body
cg16302441 chr2 cg16302441 NA chr2:25391072-25391875 Island TRUE
POMC TSS1500
cg04049033 chr17 cg04049033 NA chr17:1551730-1553249 S_Shore TRUE
RILP TSS1500
cg00509616 chr15 cg00509616 NA chr15:57883998-57884358 S_Shore TRUE
GCOM1 Body
cg21794225 chr14 cg21794225 NA chr14:30396210-30397420 S_Shore TRUE
PRKD1 TSS1500
cg00347904 chr6 cg00347904 NA chr6:35181095-35182547 Island TRUE
SCUBE3 Body
cg06550629 chr12 cg06550629 NA OpenSea TRUE GPR133 Body

cg15210999 chr6 cg15210999 NA chr6:15662444-15663412 S_Shore TRUE DTNBP1 TSS1500
cg20395892 chr12 cg20395892 NA chr12:66582695-66583345 Island TRUE IRAK3 TSS200
cg16003238 chr15 cg16003238 NA chr15:65669310-65670617 S_Shore TRUE IGDCC3 TSS1500
cg19562969 chr20 cg19562969 rs12329595 chr20:35233829-35234782 N_Shore TRUE C20orf24 TSS1500
cg21960110 chr16 cg21960110 NA chr16:203753-204866 N_Shore TRUE HBZ TSS1500
cg22919728 chr3 cg22919728 NA chr3:126242783-126243358 N_Shore TRUE CHST13 TSS1500
cg22449114 chr20 cg22449114 NA chr20:590222-591222 Island TRUE TCF15 Body
cg13603171 chr6 cg13603171 rs7772445 chr6:132722093-132722901 S_Shore TRUE MOXD1 TSS1500
cg15101633 chr14 cg15101633 NA chr14:101293427-101294433 Island TRUE MEG3 Body
cg08996748 chr15 cg08996748 NA chr15:40650055-40651102 Island TRUE DISP2 Body
cg13678049 chr6 cg13678049 NA chr6:43149736-43150009 N_Shore TRUE CUL9 TSS1500
cg24831427 chr6 cg24831427 NA chr6:99841661-99842146 S_Shore TRUE COQ3 TSS1500
cg23679724 chr20 cg23679724 NA chr20:57581902-57582595 N_Shore TRUE CTSZ Body
cg00016968 chr1 cg00016968 rs3125045 chr1:113249342-113250198 S_Shore TRUE RHOC TSS1500
cg21096399 chr11 cg21096399 NA chr11:119186947-119187894 S_Shore TRUE MCAM TSS1500
cg18149919 chr8 cg18149919 NA chr8:144911055-144912075 Island TRUE PUF60 TSS1500
cg03699904 chr3 cg03699904 NA chr3:170746072-170746292 N_Shore

TRUE SLC2A2 TSS1500
cg26527984 chr17 cg26527984 NA chr17:73760694-73761269 S_Shore TRUE GALK1 TSS1500
cg09205751 chr11 cg09205751 NA chr11:279072-281700 N_Shore TRUE NLRP6 TSS200
cg12770741 chr17 cg12770741 NA chr17:881733-883381 S_Shore TRUE NXN TSS1500
cg00461841 chr16 cg00461841 NA OpenSea TRUE ATF7IP2 5'UTR
cg25549459 chr2 cg25549459 NA chr2:105468851-105473488 Island TRUE POU3F3 TSS1500
cg09134726 chr19 cg09134726 rs62132296 chr19:843468-844085 N_Shelf TRUE PRTN3 Body
cg07586911 chr7 cg07586911 NA OpenSea TRUE FAM71F1 1stExon
cg07773116 chr10 cg07773116 NA chr10:48438411-48439320 Island TRUE GDF10 1stExon
cg05293216 chr9 cg05293216 NA chr9:35079628-35080213 S_Shore TRUE FANCG TSS1500
cg20648149 chr14 cg20648149 NA chr14:64319622-64320526 N_Shore TRUE SYNE2 TSS1500
cg01693350 chr11 cg01693350 NA chr11:32452144-32452708 Island TRUE WT1 Body
cg04513422 chr13 cg04513422 NA OpenSea TRUE C13orf29 Body
cg15284635 chr3 cg15284635 NA chr3:10857687-10858447 S_Shore TRUE SLC6A11 Body
cg05868799 chr8 cg05868799 NA chr8:71314145-71316770 S_Shore TRUE NCOA2 TSS1500
cg24621042 chr14 cg24621042 NA OpenSea TRUE SERPINA1 TSS1500
cg25201363 chr4 cg25201363 NA chr4:3533643-3534788 S_Shore TRUE LRPAP1 TSS1500
cg22424746 chr1 cg22424746 NA OpenSea TRUE VTCN1 Body
cg09143663 chr21 cg09143663 NA chr21:30670744-30671786 N_Shore

TRUE BACH1 TSS1500
cg06142324 chr11 cg06142324 NA OpenSea TRUE HEPACAM Body
cg05445326 chr3 cg05445326 NA OpenSea TRUE TM4SF19
TSS1500
cg21745164 chr16 cg21745164 NA chr16:23765969-23766869 N_Shore
TRUE CHP2 TSS1500
cg15479752 chr19 cg15479752 NA OpenSea TRUE FFAR2 1stExon
cg10612997 chr2 cg10612997 NA OpenSea TRUE GREB1 TSS1500
cg06469542 chr14 cg06469542 NA chr14:77964571-77965670 S_Shore TRUE
ISM2 TSS1500
cg13689073 chr5 cg13689073 NA OpenSea TRUE LARP1 Body
cg06154570 chr1 cg06154570 NA chr1:40105010-40105707 S_Shore TRUE
HEYL TSS1500
cg07156669 chr17 cg07156669 NA chr17:28705363-28706818 N_Shore
TRUE CPD TSS1500
cg20790056 chr19 cg20790056 NA chr19:4304542-4305122 N_Shore TRUE
TMIGD2 TSS1500
cg04590978 chr10 cg04590978 NA chr10:97802871-97804262 Island TRUE
CCNJ TSS1500
cg17259265 chr11 cg17259265 NA chr11:125757055-125758107 N_Shelf TRUE
HYLS1 TSS200
cg01892689 chr8 cg01892689 NA chr8:97657174-97657943 N_Shore TRUE
PGCP TSS1500
cg17714030 chr16 cg17714030 NA chr16:230301-231761 N_Shore TRUE
HBQ1 TSS1500
cg03599338 chr22 cg03599338 NA OpenSea TRUE SUSD2 TSS200
cg25195673 chr2 cg25195673 NA chr2:69614119-69614616 S_Shore TRUE
GFPT1 TSS1500
cg24127989 chr7 cg24127989 NA chr7:128049499-128050488 Island TRUE
IMPDH1 Body
cg23239444 chr6 cg23239444 NA chr6:34433411-34434371 N_Shore TRUE
PACSIN1 TSS1500
cg11277230 chr4 cg11277230 NA chr4:87813172-87813718 S_Shore TRUE

C4orf36 TSS1500
cg25683185 chr12 cg25683185 NA chr12:6756001-6756679 Island TRUE ACRBP Body
cg18429742 chr5 cg18429742 rs60003992 chr5:848389-848770 S_Shelf TRUE ZDHHC11 TSS200
cg04747322 chr5 cg04747322 NA chr5:121647450-121648147 N_Shore TRUE SNCAIP TSS1500
cg23555120 chr12 cg23555120 NA chr12:106532106-106533696 S_Shore TRUE NUAK1 TSS200
cg18401406 chr13 cg18401406 NA chr13:37247949-37248463 N_Shore TRUE C13orf36 TSS1500
cg22233974 chr1 cg22233974 NA chr1:39547013-39547959 N_Shore TRUE MACF1 TSS1500
cg01356829 chr1 cg01356829 NA chr1:67773329-67773767 N_Shore TRUE IL12RB2 TSS200
cg19317715 chr17 cg19317715 rs35154433 OpenSea TRUE AOC2 TSS200
cg06100324 chr16 cg06100324 NA OpenSea TRUE MSLN 5'UTR
cg19279346 chr19 cg19279346 NA OpenSea TRUE LILRB2 TSS1500
cg06048973 chr15 cg06048973 NA OpenSea TRUE ACTC1 TSS200
cg04001668 chr16 cg04001668 NA OpenSea TRUE GPR56 5'UTR
cg27625732 chr9 cg27625732 rs74586890 chr9:131549294-131549546 N_Shore TRUE TBC1D13 TSS1500
cg09462826 chr11 cg09462826 NA chr11:61582573-61584728 S_Shore TRUE FADS1 TSS1500
cg17518825 chr2 cg17518825 NA chr2:46523789-46527140 N_Shore TRUE EPAS1 TSS1500
cg19433435 chr11 cg19433435 NA chr11:125365137-125366319 S_Shore TRUE FEZ1 TSS1500
cg06874144 chr2 cg06874144 NA chr2:231577309-231578504 N_Shore TRUE CAB39 TSS1500
cg04089739 chr19 cg04089739 NA OpenSea TRUE C3 Body
cg05600717 chr13 cg05600717 NA chr13:52377821-52378573 S_Shore TRUE

DHRS12 TSS1500

cg18793806 chr2 cg18793806 NA chr2:95824802-95825721 S_Shore TRUE ZNF514 TSS1500

cg23502772 chr5 cg23502772 NA chr5:43191974-43193155 Island TRUE MGC42105 TSS1500

cg01777397 chr2 cg01777397 NA chr2:29337983-29338909 N_Shore TRUE CLIP4 TSS1500

cg14170423 chr11 cg14170423 NA chr11:64509433-64513826 Island TRUE RASGRP2 TSS1500

cg15988232 chr3 cg15988232 NA chr3:47619112-47621131 Island TRUE CSPG5 TSS1500

cg15901783 chr13 cg15901783 NA chr13:77459322-77461099 S_Shore TRUE KCTD12 TSS1500

cg02335804 chr17 cg02335804 NA chr17:36105334-36105583 N_Shore TRUE HNF1B TSS200

cg14925024 chr1 cg14925024 NA chr1:228289669-228291184 S_Shore TRUE C1orf35 TSS1500

cg10631471 chr16 cg10631471 NA chr16:48278058-48278655 S_Shore TRUE LONP2 Body

cg12058490 chr12 cg12058490 NA OpenSea TRUE A2M 1stExon

cg03693099 chr9 cg03693099 rs78792036 OpenSea TRUE CEL TSS1500

cg16907566 chr8 cg16907566 NA chr8:121137099-121137778 N_Shore TRUE COL14A1 TSS1500

cg02831604 chr15 cg02831604 rs73448114 chr15:76628998-76629207 N_Shore TRUE ISL2 TSS1500

cg22289837 chr8 cg22289837 NA chr8:86350765-86351196 N_Shore TRUE CA3 TSS1500

cg07785936 chr2 cg07785936 NA chr2:133175160-133175444 N_Shore TRUE GPR39 1stExon

cg06607866 chr11 cg06607866 NA chr11:67250344-67250864 Island TRUE AIP Body

cg19740375 chr3 cg19740375 NA chr3:38690409-38691513 Island TRUE

SCN5A 5'UTR
cg05406101 chr21 cg05406101 NA chr21:30391264-30391758 S_Shore TRUE
RWDD2B TSS1500
cg13701109 chr5 cg13701109 NA chr5:128795503-128797417 N_Shore
TRUE ADAMTS19 TSS1500
cg18168989 chr9 cg18168989 NA chr9:77642704-77643595 S_Shore TRUE
C9orf41 TSS1500
cg17177660 chr4 cg17177660 NA chr4:177713243-177714457 Island TRUE
VEGFC TSS1500
cg07408740 chr7 cg07408740 NA chr7:44646065-44646823 N_Shore TRUE
OGDH TSS1500
cg12788467 chr17 cg12788467 NA chr17:36105334-36105583 Island TRUE
HNF1B TSS1500
cg20367961 chr1 cg20367961 NA OpenSea TRUE EDN2 1stExon
cg13641903 chr11 cg13641903 NA chr11:32452144-32452708 Island TRUE
WT1 Body
cg16393207 chr11 cg16393207 NA chr11:75236189-75237781 Island TRUE
GDPD5 TSS1500
cg15534366 chr20 cg15534366 NA chr20:59826977-59828978 N_Shore
TRUE CDH4 TSS1500
cg21623671 chr5 cg21623671 NA chr5:150537019-150537418 N_Shore
TRUE ANXA6 5'UTR
cg08952029 chr11 cg08952029 rs11236236 chr11:74442260-74442874
S_Shore TRUE CHRDL2 TSS1500
cg00221494 chr13 cg00221494 NA chr13:98794398-98796241 Island TRUE
FARP1 TSS1500
cg10225525 chr14 cg10225525 NA chr14:65878486-65880370 N_Shore
TRUE FUT8 TSS1500
cg09458237 chr20 cg09458237 NA chr20:3713159-3713515 N_Shore TRUE
HSPA12B TSS1500
cg26847866 chr8 cg26847866 NA chr8:27490959-27491775 S_Shore TRUE
SCARA3 Body
cg09486093 chr5 cg09486093 NA chr5:118788125-118788428 N_Shore

TRUE HSD17B4 TSS200
cg08221207 chr11 cg08221207 NA chr11:125365137-125366319 S_Shore TRUE
FEZ1 TSS1500
cg11935638 chr3 cg11935638 NA chr3:184971641-184972002 S_Shore TRUE
EHHADH TSS1500
cg13523557 chr7 cg13523557 NA chr7:45613386-45615504 Island TRUE
ADCY1 TSS1500
cg01566404 chr1 cg01566404 NA chr1:204120398-204121109 S_Shore TRUE
ETNK2 TSS1500
cg10523671 chr3 cg10523671 NA OpenSea TRUE SLC15A2
TSS1500
cg16869108 chr3 cg16869108 NA chr3:10183305-10183941 S_Shore TRUE
VHL Body
cg23640701 chr12 cg23640701 NA chr12:52300801-52301583 S_Shore TRUE
ACVRL1 5'UTR
cg18137704 chr22 cg18137704 NA chr22:17600563-17602611 S_Shore TRUE
CECR6 TSS1500
cg01269795 chr6 cg01269795 NA OpenSea TRUE BTN3A3
TSS1500
cg25040733 chr5 cg25040733 NA chr5:141703749-141705023 Island TRUE
SPRY4 TSS200
cg05023691 chr1 cg05023691 NA OpenSea TRUE RGS13 TSS200
cg16501028 chr11 cg16501028 NA chr11:32448261-32449744 S_Shore TRUE
WT1 Body
cg27285720 chr1 cg27285720 NA OpenSea TRUE GBP4 TSS200
cg03032025 chr5 cg03032025 NA chr5:173314931-173315979 S_Shore TRUE
CPEB4 1stExon
cg05564266 chr6 cg05564266 NA chr6:118971662-118973137 S_Shore TRUE
C6orf204 TSS1500
cg18081258 chr14 cg18081258 NA chr14:21492735-21494270 Island TRUE
NDRG2 TSS1500
cg20939319 chr8 cg20939319 NA OpenSea TRUE TEX15 TSS1500
cg02250594 chr18 cg02250594 NA chr18:55103154-55108853 Island TRUE

ONECUT2 1stExon
cg10954182 chr12 cg10954182 rs115551928 chr12:104531174-104532539 Island TRUE NFYB TSS1500
cg07027513 chr18 cg07027513 NA chr18:29264703-29265504 S_Shore TRUE B4GALT6 TSS1500
cg19420968 chr1 cg19420968 NA chr1:32083149-32084069 S_Shore TRUE HCRTR1 Body
cg15544036 chr7 cg15544036 rs8629 chr7:73245434-73246045 Island TRUE CLDN4 1stExon
cg04230060 chr9 cg04230060 NA chr9:114937020-114937824 S_Shore TRUE SUSD1 TSS1500
cg15329642 chr1 cg15329642 NA chr1:45308543-45309159 N_Shore TRUE PTCH2 Body
cg24087944 chr1 cg24087944 NA chr1:1950411-1951479 N_Shore TRUE GABRD TSS1500
cg17655614 chr16 cg17655614 NA chr16:68771034-68772344 N_Shore TRUE CDH1 TSS1500
cg01718139 chr19 cg01718139 NA OpenSea TRUE VSTM1 Body
cg21301440 chr17 cg21301440 NA chr17:74533281-74534566 Island TRUE CYGB TSS1500
cg26203861 chr1 cg26203861 NA OpenSea TRUE SHISA4 Body
cg09259772 chr7 cg09259772 rs116561187 chr7:12726156-12727248 N_Shore TRUE ARL4A TSS1500
cg15446391 chr11 cg15446391 NA chr11:32452144-32452708 Island TRUE WT1 Body
cg01888601 chr11 cg01888601 NA chr11:47736739-47737106 N_Shore TRUE AGBL2 1stExon
cg02930996 chr18 cg02930996 NA chr18:29264703-29265504 S_Shore TRUE B4GALT6 TSS1500
cg12629244 chr1 cg12629244 NA chr1:178994804-178995917 N_Shore TRUE FAM20B TSS1500
cg11695358 chr8 cg11695358 NA chr8:144798487-144799038 N_Shore TRUE MAPK15 TSS1500

cg20001829 chr11 cg20001829 NA chr11:118401235-118402069 S_Shore TRUE TMEM25 5'UTR
cg20340596 chr10 cg20340596 NA OpenSea TRUE HPS1 TSS1500
cg23851011 chr1 cg23851011 NA OpenSea TRUE S100A16 TSS1500
cg26453670 chr20 cg26453670 NA chr20:55966212-55966964 N_Shore TRUE RBM38 TSS1500
cg00135056 chr3 cg00135056 NA chr3:128997382-128997600 N_Shore TRUE C3orf37 TSS1500
cg11204562 chr10 cg11204562 NA OpenSea TRUE C10orf81 TSS200
cg20324165 chr12 cg20324165 NA chr12:53297442-53297824 S_Shore TRUE KRT8 TSS1500
cg24833277 chr8 cg24833277 rs79026486 OpenSea TRUE FAM83A TSS1500
cg02275294 chr1 cg02275294 NA chr1:179262771-179263321 N_Shore TRUE SOAT1 TSS1500
cg01414934 chr1 cg01414934 NA chr1:26606362-26606913 N_Shore TRUE SH3BGRL3 TSS1500
cg08145177 chr19 cg08145177 NA chr19:17413733-17414486 Island TRUE ABHD8 5'UTR
cg15241708 chr5 cg15241708 NA chr5:179921201-179922179 N_Shore TRUE CNOT6 TSS1500
cg03381111 chr15 cg03381111 NA OpenSea TRUE SNORD116-1 TSS200
cg25151806 chr6 cg25151806 NA chr6:11043913-11045206 S_Shore TRUE ELOVL2 TSS1500
cg01033938 chr11 cg01033938 NA chr11:130297401-130298517 S_Shore TRUE ADAMTS8 TSS1500
cg06781209 chr11 cg06781209 NA chr11:61594996-61596710 Island TRUE FADS2 TSS1500
cg15821095 chr3 cg15821095 NA chr3:51428412-51429625 N_Shore TRUE RBM15B TSS1500

cg08268099 chr9 cg08268099 NA chr9:137967110-137967727 N_Shore TRUE OLFM1 TSS1500
cg21614638 chr4 cg21614638 NA OpenSea TRUE DAPP1 TSS200
cg00489401 chr5 cg00489401 NA chr5:180075688-180076906 Island TRUE FLT4 Body
cg00186701 chr8 cg00186701 NA chr8:98289604-98290404 S_Shore TRUE TSPYL5 TSS1500
cg17191178 chr3 cg17191178 NA chr3:157822973-157823836 S_Shore TRUE SHOX2 TSS1500
cg07799947 chr2 cg07799947 NA chr2:101434979-101437453 Island TRUE NPAS2 TSS1500
cg01573562 chr12 cg01573562 NA OpenSea TRUE RPH3A TSS1500
cg06488678 chr19 cg06488678 NA OpenSea TRUE TMPRSS9 Body
cg01281904 chr8 cg01281904 rs3808627 chr8:54163303-54164443 S_Shore TRUE OPRK1 TSS1500
cg24459209 chr11 cg24459209 rs669661 OpenSea TRUE PRG3 5'UTR
cg07297178 chr19 cg07297178 rs10409040 OpenSea TRUE CEACAM7 TSS1500
cg00231644 chr6 cg00231644 NA chr6:3156959-3157167 S_Shore TRUE TUBB2A TSS1500
cg06851207 chr19 cg06851207 rs8107491 chr19:46974557-46975073 S_Shore TRUE PNMAL1 TSS1500
cg07294734 chr19 cg07294734 NA chr19:1241619-1242569 Island TRUE ATP5D Body
cg03152385 chr16 cg03152385 NA chr16:15187935-15188329 S_Shore TRUE RRN3 TSS1500
cg14566624 chr1 cg14566624 NA chr1:6320137-6320942 S_Shore TRUE GPR153 TSS1500
cg17612991 chr19 cg17612991 NA OpenSea TRUE C3 TSS1500
cg26683023 chr1 cg26683023 NA chr1:227751409-227751635 N_Shore TRUE ZNF678 TSS1500
cg18170080 chr11 cg18170080 NA chr11:124632063-124633239 N_Shore

TRUE ESAM 1stExon
cg19005210 chr6 cg19005210 NA OpenSea TRUE TREML2
TSS200
cg18952647 chr15 cg18952647 NA chr15:83951980-83953930 Island TRUE
BNC1 TSS1500
cg07185695 chr1 cg07185695 NA chr1:220960016-220960603 N_Shore
TRUE MOSC1 TSS1500
cg27494383 chr15 cg27494383 NA chr15:41803443-41805531 S_Shore TRUE
LTK 1stExon
cg10885338 chr2 cg10885338 NA chr2:106681982-106682403 S_Shore TRUE
C2orf40 Body
cg19186356 chr12 cg19186356 NA chr12:112204498-112204979 N_Shore
TRUE ALDH2 TSS200
cg16408970 chr10 cg16408970 NA chr10:28965704-28967266 N_Shore
TRUE BAMBI TSS1500
cg15741583 chr11 cg15741583 rs75099526 OpenSea TRUE HPX
Body
cg27462398 chr1 cg27462398 NA chr1:86174041-86174253 S_Shore TRUE
ZNHIT6TSS1500
cg05556202 chr3 cg05556202 NA OpenSea TRUE TM4SF19 5'UTR
cg10149836 chr17 cg10149836 NA chr17:29718230-29719291 S_Shore TRUE
RAB11FIP4 Body
cg00174901 chr19 cg00174901 NA chr19:709129-709990 Island TRUE
PALM Body
cg17692403 chr20 cg17692403 NA OpenSea TRUE R3HDML
TSS1500
cg16173067 chr20 cg16173067 NA chr20:1305899-1306554 S_Shelf TRUE
SDCBP2 5'UTR
cg10693071 chr5 cg10693071 NA chr5:114514716-114516220 S_Shore TRUE
TRIM36 TSS200
cg18732541 chr19 cg18732541 NA chr19:7745490-7747714 N_Shelf TRUE
C19orf59 TSS1500
cg21057494 chr3 cg21057494 rs75097554 OpenSea TRUE CLEC3B

TSS1500

cg14859417 chr10 cg14859417 NA chr10:129705217-129706237 N_Shore TRUE PTPRE TSS1500

cg23900225 chr10 cg23900225 NA chr10:35896518-35897461 N_Shelf TRUE GJD4 TSS200

cg20125091 chr1 cg20125091 NA chr1:92945907-92952609 S_Shore TRUE GFI1 TSS1500

cg18414381 chr11 cg18414381 NA OpenSea TRUE EHF 5'UTR

cg19026260 chr11 cg19026260 NA chr11:66045211-66045708 Island TRUE CNIH2 TSS1500

cg04863713 chr1 cg04863713 NA chr1:150521349-150522368 N_Shore TRUE ADAMTSL4 TSS1500

cg05421688 chr1 cg05421688 rs2245702 chr1:179711947-179713951 N_Shore TRUE FAM163A TSS1500

cg03826976 chr11 cg03826976 NA chr11:7694711-7695685 Island TRUE CYB5R2 TSS1500

cg11877382 chr12 cg11877382 NA chr12:129337870-129338653 S_Shore TRUE GLT1D1 Body

cg27016307 chr19 cg27016307 rs3745300 chr19:49660880-49661157 N_Shore TRUE HRC TSS1500

cg17982102 chr18 cg17982102 NA chr18:9707752-9709311 Island TRUE RAB31 TSS1500

cg16386080 chr9 cg16386080 NA chr9:90589209-90589807 N_Shore TRUE CDK20 Body

cg16992787 chr19 cg16992787 rs7248090 chr19:5915007-5915301 N_Shore TRUE CAPS Body

cg00448720 chr13 cg00448720 NA chr13:24882098-24882951 S_Shore TRUE C1QTNF9 TSS1500

cg19862344 chr6 cg19862344 NA OpenSea TRUE RHAG TSS1500

cg21092324 chr4 cg21092324 NA OpenSea TRUE MMRN1 1stExon

cg16862361 chr2 cg16862361 NA OpenSea TRUE XDH TSS200

cg11277126 chr20 cg11277126 NA chr20:33680438-33681029 Island TRUE

TRPC4AP TSS1500
cg13064571 chr8 cg13064571 NA OpenSea TRUE C8orf44 TSS1500
cg15312298 chr8 cg15312298 rs78852684 chr8:127568676-127570873 S_Shore TRUE FAM84B TSS1500
cg01485645 chr17 cg01485645 NA chr17:36858278-36861697 S_Shore TRUE MLLT6 Body
cg17675150 chr18 cg17675150 NA chr18:56530395-56531288 N_Shore TRUE ZNF532 TSS1500
cg08810582 chr12 cg08810582 rs7296782 chr12:69139815-69140206 N_Shore TRUE SLC35E3 TSS1500
cg18495563 chr11 cg18495563 NA chr11:33060699-33061846 S_Shore TRUE TCP11L1 5'UTR
cg18055007 chr6 cg18055007 NA chr6:31695894-31698245 Island TRUE DDAH2 TSS200
cg18437633 chr2 cg18437633 NA OpenSea TRUE ITGB6 TSS200
cg11719297 chr21 cg11719297 rs6586238 chr21:43186280-43187436 Island TRUE RIPK4 1stExon
cg07378350 chr20 cg07378350 rs117814675 OpenSea TRUE GDF5 TSS1500
cg06339657 chr5 cg06339657 NA chr5:76506029-76507189 N_Shore TRUE PDE8B TSS1500
cg27176536 chr16 cg27176536 NA chr16:2198455-2199129 N_Shore TRUE RAB26 TSS1500
cg20584011 chr5 cg20584011 NA chr5:848389-848770 S_Shelf TRUE ZDHHC11 TSS1500
cg25484904 chr4 cg25484904 NA chr4:48987790-48988808 Island TRUE CWH43 TSS1500
cg19525717 chr20 cg19525717 NA chr20:43160339-43160817 N_Shore TRUE PKIG TSS200
cg21513385 chr12 cg21513385 NA chr12:51663404-51664411 Island TRUE SMAGP 5'UTR
cg04456238 chr11 cg04456238 NA chr11:32448261-32449744 S_Shore TRUE WT1 Body

cg06310844 chr19 cg06310844 NA chr19:33071875-33072808 Island TRUE
PDCD5 Body
cg15127733 chr20 cg15127733 NA chr20:3713159-3713515 S_Shore TRUE
HSPA12B 5'UTR
cg19255783 chr4 cg19255783 rs17006484 OpenSea TRUE PLAC8
TSS200
cg22461018 chr4 cg22461018 rs72696236 OpenSea TRUE MORF4
TSS1500
cg07965823 chr14 cg07965823 NA chr14:77964571-77965670 Island TRUE
ISM2 Body
cg01993576 chr6 cg01993576 NA chr6:44187186-44187400 S_Shore TRUE
SLC29A1 5'UTR
cg20225915 chr11 cg20225915 NA chr11:804827-805608 S_Shore TRUE
LRDD TSS1500
cg17704839 chr19 cg17704839 NA chr19:9938325-9938815 S_Shore TRUE
UBL5 Body
cg16853982 chr1 cg16853982 NA chr1:236849472-236850323 N_Shore
TRUE ACTN2 TSS1500
cg05778847 chr19 cg05778847 rs4803934 chr19:38746638-38747379
N_Shore TRUE PPP1R14A Body
cg00311768 chr8 cg00311768 rs191134608 chr8:144699233-144700481
N_Shore TRUE TSTA3 Body
cg25259754 chr1 cg25259754 NA OpenSea TRUE FCRL3 Body
cg19514469 chr16 cg19514469 NA chr16:67233032-67233862 Island TRUE
ELMO3 Body
cg19224278 chr15 cg19224278 NA chr15:101419261-101421133 N_Shore
TRUE ALDH1A3 TSS1500
cg22421699 chr1 cg22421699 NA OpenSea TRUE SMPDL3B
TSS200
cg09298623 chr3 cg09298623 NA chr3:123710680-123711108 N_Shore
TRUE ROPN1 TSS1500
cg22396755 chr1 cg22396755 NA chr1:21995063-21995914 Island TRUE
RAP1GAP 5'UTR

cg04925864 chr16 cg04925864 NA chr16:67562575-67563390 N_Shore TRUE FAM65A TSS1500
cg11536940 chr8 cg11536940 NA chr8:97657174-97657943 S_Shore TRUE PGCP 5'UTR
cg08575537 chr7 cg08575537 NA chr7:100318106-100318684 Island TRUE EPO Body
cg09191232 chr4 cg09191232 NA chr4:108640845-108641835 S_Shore TRUE PAPSS1 TSS1500
cg19418958 chr8 cg19418958 NA OpenSea TRUE TEX15 1stExon
cg11154879 chr20 cg11154879 NA OpenSea TRUE C20orf151 5'UTR
cg13631259 chr11 cg13631259 NA chr11:67396844-67398294 Island TRUE NUDT8 Body
cg27634151 chr11 cg27634151 NA chr11:94134226-94134848 Island TRUE GPR83 TSS200
cg25027167 chr8 cg25027167 NA chr8:145549984-145551217 Island TRUE DGAT1 TSS1500
cg00850538 chr2 cg00850538 NA chr2:36582397-36584260 N_Shore TRUE CRIM1 TSS1500
cg22780475 chr19 cg22780475 NA chr19:45281133-45281355 S_Shore TRUE CBLC 1stExon
cg22377998 chr22 cg22377998 NA chr22:20003521-20004695 S_Shore TRUE ARVCF TSS1500
cg20019546 chr7 cg20019546 NA chr7:37955622-37956555 Island TRUE SFRP4 1stExon
cg22740783 chr2 cg22740783 NA chr2:27341567-27341923 N_Shore TRUE CGREF1 5'UTR
cg24800810 chr1 cg24800810 NA chr1:241520103-241520790 S_Shore TRUE RGS7 TSS1500
cg04001333 chr14 cg04001333 rs2287015 chr14:76044590-76045102 S_Shore TRUE FLVCR2 1stExon
cg05222924 chr11 cg05222924 NA chr11:32452144-32452708 N_Shore TRUE WT1 Body
cg07408456 chr19 cg07408456 NA OpenSea TRUE PGLYRP2

TSS1500

cg00208967 chr19 cg00208967 NA chr19:10046824-10047067 S_Shore TRUE OLFM2 TSS1500

cg07548313 chr1 cg07548313 NA OpenSea TRUE CD1C Body

cg21073927 chr8 cg21073927 NA chr8:11559596-11562956 Island TRUE GATA4 5'UTR

cg15783027 chr8 cg15783027 NA chr8:145669110-145670460 Island TRUE NFKBIL2 Body

cg18705301 chr15 cg18705301 NA chr15:41694493-41694756 S_Shore TRUE NDUFAF1 TSS1500

cg18997129 chr7 cg18997129 NA OpenSea TRUE EPHA1 1stExon

cg09328024 chr1 cg09328024 NA chr1:206808523-206809187 N_Shore TRUE DYRK3 TSS1500

cg03389133 chr22 cg03389133 NA OpenSea TRUE SSTR3 TSS1500

cg25974617 chr8 cg25974617 rs73685686 chr8:71519680-71520857 S_Shore TRUE TRAM1 TSS1500

cg26116551 chr19 cg26116551 NA chr19:30865683-30866490 N_Shelf TRUE ZNF536 TSS200

cg00090147 chr8 cg00090147 NA chr8:11559596-11562956 Island TRUE GATA4 5'UTR

cg06734812 chr19 cg06734812 NA chr19:3224743-3225007 N_Shore TRUE BRUNOL5 TSS1500

cg02789485 chr12 cg02789485 NA chr12:75784799-75785241 N_Shore TRUE GLIPR1L2 TSS1500

cg05292376 chr17 cg05292376 NA chr17:79093076-79096677 Island TRUE AATK Body

cg03991512 chr16 cg03991512 NA chr16:75148398-75148864 S_Shore TRUE LDHD Body

cg05538432 chr12 cg05538432 NA OpenSea TRUE C1S TSS1500

cg22909609 chr13 cg22909609 NA chr13:102106165-102106488 N_Shore TRUE ITGBL1 Body

cg07123069 chr12 cg07123069 NA chr12:54366815-54369103 Island TRUE HOXC11 1stExon

cg22215728 chr3 cg22215728 NA chr3:61236606-61237227 Island TRUE FHIT 5'UTR
cg21624282 chr13 cg21624282 rs80048274 OpenSea TRUE C13orf28 1stExon
cg15652212 chr7 cg15652212 rs7806162 chr7:142985047-142985810 N_Shelf TRUE TMEM139 TSS1500
cg06690548 chr4 cg06690548 NA OpenSea TRUE SLC7A11 Body
cg11438428 chr10 cg11438428 NA chr10:23480697-23482455 Island TRUE PTF1A 1stExon
cg24364574 chr20 cg24364574 NA chr20:47443734-47445181 N_Shore TRUE PREX1 Body
cg12781568 chr11 cg12781568 NA chr11:32452144-32452708 N_Shore TRUE WT1 Body
cg24977027 chr2 cg24977027 NA chr2:88469691-88470386 N_Shore TRUE THNSL2 TSS1500
cg06595693 chr15 cg06595693 NA chr15:40650055-40651102 N_Shore TRUE DISP2 TSS1500
cg24505341 chr3 cg24505341 NA chr3:62859617-62861190 S_Shore TRUE CADPS TSS1500
cg20973210 chr19 cg20973210 rs1476563 chr19:2278605-2278848 S_Shelf TRUE C19orf35 TSS1500
cg22262140 chr22 cg22262140 NA chr22:21982786-21984484 S_Shelf TRUE CCDC116 TSS200
cg03625911 chr1 cg03625911 rs7515776 OpenSea TRUE CHI3L1 1stExon
cg26525091 chr19 cg26525091 NA chr19:496158-496481 Island TRUE MADCAM1 TSS1500
cg07327468 chr6 cg07327468 NA chr6:133562086-133563586 N_Shore TRUE EYA4 TSS1500
cg27167601 chr15 cg27167601 NA chr15:61519621-61520031 S_Shore TRUE RORA TSS1500
cg01656853 chr19 cg01656853 NA chr19:49199964-49200184 N_Shore TRUE FUT2 TSS200

cg16377872 chr19 cg16377872 NA OpenSea TRUE SLC1A6 TSS1500
cg09997082 chr19 cg09997082 rs73573246 chr19:46174372-46174575 N_Shelf TRUE GIPR TSS1500
cg16028753 chr1 cg16028753 NA chr1:53527572-53528974 N_Shore TRUE PODN TSS1500
cg25782229 chr11 cg25782229 NA chr11:32452144-32452708 N_Shore TRUE WT1 Body
cg16983159 chr5 cg16983159 NA OpenSea TRUE TMEM173 TSS200
cg04582938 chr9 cg04582938 NA chr9:139971499-139973965 N_Shore TRUE UAP1L1 TSS1500
cg23873703 chr3 cg23873703 rs9812862 OpenSea TRUE KCNAB1 1stExon
cg11378484 chr3 cg11378484 NA chr3:63849046-63850357 N_Shore TRUE THOC7 Body
cg02876062 chr10 cg02876062 NA OpenSea TRUE FAM107B 1stExon
cg14851685 chr19 cg14851685 NA chr19:15619223-15619613 N_Shore TRUE CYP4F22 TSS1500
cg11004890 chr20 cg11004890 NA chr20:3218578-3220930 N_Shore TRUE SLC4A11 TSS200
cg22774472 chr2 cg22774472 NA OpenSea TRUE COL5A2 TSS200
cg11566244 chr11 cg11566244 rs8191446 chr11:67350928-67351953 Island TRUE GSTP1 Body
cg01484156 chr8 cg01484156 NA OpenSea TRUE NCALD 5'UTR
cg03642518 chr8 cg03642518 NA OpenSea TRUE PNOC 5'UTR
cg11368643 chr5 cg11368643 NA chr5:140626444-140627373 N_Shore TRUE PCDHB15 TSS200
cg10878307 chr17 cg10878307 NA OpenSea TRUE PRR15L 5'UTR
cg21105318 chr2 cg21105318 NA OpenSea TRUE ITGB6 1stExon
cg22892904 chr17 cg22892904 NA chr17:77751378-77751673 N_Shore

TRUE CBX2 TSS1500
cg09107315 chr1 cg09107315 NA chr1:78511577-78512161 N_Shore TRUE GIPC2 TSS200
cg22748452 chr20 cg22748452 rs12481467 chr20:633541-634330 S_Shore TRUE SRXN1 TSS1500
cg25341653 chr16 cg25341653 NA chr16:67233032-67233862 Island TRUE ELMO3 1stExon
cg17836145 chr6 cg17836145 NA OpenSea TRUE VNN2 TSS200
cg03914397 chr5 cg03914397 NA OpenSea TRUE NMUR2 1stExon
cg09173897 chr14 cg09173897 NA chr14:94640366-94641648 N_Shore TRUE PPP4R4 TSS1500
cg10670077 chr19 cg10670077 NA chr19:7293340-7294450 Island TRUE INSR Body
cg26385743 chr2 cg26385743 NA chr2:131099469-131100863 S_Shore TRUE IMP4 Body
cg11809091 chr5 cg11809091 NA chr5:140553681-140554637 N_Shore TRUE PCDHB7 TSS200
cg03973663 chr8 cg03973663 NA chr8:56792115-56793325 N_Shore TRUE LYN TSS1500
cg12085660 chr19 cg12085660 NA chr19:44123485-44124244 Island TRUE ZNF428 TSS200
cg00498305 chr10 cg00498305 NA chr10:119000435-119001530 N_Shore TRUE SLC18A2 TSS1500
cg01835489 chr12 cg01835489 NA chr12:53297442-53297824 S_Shore TRUE KRT8 TSS1500
cg18133957 chr19 cg18133957 rs80271587 chr19:1450056-1450387 S_Shore TRUE APC2 5'UTR
cg11136562 chr5 cg11136562 NA OpenSea TRUE ARAP3 5'UTR
cg19601328 chr17 cg19601328 NA OpenSea TRUE MAP3K14 5'UTR
cg05924583 chr1 cg05924583 NA chr1:3566445-3569636 Island TRUE TP73 5'UTR
cg04586023 chr22 cg04586023 NA chr22:50689160-50690167 S_Shore TRUE

HDAC10 TSS1500
cg15916628 chr19 cg15916628 rs1079082 chr19:56089679-56090859
S_Shore TRUE ZNF579 TSS1500
cg17127823 chr8 cg17127823 rs73685686 chr8:71519680-71520857 S_Shore
TRUE TRAM1 TSS1500
cg10044101 chr6 cg10044101 NA OpenSea TRUE VNN2 5'UTR
cg17786776 chr7 cg17786776 NA chr7:32996732-32997591 N_Shore TRUE
FKBP9 TSS1500
cg11484576 chr11 cg11484576 NA OpenSea TRUE LGALS12
TSS1500
cg18771300 chr14 cg18771300 NA OpenSea TRUE RHOJ 1stExon
cg26929536 chr15 cg26929536 rs3809498 chr15:51973533-51973838
N_Shore TRUE SCG3 TSS200
cg20330472 chr6 cg20330472 NA chr6:133562086-133563586 N_Shore
TRUE EYA4 TSS1500
cg00893242 chr7 cg00893242 NA chr7:127291647-127292551 N_Shore
TRUE SND1 TSS1500
cg17803965 chr1 cg17803965 NA OpenSea TRUE SPOCD1
TSS1500
cg23539753 chr2 cg23539753 NA OpenSea TRUE SP100 TSS200
cg25763788 chr6 cg25763788 rs6298 chr6:78172231-78174088 Island TRUE
HTR1B 1stExon
cg01119135 chr1 cg01119135 rs2629678 OpenSea TRUE C1orf116
5'UTR
cg15776355 chr12 cg15776355 NA OpenSea TRUE C1R TSS1500
cg10305797 chr19 cg10305797 NA OpenSea TRUE KRTDAP
TSS1500
cg05647859 chr12 cg05647859 NA chr12:81330608-81331514 S_Shore TRUE
LIN7A TSS200
cg04431054 chr5 cg04431054 NA chr5:126853265-126853925 N_Shore
TRUE PRRC1 TSS1500
cg00756887 chr1 cg00756887 NA OpenSea TRUE PVRL4 TSS1500
cg24273512 chr6 cg24273512 NA chr6:105627406-105627830 S_Shore TRUE

POPDC3 TSS1500

cg23950724 chr12 cg23950724 rs6488162 chr12:33591763-33593050 S_Shore TRUE SYT10 TSS1500

cg10757144 chr5 cg10757144 rs35154204 chr5:140626444-140627373 N_Shore TRUE PCDHB15 1stExon

cg17279839 chr7 cg17279839 NA chr7:150037459-150039031 Island TRUE RARRES2 5'UTR

cg19853703 chr22 cg19853703 NA OpenSea TRUE APOL6 5'UTR

cg11300809 chr2 cg11300809 NA chr2:223288922-223290013 N_Shore TRUE SGPP2 TSS1500

cg19917856 chr19 cg19917856 NA chr19:39687597-39687964 Island TRUE NCCRP1 Body

cg22721827 chr16 cg22721827 NA chr16:2521086-2525929 N_Shore TRUE NTN3 TSS1500

cg19778698 chr7 cg19778698 rs10254597 chr7:1570406-1571136 N_Shore TRUE MAFK TSS1500

cg13530039 chr11 cg13530039 NA chr11:62690974-62691488 N_Shore TRUE CHRM1 TSS1500

cg19896198 chr1 cg19896198 NA chr1:12123488-12124148 N_Shore TRUE TNFRSF8 TSS200

cg24664957 chr12 cg24664957 NA chr12:123380333-123380894 S_Shore TRUE VPS37B TSS1500

cg21301148 chr14 cg21301148 NA OpenSea TRUE MYH6 TSS1500

cg13470920 chr19 cg13470920 rs45448796 OpenSea TRUE VAV1 1stExon

cg15798455 chr3 cg15798455 NA chr3:71834068-71834653 N_Shore TRUE PROK2 Body

cg02983451 chr2 cg02983451 NA chr2:10182635-10184702 Island TRUE KLF11 TSS1500

cg15443822 chr17 cg15443822 NA chr17:3867059-3868095 N_Shore TRUE ATP2A3 Body

cg10398682 chr15 cg10398682 NA chr15:83951980-83953930 Island TRUE BNC1 Body

cg08924430 chr4 cg08924430 NA chr4:106067473-106067968 N_Shore TRUE TET2 TSS1500
cg20368904 chr14 cg20368904 NA chr14:103593234-103593923 N_Shore TRUE TNFAIP2 Body
cg01426743 chr1 cg01426743 NA chr1:178994804-178995917 N_Shore TRUE FAM20B TSS1500
cg21475402 chr1 cg21475402 rs113194954chr1:156611639-156612447 Island TRUE BCAN 5'UTR
cg04716261 chr1 cg04716261 NA chr1:2938633-2939043 N_Shore TRUE ACTRT2 TSS200
cg19946699 chr21 cg19946699 NA chr21:40817302-40817973 Island TRUE SH3BGR TSS200
cg11473104 chr13 cg11473104 NA chr13:48611646-48612268 N_Shore TRUE NUDT15 TSS200
cg07546360 chr22 cg07546360 NA chr22:46481815-46482024 Island TRUE LOC400931 Body
cg11618577 chr2 cg11618577 NA chr2:27665251-27665670 Island TRUE KRTCAP3 Body
cg12188860 chr8 cg12188860 rs74445975 chr8:144416712-144417054 N_Shore TRUE TOP1MT Body
cg14679202 chr22 cg14679202 NA chr22:41601069-41601476 N_Shore TRUE L3MBTL2 TSS1500
cg12285118 chr10 cg12285118 NA OpenSea TRUE FAM107B TSS200
cg24421410 chr6 cg24421410 NA OpenSea TRUE HLA-DMA Body
cg25552492 chr8 cg25552492 NA chr8:22014152-22014559 N_Shore TRUE LGI3 1stExon
cg22797169 chr2 cg22797169 NA chr2:102803672-102804556 N_Shore TRUE IL1RL2 TSS200
cg19884658 chr1 cg19884658 NA chr1:6661776-6663844 S_Shore TRUE KLHL21 TSS1500
cg06817269 chr15 cg06817269 NA OpenSea TRUE GCNT3 5'UTR
cg06539804 chr20 cg06539804 NA chr20:2780978-2781497 Island TRUE

CPXM1 TSS1500

cg19145398 chr20 cg19145398 NA chr20:30432900-30433242 S_Shore TRUE FOXS1 TSS1500

cg08634464 chr19 cg08634464 NA chr19:2900329-2901203 Island TRUE ZNF57 Body

cg10458876 chr16 cg10458876 NA chr16:67926918-67927696 Island TRUE PSKH1 5'UTR

cg03702236 chr5 cg03702236 NA chr5:9544692-9546715 S_Shore TRUE SEMA5A TSS1500

cg07388493 chr1 cg07388493 rs72940829 OpenSea TRUE NDUFS5 TSS1500

cg01870826 chr7 cg01870826 rs62444320 chr7:5111620-5112088 Island TRUE LOC389458 Body

cg22402007 chr9 cg22402007 NA chr9:87283178-87285704 N_Shore TRUE NTRK2 TSS1500

cg10694914 chr11 cg10694914 NA OpenSea TRUE POU2AF1 TSS1500

cg14759043 chr12 cg14759043 NA chr12:3862068-3862606 Island TRUE EFCAB4B TSS1500

cg01081263 chr11 cg01081263 NA chr11:9112461-9113459 Island TRUE SCUBE2 Body

cg17675882 chr20 cg17675882 NA chr20:35374110-35374654 N_Shore TRUE NDRG3 5'UTR

cg00888561 chr13 cg00888561 NA chr13:52158056-52159235 N_Shore TRUE WDFY2 TSS1500

cg07730301 chr11 cg07730301 NA OpenSea TRUE ALDH3B1 5'UTR

cg08446111 chr16 cg08446111 NA chr16:48278058-48278655 S_Shore TRUE LONP2 Body

cg10919204 chr5 cg10919204 NA chr5:31193952-31194419 N_Shore TRUE CDH6 TSS1500

cg20916523 chr3 cg20916523 NA chr3:10183305-10183941 S_Shore TRUE VHL Body

cg02237119 chr7 cg02237119 NA chr7:73256704-73256926 N_Shore TRUE

WBSCR27 Body
cg16536450 chr9 cg16536450 NA chr9:139654897-139655154 S_Shelf TRUE
LCN15 1stExon
cg06424894 chr15 cg06424894 NA chr15:45315201-45315543 N_Shore
TRUE SORD TSS1500
cg09001777 chr19 cg09001777 NA OpenSea TRUE FUT3 TSS200
cg12508624 chr11 cg12508624 NA chr11:119293320-119293943 Island TRUE
THY1 5'UTR
cg13797282 chr22 cg13797282 NA chr22:37914768-37915883 Island TRUE
CARD10 Body
cg03424436 chr20 cg03424436 rs115545043 chr20:34042854-34043585
S_Shore TRUE CEP250 5'UTR
cg04329382 chr22 cg04329382 NA chr22:46481815-46482024 N_Shore
TRUE LOC400931 TSS1500
cg06540941 chr1 cg06540941 NA chr1:171710845-171711607 N_Shore
TRUE VAMP4 5'UTR
cg03317245 chr17 cg03317245 NA chr17:3599052-3599551 S_Shore TRUE
P2RX5 TSS1500
cg06800962 chr3 cg06800962 NA OpenSea TRUE TM4SF1
TSS1500
cg09313705 chr17 cg09313705 NA chr17:46620367-46621373 S_Shore TRUE
HOXB2 TSS200
cg24855780 chr11 cg24855780 NA chr11:57091614-57092831 N_Shelf TRUE
TNKS1BP1 5'UTR
cg00273068 chr20 cg00273068 NA chr20:39994545-39995810 Island TRUE
EMILIN3 TSS1500
cg16779976 chr10 cg16779976 rs7916154 OpenSea TRUE BLNK
1stExon
cg27243140 chr17 cg27243140 NA chr17:72931729-72932601 N_Shore
TRUE OTOP3 TSS1500
cg12438037 chr9 cg12438037 NA chr9:138391394-138393271 S_Shore TRUE
MRPS2 Body
cg00687686 chr16 cg00687686 NA chr16:58497033-58498595 Island TRUE

NDRG4 TSS1500
cg05657090 chr19 cg05657090 NA chr19:12917186-12917676 N_Shore TRUE RNASEH2A TSS1500
cg27109971 chr18 cg27109971 NA chr18:267710-268382 N_Shore TRUE THOC1 Body
cg06577005 chr1 cg06577005 NA chr1:26758492-26758856 N_Shore TRUE DHDDS TSS1500
cg00995520 chr1 cg00995520 NA chr1:111216244-111217937 S_Shore TRUE KCNA3 TSS1500
cg15228639 chr15 cg15228639 NA OpenSea TRUE PLA2G4E Body
cg12220493 chr14 cg12220493 NA chr14:36986362-36990576 Island TRUE NKX2-1 TSS1500
cg02537838 chr20 cg02537838 NA OpenSea TRUE C20orf151 TSS200
cg24476569 chr13 cg24476569 NA chr13:95953337-95954211 S_Shore TRUE ABCC4 TSS1500
cg20857455 chr12 cg20857455 NA chr12:39300469-39300680 N_Shore TRUE CPNE8 TSS1500
cg17891123 chr15 cg17891123 NA chr15:90931162-90932019 N_Shore TRUE IQGAP1 TSS1500
cg05590982 chr16 cg05590982 NA OpenSea TRUE NUPR1 1stExon
cg18938204 chr20 cg18938204 NA chr20:39994545-39995810 Island TRUE EMILIN3 Body
cg22620680 chr3 cg22620680 NA chr3:122920703-122921114 N_Shore TRUE SEC22A TSS1500
cg11874272 chr3 cg11874272 NA OpenSea TRUE CD86 TSS1500
cg13210534 chr11 cg13210534 NA OpenSea TRUE HSPB2 Body
cg25691167 chr7 cg25691167 NA chr7:19184818-19185033 Island TRUE FERD3L 1stExon
cg08831744 chr7 cg08831744 NA chr7:45960137-45961347 Island TRUE IGFBP3 TSS1500
cg00430287 chr6 cg00430287 NA OpenSea TRUE FRK TSS1500
cg17877656 chr10 cg17877656 NA chr10:105110265-105111008 N_Shore

TRUE PCGF6 Body

cg23092823 chr1 cg23092823 NA chr1:53527572-53528974 Island TRUE PODN Body

cg05266781 chr16 cg05266781 rs139631144 chr16:54962422-54967805 Island TRUE IRX5 Body

cg14785479 chr22 cg14785479 NA chr22:20790638-20792665 Island TRUE SCARF2 TSS1500

cg15403517 chr2 cg15403517 NA chr2:31805293-31806403 Island TRUE SRD5A2 TSS1500

cg23499956 chr1 cg23499956 NA OpenSea TRUE S100A16 5'UTR

cg13749822 chr4 cg13749822 NA chr4:145566242-145567413 Island TRUE HHIP TSS1500

cg10705800 chr1 cg10705800 NA chr1:41326949-41328285 S_Shore TRUE CITED4 TSS1500

cg01427567 chr19 cg01427567 rs641738 OpenSea TRUE TMC4 1stExon

cg16361890 chr5 cg16361890 rs149149685 chr5:179222608-179223825 N_Shelf TRUE LTC4S TSS1500

cg02330106 chr10 cg02330106 NA chr10:131264948-131265710 N_Shore TRUE MGMT TSS1500

cg21435336 chr19 cg21435336 NA chr19:17392383-17393775 Island TRUE ANKLE1 Body

cg26862286 chr2 cg26862286 NA chr2:232328445-232330149 S_Shore TRUE NCL TSS1500

cg03543593 chr6 cg03543593 NA OpenSea TRUE TNXB 5'UTR

cg10667970 chr1 cg10667970 NA chr1:11724113-11724885 N_Shore TRUE FBXO6 TSS200

cg18342900 chr16 cg18342900 NA chr16:46864453-46865561 Island TRUE C16orf87 1stExon

cg08314660 chr11 cg08314660 NA chr11:394257-394619 N_Shore TRUE PKP3 TSS200

cg09837648 chr3 cg09837648 NA chr3:48470230-48471389 N_Shelf TRUE PLXNB1 5'UTR

cg26980692 chr11 cg26980692 NA chr11:60718428-60718888 S_Shore TRUE SLC15A3 TSS1500
cg12875426 chr18 cg12875426 NA chr18:46986504-46987194 S_Shore TRUE DYM TSS1500
cg26565975 chr21 cg26565975 NA OpenSea TRUE KRTAP20-1 TSS200
cg25949363 chr17 cg25949363 NA chr17:48350422-48350832 S_Shore TRUE TMEM92 Body
cg09937039 chr14 cg09937039 rs149221817 OpenSea TRUE BATF Body
cg15164103 chr7 cg15164103 NA chr7:130126017-130126801 S_Shore TRUE MEST 5'UTR
cg14211646 chr1 cg14211646 NA chr1:26758492-26758856 S_Shore TRUE DHDDS 5'UTR
cg21052164 chr1 cg21052164 rs71651696 chr1:154540233-154540573 N_Shore TRUE CHRNB2 TSS1500
cg12105450 chr2 cg12105450 NA OpenSea TRUE CASP10 5'UTR
cg00910067 chr19 cg00910067 NA chr19:33717512-33717930 Island TRUE SLC7A10 TSS1500
cg11554507 chr7 cg11554507 NA OpenSea TRUE NEUROD6 TSS200
cg11783497 chr2 cg11783497 NA OpenSea TRUE IL1RN TSS200
cg12936747 chr10 cg12936747 NA chr10:95360389-95361387 Island TRUE RBP4 TSS1500
cg16426459 chr2 cg16426459 NA chr2:238395061-238396241 Island TRUE MLPH 5'UTR
cg06636463 chr2 cg06636463 NA chr2:15731792-15732494 N_Shore TRUE DDX1 TSS200
cg12914657 chr7 cg12914657 NA chr7:150417414-150418018 N_Shelf TRUE GIMAP1 TSS200
cg20798152 chr5 cg20798152 rs17358216 chr5:71014917-71015715 N_Shore TRUE CARTPT TSS200
cg04576021 chr6 cg04576021 NA OpenSea TRUE HLA-DOB Body

cg11832722 chr18 cg11832722 NA chr18:28621489-28623117 Island TRUE DSC3 TSS1500
cg25432696 chr4 cg25432696 NA chr4:74702420-74702627 S_Shore TRUE CXCL6 Body
cg09906488 chr10 cg09906488 NA chr10:12390802-12392589 Island TRUE CAMK1D TSS1500
cg09630437 chr19 cg09630437 NA chr19:2739851-2740393 Island TRUE SLC39A3 TSS1500
cg09214254 chr6 cg09214254 NA chr6:168841438-168841699 Island TRUE SMOC2 TSS1500
cg25140571 chr3 cg25140571 rs73132859 chr3:8808961-8811280 S_Shore TRUE OXTR TSS200
cg26911787 chr12 cg26911787 NA chr12:96587839-96588090 N_Shore TRUE ELK3 TSS1500
cg08555612 chr3 cg08555612 NA chr3:71834068-71834653 Island TRUE PROK2 TSS1500
cg21296676 chr6 cg21296676 NA chr6:133562086-133563586 N_Shore TRUE EYA4 TSS1500
cg27154163 chr4 cg27154163 NA chr4:55523409-55525297 Island TRUE KIT TSS1500
cg24715245 chr4 cg24715245 rs73809701 chr4:41258759-41259867 Island TRUE UCHL1 TSS200
cg22362636 chr4 cg22362636 NA chr4:2757653-2758010 S_Shore TRUE TNIP2 TSS1500
cg20970875 chr19 cg20970875 NA chr19:8407674-8408338 Island TRUE KANK3 TSS200
cg22190705 chr2 cg22190705 NA chr2:9346383-9347944 N_Shore TRUE ASAP2 TSS1500
cg16425577 chr8 cg16425577 NA chr8:142138208-142139342 S_Shore TRUE DENND3 5'UTR
cg27496506 chr15 cg27496506 NA OpenSea TRUE TGM5 TSS200
cg03064067 chr12 cg03064067 NA chr12:85305200-85305422 S_Shore TRUE SLC6A15 TSS1500

cg00638514 chr17 cg00638514 NA chr17:72931729-72932601 Island TRUE OTOP3 Body
cg08450982 chr19 cg08450982 NA chr19:41196279-41197107 N_Shore TRUE NUMBL Body
cg11976790 chr10 cg11976790 NA OpenSea TRUE DMBT1 TSS200
cg03148461 chr7 cg03148461 rs7810757 chr7:140623997-140624949 S_Shore TRUE BRAF TSS1500
cg20050113 chr2 cg20050113 NA chr2:103235376-103236554 S_Shore TRUE SLC9A2 Body
cg08749917 chr3 cg08749917 NA chr3:186917411-186917756 N_Shelf TRUE RTP1 1stExon
cg00673191 chr21 cg00673191 NA OpenSea TRUE DOPEY2 5'UTR
cg22054191 chr19 cg22054191 NA chr19:42637277-42637684 Island TRUE POU2F2 TSS1500
cg10710439 chr19 cg10710439 NA chr19:38182793-38183327 S_Shore TRUE ZNF781 TSS1500
cg07766612 chr1 cg07766612 NA chr1:26372281-26373313 Island TRUE SLC30A2 TSS1500
cg03561565 chr8 cg03561565 NA chr8:99438692-99440425 N_Shore TRUE KCNS2 TSS1500
cg16864658 chr3 cg16864658 NA chr3:42306149-42307520 Island TRUE CCK 5'UTR
cg10092957 chr19 cg10092957 rs867348 chr19:39687597-39687964 N_Shore TRUE NCCRP1 TSS1500
cg01772980 chr11 cg01772980 NA OpenSea TRUE SCGB1D1 TSS200
cg13765621 chr1 cg13765621 NA chr1:158150620-158151503 N_Shore TRUE CD1D TSS1500
cg08137040 chr7 cg08137040 NA chr7:127031854-127033084 S_Shore TRUE ZNF800 TSS1500
cg11105610 chr17 cg11105610 rs142528600 OpenSea TRUE LGALS3BP TSS1500
cg24122922 chr20 cg24122922 NA chr20:24449844-24452037 N_Shore

TRUE TMEM90B TSS1500
cg00386408 chr5 cg00386408 NA chr5:135364507-135365036 Island TRUE TGFBI Body
cg23067535 chr8 cg23067535 NA OpenSea TRUE FAM83A 1stExon
cg10273210 chr3 cg10273210 NA chr3:160167184-160168200 Island TRUE TRIM59 TSS200
cg17729667 chr20 cg17729667 NA chr20:25565437-25566547 Island TRUE NINL TSS1500
cg06650786 chr19 cg06650786 NA chr19:15310519-15312027 S_Shore TRUE NOTCH3 TSS1500
cg09196959 chr6 cg09196959 rs74419673 OpenSea TRUE TRIM40 TSS200
cg04597449 chr5 cg04597449 rs2194051 chr5:94955630-94957244 N_Shore TRUE GPR150 TSS1500
cg20291049 chr2 cg20291049 NA chr2:105468851-105473488 Island TRUE POU3F3 1stExon
cg10780112 chr6 cg10780112 NA chr6:138724617-138726215 Island TRUE HEBP2 TSS200
cg07215749 chr12 cg07215749 NA chr12:93963963-93967395 Island TRUE SOCS2 5'UTR
cg25577842 chr11 cg25577842 NA chr11:32459422-32459878 Island TRUE WIT1 Body
cg26607785 chr17 cg26607785 NA chr17:45810589-45811388 N_Shore TRUE TBX21 TSS200
cg26489108 chr9 cg26489108 rs3812518 chr9:976162-977828 N_Shore TRUE DMRT3 TSS1500
cg09522147 chr12 cg09522147 NA chr12:52626793-52627577 Island TRUE KRT7 1stExon
cg18750960 chr2 cg18750960 NA chr2:177016416-177016632 Island TRUE HOXD4 1stExon
cg00711916 chr2 cg00711916 NA chr2:97426335-97426554 N_Shore TRUE CNNM4 TSS1500

cg09432376 chr22 cg09432376 NA OpenSea TRUE APOL6 TSS200
cg15784332 chr19 cg15784332 NA chr19:18899037-18902284 Island TRUE COMP TSS200
cg25554036 chr4 cg25554036 rs13127445 chr4:6271280-6272182 N_Shore TRUE WFS1 TSS1500
cg26293512 chr16 cg26293512 NA OpenSea TRUE TEPP 1stExon
cg21197871 chr8 cg21197871 NA chr8:96037018-96037600 N_Shore TRUE C8orf38 TSS1500
cg11394785 chr5 cg11394785 NA chr5:179222608-179223825 N_Shore TRUE LTC4S 1stExon
cg25664034 chr7 cg25664034 NA chr7:128470443-128471161 N_Shore TRUE FLNC TSS1500
cg10016608 chr17 cg10016608 NA OpenSea TRUE GAS2L2 1stExon
cg15147516 chr1 cg15147516 NA chr1:226411007-226411880 Island TRUE MIXL1 Body
cg04052038 chr21 cg04052038 NA OpenSea TRUE CLDN8 1stExon
cg24331162 chr11 cg24331162 NA OpenSea TRUE SYT8 TSS200
cg06946880 chr2 cg06946880 rs17853498 OpenSea TRUE ATP6V1B1 1stExon
cg26530341 chr8 cg26530341 NA chr8:23081956-23082975 S_Shore TRUE TNFRSF10A TSS1500
cg24981018 chr8 cg24981018 NA chr8:143694430-143696166 Island TRUE ARC 1stExon
cg17483510 chr3 cg17483510 NA chr3:179168735-179169593 N_Shore TRUE GNB4 5'UTR
cg01657207 chr3 cg01657207 NA chr3:44690126-44690587 N_Shore TRUE ZNF35 TSS1500
cg26096837 chr11 cg26096837 rs1789364 chr11:69517840-69519929 S_Shore TRUE FGF19 TSS1500
cg13614181 chr13 cg13614181 NA chr13:42030913-42032667 Island TRUE C13orf15 TSS1500
cg05889321 chr10 cg05889321 NA chr10:97415860-97416726 S_Shore TRUE

ALDH18A1 TSS1500
cg14963371 chr1 cg14963371 NA chr1:27718892-27719349 S_Shore TRUE
GPR3 5'UTR
cg22189019 chr19 cg22189019 NA chr19:639565-640370 Island TRUE
FGF22 Body
cg05065690 chr12 cg05065690 rs2446977 chr12:49245656-49246173 Island
TRUE DDX23 TSS200
cg18746357 chr11 cg18746357 NA chr11:65430142-65431079 N_Shore
TRUE RELA Body
cg24924779 chr20 cg24924779 NA chr20:49639071-49640212 Island TRUE
KCNG1 TSS1500
cg03630088 chr10 cg03630088 NA chr10:125650820-125651373 S_Shore TRUE
CPXM2 TSS200
cg17861230 chr19 cg17861230 NA chr19:18343496-18344196 Island TRUE
PDE4C Body
cg01612158 chr1 cg01612158 rs1361889 OpenSea TRUE FCRL4
1stExon
cg02506426 chr11 cg02506426 rs7115612 chr11:77300360-77301391 Island
TRUE AQP11 TSS200
cg11814446 chr12 cg11814446 NA chr12:65563195-65564396 N_Shore
TRUE LEMD3 TSS1500
cg08017606 chr5 cg08017606 NA chr5:145316145-145316354 Island TRUE
SH3RF2 5'UTR
cg15321211 chr10 cg15321211 NA chr10:43902580-43904792 S_Shore TRUE
HNRNPF TSS1500
cg12144803 chr3 cg12144803 NA OpenSea TRUE KALRN TSS200
cg07758904 chr9 cg07758904 NA chr9:115652446-115653448 Island TRUE
SLC46A2 1stExon
cg13968061 chr1 cg13968061 NA OpenSea TRUE APCS TSS1500
cg26482939 chr19 cg26482939 rs411210 OpenSea TRUE GNA15
Body
cg12970724 chr19 cg12970724 NA chr19:18716229-18718743 Island TRUE
CRLF1 TSS1500

cg12195135 chr19 cg12195135 NA chr19:58919548-58920529 N_Shore TRUE ZNF584 TSS1500
cg09340639 chr1 cg09340639 NA OpenSea TRUE FCRL1 Body
cg18959422 chr1 cg18959422 NA OpenSea TRUE MYBPH TSS1500
cg18442986 chr2 cg18442986 NA chr2:113239249-113240584 Island TRUE TTL 1stExon
cg13121699 chr2 cg13121699 NA chr2:185463208-185463589 N_Shore TRUE ZNF804A TSS1500
cg04682845 chr3 cg04682845 NA chr3:44902959-44903623 S_Shore TRUE TMEM42 Body
cg14345882 chr6 cg14345882 NA OpenSea TRUE BTN3A2 TSS1500
cg23663332 chr8 cg23663332 NA chr8:33342171-33343141 N_Shore TRUE MAK16 TSS1500
cg19205533 chr12 cg19205533 NA chr12:15373986-15374514 Island TRUE RERG 5'UTR
cg06554928 chr17 cg06554928 NA chr17:4850942-4853907 Island TRUE PFN1 TSS1500
cg27091787 chr3 cg27091787 NA chr3:50358378-50359606 S_Shore TRUE HYAL2 TSS1500
cg10869069 chr6 cg10869069 NA chr6:101328747-101329451 S_Shore TRUE ASCC3 TSS1500
cg25682936 chr1 cg25682936 NA chr1:38455232-38455769 Island TRUE SF3A3 TSS200
cg08876932 chr11 cg08876932 NA chr11:71954816-71955659 Island TRUE PHOX2A TSS1500
cg18192417 chr10 cg18192417 NA chr10:21462128-21463808 Island TRUE NEBL TSS1500
cg10692870 chr2 cg10692870 NA chr2:216300267-216300997 Island TRUE FN1 Body
cg18790143 chr2 cg18790143 rs7577069 OpenSea TRUE OTOS TSS1500

cg17264618 chr3 cg17264618 NA chr3:40428651-40429015 Island TRUE ENTPD3 5'UTR
cg05786809 chr14 cg05786809 NA chr14:103987063-103989796 Island TRUE CKB TSS1500
cg03705396 chr3 cg03705396 NA chr3:183542496-183543804 Island TRUE MAP6D1 TSS1500
cg24516901 chr2 cg24516901 rs116629876 OpenSea TRUE FAM124B TSS200
cg22705929 chr11 cg22705929 NA chr11:57414400-57414664 S_Shelf TRUE YPEL4 TSS1500
cg11154542 chr9 cg11154542 NA chr9:90340715-90341542 N_Shore TRUE CTSL1 TSS1500
cg00282347 chr1 cg00282347 NA chr1:6241031-6241251 Island TRUE CHD5 TSS1500
cg10917602 chr16 cg10917602 NA OpenSea TRUE HSD3B7 5'UTR
cg14614211 chr10 cg14614211 NA chr10:28030182-28035211 Island TRUE MKX 5'UTR
cg17200465 chr3 cg17200465 NA chr3:40428651-40429015 N_Shore TRUE ENTPD3 TSS200
cg15736165 chr15 cg15736165 NA chr15:83951980-83953930 N_Shore TRUE BNC1 Body
cg25014318 chr6 cg25014318 NA chr6:39016307-39016998 Island TRUE GLP1R Body
cg20910746 chr11 cg20910746 NA OpenSea TRUE BBOX1 TSS200
cg01857260 chr19 cg01857260 NA chr19:50666147-50666514 S_Shore TRUE C19orf41 TSS200
cg18216249 chr12 cg18216249 NA chr12:48099317-48099905 S_Shore TRUE RPAP3 TSS1500
cg09640202 chr2 cg09640202 NA chr2:131099469-131100863 S_Shore TRUE IMP4 Body
cg25149155 chr14 cg25149155 NA chr14:23770512-23771902 S_Shelf TRUE BCL2L2 TSS1500
cg10080004 chr19 cg10080004 NA chr19:55690182-55691623 S_Shore TRUE

SYT5 TSS200
cg17199658 chr8 cg17199658 rs76477480 chr8:101661671-101662022
N_Shore TRUE SNX31 Body
cg13044277 chr15 cg13044277 NA OpenSea TRUE NRG4 5'UTR
cg15448245 chr22 cg15448245 NA OpenSea TRUE GGT5 TSS1500
cg21132577 chr8 cg21132577 rs117582683 OpenSea TRUE MYOM2
5'UTR
cg13870866 chr7 cg13870866 NA chr7:35292985-35294674 Island TRUE
TBX20 1stExon
cg14060828 chr19 cg14060828 NA chr19:49925626-49926012 S_Shore TRUE
PTH2 Body
cg16957313 chr5 cg16957313 rs3763067 chr5:172197482-172199606
S_Shore TRUE DUSP1 TSS1500
cg16752583 chr7 cg16752583 NA OpenSea TRUE TRPV6 TSS1500
cg06516124 chr11 cg06516124 NA chr11:32452144-32452708 N_Shore
TRUE WT1 Body
cg06291867 chr10 cg06291867 NA chr10:92616820-92618034 Island TRUE
HTR7 1stExon
cg26571739 chr19 cg26571739 NA OpenSea TRUE VAV1 TSS200
cg22459146 chr3 cg22459146 NA chr3:172167526-172167866 N_Shore
TRUE GHSR TSS1500
cg25608949 chr21 cg25608949 NA OpenSea TRUE TMPRSS3
TSS200
cg25604883 chr1 cg25604883 NA chr1:77747314-77748224 Island TRUE
AK5 TSS1500
cg24497819 chr12 cg24497819 NA OpenSea TRUE SELPLG
TSS200
cg03513163 chr5 cg03513163 rs2233590 OpenSea TRUE PCDHB1
1stExon
cg07337598 chr1 cg07337598 rs6672625 OpenSea TRUE ANXA9
TSS1500
cg26033681 chr15 cg26033681 NA OpenSea TRUE SNRPN 5'UTR
cg12251804 chr10 cg12251804 NA chr10:135170645-135171954 Island TRUE

C10orf125 Body
cg23338195 chr8 cg23338195 NA OpenSea TRUE SLC30A8 TSS1500
cg10052840 chr19 cg10052840 NA chr19:4558363-4558579 N_Shore TRUE SEMA6B Body
cg08912400 chr20 cg08912400 NA chr20:61992186-61993599 Island TRUE CHRNA4 TSS1500
cg05072008 chr7 cg05072008 NA chr7:50517627-50518668 Island TRUE FIGNL1 TSS1500
cg02887841 chr22 cg02887841 NA chr22:19742901-19744729 Island TRUE TBX1 5'UTR
cg17351116 chr20 cg17351116 NA chr20:25370335-25371823 Island TRUE ABHD12 TSS1500
cg19308222 chr4 cg19308222 NA OpenSea TRUE EREG TSS1500
cg17741572 chr6 cg17741572 NA OpenSea TRUE CFB Body
cg24968336 chr19 cg24968336 NA chr19:38810376-38810949 N_Shore TRUE KCNK6 TSS1500
cg21816539 chr21 cg21816539 NA chr21:31311386-31312106 S_Shore TRUE GRIK1 TSS200
cg02549424 chr22 cg02549424 NA chr22:21319178-21319912 N_Shore TRUE AIFM3 TSS1500
cg07059360 chr8 cg07059360 NA chr8:22102303-22103142 N_Shore TRUE POLR3D TSS1500
cg05535113 chr16 cg05535113 NA OpenSea TRUE CHST4 TSS1500
cg14823162 chr6 cg14823162 NA chr6:99279317-99283842 Island TRUE POU3F2 TSS1500
cg12288726 chr7 cg12288726 NA chr7:127227758-127229039 Island TRUE ARF5 TSS1500
cg07423149 chr1 cg07423149 rs57655006 OpenSea TRUE CHI3L1 TSS1500
cg06148264 chr6 cg06148264 NA chr6:2841810-2842273 N_Shore TRUE SERPINB1 5'UTR
cg11027330 chr16 cg11027330 rs62032512 chr16:764748-765994 Island

TRUE METRN TSS1500

cg18403361 chr14 cg18403361 NA chr14:38724254-38725537 S_Shore TRUE CLEC14A TSS200

cg03019000 chr3 cg03019000 NA chr3:51705062-51706022 N_Shore TRUE TEX264 TSS1500

cg03442064 chr6 cg03442064 NA chr6:41513652-41516189 Island TRUE FOXP4 5'UTR

cg01880569 chr16 cg01880569 NA chr16:82660651-82661813 N_Shore TRUE CDH13 TSS1500

cg02016419 chr17 cg02016419 NA chr17:15244705-15245126 S_Shore TRUE TEKT3 TSS1500

cg11098259 chr15 cg11098259 NA OpenSea TRUE AQP9 TSS200

cg02046532 chr20 cg02046532 rs2298148 OpenSea TRUE DEFB129 TSS200

cg22881914 chr14 cg22881914 rs10873057 chr14:52534581-52536722 Island TRUE NID2 TSS1500

cg09595479 chr12 cg09595479 NA chr12:49688873-49691360 Island TRUE PRPH 1stExon

cg11970458 chr16 cg11970458 NA chr16:31213566-31214287 N_Shore TRUE PYCARD Body

cg14944269 chr14 cg14944269 NA chr14:65438412-65439363 S_Shore TRUE RAB15 TSS1500

cg25481253 chr7 cg25481253 NA OpenSea TRUE TAS2R38 1stExon

cg16009558 chr6 cg16009558 NA chr6:101846766-101847135 N_Shore TRUE GRIK2 TSS200

cg13460409 chr21 cg13460409 NA chr21:38377937-38379437 S_Shore TRUE DSCR6 Body

cg21700166 chr14 cg21700166 NA chr14:91719576-91720228 Island TRUE GPR68 TSS200

cg26885858 chr1 cg26885858 NA chr1:21766272-21767224 Island TRUE NBPF3 TSS1500

cg04329454 chr6 cg04329454 NA chr6:33560892-33561189 Island TRUE

C6orf227 Body
cg00347729 chr11 cg00347729 NA OpenSea TRUE MMP10 TSS1500
cg19130550 chr1 cg19130550 NA chr1:161993022-161994131 S_Shore TRUE OLFML2B TSS1500
cg16449464 chr2 cg16449464 NA chr2:172864613-172865064 N_Shore TRUE MAP1D TSS1500
cg14967066 chr11 cg14967066 rs12432 chr11:315739-316539 N_Shore TRUE IFITM1 1stExon
cg16250754 chr5 cg16250754 NA OpenSea TRUE CARD6 1stExon
cg21410991 chr5 cg21410991 NA chr5:50678632-50679375 N_Shore TRUE ISL1 TSS1500
cg17207590 chr15 cg17207590 NA chr15:63569296-63570100 S_Shore TRUE APH1B Body
cg12072973 chr1 cg12072973 NA chr1:150121695-150123078 Island TRUE PLEKHO1 TSS1500
cg19005368 chr11 cg19005368 NA chr11:32851160-32851951 Island TRUE PRRG4 5'UTR
cg09119967 chr20 cg09119967 NA chr20:9495253-9495597 N_Shore TRUE C20orf103 TSS1500
cg23325242 chr22 cg23325242 rs8190371 chr22:43045009-43045679 N_Shore TRUE CYB5R3 Body
cg22406518 chr3 cg22406518 NA chr3:134092257-134093741 Island TRUE AMOTL2 TSS1500
cg10107725 chr2 cg10107725 NA chr2:160760604-160761452 S_Shore TRUE LY75 TSS1500
cg16077929 chr14 cg16077929 NA chr14:50863088-50863563 N_Shore TRUE CDKL1 1stExon
cg13705284 chr3 cg13705284 rs5021434 OpenSea TRUE ACOX2 TSS1500
cg23642747 chr10 cg23642747 NA chr10:105036628-105038084 Island TRUE INA TSS1500
cg25565479 chr11 cg25565479 NA chr11:93861560-93862773 Island TRUE PANX1 TSS1500

cg24868525 chr21 cg24868525 NA chr21:45431646-45432978 Island TRUE TRAPPC10 TSS1500
cg14870461 chr3 cg14870461 NA chr3:69062159-69063190 N_Shore TRUE C3orf64 5'UTR
cg24341800 chr11 cg24341800 NA chr11:107992004-107992731 Island TRUE ACAT1 TSS200
cg18441959 chr22 cg18441959 rs9619852 OpenSea TRUE VPREB1 Body
cg13112336 chr6 cg13112336 NA chr6:119255738-119256331 Island TRUE MCM9 5'UTR
cg00003994 chr7 cg00003994 NA OpenSea TRUE MEOX2 1stExon
cg22510943 chr19 cg22510943 rs75660485 chr19:2455863-2457078 Island TRUE LMNB2 Body
cg21664828 chr3 cg21664828 NA chr3:125093668-125094365 S_Shore TRUE ZNF148 TSS1500
cg11822659 chr20 cg11822659 NA chr20:53091733-53092993 Island TRUE DOK5 Body
cg19686152 chr15 cg19686152 NA chr15:52121816-52122452 N_Shore TRUE TMOD3 TSS1500
cg25661884 chr19 cg25661884 NA chr19:10120750-10121231 S_Shelf TRUE RDH8 1stExon
cg20959866 chr1 cg20959866 NA chr1:4713989-4716555 Island TRUE AJAP1 TSS1500
cg17860186 chr19 cg17860186 NA chr19:39903036-39904490 Island TRUE PLEKHG2 TSS1500
cg04574507 chr1 cg04574507 NA OpenSea TRUE CD1B Body
cg26125600 chr4 cg26125600 NA chr4:74719087-74719339 N_Shore TRUE PF4V1 TSS200
cg25315362 chr3 cg25315362 NA OpenSea TRUE LSAMP TSS200
cg21330703 chr11 cg21330703 rs1799978 chr11:113345068-113346328 S_Shore TRUE DRD2 TSS1500
cg08752459 chr12 cg08752459 NA OpenSea TRUE CLEC2B TSS1500

cg14950072 chr18 cg14950072 NA chr18:7116852-7118241 Island TRUE LAMA1 TSS1500
cg15149645 chr16 cg15149645 rs3785354 OpenSea TRUE NUPR1 TSS200
cg18618334 chr10 cg18618334 NA chr10:44879714-44882391 Island TRUE CXCL12 TSS1500
cg22123464 chr19 cg22123464 NA OpenSea TRUE SLC8A2 5'UTR
cg12624641 chr14 cg12624641 NA chr14:90420900-90421175 N_Shore TRUE C14orf143 Body
cg24921089 chr11 cg24921089 NA chr11:10472000-10472857 Island TRUE AMPD3 Body
cg01293647 chr17 cg01293647 NA chr17:37764092-37764304 Island TRUE NEUROD2 TSS200
cg12351433 chr2 cg12351433 NA chr2:48982621-48982958 Island TRUE LHCGR TSS200
cg07008386 chr20 cg07008386 NA chr20:8112884-8113592 S_Shore TRUE PLCB1 Body
cg10073091 chr1 cg10073091 NA chr1:55352463-55353328 N_Shore TRUE DHCR24 Body
cg05886626 chr15 cg05886626 NA chr15:39872527-39873567 Island TRUE THBS1 5'UTR
cg01892727 chr3 cg01892727 NA chr3:3221083-3221617 S_Shore TRUE CRBN TSS1500
cg15952487 chr1 cg15952487 NA OpenSea TRUE CD1B Body
cg18022496 chr3 cg18022496 NA chr3:196594576-196595486 N_Shore TRUE SENP5 TSS1500
cg06172871 chr16 cg06172871 rs5467 OpenSea TRUE HP TSS1500
cg05859264 chr6 cg05859264 rs9689522 chr6:36098039-36098621 N_Shore TRUE MAPK13 TSS1500
cg25524473 chr10 cg25524473 NA chr10:112257163-112258684 Island TRUE DUSP5 Body
cg22492020 chr15 cg22492020 NA chr15:66648784-66649178 N_Shore TRUE TIPIN 5'UTR

cg24315815 chr3 cg24315815 NA chr3:145968334-145969008 S_Shore TRUE PLSCR4 TSS1500
cg06378617 chr17 cg06378617 NA OpenSea TRUE KRT23 TSS1500
cg10925082 chr12 cg10925082 NA OpenSea TRUE ARHGDIB TSS200
cg09325101 chr15 cg09325101 NA OpenSea TRUE OSTBETA Body
cg09044743 chr1 cg09044743 NA chr1:2457241-2458259 Island TRUE PANK4 TSS1500
cg18815943 chr1 cg18815943 NA chr1:47881896-47883065 Island TRUE FOXE3 1stExon
cg23129478 chr18 cg23129478 NA chr18:44337510-44338100 Island TRUE ST8SIA5 TSS1500
cg00096922 chr7 cg00096922 NA chr7:96653467-96654199 Island TRUE DLX5 TSS200
cg20318748 chr20 cg20318748 rs35000100 chr20:25603815-25605187 Island TRUE NANP TSS1500
cg17992056 chr1 cg17992056 NA chr1:6295528-6296261 S_Shore TRUE ICMT TSS1500
cg08961832 chr11 cg08961832 rs760058 chr11:279072-281700 Island TRUE NLRP6 Body
cg03483626 chr1 cg03483626 NA chr1:111216244-111217937 S_Shore TRUE KCNA3 TSS1500
cg02588309 chr5 cg02588309 rs29742 chr5:40755537-40756257 S_Shore TRUE TTC33 TSS1500
cg27124774 chr5 cg27124774 NA chr5:140573423-140574316 N_Shore TRUE PCDHB10 TSS200
cg26620157 chr14 cg26620157 NA chr14:37131181-37132785 N_Shore TRUE PAX9 5'UTR
cg27185377 chr5 cg27185377 NA chr5:159848779-159849113 Island TRUE PTTG1 TSS200
cg13488201 chr10 cg13488201 NA chr10:128076155-128077482 Island TRUE ADAM12 TSS200
cg19826026 chr12 cg19826026 NA OpenSea TRUE ARHGDIB 5'UTR

cg23320056 chr1 cg23320056 NA chr1:155947678-155948490 S_Shore TRUE ARHGEF2 TSS1500

cg25124030 chr11 cg25124030 NA chr11:73498947-73499265 N_Shore TRUE MRPL48 TSS1500

cg26331247 chr20 cg26331247 NA OpenSea TRUE LOC284805 Body

cg17469978 chr7 cg17469978 NA chr7:116164703-116166735 Island TRUE CAV1 TSS200

cg13727946 chr19 cg13727946 NA OpenSea TRUE MBD3L1 1stExon

cg10334385 chr2 cg10334385 NA OpenSea TRUE C2orf50 TSS1500

cg18677965 chr20 cg18677965 rs73270104 chr20:47835783-47836067 S_Shore TRUE DDX27 Body

cg06751597 chr15 cg06751597 NA OpenSea TRUE SNAP23 5'UTR

cg17456704 chr19 cg17456704 NA chr19:12266998-12267686 S_Shore TRUE ZNF625 TSS1500

cg04123507 chr12 cg04123507 NA chr12:52695727-52696004 N_Shore TRUE KRT86 TSS1500

cg11801011 chr19 cg11801011 NA chr19:51221447-51222704 N_Shore TRUE SHANK1 TSS1500

cg09300114 chr17 cg09300114 NA chr17:73083866-73084495 Island TRUE SLC16A5 5'UTR

cg16933388 chr3 cg16933388 NA chr3:49591578-49592534 Island TRUE BSN Body

cg21168622 chr19 cg21168622 rs16983327 OpenSea TRUE ZNF350 5'UTR

cg26431343 chr22 cg26431343 NA chr22:32807825-32808285 S_Shore TRUE C22orf28 TSS1500

cg25230532 chr9 cg25230532 NA chr9:34646586-34647003 N_Shore TRUE GALT TSS200

cg17866455 chr15 cg17866455 NA chr15:85174489-85174787 N_Shore TRUE SCAND2 TSS1500

cg04797323 chr12 cg04797323 NA chr12:93963963-93967395 Island TRUE SOCS2 Body

cg05989054 chr19 cg05989054 NA chr19:1401092-1401784 S_Shore TRUE GAMT TSS1500
cg25465406 chr17 cg25465406 NA chr17:7905927-7907445 Island TRUE GUCY2D 5'UTR
cg23854009 chr19 cg23854009 NA chr19:58111229-58111770 N_Shore TRUE ZNF530 TSS200
cg07889201 chr20 cg07889201 NA chr20:2489190-2489644 S_Shore TRUE ZNF343 TSS1500
cg10525488 chr4 cg10525488 NA chr4:113066470-113067379 N_Shore TRUE C4orf32 TSS200
cg00308665 chr13 cg00308665 NA OpenSea TRUE HTR2A Body
cg10214058 chr5 cg10214058 rs116583376 chr5:154237008-154238362 Island TRUE CNOT8 TSS200
cg05026186 chr5 cg05026186 rs73795800 chr5:148520992-148521467 N_Shore TRUE ABLIM3 TSS200
cg14221171 chr7 cg14221171 rs2072100 chr7:97361132-97363018 Island TRUE TAC1 5'UTR
cg20773127 chr4 cg20773127 NA chr4:111397677-111398089 N_Shore TRUE ENPEP TSS200
cg19226099 chr20 cg19226099 rs61736060 chr20:54824312-54824584 N_Shore TRUE MC3R 1stExon
cg14188232 chr15 cg14188232 NA chr15:68723367-68724691 Island TRUE ITGA11 Body
cg14696870 chr1 cg14696870 NA OpenSea TRUE FCER1A TSS1500
cg20029201 chr11 cg20029201 NA chr11:118781060-118781732 S_Shore TRUE BCL9L TSS1500
cg01348086 chr5 cg01348086 NA OpenSea TRUE RGS14 Body
cg00678539 chr15 cg00678539 rs28656429 chr15:56757179-56757383 S_Shore TRUE MNS1 TSS1500
cg23877385 chr15 cg23877385 NA OpenSea TRUE GCNT3 5'UTR
cg26043257 chr4 cg26043257 NA chr4:15779998-15780729 Island TRUE CD38 1stExon

cg08088390 chr20 cg08088390 NA OpenSea TRUE DEFB125 TSS200
cg10140638 chr2 cg10140638 NA chr2:220173870-220174283 Island TRUE PTPRN TSS200
cg27196745 chr12 cg27196745 NA chr12:15475318-15475901 Island TRUE PTPRO TSS200
cg17791651 chr1 cg17791651 NA chr1:38510102-38513642 Island TRUE POU3F1 TSS1500
cg17356252 chr21 cg17356252 rs751032 chr21:45705428-45706044 Island TRUE AIRE TSS200
cg13526007 chr14 cg13526007 NA chr14:42075858-42076329 S_Shore TRUE LRFN5 TSS200
cg23547073 chr2 cg23547073 NA chr2:220082991-220084012 Island TRUE ABCB6 1stExon
cg16924616 chr7 cg16924616 NA chr7:96653467-96654199 Island TRUE DLX5 1stExon
cg25221625 chr22 cg25221625 NA OpenSea TRUE CRYBB3 5'UTR
cg23799313 chr1 cg23799313 NA chr1:26201722-26202516 N_Shelf TRUE PAQR7 TSS1500
cg06476337 chr5 cg06476337 rs2279516 chr5:52856665-52856870 N_Shore TRUE NDUFS4 1stExon
cg27015047 chr10 cg27015047 NA OpenSea TRUE DMBT1 Body
cg09686308 chr19 cg09686308 rs78608985 OpenSea TRUE CIB3 1stExon
cg06456512 chr15 cg06456512 NA chr15:69109708-69114001 Island TRUE ANP32A 1stExon
cg17803089 chr6 cg17803089 NA chr6:35419879-35420766 N_Shore TRUE FANCE TSS1500
cg03724463 chr3 cg03724463 NA chr3:42306149-42307520 Island TRUE CCK TSS200
cg06778853 chr1 cg06778853 NA chr1:6672786-6675741 N_Shore TRUE PHF13 TSS1500
cg19636861 chr19 cg19636861 NA OpenSea TRUE ZNF556 Body

cg02755525 chr16 cg02755525 NA chr16:47176787-47178446 Island TRUE
NETO2 Body
cg22533573 chr11 cg22533573 NA chr11:32452144-32452708 S_Shore TRUE
WT1 Body
cg26143719 chr22 cg26143719 NA OpenSea TRUE C1QTNF6
1stExon
cg04517429 chr4 cg04517429 NA chr4:155410623-155413393 S_Shore TRUE
DCHS2 TSS1500
cg02775617 chr10 cg02775617 NA chr10:32217353-32218392 N_Shore
TRUE ARHGAP12 5'UTR
cg01430807 chr10 cg01430807 NA chr10:102289150-102289742 S_Shore TRUE
NDUFB8 TSS200
cg24794531 chr3 cg24794531 NA chr3:142442702-142443978 Island TRUE
TRPC1 Body
cg14386312 chr19 cg14386312 rs114116776 chr19:58739943-58740554
S_Shore TRUE ZNF544 5'UTR
cg27281093 chr22 cg27281093 rs17207640 chr22:18632736-18632998
N_Shore TRUE USP18 TSS1500
cg21271753 chr22 cg21271753 NA OpenSea TRUE TMPRSS6 Body
cg19461621 chr18 cg19461621 NA chr18:499315-500722 S_Shore TRUE
COLEC12 TSS1500
cg07826255 chr17 cg07826255 NA OpenSea TRUE SGCA Body
cg09243900 chr1 cg09243900 NA OpenSea TRUE RAB25 TSS200
cg10734665 chr15 cg10734665 rs74004319 chr15:26107503-26108818
N_Shore TRUE ATP10A Body
cg17439694 chr3 cg17439694 NA OpenSea TRUE CP 1stExon
cg03848675 chr6 cg03848675 NA chr6:1389139-1391393 Island TRUE
FOXF2 TSS1500
cg19486673 chr19 cg19486673 rs73612407 OpenSea TRUE LILRA2
TSS1500
cg16639185 chr1 cg16639185 NA chr1:206785804-206786030 S_Shore TRUE
LGTN TSS1500
cg20209009 chr17 cg20209009 NA chr17:45810589-45811388 Island TRUE

TBX21 Body
cg01283289 chr12 cg01283289 NA chr12:81471569-81472119 S_Shore TRUE ACSS3 1stExon
cg21604856 chr22 cg21604856 NA OpenSea TRUE ZNF280A TSS200
cg07534467 chr8 cg07534467 NA chr8:146175835-146176337 Island TRUE ZNF16 TSS200
cg13271951 chr16 cg13271951 NA chr16:30042307-30042612 N_Shore TRUE FAM57B 1stExon
cg24454143 chr13 cg24454143 NA chr13:28674227-28675170 S_Shore TRUE FLT3 TSS1500
cg20134215 chr6 cg20134215 NA chr6:100441406-100442152 Island TRUE MCHR2 5'UTR
cg19657082 chr1 cg19657082 NA chr1:40723386-40723698 S_Shore TRUE ZMPSTE24 1stExon
cg18829411 chr21 cg18829411 NA chr21:40720185-40721625 S_Shore TRUE HMGN1 TSS1500
cg15853125 chr21 cg15853125 rs9975287 chr21:32929927-32932017 S_Shore TRUE TIAM1 TSS1500
cg15784615 chr12 cg15784615 rs11555387 chr12:6492889-6493522 S_Shore TRUE LTBR Body
cg06896207 chr19 cg06896207 NA chr19:2095395-2095727 N_Shore TRUE MOBKL2A 5'UTR
cg21740204 chr9 cg21740204 NA chr9:72873689-72874264 N_Shore TRUE SMC5 TSS1500
cg03266453 chr2 cg03266453 NA chr2:119606038-119606313 Island TRUE EN1 TSS1500
cg11504740 chr11 cg11504740 NA OpenSea TRUE GPR152 1stExon
cg12998491 chr9 cg12998491 NA chr9:134151853-134153015 Island TRUE FAM78A TSS1500
cg02314308 chr1 cg02314308 NA chr1:184005359-184006766 S_Shore TRUE GLT25D2 TSS200
cg24695828 chr19 cg24695828 NA chr19:36980190-36980733 Island TRUE

ZNF566 5'UTR

cg12827555 chr14 cg12827555 rs144911201 chr14:74706188-74708192 N_Shore TRUE VSX2 TSS200

cg22506059 chr22 cg22506059 rs139554376 chr22:37914768-37915883 S_Shore TRUE CARD10 TSS1500

cg22083639 chr8 cg22083639 NA chr8:70744098-70747441 S_Shore TRUE SLCO5A1 TSS1500

cg23440155 chr1 cg23440155 NA OpenSea TRUE CYP4B1 TSS200

cg22830895 chr7 cg22830895 NA chr7:151136933-151137273 S_Shore TRUE CRYGN TSS1500

cg02938601 chr8 cg02938601 NA chr8:22960384-22960927 S_Shore TRUE TNFRSF10C Body

cg18565355 chr8 cg18565355 NA chr8:95653898-95654733 N_Shore TRUE ESRP1 Body

cg14662379 chr2 cg14662379 NA chr2:241758141-241760783 Island TRUE KIF1A 5'UTR

cg24698622 chr8 cg24698622 NA chr8:30669450-30670475 S_Shore TRUE PPP2CB TSS1500

cg09503974 chr3 cg09503974 NA chr3:158449795-158450597 N_Shore TRUE RARRES1 Body

cg11812218 chr3 cg11812218 NA chr3:172167526-172167866 N_Shore TRUE GHSR TSS1500

cg06638433 chr17 cg06638433 NA chr17:47072820-47076042 Island TRUE IGF2BP1 1stExon

cg02512226 chr10 cg02512226 NA chr10:28287447-28288057 S_Shore TRUE ARMC4 TSS1500

cg11442717 chr6 cg11442717 rs17860886 OpenSea TRUE PHF10 TSS1500

cg07807709 chr1 cg07807709 NA chr1:149857769-149859470 S_Shore TRUE HIST2H2AB TSS1500

cg21126943 chr19 cg21126943 NA OpenSea TRUE CEACAM6 TSS200

cg10957151 chr2 cg10957151 NA chr2:176986424-176988291 Island TRUE
HOXD9 TSS1500
cg23037133 chr13 cg23037133 NA chr13:97646394-97646737 S_Shore TRUE
OXGR1 TSS1500
cg16678925 chr17 cg16678925 NA OpenSea TRUE OR1A2 1stExon
cg22199080 chr12 cg22199080 NA chr12:58130870-58132047 S_Shelf TRUE
AGAP2 1stExon
cg01333011 chr12 cg01333011 NA chr12:28123023-28123773 S_Shore TRUE
PTHLH 5'UTR
cg26531804 chr15 cg26531804 rs668750 chr15:41135719-41137210 Island
TRUE SPINT1 TSS1500
cg18602811 chr3 cg18602811 NA chr3:32611700-32612763 Island TRUE
DYNC1LI1 1stExon
cg24901042 chr21 cg24901042 NA chr21:42878751-42880674 Island TRUE
TMPRSS2 TSS1500
cg04457979 chr11 cg04457979 NA chr11:2890388-2891337 Island TRUE
KCNQ1DN TSS1500
cg26160573 chr2 cg26160573 NA chr2:131113090-131114277 N_Shore
TRUE PTPN18 TSS1500
cg27544190 chr21 cg27544190 NA chr21:33783914-33785717 Island TRUE
C21orf63 Body
cg08872742 chr16 cg08872742 NA OpenSea TRUE CDH5 TSS1500
cg13517305 chr3 cg13517305 NA chr3:126422888-126423609 Island TRUE
CHCHD6 Body
cg03192551 chr17 cg03192551 NA chr17:42402787-42403266 Island TRUE
SLC25A39 TSS1500
cg26727372 chr18 cg26727372 NA chr18:48405135-48405983 N_Shore
TRUE ME2 TSS1500
cg18501026 chr3 cg18501026 NA chr3:50712068-50713007 Island TRUE
DOCK3 TSS1500
cg16158681 chr16 cg16158681 NA chr16:56623068-56623914 Island TRUE
MT3 TSS200
cg14196790 chr5 cg14196790 NA chr5:131705169-131705971 N_Shore

TRUE SLC22A5 TSS1500
cg12069042 chr3 cg12069042 NA chr3:48470230-48471389 N_Shelf TRUE PLXNB1 5'UTR
cg19718882 chr11 cg19718882 NA chr11:32459422-32459878 N_Shore TRUE WIT1 Body
cg07363637 chr6 cg07363637 NA OpenSea TRUE SLC44A4 TSS200
cg05592398 chr7 cg05592398 rs710968 chr7:73498098-73498697 N_Shore TRUE LIMK1 TSS1500
cg00128877 chr1 cg00128877 NA chr1:47069615-47070179 Island TRUE MKNK1 TSS1500
cg12809314 chr3 cg12809314 NA chr3:44518838-44519368 N_Shore TRUE ZNF445 5'UTR
cg13565157 chr12 cg13565157 NA OpenSea TRUE PDE1B TSS200
cg16377880 chr19 cg16377880 NA OpenSea TRUE CYP4F3 TSS1500
cg10159529 chr3 cg10159529 rs334787 OpenSea TRUE IL5RA TSS1500
cg15746719 chr9 cg15746719 NA chr9:90112514-90113817 Island TRUE DAPK1 5'UTR
cg11199399 chr2 cg11199399 NA chr2:33171448-33173164 Island TRUE LTBP1 1stExon
cg06497752 chr20 cg06497752 NA chr20:61447685-61448891 Island TRUE COL9A3 Body
cg04375036 chr12 cg04375036 NA chr12:111180006-111181028 S_Shore TRUE PPP1CC TSS1500
cg00077877 chr8 cg00077877 NA OpenSea TRUE ASAP1 TSS1500
cg25119415 chr1 cg25119415 NA OpenSea TRUE MNDA TSS1500
cg11170179 chr19 cg11170179 rs77303625 chr19:51522004-51522803 Island TRUE KLK10 5'UTR
cg14833385 chr6 cg14833385 NA OpenSea TRUE HLA-DMA TSS1500
cg21926138 chr1 cg21926138 rs11102256 chr1:111889138-111889539 Island

TRUE C1orf88 Body
cg06722216 chr18 cg06722216 NA chr18:31802358-31803792 Island TRUE NOL4 1stExon
cg27398499 chr1 cg27398499 NA chr1:59042013-59043295 S_Shore TRUE TACSTD2 TSS1500
cg14141399 chr19 cg14141399 rs10417548 OpenSea TRUE HAS1 TSS1500
cg04478795 chr7 cg04478795 NA chr7:128828333-128829366 N_Shore TRUE SMO TSS1500
cg00563926 chr1 cg00563926 NA chr1:92350913-92352085 S_Shore TRUE TGFBR3 TSS1500
cg13494498 chr16 cg13494498 NA chr16:89990069-89990507 Island TRUE TUBB3 Body
cg16175792 chr1 cg16175792 NA OpenSea TRUE HSD3B1 Body
cg02205739 chr9 cg02205739 NA chr9:132427457-132427679 Island TRUE PRRX2 TSS1500
cg08097755 chr7 cg08097755 NA chr7:100806279-100809064 Island TRUE VGF 5'UTR
cg08085267 chr17 cg08085267 rs59392615 chr17:45400874-45401440 S_Shore TRUE C17orf57 5'UTR
cg05698069 chr8 cg05698069 NA chr8:22436295-22437076 N_Shore TRUE PDLIM2 TSS1500
cg03945800 chr16 cg03945800 rs3730097 chr16:4164808-4166258 Island TRUE ADCY9 5'UTR
cg07613278 chr11 cg07613278 NA chr11:43333439-43333887 N_Shore TRUE API5 TSS200
cg02096656 chr7 cg02096656 NA chr7:139024930-139026298 Island TRUE C7orf55 TSS1500
cg20789620 chr19 cg20789620 NA chr19:4304542-4305122 N_Shore TRUE FSD1 TSS1500
cg04438497 chr11 cg04438497 NA chr11:68517667-68518179 S_Shore TRUE MTL5 TSS1500
cg21663431 chr19 cg21663431 NA chr19:10735999-10736396 Island TRUE

SLC44A2 Body
cg06207460 chr9 cg06207460 NA chr9:4740694-4741868 Island TRUE
AK3 1stExon
cg15540820 chr3 cg15540820 rs751718 chr3:27765196-27765675 Island
TRUE EOMES TSS1500
cg05345286 chr6 cg05345286 NA chr6:41604583-41606663 Island TRUE
MDFI Body
cg01804429 chr4 cg01804429 NA chr4:69215304-69215921 S_Shore TRUE
YTHDC1 TSS1500
cg13818573 chr17 cg13818573 NA chr17:43044369-43046424 Island TRUE
C1QL1 1stExon
cg11263296 chr12 cg11263296 NA chr12:120426547-120428066 S_Shore TRUE
CCDC64 Body
cg25853078 chr11 cg25853078 NA OpenSea TRUE OPCML TSS1500
cg27566805 chr1 cg27566805 NA OpenSea TRUE USH2A TSS200
cg14056644 chr4 cg14056644 NA chr4:111560563-111560808 N_Shore
TRUE PITX2 TSS1500
cg20664247 chr1 cg20664247 NA chr1:2345891-2346156 N_Shore TRUE
PEX10 TSS1500
cg00546897 chr21 cg00546897 NA OpenSea TRUE LOC284837 Body
cg26599006 chr22 cg26599006 NA chr22:19136293-19138512 Island TRUE
GSC2 Body
cg09001953 chr4 cg09001953 NA chr4:186346794-186347571 S_Shore TRUE
UFSP2 TSS1500
cg13509147 chr19 cg13509147 rs73538174 OpenSea TRUE CREB3L3
TSS200
cg07214572 chr1 cg07214572 NA chr1:197880016-197881637 Island TRUE
LHX9 TSS1500
cg20780953 chr19 cg20780953 rs55945847 chr19:11450016-11450414
S_Shore TRUE RAB3D TSS200
cg00392257 chr1 cg00392257 NA chr1:156698111-156698916 N_Shore
TRUE ISG20L2 1stExon
cg18671950 chr15 cg18671950 NA chr15:48936810-48938577 Island TRUE

FBN1 Body
cg20870559 chr12 cg20870559 NA OpenSea TRUE OAS2 1stExon
cg23187653 chr5 cg23187653 NA chr5:95768874-95769080 Island TRUE
PCSK1 TSS200
cg23061578 chr5 cg23061578 NA chr5:523676-524887 S_Shore TRUE
SLC9A3 TSS1500
cg23412850 chr12 cg23412850 NA chr12:93963963-93967395 Island TRUE
SOCS2 Body
cg06638012 chr5 cg06638012 NA chr5:132298789-132299740 S_Shore TRUE
AFF4 TSS1500
cg08026144 chr9 cg08026144 NA chr9:78505230-78505483 Island TRUE
PCSK5 TSS1500
cg17204557 chr2 cg17204557 NA OpenSea TRUE LOC84931 Body
cg16604516 chr3 cg16604516 NA chr3:13590414-13591008 Island TRUE
FBLN2 TSS1500
cg18886444 chr3 cg18886444 NA chr3:49377340-49377854 S_Shore TRUE
USP4 TSS1500
cg16516400 chr1 cg16516400 NA chr1:231175062-231176317 S_Shore TRUE
FAM89A TSS1500
cg02388150 chr8 cg02388150 NA chr8:41165852-41167140 N_Shore TRUE
SFRP1 Body
cg26580095 chr1 cg26580095 NA chr1:92545819-92546480 N_Shore TRUE
BTBD8 TSS1500
cg11646887 chr17 cg11646887 NA chr17:45918094-45918774 S_Shore TRUE
SCRN2 TSS200
cg08843492 chr11 cg08843492 NA chr11:310728-311419 N_Shelf TRUE
IFITM2 1stExon
cg13461600 chr3 cg13461600 NA chr3:183903386-183904495 Island TRUE
ABCF3 TSS1500
cg12441928 chr3 cg12441928 rs34919616 OpenSea TRUE CCR1
TSS200
cg26610808 chr10 cg26610808 NA chr10:102046142-102046632 S_Shore TRUE
BLOC1S2 TSS1500

cg21472642	chr7	cg21472642	rs39050	chr7:29233704-29235046	N_Shore	TRUE	CHN2	TSS1500
cg14333454	chr1	cg14333454	NA	chr1:27189912-27190128	N_Shore	TRUE	SFN	TSS1500
cg06675478	chr13	cg06675478	NA	chr13:112720564-112723582	Island	TRUE	SOX1	TSS200
cg11380624	chr12	cg11380624	NA	chr12:56223263-56223598	N_Shore	TRUE	DNAJC14	5'UTR
cg11164400	chr7	cg11164400	NA	chr7:94536849-94537477	N_Shore	TRUE	PPP1R9A	TSS200
cg10515956	chr1	cg10515956	NA	chr1:85513509-85514555	S_Shore	TRUE	MCOLN3	TSS1500
cg17471102	chr19	cg17471102	NA		OpenSea	TRUE	FUT3	5'UTR
cg25836326	chr3	cg25836326	NA		OpenSea	TRUE	DRD3	TSS1500
cg14473924	chr3	cg14473924	rs76684526	chr3:73673230-73674375	Island	TRUE	PDZRN3	TSS200
cg02506908	chr12	cg02506908	NA		OpenSea	TRUE	HPD	TSS1500
cg02654291	chr9	cg02654291	NA	chr9:86571047-86572027	Island	TRUE	C9orf64	TSS1500
cg19596755	chr11	cg19596755	NA	chr11:68611250-68611807	N_Shore	TRUE	CPT1A	TSS1500
cg06397837	chr7	cg06397837	NA	chr7:27238690-27240311	Island	TRUE	HOXA13	TSS1500
cg20264732	chr16	cg20264732	NA	chr16:68269010-68272143	Island	TRUE	ESRP2	1stExon
cg06504820	chr14	cg06504820	NA	chr14:101192851-101193499	N_Shore	TRUE	DLK1	TSS1500
cg26960719	chr17	cg26960719	rs8076244		OpenSea	TRUE	KCNJ16	TSS200
cg19751300	chr18	cg19751300	NA	chr18:44336183-44337110	Island	TRUE	ST8SIA5	1stExon
cg10848367	chr11	cg10848367	NA		OpenSea	TRUE	SCGB1D2	TSS1500

cg11804789 chr20 cg11804789 rs61298216 OpenSea TRUE CST7 TSS200
cg22750254 chr10 cg22750254 NA chr10:13043094-13043781 Island TRUE CCDC3 1stExon
cg19014419 chr5 cg19014419 rs116669049 chr5:150284385-150284635 Island TRUE ZNF300 TSS200
cg24989962 chr14 cg24989962 NA chr14:52734207-52735486 Island TRUE PTGDR TSS200
cg16929104 chr19 cg16929104 rs141542836 chr19:18497063-18497269 N_Shore TRUE GDF15 TSS200
cg22285621 chr11 cg22285621 NA chr11:67070807-67071801 Island TRUE SSH3 Body
cg16248277 chr10 cg16248277 NA chr10:103535636-103536505 Island TRUE FGF8 TSS1500
cg02412050 chr6 cg02412050 NA chr6:35995245-35996372 Island TRUE MAPK14 TSS200
cg15376097 chr11 cg15376097 NA OpenSea TRUE MPZL2 TSS1500
cg20053158 chr12 cg20053158 NA chr12:103351579-103352695 N_Shore TRUE ASCL1 TSS1500
cg16352283 chr1 cg16352283 NA chr1:27338879-27339441 Island TRUE FAM46B 1stExon
cg14386691 chr14 cg14386691 NA chr14:23479310-23479532 S_Shore TRUE C14orf93 TSS1500
cg18420965 chr4 cg18420965 NA chr4:66535193-66535620 N_Shore TRUE EPHA5 Body
cg23114866 chr20 cg23114866 rs73127859 chr20:21485932-21496714 Island TRUE NKX2-2 TSS1500
cg00466249 chr17 cg00466249 NA chr17:79268367-79269467 S_Shore TRUE SLC38A10 TSS1500
cg21048669 chr16 cg21048669 NA OpenSea TRUE CLEC3A TSS200
cg09619786 chr15 cg09619786 NA chr15:75248277-75249922 Island TRUE RPP25 TSS200

cg21905630 chr4 cg21905630 NA chr4:54966163-54968063 N_Shore TRUE GSX2 TSS1500
cg15765212 chr6 cg15765212 NA chr6:31587779-31589024 S_Shore TRUE BAT2 5'UTR
cg13901526 chr2 cg13901526 NA chr2:127413696-127414171 S_Shore TRUE GYPC Body
cg08256781 chr19 cg08256781 NA OpenSea TRUE ACSBG2 TSS200
cg22880820 chr9 cg22880820 rs73436764 chr9:35665059-35665364 S_Shore TRUE C9orf100 TSS1500
cg14672994 chr17 cg14672994 NA chr17:48503056-48503887 Island TRUE ACSF2 TSS1500
cg19254558 chr3 cg19254558 rs6803120 chr3:183602432-183602874 N_Shore TRUE PARL Body
cg19093820 chr3 cg19093820 NA OpenSea TRUE GPR156 TSS200
cg13297865 chr6 cg13297865 NA chr6:80656744-80657593 Island TRUE ELOVL4 TSS200
cg10908369 chr2 cg10908369 NA chr2:68546339-68547111 S_Shore TRUE CNRIP1 TSS200
cg20673481 chr2 cg20673481 NA chr2:18059493-18060920 N_Shore TRUE KCNS3 TSS1500
cg05602212 chr5 cg05602212 NA chr5:79703436-79704444 N_Shore TRUE ZFYVE16 TSS1500
cg14865868 chr2 cg14865868 NA chr2:12856625-12859019 N_Shore TRUE TRIB2 TSS1500
cg21137823 chr1 cg21137823 NA chr1:1981251-1983212 Island TRUE PRKCZ TSS1500
cg18459342 chr8 cg18459342 NA chr8:81083320-81084057 Island TRUE TPD52 TSS1500
cg01580681 chr4 cg01580681 NA chr4:174450046-174451469 N_Shore TRUE HAND2 1stExon
cg04837898 chr3 cg04837898 rs4682797 chr3:45730615-45731294 Island TRUE SACM1L Body

cg12340144 chr3 cg12340144 rs112665255 chr3:38388122-38388769 S_Shore TRUE XYLB Body

cg04956511 chr12 cg04956511 NA chr12:7053149-7053927 S_Shelf TRUE PTPN6 Body

cg01200177 chr2 cg01200177 NA chr2:128615479-128616004 S_Shore TRUE POLR2D TSS1500

cg21187265 chr1 cg21187265 NA OpenSea TRUE SEPN1 Body

cg23886551 chr14 cg23886551 NA chr14:105992499-105996414 Island TRUE TMEM121 TSS1500

cg20595215 chr3 cg20595215 NA chr3:8543201-8543579 N_Shore TRUE LMCD1 TSS1500

cg25938646 chr13 cg25938646 NA chr13:84453664-84453897 S_Shelf TRUE SLITRK1 TSS200

cg02175308 chr1 cg02175308 NA chr1:109940074-109940894 S_Shore TRUE SORT1 TSS1500

cg25993718 chr20 cg25993718 NA chr20:54578603-54580678 Island TRUE CBLN4 TSS200

cg11750183 chr9 cg11750183 NA chr9:77566773-77568038 Island TRUE C9orf40 TSS1500

cg27223047 chr5 cg27223047 rs55770077 chr5:127872563-127874945 Island TRUE FBN2 TSS1500

cg19789466 chr12 cg19789466 NA OpenSea TRUE OAS1 1stExon

cg27177839 chr14 cg27177839 NA OpenSea TRUE JAG2 TSS1500

cg00554702 chr11 cg00554702 NA chr11:125495233-125495866 S_Shore TRUE CHEK1 5'UTR

cg01603095 chr12 cg01603095 NA chr12:40498962-40500017 S_Shore TRUE SLC2A13 TSS1500

cg06339706 chr19 cg06339706 NA OpenSea TRUE PLEKHA4 5'UTR

cg00547018 chr16 cg00547018 rs17523310 chr16:5121749-5122192 N_Shore TRUE ALG1 TSS200

cg02553486 chr4 cg02553486 NA chr4:47838604-47839946 S_Shore TRUE CORIN TSS200

cg11484872 chr6 cg11484872 NA OpenSea TRUE TNF TSS200

cg13619408 chr1 cg13619408 NA chr1:109756442-109757098 N_Shore TRUE SARS TSS1500
cg10883303 chr7 cg10883303 NA chr7:27238690-27240311 Island TRUE HOXA13 1stExon
cg03732545 chr1 cg03732545 NA OpenSea TRUE CNTN2 TSS1500
cg05270634 chr17 cg05270634 NA chr17:41177336-41177593 Island TRUE RND2 1stExon
cg15387123 chr9 cg15387123 NA chr9:139888934-139890343 S_Shore TRUE CLIC3 TSS200
cg01062029 chr9 cg01062029 NA chr9:94711117-94713075 Island TRUE ROR2 TSS1500
cg02932669 chr13 cg02932669 NA chr13:102068116-102069258 Island TRUE NALCN 5'UTR
cg23748737 chr22 cg23748737 NA chr22:20790638-20792665 Island TRUE SCARF2 Body
cg03292149 chr19 cg03292149 NA chr19:45281133-45281355 N_Shore TRUE CBLC TSS1500
cg01351032 chr16 cg01351032 NA chr16:10972782-10973305 N_Shore TRUE CIITA 1stExon
cg26514492 chr14 cg26514492 NA OpenSea TRUE GPR132 TSS200
cg03103192 chr4 cg03103192 NA chr4:52917388-52918280 N_Shore TRUE SPATA18 TSS1500
cg20070090 chr1 cg20070090 NA OpenSea TRUE S100A8 5'UTR
cg14289461 chr6 cg14289461 NA chr6:112575091-112575483 S_Shore TRUE LAMA4 TSS200
cg04809787 chr17 cg04809787 NA chr17:7348274-7348830 Island TRUE CHRNB1 TSS200
cg26667975 chr19 cg26667975 NA chr19:1236946-1238311 Island TRUE C19orf26 TSS1500
cg06533629 chr7 cg06533629 NA chr7:130417912-130419378 Island TRUE KLF14 TSS1500
cg14634738 chr3 cg14634738 NA chr3:50126252-50127204 N_Shore TRUE RBM5 TSS1500

cg09089053 chr19 cg09089053 NA chr19:40732075-40732665 N_Shore TRUE CNTD2 Body
cg03565323 chr17 cg03565323 NA chr17:16472074-16472800 S_Shore TRUE ZNF287 TSS1500
cg11063110 chr7 cg11063110 NA OpenSea TRUE CPA5 5'UTR
cg23214764 chr11 cg23214764 NA chr11:86511184-86511889 Island TRUE PRSS23 TSS1500
cg05937453 chr10 cg05937453 NA chr10:99531025-99531968 Island TRUE SFRP5 TSS200
cg26894575 chr1 cg26894575 NA OpenSea TRUE S100A4 5'UTR
cg08558340 chr7 cg08558340 NA chr7:100472375-100473393 N_Shore TRUE SRRT TSS1500
cg15050111 chr1 cg15050111 NA chr1:24648202-24648985 N_Shelf TRUE GRHL3 TSS200
cg11885098 chr19 cg11885098 NA chr19:1287416-1287885 N_Shelf TRUE EFNA2 TSS1500
cg24860534 chr1 cg24860534 NA chr1:227505446-227506752 S_Shore TRUE CDC42BPA TSS1500
cg11330108 chr18 cg11330108 NA chr18:60986621-60988286 N_Shore TRUE BCL2 5'UTR
cg17389519 chr10 cg17389519 NA chr10:23480697-23482455 Island TRUE PTF1A TSS1500
cg02310296 chr20 cg02310296 NA chr20:44639135-44639988 N_Shore TRUE MMP9 TSS200
cg05671350 chr11 cg05671350 NA chr11:74022428-74022703 N_Shore TRUE P4HA3 Body
cg16080552 chr1 cg16080552 NA chr1:59042013-59043295 Island TRUE TACSTD2 TSS200
cg06200697 chr11 cg06200697 NA OpenSea TRUE CTNND1 TSS1500
cg07845392 chr17 cg07845392 NA chr17:79678936-79679805 N_Shore TRUE SLC25A10 TSS1500
cg06425556 chr20 cg06425556 NA chr20:33412857-33413602 S_Shore TRUE

NCOA6 TSS1500

cg06908778 chr10 cg06908778 NA chr10:22634000-22634862 Island TRUE SPAG6 Body

cg27352992 chr12 cg27352992 NA chr12:41086522-41087102 Island TRUE CNTN1 5'UTR

cg26366091 chr1 cg26366091 NA OpenSea TRUE CHI3L2 TSS200

cg14163776 chr3 cg14163776 NA chr3:195162891-195164119 S_Shore TRUE ACAP2 TSS1500

cg09022993 chr4 cg09022993 NA chr4:52917388-52918280 Island TRUE SPATA18 1stExon

cg22791453 chr9 cg22791453 NA chr9:133319864-133320810 Island TRUE ASS1 TSS200

cg06953304 chr3 cg06953304 NA chr3:42641844-42642605 N_Shore TRUE NKTR TSS1500

cg21604803 chr19 cg21604803 NA chr19:50193020-50194798 Island TRUE CPT1C 5'UTR

cg20895877 chr14 cg20895877 rs78825507 chr14:94253952-94255733 Island TRUE PRIMA1 5'UTR

cg05881135 chr15 cg05881135 rs12324621 chr15:99645030-99646444 N_Shore TRUE SYNM TSS1500

cg16990174 chr3 cg16990174 rs2118593 chr3:72495853-72496852 S_Shore TRUE RYBP TSS1500

cg21360828 chr2 cg21360828 NA chr2:27008760-27009465 N_Shore TRUE CENPA TSS1500

cg19674669 chr11 cg19674669 NA chr11:134145559-134147180 Island TRUE GLB1L3 Body

cg23656386 chr16 cg23656386 NA chr16:68003407-68003759 N_Shore TRUE SLC12A4 TSS1500

cg09721427 chr10 cg09721427 NA chr10:94448693-94450479 Island TRUE HHEX TSS1500

cg16431978 chr21 cg16431978 NA OpenSea TRUE KRTAP13-3 1stExon

cg02748539 chr5 cg02748539 NA chr5:523676-524887 Island TRUE

SLC9A3 1stExon
cg14384532 chr15 cg14384532 NA chr15:88799522-88801017 Island TRUE
NTRK3 TSS1500
cg17031727 chr1 cg17031727 NA chr1:230778077-230778593 N_Shore
TRUE COG2 TSS1500
cg11896923 chr17 cg11896923 NA chr17:37823693-37824989 S_Shore TRUE
PNMT Body
cg22947000 chr16 cg22947000 NA OpenSea TRUE BCMO1 TSS200
cg06537829 chr17 cg06537829 NA chr17:16283927-16284768 Island TRUE
UBB TSS1500
cg23904249 chr11 cg23904249 rs561285 chr11:60609392-60609786
N_Shore TRUE CCDC86 TSS1500
cg24686358 chr1 cg24686358 NA chr1:40782220-40783145 Island TRUE
COL9A2 Body
cg05158615 chr7 cg05158615 NA chr7:24323558-24325080 Island TRUE
NPY TSS1500
cg13877915 chr19 cg13877915 rs11084545 chr19:58951214-58952250 Island
TRUE ZNF132 TSS200
cg17651821 chr6 cg17651821 NA chr6:27839698-27840069 S_Shore TRUE
HIST1H4L TSS1500
cg03929796 chr3 cg03929796 NA chr3:52231942-52232541 N_Shore TRUE
ALAS1 TSS1500
cg18818531 chr11 cg18818531 NA chr11:65666676-65668209 S_Shore TRUE
FOSL1 TSS1500
cg20451680 chr5 cg20451680 NA OpenSea TRUE ESM1 1stExon
cg01125463 chr6 cg01125463 NA chr6:42946310-42947127 N_Shore TRUE
PEX6 1stExon
cg17808849 chr16 cg17808849 rs57627126 chr16:56965734-56966476
N_Shore TRUE HERPUD1 TSS1500
cg15979932 chr17 cg15979932 NA OpenSea TRUE CUEDC1 5'UTR
cg02376163 chr5 cg02376163 NA chr5:147763283-147763666 N_Shore
TRUE FBXO38 TSS1500
cg15582789 chr9 cg15582789 rs66567256 chr9:99145524-99145849 Island

TRUE SLC35D2 Body
cg12374431 chr2 cg12374431 NA chr2:71126720-71129209 Island TRUE VAX2 TSS1500
cg18992688 chr1 cg18992688 NA chr1:206223537-206224028 N_Shore TRUE AVPR1B TSS1500
cg06742978 chr12 cg06742978 NA chr12:50451197-50451943 Island TRUE ACCN2 5'UTR
cg19914607 chr3 cg19914607 NA chr3:50242550-50243589 N_Shore TRUE SLC38A3 TSS200
cg24625388 chr10 cg24625388 rs73609219 chr10:21462128-21463808 S_Shore TRUE NEBL TSS1500
cg09072120 chr10 cg09072120 NA chr10:25241110-25241900 S_Shore TRUE PRTFDC1 TSS1500
cg06269753 chr8 cg06269753 NA chr8:72755783-72756667 Island TRUE MSC Body
cg10725344 chr7 cg10725344 NA OpenSea TRUE FAM180A TSS200
cg03082060 chr1 cg03082060 rs10489789 OpenSea TRUE LGALS8 TSS1500
cg05483509 chr16 cg05483509 NA chr16:2563430-2564903 Island TRUE ATP6V0C TSS1500
cg20401945 chr16 cg20401945 NA chr16:29912896-29913203 N_Shore TRUE ASPHD1 1stExon
cg20275133 chr1 cg20275133 NA chr1:1342004-1342952 Island TRUE MRPL20 Body
cg07642638 chr20 cg07642638 rs16991472 chr20:5891878-5892417 N_Shore TRUE CHGB TSS200
cg12024292 chr9 cg12024292 NA chr9:120175253-120177496 S_Shore TRUE ASTN2 TSS1500
cg07747970 chr17 cg07747970 NA chr17:35291899-35300875 Island TRUE LHX1 TSS1500
cg22325703 chr11 cg22325703 NA chr11:94134226-94134848 Island TRUE GPR83 1stExon

cg25999015 chr19 cg25999015 NA chr19:44037297-44038286 Island TRUE ZNF575 5'UTR
cg00834796 chr5 cg00834796 NA OpenSea TRUE JAKMIP2 5'UTR
cg20876010 chr1 cg20876010 NA chr1:64971358-64971604 S_Shore TRUE CACHD1 Body
cg18515587 chr1 cg18515587 NA OpenSea TRUE SELENBP1 Body
cg19639622 chr4 cg19639622 NA chr4:176922473-176922820 S_Shore TRUE GPM6A TSS200
cg24596472 chr8 cg24596472 NA chr8:144416712-144417054 S_Shore TRUE TOP1MT TSS1500
cg26672426 chr9 cg26672426 NA OpenSea TRUE PTGES Body
cg18636558 chr3 cg18636558 NA OpenSea TRUE SLC15A2 Body
cg04270799 chr12 cg04270799 NA chr12:57943662-57943926 S_Shore TRUE KIF5A 1stExon
cg19744952 chr19 cg19744952 NA chr19:42784074-42785542 S_Shelf TRUE CIC Body
cg12894126 chr9 cg12894126 NA chr9:134406025-134407173 N_Shore TRUE UCK1 Body
cg26304237 chr1 cg26304237 NA chr1:65731411-65731849 N_Shore TRUE DNAJC6 TSS200
cg19008809 chr3 cg19008809 NA chr3:53078956-53081101 Island TRUE SFMBT1 TSS1500
cg01888566 chr7 cg01888566 rs78305161 chr7:130126017-130126801 Island TRUE MEST 5'UTR
cg05249393 chr19 cg05249393 NA chr19:11200919-11201686 N_Shore TRUE LDLR TSS200
cg17966192 chr2 cg17966192 NA OpenSea TRUE SULT1C4 TSS1500
cg04528819 chr7 cg04528819 NA chr7:130417912-130419378 Island TRUE KLF14 1stExon
cg16632715 chr2 cg16632715 NA chr2:176971706-176972305 Island TRUE HOXD11 1stExon
cg14896516 chr7 cg14896516 NA chr7:30721372-30722445 Island TRUE

CRHR2 TSS1500
cg18110483 chr5 cg18110483 NA chr5:79330928-79331488 Island TRUE
THBS4 TSS200
cg18804206 chr17 cg18804206 NA chr17:18161711-18162316 Island TRUE
FLII TSS1500
cg24719984 chr12 cg24719984 NA chr12:82152320-82152674 S_Shore TRUE
PPFIA2 TSS1500
cg04330084 chr7 cg04330084 rs1917710 chr7:123174472-123174682
S_Shore TRUE IQUB TSS1500
cg18464137 chr7 cg18464137 NA OpenSea TRUE SEMA3E
TSS200
cg23557926 chr1 cg23557926 NA OpenSea TRUE CFH TSS200
cg22264436 chr17 cg22264436 rs117857467 chr17:41832717-41833195
S_Shelf TRUE SOST TSS1500
cg23918047 chr17 cg23918047 NA chr17:79828636-79830135 N_Shore
TRUE ARHGDIA 5'UTR
cg06834875 chr14 cg06834875 NA chr14:100111120-100111906 Island TRUE
HHIPL1 TSS1500
cg03544320 chr4 cg03544320 NA chr4:5894071-5895116 Island TRUE
CRMP1 1stExon
cg08124030 chr3 cg08124030 rs34567733 OpenSea TRUE TM4SF1
1stExon
cg04645342 chr1 cg04645342 NA chr1:72748471-72749736 N_Shore TRUE
NEGR1 1stExon
cg18508125 chr8 cg18508125 NA chr8:28351456-28352560 N_Shelf TRUE
FBXO16 5'UTR
cg26557658 chr1 cg26557658 NA chr1:20878894-20880592 N_Shore TRUE
FAM43B TSS200
cg11354906 chr4 cg11354906 NA chr4:154709512-154710827 Island TRUE
SFRP2 TSS200
cg18847227 chr3 cg18847227 NA chr3:4508608-4508964 S_Shore TRUE
SUMF1 TSS1500
cg04095468 chr9 cg04095468 NA chr9:91933284-91933907 Island TRUE

SECISBP2 Body
cg08596000 chr11 cg08596000 NA chr11:104034536-104035058 S_Shore TRUE
PDGFD TSS200
cg03190825 chr19 cg03190825 rs115355503 OpenSea TRUE CYP4F11
TSS200
cg05369166 chr1 cg05369166 rs7535216 chr1:204797610-204797930
N_Shore TRUE NFASC TSS1500
cg20587394 chr12 cg20587394 NA chr12:54332805-54333731 N_Shore
TRUE HOXC13 TSS1500
cg08242493 chr10 cg08242493 NA chr10:103577394-103578256 S_Shore TRUE
MGEA5 TSS200
cg12800028 chr6 cg12800028 NA chr6:110299365-110301267 Island TRUE
GPR6 TSS200
cg02196730 chr15 cg02196730 NA chr15:80189006-80189695 N_Shore
TRUE MTHFS Body
cg08598221 chr8 cg08598221 NA chr8:121823534-121824720 S_Shore TRUE
SNTB1 TSS1500
cg06171787 chr11 cg06171787 NA chr11:59578090-59578379 S_Shore TRUE
MRPL16 TSS200
cg25882366 chr17 cg25882366 NA chr17:46620367-46621373 S_Shore TRUE
HOXB2 1stExon
cg26781575 chr2 cg26781575 rs2594700 chr2:187558794-187559012
N_Shore TRUE FAM171B TSS1500
cg15059932 chr18 cg15059932 NA chr18:74533809-74535905 Island TRUE
ZNF236 TSS1500
cg12876594 chr9 cg12876594 NA chr9:35791584-35791924 Island TRUE
NPR2 TSS1500
cg25370441 chr4 cg25370441 NA OpenSea TRUE FLJ20184
TSS1500
cg18107072 chr16 cg18107072 rs9922191 chr16:67427284-67428950 Island
TRUE TPPP3 TSS1500
cg06459327 chr15 cg06459327 NA OpenSea TRUE NOP10 TSS200
cg03763616 chr19 cg03763616 rs77779859 OpenSea TRUE SPIB

TSS200
cg11816577 chr17 cg11816577 NA chr17:1302606-1303889 Island TRUE
YWHAE TSS1500
cg03557733 chr17 cg03557733 NA chr17:41984148-41985012 S_Shore TRUE
MPP2 TSS200
cg15982419 chr18 cg15982419 rs140451871 chr18:9474804-9475978 N_Shore
TRUE RALBP1 TSS1500
cg13084525 chr11 cg13084525 rs286926 OpenSea TRUE EHF
TSS1500
cg11919694 chr16 cg11919694 NA chr16:30381077-30381952 N_Shore
TRUE TBC1D10B 1stExon
cg11393848 chr1 cg11393848 rs12404537 OpenSea TRUE C1QC
TSS1500
cg05221167 chr19 cg05221167 NA chr19:9608804-9609343 S_Shore TRUE
ZNF560 TSS200
cg05358404 chr20 cg05358404 NA chr20:62288941-62290058 Island TRUE
RTEL1 TSS1500
cg13704548 chr15 cg13704548 NA chr15:65714742-65715571 Island TRUE
IGDCC4 Body
cg14419187 chr2 cg14419187 NA chr2:210636344-210636935 S_Shore TRUE
UNC80 Body
cg01311051 chr16 cg01311051 NA chr16:30064200-30064678 S_Shore TRUE
ALDOA 5'UTR
cg14671488 chr18 cg14671488 NA chr18:811649-812709 Island TRUE
YES1 5'UTR
cg04084157 chr7 cg04084157 NA chr7:100806279-100809064 Island TRUE
VGF TSS200
cg26954174 chr16 cg26954174 NA OpenSea TRUE NOD2 TSS1500
cg27234090 chr11 cg27234090 NA chr11:119252130-119252564 N_Shore
TRUE USP2 5'UTR
cg02361557 chr1 cg02361557 NA chr1:107682889-107684463 Island TRUE
NTNG1 5'UTR
cg09558502 chr1 cg09558502 NA OpenSea TRUE OVGP1 TSS200

cg04893119 chr8 cg04893119 NA OpenSea TRUE PI15 TSS1500
cg26256793 chr1 cg26256793 NA OpenSea TRUE COL11A1 TSS1500
cg15543551 chr3 cg15543551 NA OpenSea TRUE FGF12 5'UTR
cg24206256 chr5 cg24206256 NA chr5:162932454-162932946 N_Shelf TRUE MAT2B TSS200
cg12000587 chr17 cg12000587 rs11658984 chr17:30185764-30186580 S_Shore TRUE C17orf79 TSS1500
cg04689061 chr8 cg04689061 NA chr8:79428433-79428754 N_Shore TRUE PKIA TSS1500
cg08186362 chr20 cg08186362 NA chr20:60794179-60796121 Island TRUE HRH3 1stExon
cg03214212 chr7 cg03214212 NA chr7:16793188-16794100 Island TRUE TSPAN13 TSS200
cg20948472 chr5 cg20948472 NA OpenSea TRUE ZMAT2 TSS200
cg24662718 chr1 cg24662718 NA chr1:108507251-108507841 Island TRUE VAV3 1stExon
cg10604333 chr19 cg10604333 NA chr19:44529393-44529778 N_Shore TRUE ZNF222 TSS1500
cg03506489 chr11 cg03506489 NA chr11:30038522-30038823 Island TRUE KCNA4 TSS1500
cg22970435 chr6 cg22970435 NA OpenSea TRUE SPATS1 TSS200
cg05874450 chr6 cg05874450 NA chr6:105627406-105627830 N_Shore TRUE POPDC3 5'UTR
cg08548888 chr3 cg08548888 NA chr3:38179857-38180689 S_Shore TRUE MYD88 Body
cg00431549 chr12 cg00431549 rs76567129 OpenSea TRUE MGP TSS200
cg01441777 chr22 cg01441777 NA chr22:38712684-38713333 S_Shore TRUE CSNK1E TSS1500
cg09988116 chr1 cg09988116 NA OpenSea TRUE C1orf210 5'UTR
cg02064106 chr6 cg02064106 NA OpenSea TRUE C6orf118 Body
cg01986577 chr12 cg01986577 NA chr12:27485683-27486676 N_Shore

TRUE ARNTL2 TSS1500
cg25985103 chr15 cg25985103 NA chr15:75660388-75661060 Island TRUE MAN2C1 Body
cg21604042 chr6 cg21604042 NA chr6:35181095-35182547 Island TRUE SCUBE3 TSS1500
cg04335339 chr10 cg04335339 rs192237837 chr10:116852261-116854094 Island TRUE ATRNL1 TSS200
cg20131596 chr5 cg20131596 NA chr5:102201583-102201912 N_Shore TRUE PAM TSS1500
cg16120811 chr11 cg16120811 NA chr11:9594346-9596536 Island TRUE WEE1 TSS1500
cg19103704 chr19 cg19103704 NA OpenSea TRUE FCGBP TSS1500
cg10539507 chr4 cg10539507 NA chr4:85417659-85420799 Island TRUE NKX6-1 TSS1500
cg15319457 chr2 cg15319457 NA chr2:239147592-239149900 Island TRUE HES6 Body
cg23983173 chr15 cg23983173 NA chr15:42565452-42565791 N_Shore TRUE TMEM87A Body
cg12603560 chr11 cg12603560 NA chr11:57091614-57092831 N_Shore TRUE TNKS1BP1 5'UTR
cg02630207 chr2 cg02630207 rs7596942 chr2:45838292-45838582 S_Shore TRUE SRBD1 TSS1500
cg13218435 chr4 cg13218435 NA chr4:152682041-152682293 S_Shore TRUE PET112L TSS1500
cg03954587 chr17 cg03954587 NA chr17:16283927-16284768 Island TRUE UBB 5'UTR
cg06379754 chr7 cg06379754 NA chr7:82072021-82073520 S_Shore TRUE CACNA2D1 TSS1500
cg18722841 chr11 cg18722841 NA chr11:71954816-71955659 Island TRUE PHOX2A 1stExon
cg17251713 chr18 cg17251713 NA OpenSea TRUE SERPINB7 5'UTR
cg03243946 chr12 cg03243946 NA chr12:21810488-21810766 S_Shore TRUE LDHB TSS1500

cg00970325 chr3 cg00970325 NA chr3:142681137-142683268 Island TRUE PAQR9 TSS1500
cg12613383 chr1 cg12613383 NA chr1:242687922-242688682 Island TRUE PLD5 TSS1500
cg20492933 chr5 cg20492933 rs28372688 chr5:154317459-154317930 S_Shelf TRUE MRPL22 TSS200
cg27138584 chr2 cg27138584 NA chr2:47796923-47799166 N_Shore TRUE KCNK12 Body
cg16188243 chr19 cg16188243 rs73522750 chr19:2785211-2785839 N_Shore TRUE SGTA TSS1500
cg03876618 chr4 cg03876618 NA chr4:57975860-57976916 Island TRUE IGFBP7 Body
cg09309269 chr17 cg09309269 NA chr17:30770960-30772137 Island TRUE PSMD11 TSS1500
cg26898166 chr2 cg26898166 rs112471859 chr2:73143055-73148260 Island TRUE EMX1 TSS1500
cg08911391 chr1 cg08911391 rs531268 chr1:20987516-20988238 S_Shore TRUE DDOST TSS1500
cg06656924 chr6 cg06656924 NA chr6:35226897-35227851 S_Shore TRUE ZNF76 5'UTR
cg08392591 chr16 cg08392591 NA chr16:89555775-89557753 Island TRUE ANKRD11 5'UTR
cg14039952 chr5 cg14039952 NA chr5:170814447-170815655 Island TRUE NPM1 Body
cg19995014 chr19 cg19995014 NA chr19:36359232-36359775 Island TRUE APLP1 Body
cg02867079 chr14 cg02867079 NA chr14:100111120-100111906 Island TRUE HHIPL1 TSS1500
cg24697184 chr1 cg24697184 NA chr1:55266277-55267058 Island TRUE TTC22 1stExon
cg04785227 chr15 cg04785227 NA chr15:32322164-32323060 N_Shore TRUE CHRNA7 TSS1500
cg00029826 chr14 cg00029826 rs75163418 OpenSea TRUE CCNB1IP1

TSS200
cg19579782 chr10 cg19579782 NA chr10:17243304-17243787 S_Shore TRUE TRDMT1 TSS200
cg23003881 chr5 cg23003881 rs1469068 chr5:173043123-173043895 S_Shore TRUE BOD1 TSS1500
cg05389335 chr4 cg05389335 NA chr4:104640559-104640797 S_Shore TRUE TACR3 TSS1500
cg27329371 chr17 cg27329371 rs4646784 chr17:19648139-19648492 S_Shelf TRUE ALDH3A1 TSS200
cg20977864 chr16 cg20977864 NA chr16:71264360-71264659 N_Shore TRUE HYDIN 5'UTR
cg09134747 chr3 cg09134747 rs79477674 chr3:130612285-130613485 Island TRUE ATP2C1 TSS1500
cg21838334 chr1 cg21838334 rs4278369 chr1:156646292-156647260 Island TRUE NES 1stExon
cg25422943 chr13 cg25422943 NA chr13:67804593-67805459 Island TRUE PCDH9 TSS1500
cg05855071 chr15 cg05855071 NA chr15:34501701-34502499 Island TRUE C15orf29 5'UTR
cg05158538 chr4 cg05158538 NA OpenSea TRUE PPARGC1A Body
cg14844130 chr22 cg14844130 NA OpenSea TRUE VPREB1 TSS1500
cg03682712 chr15 cg03682712 NA chr15:74218696-74220373 Island TRUE LOXL1 1stExon
cg25854162 chr2 cg25854162 NA chr2:217497811-217498847 N_Shore TRUE IGFBP2 TSS1500
cg09109450 chr2 cg09109450 NA chr2:21022564-21022934 Island TRUE C2orf43 5'UTR
cg07621046 chr10 cg07621046 NA chr10:118429430-118429853 Island TRUE C10orf82 TSS200
cg08009622 chr6 cg08009622 NA chr6:75914705-75916387 Island TRUE COL12A1 TSS1500
cg25372693 chr3 cg25372693 NA chr3:118959339-118959934 S_Shore TRUE

B4GALT4 TSS1500
cg21869532 chr19 cg21869532 NA chr19:9731734-9731994 S_Shore TRUE ZNF561 TSS200
cg00888007 chr12 cg00888007 NA chr12:4378366-4382222 Island TRUE CCND2 TSS1500
cg02581667 chr19 cg02581667 NA chr19:39322264-39322574 N_Shore TRUE ECH1 Body
cg00410898 chr8 cg00410898 NA OpenSea TRUE STC1 TSS200
cg08301503 chr6 cg08301503 NA chr6:33560892-33561189 Island TRUE C6orf227 TSS200
cg03663715 chr5 cg03663715 NA chr5:72746989-72747587 N_Shelf TRUE FOXD1 TSS1500
cg04949741 chr11 cg04949741 NA chr11:65639835-65641374 Island TRUE EFEMP2 TSS200
cg20134151 chr6 cg20134151 NA chr6:150463771-150465002 N_Shore TRUE PPP1R14C TSS1500
cg08775230 chr11 cg08775230 NA OpenSea TRUE C11orf52 TSS200
cg07115304 chr20 cg07115304 NA chr20:361489-362805 Island TRUE TRIB3 5'UTR
cg27554782 chr15 cg27554782 NA chr15:78933187-78933821 N_Shore TRUE CHRNB4 Body
cg06491116 chr11 cg06491116 NA OpenSea TRUE MPZL3 Body
cg05065037 chr9 cg05065037 NA chr9:88969007-88969904 S_Shore TRUE ZCCHC6 TSS1500
cg26806924 chr20 cg26806924 NA chr20:33762403-33762774 N_Shelf TRUE PROCR 1stExon
cg10481740 chr4 cg10481740 NA chr4:1857065-1858887 Island TRUE LETM1 Body
cg00775197 chr6 cg00775197 NA chr6:44264970-44265711 Island TRUE TCTE1 5'UTR
cg12420104 chr9 cg12420104 NA chr9:976162-977828 Island TRUE DMRT3 1stExon

cg15536490 chr20 cg15536490 rs66891256 chr20:55200279-55206789 Island TRUE TFAP2C TSS1500
cg18221862 chr2 cg18221862 NA chr2:193058995-193060812 Island TRUE TMEFF2 1stExon
cg12513379 chr19 cg12513379 NA chr19:15838996-15839277 N_Shore TRUE OR10H2 TSS1500
cg25720804 chr5 cg25720804 NA chr5:170735169-170739863 Island TRUE TLX3 1stExon
cg26045434 chr8 cg26045434 NA chr8:21987532-21989013 Island TRUE HR 5'UTR
cg11882252 chr5 cg11882252 NA chr5:10307520-10307913 Island TRUE CMBL 5'UTR
cg01425670 chr1 cg01425670 NA chr1:72748471-72749736 Island TRUE NEGR1 TSS1500
cg24713204 chr19 cg24713204 NA chr19:57018743-57019506 Island TRUE ZNF471 5'UTR
cg17307280 chr5 cg17307280 NA chr5:174870753-174872345 Island TRUE DRD1 TSS1500
cg03752628 chr1 cg03752628 NA chr1:117452203-117453452 S_Shore TRUE PTGFRN Body
cg17272843 chr11 cg17272843 rs78436350 OpenSea TRUE KCTD14 1stExon
cg01027739 chr9 cg01027739 NA chr9:131842931-131843748 N_Shore TRUE DOLPP1 TSS1500
cg00480115 chr19 cg00480115 NA OpenSea TRUE FXYD3 5'UTR
cg16504798 chr19 cg16504798 NA OpenSea TRUE MYO1F TSS1500
cg02913089 chr15 cg02913089 rs117370946 chr15:85523512-85525865 Island TRUE PDE8A Body
cg13885201 chr16 cg13885201 NA chr16:71495773-71496260 S_Shore TRUE ZNF23 TSS1500
cg00105253 chr14 cg00105253 NA chr14:105647469-105647827 N_Shore TRUE NUDT14 Body
cg26833602 chr9 cg26833602 NA chr9:95820744-95821526 S_Shore TRUE

SUSD3 Body
cg01530101 chr11 cg01530101 NA chr11:2890388-2891337 Island TRUE KCNQ1DN TSS200
cg24130043 chr3 cg24130043 NA chr3:44666477-44666820 N_Shore TRUE ZNF197 TSS200
cg12515638 chr7 cg12515638 NA chr7:37955622-37956555 Island TRUE SFRP4 1stExon
cg10078415 chr16 cg10078415 NA OpenSea TRUE ACSM3 TSS1500
cg17565490 chr20 cg17565490 rs202224991 chr20:3766343-3767636 Island TRUE CENPB 1stExon
cg06214007 chr1 cg06214007 NA OpenSea TRUE GBP6 TSS200
cg09226684 chr19 cg09226684 NA chr19:51520153-51520907 N_Shore TRUE KLK10 Body
cg20330296 chr19 cg20330296 NA chr19:1383437-1384251 N_Shore TRUE NDUFS7 TSS1500
cg11241627 chr7 cg11241627 NA chr7:19184221-19184686 S_Shore TRUE FERD3L TSS200
cg18008766 chr2 cg18008766 NA chr2:38977535-38978684 S_Shore TRUE SFRS7 TSS1500
cg09396217 chr8 cg09396217 NA OpenSea TRUE ANGPT1 TSS200
cg26486663 chr16 cg26486663 NA chr16:2041825-2043026 N_Shore TRUE SYNGR3 Body
cg12339802 chr1 cg12339802 NA chr1:38157295-38158586 N_Shore TRUE C1orf109 TSS1500
cg20287640 chr5 cg20287640 NA chr5:131825842-131826935 N_Shore TRUE IRF1 5'UTR
cg02121427 chr3 cg02121427 rs78310992 OpenSea TRUE LRRC15 TSS1500
cg19362572 chr15 cg19362572 NA chr15:49254984-49255564 S_Shore TRUE SHC4 TSS1500
cg26764244 chr1 cg26764244 rs7540541 chr1:68298371-68299315 S_Shore TRUE GNG12 TSS1500

cg06509239 chr2 cg06509239 NA chr2:19555789-19558403 S_Shore TRUE
OSR1 TSS200
cg11639651 chr10 cg11639651 NA OpenSea TRUE HKDC1 TSS1500
cg04270835 chr11 cg04270835 NA chr11:22362862-22363377 N_Shelf TRUE
SLC17A6 TSS1500
cg11990309 chr6 cg11990309 NA chr6:87647253-87647707 Island TRUE
HTR1E 5'UTR
cg08090640 chr17 cg08090640 NA OpenSea TRUE IFI35 Body
cg10414946 chr11 cg10414946 NA OpenSea TRUE MS4A2 Body
cg06423920 chr3 cg06423920 NA chr3:62357639-62359774 Island TRUE
FEZF2 TSS1500
cg22764338 chr5 cg22764338 NA chr5:141255564-141257968 Island TRUE
PCDH1 Body
cg01242619 chr8 cg01242619 NA chr8:145047293-145052305 Island TRUE
PLEC1 Body
cg24453664 chr11 cg24453664 NA chr11:33757476-33758122 S_Shore TRUE
CD59 TSS1500
cg23881725 chr3 cg23881725 NA chr3:38080628-38081187 Island TRUE
DLEC1 TSS200
cg24030630 chr11 cg24030630 NA chr11:67806251-67806611 S_Shore TRUE
TCIRG1 5'UTR
cg20498685 chr7 cg20498685 NA chr7:19156050-19158042 Island TRUE
TWIST1 3'UTR
cg08395365 chr3 cg08395365 NA chr3:182816771-182817455 S_Shore TRUE
MCCC1 TSS200
cg12758687 chr11 cg12758687 rs1799978 chr11:113345068-113346328 Island
TRUE DRD2 TSS1500
cg09874127 chr3 cg09874127 NA OpenSea TRUE UBA7 TSS200
cg08008403 chr1 cg08008403 NA chr1:197880016-197881637 S_Shore TRUE
LHX9 Body
cg15864184 chr20 cg15864184 NA chr20:49126617-49127435 Island TRUE
PTPN1 TSS200
cg08886154 chr7 cg08886154 NA OpenSea TRUE PAX4 TSS1500

cg27294629 chr5 cg27294629 NA chr5:176513886-176514433 N_Shore TRUE FGFR4 TSS1500
cg19769182 chr16 cg19769182 NA chr16:29823163-29823906 Island TRUE PRRT2 5'UTR
cg09423836 chr4 cg09423836 NA chr4:77069066-77069672 S_Shore TRUE NUP54 TSS1500
cg26500816 chr17 cg26500816 NA OpenSea TRUE AIPL1 TSS1500
cg22139878 chr10 cg22139878 NA chr10:91174405-91175166 N_Shore TRUE IFIT5 TSS200
cg05091653 chr2 cg05091653 NA OpenSea TRUE SP100 Body
cg10377764 chr12 cg10377764 NA chr12:1058481-1059430 Island TRUE RAD52 TSS1500
cg20398399 chr3 cg20398399 rs9815899 OpenSea TRUE KLHL6 TSS1500
cg13325529 chr17 cg13325529 NA chr17:8079490-8080125 Island TRUE TMEM107 TSS1500
cg06653796 chr20 cg06653796 NA chr20:62368955-62371962 N_Shore TRUE LIME1 TSS200
cg13140267 chr2 cg13140267 rs59525841 chr2:96971125-96971556 S_Shore TRUE SNRNP200 TSS1500
cg09227563 chr19 cg09227563 NA chr19:54982388-54982897 S_Shore TRUE CDC42EP5 TSS1500
cg19586576 chr17 cg19586576 rs147136062 chr17:42906810-42908061 Island TRUE GJC1 5'UTR
cg01236137 chr5 cg01236137 NA chr5:32585603-32586365 N_Shore TRUE SUB1 TSS200
cg26036443 chr19 cg26036443 NA chr19:48216486-48216837 N_Shore TRUE EHD2 TSS200
cg17001035 chr10 cg17001035 NA chr10:92980347-92980832 N_Shore TRUE PCGF5 TSS1500
cg09102409 chr8 cg09102409 NA chr8:134308328-134310145 Island TRUE NDRG1 5'UTR
cg10059959 chr9 cg10059959 rs116283142 chr9:37034135-37038341 Island

TRUE PAX5 TSS1500
cg10896774 chr7 cg10896774 rs6943031 OpenSea TRUE C7orf34 1stExon
cg18454685 chr17 cg18454685 rs138906232chr17:48636103-48639279 Island TRUE CACNA1G Body
cg06638966 chr6 cg06638966 rs28433654 chr6:70576974-70577572 N_Shore TRUE COL19A1 TSS200
cg25511429 chr6 cg25511429 NA chr6:6008857-6009299 N_Shore TRUE NRN1 TSS1500
cg09529667 chr19 cg09529667 NA chr19:18527651-18531118 Island TRUE SSBP4 Body
cg20312687 chr20 cg20312687 NA OpenSea TRUE DEFB118 Body
cg11668844 chr13 cg11668844 rs3742239 chr13:113656116-113656379 N_Shore TRUE MCF2L Body
cg06277481 chr19 cg06277481 NA chr19:8407674-8408338 Island TRUE KANK3 TSS200
cg06121469 chr15 cg06121469 NA chr15:44955291-44955983 S_Shore TRUE SPG11 TSS1500
cg02019333 chr3 cg02019333 NA OpenSea TRUE UPK1B TSS1500
cg15683488 chr1 cg15683488 NA chr1:52607400-52608504 Island TRUE ZFYVE9 TSS1500
cg18275051 chr1 cg18275051 NA chr1:202936045-202936252 S_Shore TRUE CYB5R1 TSS1500
cg06609049 chr19 cg06609049 NA chr19:2785211-2785839 N_Shore TRUE THOP1 TSS1500
cg26222229 chr6 cg26222229 NA chr6:36842308-36842845 Island TRUE PPIL1 TSS200
cg21602520 chr18 cg21602520 NA chr18:60985503-60985741 N_Shore TRUE BCL2 Body
cg16025584 chr9 cg16025584 NA chr9:139888934-139890343 N_Shore TRUE C9orf142 Body
cg24428760 chr20 cg24428760 NA chr20:32580799-32582502 Island TRUE RALY 5'UTR

cg25523753 chr15 cg25523753 NA chr15:89631546-89632209 N_Shore TRUE ABHD2 TSS200
cg05010967 chr6 cg05010967 rs883194 OpenSea TRUE SPATS1 5'UTR
cg10742801 chr10 cg10742801 NA OpenSea TRUE PRAP1 Body
cg17775235 chr17 cg17775235 NA chr17:78449507-78452783 Island TRUE NPTX1 TSS1500
cg16427670 chr13 cg16427670 NA OpenSea TRUE ARHGEF7 TSS1500
cg05628549 chr11 cg05628549 NA chr11:6340445-6341909 Island TRUE PRKCDBP TSS200
cg03565081 chr14 cg03565081 NA chr14:23479310-23479532 N_Shore TRUE C14orf93 5'UTR
cg07537523 chr12 cg07537523 NA chr12:25101607-25102073 S_Shore TRUE BCAT1 TSS200
cg01407797 chr22 cg01407797 NA chr22:29168628-29169048 N_Shore TRUE CCDC117 TSS200
cg15435730 chr6 cg15435730 NA chr6:50682334-50683214 N_Shelf TRUE TFAP2D TSS1500
cg18089852 chr5 cg18089852 NA chr5:76506029-76507189 Island TRUE PDE8B 1stExon
cg04101379 chr13 cg04101379 NA chr13:96296143-96297059 S_Shore TRUE DZIP1 TSS1500
cg01404615 chr4 cg01404615 NA chr4:107956555-107957453 S_Shore TRUE DKK2 TSS1500
cg15379887 chr3 cg15379887 NA chr3:88107550-88108509 N_Shore TRUE CGGBP1 5'UTR
cg19006008 chr19 cg19006008 NA chr19:17000627-17001398 N_Shore TRUE F2RL3 TSS200
cg18438777 chr4 cg18438777 NA chr4:164264821-164265772 Island TRUE NPY5R 5'UTR
cg08530414 chr11 cg08530414 NA chr11:35160375-35161000 Island TRUE CD44 TSS200

cg15972294 chr3 cg15972294 NA chr3:50273268-50273901 N_Shore TRUE GNAI2 TSS1500

cg09038885 chr5 cg09038885 NA chr5:159343215-159343574 N_Shore TRUE ADRA1B TSS1500

cg11961618 chr11 cg11961618 NA chr11:2949788-2951756 S_Shore TRUE PHLDA2 TSS1500

cg27433088 chr4 cg27433088 rs1054497 chr4:174090074-174091423 N_Shore TRUE GALNT7 TSS1500

cg24022301 chr1 cg24022301 rs17101783 chr1:78956624-78957516 N_Shore TRUE PTGFR TSS200

cg19761273 chr17 cg19761273 NA chr17:80231019-80231820 S_Shore TRUE CSNK1D TSS1500

cg22747092 chr9 cg22747092 NA chr9:138985837-138987846 Island TRUE NACC2 TSS1500

cg21296230 chr15 cg21296230 rs142114172 chr15:33009530-33011696 Island TRUE GREM1 5'UTR

cg17190608 chr12 cg17190608 NA chr12:111471061-111471578 Island TRUE CUX2 TSS1500

cg06043042 chr10 cg06043042 NA chr10:71331926-71333392 Island TRUE NEUROG3 TSS200

cg01520924 chr14 cg01520924 NA chr14:65006874-65009197 N_Shore TRUE HSPA2 TSS1500

cg04527363 chr3 cg04527363 NA chr3:6902823-6903516 N_Shore TRUE GRM7 TSS1500

cg23123362 chr5 cg23123362 NA chr5:157158378-157158856 N_Shore TRUE THG1L TSS200

cg18053505 chr3 cg18053505 NA chr3:20081458-20082426 N_Shore TRUE KAT2B TSS1500

cg17387870 chr12 cg17387870 NA chr12:133463807-133464858 N_Shore TRUE CHFR Body

cg21091679 chr5 cg21091679 NA chr5:95296368-95297438 S_Shore TRUE ELL2 TSS1500

cg11612345 chr6 cg11612345 NA chr6:168841818-168843100 Island TRUE

SMOC2 Body

cg17602451 chr18 cg17602451 rs61733416 chr18:60985503-60985741 Island TRUE BCL2 Body

cg18142353 chr12 cg18142353 NA chr12:12419679-12420541 S_Shore TRUE LRP6 TSS1500

cg09593286 chr15 cg09593286 NA chr15:45926877-45927758 Island TRUE SQRDL TSS1500

cg20420433 chr7 cg20420433 NA chr7:156802170-156804414 S_Shore TRUE MNX1 TSS1500

cg10891879 chr1 cg10891879 NA chr1:10753773-10754176 Island TRUE CASZ1 Body

cg01389761 chr3 cg01389761 NA chr3:169482338-169483052 Island TRUE TERC TSS200

cg07141002 chr22 cg07141002 NA chr22:38200672-38201691 Island TRUE H1F0 1stExon

cg07499072 chr5 cg07499072 NA chr5:52777788-52777996 N_Shore TRUE FST 1stExon

cg12645220 chr20 cg12645220 NA chr20:9819271-9819861 Island TRUE PAK7 5'UTR

cg10685945 chr4 cg10685945 NA chr4:84457115-84457860 Island TRUE AGPAT9 1stExon

cg02409351 chr12 cg02409351 NA chr12:85673878-85674700 Island TRUE ALX1 TSS200

cg02883230 chr15 cg02883230 NA chr15:72564442-72565165 N_Shore TRUE PARP6 TSS1500

cg14254380 chr1 cg14254380 NA OpenSea TRUE WDR63 TSS200

cg00259755 chr10 cg00259755 rs77301799 chr10:134209367-134211056 Island TRUE PWWP2B TSS1500

cg25388528 chr21 cg25388528 rs1507391 OpenSea TRUE KRTAP20-1 TSS1500

cg15223781 chr5 cg15223781 NA chr5:108083987-108085203 N_Shore TRUE FER 5'UTR

cg19147390 chr19 cg19147390 NA chr19:4909262-4910256 S_Shore TRUE

UHRF1 Body
cg01794265 chr5 cg01794265 NA chr5:14581497-14582569 Island TRUE FAM105A Body
cg17125623 chr16 cg17125623 NA chr16:68118564-68119986 Island TRUE NFATC3 Body
cg19035457 chr4 cg19035457 rs10155441 chr4:56261697-56262503 S_Shore TRUE TMEM165 Body
cg22815110 chr1 cg22815110 rs2274187 chr1:63782394-63790471 Island TRUE FOXD3 1stExon
cg10331779 chr5 cg10331779 NA chr5:11903550-11904703 S_Shore TRUE CTNND2 TSS1500
cg03005261 chr4 cg03005261 NA chr4:110480827-110482060 Island TRUE CCDC109B TSS1500
cg07671976 chr1 cg07671976 NA chr1:1141670-1142150 S_Shore TRUE TNFRSF18 TSS200
cg20530056 chr1 cg20530056 NA OpenSea TRUE IKBKE TSS1500
cg03684977 chr17 cg03684977 NA OpenSea TRUE GRB7 TSS1500
cg11516377 chr17 cg11516377 NA chr17:1933138-1933852 Island TRUE DPH1 TSS1500
cg20300246 chr9 cg20300246 NA chr9:139096665-139096993 Island TRUE LHX3 Body
cg13640200 chr12 cg13640200 NA chr12:47473177-47474120 S_Shore TRUE AMIGO2 TSS1500
cg27202708 chr1 cg27202708 NA chr1:223566642-223567268 Island TRUE C1orf65 TSS200
cg20449692 chr3 cg20449692 NA chr3:170136242-170137886 Island TRUE CLDN11 1stExon
cg17530977 chr7 cg17530977 NA chr7:42276003-42277850 N_Shore TRUE GLI3 5'UTR
cg14265075 chr1 cg14265075 NA chr1:157015149-157016229 S_Shore TRUE ARHGEF11 TSS1500
cg15966757 chr12 cg15966757 NA OpenSea TRUE SLC6A13 TSS1500

cg10194829 chr8 cg10194829 NA OpenSea TRUE FGF17 TSS1500
cg12164282 chr2 cg12164282 NA chr2:1746833-1748971 Island TRUE PXDN 1stExon
cg05337441 chr2 cg05337441 rs12720761 chr2:21266669-21266961 N_Shore TRUE APOB Body
cg14934766 chr2 cg14934766 NA chr2:27008760-27009465 Island TRUE CENPA Body
cg14072120 chr22 cg14072120 NA OpenSea TRUE RAC2 TSS200
cg20387341 chr5 cg20387341 NA OpenSea TRUE FGF10 TSS1500
cg10073723 chr7 cg10073723 NA chr7:33943665-33945509 Island TRUE BMPER TSS1500
cg14036856 chr1 cg14036856 NA OpenSea TRUE C1orf210 TSS1500
cg01971122 chr11 cg01971122 NA chr11:14995128-14995908 N_Shore TRUE CALCA TSS200
cg07629017 chr8 cg07629017 NA chr8:53851701-53854426 N_Shore TRUE NPBWR1 TSS1500
cg15674432 chr6 cg15674432 rs12664506 chr6:35995245-35996372 N_Shelf TRUE SLC26A8 5'UTR
cg07651242 chr7 cg07651242 NA chr7:45613386-45615504 Island TRUE ADCY1 1stExon
cg07935264 chr2 cg07935264 NA OpenSea TRUE IL1B TSS200
cg08572611 chr7 cg08572611 NA chr7:100253782-100254150 Island TRUE ACTL6B Body
cg24056567 chr8 cg24056567 NA chr8:18871072-18872046 Island TRUE PSD3 TSS1500
cg13549845 chr4 cg13549845 NA chr4:93226348-93227007 N_Shore TRUE GRID2 Body
cg26239233 chr16 cg26239233 NA chr16:10972782-10973305 N_Shore TRUE CIITA TSS1500
cg10970251 chr11 cg10970251 NA chr11:797640-798544 N_Shore TRUE SLC25A22 TSS1500
cg12300353 chr4 cg12300353 rs149091250chr4:44449386-44451133 Island

TRUE KCTD8 1stExon
cg26578617 chr4 cg26578617 NA chr4:90758008-90758870 N_Shore TRUE
SNCA 5'UTR
cg22660578 chr17 cg22660578 NA chr17:35291899-35300875 Island TRUE
LHX1 TSS1500
cg20120491 chr22 cg20120491 NA chr22:38965733-38966196 Island TRUE
DMC1 5'UTR
cg24653181 chr15 cg24653181 NA chr15:90039464-90039984 Island TRUE
RHCG Body
cg22129364 chr1 cg22129364 NA chr1:53067880-53068608 Island TRUE
GPX7 TSS200
cg14667273 chr1 cg14667273 NA chr1:1370767-1371449 N_Shore TRUE
VWA1 TSS1500
cg21215336 chr19 cg21215336 NA chr19:7953280-7953708 Island TRUE
LRRC8E 5'UTR
cg26829131 chr12 cg26829131 rs115006165 chr12:54393374-54394648 Island
TRUE HOXC9 TSS1500
cg20372689 chr15 cg20372689 NA chr15:77223771-77224466 N_Shore
TRUE RCN2 TSS1500
cg26626089 chr19 cg26626089 rs2547362 chr19:54385315-54385604
S_Shore TRUE PRKCG 1stExon
cg10521852 chr19 cg10521852 NA chr19:19738572-19739821 Island TRUE
LPAR2 TSS1500
cg19616230 chr4 cg19616230 NA chr4:25657119-25657547 Island TRUE
SLC34A2 TSS200
cg10530281 chr12 cg10530281 NA chr12:115120774-115122945 Island TRUE
TBX3 TSS200
cg13228642 chr1 cg13228642 rs145472470 chr1:181057503-181059176
N_Shore TRUE IER5 TSS1500
cg00564163 chr7 cg00564163 NA OpenSea TRUE STEAP4 5'UTR
cg21311175 chr22 cg21311175 NA chr22:19418745-19420237 N_Shore
TRUE HIRA Body
cg22335801 chr1 cg22335801 NA chr1:1149055-1149524 Island TRUE

TNFRSF4 Body
cg25263140 chr6 cg25263140 NA chr6:106959764-106960985 N_Shore TRUE AIM1 TSS200
cg06337239 chr9 cg06337239 NA chr9:32526144-32526424 S_Shore TRUE DDX58 TSS1500
cg11984608 chr3 cg11984608 NA OpenSea TRUE CLDN16 TSS1500
cg18884137 chr17 cg18884137 NA chr17:7348274-7348830 Island TRUE CHRNB1 1stExon
cg12686915 chr8 cg12686915 NA chr8:117767930-117768139 S_Shore TRUE EIF3H TSS1500
cg11847808 chr1 cg11847808 NA chr1:22889595-22890096 Island TRUE EPHA8 TSS1500
cg13022174 chr18 cg13022174 NA chr18:23669971-23671275 Island TRUE SS18 TSS1500
cg03085312 chr17 cg03085312 NA OpenSea TRUE RARA TSS200
cg24429836 chr16 cg24429836 NA chr16:75148398-75148864 S_Shore TRUE LDHD TSS200
cg13102585 chr17 cg13102585 NA chr17:42385810-42386393 Island TRUE RUNDC3A 1stExon
cg01284306 chr21 cg01284306 NA chr21:19191095-19191952 N_Shore TRUE C21orf91 Body
cg21428681 chr8 cg21428681 NA chr8:23539807-23540602 S_Shore TRUE NKX3-1 TSS1500
cg03030757 chr6 cg03030757 NA chr6:35419879-35420766 Island TRUE FANCE Body
cg14471615 chr11 cg14471615 NA chr11:76494416-76495672 N_Shore TRUE TSKU TSS200
cg23043245 chr6 cg23043245 NA chr6:34433411-34434371 Island TRUE PACSIN1 5'UTR
cg02293044 chr22 cg02293044 NA chr22:29702245-29704723 Island TRUE GAS2L1 5'UTR
cg19194454 chr16 cg19194454 NA chr16:69760293-69760506 S_Shore TRUE

NQO1 TSS1500
cg15940569 chr15 cg15940569 rs4363842 chr15:27016892-27018943
S_Shore TRUE GABRB3 TSS1500
cg21922574 chr18 cg21922574 rs79830958 chr18:70533965-70536871 Island
TRUE NETO1 TSS1500
cg01246254 chr10 cg01246254 NA chr10:124134088-124134933 Island TRUE
PLEKHA1 5'UTR
cg23582408 chr20 cg23582408 rs190156411 chr20:62125930-62126461
S_Shelf TRUE EEF1A2 5'UTR
cg05154390 chr1 cg05154390 NA chr1:36929414-36930131 N_Shore TRUE
MRPS15 Body
cg15043057 chr1 cg15043057 NA chr1:41444870-41446116 N_Shore TRUE
CTPS TSS1500
cg00685836 chr12 cg00685836 NA chr12:82152320-82152674 S_Shore TRUE
PPFIA2 TSS200
cg10104451 chr8 cg10104451 rs28427138 chr8:143694430-143696166 Island
TRUE ARC TSS200
cg18445047 chr16 cg18445047 NA chr16:89767253-89768882 Island TRUE
SPATA2L 5'UTR
cg17178336 chr2 cg17178336 NA chr2:219924819-219926130 Island TRUE
IHH TSS200
cg19528976 chr7 cg19528976 rs181480684 chr7:100823307-100823701
N_Shore TRUE C7orf52 5'UTR
cg01998146 chr9 cg01998146 NA chr9:15510176-15511338 S_Shore TRUE
PSIP1 TSS1500
cg05389183 chr5 cg05389183 rs4602620 chr5:122371569-122372822
S_Shore TRUE PPIC TSS1500
cg01254505 chr19 cg01254505 NA OpenSea TRUE BST2 TSS200
cg12317456 chr16 cg12317456 NA chr16:55513220-55513526 N_Shore
TRUE MMP2 TSS1500
cg21053529 chr15 cg21053529 NA chr15:35046443-35047480 Island TRUE
GJD2 TSS200
cg13794888 chr15 cg13794888 NA chr15:75917646-75918212 Island TRUE

SNUPN 5'UTR
cg15835825 chr7 cg15835825 NA chr7:154861797-154862074 Island TRUE
HTR5A TSS1500
cg00792849 chr15 cg00792849 NA chr15:35046443-35047480 Island TRUE
GJD2 Body
cg09188980 chr11 cg09188980 rs5238 chr11:14993452-14993661 N_Shore
TRUE CALCA 5'UTR
cg03382304 chr21 cg03382304 NA chr21:27011624-27012398 Island TRUE
JAM2 1stExon
cg22915732 chr8 cg22915732 NA OpenSea TRUE CLVS1 TSS200
cg07525077 chr14 cg07525077 NA OpenSea TRUE RNASE3 Body
cg20312228 chr3 cg20312228 NA chr3:126113547-126113967 Island TRUE
CCDC37 TSS200
cg01555431 chr6 cg01555431 rs4568462 chr6:151561283-151562550 Island
TRUE AKAP12 Body
cg03262773 chr1 cg03262773 NA OpenSea TRUE RAD54L
TSS1500
cg01626227 chr7 cg01626227 NA chr7:99516602-99517296 Island TRUE
TRIM4 TSS200
cg14147105 chr19 cg14147105 NA chr19:17530650-17531535 Island TRUE
FAM125A Body
cg23559331 chr17 cg23559331 NA chr17:40332597-40333471 Island TRUE
KCNH4 1stExon
cg25186143 chr17 cg25186143 rs2657621 chr17:635203-636499 N_Shore
TRUE FAM57A TSS1500
cg12640109 chr11 cg12640109 NA chr11:62104779-62105636 N_Shore
TRUE ASRGL1 TSS200
cg04389838 chr3 cg04389838 NA chr3:44770936-44771137 N_Shore TRUE
ZNF501 TSS1500
cg06027949 chr8 cg06027949 NA chr8:82754054-82754476 S_Shore TRUE
SNX16 TSS1500
cg15647515 chr7 cg15647515 NA chr7:116962841-116964803 Island TRUE
WNT2 TSS1500

cg05362516 chr16 cg05362516 NA chr16:54962422-54967805 Island TRUE IRX5 TSS200
cg23889010 chr20 cg23889010 NA OpenSea TRUE SLPI Body
cg08806153 chr2 cg08806153 NA chr2:176971706-176972305 Island TRUE HOXD11 TSS200
cg04245402 chr19 cg04245402 rs117425168 OpenSea TRUE C19orf21 5'UTR
cg00512279 chr10 cg00512279 rs112647562chr10:119000435-119001530 Island TRUE SLC18A2 5'UTR
cg15494980 chr11 cg15494980 rs79429455 chr11:62598679-62600064 Island TRUE STX5 5'UTR
cg25884854 chr12 cg25884854 NA chr12:48357195-48357507 S_Shore TRUE TMEM106C Body
cg25416372 chr19 cg25416372 rs10418435 chr19:10627980-10628417 Island TRUE S1PR5 5'UTR
cg21672276 chr3 cg21672276 NA chr3:44754099-44754399 N_Shore TRUE ZNF502 TSS200
cg07379574 chr19 cg07379574 rs916791 chr19:18722358-18724001 S_Shore TRUE TMEM59L Body
cg25908985 chr2 cg25908985 NA chr2:219924819-219926130 Island TRUE IHH 1stExon
cg07212894 chr3 cg07212894 NA chr3:50242550-50243589 Island TRUE SLC38A3 5'UTR
cg22936016 chr15 cg22936016 NA chr15:40074645-40075188 Island TRUE FSIP1 TSS200
cg21092462 chr1 cg21092462 NA chr1:211306667-211307675 S_Shore TRUE KCNH1 TSS1500
cg02665570 chr1 cg02665570 NA chr1:219347109-219347572 Island TRUE LYPLAL1 Body
cg24873414 chr19 cg24873414 NA OpenSea TRUE KLHL26 TSS1500
cg26021627 chr8 cg26021627 NA chr8:53477544-53478627 S_Shore TRUE FAM150A TSS1500

cg18997990 chr15 cg18997990 NA chr15:44084144-44085272 Island TRUE SERF2 TSS1500
cg11591325 chr5 cg11591325 NA chr5:76011120-76012292 S_Shore TRUE F2R Body
cg13060154 chr9 cg13060154 NA chr9:124461797-124462190 Island TRUE DAB2IP Body
cg14958635 chr5 cg14958635 NA chr5:134870740-134872051 Island TRUE NEUROG1 1stExon
cg19996355 chr19 cg19996355 NA chr19:19729127-19729814 Island TRUE PBX4 1stExon
cg09297361 chr15 cg09297361 NA chr15:64443587-64444821 Island TRUE SNX22 Body
cg24939733 chr11 cg24939733 NA OpenSea TRUE CCDC89 TSS1500
cg04062391 chr19 cg04062391 NA chr19:9608804-9609343 Island TRUE ZNF560 5'UTR
cg04759439 chr3 cg04759439 rs141118348 chr3:56502611-56502815 Island TRUE ERC2 TSS1500
cg26728422 chr16 cg26728422 NA chr16:1429050-1430104 N_Shore TRUE UNKL 5'UTR
cg09405612 chr7 cg09405612 NA chr7:42276003-42277850 N_Shore TRUE GLI3 5'UTR
cg12089439 chr8 cg12089439 NA chr8:15397636-15398287 S_Shore TRUE TUSC3 Body
cg26952662 chr8 cg26952662 NA chr8:104383409-104384109 Island TRUE CTHRC1 TSS1500
cg18511007 chr7 cg18511007 NA OpenSea TRUE COL1A2 TSS200
cg22165175 chr1 cg22165175 NA chr1:111148983-111150186 N_Shore TRUE KCNA2 TSS1500
cg18676237 chr6 cg18676237 NA chr6:2903175-2903906 Island TRUE SERPINB9 5'UTR
cg19319069 chr7 cg19319069 NA chr7:129592032-129592891 S_Shore TRUE

UBE2H TSS1500
cg05454446 chr10 cg05454446 NA chr10:43277849-43278532 N_Shore
TRUE BMS1 TSS200
cg23473904 chr21 cg23473904 NA chr21:47517651-47518999 Island TRUE
COL6A2 TSS1500
cg12359315 chr3 cg12359315 NA chr3:45837480-45838256 N_Shore TRUE
SLC6A20 Body
cg21387302 chr9 cg21387302 NA chr9:3526015-3526945 Island TRUE
RFX3 TSS1500
cg09085198 chr18 cg09085198 rs1613739 chr18:21242366-21242805
N_Shore TRUE ANKRD29 Body
cg14070647 chr8 cg14070647 NA chr8:109094485-109095849 Island TRUE
RSPO2 5'UTR
cg22708914 chr8 cg22708914 NA chr8:19170999-19172056 N_Shore TRUE
SH2D4A TSS1500
cg08169325 chr12 cg08169325 NA chr12:131323203-131323959 S_Shore TRUE
STX2 TSS1500
cg23414387 chr1 cg23414387 NA OpenSea TRUE CYP4B1
1stExon
cg25332298 chr19 cg25332298 NA chr19:11590295-11593643 Island TRUE
ELAVL3 TSS1500
cg01366419 chr7 cg01366419 NA chr7:70596228-70598382 Island TRUE
WBSCR17 TSS1500
cg13043862 chr8 cg13043862 NA OpenSea TRUE EYA1 TSS200
cg21696393 chr16 cg21696393 rs12443534 chr16:1029878-1035327 Island
TRUE SOX8 TSS1500
cg25887294 chr6 cg25887294 NA chr6:90539454-90539954 N_Shore TRUE
CASP8AP2 TSS1500
cg15747595 chr8 cg15747595 NA chr8:98289604-98290404 Island TRUE
TSPYL5 1stExon
cg02497700 chr1 cg02497700 NA chr1:244213397-244213619 N_Shore
TRUE ZNF238 TSS1500
cg23526055 chr8 cg23526055 NA chr8:74884234-74884602 S_Shore TRUE

TCEB1 TSS1500
cg04575343 chr12 cg04575343 NA chr12:40498962-40500017 Island TRUE
SLC2A13 1stExon
cg24562819 chr20 cg24562819 NA chr20:23028403-23032218 Island TRUE
THBD 1stExon
cg09879797 chr12 cg09879797 NA chr12:118499103-118499473 S_Shore TRUE
WSB2 TSS1500
cg06055013 chr10 cg06055013 NA chr10:116852261-116854094 Island TRUE
ATRNL1 1stExon
cg17119387 chr17 cg17119387 NA chr17:2614320-2615630 Island TRUE
KIAA0664 TSS1500
cg25616762 chr5 cg25616762 NA chr5:1344545-1345469 S_Shore TRUE
CLPTM1L TSS1500
cg13576290 chr2 cg13576290 NA OpenSea TRUE FAM179A Body
cg22131172 chr13 cg22131172 NA OpenSea TRUE C13orf29
TSS1500
cg10057295 chr13 cg10057295 NA chr13:99228319-99229576 S_Shore TRUE
STK24 TSS1500
cg25942450 chr5 cg25942450 NA chr5:170735169-170739863 Island TRUE
TLX3 TSS200
cg22334665 chr6 cg22334665 NA chr6:110797297-110798201 Island TRUE
SLC22A16 Body
cg01918706 chr1 cg01918706 NA chr1:202311043-202311312 N_Shore
TRUE UBE2T 5'UTR
cg26872475 chr11 cg26872475 NA chr11:119039429-119039944 Island TRUE
NLRX1 TSS200
cg00243313 chr5 cg00243313 NA chr5:1881924-1887743 Island TRUE
IRX4 TSS1500
cg24250393 chr16 cg24250393 NA chr16:23846941-23848102 N_Shore
TRUE PRKCB TSS1500
cg13918811 chr4 cg13918811 NA chr4:25378793-25379275 N_Shore TRUE
ANAPC4 TSS1500
cg19890858 chr22 cg19890858 NA chr22:50623164-50625226 Island TRUE

TRABD 5'UTR
cg25097436 chr14 cg25097436 rs2145797 chr14:60336951-60337461 Island TRUE RTN1 Body
cg14645481 chr5 cg14645481 NA chr5:130500226-130501095 S_Shore TRUE HINT1 TSS200
cg27650434 chr1 cg27650434 NA chr1:40366595-40368213 Island TRUE MYCL1 TSS1500
cg10844844 chr6 cg10844844 NA chr6:39196817-39197517 Island TRUE KCNK5 TSS1500
cg14116122 chr12 cg14116122 NA chr12:85673878-85674700 Island TRUE ALX1 TSS200
cg07080358 chr2 cg07080358 NA chr2:68546339-68547111 Island TRUE CNRIP1 1stExon
cg06226384 chr17 cg06226384 NA OpenSea TRUE CACNG5 TSS200
cg12456510 chr21 cg12456510 NA OpenSea TRUE TFF2 TSS200
cg25123470 chr10 cg25123470 NA chr10:103603166-103603642 S_Shore TRUE KCNIP2 TSS200
cg02309273 chr1 cg02309273 NA OpenSea TRUE INPP5B TSS200
cg26450866 chr19 cg26450866 NA chr19:49575129-49576076 S_Shore TRUE KCNA7 TSS200
cg05590257 chr17 cg05590257 rs187908734 chr17:17108771-17109701 Island TRUE PLD6 1stExon
cg16158807 chr9 cg16158807 NA chr9:95946147-95947835 Island TRUE WNK2 TSS1500
cg14885742 chr11 cg14885742 rs6598045 chr11:318361-319052 S_Shore TRUE IFITM3 TSS200
cg19713196 chr1 cg19713196 NA chr1:201368560-201369032 N_Shore TRUE LAD1 Body
cg23696949 chr1 cg23696949 NA OpenSea TRUE LAMC2 Body
cg17183546 chr4 cg17183546 NA chr4:4387593-4389919 Island TRUE D4S234E TSS200
cg11628034 chr19 cg11628034 NA chr19:39926224-39926863 S_Shore TRUE

RPS16 TSS1500
cg13191049 chr15 cg13191049 NA chr15:99645030-99646444 Island TRUE
SYNM 1stExon
cg21200703 chr4 cg21200703 NA chr4:25657119-25657547 S_Shore TRUE
SLC34A2 5'UTR
cg06038133 chr17 cg06038133 NA chr17:27942532-27945388 Island TRUE
CORO6 Body
cg18988110 chr17 cg18988110 NA OpenSea TRUE PRR15L
TSS1500
cg16179125 chr20 cg16179125 NA chr20:57581902-57582595 Island TRUE
CTSZ 1stExon
cg16741710 chr1 cg16741710 NA chr1:3566445-3569636 Island TRUE
TP73 5'UTR
cg11311499 chr2 cg11311499 NA chr2:47630067-47630791 S_Shore TRUE
MSH2 Body
cg16388829 chr2 cg16388829 NA chr2:97202313-97203787 Island TRUE
ARID5A TSS200
cg22040627 chr17 cg22040627 NA chr17:6616422-6617471 Island TRUE
SLC13A5 TSS1500
cg14576824 chr1 cg14576824 NA chr1:213224611-213224867 N_Shore
TRUE RPS6KC1 TSS200
cg03469054 chr12 cg03469054 rs77737941 chr12:130387609-130389139 Island
TRUE TMEM132D1stExon
cg20855565 chr1 cg20855565 NA chr1:248020330-248021252 Island TRUE
TRIM58 TSS200
cg26704579 chr15 cg26704579 NA chr15:75018186-75019336 Island TRUE
CYP1A1 TSS1500
cg25418748 chr5 cg25418748 NA chr5:178977298-178978291 N_Shore
TRUE RUFY1 TSS1500
cg25913233 chr5 cg25913233 NA OpenSea TRUE SPARC TSS200
cg25095814 chr2 cg25095814 NA OpenSea TRUE CASP8 TSS200
cg24496666 chr1 cg24496666 rs17101180 chr1:78511577-78512161 Island
TRUE GIPC2 1stExon

cg01837719	chr6	cg01837719	NA	chr6:96463869-96464136	N_Shore	TRUE	FUT9	TSS1500
cg21426387	chr16	cg21426387	NA	chr16:11762563-11763246	Island	TRUE	SNN	5'UTR
cg07200280	chr3	cg07200280	NA	chr3:16554327-16555413	Island	TRUE	RFTN1	5'UTR
cg10983208	chr10	cg10983208	NA	chr10:73846806-73848233	S_Shore	TRUE	SPOCK2	5'UTR
cg08977371	chr16	cg08977371	NA	chr16:82660651-82661813	N_Shore	TRUE	CDH13	TSS200
cg16063112	chr10	cg16063112	rs7071852	chr10:63422538-63423105	Island	TRUE	C10orf107	5'UTR
cg06816106	chr2	cg06816106	NA	chr2:29033351-29034011	Island	TRUE	SPDYA	TSS1500
cg26509022	chr15	cg26509022	NA	chr15:101419261-101421133	Island	TRUE	ALDH1A3	TSS1500
cg14614901	chr9	cg14614901	NA	chr9:32782936-32783625	Island	TRUE	TMEM215	TSS200
cg03310469	chr2	cg03310469	NA	chr2:45235511-45237792	Island	TRUE	SIX2	TSS200
cg06668300	chr2	cg06668300	NA	chr2:95690857-95692431	Island	TRUE	MAL	Body
cg04498679	chr6	cg04498679	rs9332704		OpenSea	TRUE	C2	Body
cg03013422	chr12	cg03013422	rs113903289	chr12:125549672-125550311	S_Shore	TRUE	AACS	Body
cg05497616	chr5	cg05497616	NA	chr5:110559569-110560446	Island	TRUE	CAMK4	Body
cg13351161	chr8	cg13351161	NA	chr8:27490959-27491775	N_Shore	TRUE	SCARA3	TSS1500
cg21265783	chr7	cg21265783	NA	chr7:73703458-73704127	S_Shore	TRUE	CLIP2	5'UTR
cg02672493	chr3	cg02672493	NA	chr3:136537559-136539204	Island	TRUE	TMEM22	TSS200

cg19779211 chr11 cg19779211 NA chr11:2465171-2465648 Island TRUE KCNQ1 TSS1500
cg07693270 chr3 cg07693270 NA chr3:186856927-186857659 Island TRUE RPL39L 5'UTR
cg14213992 chr19 cg14213992 NA chr19:40502749-40503294 S_Shore TRUE ZNF546 5'UTR
cg15146752 chr1 cg15146752 NA chr1:16481484-16482782 Island TRUE EPHA2 TSS1500
cg21697134 chr17 cg21697134 rs2256316 chr17:80693067-80693729 S_Shore TRUE FN3K Body
cg02433671 chr19 cg02433671 rs77522653 chr19:11529722-11529966 Island TRUE RGL3 Body
cg13492340 chr10 cg13492340 NA chr10:15761423-15762101 S_Shore TRUE ITGA8 TSS1500
cg07684809 chr12 cg07684809 NA OpenSea TRUE GLIPR1L1 TSS1500
cg22740835 chr1 cg22740835 NA OpenSea TRUE DDR2 5'UTR
cg19536127 chr2 cg19536127 NA chr2:47402809-47404105 S_Shore TRUE CALM2 TSS1500
cg03621406 chr19 cg03621406 NA chr19:9929166-9930494 Island TRUE FBXL12 TSS1500
cg26775866 chr5 cg26775866 NA chr5:159848779-159849113 S_Shore TRUE PTTG1 5'UTR
cg25766046 chr9 cg25766046 rs148237260 chr9:94711117-94713075 Island TRUE ROR2 Body
cg25282780 chr9 cg25282780 NA chr9:4740694-4741868 Island TRUE AK3 TSS1500
cg04283938 chr22 cg04283938 NA chr22:42372483-42373299 Island TRUE 3-Sep Body
cg06688396 chr8 cg06688396 NA OpenSea TRUE TMEM55A TSS1500
cg04583874 chr8 cg04583874 NA chr8:144240761-144242113 S_Shore TRUE LY6H TSS1500

cg26446827 chr20 cg26446827 NA chr20:18268544-18269405 Island TRUE ZNF133 TSS200
cg17162024 chr8 cg17162024 NA chr8:53477544-53478627 Island TRUE FAM150A TSS1500
cg22236626 chr1 cg22236626 NA chr1:212606105-212606844 Island TRUE NENF Body
cg24304714 chr1 cg24304714 NA OpenSea TRUE LCE1C TSS1500
cg11695266 chr7 cg11695266 NA chr7:157129089-157130306 Island TRUE DNAJB6 TSS1500
cg10257049 chr5 cg10257049 NA OpenSea TRUE C5orf4 TSS200
cg19695867 chr1 cg19695867 NA chr1:109583855-109585032 S_Shore TRUE WDR47 TSS1500
cg14603345 chr20 cg14603345 NA chr20:11871374-11872207 Island TRUE BTBD3 TSS200
cg11299964 chr9 cg11299964 NA chr9:128469269-128469635 S_Shore TRUE MAPKAP1 TSS1500
cg20640433 chr6 cg20640433 NA OpenSea TRUE LAMA2 TSS200
cg08191854 chr21 cg08191854 rs9975366 OpenSea TRUE TRPM2 TSS200
cg01589580 chr18 cg01589580 NA chr18:77439094-77440398 S_Shore TRUE CTDP1 Body
cg08900043 chr12 cg08900043 NA chr12:54378696-54380102 Island TRUE HOXC10 TSS200
cg20587543 chr16 cg20587543 NA chr16:8768256-8768619 N_Shore TRUE ABAT TSS1500
cg19280968 chr3 cg19280968 NA chr3:195269553-195270363 S_Shore TRUE PPP1R2 TSS1500
cg17194182 chr7 cg17194182 NA chr7:100318106-100318684 Island TRUE EPO TSS1500
cg01683883 chr16 cg01683883 NA chr16:66612749-66613412 Island TRUE CMTM2 TSS1500
cg25947945 chr1 cg25947945 NA chr1:201368560-201369032 Island TRUE LAD1 TSS1500

cg06850526 chr17 cg06850526 NA chr17:79268367-79269467 S_Shore TRUE SLC38A10 TSS1500
cg26219051 chr22 cg26219051 NA chr22:43738882-43740285 Island TRUE SCUBE1 TSS1500
cg16378421 chr3 cg16378421 NA chr3:183735183-183736103 S_Shore TRUE ABCC5 TSS1500
cg11572744 chr5 cg11572744 rs141149651 chr5:146832560-146833938 Island TRUE DPYSL3 Body
cg16340268 chr1 cg16340268 rs138545670 chr1:226924560-226926553 Island TRUE ITPKB 5'UTR
cg27201297 chr3 cg27201297 NA chr3:37901938-37904039 Island TRUE CTDSPL TSS1500
cg15937081 chr14 cg15937081 NA chr14:45722148-45722802 N_Shore TRUE C14orf106 5'UTR
cg08896053 chr8 cg08896053 NA chr8:50822270-50822860 S_Shore TRUE SNTG1 TSS1500
cg16076328 chr2 cg16076328 rs78328381 chr2:47596629-47597256 Island TRUE EPCAM Body
cg23207990 chr4 cg23207990 rs4077965 chr4:154709512-154710827 Island TRUE SFRP2 TSS1500
cg23009046 chr11 cg23009046 NA chr11:119598382-119600157 S_Shore TRUE PVRL1 TSS1500
cg20052718 chr7 cg20052718 NA chr7:19156050-19158042 Island TRUE TWIST1 1stExon
cg13801416 chr7 cg13801416 NA chr7:134143115-134144063 Island TRUE AKR1B1 TSS200
cg13997435 chr1 cg13997435 NA OpenSea TRUE S100A2 TSS200
cg26796283 chr5 cg26796283 NA chr5:174151478-174152364 N_Shore TRUE MSX2 TSS1500
cg09009111 chr18 cg09009111 NA chr18:2846565-2848175 Island TRUE EMILIN2 1stExon
cg07172280 chr9 cg07172280 NA chr9:140008309-140009473 Island TRUE DPP7 TSS1500

cg15796978 chr2 cg15796978 rs4073367 chr2:48982621-48982958 N_Shore
TRUE LHCGR Body
cg13975625 chr4 cg13975625 rs4314240 chr4:164264821-164265772
N_Shore TRUE NPY5R TSS1500
cg21402071 chr15 cg21402071 rs71653606 chr15:78933187-78933821
S_Shore TRUE CHRNB4 TSS1500
cg18145505 chr15 cg18145505 rs1406389 chr15:33009530-33011696
N_Shore TRUE GREM1 TSS1500
cg15160742 chr1 cg15160742 NA chr1:244816199-244817080 S_Shore TRUE
PPPDE1 Body
cg10837843 chr5 cg10837843 NA chr5:172197482-172199606 Island TRUE
DUSP1 TSS1500
cg21146268 chr6 cg21146268 NA chr6:26184036-26184336 N_Shore TRUE
HIST1H2BE TSS1500
cg27637521 chr17 cg27637521 NA chr17:76354818-76357038 Island TRUE
SOCS3 5'UTR
cg23213217 chr1 cg23213217 NA chr1:224370585-224371755 N_Shore
TRUE DEGS1 TSS1500
cg17525406 chr1 cg17525406 NA chr1:4713989-4716555 Island TRUE
AJAP1 Body
cg16092786 chr11 cg16092786 NA chr11:32454874-32457311 Island TRUE
WT1 Body
cg01120308 chr11 cg01120308 NA chr11:85779725-85780397 S_Shore TRUE
PICALM TSS1500
cg07888234 chr18 cg07888234 NA chr18:59992069-59993556 Island TRUE
TNFRSF11A TSS1500
cg12343777 chr17 cg12343777 NA OpenSea TRUE ALOX15B
TSS200
cg08532057 chr13 cg08532057 rs7335827 chr13:25874994-25876200 Island
TRUE NUPL1 TSS1500
cg10146929 chr6 cg10146929 NA chr6:26020671-26021125 N_Shelf TRUE
HIST1H1A 1stExon
cg00582628 chr8 cg00582628 NA OpenSea TRUE RGS20 1stExon

cg22892110 chr8 cg22892110 rs62524242 chr8:144798487-144799038 Island TRUE MAPK15 Body

cg23300372 chr5 cg23300372 rs11575893 chr5:71014917-71015715 Island TRUE CARTPT Body

cg04504095 chr6 cg04504095 NA chr6:1604606-1615866 Island TRUE FOXC1 1stExon

cg15796941 chr12 cg15796941 NA chr12:107711603-107714107 Island TRUE BTBD11 TSS1500

cg10729531 chr20 cg10729531 NA chr20:61492119-61493626 Island TRUE TCFL5 1stExon

cg27444994 chr16 cg27444994 NA chr16:62069121-62070634 Island TRUE CDH8 TSS200

cg09470640 chr12 cg09470640 NA chr12:15475318-15475901 S_Shore TRUE PTPRO Body

cg08560942 chr7 cg08560942 rs17880615 chr7:100492217-100494941 Island TRUE ACHE 5'UTR

cg18230771 chr15 cg18230771 NA chr15:89346043-89347203 Island TRUE ACAN 5'UTR

cg07054641 chr3 cg07054641 NA chr3:113160299-113160641 Island TRUE WDR52 TSS200

cg10887021 chr5 cg10887021 NA chr5:140475599-140476607 N_Shore TRUE PCDHB2 TSS200

cg25905812 chr9 cg25905812 NA chr9:841374-843364 Island TRUE DMRT1 TSS200

cg08431931 chr22 cg08431931 NA chr22:42394589-42395255 Island TRUE WBP2NL TSS200

cg22083798 chr7 cg22083798 NA chr7:45960137-45961347 S_Shore TRUE IGFBP3 TSS1500

cg12966875 chr20 cg12966875 NA OpenSea TRUE SLPI TSS1500

cg04518808 chr19 cg04518808 NA chr19:54385315-54385604 N_Shore TRUE PRKCG TSS1500

cg16363586 chr19 cg16363586 NA OpenSea TRUE BST2 1stExon

cg17749443 chr9 cg17749443 NA chr9:115818836-115819343 Island TRUE

ZFP37 TSS200
cg02497758 chr20 cg02497758 NA chr20:39316550-39319987 Island TRUE MAFB 1stExon
cg01344452 chr17 cg01344452 NA chr17:54910496-54912470 Island TRUE DGKE 5'UTR
cg01663295 chr11 cg01663295 NA chr11:65837254-65838649 N_Shore TRUE PACS1 TSS1500
cg00240432 chr7 cg00240432 NA chr7:19156050-19158042 Island TRUE TWIST1 TSS200
cg14019317 chr1 cg14019317 NA chr1:214161197-214161415 Island TRUE PROX1 TSS1500
cg22796458 chr1 cg22796458 rs11264431 chr1:156022962-156025174 N_Shore TRUE UBQLN4 Body
cg26388152 chr17 cg26388152 NA chr17:27942532-27945388 Island TRUE CORO6 Body
cg13694867 chr21 cg13694867 NA chr21:38068193-38073891 Island TRUE SIM2 1stExon
cg01294695 chr15 cg01294695 rs76741626 chr15:90293221-90294733 Island TRUE MESP1 TSS200
cg18678185 chr19 cg18678185 NA chr19:48896890-48897199 N_Shore TRUE KDELR1 TSS1500
cg14137939 chr11 cg14137939 NA OpenSea TRUE CCDC89 1stExon
cg04425624 chr6 cg04425624 NA OpenSea TRUE TNF 1stExon
cg25742201 chr17 cg25742201 NA chr17:35305758-35306757 S_Shore TRUE AATF Body
cg17561435 chr7 cg17561435 NA chr7:33943665-33945509 Island TRUE BMPER TSS200
cg19965810 chr2 cg19965810 NA OpenSea TRUE KCNH7 TSS1500
cg08233173 chr5 cg08233173 rs6895696 chr5:172410691-172411161 Island TRUE ATP6V0E1 TSS200
cg23959705 chr1 cg23959705 NA chr1:8002408-8002699 N_Shore TRUE TNFRSF9 TSS1500

cg21988041 chr12 cg21988041 NA OpenSea TRUE GLT8D2
TSS200
cg20632573 chr11 cg20632573 NA chr11:20622720-20623399 N_Shore
TRUE SLC6A5 Body
cg24719601 chr4 cg24719601 NA chr4:41752280-41752502 N_Shore TRUE
PHOX2B 1stExon
cg03969906 chr6 cg03969906 rs9467664 chr6:26021957-26022193 N_Shore
TRUE HIST1H4A TSS200
cg00002426 chr3 cg00002426 NA chr3:57741925-57742990 S_Shore TRUE
SLMAP 1stExon
cg00265490 chr8 cg00265490 NA chr8:98656153-98657230 N_Shore TRUE
MTDH TSS1500
cg05056120 chr5 cg05056120 NA chr5:158527374-158527983 Island TRUE
EBF1 TSS1500
cg03734874 chr14 cg03734874 NA chr14:105070518-105071463 Island TRUE
TMEM179 TSS1500
cg19430897 chr6 cg19430897 NA chr6:26031880-26032264 S_Shore TRUE
HIST1H2AB TSS1500
cg24530795 chr12 cg24530795 NA chr12:111806559-111807060 S_Shore TRUE
FAM109A TSS1500
cg25234611 chr1 cg25234611 NA chr1:1370767-1371449 Island TRUE
VWA1 Body
cg24322623 chr11 cg24322623 NA chr11:17740789-17743779 N_Shore
TRUE MYOD1 TSS1500
cg02387679 chr5 cg02387679 NA chr5:75698759-75700160 Island TRUE
IQGAP2 Body
cg15379633 chr22 cg15379633 NA chr22:23487368-23487970 Island TRUE
RAB36 1stExon
cg25725843 chr2 cg25725843 NA chr2:107502353-107504216 Island TRUE
ST6GAL2 TSS1500
cg02131967 chr17 cg02131967 NA chr17:61553834-61555063 Island TRUE
ACE TSS1500
cg27601582 chr19 cg27601582 rs2304191 chr19:3933473-3933816 N_Shore

TRUE ITGB1BP3 5'UTR
cg11285843 chr3 cg11285843 NA chr3:185216310-185217131 Island TRUE
TMEM41A TSS1500
cg09548179 chr16 cg09548179 NA chr16:2569559-2571071 Island TRUE
AMDHD2 1stExon
cg02104644 chr11 cg02104644 NA chr11:61347808-61349038 Island TRUE
SYT7 Body
cg08128768 chr9 cg08128768 NA chr9:124461797-124462190 Island TRUE
DAB2IP Body
cg10708675 chr16 cg10708675 NA chr16:69760293-69760506 S_Shore TRUE
NQO1 TSS1500
cg09191327 chr9 cg09191327 NA chr9:133534534-133542394 Island TRUE
PRDM12 1stExon
cg05871607 chr1 cg05871607 NA chr1:28240584-28241535 Island TRUE
RPA2 TSS200
cg12078929 chr22 cg12078929 NA chr22:42896636-42897041 Island TRUE
SERHL Body
cg09149294 chr15 cg09149294 NA chr15:98503534-98504649 Island TRUE
ARRDC4 TSS200
cg19257550 chr9 cg19257550 NA chr9:35675648-35676375 N_Shore TRUE
CA9 TSS200
cg01599709 chr5 cg01599709 NA chr5:139725530-139726234 Island TRUE
HBEGF Body
cg20616414 chr9 cg20616414 NA chr9:95946147-95947835 Island TRUE
WNK2 1stExon
cg22026853 chr6 cg22026853 NA chr6:99279317-99283842 Island TRUE
POU3F2 1stExon
cg12513481 chr17 cg12513481 NA chr17:46507344-46507778 S_Shore TRUE
SKAP1 TSS1500
cg06797533 chr9 cg06797533 NA chr9:19127179-19127880 Island TRUE
PLIN2 TSS1500
cg21421701 chr1 cg21421701 NA chr1:212872460-212874048 Island TRUE
BATF3 Body

cg14013300 chr22 cg14013300 NA chr22:29196082-29196962 N_Shore TRUE XBP1 Body
cg13216303 chr1 cg13216303 NA chr1:153643318-153643529 N_Shore TRUE ILF2 Body
cg13079099 chr19 cg13079099 NA chr19:54974175-54976819 Island TRUE LENG9 1stExon
cg18006568 chr2 cg18006568 NA chr2:71205563-71206529 Island TRUE ANKRD53 1stExon
cg23432345 chr7 cg23432345 NA chr7:27195601-27196567 Island TRUE HOXA7 1stExon
cg07175883 chr2 cg07175883 NA chr2:176957054-176958279 Island TRUE HOXD13 1stExon
cg07314414 chr2 cg07314414 rs115599916 chr2:128784499-128786247 Island TRUE SAP130 TSS200
cg12998614 chr19 cg12998614 rs116938043 chr19:917034-920787 Island TRUE KISS1R TSS1500
cg16042149 chr22 cg16042149 NA chr22:29875919-29877242 Island TRUE NEFH TSS1500
cg03891319 chr3 cg03891319 rs150416778 chr3:52017056-52017592 N_Shore TRUE ACY1 TSS1500
cg21303011 chr3 cg21303011 NA chr3:24535844-24537436 Island TRUE THRB TSS1500
cg01803059 chr4 cg01803059 NA chr4:146402870-146403983 N_Shore TRUE SMAD1 TSS1500
cg10549973 chr14 cg10549973 NA chr14:58862541-58863209 Island TRUE TOMM20L Body
cg09238598 chr5 cg09238598 NA chr5:14872206-14872572 N_Shore TRUE ANKH TSS200
cg02248486 chr7 cg02248486 NA chr7:27182613-27185562 Island TRUE HOXA5 1stExon
cg14696396 chr15 cg14696396 NA chr15:83775861-83776922 Island TRUE TM6SF1 1stExon
cg12091331 chr8 cg12091331 NA OpenSea TRUE PLAT TSS200

cg03169527 chr3 cg03169527 NA chr3:11887799-11888538 S_Shore TRUE C3orf31 TSS1500
cg16041611 chr6 cg16041611 NA chr6:43138711-43140292 Island TRUE SRF 1stExon
cg00343092 chr22 cg00343092 rs9333315 chr22:43547319-43548169 Island TRUE TSPO 5'UTR
cg01401376 chr6 cg01401376 NA chr6:133562086-133563586 Island TRUE EYA4 5'UTR
cg01988129 chr8 cg01988129 NA chr8:67344497-67344989 Island TRUE ADHFE1 Body
cg06385087 chr20 cg06385087 NA chr20:57581902-57582595 Island TRUE CTSZ Body
cg03405173 chr19 cg03405173 NA chr19:11877720-11878280 Island TRUE ZNF441 TSS200
cg06268694 chr22 cg06268694 NA chr22:46929317-46934861 Island TRUE CELSR1 1stExon
cg12109455 chr10 cg12109455 NA chr10:133998912-134001500 Island TRUE DPYSL4 Body
cg05832051 chr19 cg05832051 NA chr19:54369387-54369809 Island TRUE MYADM TSS200
cg01126560 chr9 cg01126560 NA chr9:139886312-139887180 N_Shore TRUE C9orf142 TSS1500
cg12532500 chr13 cg12532500 NA OpenSea TRUE STARD13 1stExon
cg24801210 chr3 cg24801210 NA chr3:101292885-101293612 Island TRUE PCNP Body
cg05820087 chr3 cg05820087 NA OpenSea TRUE ATP13A4 1stExon
cg24517609 chr3 cg24517609 NA chr3:156272636-156273099 Island TRUE SSR3 1stExon
cg12045002 chr2 cg12045002 rs72850570 chr2:27070302-27072922 N_Shore TRUE DPYSL5 TSS1500
cg24794433 chr9 cg24794433 NA chr9:124461797-124462190 Island TRUE

DAB2IP Body
cg27092035 chr5 cg27092035 rs114678394 chr5:175792355-175793505 Island TRUE ARL10 Body
cg09088508 chr12 cg09088508 NA chr12:122230903-122232359 Island TRUE RHOF 1stExon
cg26091679 chr2 cg26091679 NA chr2:74734322-74735273 Island TRUE PCGF1 TSS1500
cg27096144 chr5 cg27096144 NA chr5:174151478-174152364 Island TRUE MSX2 1stExon
cg22674717 chr16 cg22674717 NA chr16:51183699-51188763 Island TRUE SALL1 TSS1500
cg01557989 chr20 cg01557989 NA chr20:19192459-19193902 Island TRUE SLC24A3 TSS1500
cg11673092 chr10 cg11673092 NA OpenSea TRUE ARMC3 TSS1500
cg18689253 chr9 cg18689253 NA chr9:82185479-82188560 Island TRUE TLE4 TSS200
cg12696750 chr1 cg12696750 rs2273014 chr1:38022452-38022719 Island TRUE DNALI1 TSS200
cg18292394 chr10 cg18292394 NA OpenSea TRUE MAT1A TSS200
cg10409680 chr6 cg10409680 rs7383157 chr6:31238852-31240120 Island TRUE HLA-C Body
cg04265576 chr7 cg04265576 NA chr7:27186927-27187692 Island TRUE HOXA6 TSS200
cg13801381 chr12 cg13801381 NA chr12:4383193-4384405 Island TRUE CCND2 Body
cg18197795 chr19 cg18197795 NA chr19:13067585-13068152 N_Shore TRUE GADD45GIP1 Body
cg17371081 chr11 cg17371081 NA chr11:20690579-20691845 Island TRUE NELL1 TSS200
cg16474684 chr4 cg16474684 NA chr4:2470219-2471728 N_Shore TRUE RNF4 TSS1500
cg07935568 chr13 cg07935568 NA chr13:49794125-49795542 Island TRUE MLNR TSS1500

cg08687825 chr6 cg08687825 NA OpenSea TRUE PRSS35 TSS1500
cg23596123 chr5 cg23596123 NA chr5:140531157-140532017 N_Shore TRUE PCDHB6 1stExon
cg18244915 chr19 cg18244915 NA chr19:37959852-37960615 N_Shore TRUE ZNF569 TSS200
cg10523019 chr2 cg10523019 NA chr2:227700313-227701115 Island TRUE RHBDD1 TSS1500
cg04315264 chr6 cg04315264 rs138610837 chr6:35435810-35437084 Island TRUE RPL10A Body
cg03409548 chr1 cg03409548 NA chr1:47082016-47082526 S_Shore TRUE MOBKL2C TSS200
cg08398233 chr1 cg08398233 NA chr1:20810462-20813511 Island TRUE CAMK2N1 Body
cg19904653 chr8 cg19904653 NA chr8:37707149-37707507 S_Shore TRUE BRF2 TSS1500
cg20647888 chr10 cg20647888 NA chr10:13043094-13043781 S_Shore TRUE CCDC3 TSS1500
cg10723020 chr2 cg10723020 NA chr2:208393955-208395024 N_Shore TRUE CREB1 TSS1500
cg16703647 chr15 cg16703647 NA chr15:78632669-78633108 N_Shore TRUE CRABP1 TSS200
cg19615059 chr12 cg19615059 rs4635144 chr12:110271000-110271582 N_Shore TRUE TRPV4 5'UTR
cg10217449 chr4 cg10217449 rs1110368 chr4:44728033-44728719 N_Shore TRUE GNPDA2 5'UTR
cg19428336 chr4 cg19428336 NA chr4:187644319-187648253 Island TRUE FAT1 TSS1500
cg25823578 chr14 cg25823578 rs1353410 chr14:52780687-52781969 Island TRUE PTGER2 1stExon
cg08996521 chr3 cg08996521 rs57987466 chr3:50648939-50649829 S_Shore TRUE CISH TSS1500
cg03506799 chr15 cg03506799 NA chr15:101791151-101792714 S_Shore TRUE CHSY1 TSS1500

cg21516384 chr17 cg21516384 NA chr17:78233888-78235283 N_Shore TRUE RNF213 TSS1500
cg11846236 chr3 cg11846236 NA chr3:48631882-48632901 Island TRUE COL7A1 1stExon
cg20047732 chr3 cg20047732 NA chr3:180319823-180320393 Island TRUE TTC14 TSS200
cg01513661 chr22 cg01513661 NA chr22:50781190-50782009 Island TRUE SAPS2 5'UTR
cg05654164 chr1 cg05654164 rs74883561 chr1:85724701-85725524 S_Shore TRUE C1orf52 TSS1500
cg03879902 chr19 cg03879902 NA chr19:46174372-46174575 N_Shelf TRUE GIPR 5'UTR
cg17491456 chr5 cg17491456 NA chr5:39424892-39425320 N_Shore TRUE DAB2 5'UTR
cg24884084 chr1 cg24884084 NA OpenSea TRUE SPRR1B TSS1500
cg18342279 chr4 cg18342279 NA chr4:48492117-48493589 Island TRUE ZAR1 1stExon
cg05705583 chr5 cg05705583 NA chr5:134074083-134074494 N_Shore TRUE CAMLG TSS1500
cg11467738 chr7 cg11467738 NA chr7:98971649-98973170 Island TRUE ARPC1B 5'UTR
cg21178548 chr17 cg21178548 NA chr17:48206663-48207601 Island TRUE SAMD14 TSS200
cg09929612 chr7 cg09929612 rs116894034 chr7:99063423-99063624 S_Shore TRUE ATP5J2 Body
cg09553358 chr11 cg09553358 rs142363186 chr11:791953-792400 N_Shore TRUE CEND1 TSS200
cg05899618 chr2 cg05899618 rs1898129 chr2:20865289-20867589 Island TRUE GDF7 TSS1500
cg21599792 chr11 cg21599792 NA chr11:86667041-86667289 N_Shore TRUE FZD4 TSS1500
cg08949296 chr8 cg08949296 NA chr8:75232649-75234101 Island TRUE

JPH1 1stExon

cg15839448 chr8 cg15839448 NA chr8:41165852-41167140 Island TRUE SFRP1 1stExon

cg16638540 chr19 cg16638540 NA chr19:58570393-58571779 Island TRUE ZNF135 TSS200

cg08260959 chr6 cg08260959 NA chr6:26240697-26240951 Island TRUE HIST1H4F 1stExon

cg11724134 chr6 cg11724134 NA chr6:30042918-30043500 S_Shore TRUE RNF39 TSS200

cg22224704 chr11 cg22224704 NA chr11:67350928-67351953 S_Shore TRUE GSTP1 Body

cg26538116 chr2 cg26538116 NA chr2:44222741-44223732 Island TRUE LRPPRC TSS200

cg25432997 chr17 cg25432997 NA chr17:7137358-7138133 Island TRUE DVL2 1stExon

Supplementary table 5. 742 genes were Hypermethylated, and 921 genes were Hypomethylated

Genes	Type
A2M	Hypermethylation
AACS	Hypermethylation
AATF	Hypermethylation
AATK	Hypermethylation
ABCB6	Hypermethylation
ABHD12	Hypermethylation
ABHD2	Hypermethylation
ACAT1	Hypermethylation
ACOX2	Hypermethylation

ACSM3	Hypermethylation
ACY1	Hypermethylation
ADCY3	Hypermethylation
ADHFE1	Hypermethylation
AIFM3	Hypermethylation
AIPL1	Hypermethylation
AIRE	Hypermethylation
AK3	Hypermethylation
AKAP12	Hypermethylation
ALDH18A1	Hypermethylation
ALDH3A1	Hypermethylation
ALDH3B1	Hypermethylation
ALDOA	Hypermethylation
ALG1	Hypermethylation
AMDHD2	Hypermethylation
AMIGO2	Hypermethylation
AMOTL2	Hypermethylation
AMPD3	Hypermethylation
ANKH	Hypermethylation
ANKRD11	Hypermethylation
ANKRD53	Hypermethylation
ANP32A	Hypermethylation
ANXA9	Hypermethylation
AOC2	Hypermethylation
API5	Hypermethylation
APOB	Hypermethylation
APOL6	Hypermethylation
AQP11	Hypermethylation
AQP9	Hypermethylation
ARAP3	Hypermethylation
ARC	Hypermethylation
ARHGAP12	Hypermethylation
ARHGDIA	Hypermethylation

ARHGDIB Hypermethylation
ARHGEF2 Hypermethylation
ARPC1B Hypermethylation
ASCC3 Hypermethylation
ASPHD1 Hypermethylation
ASRGL1 Hypermethylation
ASS1 Hypermethylation
ATF7IP2 Hypermethylation
ATP10A Hypermethylation
ATP13A4 Hypermethylation
ATP2C1 Hypermethylation
ATP5D Hypermethylation
ATP5J2 Hypermethylation
ATP6V0E1 Hypermethylation
ATP6V1B1 Hypermethylation
B4GALT4 Hypermethylation
BAT2 Hypermethylation
BBOX1 Hypermethylation
BCL2L2 Hypermethylation
BCL9L Hypermethylation
BCMO1 Hypermethylation
BLNK Hypermethylation
BOD1 Hypermethylation
BRAF Hypermethylation
BRF2 Hypermethylation
BRUNOL5 Hypermethylation
BST2 Hypermethylation
BTBD3 Hypermethylation
BTBD8 Hypermethylation
BTN3A2 Hypermethylation
BTN3A3 Hypermethylation
C10orf81 Hypermethylation
C11orf52 Hypermethylation

C14orf106 Hypermethylation
C14orf143 Hypermethylation
C15orf29 Hypermethylation
C15orf52 Hypermethylation
C16orf87 Hypermethylation
C19orf21 Hypermethylation
C1orf116 Hypermethylation
C1orf210 Hypermethylation
C1orf52 Hypermethylation
C1QC Hypermethylation
C1QTNF6 Hypermethylation
C1R Hypermethylation
C1SHypermethylation
C2 Hypermethylation
C20orf151 Hypermethylation
C21orf63 Hypermethylation
C21orf91 Hypermethylation
C2orf43 Hypermethylation
C2orf50 Hypermethylation
C3 Hypermethylation
C3orf31 Hypermethylation
C3orf37 Hypermethylation
C5orf4 Hypermethylation
C6orf227 Hypermethylation
C7orf34 Hypermethylation
C7orf55 Hypermethylation
C8orf44 Hypermethylation
C9orf100 Hypermethylation
C9orf142 Hypermethylation
C9orf40 Hypermethylation
C9orf64 Hypermethylation
CA9 Hypermethylation
CACHD1 Hypermethylation

CACNG5 Hypermethylation
CADPS Hypermethylation
CALM2 Hypermethylation
CAMK2N1 Hypermethylation
CAMLG Hypermethylation
CAPS Hypermethylation
CARD10 Hypermethylation
CARD6 Hypermethylation
CASP10 Hypermethylation
CASP8 Hypermethylation
CASP8AP2 Hypermethylation
CASZ1 Hypermethylation
CBLC Hypermethylation
CBLN4 Hypermethylation
CCDC117 Hypermethylation
CCDC37 Hypermethylation
CCDC89 Hypermethylation
CCNB1IP1 Hypermethylation
CCR1 Hypermethylation
CD59 Hypermethylation
CD86 Hypermethylation
CDH1 Hypermethylation
CDH5 Hypermethylation
CDKL1 Hypermethylation
CEL Hypermethylation
CELSR1 Hypermethylation
CENPB Hypermethylation
CFB Hypermethylation
CFH Hypermethylation
CGGBP1 Hypermethylation
CHEK1 Hypermethylation
CHI3L1 Hypermethylation
CHI3L2 Hypermethylation

CHRM1 Hypermethylation
CHST4 Hypermethylation
CIB3 Hypermethylation
CIC Hypermethylation
CIITA Hypermethylation
CITED4 Hypermethylation
CLDN11 Hypermethylation
CLDN16 Hypermethylation
CLDN4 Hypermethylation
CLEC2B Hypermethylation
CLEC3B Hypermethylation
CLIC3 Hypermethylation
CMBL Hypermethylation
CNNM4 Hypermethylation
CNOT8 Hypermethylation
CNTD2 Hypermethylation
CORO6 Hypermethylation
CP Hypermethylation
CPEB4 Hypermethylation
CPNE8 Hypermethylation
CRBN Hypermethylation
CRLF1 Hypermethylation
CRMP1 Hypermethylation
CRYBB3 Hypermethylation
CSNK1D Hypermethylation
CSNK1E Hypermethylation
CTDSPL Hypermethylation
CTNND1 Hypermethylation
CTSZ Hypermethylation
CUEDC1 Hypermethylation
CYB5R3 Hypermethylation
CYP1A1 Hypermethylation
CYP4B1 Hypermethylation

CYP4F11 Hypermethylation
CYP4F3 Hypermethylation
DAB2 Hypermethylation
DAB2IP Hypermethylation
DAPP1 Hypermethylation
DDR2 Hypermethylation
DDX23 Hypermethylation
DDX27 Hypermethylation
DDX58 Hypermethylation
DEGS1 Hypermethylation
DHCR24 Hypermethylation
DLK1 Hypermethylation
DMBT1 Hypermethylation
DMC1 Hypermethylation
DNAJC14 Hypermethylation
DNALI1 Hypermethylation
DOK5 Hypermethylation
DOPEY2 Hypermethylation
DPYSL3 Hypermethylation
DPYSL4 Hypermethylation
DVL2 Hypermethylation
DYNC1LI1 Hypermethylation
EDN2 Hypermethylation
EFEMP2 Hypermethylation
EHD2 Hypermethylation
EHF Hypermethylation
EIF3H Hypermethylation
ELMO3 Hypermethylation
ENPEP Hypermethylation
EOMES Hypermethylation
EPCAM Hypermethylation
EPHA1 Hypermethylation
EPHA2 Hypermethylation

ERC2	Hypermethylation
ESRP1	Hypermethylation
ESRP2	Hypermethylation
F2RL3	Hypermethylation
FAM179A	Hypermethylation
FAM180A	Hypermethylation
FAM83A	Hypermethylation
FAM84B	Hypermethylation
FAT1	Hypermethylation
FBXL12	Hypermethylation
FBXO16	Hypermethylation
FBXO38	Hypermethylation
FBXO6	Hypermethylation
FCGBP	Hypermethylation
FER	Hypermethylation
FERD3L	Hypermethylation
FEZF2	Hypermethylation
FGF12	Hypermethylation
FGF17	Hypermethylation
FGF8	Hypermethylation
FGFR4	Hypermethylation
FLII	Hypermethylation
FLJ20184	Hypermethylation
FLNC	Hypermethylation
FN3K	Hypermethylation
FOXP4	Hypermethylation
FOXS1	Hypermethylation
FRK	Hypermethylation
FSD1	Hypermethylation
FSIP1	Hypermethylation
FUT2	Hypermethylation
FUT3	Hypermethylation
FUT8	Hypermethylation

FXYD3 Hypermethylation
FZD4 Hypermethylation
GADD45GIP1 Hypermethylation
GALK1 Hypermethylation
GALNT7 Hypermethylation
GALT Hypermethylation
GAS2L1 Hypermethylation
GAS2L2 Hypermethylation
GBP4 Hypermethylation
GBP6 Hypermethylation
GCNT3 Hypermethylation
GDF15 Hypermethylation
GGT5 Hypermethylation
GHSR Hypermethylation
GLIPR1L1 Hypermethylation
GLIPR1L2 Hypermethylation
GNAI2 Hypermethylation
GNG12 Hypermethylation
GNPDA2 Hypermethylation
GPR132 Hypermethylation
GPR152 Hypermethylation
GPR156 Hypermethylation
GPR39 Hypermethylation
GPR56 Hypermethylation
GRB7 Hypermethylation
GREB1 Hypermethylation
GRHL3 Hypermethylation
GRIK2 Hypermethylation
GRM7 Hypermethylation
GSTP1 Hypermethylation
GYPC Hypermethylation
H1F0 Hypermethylation
HAND2 Hypermethylation

HBEGF Hypermethylation
HDAC10 Hypermethylation
HEBP2 Hypermethylation
HINT1 Hypermethylation
HIRA Hypermethylation
HIST1H1A Hypermethylation
HIST1H2AB Hypermethylation
HIST1H2BEHypermethylation
HIST1H4A Hypermethylation
HIST1H4F Hypermethylation
HIST2H2AB Hypermethylation
HKDC1 Hypermethylation
HLA-C Hypermethylation
HLA-DMA Hypermethylation
HLA-DOB Hypermethylation
HNF1B Hypermethylation
HOXA5 Hypermethylation
HOXB2 Hypermethylation
HOXD4 Hypermethylation
HP Hypermethylation
HPD Hypermethylation
HPX Hypermethylation
HSD3B1 Hypermethylation
HSD3B7 Hypermethylation
HSPB2 Hypermethylation
HTR1B Hypermethylation
HYAL2 Hypermethylation
HYDIN Hypermethylation
HYLS1 Hypermethylation
IDO1 Hypermethylation
IFI35 Hypermethylation
IFITM1 Hypermethylation
IFITM2 Hypermethylation

IFITM3 Hypermethylation
IGDCC4 Hypermethylation
IKBKE Hypermethylation
IL1B Hypermethylation
IL1RL2 Hypermethylation
IL1RN Hypermethylation
IL5RA Hypermethylation
ILF2 Hypermethylation
INSR Hypermethylation
IQGAP1 Hypermethylation
IQUB Hypermethylation
IRF1 Hypermethylation
ISG20L2 Hypermethylation
ITGA8 Hypermethylation
ITGB6 Hypermethylation
ITPKB Hypermethylation
KALRN Hypermethylation
KAT2B Hypermethylation
KCNIP2 Hypermethylation
KCNJ16 Hypermethylation
KCNK5 Hypermethylation
KCTD14 Hypermethylation
KDELR1 Hypermethylation
KIAA0664 Hypermethylation
KIF1A Hypermethylation
KISS1R Hypermethylation
KLHL26 Hypermethylation
KLHL6 Hypermethylation
KLK10 Hypermethylation
KRT23 Hypermethylation
KRT7 Hypermethylation
KRT8 Hypermethylation
KRTAP13-3 Hypermethylation

KRTCAP3	Hypermethylation
LAD1	Hypermethylation
LAMA4	Hypermethylation
LAMC2	Hypermethylation
LARP1	Hypermethylation
LCE1C	Hypermethylation
LDHD	Hypermethylation
LDLR	Hypermethylation
LENG9	Hypermethylation
LETM1	Hypermethylation
LGALS12	Hypermethylation
LGALS3BP	Hypermethylation
LGALS8	Hypermethylation
LIME1	Hypermethylation
LMCD1	Hypermethylation
LMNB2	Hypermethylation
LOC284837	Hypermethylation
LOC400931	Hypermethylation
LOC84931	Hypermethylation
LONP2	Hypermethylation
LOXL1	Hypermethylation
LPAR2	Hypermethylation
LRDD	Hypermethylation
LRG1	Hypermethylation
LRP6	Hypermethylation
LRPPRC	Hypermethylation
LRRC15	Hypermethylation
LRRC8E	Hypermethylation
LTBP1	Hypermethylation
LTBR	Hypermethylation
LTC4S	Hypermethylation
LY6H	Hypermethylation
LY75	Hypermethylation

LYPLAL1	Hypermethylation
MAN2C1	Hypermethylation
MAP3K14	Hypermethylation
MAPK13	Hypermethylation
MAPK14	Hypermethylation
MAPKAP1	Hypermethylation
MAT1A	Hypermethylation
MAT2B	Hypermethylation
MCCC1	Hypermethylation
MCF2L	Hypermethylation
MCM9	Hypermethylation
ME2	Hypermethylation
MED27	Hypermethylation
MEG3	Hypermethylation
MEST	Hypermethylation
MGEA5	Hypermethylation
MGP	Hypermethylation
MKNK1	Hypermethylation
MLLT6	Hypermethylation
MLPH	Hypermethylation
MMP10	Hypermethylation
MMRN1	Hypermethylation
MOBKL2C	Hypermethylation
MORF4	Hypermethylation
MPRIP	Hypermethylation
MPZL2	Hypermethylation
MPZL3	Hypermethylation
MRPL20	Hypermethylation
MRPL22	Hypermethylation
MRPL48	Hypermethylation
MRPS15	Hypermethylation
MRPS2	Hypermethylation
MS4A2	Hypermethylation

MSH2 Hypermethylation
MSLN Hypermethylation
MSX2 Hypermethylation
MTDH Hypermethylation
MTL5 Hypermethylation
MYBPH Hypermethylation
MYD88 Hypermethylation
MYH6 Hypermethylation
MYO1F Hypermethylation
NACC2 Hypermethylation
NCALD Hypermethylation
NCOA6 Hypermethylation
NDRG1 Hypermethylation
NDRG3 Hypermethylation
NDUFB8 Hypermethylation
NDUFS4 Hypermethylation
NDUFS7 Hypermethylation
NENF Hypermethylation
NETO1 Hypermethylation
NFATC3 Hypermethylation
NKTR Hypermethylation
NLRX1 Hypermethylation
NOD2 Hypermethylation
NOP10 Hypermethylation
NPAS2 Hypermethylation
NPM1 Hypermethylation
NPR2 Hypermethylation
NQO1 Hypermethylation
NRG4 Hypermethylation
NUDT14 Hypermethylation
NUDT15 Hypermethylation
NUDT8 Hypermethylation
NUPR1 Hypermethylation

OAS1	Hypermethylation
OAS2	Hypermethylation
OR1A2	Hypermethylation
OVGP1	Hypermethylation
OXGR1	Hypermethylation
OXTR	Hypermethylation
PACS1	Hypermethylation
PAM	Hypermethylation
PANX1	Hypermethylation
PAQR7	Hypermethylation
PARL	Hypermethylation
PARP6	Hypermethylation
PAX4	Hypermethylation
PCDH1	Hypermethylation
PCDHB1	Hypermethylation
PCDHB10	Hypermethylation
PCDHB15	Hypermethylation
PCDHB2	Hypermethylation
PCDHB6	Hypermethylation
PCDHB7	Hypermethylation
PCGF1	Hypermethylation
PCNP	Hypermethylation
PCSK5	Hypermethylation
PDCD5	Hypermethylation
PDZRN3	Hypermethylation
PEX10	Hypermethylation
PEX6	Hypermethylation
PFN1	Hypermethylation
PGLYRP2	Hypermethylation
PHLDA2	Hypermethylation
PHOX2B	Hypermethylation
PI15	Hypermethylation
PICALM	Hypermethylation

PKP3 Hypermethylation
PLA2G4E Hypermethylation
PLAC8 Hypermethylation
PLAT Hypermethylation
PLCB1 Hypermethylation
PLEC1 Hypermethylation
PLEKHA1 Hypermethylation
PLEKHA4 Hypermethylation
PLEKHG2 Hypermethylation
PLIN2 Hypermethylation
PLSCR4 Hypermethylation
PLXNB1 Hypermethylation
PNMT Hypermethylation
PPARGC1A Hypermethylation
PPIC Hypermethylation
PPIL1 Hypermethylation
PPP2CB Hypermethylation
PRIMA1 Hypermethylation
PROCR Hypermethylation
PRR15L Hypermethylation
PRRG4 Hypermethylation
PSIP1 Hypermethylation
PSKH1 Hypermethylation
PTGES Hypermethylation
PTGFRN Hypermethylation
PTH2 Hypermethylation
PTPN1 Hypermethylation
PTPN18 Hypermethylation
PTPN6 Hypermethylation
PTPRE Hypermethylation
PTTG1 Hypermethylation
PVRL1 Hypermethylation
PVRL4 Hypermethylation

PXDN Hypermethylation
PYCARD Hypermethylation
RAB25 Hypermethylation
RAB26 Hypermethylation
RAB36 Hypermethylation
RAB3D Hypermethylation
RAC2 Hypermethylation
RAD52 Hypermethylation
RAD54L Hypermethylation
RALY Hypermethylation
RARA Hypermethylation
RCN2 Hypermethylation
RELA Hypermethylation
RERG Hypermethylation
RFTN1 Hypermethylation
RFX3 Hypermethylation
RGL3 Hypermethylation
RGS13 Hypermethylation
RGS14 Hypermethylation
RGS20 Hypermethylation
RHOC Hypermethylation
RHOF Hypermethylation
RILP Hypermethylation
RIPK4 Hypermethylation
RNASE3 Hypermethylation
RNF39 Hypermethylation
RPA2 Hypermethylation
RPAP3 Hypermethylation
RPL10A Hypermethylation
RPS16 Hypermethylation
RUFY1 Hypermethylation
RYBP Hypermethylation
S100A16 Hypermethylation

S100A2 Hypermethylation
S100A4 Hypermethylation
S100A8 Hypermethylation
SACM1L Hypermethylation
SALL1 Hypermethylation
SAMD14 Hypermethylation
SAP130 Hypermethylation
SAPS2 Hypermethylation
SCARA3 Hypermethylation
SCGB1D1 Hypermethylation
SCGB1D2 Hypermethylation
SCRN2 Hypermethylation
SDCBP2 Hypermethylation
SEC22A Hypermethylation
SECISBP2 Hypermethylation
SELENBP1 Hypermethylation
SELPLG Hypermethylation
SEMA6B Hypermethylation
SENP5 Hypermethylation
SEPN1 Hypermethylation
SERF2 Hypermethylation
SERHL Hypermethylation
SERPINA1 Hypermethylation
SERPINB1 Hypermethylation
SERPINB7 Hypermethylation
SERPINB9 Hypermethylation
SF3A3 Hypermethylation
SFN Hypermethylation
SGCA Hypermethylation
SGPP2 Hypermethylation
SGTA Hypermethylation
SH2D4A Hypermethylation
SH3BGR Hypermethylation

SH3RF2 Hypermethylation
SHANK1 Hypermethylation
SHC4 Hypermethylation
SIX2 Hypermethylation
SLC12A4 Hypermethylation
SLC15A2 Hypermethylation
SLC15A3 Hypermethylation
SLC16A5 Hypermethylation
SLC22A5 Hypermethylation
SLC25A22 Hypermethylation
SLC26A8 Hypermethylation
SLC30A8 Hypermethylation
SLC34A2 Hypermethylation
SLC35D2 Hypermethylation
SLC38A10 Hypermethylation
SLC44A2 Hypermethylation
SLC44A4 Hypermethylation
SLC6A13 Hypermethylation
SLC6A20 Hypermethylation
SLC7A11 Hypermethylation
SLMAP Hypermethylation
SLPI Hypermethylation
SMAD1 Hypermethylation
SMC5 Hypermethylation
SMPDL3B Hypermethylation
SNAP23 Hypermethylation
SNORD116-1 Hypermethylation
SNRPN Hypermethylation
SNUPN Hypermethylation
SNX16 Hypermethylation
SNX22 Hypermethylation
SOCS3 Hypermethylation
SORT1 Hypermethylation

SOST	Hypermethylation
SOX1	Hypermethylation
SP100	Hypermethylation
SPAG6	Hypermethylation
SPATA18	Hypermethylation
SPATA2L	Hypermethylation
SPDEF	Hypermethylation
SPDYA	Hypermethylation
SPINT1	Hypermethylation
SPOCD1	Hypermethylation
SPRR1B	Hypermethylation
SPRY4	Hypermethylation
SQRDL	Hypermethylation
SRBD1	Hypermethylation
SRF	Hypermethylation
SS18	Hypermethylation
SSH3	Hypermethylation
SSR3	Hypermethylation
SSTR3	Hypermethylation
STARD13	Hypermethylation
STC1	Hypermethylation
STK24	Hypermethylation
STX5	Hypermethylation
SUB1	Hypermethylation
SUSD2	Hypermethylation
SUSD3	Hypermethylation
SYT8	Hypermethylation
TACSTD2	Hypermethylation
TAS2R38	Hypermethylation
TBC1D10B	Hypermethylation
TBX4	Hypermethylation
TCEB1	Hypermethylation
TCIRG1	Hypermethylation

TEPP	Hypermethylation
TERC	Hypermethylation
TEX15	Hypermethylation
TFAP2C	Hypermethylation
TFF2	Hypermethylation
TGM5	Hypermethylation
THBS1	Hypermethylation
THG1L	Hypermethylation
THOC1	Hypermethylation
THOC7	Hypermethylation
THOP1	Hypermethylation
THRB	Hypermethylation
TIPIN	Hypermethylation
TM4SF1	Hypermethylation
TMC4	Hypermethylation
TMEM106C	Hypermethylation
TMEM107	Hypermethylation
TMEM139	Hypermethylation
TMEM165	Hypermethylation
TMEM173	Hypermethylation
TMEM41A	Hypermethylation
TMEM55A	Hypermethylation
TMEM87A	Hypermethylation
TMEM92	Hypermethylation
TMIGD2	Hypermethylation
TMOD3	Hypermethylation
TMPRSS3	Hypermethylation
TNF	Hypermethylation
TNFAIP2	Hypermethylation
TNKS1BP1	Hypermethylation
TOMM20L	Hypermethylation
TOP1MT	Hypermethylation
TPD52	Hypermethylation

TRABD	Hypermethylation
TRAPPC10	Hypermethylation
TRDMT1	Hypermethylation
TRIB2	Hypermethylation
TRIM22	Hypermethylation
TRIM4	Hypermethylation
TRIM59	Hypermethylation
TRPM2	Hypermethylation
TRPV6	Hypermethylation
TSPAN13	Hypermethylation
TSPO	Hypermethylation
TSTA3	Hypermethylation
TTC14	Hypermethylation
TTC22	Hypermethylation
TTL	Hypermethylation
TUSC3	Hypermethylation
UBA7	Hypermethylation
UBB	Hypermethylation
UBE2H	Hypermethylation
UBL5	Hypermethylation
UBQLN4	Hypermethylation
UCK1	Hypermethylation
UHRF1	Hypermethylation
UNKL	Hypermethylation
UPK1B	Hypermethylation
USP18	Hypermethylation
VAMP4	Hypermethylation
VAV1	Hypermethylation
VNN2	Hypermethylation
VPREB1	Hypermethylation
VTCN1	Hypermethylation
VWA1	Hypermethylation
WBP2NL	Hypermethylation

WBSCR17 Hypermethylation
WBSCR27 Hypermethylation
WDR47 Hypermethylation
WDR52 Hypermethylation
WIT1 Hypermethylation
WNK2 Hypermethylation
WSB2 Hypermethylation
WT1 Hypermethylation
XDH Hypermethylation
YES1 Hypermethylation
YWHAE Hypermethylation
ZAR1 Hypermethylation
ZCCHC6 Hypermethylation
ZFYVE16 Hypermethylation
ZFYVE9 Hypermethylation
ZMAT2 Hypermethylation
ZMPSTE24 Hypermethylation
ZNF132 Hypermethylation
ZNF133 Hypermethylation
ZNF135 Hypermethylation
ZNF148 Hypermethylation
ZNF16 Hypermethylation
ZNF236 Hypermethylation
ZNF300 Hypermethylation
ZNF350 Hypermethylation
ZNF428 Hypermethylation
ZNF441 Hypermethylation
ZNF445 Hypermethylation
ZNF471 Hypermethylation
ZNF501 Hypermethylation
ZNF502 Hypermethylation
ZNF544 Hypermethylation
ZNF556 Hypermethylation

ZNF560 Hypermethylation
ZNF561 Hypermethylation
ZNF566 Hypermethylation
ZNF57 Hypermethylation
ZNF579 Hypermethylation
ZNF625 Hypermethylation
ZNF76 Hypermethylation
ABAT Hypomethylation
ABCC4 Hypomethylation
ABCC5 Hypomethylation
ABCF3 Hypomethylation
ABHD8 Hypomethylation
ABLIM3 Hypomethylation
ACAN Hypomethylation
ACAP2 Hypomethylation
ACCN2 Hypomethylation
ACE Hypomethylation
ACHE Hypomethylation
ACRBP Hypomethylation
ACSBG2 Hypomethylation
ACSF2 Hypomethylation
ACSS3 Hypomethylation
ACTC1 Hypomethylation
ACTL6B Hypomethylation
ACTN2 Hypomethylation
ACTRT2 Hypomethylation
ACVRL1 Hypomethylation
ADAM12 Hypomethylation
ADAMTS19 Hypomethylation
ADAMTS8 Hypomethylation
ADAMTSL4 Hypomethylation
ADCY1 Hypomethylation
ADCY9 Hypomethylation

ADRA1B Hypomethylation
AFF4 Hypomethylation
AGAP2 Hypomethylation
AGBL2 Hypomethylation
AGPAT9 Hypomethylation
AIM1 Hypomethylation
AIP Hypomethylation
AJAP1 Hypomethylation
AK5 Hypomethylation
AKR1B1 Hypomethylation
ALAS1 Hypomethylation
ALDH1A3 Hypomethylation
ALDH2 Hypomethylation
ALOX15B Hypomethylation
ALX1 Hypomethylation
ANAPC4 Hypomethylation
ANGPT1 Hypomethylation
ANKLE1 Hypomethylation
ANKRD29 Hypomethylation
ANXA6 Hypomethylation
APC2 Hypomethylation
APCS Hypomethylation
APH1B Hypomethylation
APLP1 Hypomethylation
ARF5 Hypomethylation
ARHGEF11 Hypomethylation
ARHGEF7 Hypomethylation
ARID5A Hypomethylation
ARL10 Hypomethylation
ARL4A Hypomethylation
ARMC3 Hypomethylation
ARMC4 Hypomethylation
ARNTL2 Hypomethylation

ARRDC4 Hypomethylation
ARVCF Hypomethylation
ASAP1 Hypomethylation
ASAP2 Hypomethylation
ASCL1 Hypomethylation
ASTN2 Hypomethylation
ATP2A3 Hypomethylation
ATP6V0C Hypomethylation
AVPR1B Hypomethylation
B4GALT6 Hypomethylation
BACH1 Hypomethylation
BAMBI Hypomethylation
BATF Hypomethylation
BATF3 Hypomethylation
BCAN Hypomethylation
BCAT1 Hypomethylation
BIKHypomethylation
BLOC1S2 Hypomethylation
BMPERHypomethylation
BMS1 Hypomethylation
BNC1 Hypomethylation
BNIP3 Hypomethylation
BSN Hypomethylation
BTBD11 Hypomethylation
C10orf107 Hypomethylation
C10orf125 Hypomethylation
C10orf82 Hypomethylation
C13orf15 Hypomethylation
C13orf28 Hypomethylation
C13orf36 Hypomethylation
C17orf57 Hypomethylation
C17orf79 Hypomethylation
C19orf26 Hypomethylation

C19orf35	Hypomethylation
C19orf41	Hypomethylation
C19orf59	Hypomethylation
C1orf109	Hypomethylation
C1orf35	Hypomethylation
C1orf65	Hypomethylation
C1orf88	Hypomethylation
C1QL1	Hypomethylation
C1QTNF9	Hypomethylation
C20orf103	Hypomethylation
C20orf24	Hypomethylation
C22orf28	Hypomethylation
C2orf40	Hypomethylation
C3orf64	Hypomethylation
C4orf32	Hypomethylation
C4orf36	Hypomethylation
C6orf118	Hypomethylation
C6orf204	Hypomethylation
C7orf52	Hypomethylation
C8orf38	Hypomethylation
C9orf41	Hypomethylation
CA3	Hypomethylation
CAB39	Hypomethylation
CACNA1G	Hypomethylation
CACNA2D1	Hypomethylation
CAMK1D	Hypomethylation
CAMK4	Hypomethylation
CAV1	Hypomethylation
CBX2	Hypomethylation
CCDC109B	Hypomethylation
CCDC116	Hypomethylation
CCDC64	Hypomethylation
CCDC86	Hypomethylation

CCK Hypomethylation
CCND2 Hypomethylation
CCNJ Hypomethylation
CD1B Hypomethylation
CD1C Hypomethylation
CD1D Hypomethylation
CD38 Hypomethylation
CD44 Hypomethylation
CDC42BPA Hypomethylation
CDC42EP5 Hypomethylation
CDH13 Hypomethylation
CDH4 Hypomethylation
CDH6 Hypomethylation
CDH8 Hypomethylation
CDK20 Hypomethylation
CEACAM6 Hypomethylation
CEACAM7 Hypomethylation
CEBPG Hypomethylation
CECR6 Hypomethylation
CEND1 Hypomethylation
CENPA Hypomethylation
CEP250 Hypomethylation
CGREF1 Hypomethylation
CHCHD6 Hypomethylation
CHD5 Hypomethylation
CHFR Hypomethylation
CHGB Hypomethylation
CHN2 Hypomethylation
CHP2 Hypomethylation
CHRDL2 Hypomethylation
CHRNA4 Hypomethylation
CHRNA7 Hypomethylation
CHRNB1 Hypomethylation

CHRNB2 Hypomethylation
CHST13 Hypomethylation
CHSY1 Hypomethylation
CISH Hypomethylation
CKB Hypomethylation
CLDN8 Hypomethylation
CLEC14A Hypomethylation
CLEC3A Hypomethylation
CLIP2 Hypomethylation
CLIP4 Hypomethylation
CLPTM1L Hypomethylation
CLVS1 Hypomethylation
CMTM2 Hypomethylation
CNIH2 Hypomethylation
CNOT6 Hypomethylation
CNRIP1 Hypomethylation
CNTN1 Hypomethylation
CNTN2 Hypomethylation
COG2 Hypomethylation
COL11A1 Hypomethylation
COL12A1 Hypomethylation
COL14A1 Hypomethylation
COL19A1 Hypomethylation
COL1A2 Hypomethylation
COL5A2 Hypomethylation
COL6A2 Hypomethylation
COL7A1 Hypomethylation
COL9A2 Hypomethylation
COL9A3 Hypomethylation
COLEC12 Hypomethylation
COMP Hypomethylation
COQ3 Hypomethylation
CORIN Hypomethylation

CPA5 Hypomethylation
CPD Hypomethylation
CPT1A Hypomethylation
CPT1C Hypomethylation
CPXM1 Hypomethylation
CPXM2 Hypomethylation
CRABP1 Hypomethylation
CREB1 Hypomethylation
CREB3L3 Hypomethylation
CRHR2 Hypomethylation
CRIM1 Hypomethylation
CRYGN Hypomethylation
CSPG5 Hypomethylation
CST7 Hypomethylation
CTDP1 Hypomethylation
CTHRC1 Hypomethylation
CTNND2 Hypomethylation
CTPS Hypomethylation
CTSL1 Hypomethylation
CUL9 Hypomethylation
CUX2 Hypomethylation
CWH43 Hypomethylation
CXCL12 Hypomethylation
CXCL6 Hypomethylation
CYB5R1 Hypomethylation
CYB5R2 Hypomethylation
CYGB Hypomethylation
CYP4F22 Hypomethylation
D4S234E Hypomethylation
DAPK1 Hypomethylation
DCHS2 Hypomethylation
DDAH2 Hypomethylation
DDOST Hypomethylation

DDX1 Hypomethylation
DEFB118 Hypomethylation
DEFB125 Hypomethylation
DEFB129 Hypomethylation
DENND3 Hypomethylation
DGAT1 Hypomethylation
DGKE Hypomethylation
DHRS12 Hypomethylation
DISP2 Hypomethylation
DKK2 Hypomethylation
DLEC1 Hypomethylation
DLX5 Hypomethylation
DMRT1 Hypomethylation
DNAJB6 Hypomethylation
DNAJC6 Hypomethylation
DOCK3 Hypomethylation
DOLPP1 Hypomethylation
DPH1 Hypomethylation
DPP7 Hypomethylation
DPYSL5 Hypomethylation
DRD1 Hypomethylation
DRD2 Hypomethylation
DRD3 Hypomethylation
DSC3 Hypomethylation
DSCR6 Hypomethylation
DTNBP1 Hypomethylation
DUSP1 Hypomethylation
DUSP5 Hypomethylation
DYM Hypomethylation
DYRK3 Hypomethylation
DZIP1 Hypomethylation
EBF1 Hypomethylation
ECH1 Hypomethylation

EEF1A2 Hypomethylation
EFCAB4B Hypomethylation
EFNA2 Hypomethylation
EHHADH Hypomethylation
ELAVL3 Hypomethylation
ELK3 Hypomethylation
ELL2 Hypomethylation
ELOVL1 Hypomethylation
ELOVL2 Hypomethylation
ELOVL3 Hypomethylation
ELOVL4 Hypomethylation
EMILIN2 Hypomethylation
EMILIN3 Hypomethylation
EMX1 Hypomethylation
EN1 Hypomethylation
ENTPD3 Hypomethylation
EPAS1 Hypomethylation
EPHA5 Hypomethylation
EPHA8 Hypomethylation
EPO Hypomethylation
EREG Hypomethylation
ESAM Hypomethylation
ESM1 Hypomethylation
ETNK2 Hypomethylation
EYA1 Hypomethylation
EYA4 Hypomethylation
F2RHypomethylation
FADS1 Hypomethylation
FADS2 Hypomethylation
FAM105A Hypomethylation
FAM107B Hypomethylation
FAM109A Hypomethylation
FAM124B Hypomethylation

FAM125A	Hypomethylation
FAM150A	Hypomethylation
FAM163A	Hypomethylation
FAM171B	Hypomethylation
FAM20B	Hypomethylation
FAM43B	Hypomethylation
FAM46B	Hypomethylation
FAM57A	Hypomethylation
FAM57B	Hypomethylation
FAM65A	Hypomethylation
FAM71F1	Hypomethylation
FAM78A	Hypomethylation
FAM89A	Hypomethylation
FANCE	Hypomethylation
FANCG	Hypomethylation
FARP1	Hypomethylation
FBLN2	Hypomethylation
FBN1	Hypomethylation
FBN2	Hypomethylation
FCER1A	Hypomethylation
FCRL1	Hypomethylation
FCRL3	Hypomethylation
FCRL4	Hypomethylation
FEZ1	Hypomethylation
FFAR2	Hypomethylation
FGF10	Hypomethylation
FGF19	Hypomethylation
FGF22	Hypomethylation
FHIT	Hypomethylation
FIGNL1	Hypomethylation
FKBP9	Hypomethylation
FLT3	Hypomethylation
FLT4	Hypomethylation

FLVCR2 Hypomethylation
FN1 Hypomethylation
FOSL1 Hypomethylation
FOXC1 Hypomethylation
FOXD1 Hypomethylation
FOXD3 Hypomethylation
FOXE3 Hypomethylation
FOXF2 Hypomethylation
FOXH1 Hypomethylation
FSTHypomethylation
FUT9 Hypomethylation
GABRB3 Hypomethylation
GABRD Hypomethylation
GAMT Hypomethylation
GATA4 Hypomethylation
GCOM1 Hypomethylation
GDF10 Hypomethylation
GDF5 Hypomethylation
GDF7 Hypomethylation
GDNF Hypomethylation
GDPD5 Hypomethylation
GFI1 Hypomethylation
GFPT1 Hypomethylation
GIMAP1 Hypomethylation
GIPC2 Hypomethylation
GIPR Hypomethylation
GJC1 Hypomethylation
GJD4 Hypomethylation
GLB1L3 Hypomethylation
GLI3 Hypomethylation
GLP1R Hypomethylation
GLT1D1 Hypomethylation
GLT25D2 Hypomethylation

GLT8D2 Hypomethylation
GNA15 Hypomethylation
GNB4 Hypomethylation
GNS Hypomethylation
GPM6A Hypomethylation
GPR133 Hypomethylation
GPR150 Hypomethylation
GPR153 Hypomethylation
GPR160 Hypomethylation
GPR3 Hypomethylation
GPR6 Hypomethylation
GPR63 Hypomethylation
GPR68 Hypomethylation
GPR83 Hypomethylation
GPX7 Hypomethylation
GREM1 Hypomethylation
GRID2 Hypomethylation
GRIK1 Hypomethylation
GSC2 Hypomethylation
GSX2 Hypomethylation
GUCY2D Hypomethylation
HAS1 Hypomethylation
HBQ1 Hypomethylation
HBZ Hypomethylation
HCRTR1 Hypomethylation
HEPACAM Hypomethylation
HERPUD1 Hypomethylation
HES6 Hypomethylation
HEYL Hypomethylation
HHEX Hypomethylation
HHIP Hypomethylation
HHIPL1 Hypomethylation
HIST1H4L Hypomethylation

HMGN1 Hypomethylation
HNRNPF Hypomethylation
HOXA13 Hypomethylation
HOXA6 Hypomethylation
HOXA7 Hypomethylation
HOXC10 Hypomethylation
HOXC11 Hypomethylation
HOXC13 Hypomethylation
HOXC9 Hypomethylation
HOXD11 Hypomethylation
HOXD13 Hypomethylation
HOXD9 Hypomethylation
HPCAL1 Hypomethylation
HPS1 Hypomethylation
HR Hypomethylation
HRC Hypomethylation
HRH3 Hypomethylation
HSD17B4 Hypomethylation
HSPA12B Hypomethylation
HSPA2 Hypomethylation
HTR1E Hypomethylation
HTR2A Hypomethylation
HTR3E Hypomethylation
HTR5A Hypomethylation
HTR7 Hypomethylation
ICMT Hypomethylation
IER5 Hypomethylation
IFIT5 Hypomethylation
IFNGR2 Hypomethylation
IGDCC3 Hypomethylation
IGF2BP1 Hypomethylation
IGFBP2 Hypomethylation
IGFBP3 Hypomethylation

IGFBP7 Hypomethylation
IHH Hypomethylation
IL12RB2 Hypomethylation
IMP4 Hypomethylation
IMPDH1 Hypomethylation
INA Hypomethylation
INPP5B Hypomethylation
IQGAP2 Hypomethylation
IRAK3 Hypomethylation
IRX4 Hypomethylation
IRX5 Hypomethylation
ISL1 Hypomethylation
ISL2 Hypomethylation
ISM2 Hypomethylation
ITGA11 Hypomethylation
ITGB1BP3 Hypomethylation
ITGBL1 Hypomethylation
JAG2 Hypomethylation
JAKMIP2 Hypomethylation
JAM2 Hypomethylation
JPH1 Hypomethylation
KANK3 Hypomethylation
KCNA2 Hypomethylation
KCNA3 Hypomethylation
KCNA4 Hypomethylation
KCNA7 Hypomethylation
KCNAB1 Hypomethylation
KCNG1 Hypomethylation
KCNH1 Hypomethylation
KCNH4 Hypomethylation
KCNH7 Hypomethylation
KCNK12 Hypomethylation
KCNK6 Hypomethylation

KCNQ1 Hypomethylation
KCNS2 Hypomethylation
KCNS3 Hypomethylation
KCTD12 Hypomethylation
KCTD8 Hypomethylation
KIF17 Hypomethylation
KIF5A Hypomethylation
KIT Hypomethylation
KLF11 Hypomethylation
KLF14 Hypomethylation
KLHL21 Hypomethylation
KRT86 Hypomethylation
KRTDAP Hypomethylation
L3MBTL2 Hypomethylation
LAMA1 Hypomethylation
LAMA2 Hypomethylation
LCN15 Hypomethylation
LDHB Hypomethylation
LEMD3 Hypomethylation
LGI3 Hypomethylation
LGTN Hypomethylation
LHCGR Hypomethylation
LHX1 Hypomethylation
LHX3 Hypomethylation
LHX9 Hypomethylation
LILRA2 Hypomethylation
LILRB2 Hypomethylation
LIMK1 Hypomethylation
LIN7A Hypomethylation
LOC284805 Hypomethylation
LOC389458 Hypomethylation
LRFN5 Hypomethylation
LRPAP1 Hypomethylation

LSAMP Hypomethylation
LTK Hypomethylation
LUC7L Hypomethylation
LYN Hypomethylation
MACF1 Hypomethylation
MADCAM1 Hypomethylation
MAFB Hypomethylation
MAFK Hypomethylation
MAK16 Hypomethylation
MAL Hypomethylation
MAP1D Hypomethylation
MAP6D1 Hypomethylation
MBD3L1 Hypomethylation
MC3R Hypomethylation
MCAM Hypomethylation
MCHR2 Hypomethylation
MCOLN3 Hypomethylation
MDFI Hypomethylation
MEOX2 Hypomethylation
MESP1 Hypomethylation
METRN Hypomethylation
MGC42105 Hypomethylation
MGMT Hypomethylation
MIXL1 Hypomethylation
MKX Hypomethylation
MLNR Hypomethylation
MMP2 Hypomethylation
MMP9 Hypomethylation
MNDA Hypomethylation
MNS1 Hypomethylation
MNX1 Hypomethylation
MOBKL2A Hypomethylation
MOSC1 Hypomethylation

MOXD1	Hypomethylation
MPP2	Hypomethylation
MRPL16	Hypomethylation
MSC	Hypomethylation
MT3	Hypomethylation
MTHFS	Hypomethylation
MYADM	Hypomethylation
MYCL1	Hypomethylation
MYOD1	Hypomethylation
MYOM2	Hypomethylation
NALCN	Hypomethylation
NANP	Hypomethylation
NBPF3	Hypomethylation
NCCRP1	Hypomethylation
NCL	Hypomethylation
NCOA2	Hypomethylation
NDRG2	Hypomethylation
NDRG4	Hypomethylation
NDUFAF1	Hypomethylation
NDUFS5	Hypomethylation
NEBL	Hypomethylation
NEFH	Hypomethylation
NEGR1	Hypomethylation
NELL1	Hypomethylation
NES	Hypomethylation
NETO2	Hypomethylation
NEUROD2	Hypomethylation
NEUROD6	Hypomethylation
NEUROG1	Hypomethylation
NEUROG3	Hypomethylation
NFASC	Hypomethylation
NFKBIL2	Hypomethylation
NFYB	Hypomethylation

NGF	Hypomethylation
NID2	Hypomethylation
NINL	Hypomethylation
NKX2-1	Hypomethylation
NKX2-2	Hypomethylation
NKX3-1	Hypomethylation
NKX6-1	Hypomethylation
NLRP6	Hypomethylation
NMUR2	Hypomethylation
NOL4	Hypomethylation
NOTCH3	Hypomethylation
NPBWR1	Hypomethylation
NPTX1	Hypomethylation
NPY	Hypomethylation
NPY5R	Hypomethylation
NRM	Hypomethylation
NRN1	Hypomethylation
NTM	Hypomethylation
NTN3	Hypomethylation
NTNG1	Hypomethylation
NTRK2	Hypomethylation
NTRK3	Hypomethylation
NUAK1	Hypomethylation
NUMBL	Hypomethylation
NUP54	Hypomethylation
NUPL1	Hypomethylation
NXN	Hypomethylation
ODF3L2	Hypomethylation
OGDH	Hypomethylation
OLFM1	Hypomethylation
OLFM2	Hypomethylation
OLFML2B	Hypomethylation
ONECUT2	Hypomethylation

OPCML Hypomethylation
OPRK1 Hypomethylation
OR10H2 Hypomethylation
OSR1 Hypomethylation
OSTBETA Hypomethylation
OTOP3 Hypomethylation
OTOS Hypomethylation
P2RX5 Hypomethylation
P4HA3 Hypomethylation
PAK7 Hypomethylation
PALM Hypomethylation
PANK4 Hypomethylation
PAPSS1 Hypomethylation
PAQR9 Hypomethylation
PAX5 Hypomethylation
PAX9 Hypomethylation
PBX4 Hypomethylation
PCDH9 Hypomethylation
PCGF5 Hypomethylation
PCGF6 Hypomethylation
PCSK1 Hypomethylation
PDE1B Hypomethylation
PDE4C Hypomethylation
PDE8A Hypomethylation
PDE8B Hypomethylation
PDGFD Hypomethylation
PDLIM2 Hypomethylation
PET112L Hypomethylation
PF4V1 Hypomethylation
PGCP Hypomethylation
PHF10 Hypomethylation
PHF13 Hypomethylation
PHOX2A Hypomethylation

PITX2 Hypomethylation
PKIA Hypomethylation
PKIG Hypomethylation
PLD5 Hypomethylation
PLD6 Hypomethylation
PLEK2 Hypomethylation
PLEKHO1 Hypomethylation
PNMAL1 Hypomethylation
PNOC Hypomethylation
PODN Hypomethylation
POLR2D Hypomethylation
POLR3D Hypomethylation
POMC Hypomethylation
POPDC3 Hypomethylation
POU2AF1 Hypomethylation
POU2F2 Hypomethylation
POU3F1 Hypomethylation
POU3F2 Hypomethylation
POU3F3 Hypomethylation
PPDPF Hypomethylation
PPFIA2 Hypomethylation
PPP1CC Hypomethylation
PPP1R14A Hypomethylation
PPP1R14C Hypomethylation
PPP1R2 Hypomethylation
PPP1R9A Hypomethylation
PPP4R4 Hypomethylation
PPPDE1 Hypomethylation
PRAP1 Hypomethylation
PRDM12 Hypomethylation
PREX1 Hypomethylation
PRG3 Hypomethylation
PRKCB Hypomethylation

PRKCDBP Hypomethylation
PRKCZ Hypomethylation
PRKD1 Hypomethylation
PROK2 Hypomethylation
PROX1 Hypomethylation
PRPH Hypomethylation
PRRC1 Hypomethylation
PRRT2 Hypomethylation
PRRX2 Hypomethylation
PRSS23 Hypomethylation
PRSS35 Hypomethylation
PRTFDC1 Hypomethylation
PRTN3 Hypomethylation
PSD3 Hypomethylation
PSMD11 Hypomethylation
PTCH2 Hypomethylation
PTGDR Hypomethylation
PTGER2 Hypomethylation
PTGFR Hypomethylation
PTHLH Hypomethylation
PTPRN Hypomethylation
PTPRO Hypomethylation
PUF60 Hypomethylation
PWWP2B Hypomethylation
R3HDML Hypomethylation
RAB11FIP4 Hypomethylation
RAB15 Hypomethylation
RAB31 Hypomethylation
RALBP1 Hypomethylation
RAP1GAP Hypomethylation
RARRES1 Hypomethylation
RARRES2 Hypomethylation
RASGRP2 Hypomethylation

RBM15B Hypomethylation
RBM38 Hypomethylation
RBM5 Hypomethylation
RBP4 Hypomethylation
RDH8 Hypomethylation
RGS7 Hypomethylation
RHAG Hypomethylation
RHBDD1 Hypomethylation
RHCG Hypomethylation
RHOBTB1 Hypomethylation
RHOJ Hypomethylation
RNASEH2A Hypomethylation
RND2 Hypomethylation
RNF213 Hypomethylation
RNF4 Hypomethylation
ROPN1 Hypomethylation
ROR2 Hypomethylation
RORA Hypomethylation
RPH3A Hypomethylation
RPL31 Hypomethylation
RPL39L Hypomethylation
RPP25 Hypomethylation
RPS6KC1 Hypomethylation
RRN3 Hypomethylation
RSPO2 Hypomethylation
RTEL1 Hypomethylation
RTN1 Hypomethylation
RTP1 Hypomethylation
RUNDC3A Hypomethylation
RWDD2B Hypomethylation
S1PR5 Hypomethylation
SARS Hypomethylation
SCAND2 Hypomethylation

SCARF2 Hypomethylation
SCG3 Hypomethylation
SCMH1 Hypomethylation
SCN5A Hypomethylation
SCUBE1 Hypomethylation
SCUBE2 Hypomethylation
SCUBE3 Hypomethylation
SEMA3E Hypomethylation
SEMA5A Hypomethylation
3-Sep Hypomethylation
SERPINE2 Hypomethylation
SFMBT1 Hypomethylation
SFRP1 Hypomethylation
SFRP2 Hypomethylation
SFRP4 Hypomethylation
SFRP5 Hypomethylation
SFRS7 Hypomethylation
SH3BGRL3 Hypomethylation
SHISA4 Hypomethylation
SHOX2 Hypomethylation
SIM2 Hypomethylation
SKAP1 Hypomethylation
SLC13A5 Hypomethylation
SLC17A6 Hypomethylation
SLC18A2 Hypomethylation
SLC1A6 Hypomethylation
SLC22A16 Hypomethylation
SLC24A3 Hypomethylation
SLC25A10 Hypomethylation
SLC25A39 Hypomethylation
SLC26A5 Hypomethylation
SLC29A1 Hypomethylation
SLC2A13 Hypomethylation

SLC2A2	Hypomethylation
SLC30A2	Hypomethylation
SLC35E3	Hypomethylation
SLC38A3	Hypomethylation
SLC39A3	Hypomethylation
SLC46A2	Hypomethylation
SLC4A11	Hypomethylation
SLC6A11	Hypomethylation
SLC6A15	Hypomethylation
SLC6A4	Hypomethylation
SLC6A5	Hypomethylation
SLC7A10	Hypomethylation
SLC8A2	Hypomethylation
SLC9A2	Hypomethylation
SLC9A3	Hypomethylation
SLCO5A1	Hypomethylation
SLITRK1	Hypomethylation
SMAD2	Hypomethylation
SMAGP	Hypomethylation
SMO	Hypomethylation
SNCA	Hypomethylation
SNCAIP	Hypomethylation
SND1	Hypomethylation
SNN	Hypomethylation
SNRNP200	Hypomethylation
SNTB1	Hypomethylation
SNTG1	Hypomethylation
SNX31	Hypomethylation
SOAT1	Hypomethylation
SOCS2	Hypomethylation
SORD	Hypomethylation
SOX8	Hypomethylation
SPARC	Hypomethylation

SPATS1 Hypomethylation
SPG11 Hypomethylation
SPIB Hypomethylation
SPOCK2 Hypomethylation
SRD5A2 Hypomethylation
SRRT Hypomethylation
SRXN1 Hypomethylation
SSBP4 Hypomethylation
ST6GAL2 Hypomethylation
ST8SIA5 Hypomethylation
STC2 Hypomethylation
STEAP4 Hypomethylation
STX2 Hypomethylation
STXBP2 Hypomethylation
SULT1C4 Hypomethylation
SUMF1 Hypomethylation
SUSD1 Hypomethylation
SYNE2 Hypomethylation
SYNGR3 Hypomethylation
SYNM Hypomethylation
SYT10 Hypomethylation
SYT5 Hypomethylation
SYT7 Hypomethylation
TAC1 Hypomethylation
TACR3 Hypomethylation
TBC1D13 Hypomethylation
TBX1 Hypomethylation
TBX20 Hypomethylation
TBX21 Hypomethylation
TBX3 Hypomethylation
TCF15 Hypomethylation
TCFL5 Hypomethylation
TCP11L1 Hypomethylation

TCTE1 Hypomethylation
TEKT3 Hypomethylation
TET2 Hypomethylation
TEX264 Hypomethylation
TFAP2D Hypomethylation
TGFBI Hypomethylation
TGFBR3 Hypomethylation
THBD Hypomethylation
THBS4 Hypomethylation
THNSL2 Hypomethylation
THY1 Hypomethylation
TIAM1 Hypomethylation
TLE4 Hypomethylation
TLX3 Hypomethylation
TM4SF19 Hypomethylation
TM6SF1 Hypomethylation
TMEFF2 Hypomethylation
TMEM121 Hypomethylation
TMEM132D Hypomethylation
TMEM179 Hypomethylation
TMEM215 Hypomethylation
TMEM22 Hypomethylation
TMEM25 Hypomethylation
TMEM42 Hypomethylation
TMEM59L Hypomethylation
TMEM90B Hypomethylation
TMPRSS2 Hypomethylation
TMPRSS6 Hypomethylation
TMPRSS9 Hypomethylation
TNFRSF10A Hypomethylation
TNFRSF10C Hypomethylation
TNFRSF11A Hypomethylation
TNFRSF18 Hypomethylation

TNFRSF4 Hypomethylation
TNFRSF8 Hypomethylation
TNFRSF9 Hypomethylation
TNIP2 Hypomethylation
TNXB Hypomethylation
TP73 Hypomethylation
TPPP3 Hypomethylation
TRAM1 Hypomethylation
TREML2 Hypomethylation
TRIB3 Hypomethylation
TRIM36 Hypomethylation
TRIM40 Hypomethylation
TRIM58 Hypomethylation
TRPC1 Hypomethylation
TRPC4AP Hypomethylation
TRPV4 Hypomethylation
TSKU Hypomethylation
TSPYL5 Hypomethylation
TTC33 Hypomethylation
TUBB2A Hypomethylation
TUBB3 Hypomethylation
TWIST1 Hypomethylation
UAP1L1 Hypomethylation
UBE2T Hypomethylation
UCHL1 Hypomethylation
UFSP2 Hypomethylation
UNC80 Hypomethylation
USH2A Hypomethylation
USP2 Hypomethylation
USP4 Hypomethylation
VAV3 Hypomethylation
VAX2 Hypomethylation
VEGFC Hypomethylation

VGF	Hypomethylation
VHL	Hypomethylation
VPS37B	Hypomethylation
VSTM1	Hypomethylation
VSX2	Hypomethylation
WDFY2	Hypomethylation
WDR63	Hypomethylation
WEE1	Hypomethylation
WFS1	Hypomethylation
WNT2	Hypomethylation
XBP1	Hypomethylation
XYLB	Hypomethylation
YPEL4	Hypomethylation
YTHDC1	Hypomethylation
ZDHHC11	Hypomethylation
ZFP37	Hypomethylation
ZNF197	Hypomethylation
ZNF222	Hypomethylation
ZNF23	Hypomethylation
ZNF238	Hypomethylation
ZNF280A	Hypomethylation
ZNF287	Hypomethylation
ZNF343	Hypomethylation
ZNF35	Hypomethylation
ZNF514	Hypomethylation
ZNF530	Hypomethylation
ZNF532	Hypomethylation
ZNF536	Hypomethylation
ZNF546	Hypomethylation
ZNF569	Hypomethylation
ZNF575	Hypomethylation
ZNF584	Hypomethylation
ZNF678	Hypomethylation

ZNF781 Hypomethylation
ZNF800 Hypomethylation
ZNF804A Hypomethylation
ZNHIT6 Hypomethylation

Supplementary table 6. A total of 2213 DEGs were obtained between C2 and C1 molecular subtypes

	logFC	AveExpr	t	P.Value	adj.P.Val	B
CLDN6	2.152310354	5.707754455	6.743847298	5.98E-11	4.84E-08	14.46722993
IGF2	2.025137217	6.920790004	4.878265194	1.60E-06	9.32E-05	4.857270145
DPEP3	1.758987535	2.134409132	5.097353005	5.52E-07	4.60E-05	5.850501281
CTCFL	1.575609709	2.544204292	7.405309901	8.98E-13	1.82E-09	18.45574107
KIF1A	1.485203828	3.730562897	6.200890638	1.51E-09	6.77E-07	11.41019272
TKTL1	1.479910899	1.385069088	5.831774153	1.20E-08	2.38E-06	9.450104823
HIF3A	1.47481119	2.857399512	8.168853656	5.03E-15	2.71E-11	23.39386382
MFAP2	1.449104725	5.851249758	7.152588189	4.62E-12	5.75E-09	16.89883172
MAGEA4	1.32409669	1.562846743	5.277473983	2.24E-07	2.46E-05	6.695260366
CNTFR	1.272880908	2.770337599	5.209569752	3.16E-07	3.17E-05	6.373833892
WNT11	1.269299419	3.885516421	5.477218228	8.01E-08	1.11E-05	7.661285588
FBXO2	1.255870058	4.230305574	6.527944811	2.21E-10	1.55E-07	

13.22743571
FXYD7 1.215418866 2.0791165286.327107409 7.25E-10 4.04E-07
12.10269102
TUBB2B 1.205417818 2.0611095084.829301672 2.01E-06
0.000109973 4.640503322
XAGE2 1.196002697 2.1157385874.941476957 1.18E-06 7.48E-05
5.139941411
NUPR2 1.172826898 4.352760875 5.56606323 5.02E-08 7.39E-06
8.100710208
IGLON5 1.163823043 1.976219884 5.359508856 1.47E-07 1.81E-
05 7.088314586
LHX1 1.152086153 2.514216962 3.953249691 9.24E-05 0.00174091
1.093441966
INA 1.15200831 1.9860118966.168967075 1.81E-09 7.32E-07
11.23681859
ALG1L 1.1103302932.859098084 7.689630632 1.36E-13 5.49E-10
20.25434018
PRSS50 1.1102502842.5400119066.122308551 2.36E-09 8.69E-07
10.98471982
GTSF1 1.106541789 1.541430451 4.830052577 2.00E-06
0.000109955 4.643813187
NEFH 1.104508894 2.2511708285.057462246 6.71E-07 5.15E-05
5.666838077
GCK 1.088568604 1.20547644 8.462027837 6.27E-16 5.07E-12
25.37832662
PHOX2A 1.083128147 3.187327282 4.128609625 4.52E-05
0.001023623 1.752289019
GAL3ST3 1.074291778 2.681923825 4.435820673 1.21E-05
0.000402821 2.9688367
RNF2121.074236164 2.930612143 4.781986526 2.51E-06
0.000132029 4.432857342
GABRA3 1.05887176 1.017614844 7.283151413 1.99E-12 3.58E-09
17.698182

SFRP5	1.045630494	1.42166936	6.254497174	1.11E-09	5.26E-07	11.70294315
WDR72	1.040262888	2.241899804	5.114443196	5.07E-07	4.29E-05	5.92956868
ALPL	1.015687624	6.109090396	5.063976773	6.50E-07	5.08E-05	5.696746658
CDKN1C	1.01364522	5.246934174	7.153932391	4.58E-12	5.75E-09	16.90700643
PTGDS	1.009651733	6.834836234	3.936019223	9.90E-05	0.001835309	1.030116093
LTBP4	0.974475376	5.329825078	5.740976826	1.97E-08	3.46E-06	8.98311215
ECEL1	0.963082361	2.204797248	4.951106212	1.13E-06	7.24E-05	5.183280264
COL9A3	0.956377154	1.728002836	5.020625844	8.04E-07	5.66E-05	5.498347834
GNGT1	0.949500281	1.620930592	8.669865246	1.39E-16	2.25E-12	26.81320014
LRATD1	0.947547784	2.3230191	5.388258715	1.27E-07	1.62E-05	7.227284935
CLDN9	0.947110661	3.956097962	4.722014198	3.32E-06	0.000160159	4.1722504
ECRG4	0.935034401	3.160204915	4.481441285	9.91E-06	0.000347752	3.156175994
CLDN19	0.930482614	0.850901644	5.961240754	5.86E-09	1.66E-06	10.12641717
PDCL2	0.926763653	1.350249379	4.730109949	3.20E-06	0.000156482	4.207260868
APOA1	0.926720969	6.520570615	3.514559645	0.000495316	0.005744247	-0.439183491
ITM2C	0.922353321	7.208141027	4.827398276	2.03E-06	0.000110323	4.632115487
FOXA3	0.919950022	1.469993666	5.937259833	6.70E-09	1.79E-06	

10.00022503
LGALS7B 0.91715121 1.668963109 5.417873969 1.09E-07 1.46E-05
7.371098264
PLEKHG4 0.912638798 2.9641173856.821902454 3.70E-11 3.32E-08
14.9231365
DRD4 0.9023411042.946481315 6.154510782 1.97E-09 7.57E-07
11.1585451
SEMA6C 0.901659937 3.153418684 7.414683762 8.45E-13 1.82E-
09 18.51425449
NPW 0.900366685 4.62743167 4.436128532 1.21E-05 0.000402821
2.970095164
CLIP3 0.899425953 5.181742763 6.46123611 3.29E-10 2.13E-07
12.85076655
SAMD11 0.886296061 2.73900193 4.005467484 7.49E-05
0.001491842 1.286900302
FAM171A2 0.875942664 4.7711448435.588237691 4.47E-08 6.75E-06
8.211311083
MEX3A0.870703549 4.8609291145.929489254 7.00E-09 1.79E-06
9.959424202
NKX6-10.864133322 1.737068866 4.452104972 1.13E-05
0.000382748 3.035510761
FIGNL20.85917129 1.583992914 6.864377894 2.84E-11 2.71E-08
15.17292133
DES 0.855951898 3.399789547 4.167826695 3.84E-05 0.00090993
1.903191378
ZIC1 0.849077626 1.692297342 3.638169055 0.000313737
0.004118592-0.024221413
LGR5 0.84775158 3.376122852 3.338527004 0.000928141
0.009027103 -1.006981832
TNNT3 0.847025555 0.955228396 5.930382146 6.96E-09 1.79E-06
9.964110268
HMGA2 0.829099654 2.28211092 4.163647889 3.90E-05
0.000916578 1.887050222

RCOR2 0.828010607 3.768482135 4.720753198 3.34E-06
0.000160515 4.166801905
PTH2R 0.827053482 2.675757435 4.133284532 4.43E-05
0.001008157 1.77020945
CCDC3 0.826224989 5.0442141154.512335792 8.63E-06 0.000316771
3.284015412
NKAIN4 0.825980537 1.458552222 4.270543714 2.48E-05
0.000665382 2.304555557
GDF11 0.82309566 3.539423831 5.901082358 8.19E-09 1.92E-06
9.810641834
PNMT 0.818795623 1.581372444 5.330666553 1.71E-07 2.01E-05
6.949533149
C10orf82 0.813961099 1.171075518 7.061885665 8.23E-12 9.51E-09 16.34989139
SULT1C4 0.80591648 1.829354978 5.3436311341.60E-07 1.92E-05
7.011836352
FXYD1 0.80573054 1.9211036465.5411017845.73E-08 8.27E-06
7.976650553
EYA4 0.805490847 1.746279593 4.775588868 2.59E-06
0.000135166 4.404918667
DACH1 0.804551888 2.822052267 4.289397607 2.29E-05
0.000629435 2.379185553
NDUFA4L2 0.80334118 4.970974712 4.377581988 1.57E-05
0.000477138 2.732177961
FRZB 0.802781301 3.578433822 4.3299117911.92E-05 0.000554465
2.540556581
NCCRP1 0.80054085 2.042417083 4.271660349 2.47E-05
0.000663786 2.308967307
TSPYL5 0.799381557 4.651024908 5.367806901 1.41E-07 1.76E-05 7.128360584
PDIA2 0.798612294 1.5733363 5.024034861 7.90E-07 5.66E-05
5.513895875
PPP1R14A 0.798342149 4.991798205 4.5947534115.96E-06

0.000246896 3.628880119

DLK1 0.795206147 1.444142885 3.133205605 0.001867518 0.014602563 -1.634555681

COL9A1 0.794041596 1.548227367 4.20897289 3.23E-05 0.00079937 2.06290727

BMP7 0.793089627 4.709192803 3.180925531 0.001592518 0.013042305 -1.492050586

UCHL1 0.78998584 6.072466262 3.419369444 0.000697879 0.0073171 -0.749614051

THBS4 0.7891105962.196910108 5.047299032 7.05E-07 5.26E-05 5.620244753

FGF17 0.784963919 0.967206883 4.194562306 3.43E-05 0.000842979 2.00680839

FBLN2 0.783253823 3.986075207 3.909787328 0.00010995 0.001998997 0.934195031

MFAP4 0.783163075 5.1031176133.241555507 0.001297091 0.01125769 -1.308058617

RSPO3 0.7811020792.73865794 3.528828551 0.000470193 0.005548341 -0.391964393

PRAME0.77581014 5.722175799 3.885743126 0.000120945 0.002127303 0.846790844

CPAMD8 0.770522795 2.899742324 4.4351160641.22E-05 0.000403246 2.965956702

RSPO4 0.769525279 2.828328132 3.835203964 0.000147539 0.002426682 0.664689914

COL2A1 0.766644561 1.190232072 3.958780024 9.04E-05 0.00170469 1.113820915

ANKLE1 0.761775974 1.930031944 5.40914598 1.14E-07 1.50E-05 7.328645169

TCF7L10.758274817 3.809575202 4.54414086 7.49E-06 0.000289116 3.416440702

SOX15 0.757062221 2.349590132 4.250287909 2.71E-05 0.000707821 2.224707095

SEMA3E 0.746366453 1.486849028 4.500234504 9.11E-06 0.000328395 3.233847694

IGFBP2 0.745268185 8.651783788 4.945475208 1.16E-06 7.37E-05 5.157927606

TRO 0.743169844 3.086909056 5.389572201 1.26E-07 1.62E-05 7.233649116

RND2 0.743081953 1.894149231 6.182996258 1.67E-09 6.93E-07 11.31292107

ADRA2B 0.7413001141.734364617 5.321494824 1.79E-07 2.06E-05 6.905534955

PHOSPHO1 0.737992996 2.202775176 4.222805981 3.04E-05 0.000768425 2.116921863

EPHX3 0.73660433 2.565174393 3.269764415 0.0011777280.010555835 -1.221337408

FOXO6 0.736547557 4.325237407 4.759336383 2.79E-06 0.0001426114.334091821

CACNA1B 0.736430662 1.615579613 5.890407004 8.69E-09 1.98E-06 9.754880939

SLC44A5 0.735456386 1.493407151 5.309762636 1.90E-07 2.17E-05 6.849348039

UPK2 0.732244379 2.192746446 3.871524299 0.000127928 0.002209805 0.795336438

DTNA 0.729563231 1.438773862 5.930429181 6.96E-09 1.79E-06 9.964357131

LMO3 0.726457069 1.724867582 3.8331130340.00014875 0.002440646 0.657203212

FIGN 0.722709921 2.024002356 5.872893966 9.57E-09 2.11E-06 9.663584428

MXRA8 0.722173554 5.7731104593.745549969 0.000208809 0.003087858 0.347060237

EMID1 0.721257848 4.433492551 4.968734851 1.03E-06 6.80E-05 5.26281274

TMEM88 0.720929804 3.015390196 5.712212525 2.30E-08 4.00E-

06 8.836438809

DUSP15 0.71799508 1.927555487 5.746232508 1.91E-08 3.40E-06 9.009977758

SPINK5 0.717141856 1.449222628 6.928693446 1.90E-11 1.93E-08 15.55339683

POU6F2 0.715022966 0.668853638 6.965113703 1.52E-11 1.63E-08 15.7700488

NEURL1 0.711402516 1.691273249 4.367955399 1.63E-05 0.00049265 2.693329716

RPRM 0.711259156 1.945779813 4.416876244 1.32E-05 0.00042599 2.891546422

COLEC11 0.710108821 2.179525174 3.099770225 0.002085611 0.015818571 -1.733188251

FSCN1 0.709215332 6.636218069 4.888691141 1.52E-06 9.04E-05 4.903674064

IMPG2 0.707876436 1.719043595 3.40859733 0.000725137 0.007515222 -0.784241217

LRP4 0.707224855 1.723418368 4.378341665 1.56E-05 0.000477091 2.735246921

MYRF 0.707172453 4.074698443 3.60494147 0.000355162 0.004513592 -0.137079078

DEFB126 0.705840836 1.586431158 3.820851842 0.000156045 0.002537181 0.613376967

PRAP1 0.704892955 1.683739102 4.335402607 1.88E-05 0.000545003 2.56253202

REG1A 0.703913311 1.409945329 3.317318438 0.000999313 0.009470939 -1.073543325

SOX11 0.703520224 1.272336568 3.620952772 0.000334598 0.004330498 -0.082816708

COL9A2 0.701925819 4.606320799 3.839346001 0.000145166 0.002403788 0.679531825

TSPAN7 0.701855475 3.84342519 3.473600289 0.000574593 0.006410869 -0.573735062

PLBD1	0.701462694	4.394782025	5.020304271	8.05E-07	5.66E-05	5.496881658
HPN	0.698617611	4.7912624	2.920901953	0.00370504	0.023667305	-2.243762233
MYOD1	0.69604798	0.817898915	4.767404668	2.69E-06	0.00013821	4.369226108
HENMT1	0.693464556	3.77433424	5.885830421	8.91E-09	2.00E-06	9.731001353
WNT6	0.691175863	3.627389203	3.144577354	0.001798273	0.014205301	-1.600781362
NR5A1	0.690545291	1.69747037	3.322129712	0.000982736	0.009368714	-1.058478467
IGF2BP3	0.690379499	1.488174318	3.806398184	0.000165078	0.002649443	0.561880172
CLEC2L	0.690343717	0.775049131	4.312027051	2.08E-05	0.000588092	2.469151824
FABP6	0.68053536	2.569991763	2.75863629	0.006093733	0.033425017	-2.681895715
ATP1B2	0.676332093	2.397875146	3.487013858	0.00054741	0.006184354	-0.529833684
NTF3	0.675792654	1.942759342	3.715117315	0.000234579	0.003334921	0.240820474
TUBB4A	0.674535653	3.265022102	3.01727056	0.002727824	0.019153963	-1.972263454
FXYD4	0.674458697	1.654353216	3.488258494	0.000544949	0.006165169	-0.52575209
LRRN1	0.668290366	2.467978854	3.739221336	0.000213938	0.003142275	0.324901101
FZD2	0.668088723	4.678759468	4.148690641	4.16E-05	0.000960351	1.829396429
SLC7A10	0.667722818	0.791045629	5.652726649	3.17E-08	5.18E-06	8.535058909
NINL	0.663496543	2.881363265	5.919250884	7.40E-09	1.79E-06	

9.905732704

SELENOM 0.662657599 5.352667527 3.922304882 0.000104608 0.001927136 0.979894049

SFRP1 0.661848133 2.9180911723.027636476 0.002638195 0.018703204 -1.942560465

CSDC2 0.660991512 2.06409917 4.767854567 2.69E-06 0.00013821 4.371186786

NOTCH3 0.660132805 6.240754645 4.82323107 2.07E-06 0.0001120344.613761678

PSD 0.659575416 2.326000382 6.285542505 9.24E-10 4.65E-07 11.87340833

ID1 0.659366083 5.442149548 3.796760096 0.000171374 0.002712813 0.527640733

PDE6G 0.655870989 2.2011530933.689037279 0.000259018 0.003587664 0.150414034

LAMC3 0.654318375 2.19471679 4.304919518 2.14E-05 0.000601101 2.440848951

MAP7D2 0.653597765 1.716134679 4.556606673 7.08E-06 0.000278837 3.468569987

FBN3 0.651920853 2.326719369 3.317886915 0.000997341 0.009463336 -1.071764401

ACTL8 0.649455826 1.193759212 3.576198598 0.000395085 0.0048569 -0.233927637

FLNC 0.649080459 2.64383288 3.738279182 0.0002147110.003143529 0.321605199

MAGEC1 0.642020901 0.792976161 3.710710425 0.00023855 0.0033665110.225502587

MSI1 0.64127293 4.176305967 3.30671067 0.001036785 0.009712276 -1.106685382

EFS0.637338076 3.414605362 3.516019985 0.000492688 0.005726884 -0.434359088

ADAM33 0.631624884 1.95405419 3.9391168799.78E-05 0.001824094 1.041481989

CACNA2D20.630557685 2.193322632 3.936719849 9.88E-05
0.001832829 1.032686109
CPT1C 0.630510171 2.133601041 4.531737276 7.92E-06 0.00029716
3.36469788
GFRA3 0.627947402 1.465045242 4.369503353 1.62E-05
0.000490257 2.699571325
CASTOR2 0.6260894 3.146432124 4.878358129 1.59E-06 9.32E-05
4.857683395
PLCB1 0.624275417 2.088887301 6.185393976 1.65E-09 6.93E-07
11.32594164
EFNB3 0.621300076 3.458910642 3.883747986 0.000121902
0.002136656 0.839560471
EPOP 0.620632608 3.282146509 4.6566158114.49E-06 0.000198954
3.891375035
KCNS1 0.620247491 1.672562981 4.549029761 7.33E-06
0.000285512 3.436869858
MAP4K1 0.617998415 3.230760944 5.370255366 1.40E-07 1.75E-
05 7.140186832
BNIPL 0.617887015 3.079964365 2.972289227 0.003150032
0.021082074 -2.1000329
CLSTN3 0.615902779 5.270891087 5.079490077 6.02E-07 4.90E-
05 5.768103295
NACAD 0.615467403 2.437754138 5.004122351 8.71E-07 5.94E-
05 5.423207311
XKR9 0.612648056 1.318622905 5.839208981 1.15E-08 2.33E-06
9.48861223
NPY 0.609500375 1.375857537 2.869491903 0.004348285
0.026412896 -2.38516307
TPPP3 0.609467058 4.355317305 3.3311963030.00095219 0.009191251
-1.030033768
RAPSN 0.609215682 0.852352517 5.149973401 4.25E-07 3.93E-05
6.094678978
SNN 0.6091193764.575910821 5.788282074 1.52E-08 2.90E-06

9.225657523
C9orf24 0.609039507 2.446949197 2.679956765 0.007693559 0.039375642 -2.88570211
CRISPLD1 0.602024817 2.447519564 3.851653794 0.000138328 0.0023311220.723720607
TMEFF1 0.599674184 1.3072790115.828829208 1.22E-08 2.38E-06 9.434863189
KCNG1 0.598929234 2.250698974 3.476322194 0.000568977 0.006361371 -0.564839302
CFTR 0.596906839 1.503478017 3.093092165 0.002131888 0.016086604 -1.75276805
CDIP1 0.596438351 3.856601457 5.849346621 1.09E-08 2.23E-06 9.54118359
SPATC1L 0.596220987 4.730193306 4.257992873 2.62E-05 0.000693178 2.255039687
THEMIS2 0.595852087 4.839393315 4.1164674654.75E-05 0.001069357 1.705830313
ALDOC0.595590036 3.605934738 3.701933641 0.000246648 0.003466783 0.195045557
CILP2 0.595554605 3.61903921 3.720196912 0.000230079 0.003299833 0.258497492
JARID2 0.594781424 3.62319469 5.850170413 1.08E-08 2.23E-06 9.54545888
IGFBP5 0.593748692 6.896591671 3.204222571 0.001472324 0.012360798 -1.421739701
PAM 0.591342628 5.181361913 4.548529127 7.34E-06 0.000285512 3.434776967
HSPB6 0.59052585 4.9711733763.292265476 0.001089913 0.010001449 -1.151656213
C1QTNF4 0.589349625 2.202571801 3.900339174 0.000114152 0.002042871 0.899790562
SDK2 0.588577056 1.703341413 3.78849164 0.000176956 0.00276331 0.498330741

SMTNL2 0.587948697 1.015577341 4.215787036 3.13E-05 0.000782682 2.089494675

FBN2 0.587552382 1.542683709 3.678188221 0.000269874 0.003700022 0.112979559

PLEKHG2 0.5868331124.224903692 4.143500962 4.25E-05 0.000973018 1.809436453

PLD3 0.585546572 7.342922359 5.305733759 1.94E-07 2.20E-05 6.830077646

NANOS1 0.585445879 2.099945256 5.036122126 7.45E-07 5.45E-05 5.569097932

RYR1 0.581252346 2.242287612 4.280592042 2.38E-05 0.000645954 2.344293274

PLIN2 0.580941489 4.664819357 4.1865911473.55E-05 0.000861251 1.975852386

BHMT2 0.578704023 1.07194211 4.841998559 1.89E-06 0.000105346 4.696529807

LRRN2 0.577754492 5.163678899 3.750517603 0.000204865 0.003049043 0.364478222

COL26A1 0.576597652 2.812532331 2.575500412 0.01039853 0.04877571 -3.147518594

TYRP1 0.576099872 0.869690699 4.722845901 3.31E-06 0.000160159 4.175844702

SALL1 0.575650798 1.071432535 3.533894784 0.000461563 0.005470454 -0.375156137

EFNA3 0.572944977 3.67317539 5.130278887 4.69E-07 4.12E-05 6.003036019

RASL11B 0.570729257 2.369744534 4.088224459 5.34E-05 0.0011641811.598247593

RDH10 0.568656725 3.11602224 4.053953536 6.15E-05 0.001291222 1.468609467

PNMA3 0.56739288 2.612512014 3.523669489 0.000479135 0.005635058 -0.409057517

EDN3 0.566912968 1.049271265 3.142868824 0.001808523

0.01425275 -1.605863121

LRRC73 0.566782631 2.300022324 4.612056599 5.51E-06 0.000230855 3.701987426

MROH2A 0.565178322 0.725039226 5.931258466 6.93E-09 1.79E-06 9.968709921

SLC29A4 0.564464722 2.198520377 3.961563163 8.94E-05 0.001695979 1.124086486

CKB 0.563514433 6.924438028 3.490055464 0.000541416 0.006146518 -0.519856802

RAP1GAP2 0.563279706 3.038193388 4.28982377 2.29E-05 0.000629355 2.380875875

ATP8B2 0.562733985 3.312126082 4.858162105 1.76E-06 0.000100338 4.768039846

SCEL 0.560696788 2.222482006 3.346164532 0.000903687 0.008844432 -0.982914423

METTL24 0.560143144 2.120724241 3.576557818 0.000394561 0.004854147 -0.232721707

FHL1 0.560088694 3.920591217 3.761120334 0.000196681 0.002954414 0.401725868

RHOBTB3 0.560001622 2.595185527 4.241296605 2.81E-05 0.000729555 2.189373266

ACKR3 0.559995307 4.739854401 3.528305673 0.000471092 0.0055549 -0.393697864

ZNF750 0.55912494 1.679360711 3.590413759 0.000374841 0.004690009 -0.186120068

KIAA1324L 0.559012645 1.840360009 4.659682008 4.43E-06 0.000198034 3.904466441

F2RL3 0.558955162 1.602760487 5.239625767 2.71E-07 2.83E-05 6.515664937

SCUBE3 0.558617899 1.786647677 3.617905718 0.000338423 0.004365492 -0.093160407

TDRD5 0.558377605 1.61437032 5.477177782 8.02E-08 1.11E-05 7.661086903

BMF 0.557434625 3.972754592 4.262606564 2.57E-05
0.000684909 2.273226431
KCP 0.557104719 1.461216361 4.3423864 1.82E-05 0.000534525
2.590518882
GAREM2 0.556054536 2.534573612 4.533869414 7.84E-06
0.000296242 3.373583343
DNMT3A 0.55598134 3.903087509 5.527962488 6.14E-08 8.72E-06
7.911536012
CXXC4 0.554149518 0.829021598 5.593717133 4.34E-08 6.62E-06
8.238698053
ZBTB12 0.553955091 4.463877099 4.506397895 8.87E-06
0.000320918 3.259383944
LEFTY2 0.553909409 1.012968167 4.195472177 3.42E-05
0.000841035 2.010345265
AREG 0.55338165 2.0468623113.190930572 0.00153983 0.012753909 -
1.461914564
PTGER1 0.553282698 2.672272387 3.072616332 0.002279736
0.016871715 -1.812552785
FOXP4 0.55248434 6.478061217 4.35746948 1.71E-05 0.000506183
2.65110107
POPDC3 0.552340892 0.827588903 4.201864842 3.32E-05
0.000822442 2.035214611
VSTM2B 0.551288648 0.718293601 3.584326368 0.000383388
0.004767449 -0.206614418
DKK3 0.550674947 3.93668849 4.072727978 5.69E-05 0.001217868
1.539505216
CDH18 0.55018424 0.892152288 3.648658048 0.000301634
0.004013021 0.011604181
SYCP2 0.550183857 1.1011637 4.899257635 1.44E-06 8.76E-05
4.950791891
XXYLT1 0.550047838 4.320986708 5.460225161 8.76E-08 1.20E-
05 7.577918057
IGF2BP2 0.54974637 5.412476077 3.158436143 0.001717089

0.013742927 -1.559463996
ATP7B 0.549380207 2.865707873 4.536130738 7.76E-06
0.000296171 3.383011247
VAX2 0.549141971 3.175996805 3.30390071 0.001046927
0.009778732 -1.115447871
RAB11FIP4 0.548699959 3.410467647 4.622094288 5.26E-06
0.000223437 3.74450919
IQCA1 0.548219782 3.075397162 4.425944352 1.27E-05
0.000414761 2.928505898
TNNC2 0.547768626 1.530252472 4.93609219 1.21E-06 7.62E-05
5.115737995
NCAM1 0.545790541 2.337562043 3.1193198540.001955404
0.015078421 -1.675639566
SARS2 0.545077719 2.743953701 5.352245245 1.53E-07 1.85E-05
7.053303979
GPAT2 0.5448745 0.963842512 3.686030157 0.000261985
0.003613295 0.140027779
CPNE1 0.544528699 7.012090731 5.629740078 3.58E-08 5.74E-06
8.419305126
CPLX2 0.542894931 1.361261557 2.741773968 0.006408791
0.03468766 -2.72605097
LAMA10.542453023 1.35520575 4.09711426 5.15E-05 0.001137589
1.632037766
DYRK1B 0.542041048 6.121862849 5.156070201 4.12E-07 3.88E-
05 6.123110065
PIANP 0.541336924 1.880195338 2.878502193 0.004228672
0.025942909 -2.360553899
MDK 0.539306416 9.721649409 3.677305097 0.000270777
0.0037061120.109936857
LRP2 0.539180121 1.136075549 3.451099666 0.000623043
0.006778469 -0.647022304
NYAP1 0.538914804 1.627974758 5.04554345 7.12E-07 5.28E-05
5.612204497

KIRREL2	0.537610103	0.999717429	3.618178625	0.000338079	0.004365073	-0.092234309
PKHD1	0.53728913	0.58680662	6.667227396	9.54E-11	7.35E-08	14.02365494
TMEM45A	0.536741814	2.819114369	4.431014961	1.24E-05	0.000408087	2.949202107
SLC4A3	0.535781113	3.559858657	4.508158255	8.80E-06	0.000319784	3.266683214
MAG	0.534951781	2.139998661	3.035795099	0.002569561	0.018393961	-1.919114306
LYPD3	0.534703671	2.888715111	3.356572806	0.000871326	0.008621595	-0.950032683
PLPP3	0.534473089	4.685982269	4.02427562	6.94E-05	0.00140834	1.357149786
OLFM2	0.533754854	4.332052357	2.817448891	0.005101479	0.029512538	-2.525861656
RASSF10	0.53295587	3.786031207	2.666066259	0.008012381	0.040520257	-2.921095119
CAVIN2	0.531775439	2.117403965	4.233159382	2.91E-05	0.000746347	2.157454097
ZNF423	0.530450049	2.441772383	3.260203849	0.001216991	0.010776397	-1.250808287
KAZALD1	0.529608577	2.840322562	2.997375268	0.002907718	0.019983462	-2.029001104
PABPC1L	0.52920839	4.388997953	3.835495452	0.00014737	0.002426682	0.665733904
CLEC18B	0.528011657	1.272274599	3.687602901	0.000260429	0.003604127	0.145458877
CHRNE	0.527969761	1.492219869	6.312979184	7.87E-10	4.11E-07	12.02462073
TRIB3	0.527938397	4.165943591	4.002615892	7.58E-05	0.001500571	1.276275695
ELOVL3	0.527745171	1.995729538	3.772901844	0.000187949		

0.002880873	0.443228505
ASTL	0.527380048	1.199471336	5.090976629	5.69E-07	4.72E-05	5.821059683
FADS1	0.524845654	3.233647502	3.973644634	8.52E-05	0.001648048	1.168725398
IGDCC3	0.5233541160.582430737	3.853846352	0.000137143	0.00231993	0.731606227
ZFP42	0.522757007	1.026690307	3.125408774	0.001916407	0.014862717	-1.657645496
ADCY5	0.522356248	2.424616571	4.046298879	6.34E-05	0.001313349	1.439789794
COX6A2	0.522304257	0.9613381124.627381425	5.14E-06	0.000219765	3.766939583
IGF2BP1	0.521923839	0.634875829	4.366705606	1.64E-05	0.000493495	2.688291792
MUC4	0.521795302	1.279622463	3.721865043	0.000228619	0.003284424	0.264307472
GATA2	0.521543904	1.659146246	3.249765045	0.001261245	0.01104306	-1.282893511
SPSB4	0.520989047	0.941053053	4.038742226	6.54E-05	0.0013411791.411387848
SLC5A12	0.520860939	0.580699155	6.2173551181.37E-09	6.33E-07	11.49989139
UGT2B17	0.520386914	0.6987811343.313352232	0.001013174	0.009546376	-1.085946703
MIXL1	0.520023541	0.977548823	6.061379024	3.34E-09	1.10E-06	10.65784864
NREP	0.519430933	4.825470616	3.942244672	9.66E-05	0.00180466	1.05296678
CDK6	0.518374674	2.539190636	3.725927737	0.000225099	0.003254387	0.278467616
ADGRL2	0.516916127	4.5115395443.247414066	0.001271415	0.011094364-1.290106215

EGFLAM 0.51683092 2.465432847 4.655679275 4.51E-06 0.000198954 3.887377932
SRSF12 0.516173108 1.639754963 4.858296238 1.75E-06 0.000100338 4.768634144
MYT1 0.516051082 0.841753293 5.121553906 4.89E-07 4.21E-05 5.962533525
TBX1 0.515928488 1.354191019 3.351052213 0.000888353 0.008726036 -0.96748524
ZCCHC12 0.515879182 1.145058003 3.298662196 0.001066082 0.009881821 -1.131764772
COX8C 0.51586917 1.033259523 2.602004302 0.009641797 0.046272616 -3.082035794
KIF3C 0.515389399 3.440326691 4.596492401 5.91E-06 0.000245856 3.636216459
FAM229B 0.515228357 3.449280169 5.228949535 2.86E-07 2.95E-05 6.465204841
GPR1580.5141275 1.032128803 4.721905699 3.33E-06 0.000160159 4.171781548
TCEAL2 0.514044603 2.352915915 2.6861106880.007555997 0.03889307 -2.869965439
KDM5B 0.513086221 4.46652896 5.143683409 4.39E-07 3.99E-05 6.065377357
PLAC1 0.513003583 0.992435061 3.43587714 0.000657957 0.00699831 -0.696351226
ANKRD53 0.512686262 1.494586783 5.0586791156.67E-07 5.14E-05 5.672422262
FXYD6 0.512662064 3.60789424 3.238589643 0.001310271 0.011347735-1.317135275
BVES 0.5117521761.098239506 4.878798547 1.59E-06 9.32E-05 4.859641891
NAT8L 0.5113528394.007300381 3.232450647 0.001337949 0.011507357-1.335898008
RASD1 0.510420005 3.213647992 3.044044407 0.002501834

0.018052932 -1.895346614
SYT1 0.507239552 1.061509342 5.17721983 3.71E-07 3.64E-05 6.221961098
GALNT13 0.505506056 0.920703133 4.848391656 1.84E-06 0.00010327 4.724788899
FGFR3 0.504912845 2.712610439 2.574934168 0.010415261 0.048840027 -3.148910558
NETO2 0.50347865 1.993816452 4.359230695 1.70E-05 0.000504172 2.658187417
MARCKSL1 0.501748839 9.157851851 4.696714866 3.74E-06 0.000175238 4.063183417
TMEM59L 0.501585174 1.216558431 3.56610724 0.000410073 0.005010085 -0.267759133
KLHL35 0.501265307 2.504942855 4.1107476854.87E-05 0.0010871161.683988227
IGSF11 0.5011265121.375176457 4.6633939114.36E-06 0.000196317 3.92032495
FADS2 0.501074269 4.255794512 2.988091316 0.00299533 0.020386393 -2.055355058
FGFR4 0.500336621 2.572999823 4.033818206 6.67E-05 0.001363205 1.392906815
TCP11 0.500073036 0.954804343 5.191225451 3.46E-07 3.43E-05 6.28761334
ABHD8 0.498838078 5.367815923 4.946180647 1.15E-06 7.37E-05 5.161102352
PLCG1 0.498216362 3.986942358 4.4006114941.42E-05 0.000449868 2.825425382
LEFTY1 0.498039164 0.861629191 3.698909205 0.000249498 0.003494528 0.184565686
CYP2W1 0.4971105641.029106939 3.2056311380.001465333 0.012314888 -1.417473089
WNT5A0.496927987 2.935403976 3.766781782 0.000192438 0.002923253 0.421654446

TBC1D32 0.496416581 1.666828862 7.494408043 5.00E-13 1.62E-09 19.01409009

DCAF12L2 0.496271578 1.1110567344.542682297 7.54E-06 0.000289635 3.410349623

PADI3 0.495344263 0.721314431 3.492038361 0.000537541 0.006106977 -0.513348254

PALM 0.495154954 4.67217316 3.2210419 0.001390829 0.011855019-1.370677596

NLRP1 0.49491629 1.857345568 4.852022844 1.81E-06 0.000101862 4.740854211

TSPYL4 0.493497239 4.548503482 4.66090344 4.41E-06 0.000198022 3.909683581

CENPV 0.492091731 3.1126582293.357152504 0.000869555 0.008614616 -0.948198485

MAPK8IP1 0.491777942 5.303616556 3.471818506 0.000578297 0.006438878 -0.579554779

FSTL4 0.491423125 0.95810893 4.481539707 9.91E-06 0.000347752 3.156582012

CRLF1 0.49140143 1.677896019 3.304139281 0.001046062 0.009778732 -1.114704191

GRASP 0.490800632 3.205229638 4.534154659 7.83E-06 0.000296242 3.374772357

BMI1 0.490643289 5.000086201 5.691800225 2.57E-08 4.37E-06 8.732725408

HSPA12B 0.490613179 2.2011065144.509982326 8.73E-06 0.000318674 3.274249349

COL11A2 0.489996453 1.239001408 4.046330007 6.34E-05 0.001313349 1.439906888

STK31 0.489844901 0.657621368 6.155305164 1.96E-09 7.57E-07 11.16284243

ADGRL3 0.4898111520.782566328 5.13950859 4.48E-07 4.04E-05 6.045946202

FBXO17 0.489332462 3.34154292 2.960560083 0.003269551

0.021607351	-2.13304936
CPE	0.488243855	5.720880688	3.387935941	0.000780218	0.007947678	-0.850371081
GPC2	0.487380997	1.512339855	4.182179081	3.61E-05	0.000869696	1.958741072
NKAIN1	0.485338111 1.009438443	4.174938995	3.72E-05	0.000888421	1.930697289
IQCG	0.485218529	3.056007311 4.856081686	1.77E-06	0.000100622	4.758824039
COL14A1	0.484632557	2.699268876	3.188830347	0.001550754	0.012813126	-1.468248032
BHLHA15	0.482868753	2.21348348 3.642036928	0.000309222	0.004080417	-0.011021672
MKRN3	0.482508327	0.623440467	5.147917222	4.29E-07	3.95E-05	6.085096978
GATM	0.482298021	2.604685289	3.824246956	0.000153992	0.002508841	0.625499468
HIST1H3I	0.482186419	1.83093602 3.030537421	0.0026136	0.01857769	-1.934230628
EVA1B	0.481923715	5.912294439	4.556470123	7.08E-06	0.000278837	3.467998279
TCTN2	0.481812535	3.947500558	4.591313554	6.05E-06	0.000248503	3.614375538
BEND5	0.481640968	2.585409708	4.117858219 4.72E-05	0.00106586	1.71114535
NELL2	0.48159183 1.46991433 4.019706911 7.07E-05	0.001424033	1.34005775
DUSP9	0.480234653	2.351353488	3.014339474	0.002753669	0.019285221	-1.980644772
SELENOV	0.480179518	0.602046006	4.222700894	3.04E-05	0.000768425	2.116510922
WASF1	0.480084717	4.0680407	3.846022637	0.000141418	0.002363497	0.703486857

CES1 0.479707005 1.240880945 3.610525972 0.000347858
0.00444171 -0.118178562
LMO1 0.478865229 1.417522816 3.67518903 0.00027295 0.003720125
0.102648938
TBKBP1 0.478476931 3.339918812 4.741707479 3.03E-06
0.000150165 4.257507087
DLX6 0.478134296 0.948935485 3.079429584 0.002229524
0.016612085 -1.79270145
LY6K 0.477103217 1.42857434 2.7319113570.006599815 0.035401791
-2.751756645
STUM 0.477002098 0.825745717 5.161035663 4.02E-07 3.87E-05
6.1462868
LAMA20.4768295111.554651264 4.616056869 5.41E-06 0.000227854
3.718923583
NTN3 0.476691298 0.974595042 4.596596487 5.91E-06
0.000245856 3.636655651
PLEKHG6 0.475468037 3.062971568 3.820514321 0.00015625
0.002537971 0.612172365
CACNA1A 0.473380161 2.22628418 3.91248318 0.000108778
0.001981768 0.944025708
KCNIP3 0.47254536 2.337896519 3.969449762 8.66E-05
0.001662194 1.153211981
TAS1R30.472077787 1.347949214 3.75013093 0.00020517 0.003050768
0.363121668
CCDC184 0.471743761 1.671096302 4.460136101 1.09E-05
0.000372362 3.068473715
CYTL1 0.471468543 2.4114439032.613784121 0.009321578
0.045286635 -3.052724352
NGEF 0.471024335 3.122570812 2.970772361 0.00316526
0.021142684-2.104309735
SALL2 0.470151373 3.791756477 3.327761809 0.000963656
0.009248456 -1.040817409
CHRNB1 0.469969437 2.514042952 5.158461668 4.08E-07 3.88E-

05 6.13427005
M1AP 0.46991485 3.068808892 3.226561251 0.001365009
0.011678012-1.353866328
SLC16A8 0.46977021 1.684977566 3.853306263 0.000137434
0.002320883 0.72966339
RB1 0.469428484 4.385662988 4.557719437 7.04E-06
0.000278631 3.473229502
COX4I20.469238515 2.609195284 4.605993746 5.66E-06
0.000236693 3.676343644
SLC6A13 0.468197865 1.315512556 3.093868447 0.00212646
0.016053137 -1.750494078
AC005041.1 0.467908134 3.719615218 4.189706462 3.50E-05
0.000856154 1.987944364
PLAG1 0.467581753 2.303400836 3.7511729560.00020435 0.003046984
0.366777677
KSR1 0.466945054 2.08371967 4.772195351 2.63E-06 0.00013646
4.390112478
PLAGL1 0.466520074 2.263366035 3.217353724 0.001408333
0.011947569-1.381896193
ENAH 0.465614263 4.588530353 5.067965955 6.38E-07 5.03E-05
5.71507768
AVPR2 0.465403376 1.5324203113.220625586 0.001392794
0.011865523-1.371944537
MEST 0.4649235 6.373603657 3.058059802 0.002390517
0.017491532 -1.854825451
ETNK2 0.463320088 3.523817866 4.324660275 1.97E-05
0.000563821 2.519562322
CDH3 0.463141214 4.725235855 2.788449926 0.005570785
0.031522964 -2.603191829
FGF12 0.463065956 1.710831226 4.222737546 3.04E-05
0.000768425 2.11665425
SLC29A1 0.462697652 5.602438791 4.19875945 3.37E-05
0.000831297 2.023129441

KCNK9 0.462001352 1.102172569 3.537189294 0.000456032 0.005420778 -0.364213869
PKIG 0.461837204 5.495922846 5.496453873 7.25E-08 1.02E-05 7.755919059
FAM90A1 0.461458537 2.916870478 4.213846455 3.16E-05 0.000786264 2.081918962
MLF1 0.461055932 5.318176432 4.164485117 3.89E-05 0.000916578 1.89028294
ANGPTL1 0.46076191 1.563773593 3.226717959 0.001364283 0.011677972 -1.353388617
SLCO1A2 0.460479794 0.605105552 5.830012182 1.21E-08 2.38E-06 9.440984936
RHCG 0.460466234 1.111544163 3.825342536 0.000153335 0.002500655 0.629413434
PREP 0.459828606 4.628435805 5.301415344 1.98E-07 2.21E-05 6.809436236
EIF4G3 0.458870323 4.371221614 4.695084138 3.77E-06 0.000175238 4.056171
TNFRSF13C 0.458609808 1.404455636 5.021643193 8.00E-07 5.66E-05 5.502986858
ANKRD45 0.457757184 2.141018918 3.630563473 0.000322797 0.004197922 -0.050138699
GRIN2D 0.457572263 2.167579304 3.18990783 0.001545141 0.012786335 -1.46499925
STEAP1B 0.456959234 0.881465276 4.878824003 1.59E-06 9.32E-05 4.859755096
LRRC4B 0.456886904 1.639791877 3.501840599 0.000518765 0.005941417 -0.481123242
SEMA6A 0.456718974 1.787131584 3.590495349 0.000374728 0.004690009 -0.185845162
SBK2 0.456608367 1.049152662 2.811998231 0.005186853 0.029904814 -2.540455094
TMEM37 0.456309362 3.225011344 3.527800337 0.000471962

0.005561112-0.395372956

CCL28 0.455576383 4.19578547 2.948874373 0.003392739 0.022185517 -2.165819942

NEBL 0.453273594 1.537595356 4.133387733 4.43E-05 0.001008157 1.770605262

TMEM132B0.453023084 0.790222967 4.5110209658.69E-06 0.000317917 3.278558778

LRRC43 0.451910256 1.609043063 3.921780734 0.000104826 0.001927136 0.977977803

VANGL2 0.451709375 4.979637668 3.4171131060.000703508 0.007357047 -0.756875566

TPST1 0.451462805 4.062722294 5.157323217 4.10E-07 3.88E-05 6.128956816

DLK2 0.45123019 1.793609915 4.639673458 4.86E-06 0.000210071 3.819175726

PRTFDC1 0.45100522 3.7027051 3.094784738 0.00212007 0.016019848 -1.747809281

AGPAT4 0.450710853 1.921991746 5.081486937 5.97E-07 4.87E-05 5.777301982

EPB41L5 0.450446149 3.334336472 4.399754542 1.42E-05 0.000450674 2.821947682

PLA2G4A 0.450313068 3.043054063 3.158675391 0.001715718 0.013741029 -1.558749214

MS4A15 0.450274001 1.441250032 3.206241559 0.001462313 0.012295894 -1.415623545

N4BP3 0.449787602 2.791252574 3.945523371 9.53E-05 0.001787326 1.065014643

ENTPD7 0.449774327 2.732362596 4.366224487 1.64E-05 0.000493612 2.686352743

SDK1 0.449665812 2.249055323 3.000329657 0.002880335 0.019854305 -2.0205983

KIF5A 0.448419577 1.71641762 3.302330294 0.001052635 0.009792718 -1.120341953

AGMAT 0.44825911 2.188955933 4.546603812 7.41E-06
0.000287304 3.426730147
CHD7 0.447779478 3.314972529 4.355804593 1.72E-05
0.000508923 2.644404665
NR2F1 0.446555923 3.351383955 2.874674285 0.004279117
0.026153214 -2.371017795
EMILIN3 0.446174276 0.868146088 3.729136939 0.000222356
0.003234956 0.2896631
HEYL 0.446153177 3.05349174 3.561440313 0.000417184
0.005078404 -0.283375002
XCL1 0.445926363 1.715077748 3.40305186 0.000739553
0.007620695 -0.80202732
C17orf107 0.445723159 1.792674801 4.912955373 1.35E-06 8.28E-05 5.012004754
ALDH4A1 0.445473022 3.222189597 4.75494831 2.85E-06
0.000144647 4.315005442
IGFALS0.445465914 1.44188674 3.641281289 0.000310099
0.004088659 -0.013601435
AKR1E2 0.445179508 1.541902464 3.605396151 0.000354562
0.004513058 -0.135541248
CGB2 0.445100907 0.565762387 4.083631057 5.44E-05
0.0011800611.580814237
UPK1A 0.444762176 1.031490972 2.857290856 0.004515166
0.027124528 -2.418369488
ZNF703 0.443925799 5.634741254 2.675879961 0.007785928
0.039700488 -2.896108127
C4orf54 0.443729995 0.590035919 5.132562972 4.64E-07 4.10E-05
6.01364886
FLRT3 0.443146132 2.204475427 2.935358549 0.003540503
0.022892988 -2.203568789
HSD17B14 0.442301374 5.31338981 3.256637167 0.001231947
0.010867194 -1.26178192
NRXN2 0.442122899 1.908758255 2.854510458 0.004554001

0.027266702	-2.425917719
RINL	0.441659607	2.656775437	4.096081941	5.17E-05	0.0011399961.628110488
KIAA1549	0.441639094	2.250859596	3.843995632	0.000142546	0.00237499	0.696210138
DENND5B	0.441559959	1.914831375	5.022220287	7.97E-07	5.66E-05	5.505618719
PDRG1	0.441049143	5.22943184	5.802947716	1.41E-08	2.71E-06	9.301186496
DSC2	0.440970215	3.994206397	2.906052774	0.003881285	0.024461014	-2.284850187
SLC22A17	0.440139155	3.523354507	2.800821495	0.005365981	0.030740385	-2.570294793
CIB2	0.4401183794.056140471	3.425340542	0.000683185	0.007195677	-0.73037576
ADAMTS7	0.439483593	2.1595787113.292894914	0.001087546	0.009991097	-1.149700503
IL11RA	0.439003392	3.021447256	3.061753444	0.002361948	0.017337381	-1.844117021
LLGL1	0.43887637	4.130082544	4.492733449	9.42E-06	0.000337302	3.202811299
CDO1	0.438801893	1.218952744	3.57273219	0.000400174	0.004904561	-0.245558882
BTBD3	0.438547625	4.2611591674.520101623	8.34E-06	0.000308761	3.316273138
DNAH2	0.43820829	1.045607834	4.571728369	6.61E-06	0.000264152	3.531976143
ACRBP	0.438019289	2.321781592	4.214355391	3.15E-05	0.000786219	2.083905458
TSPAN18	0.437510181	2.836706947	3.039346445	0.0025402	0.018264606	-1.908889766
PACRG	0.437420906	1.574386085	3.756001413	0.000200593	0.002999254	0.383730846

P3H3 0.436413644 3.448639831 2.888572492 0.004098538 0.025453417 -2.332962448
LDHC 0.436146846 1.108627059 4.238171213 2.85E-05 0.000734548 2.17710701
MLLT11 0.436062408 2.958106165 3.760784445 0.000196935 0.002955493 0.400544391
C4orf48 0.436061653 6.425870412 2.99049668 0.002972402 0.020290093 -2.048534517
LYRM4 0.435030684 3.603894803 5.138684003 4.50E-07 4.04E-05 6.042109878
DLL1 0.435005721 1.872431434 3.347292405 0.000900127 0.008814923 -0.979355887
SMAGP 0.434926855 3.417803174 3.539443438 0.000452283 0.005384126 -0.356721557
FAM133A 0.434914276 0.603626784 3.608398128 0.000350624 0.004469973 -0.12538337
RBPJL 0.434613157 0.463524733 4.518038637 8.42E-06 0.00031007 3.307699077
PACC1 0.434569056 3.327937924 4.916730122 1.33E-06 8.17E-05 5.028899693
ACAA2 0.434217571 4.438137168 5.06277423 6.54E-07 5.09E-05 5.6912232
TUBA1A 0.433509561 7.157518672 3.04757881 0.002473322 0.01789508 -1.885144645
TREML2 0.433398815 0.549394087 4.827202664 2.03E-06 0.0001103234.631253634
ZNF219 0.432678512 4.3981186813.312203623 0.001017221 0.009567793 -1.089536104
ALX1 0.431988196 0.669188321 3.448784994 0.000628238 0.006802963 -0.6545362
TNNI1 0.431517957 1.626188236 3.728481496 0.000222914 0.003238816 0.287375823
CCNJL 0.430781491 2.29706106 3.747510951 0.000207244

0.003073137	0.353933468
MATN4 0.430517132	0.746885355	4.397091415	1.44E-05	0.000454176	2.811144011
FSD1	0.430357836	1.379320247	3.056156836	0.002405359	0.017544694	-1.860337652
CCNJ	0.429960038	2.652982318	5.007461027	8.57E-07	5.90E-05	5.43839099
KIF17	0.429924005	1.277660641	5.392688188	1.24E-07	1.62E-05	7.248752156
ARG2	0.429767364	2.124121553	4.35778812 1.71E-05	0.000506183	2.652382944
KDM1A	0.42931933 5.792656497	5.145183498	4.35E-07	3.98E-05	6.072362649
SUGCT 0.429048798	2.191254603	3.455171684	0.000614001	0.006711692-0.633792259
SGCG	0.428445427	1.407327973	2.998727494	0.002895155	0.019930989	-2.025156113
HDAC2 0.428419933	4.350159421	5.074204886	6.18E-07	4.98E-05	5.743771759
MFSD4B	0.428261913	2.189494562	5.555298081	5.32E-08	7.75E-06	8.047149402
RAP1GAP	0.427344883	4.47111158 3.02888931 0.002627547	0.018654636	-1.938963981
ZNF521 0.426917387	2.285296027	2.584718244	0.010129529	0.047874824	-3.124817463
IGSF3	0.426489139	2.824700279	3.730587287	0.000221126	0.003219963	0.294725644
SEMA4G	0.426467946	2.5116821953.895937305	0.000116162	0.002065125	0.883787712
VCX	0.425887885	0.488856806	4.957477515	1.09E-06	7.09E-05	5.211996304
TMEM108	0.425712327	1.727863801	3.412393979	0.000715418	0.007428779	-0.772048497

C11orf95 0.425531628 3.76780237 4.584188498 6.25E-06
0.000252804 3.584362554
SELENOP 0.425316905 5.406497541 2.825304844 0.004980674
0.029028387 -2.504780805
LRRTM4 0.425293528 0.457543251 5.753739743 1.84E-08 3.30E-06 9.048388079
FBXO36 0.42523885 2.345291704 5.078322638 6.06E-07 4.90E-05
5.762726846
UBTD1 0.425199921 5.862989528 4.695946045 3.75E-06
0.000175238 4.059877081
HIST1H1B 0.424637998 1.891060661 2.724701473 0.006742696
0.035989222 -2.770492099
C19orf12 0.424022037 4.349236926 4.06666968 5.83E-05
0.001243612 1.51659534
ADAMTS5 0.424000528 1.509875711 3.617725183 0.000338651
0.004365492 -0.093773007
RASL12 0.423881807 2.520565635 3.960478497 8.98E-05
0.001701034 1.120084925
CHRM3 0.42360762 0.777742145 4.857370851 1.76E-06 0.000100361
4.764534353
SOX12 0.422641858 6.505955159 4.18806728 3.52E-05 0.000857239
1.981580924
ASCL2 0.422449692 2.894521255 2.641982877 0.008593406
0.04263173 -2.982040591
CDK19 0.422337629 3.35981833 4.973505596 1.01E-06 6.67E-05
5.284378495
ARHGAP40 0.421247183 1.452727843 3.271535671 0.001170584
0.010503444 -1.215868504
NRCAM 0.421196145 3.2017971 3.051033249 0.002445743
0.017767056 -1.875162633
BLMH 0.421079581 3.451604311 5.223557008 2.94E-07 3.01E-05
6.439751172
SPEF1 0.420763229 2.536492444 2.693258003 0.007399011

0.038390376	-2.851644981
SLC2A1	0.420583456	7.369711189	3.29301816	0.001087083	0.009991097	-1.149317529
MGAT3	0.420113689	3.516908394	2.586722702	0.010071864	0.047713795	-3.119870659
PPP1R1C	0.420061464	0.850317927	3.768494726	0.000191172	0.002913914	0.427689551
DLX4	0.419948566	1.347598059	4.143700351	4.24E-05	0.000973018	1.810202902
LNP1	0.419833188	2.709096391	4.273904344	2.45E-05	0.000660178	2.317836323
EDNRB	0.41982696	1.846483926	3.453118384	0.000618545	0.00674569	-0.640465283
LGSN	0.419440365	0.661077493	4.157939599	4.00E-05	0.000934606	1.865024984
EFNA4	0.419427343	5.466022737	4.990799378	9.29E-07	6.21E-05	5.362704504
KDM6B	0.419416974	4.499504274	4.08235755	5.47E-05	0.001184677	1.575984052
STMN1	0.419413809	6.651957233	3.718635113	0.000231453	0.003308819	0.253060046
TET3	0.418843289	3.190963352	4.163990213	3.90E-05	0.000916578	1.888371939
DDAH2	0.418801012	7.048938275	4.387908319	1.50E-05	0.000465268	2.773935395
CAVIN4	0.417970174	1.01599768	5.7626985	1.75E-08	3.18E-06	9.094279474
DBN1	0.417901499	6.617252476	4.584200525	6.25E-06	0.000252804	3.584413181
TRDN	0.417511719	0.410778582	6.096505357	2.74E-09	9.62E-07	10.84596875
SMPDL3B	0.417345218	5.635380261	3.863144811	0.00013222	0.002258191	0.765094368

FYN 0.417153794 3.930100577 3.261425647 0.001211906
0.010760824 -1.24704657
TBX3 0.416795542 2.183307701 2.749205646 0.006268166
0.034137778 -2.706622545
ZNF853 0.416294661 4.0726246113.479403731 0.000562681
0.006312792 -0.554760334
SLC6A8 0.4161108664.82577684 2.718395842 0.006869939
0.036475836 -2.786838848
MYL7 0.415276691 0.459613851 4.232919239 2.92E-05
0.000746347 2.156512947
ARHGAP4 0.414816365 4.505701708 3.5119913150.00049997
0.005781636 -0.447663729
CCDC77 0.414531366 3.134735554 5.234236255 2.79E-07 2.89E-
05 6.490180946
NDRG3 0.414342023 5.622558783 5.258748981 2.46E-07 2.64E-05
6.606269168
ERGIC3 0.414284416 7.500134888 6.5131185022.42E-10 1.63E-07
13.14345607
ZNF608 0.413556799 3.458439025 3.667379612 0.000281115
0.003802574 0.075786299
DHX35 0.413553633 3.386876945 5.61966259 3.78E-08 5.94E-06
8.368682167
MACROD2 0.412867052 2.157500939 3.018689671 0.00271539
0.01907494 -1.968202787
APPL2 0.412733238 4.369837713 4.990784103 9.29E-07 6.21E-05
5.362635221
C20orf96 0.412476362 5.2128129 3.831916131 0.000149448
0.002449544 0.652919321
FRAT1 0.41239564 2.83643891 5.023229669 7.93E-07 5.66E-05
5.510222682
FAM131C 0.4117087062.286640161 3.613937697 0.000343466
0.004399516 -0.106618336
SH3PXD2A 0.4116784533.829289297 3.213886834 0.001424973

0.01206343 -1.392430627
ERBB4 0.4116201962.634257251 2.695020064 0.007360762
0.038265556 -2.84712117
PBXIP1 0.4112059776.089695031 4.569040068 6.69E-06 0.00026672
3.520690287
JPH4 0.410426553 1.1142550483.355785487 0.000873735
0.008627549 -0.952523327
EPHA4 0.410173883 2.446774214 3.1197225110.001952803
0.015065543 -1.674450654
IGSF23 0.409932558 1.389298706 2.735722279 0.006525402
0.035130764 -2.741834477
UNC119 0.409794048 4.356867329 4.745350141 2.98E-06
0.000149389 4.273311273
SKIDA1 0.409560856 1.084101894 3.57749798 0.000393193
0.004847929 -0.229564965
TLE4 0.409250132 2.377903561 3.068515076 0.00231046
0.017060077 -1.824482243
SRMS 0.40922983 1.754831075 3.283953279 0.0011216190.010194135
-1.177449789
CADM4 0.408699717 4.699797778 3.516228154 0.000492315
0.005726884 -0.433671225
LKAAEAR1 0.408480596 1.798345733 2.893667768 0.004034094
0.02517885 -2.318966946
SLC6A12 0.408315966 2.794575178 2.726149761 0.006713772
0.035858504 -2.76673242
PIGT 0.40818511 8.214746687 4.635214616 4.96E-06 0.000213823
3.800213264
STK33 0.407129075 1.744307834 4.524054903 8.19E-06
0.000304041 3.332713274
ZIC4 0.4070220110.802858433 2.933006047 0.003566815 0.0230355
-2.210122297
FAM222B 0.406851421 4.383500821 4.733852778 3.15E-06
0.00015472 4.223464792

SOCS7 0.406819546 2.665100159 4.377022048 1.57E-05
0.000477138 2.729916214
PBX2 0.406660832 6.409617154 4.275722764 2.43E-05
0.000657345 2.325026412
NLGN2 0.406492961 4.61329543 3.443337169 0.000640625
0.006884883 -0.672202338
PTH1R 0.406268484 1.180128576 3.542145443 0.000447827
0.005351904 -0.347734785
IGSF1 0.406203967 1.079307332 3.71340358 0.0002361150.003341972
0.234861711
LRRC56 0.405976599 3.128709316 3.292170565 0.00109027
0.010001449 -1.151951078
UBE4B 0.405971862 4.544929328 4.329767973 1.93E-05
0.000554465 2.539981327
SCPEP10.405183984 5.0116506943.139909727 0.001826403
0.014364632 -1.614658317
TRIM58 0.405040199 2.417005109 2.669949007 0.007922087
0.040214474 -2.911219673
APOD 0.4048821153.677055723 2.75655627 0.006131824 0.033581805
-2.687356431
MAST1 0.404292882 2.212280503 3.519876507 0.000485811
0.005680656 -0.421609632
FNDC5 0.404188283 1.309266522 4.362442132 1.67E-05 0.00049902
2.671115443
BMPR1B 0.403486689 3.137264031 3.341263071 0.000919311
0.008981043 -0.998365865
CBFA2T2 0.402714651 3.969693142 4.8220346112.08E-06
0.0001122924.608494635
F2R0.40255847 4.057272489 2.953609491 0.00334232 0.021962654 -
2.152555984
OPRL1 0.402524797 1.689340973 4.507692726 8.82E-06
0.000319784 3.264752669
MYOM3 0.402003526 0.578189283 4.390190021 1.48E-05

0.000461776	2.783174015
INHBE	0.401758877	0.624526818	4.615502141	5.42E-06	0.000227854	3.716574222
OTUD3	0.401694143	2.000320032	4.898912606	1.45E-06	8.76E-05	4.949251945
SLC2A12	0.401478463	2.295476796	3.088500901	0.00216425	0.016239907	-1.766206231
PGF	0.40107612	1.819477565	3.838866057	0.000145439	0.002405812	0.677811314
TMEM44	0.400986386	2.859993682	4.347626621	1.78E-05	0.000524426	2.61154516
RIC3	0.40077674	1.653704998	2.900061624	0.003954533	0.024807647	-2.301371261
GLMP	0.40029613	5.736939893	4.862264052	1.72E-06	9.91E-05	4.786220702
PODXL2	0.399666351	5.720175388	2.875094861	0.004273548	0.026129047	-2.369868768
CABYR	0.399417204	2.303987023	3.793994941	0.000173222	0.002726058	0.517832261
MEX3B	0.398909992	1.727479979	3.663357227	0.00028541	0.003841394	0.061970873
ASIP	0.398353689	1.269093876	4.320244069	2.01E-05	0.000572639	2.501925188
GLIS2	0.397845927	5.086561738	3.324169062	0.000975787	0.009313443	-1.052086748
ZC4H2	0.397728833	2.190958173	3.061207953	0.002366147	0.017352459	-1.845699253
SERPINI1	0.397713301	2.473836447	3.218191523	0.001404339	0.011932457-1.37934886
GDPD1	0.397080744	1.964310377	4.888408758	1.52E-06	9.04E-05	4.902416089
KCNN1	0.397018312	1.309933413	3.0114451020.0027794110.019406696	-1.988913511

KPTN 0.396946779 3.056307476 4.292887281 2.26E-05 0.000625394 2.393031369
C17orf100 0.396427815 2.838309338 4.765886925 2.71E-06 0.000138753 4.362612921
MAP3K10 0.396424659 3.324206343 3.633144804 0.000319695 0.004174356 -0.04134803
C6orf120 0.396354649 4.284548231 4.182908158 3.60E-05 0.00086946 1.961567522
FAM43B 0.396350733 0.852903051 4.420223727 1.30E-05 0.000422757 2.905182064
TCF15 0.395677226 1.893899247 3.314194239 0.001010217 0.009533493 -1.083314689
STARD3 0.395561685 4.159835662 4.564102497 6.84E-06 0.000271392 3.499977058
CNPY4 0.39497235 3.365548066 4.974333133 1.01E-06 6.67E-05 5.288121143
NPFFR1 0.394954567 1.745660251 3.067909475 0.002315029 0.017063599 -1.826242495
LFNG 0.394917944 3.697555395 3.1677845110.001664253 0.01343527 -1.531496561
PLTP 0.394696727 8.15040564 3.320064551 0.00098982 0.00941243 -1.064947325
TDRD12 0.394563396 0.563121521 2.964048043 0.003233583 0.021450483 -2.123244056
PSEN2 0.394271434 3.598786037 5.017864661 8.14E-07 5.70E-05 5.485761188
CYP11A1 0.39405117 1.677567919 2.832543881 0.004871661 0.028586772 -2.485305709
SBK1 0.393897844 4.480732079 2.661552191 0.008118519 0.040916324 -2.932558914
SLC10A4 0.393736033 0.985474183 3.6980676110.000250296 0.0034968 0.181650919
LRP1 0.393540374 4.949962458 2.849579001 0.004623629

0.027540894 -2.439288382
ZCCHC3 0.393482499 5.142557944 4.291899607 2.27E-05
0.00062673 2.389111591
ADGRL1 0.393136123 4.993412302 2.751980912 0.006216371
0.033952885 -2.69935432
THAP8 0.392945217 4.819387787 4.443053555 1.17E-05
0.000395044 2.998423853
NDRG2 0.392909604 4.076919661 2.737129313 0.00649812
0.035018847 -2.738167754
PPM1H 0.392732788 3.2583345 3.04642069 0.002482631 0.01793837 -
1.888488769
ART5 0.39251507 1.387991618 3.7907346 0.000175425 0.002747361
0.506275751
S100P 0.392226371 1.3563147 2.608754248 0.009457125
0.045643604 -3.065255657
BCAS4 0.392198334 2.641214548 4.336336225 1.87E-05
0.000543781 2.566271053
ESPNL 0.391705525 1.218586293 2.826850161 0.004957219
0.028933913 -2.500627455
RTN4RL2 0.390957042 2.126581889 2.9934361150.002944601
0.020176941 -2.040192503
DHRS13 0.390488973 2.712453069 3.864552491 0.00013149
0.00224867 0.770170557
P3H2 0.390486245 2.751225943 2.861698059 0.004454225
0.026878202 -2.406390418
TGIF2 0.390458032 5.864741421 3.99602496 7.78E-05 0.00153321
1.251745327
ADGRB2 0.390035992 2.418254144 3.079326168 0.002230279
0.016612085 -1.79300308
ANK2 0.389240606 1.198155354 3.802704517 0.000167465
0.002674241 0.548748909
C19orf47 0.38896053 3.600427206 4.165843462 3.87E-05
0.000916173 1.895529062

MAP4K2 0.387753939 3.215122182 4.626870799 5.15E-06 0.000219765 3.764772287
RASIP1 0.38771375 3.46759542 2.62531146 0.00901748 0.044194122 -3.023917796
MAFA 0.387644958 0.680652722 3.413367674 0.000712946 0.007422158 -0.768919481
IGF2R 0.387305648 3.90414382 3.501842372 0.000518762 0.005941417 -0.481117404
GPR153 0.387053824 3.92114303 2.787166504 0.005592431 0.031590207 -2.606596572
ELOVL2 0.386514284 1.047908798 3.802734806 0.000167445 0.002674241 0.54885654
PSAT1 0.386032807 7.210839276 2.834082296 0.004848775 0.02850463 -2.48116079
CRYGB 0.385699269 0.735406496 2.594650435 0.00984666 0.046966052 -3.100269614
SNCB 0.385570646 0.75276912 3.329289907 0.000958539 0.009241503 -1.03602077
HIST1H4E 0.385487503 3.287743955 2.578054688 0.010323358 0.048578034 -3.141235899
SRC 0.385373097 4.452296933 3.565935562 0.000410333 0.005010085 -0.268333918
CCR10 0.385249887 1.089615651 5.339833066 1.63E-07 1.94E-05 6.993570826
USH1C 0.384365678 0.506546743 4.479256741 1.00E-05 0.000349627 3.147166185
TEAD1 0.383710818 4.416550283 3.303317091 0.001049045 0.009778732 -1.11726693
SSC4D 0.382879361 3.017485913 3.037642439 0.002554249 0.018339773 -1.913797118
HBQ1 0.382547391 1.039575554 2.972177239 0.003151154 0.021082074 -2.100348723
CTNNA2 0.381955062 1.424396949 2.914602391 0.003778897

0.02397451 -2.261217626

C2CD6 0.381218974 0.675400931 5.539572518 5.78E-08 8.27E-06 7.969065268

MAP10 0.380770915 2.101987155 3.71962889 0.000230578 0.003301374 0.256519665

TESC 0.3807191141.731088951 3.573225423 0.000399446 0.004899351 -0.243904519

POGLUT2 0.380623437 2.984726498 3.560653476 0.000418394 0.005089306 -0.286005937

PLEKHB1 0.379983572 4.877815131 2.59292275 0.009895351 0.047153751 -3.104546179

KLHL32 0.379727547 1.251788547 4.098521342 5.12E-05 0.0011340971.637392214

LSM14B 0.379301723 5.335539842 4.758097134 2.81E-06 0.000142987 4.328700007

AGBL5 0.378991809 5.230833699 4.183194294 3.60E-05 0.00086946 1.962676924

UBE2C 0.378471527 7.468738225 3.037558438 0.002554944 0.018339773 -1.914038965

AHDC1 0.378453025 5.408835386 3.274379277 0.0011591990.01044183 -1.207082801

PEX3 0.3780042114.178036701 4.917994352 1.32E-06 8.17E-05 5.034560639

DQX1 0.377541675 0.574149361 4.701988481 3.65E-06 0.000172016 4.085875615

SLC35E2B 0.377036236 4.090482313 3.262035744 0.001209375 0.010752825 -1.245167683

TM7SF2 0.376546467 5.605563939 3.407701614 0.000727447 0.0075199 -0.787115902

TIE1 0.376425852 2.506199902 4.275863842 2.43E-05 0.000657345 2.325584354

DMKN 0.376142303 5.196607268 2.600985591 0.009669946 0.04636225 -3.08456464

B3GNT8	0.375688476	2.403576841	3.599193605	0.000362829	0.004589055	-0.156504085
NUAK1	0.374916924	2.746737158	2.720620113	0.006824809	0.036295782	-2.781076784
VTA1	0.374906211	4.320867331	5.070910471	6.28E-07	5.01E-05	5.728616285
RTL5	0.374726562	2.811601359	2.980547845	0.003068291	0.020734676	-2.076711114
PNMA8A	0.374547266	4.494935705	3.15689496	0.001725947	0.013788823	-1.564067233
THBS3	0.374201711	5.033450513	3.36976581	0.000831856	0.00832268	-0.908215681
GPR156	0.373990339	1.406768628	4.051465805	6.21E-05	0.001297906	1.459237745
MFSD13A	0.373822227	2.492801342	4.294954793	2.24E-05	0.000622806	2.401239331
DZANK1	0.373663306	1.81165906	5.022973904	7.94E-07	5.66E-05	5.509056019
SMTN	0.373525063	3.516089376	3.830591567	0.000150223	0.002452384	0.64817995
KLC4	0.373313748	3.774901044	4.676192924	4.11E-06	0.000186141	3.97509236
AFDN	0.373291867	4.649383152	3.738644614	0.000214411	0.003143529	0.322883486
JMY	0.372776139	2.799760242	4.896563549	1.46E-06	8.79E-05	4.938770049
KLF12	0.372583398	2.340520908	4.266270531	2.53E-05	0.000676514	2.287682121
LRRC75A	0.37255778	1.852681465	4.377065848	1.57E-05	0.000477138	2.730093123
LIMD2	0.372358011	4.021084102	3.254093596	0.001242716	0.010936272	-1.26960083
SMAD9	0.37218591	1.516309558	4.176645864	3.70E-05	0.000884694	

1.937304714

PTPRU 0.371867661 5.214774474 2.608429622 0.009465933
0.045672491 -3.066063624

KCNJ110.37180952 2.227927879 3.721725372 0.000228741
0.003284424 0.263820915

NGF 0.37128033 1.718084529 2.944504934 0.00343988 0.022430626
-2.17804158

MAP1B 0.370728253 3.244461246 2.616678865 0.009244362
0.045006103 -3.045501923

DCHS1 0.370660297 2.574709825 2.643421403 0.008557667
0.042520251 -2.978415184

SEC61A2 0.370437001 3.188740461 4.087634149 5.35E-05
0.0011654461.596006186

ATP1A2 0.370014025 0.844468194 2.92722648 0.003632217
0.023352135 -2.226201525

TDRKH0.369970902 3.345428857 4.04982546 6.25E-05 0.001302637
1.453061149

SERAC1 0.369694528 1.848775405 4.902366842 1.42E-06 8.68E-
05 4.964673314

CPVL 0.369691853 4.486471539 2.687963799 0.007515009
0.038734752 -2.865219915

SLC18A3 0.369535576 0.781809368 2.8694119330.00434936
0.026412896 -2.385381158

BMERB1 0.369288688 2.809223944 2.85368891 0.004565534
0.027315536 -2.428146716

PAPLN 0.369224981 2.178052739 2.886383828 0.004126507
0.025549079 -2.338966956

MGARP 0.369035827 1.643415551 2.907501574 0.003863758
0.024379048 -2.280850121

ADAMTS9 0.368816903 2.383714971 2.590437673 0.009965765
0.047391577 -3.110692716

SLC25A33 0.36857722 3.817134839 4.416664163 1.32E-05 0.00042599
2.890682841

SP4	0.368540288	2.63720877	4.588533993	6.13E-06	0.000250382	3.602662223
HRK	0.368376494	1.162908325	3.055647356	0.002409347	0.017557846	-1.861812878
USP22	0.368328118	5.887374185	3.921231635	0.000105056	0.001929164	0.975970591
ATXN7L3	0.368316706	5.176884406	4.657629079	4.47E-06	0.000198809	3.89570043
DDX25	0.368313478	0.603845752	4.533352586	7.86E-06	0.000296242	3.371429174
ARHGEF19	0.368126182	4.259027843	3.715921065	0.000233861	0.003334797	0.24361604
RBP7	0.367664921	2.887176582	2.945009399	0.003434407	0.022403968	-2.176631438
ABAT	0.367626382	2.546384054	3.067917435	0.002314969	0.017063599	-1.826219361
FAAP24	0.367464465	3.283369001	4.049630683	6.26E-05	0.001302637	1.452327881
SEMA3D	0.367177096	1.046325635	3.19768815	0.00150516	0.012525969	-1.441509597
C8orf88	0.366965293	1.653238726	2.955846999	0.003318734	0.021843159	-2.146281252
PLCXD3	0.366623048	0.881971276	3.115810281	0.001978213	0.015225273	-1.685996012
CRYBG2	0.366491007	1.768847793	3.463305193	0.000596306	0.006585014	-0.607322693
BMP6	0.366487743	2.09641728	2.593210576	0.009887224	0.047128908	-3.10383391
GIPC2	0.366445249	0.9035847	3.29542733	0.001078072	0.009945764	-1.141828553
PAGE2B	0.366410778	0.492130214	2.602740889	0.009621489	0.046216284	-3.080206698
ASAP3	0.36596122	4.187212212	3.314479784	0.001009215		

0.009531283 -1.08242197
PLAGL2 0.365554208 5.43401938 3.378285727 0.000807259
0.008121792 -0.881128863
CCNG2 0.365279864 4.1105638224.432551923 1.23E-05 0.000406162
2.955479568
FBLIM1 0.364725944 4.915010528 2.988616535 0.00299031
0.020364122 -2.053866216
CUL7 0.364579878 5.476630046 3.34092587 0.000920395
0.008986207 -0.999428079
SULT4A1 0.364249729 1.670634254 3.376496133 0.000812369
0.008148316 -0.886823727
NHLRC1 0.36414311 3.494479331 3.222925831 0.001381965
0.011788971-1.364942426
DVL2 0.364078965 4.459317495 4.717332839 3.40E-06
0.0001621174.152029779
DLG4 0.364027156 3.255422808 3.380736597 0.00080031
0.008092136 -0.873325088
FBL 0.363992445 7.976931953 3.470054305 0.000581987
0.006465331 -0.585314317
NPHS1 0.363699898 0.481001953 3.207315164 0.001457015
0.012264098 -1.412369774
DLGAP3 0.363673555 2.068303041 2.624988837 0.009025869
0.044221837 -3.024725684
ADAMTS160.363601882 1.12241196 2.634804919 0.008773749
0.043301315 -3.000102279
TET1 0.363409827 1.343209276 4.326499639 1.95E-05
0.000561349 2.526913033
CTNNBL1 0.363388056 4.58697473 5.039050189 7.35E-07 5.40E-05
5.582487585
B3GALT1 0.363341829 0.842640941 3.309880227 0.001025454
0.009634025 -1.096793132
RBBP9 0.363089901 4.515909358 4.084430003 5.42E-05
0.0011777641.583845197

PLEKHG1 0.362630626 3.672236124 3.51014681 0.000503337 0.00581303 -0.453750437
ECH1 0.36237123 7.108528942 3.80038436 0.000168981 0.00269337 0.540506589
SERPINB7 0.361966681 0.706376107 2.657984746 0.008203291 0.041240785 -2.941605484
JSRP1 0.361800887 1.459302433 2.736158664 0.006516929 0.035096831 -2.740697454
SLIT1 0.36149058 0.449704 4.365198188 1.65E-05 0.000494902 2.682217101
TTC25 0.3611805051.743866907 3.231675072 0.001341484 0.011523972-1.338266023
CACNB3 0.360800479 3.742955245 3.869014019 0.0001292 0.002218889 0.786270366
KPNA5 0.36071875 2.313545262 4.875889646 1.61E-06 9.39E-05 4.84670915
ASXL1 0.360507894 4.966121601 4.2826570112.36E-05 0.000642425 2.352469961
ARL3 0.360492275 4.649968547 4.708602264 3.54E-06 0.000167814 4.114366298
SLC9A2 0.360292448 1.695579093 2.697544954 0.007306266 0.038055623 -2.840633964
TRAF4 0.360164854 5.371309908 3.669424195 0.000278955 0.003786025 0.082814084
PACSIN1 0.360079475 2.737421546 2.681925358 0.007649311 0.039198782 -2.88067184
CCDC157 0.360023905 1.658434346 4.963123339 1.06E-06 6.93E-05 5.237469422
SLC26A11 0.358801739 3.60733492 4.163775522 3.90E-05 0.000916578 1.887543003
MIIP 0.358761024 5.344439251 5.1106029495.17E-07 4.33E-05 5.911781955
SLC2A10 0.35871092 3.893265109 3.001209313 0.002872227

0.019815304 -2.018094884

KCNJ12 0.35792084 1.85325987 2.661755318 0.0081137160.04090486 -
2.93204346

IQGAP2 0.357836735 2.310946102 3.328544508 0.000961032
0.009248456 -1.038360808

SCNN1D 0.357829256 3.108090866 2.710724955 0.00702765
0.037045723 -2.806675895

MAP1LC3C 0.3576111160.805634641 3.660427698 0.000288576
0.003877561 0.051917863

RBP5 0.357268783 2.473882627 2.973345731 0.003139465
0.021045205 -2.097052838

MAGEL2 0.357006025 0.944584004 3.198842073 0.001499313
0.012509481 -1.43802117

IFT52 0.3568307 5.564756688 4.417365562 1.32E-05 0.00042599
2.893539033

LGI4 0.356616633 0.935441989 4.1511677834.11E-05 0.000955665
1.838931739

GLCCI1 0.356606138 2.270480921 4.235476672 2.88E-05
0.000741815 2.166538282

EZR 0.356501354 7.663572661 3.715144435 0.000234554
0.003334921 0.240914791

HSF2 0.356350298 3.817032067 4.491739718 9.47E-06
0.000337732 3.198703101

FKBP1B 0.356339019 3.002485375 3.259550526 0.001219718
0.010786033 -1.252819215

TMC7 0.35606224 0.941843974 5.894933576 8.47E-09 1.96E-06
9.778514581

GGT7 0.356014206 5.077310282 3.29591904 0.001076242
0.009943227 -1.140299426

PRDM11 0.355838178 2.2253861174.0116657557.30E-05 0.001462208
1.310017991

EYA1 0.355627461 0.586022206 3.010937385 0.00278395
0.019429998 -1.990363195

GALNT8 0.355585484 0.50480606 4.524404939 8.18E-06 0.000304041 3.334169553
MATN3 0.35552801 1.783486702 2.929773989 0.003603255 0.023196763 -2.219117871
ARMC2 0.355380808 1.669856837 4.804111799 2.27E-06 0.000121372 4.529732208
TAF11 0.355372878 5.784932944 4.620635161 5.30E-06 0.000224338 3.738322929
SPOCK3 0.355182255 0.860115214 2.621310207 0.009122013 0.044544499 -3.033930658
DYNC1I1 0.355045412 0.980655578 4.147841296 4.17E-05 0.000960666 1.826128219
CDK5R2 0.354454259 0.652444628 4.077931742 5.57E-05 0.001201626 1.559208415
CCDC181 0.354429566 1.137884157 3.182650888 0.001583314 0.012982696 -1.486860046
SLC26A7 0.354168466 1.156040411 2.709752351 0.007047878 0.037116071 -2.809187224
UQCC1 0.354069953 4.312025879 4.704124634 3.61E-06 0.000170824 4.095073813
LRP6 0.353612861 3.696154534 3.537632819 0.000455292 0.005415961 -0.362740034
CLVS1 0.353354828 0.582599159 5.003204437 8.75E-07 5.95E-05 5.419034348
NOVA1 0.353296537 1.168946119 3.339211349 0.000925925 0.009023864 -1.004827428
TUBB8P12 0.352371842 0.531342987 4.474974284 1.02E-05 0.000353668 3.129515255
CASP9 0.352206077 3.235111756 4.580189763 6.36E-06 0.000256777 3.567536771
ERBB2 0.352154377 5.789072412 3.295363979 0.001078308 0.009945764 -1.142025548
WDR54 0.35204115 4.70936857 3.313067828 0.001014175 0.009546578

-1.086835573

TUBD1 0.351715466 2.828378621 4.749717159 2.92E-06 0.000146836 4.29227227

USP42 0.351645557 3.429356157 4.671604309 4.20E-06 0.00018958 3.955442251

RHOBTB1 0.351532267 2.922006215 3.183676679 0.001577866 0.012970892 -1.483772809

CCM2L 0.351488182 1.991431872 3.797753245 0.000170715 0.002705026 0.531165209

DGKG 0.351470344 1.164171689 2.601484269 0.009656157 0.046327792 -3.083326842

HACE1 0.35143356 1.955763723 4.78317696 2.50E-06 0.000131723 4.438059613

HDAC1 0.3511758376.351846099 4.752698134 2.88E-06 0.000145262 4.305224084

RMND1 0.35106321 4.229718569 4.3717690111.61E-05 0.000486355 2.708710417

SPART 0.350797727 4.079150635 4.039405004 6.52E-05 0.001339253 1.413876982

NUDT17 0.350744043 2.650992966 3.686231479 0.000261785 0.003613295 0.140722878

MAP1LC3A 0.350740494 5.402187038 3.338419582 0.000928489 0.009027103 -1.007319971

VPS37D 0.350423906 4.696404148 3.325562145 0.000971067 0.009280156 -1.047718435

C12orf75 0.350259585 4.808421398 3.074862544 0.002263068 0.01677802 -1.80601275

CYP26B1 0.349472925 1.261246857 2.733799141 0.006562858 0.035320664 -2.746843242

USP44 0.349459731 0.902646385 3.92782928 0.000102329 0.001891975 1.000105111

NEK3 0.349437241 2.323637899 4.5089711818.77E-06 0.000319396 3.270054845

MAGED2 0.349330226 7.792263443 3.906412864 0.000111434 0.002014273 0.921898501
PCMTD2 0.349278568 4.7886811254.055397435 6.11E-05 0.001286644 1.474051296
GRIN2C 0.349233242 0.532227637 4.306966692 2.13E-05 0.000596866 2.44899668
SLC38A4 0.349002962 0.719187423 5.025100074 7.86E-07 5.66E-05 5.518756036
CYP2D6 0.348873258 1.631220393 3.162963034 0.001691313 0.013575727 -1.545930694
MPP2 0.348594303 1.506400366 3.521996491 0.000482069 0.005655486 -0.414595522
ARMCX1 0.348591561 3.905802899 3.270330334 0.001175441 0.010541178-1.219590388
ZMIZ1 0.348375768 5.597331765 3.122737429 0.001933427 0.01495886 -1.665543988
KLHL7 0.348343787 3.512133621 4.691500608 3.83E-06 0.000177445 4.04076875
GDPD5 0.348195376 2.616359188 2.733108671 0.006576353 0.035358008 -2.74864073
FLRT1 0.348003443 0.654625918 3.68465122 0.000263356 0.0036291150.135267714
TCF24 0.347599959 0.466395022 6.02807071 4.03E-09 1.28E-06 10.48028135
CERK 0.347443986 4.838047396 3.587745569 0.000378565 0.004721995 -0.195107016
ERAL1 0.3472115745.982108475 4.642728204 4.79E-06 0.000208271 3.832176204
SV2A 0.3470491172.531875756 2.569083973 0.010589529 0.049356784 -3.163274488
FOXRED2 0.3467658114.7001172913.183168018 0.001580566 0.012972992 -1.485303801
STK11IP 0.346588684 3.480476677 4.176158858 3.70E-05

0.000885193 1.935419226
BTBD9 0.346183296 2.808750156 4.095941584 5.17E-05
0.0011399961.627576595
CROCC 0.34617116 3.030650335 3.539896701 0.000451532
0.005379151 -0.355214466
PHF23 0.34564779 5.442638656 5.921419568 7.32E-09 1.79E-06
9.917099241
MORN4 0.345613109 3.432773094 3.91314047 0.000108494
0.001978823 0.946423519
ARHGEF40 0.3453901194.125194377 2.789464047 0.005553734
0.03147051 -2.600500442
RGS22 0.345280309 0.793504903 3.889826899 0.000119007
0.0021104440.861601044
TMEM151A0.3451169010.923085622 2.609047337 0.009449178
0.045618861 -3.064526099
ZNF280A 0.344888474 0.493675658 5.150853903 4.23E-07 3.93E-
05 6.098783217
WSB1 0.344661315 4.248922834 3.588596875 0.000377373
0.00471076 -0.192240338
FXR2 0.344569262 4.464347048 4.3466611771.79E-05 0.00052567
2.607669626
FAM120B 0.344279509 3.836221094 4.352872534 1.74E-05
0.000513563 2.632617121
CHST13 0.343916572 1.336623769 3.347691332 0.000898871
0.008813292 -0.97809697
CCNF 0.343800068 3.355013186 3.512338068 0.000499339
0.005778473 -0.446519141
PITPNC1 0.343542327 3.140776366 2.799632971 0.005385356
0.03079685 -2.573461228
GTF2IRD1 0.342882404 4.514606464 4.218618165 3.10E-05
0.000775763 2.100552609
HSPB7 0.342606828 1.601737788 2.857944003 0.004506087
0.027090106 -2.416595304

CEP170 0.342560885 2.420974048 4.448201995 1.15E-05
0.000387779 3.019510581
DDOST 0.342386341 8.161233139 5.023161562 7.94E-07 5.66E-05
5.509912007
ARID1B 0.342348743 3.905051791 4.10360457 5.01E-05 0.00111664
1.65674965
PREX1 0.341620904 3.860773357 2.879243777 0.004218962
0.025915851 -2.358525183
SHISA4 0.34124974 5.041925304 3.272057102 0.0011684890.010502117-
1.214258011
BRCA1 0.340420868 1.860669141 3.243333912 0.001289246
0.01121367 -1.302612276
CR2 0.340340827 0.746692005 3.441212921 0.000645516
0.006911422-0.679083742
TPX2 0.340306865 6.290341636 2.877236189 0.004245295
0.025996592 -2.364016097
MZT1 0.339723914 4.977095654 4.231528841 2.93E-05
0.000749348 2.151064783
CYTH3 0.339500188 3.929562886 3.615753846 0.000341149
0.004380244 -0.100460392
PIGU 0.339486261 5.651013396 4.534302718 7.83E-06
0.000296242 3.375389551
DUSP8 0.339056844 3.164659813 2.602131618 0.009638284
0.046272616 -3.081719678
ADAMTSL4 0.338837784 2.358717283 3.041930003 0.002519035
0.018152763 -1.901444418
ORAI2 0.338739345 3.363974609 4.045497896 6.36E-05
0.001314681 1.436776975
LYPD8 0.338659929 0.4452711364.393094632 1.46E-05 0.000458601
2.794941032
PRB3 0.338514187 0.572463619 4.252028971 2.69E-05
0.000705394 2.2315569
ULK1 0.338489006 4.65398383 3.319539593 0.000991628

0.009420179	-1.066591089
PDCD2 0.338010891	4.408021314	4.874650991	1.62E-06	9.39E-05	4.841204235
DUOX1 0.337898897	1.6667811992.612915576	0.009344859	0.045342074	-3.054889883
RNF2	0.33745956	4.65334831	4.684155157	3.96E-06	0.000181476	4.009230003
FAM222A	0.337132547	2.466743382	2.674858531	0.007809226	0.039753455	-2.898712935
SPATA2 0.336902571	4.180850937	4.695734845	3.75E-06	0.000175238	4.058968894
PIMREG	0.336524259	3.560470632	3.050400087	0.002450777	0.017787648	-1.876993032
ARL10	0.336278814	1.591548778	4.006352054	7.46E-05	0.001488599	1.290197484
FAM172A	0.336131093	3.830482843	4.473626819	1.03E-05	0.000353889	3.123964555
DCAF15	0.335637997	5.879831263	3.45007204	0.000625344	0.006789809	-0.65035877
KLHDC3	0.335566089	7.564531824	3.967659148	8.72E-05	0.001667047	1.146594526
FBXL18	0.335474991	2.547128751	3.601472494	0.000359771	0.004557845	-0.14880599
PTK7	0.335412446	5.623950754	3.249838391	0.001260929	0.01104306	-1.282668409
TRAF7	0.335293003	6.303541033	4.00342203	7.55E-05	0.001500571	1.279278543
ORMDL3	0.335208866	4.523997894	3.965493144	8.80E-05	0.001674948	1.138593417
CES4A	0.335053565	1.140546761	3.440926538	0.000646178	0.006913935	-0.68001116
IFT81	0.334588577	2.649318124	3.76024344	0.000197346	0.002958909	0.398641633

BCORL1 0.334473772 3.465343786 3.3886111480.000778358
0.007944095 -0.848215937
ATOH8 0.334463126 1.674904601 2.723875526 0.006759241
0.036041858 -2.772635356
EPB41L2 0.334180362 3.995615208 2.676678755 0.007767751
0.039654995 -2.894070409
DTX1 0.333955205 2.21231203 3.197749034 0.001504851
0.012525969 -1.441325568
ICAM2 0.333533508 1.969344844 3.68633219 0.000261686
0.003613295 0.141070612
RAB34 0.333425125 6.4958711013.94827792 9.43E-05 0.001769768
1.075143564
AURKB 0.333103827 4.694564749 2.89773296 0.003983343
0.024925716 -2.307783957
PLCB4 0.33305205 2.363409904 2.802684506 0.005335739
0.030599639 -2.565328816
CHAD 0.332733798 1.342442593 3.259227815 0.001221067
0.010788868 -1.253812382
ARHGEF4 0.33270113 1.999672067 3.171641209 0.001642896
0.013302689 -1.519935745
ADAM21 0.332579057 0.683622022 4.408632185 1.37E-05
0.00043691 2.858004538
RALGAPB 0.332143883 4.237867253 3.799048803 0.000169859
0.002699391 0.535764148
AC233723.1 0.331952108 2.420005863 2.828968164 0.004925234
0.028797441 -2.494931373
CKM 0.331947833 0.721869795 4.451327131 1.13E-05
0.000383263 3.032321015
TMEM189 0.331796227 3.7551545115.153482872 4.18E-07 3.91E-05
6.111041085
REV3L 0.331510802 2.215589376 4.1768710113.69E-05 0.000884694
1.938176457
FNDC3A 0.331446754 4.20598082 3.9081160610.0001106820.002002931

0.928103724
PRPF40B 0.331416968 2.234937682 4.245179699 2.77E-05 0.000721037 2.204624665
MAP9 0.331404691 2.560199744 3.725099621 0.000225813 0.003261783 0.275580135
CACFD1 0.331256437 4.39444124 3.4789116570.000563682 0.00631964 -0.55637035
ZFP69B 0.330970497 1.827259551 3.47320763 0.000575407 0.006415532 -0.575017817
GPR3 0.330860002 1.730046395 3.533338098 0.000462504 0.005471205 -0.377004155
CRNKL1 0.330242568 4.455444 5.005703681 8.64E-07 5.92E-05 5.43039781
ANXA6 0.33015781 5.139201266 3.138245894 0.001836528 0.014429993 -1.619600209
KRAS 0.3301157324.43544142 2.777522733 0.00575754 0.032252703 -2.632132177
FBXO30 0.329875682 2.336984206 5.007681631 8.56E-07 5.90E-05 5.439394569
RNFT2 0.3298581 2.3028911443.72193032 0.000228562 0.003284424 0.264534876
GLIS1 0.329852993 1.18916304 3.124754739 0.001920561 0.014887798 -1.659579903
PARD6G 0.329537132 2.996520877 3.255693315 0.001235933 0.010896415 -1.264683984
TSPEAR 0.329501929 0.527094294 3.967548237 8.73E-05 0.001667047 1.146184727
ODC1 0.329443548 7.407306493 3.121826195 0.001939264 0.014989687 -1.668236812
LAMC1 0.329388904 6.462234144 3.206785639 0.001459626 0.012279683 -1.413974731
ZNF850 0.329150435 1.708029159 3.477805505 0.000565938 0.006336154 -0.559988792

TNRC18 0.328914107 5.560246992 2.712891688 0.006982775 0.036871986 -2.801078136
DPCD 0.328846831 5.340823272 4.023826504 6.95E-05 0.001409148 1.35546881
ARMH4 0.328704874 1.491342679 3.698648729 0.000249745 0.003494528 0.183663492
POLR3F 0.328702516 3.870279887 4.92871619 1.25E-06 7.86E-05 5.082621743
XKR8 0.328658443 3.55286843 4.787526657 2.45E-06 0.000129911 4.457077755
CAMK1G 0.328614479 1.535864557 2.572299089 0.010493436 0.049064396 -3.15538434
KLHL24 0.328605556 5.003335916 3.897772832 0.00011532 0.002054672 0.89045869
TSC22D3 0.328583432 4.950271731 2.646623229 0.008478598 0.042257164 -2.970339059
NCOR2 0.328527791 5.976039197 2.932387639 0.003573761 0.023045021 -2.211844203
KPNA3 0.328298246 4.505890781 4.238659746 2.85E-05 0.000734195 2.179023818
VPS33A 0.32782544 3.1108120725.05299992 6.86E-07 5.21E-05 5.646370513
AKAP8L 0.327799304 5.02642803 3.684099778 0.000263907 0.003633603 0.13336461
RPP25 0.3274711974.297237771 2.65448037 0.008287338 0.04154058 -2.950480763
GPATCH3 0.327436475 3.780532593 5.08903455 5.75E-07 4.74E-05 5.812098865
MMS19 0.327168283 5.16663838 4.0611856115.97E-05 0.001265235 1.495883756
SAT2 0.327057069 5.189536883 3.97559772 8.45E-05 0.001639055 1.175953388
GPX7 0.327052534 5.48707577 2.878537227 0.004228213

0.025942909 -2.360458071
LTV1 0.326520008 4.508759454 4.834740019 1.96E-06
0.000107913 4.664484867
OTUD1 0.326188899 4.727678294 3.166958883 0.001668858
0.01344561 -1.533969732
SPSB2 0.326090526 3.663854962 3.918235407 0.000106317
0.001947897 0.965022528
MERTK0.326072031 3.294916542 3.364040968 0.000848774
0.008439748 -0.926380246
NATD1 0.32596953 3.196807068 3.281427879 0.0011314210.010254412
-1.185274201
SUPT5H 0.325925843 5.727251242 3.257543497 0.00122813
0.010845355 -1.258994482
AKIRIN1 0.32565584 6.204697623 3.124104106 0.001924702
0.014909901 -1.661503867
CCDC152 0.325614975 1.50926671 3.416325936 0.000705482
0.00736441 -0.759407839
PAF1 0.325594617 5.86439735 3.454785109 0.000614854
0.006716479 -0.635048872
MTURN 0.325343913 3.5011321173.791210159 0.000175102
0.002747361 0.507960832
RCBTB1 0.32514289 3.815045734 4.032449725 6.71E-05
0.001367378 1.38777423
FIG4 0.3251146193.982146635 4.1479041174.17E-05 0.000960666
1.826369929
FOXO3B 0.325045856 1.086052287 4.262186216 2.57E-05
0.00068501 2.271568722
PHF10 0.324988288 4.532046873 3.68207954 0.000265932
0.003658374 0.126394731
ARID3A 0.324771062 2.222985359 2.763771427 0.006000609
0.033123687 -2.668397436
TUB 0.324641048 2.404990778 2.6397659110.008648748
0.042841227 -2.987624123

PEPD 0.324426956 5.770744498 3.380245421 0.000801698 0.008101109-0.87488946
PRKN 0.324024824 1.032757666 4.574348693 6.53E-06 0.00026169 3.542982308
TRPM5 0.3238147110.995917514 2.864752749 0.004412427 0.026665761 -2.398077223
CECR2 0.323666332 0.774581756 2.958937942 0.003286403 0.021709853 -2.137605755
SCN8A 0.323542855 1.321508016 3.872699639 0.000127336 0.002203261 0.799583126
PAN2 0.323527499 3.9051171353.23287659 0.0013360110.011496803-1.334597275
ASPRV1 0.323474784 1.267788767 4.169402349 3.81E-05 0.000905272 1.909281349
MYL3 0.322802439 0.76552546 3.655747767 0.000293703 0.003933386 0.035873625
CSPG4 0.322754999 2.4241136072.688274034 0.007508167 0.038726224 -2.864425144
C12orf49 0.322686056 3.708454467 3.7789671170.000183597 0.002836544 0.464641328
IFNLR1 0.322592846 2.576257216 2.909042708 0.003845194 0.024318822 -2.276593042
SPERT 0.3221120390.779866375 4.456480018 1.11E-05 0.000376996 3.053461105
CLUL1 0.322043437 2.144175599 2.649024493 0.00841973 0.042028505 -2.96427606
TIGD1 0.321966316 3.343333307 3.365399102 0.000844732 0.00840472 -0.922073595
DNMT3B 0.321849007 2.995868637 3.102788952 0.002064997 0.015713793 -1.724324332
HAL 0.321577622 0.763628778 3.245259954 0.0012808 0.011158205-1.296710619
RNF1220.321499896 4.601382419 3.308513587 0.001030325

0.009674173 -1.101059536
HIST1H4L 0.321478572 0.873241573 2.79438532 0.00547166
0.031169197-2.587426516
GRB14 0.320775206 1.3573457 2.739751499 0.006447551
0.034839171 -2.731329524
KIF3B 0.320699591 4.948262294 4.162542017 3.92E-05
0.000919494 1.882781113
AC092835.1 0.320583262 0.805481564 5.221498612 2.97E-07 3.02E-05 6.43004112
PLPP7 0.32056171 1.165791574 3.471396986 0.000579177
0.006444236 -0.580931151
WRAP53 0.320296945 3.047759454 4.475378875 1.02E-05
0.000353668 3.131182209
SYS1 0.320139918 4.491468271 4.721873455 3.33E-06
0.000160159 4.171642216
SASH1 0.320074194 3.040458206 3.17566281 0.001620896
0.013177314 -1.507866454
TP53TG3D 0.319875367 0.751671257 3.877090168 0.00012515
0.002174734 0.815457287
BTRC 0.3198086 3.19076988 4.291592992 2.27E-05 0.00062673
2.387894895
MAPRE1 0.319798249 7.068801441 4.650977745 4.61E-06
0.000202185 3.867322718
NEURL4 0.319785904 2.917668808 3.853807887 0.000137163
0.00231993 0.731467849
SVBP 0.31958383 5.530990434 3.693474642 0.000254697
0.003549087 0.165754504
NDNF 0.319473833 1.031572819 2.872603363 0.004306636
0.026261877 -2.376673288
ALPK3 0.319430006 1.437723574 2.590105955 0.009975198
0.047408564 -3.11151275
VAT1 0.319255954 6.595740692 3.4419458 0.000643824
0.006897876 -0.676710061

SWT1 0.319123676 2.6101128384.659853556 4.43E-06 0.000198034
3.905199107
GPR137C 0.318887274 1.731867286 3.376304598 0.000812918
0.008148316 -0.887433064
ZNF3160.318425381 4.53228028 2.857484104 0.004512478
0.027118451-2.417844597
FAM110B 0.318202279 2.863252929 2.748169575 0.006287602
0.034199048 -2.709334142
KIT0.318188444 1.056615265 3.056278433 0.002404408
0.017544694 -1.859985527
SV2C 0.318164741 0.672306592 3.2531146150.001246885
0.010957143 -1.272608672
BIRC7 0.318038442 0.552945135 3.768099418 0.000191463
0.002913914 0.426296562
MMP23B 0.317773328 1.482828036 3.065350863 0.002334425
0.017166511-1.833675765
RIPOR30.31749644 1.1182004083.468039154 0.000586228 0.006495892
-0.591889773
ZNF1330.317401914 3.341755808 4.410562881 1.36E-05 0.00043581
2.865854756
CLCN6 0.317354945 3.19274175 3.184322222 0.001574446
0.012955946 -1.481829491
AUNIP 0.317354219 2.929375866 3.35902258 0.000863867
0.008574017 -0.942279434
PHC1 0.317107519 3.502006853 2.925654575 0.003650193
0.023405798 -2.230569463
TBP 0.316962286 4.5465898 4.392164375 1.47E-05 0.000459476
2.79117166
SLC15A1 0.316908247 0.96629948 3.0551148890.002413522
0.017564532 -1.863354414
ARL6 0.316801093 2.683774595 4.189627814 3.50E-05
0.000856154 1.987638994
CCDC126 0.316674269 2.544945373 4.364102797 1.66E-05

0.000496346 2.677804006
SCAF8 0.316527306 4.191378173 4.290908672 2.28E-05
0.000627501 2.38517969
ENPEP 0.316388981 1.794295254 3.846243216 0.000141296
0.002363497 0.704278924
TEAD2 0.316230209 6.180496762 3.070234191 0.002297536
0.016980143 -1.819483639
PNMA8B 0.315879397 1.284536413 4.424864701 1.27E-05
0.000415893 2.92410193
LATS1 0.31577296 3.299785538 4.274507093 2.44E-05 0.000659668
2.320219308
PTCH2 0.315731606 1.582298937 2.958374947 0.003292271
0.021717462 -2.139186582
EFEMP2 0.31562067 5.489992639 2.713688587 0.006966336
0.036818484 -2.799018268
FAM221A 0.315566152 2.240675891 4.139141631 4.32E-05
0.000986657 1.792687606
GRIK4 0.315451641 0.766831729 3.839913785 0.000144844
0.002403788 0.68156748
YWHAE 0.314963527 8.647069383 4.0980006 5.13E-05
0.0011349841.635410411
PCIF1 0.314866222 6.364660463 4.54598219 7.43E-06 0.000287419
3.424132733
MMP24OS 0.314787486 5.2591611323.442662324 0.000642175
0.006884883 -0.674388897
ZBTB39 0.314541932 3.1165033163.64083682 0.000310616
0.004088812 -0.015118628
IFT88 0.314533605 2.344939501 4.533714094 7.85E-06
0.000296242 3.372935938
FOXO3 0.313910847 4.727101378 3.313010768 0.001014376
0.009546578 -1.087013901
KATNA1 0.313815622 4.144138288 4.7178942113.39E-06
0.0001621174.154453629

SLC38A8 0.313519334 0.530523328 2.761301043 0.006045247 0.033299286 -2.674894118
YIF1B 0.313476132 5.573733704 3.277401029 0.001147214 0.010380105 -1.19773881
NCAPH 0.313428177 3.967313854 2.877332815 0.004244024 0.025996592 -2.3637519
SIRT4 0.313414906 1.857147581 4.645423952 4.73E-06 0.00020683 3.843655146
EPHA7 0.313206317 0.570162686 3.564916988 0.000411876 0.005018388 -0.27174361
LRIG3 0.313078344 3.426002496 3.032312872 0.002598652 0.018503955 -1.92912882
KCND1 0.312965095 2.0073913 2.73079833 0.006621692 0.035483846 -2.754652033
MB21D2 0.312884414 3.266971753 3.110148111 0.002015528 0.015426382 -1.702681312
MBTD1 0.312813556 3.310866937 3.866841862 0.00013031 0.002233833 0.778429818
ABCA3 0.312410467 3.741737531 2.646051294 0.008492673 0.042314281 -2.971782368
ZNF318 0.312299439 3.324091532 3.605443234 0.000354499 0.004513058 -0.135381991
MAP3K7 0.312186078 4.022620888 3.978049668 8.37E-05 0.001624889 1.185032174
NOXA1 0.312075987 5.315512337 2.597422482 0.009768984 0.046702901 -3.093402195
PPP3CA 0.312020947 4.410663203 3.666084373 0.000282491 0.003817997 0.071336088
URI1 0.3119447525.926121912 3.136736574 0.001845758 0.014474394 -1.624081028
DACT3 0.31185787 1.680799378 2.779669608 0.005720406 0.032139203 -2.626454831
TAS1R10.3117942840.650267257 4.530598077 7.96E-06 0.000297991

3.359951915
ZNF117 0.3117643983.021778604 2.9189119650.003728227 0.023755518
-2.249280155
APMAP 0.3116433816.894334463 4.3841132221.52E-05 0.000468764
2.758578587
ZNF697 0.3116230441.738434883 3.663540956 0.000285212
0.003841394 0.062601609
AL162231.1 0.3114796811.164649233 3.908894634 0.000110341
0.002001222 0.93094111
C2orf68 0.31147 5.131405891 5.086418108 5.82E-07 4.78E-05
5.800031182
OSTC 0.3111777977.024272386 4.076308472 5.61E-05 0.001206458
1.553059711
PNMA6A 0.3111634062.244648897 2.77535312 0.005795288
0.032396706 -2.637865387
NKIRAS2 0.3110773954.717850269 4.300346873 2.19E-05
0.0006119712.422662488
CNTN5 0.310860313 0.456482336 5.673383465 2.84E-08 4.73E-06
8.639416687
CKAP4 0.310529883 6.614297688 3.183075841 0.001581055
0.012972992 -1.485581216
CRY1 0.310428156 3.61203708 3.652160387 0.000297691
0.003970362 0.023587886
CCDC138 0.310255381 2.295585739 4.216800516 3.12E-05
0.00078054 2.093452396
UST 0.3101191582.169984643 2.93627255 0.003530328 0.022845459
-2.20102125
ADGRD1 0.309698568 0.586449628 3.869861014 0.000128769
0.002216201 0.789328752
PRKAR1B 0.309406447 4.375719172 3.33891505 0.000926884
0.009027103 -1.005760265
ZNF727 0.309395133 1.015684589 3.461006367 0.000601259
0.006621756 -0.614809857

ARL5B 0.309108947 3.919507154 3.521384876 0.000483146 0.005664011-0.416619495
PRRG3 0.309106233 1.069369122 3.008967545 0.002801621 0.019493101 -1.995985483
FOXN1 0.309049776 1.079961294 2.850564999 0.004609631 0.027477746 -2.43661681
DNASE1 0.308866731 2.00275835 4.049721785 6.25E-05 0.001302637 1.452670845
LINGO2 0.3087471150.5911952353.418393907 0.000700308 0.007333058 -0.752754149
ACTR5 0.308717814 3.391718043 4.379571533 1.55E-05 0.000475438 2.740216382
TAB2 0.308699414 5.404186816 3.943073296 9.63E-05 0.00180285 1.056010764
SAMD4B 0.308683879 6.077673139 2.814147272 0.005153039 0.029741657 -2.534704541
PTPRG 0.308635256 3.362585792 2.917946243 0.003739528 0.023808767 -2.251956656
TULP4 0.308537834 2.834532327 3.598351716 0.000363965 0.004589055 -0.159346842
BRSK2 0.308416366 0.720340685 3.020080598 0.002703255 0.019014458 -1.964221001
B4GALT6 0.3082841141.808703242 3.170094682 0.001651429 0.013338405 -1.524573203
SUN1 0.308239493 4.583468733 3.630844473 0.000322458 0.004196886 -0.049182039
SUPT6H 0.308195836 4.5671411023.433987712 0.000662416 0.007036355 -0.702459721
PTPN200.308060077 0.914324123 2.722717178 0.006782506 0.036139929 -2.775640113
OXT 0.308047096 0.993853882 3.48986907 0.000541781 0.006146518 -0.520468431
COQ8B 0.307881056 4.32495881 3.326986135 0.000966263

0.009260786 -1.043251428
FRRS1L 0.307587685 0.869580129 3.139904658 0.001826434
0.014364632 -1.614673375
RFTN2 0.307396073 1.2873195114.525978365 8.12E-06 0.000302822
3.340716832
LBX2 0.307303958 0.833190727 3.271732558 0.001169792
0.010503444 -1.215260428
SERP2 0.30722567 1.857084678 3.509608617 0.000504323
0.005815355 -0.455525868
AMHR2 0.307058096 0.753424916 2.631358549 0.008861538
0.043628107 -3.008757487
NR2F6 0.30687681 7.80861247 3.056103906 0.002405773 0.017544694
-1.860490923
ST6GALNAC3 0.306658109 1.065402931 3.564872476 0.000411943
0.005018388 -0.271892594
AAR2 0.306603685 5.532235407 4.421595866 1.29E-05
0.000421062 2.910774012
FER1L50.306461922 0.686676606 4.05234253 6.19E-05 0.001296608
1.462539924
ANKS1A 0.306344509 4.535812448 2.974220464 0.003130741
0.021007517 -2.094584733
CASTOR3 0.306312772 3.468474295 2.657862772 0.008206204
0.04124261 -2.941914587
PPIL4 0.305693151 4.816837498 4.486672349 9.68E-06
0.000342732 3.177766638
ADNP 0.305581287 5.776176265 3.972670987 8.55E-05
0.001650573 1.165123333
ZNF678 0.305533327 1.913075837 4.259580784 2.60E-05
0.000690391 2.261297077
HLTF 0.305452255 4.552660319 3.406415451 0.000730777
0.007544681 -0.791242442
UVRAG 0.305257181 4.246308015 3.131258399 0.001879619
0.01466175 -1.640327309

TACR2 0.305250352 1.641755727 2.982756795 0.003046759
0.020632262 -2.070462752
ULBP3 0.305226806 1.844102996 2.848946871 0.004632624
0.027584316 -2.441000677
ATAT1 0.305022597 4.231204587 2.992662159 0.002951898
0.020192729 -2.04238972
ARRDC3 0.304957761 5.317496903 2.929245254 0.003609249
0.023226105 -2.220588567
SLC16A10 0.304743524 0.986672092 3.881496853 0.000122991
0.002145295 0.831406479
LIMS2 0.304734469 3.166406275 2.672792125 0.007856552
0.039946786 -2.903979679
HIST1H1A 0.304650454 0.65193767 3.266162824 0.001192379
0.010645862 -1.232449019
LCOR 0.304532305 2.88629019 4.28451791 2.34E-05 0.0006384
2.359841635
UBA2 0.304410689 5.97543432 3.144993708 0.001795783
0.014192564 -1.599542586
CASKIN1 0.303635569 1.687086168 3.140358067 0.001823684
0.014356961 -1.613326237
COL25A1 0.303482747 0.736915526 2.871233007 0.004324934
0.026333756 -2.380413459
PCDH10 0.3034693 0.712571703 2.760080649 0.006067409
0.033341896 -2.678101488
MFSD6L 0.303457364 2.879692609 2.779900842 0.00571642
0.032133508 -2.62584309
TEDC2 0.30329109 3.1897852 3.313909044 0.0010112170.009533493 -
1.084206246
MED21 0.302945043 5.15460509 3.179796485 0.001598567
0.013074628 -1.495445753
HBS1L 0.30285554 3.31627267 4.354497306 1.73E-05 0.000510886
2.639148194
SRCIN10.302838777 1.2110209212.673187085 0.007847486

0.039923469 -2.902973328
NAP1L3 0.302756554 1.78079245 2.706508592 0.007115721 0.037339647 -2.817556572
FKBP7 0.302655673 3.53905666 2.966876651 0.00320468 0.021291709 -2.115284244
RPN2 0.302576111 8.412593846 3.761124745 0.000196678 0.002954414 0.401741384
SSBP3 0.301255098 6.680643585 4.17109891 3.78E-05 0.000900182 1.915840966
AMIGO1 0.300903571 2.460954016 3.368286691 0.000836197 0.008360937 -0.912911612
CHST7 0.300689064 1.999085083 3.437489921 0.000654173 0.006976411 -0.691134639
SOX6 0.300579915 1.293382404 3.151115112 0.001759543 0.013987153 -1.581311683
HMCN1 0.300482093 1.580007663 2.865260214 0.004405518 0.026633956 -2.39669536
CD302 0.300196824 1.731708208 3.407771449 0.000727267 0.0075199 -0.786891803
SHKBP1 0.300028071 6.171050242 3.000008156 0.002883303 0.019866302 -2.021513088
TBPL1 0.300009964 3.363278773 4.059507041 6.01E-05 0.001269248 1.489549422
NFKBIB 0.300008505 4.89211512 3.187609397 0.001557138 0.012852744 -1.471928134
SOGA1 0.29997543 4.822038577 2.572884018 0.010476038 0.049011377 -3.153947854
SENP3 0.299963627 4.203444271 4.794609674 2.37E-06 0.000126494 4.48807928
CLCNKB 0.299776328 1.593806228 2.837633137 0.004796324 0.028282758 -2.47158562
LZIC 0.299746471 4.592923134 3.768168328 0.000191412 0.002913914 0.426539378

SPATS2 0.299722055 3.628552182 3.385396017 0.000787252 0.007995084 -0.858474467
EFR3B 0.299671021 0.970752572 3.522156908 0.000481787 0.005655486 -0.414064613
VASN 0.299553185 6.711135551 2.590185734 0.009972929 0.047408564 -3.11131554
TRMT6 0.299481689 4.180274229 3.971484523 8.59E-05 0.001654526 1.16073503
PAQR7 0.299350233 3.809443861 3.02468406 0.002663443 0.018865669 -1.951030316
PRKG2 0.299198613 0.763986825 4.185855389 3.56E-05 0.000862623 1.97299776
DENND2A 0.299161429 1.613795262 2.611720091 0.009376988 0.045392255 -3.057869438
NPY1R 0.299096569 0.691115689 2.684422504 0.007593512 0.03903649 -2.874285876
PNPLA2 0.29894056 6.831808706 3.186388782 0.001563544 0.012885899 -1.475605896
BORCS5 0.298806229 2.796119834 4.149429915 4.14E-05 0.000958763 1.832241587
PFAS 0.298799296 3.59007751 3.425480746 0.000682843 0.007195677 -0.72992366
NCKAP5L 0.298798841 4.119865132 3.326080919 0.000969314 0.009273545 -1.046091267
TCAP 0.298776585 1.317017799 3.44571302 0.000635195 0.006855356 -0.664501172
CSRNP2 0.298562333 4.653679759 3.765864401 0.00019312 0.002927022 0.418423339
LDB1 0.29815089 6.963620613 3.508575191 0.000506223 0.005833098 -0.458934292
MPZ 0.298021747 1.597665321 2.676429999 0.007773408 0.039658842 -2.894705046
ACTR1A 0.297311424 5.841339969 4.316865784 2.04E-05

0.000578843 2.488444182
EID3 0.297173713 2.014340894 3.488662873 0.000544152
0.00616323 -0.524425699
NLK 0.297172894 3.424266793 3.766487745 0.000192656
0.002923822 0.420618742
RPRD1B 0.29707616 4.539820796 3.3554622 0.000874727
0.008628859 -0.95354587
NHLRC4 0.29694124 1.38726682 2.865468871 0.00440268 0.026633956
-2.396127103
CYGB 0.296874003 2.71613612 2.809012129 0.005234172
0.030166882 -2.548438532
UCKL1 0.296722186 5.714679471 3.680560501 0.000267464
0.003673209 0.121156339
KLHL30 0.296660173 1.026431327 3.245560665 0.001279486
0.011152764-1.295788903
RNF1460.296490422 4.293668096 4.129986389 4.49E-05
0.001020642 1.757564687
TIMELESS 0.29648413 4.444533558 2.645863734 0.008497294
0.042324268 -2.97225562
MINPP1 0.296483249 4.040477988 3.594997126 0.000368525
0.004625284 -0.170667948
ATG9A 0.296268888 5.032934373 3.549437913 0.000436005
0.005240486 -0.323448388
GRID1 0.296025378 0.928347731 3.155302052 0.001735146
0.013848642 -1.568822734
NRIP3 0.295977536 1.703198849 3.4692447110.000583687
0.006476608 -0.58795646
SLC7A6 0.295757522 1.788477434 4.019813683 7.07E-05
0.001424033 1.340456994
C20orf194 0.295315453 2.672868572 3.352885269 0.000882666
0.008691274 -0.96169329
MAPK8IP3 0.295219638 3.688735531 2.828922991 0.004925914
0.028797441 -2.495052903

RIN2 0.295155024 5.10244812 3.351675447 0.000886416
0.008717589 -0.965516325
PBX4 0.295123126 2.1146918423.437967623 0.000653056
0.006969094 -0.68958906
CELSR3 0.295088661 1.353953515 3.327812207 0.000963487
0.009248456 -1.040659247
NUP88 0.295080257 4.401378448 3.8394111850.000145129
0.002403788 0.67976551
CHTF18 0.295026346 3.838956471 3.18058416 0.001594344
0.013046689 -1.493077246
BRD4 0.294823344 5.665226635 3.158315829 0.001717779
0.013742927 -1.559823427
TIMM50 0.2947811465.242635458 3.024003217 0.002669297
0.018884217 -1.952982396
SLC66A1 0.294654327 4.299999258 3.846209156 0.000141315
0.002363497 0.704156617
RTBDN 0.294364572 0.523258839 3.032700191 0.002595401
0.018497092 -1.928015473
EMC1 0.294353014 4.0470541173.692176265 0.000255954
0.003560472 0.1612641
PIK3CD 0.294344887 3.248954813 2.626266738 0.008992683
0.04413945 -3.021525105
TMEM8A 0.293845498 5.847586515 3.416287716 0.000705578
0.00736441 -0.759530775
CCDC88A 0.293770179 2.233707244 3.338566634 0.000928013
0.009027103 -1.006857082
NMB 0.293662158 4.712703695 2.625436588 0.009014229
0.044191574 -3.023604436
AKR1C1 0.293661862 0.685625656 4.394791649 1.45E-05
0.000456095 2.801819131
TWNK 0.293394234 3.915869075 3.41897443 0.000698861
0.007322655 -0.750885639
OTOS 0.2933179110.392226453 3.398308839 0.000752095

0.007730237 -0.817218179
CCZ1B 0.2931676112.205058145 4.147465957 4.18E-05 0.000960666
1.824684137
FAM117A 0.293140507 4.577191543 3.898897949 0.000114807
0.002047783 0.894549208
FCGRT 0.293132196 6.209522203 3.180834894 0.001593002
0.013042305 -1.492323184
ATP13A2 0.293083807 5.223569946 3.4430651160.000641249
0.006884883 -0.673083863
CCDC91 0.292853128 3.164734155 4.068377927 5.79E-05
0.001236551 1.523052049
ZYG11A 0.292782298 1.034735259 2.981048891 0.003063395
0.020712426 -2.075294214
PRAF2 0.292722884 6.343794675 2.88638258 0.004126523
0.025549079 -2.338970379
MAPK10 0.2926119070.899620409 3.269248088 0.001179818
0.010562864 -1.222931086
DESI2 0.292521335 5.105031836 3.385024274 0.000788287
0.008000567 -0.859660001
NFYA 0.292477314 4.505520243 3.337786247 0.000930545
0.009041658 -1.009313358
TBCB 0.292453382 6.268106738 3.640958183 0.000310475
0.004088812 -0.014704372
RHBDL1 0.292270001 2.207393977 2.566182968 0.010676907
0.049692463 -3.170385618
ZKSCAN7 0.292233491 2.093468751 3.446414842 0.000633599
0.00684727 -0.66222531
SNPH 0.292125322 1.759310612 2.750289179 0.006247897
0.034044623 -2.703785684
ST3GAL2 0.292075693 3.247343668 3.098812983 0.002092187
0.015853583 -1.735997302
RGL2 0.291879974 5.384274488 2.951016756 0.003369842
0.022079825 -2.159821234

LURAP1 0.291528015 1.018193018 3.83694319 0.000146538 0.002419074 0.670920181
ERC1 0.291522057 3.818941994 2.903512473 0.00391219 0.02460786 -2.291859242
RNLS 0.29068402 3.222977779 3.795471477 0.000172233 0.0027211510.523068964
IGDCC4 0.29043336 1.386563552 2.873436802 0.004295542 0.026213986 -2.374397711
TMEM256 0.290202104 6.041316284 2.834646675 0.004840404 0.028475655 -2.479639656
ZNF232 0.289917166 2.97489772 3.217883655 0.001405806 0.011936722-1.380285006
P3H4 0.289846526 4.6011840793.008740799 0.002803662 0.019493101 -1.996632431
DDX43 0.289767448 0.552293251 2.932659703 0.003570703 0.023045021 -2.211086705
CHKA 0.289607888 4.079313236 3.251585358 0.001253422 0.010996669 -1.277305486
CREBBP 0.289470228 4.644333759 2.951613664 0.003363487 0.022056951 -2.158149141
EDEM2 0.289445534 6.165836355 3.848091708 0.000140275 0.002356569 0.710918231
SLC39A5 0.289346167 0.499371846 3.523765766 0.000478967 0.005635058 -0.408738742
TMEM184B 0.289328972 5.754196859 3.124017206 0.001925255 0.014909901 -1.661760809
SLC43A1 0.289085925 2.295475857 2.727348481 0.006689918 0.03576652 -2.763619157
NUP98 0.288918994 4.658358163 3.555272268 0.000426759 0.005160019 -0.303984497
SETDB1 0.288795665 4.6911794613.690512043 0.000257574 0.003575102 0.155510565
POP4 0.288759432 4.366456169 2.773594781 0.005826045

0.032478893 -2.642508652
PDK2 0.288648523 4.219777374 3.036142778 0.002566673
0.018384457 -1.918113817
TUBE1 0.288525567 2.291384562 4.3121133432.08E-05 0.000588092
2.469495704
HDAC5 0.288307107 4.333544 3.366335001 0.000841957
0.008382281 -0.919104903
KIF1B 0.288270562 3.745809762 2.900816666 0.003945233
0.024776248 -2.299290964
CEP89 0.288253083 3.551705319 3.598900308 0.000363224
0.004589055 -0.157494512
ING1 0.288124919 3.604515357 4.317851289 2.03E-05
0.000577558 2.492375848
IL20RB 0.287805064 1.249543839 3.905436254 0.000111867
0.002019842 0.918341555
PLEKHM2 0.287751098 5.862858182 3.274399391 0.001159119
0.01044183 -1.207020628
MYH7B 0.287524729 1.241073756 3.163228181 0.001689814
0.013575727 -1.545137458
ARL2 0.287310382 6.252882307 3.625724101 0.00032869
0.004260849 -0.066603439
CD79B 0.286952973 1.512870247 2.717967101 0.006878669
0.036510204 -2.787948997
SH3D21 0.286798906 2.875562607 2.662313791 0.008100524
0.040851082 -2.930626086
SPAG5 0.286647346 3.51851011 2.650717313 0.00837845 0.041887072
-2.95999865
ZNF695 0.286623148 1.531420608 3.648173716 0.000302183
0.004013633 0.009947816
MANBAL 0.286595989 6.450542162 4.481898334 9.89E-06
0.000347752 3.158061515
MAD2L2 0.286421379 5.092644223 2.890357621 0.004075854
0.02536122 -2.328061805

TCP1 0.2863112476.263156856 3.7493110250.000205817 0.003054773 0.360245646
SNRNP200 0.286242469 6.135408885 3.1109922740.002009924 0.015407392 -1.700195553
TP53BP2 0.286220217 4.666227809 3.131270531 0.001879543 0.01466175 -1.640291359
RSPH9 0.286137451 1.729427999 2.58962101 0.009989003 0.047446296 -3.112711393
ETNK1 0.286094442 4.200516627 3.210435583 0.001441719 0.012179699 -1.402906891
TM6SF2 0.286090143 0.86761633 4.142538241 4.26E-05 0.000975548 1.805736235
RAD17 0.285937738 3.816500995 4.150605439 4.12E-05 0.000955665 1.836766644
CLCN4 0.285764947 2.083792504 2.687586155 0.007523346 0.038749661 -2.866187256
RPL6 0.285540978 9.048834042 3.54405919 0.000444696 0.005325157 -0.341365863
TTLL4 0.285512928 3.665005699 3.198640212 0.001500334 0.012511551-1.438631503
GJB7 0.285229339 0.653914813 3.03294542 0.002593345 0.018490583 -1.927310492
TRPC4AP 0.28509311 6.500346344 3.900712349 0.000113983 0.002042871 0.90114799
ZGPAT 0.284974245 2.906366894 3.988643577 8.02E-05 0.001569793 1.224316915
PI15 0.284882442 0.737771531 3.179230458 0.001601608 0.0130833 -1.497147429
WTAP 0.284839245 5.031263586 3.970597591 8.62E-05 0.001658469 1.157455374
ATP6V0A1 0.284825043 3.419806077 3.554468592 0.000428022 0.005160836 -0.306667393
ZNF518A 0.284823906 2.471546369 4.002244836 7.59E-05

0.001500571 1.274893701
GPR62 0.284752151 0.695931046 3.990870232 7.94E-05
0.001557912 1.232586036
VASH2 0.284628598 1.327782803 3.042418951 0.002515048
0.018132106 -1.90003468
CCNC 0.28459282 4.52051899 2.986524516 0.003010353 0.020453622
-2.059794997
NUP43 0.284562549 4.228928131 3.909739431 0.000109971
0.001998997 0.934020429
SPAST 0.284358948 3.893614891 3.739092959 0.000214043
0.003142275 0.324451961
INPP4A 0.283926578 2.600076838 4.160724153 3.95E-05
0.000925171 1.875765664
PROSER3 0.283919381 3.484040905 2.742173805 0.006401153
0.034669518 -2.725006969
AKIRIN2 0.283891587 6.328527281 3.76213266 0.000195916
0.002948397 0.405287274
RAB2B 0.283875489 4.416160394 3.501753901 0.000518928
0.005941417 -0.481408632
RAB3B 0.283689166 0.767773746 3.285986887 0.001113784
0.010140012 -1.171144972
RAP2A 0.283454916 3.776330357 3.577359892 0.000393393
0.004847929 -0.230028666
GLT1D1 0.283235584 0.649522427 2.9991142320.002891571
0.019914791 -2.024056138
SNCAIP 0.283227197 1.177815804 2.867868384 0.004370162
0.026519313 -2.389589441
TCP11L2 0.283093224 1.877490813 3.259464027 0.001220079
0.010786033 -1.253085431
BBS10 0.283049417 3.980146252 3.299545678 0.001062829
0.009870518 -1.129014613
PLA2G4F 0.282823003 0.485041712 3.88095104 0.000123257
0.002145295 0.829430106

BBS5 0.282789877 2.871632353 3.904189158 0.000112422
0.002025345 0.913800651
COX7A1 0.282779295 2.881286596 2.930719263 0.003592563
0.023137133 -2.216487931
SH3BP5L 0.282769214 4.13703276 3.535146247 0.000459455
0.005449455 -0.371000692
MRPL28 0.282554733 5.856501863 4.053676403 6.15E-05
0.001291222 1.467565197
BBS12 0.282220255 2.345984627 3.533685073 0.000461917
0.005470645 -0.375852342
PTBP2 0.281895751 2.8371547112.871565682 0.004320486
0.026326091 -2.379505632
WIPI2 0.28171106 4.892733498 3.998256857 7.71E-05 0.001523136
1.260047945
MYMX 0.281513876 0.524972144 4.185428852 3.56E-05
0.000862877 1.971343072
PRPSAP2 0.281348598 3.591224252 4.397445396 1.44E-05
0.000454176 2.812579692
HPCA 0.280855722 1.520271304 2.676952423 0.007761533
0.039644513 -2.893372149
DRC3 0.280809251 1.665259747 2.9501176710.003379434
0.022125649 -2.162339193
RCC2 0.280716298 7.236427969 3.314013924 0.001010849
0.009533493 -1.083878385
PSD3 0.280563254 1.52507571 3.468940366 0.000584328
0.006479271 -0.588949553
IFT20 0.280444771 3.717143372 3.753276372 0.000202705
0.003025246 0.374160491
NDFIP2 0.280380267 4.7433117293.48369935 0.000554013 0.006241514
-0.54069647
RAB26 0.280257971 1.769351886 2.668687093 0.007951332
0.040312333 -2.914430759
UBAC2 0.279999239 5.38144412 3.676948492 0.0002711420.003707972

0.108708406
VARS 0.279941513 5.581294688 3.326286745 0.00096862
0.009273545 -1.045445615
CHD6 0.279914369 3.754839348 3.008186697 0.002808655
0.019493101 -1.998213198
SINHCAF 0.279819036 6.30892898 2.837578648 0.004797125
0.028282758 -2.471732642
DCUN1D2 0.279799359 3.471515505 3.229012587 0.001353685
0.011605679-1.346391153
PROC 0.279633305 1.091095226 3.715810748 0.000233959
0.003334797 0.243232306
PTDSS2 0.279457822 3.945353601 3.816360415 0.000158801
0.002569301 0.597355242
BMPR1A 0.279319579 3.424015061 3.775607695 0.000185996
0.002865266 0.45277731
ADA 0.279289037 3.502886776 3.244459955 0.001284302
0.011176676-1.299162327
CBX8 0.278800014 2.955729932 4.040203636 6.50E-05
0.001336591 1.416876829
BACH2 0.27842318 0.930786899 3.520666263 0.000484414
0.005674765 -0.418997131
NR0B1 0.277950755 0.602420082 2.613436807 0.009330882
0.04529103 -3.053590389
KALRN0.277883689 1.567388361 3.137440496 0.001841448
0.014454609 -1.621991501
PSMD13 0.277883334 5.539971736 4.41910104 1.31E-05
0.000423994 2.900607878
ARHGEF33 0.2778811380.559034617 5.662895725 3.00E-08 4.95E-06
8.586393149
GLYCTK 0.277787448 2.143789802 2.727198368 0.006692901
0.035770647 -2.764009096
CCDC167 0.277782526 6.935305408 2.6831102280.007622789
0.039137252 -2.877642478

TDG 0.2777711293.800919132 3.325367169 0.000971726
0.009280156 -1.048329929
NFS1 0.277622909 3.657948024 4.294653354 2.24E-05
0.000622806 2.400042405
RPL27A 0.277553043 8.77954523 2.919140034 0.003725563
0.023749215 -2.248647935
PGD 0.277472416 6.563170929 3.023949368 0.002669761
0.018884217 -1.953136772
TAOK1 0.277355334 3.129968031 3.799439894 0.000169601
0.002699391 0.537152716
EME1 0.277185318 2.455770018 2.942477963 0.003461952
0.02253821 -2.183705305
LBR 0.277049162 4.155666929 2.750601434 0.006242067
0.034024315 -2.702967953
KAT14 0.277032174 4.231341227 3.519390975 0.000486672
0.005680983 -0.423215491
GAST 0.276781962 0.562220813 2.7111750170.007018308 0.0370206
-2.805513512
SLC7A9 0.276579708 0.90641403 3.605013683 0.000355066
0.004513592 -0.136834852
MIEF2 0.276512875 3.53573163 4.264036971 2.55E-05 0.000681866
2.278868577
WTIP 0.276462009 2.617877698 2.701233162 0.007227314
0.03773477 -2.831147362
DISP3 0.276428348 0.592926422 2.635718561 0.008750607
0.043200282 -2.997805935
TMEM106B0.276255564 4.007381903 3.648391202 0.000301936
0.004013633 0.010691573
MTRF1L 0.276075958 2.776820526 4.591504442 6.05E-06
0.000248503 3.61518019
BAG6 0.275964938 7.312442625 3.555649961 0.000426167
0.005160019 -0.302723455
DAAM1 0.275940727 2.933736496 3.005888321 0.002829452

0.019587023 -2.004767145
SLC12A1 0.275918254 0.444901703 3.204502624 0.001470932 0.01235552 -1.420891549
SLC8A1 0.275887926 1.327590324 3.414847207 0.000709203 0.007397482 -0.764163333
SPHK2 0.2758811253.539314365 3.1107787150.0020113410.015407392 -1.700824469
KCNIP2 0.275846043 1.44912509 3.366666386 0.000840976 0.008382281 -0.918053557
ALOX12 0.275814884 1.726435134 2.923076572 0.003679851 0.023539991 -2.237728263
DHX37 0.275780483 3.885902477 3.28948241 0.0011004350.0100581 -1.160299179
RPS6KC1 0.275212466 3.453630831 3.69565467 0.000252599 0.003522889 0.17329737
NUDT16L1 0.275131872 5.670493493 3.600033939 0.000361698 0.004578681 -0.153665959
REPS1 0.27505293 3.548770388 3.542087549 0.000447922 0.005351904 -0.347927407
RRP12 0.274966694 3.650005421 3.298551756 0.001066489 0.009881821 -1.132108508
CPEB1 0.274896609 0.488625673 4.103827886 5.01E-05 0.00111664 1.657600557
ADH5 0.274862301 5.575331823 3.806084512 0.00016528 0.002650047 0.560764591
KLRG1 0.2747895 1.291476578 3.965765016 8.79E-05 0.001674948 1.13959748
SBNO1 0.27465319 3.575181453 3.7112876740.000238026 0.003363761 0.227508086
EIF4E2 0.274545607 4.630215937 5.297750472 2.02E-07 2.24E-05 6.791929924
CPB2 0.274500352 0.392321224 6.281049983 9.48E-10 4.65E-07 11.84869877

SUFU 0.274486835 3.718212992 3.268081681 0.001184552 0.010587671 -1.226530407
NMUR1 0.274464786 0.606839591 4.189435951 3.50E-05 0.000856154 1.986894061
CLCN7 0.274293532 4.4174031172.906341772 0.003877783 0.024448468 -2.28405243
CPLANE2 0.274106034 3.003740498 3.046941265 0.002478443 0.017924116-1.886985734
GLI1 0.274060225 0.750344098 2.770483347 0.005880832 0.03267139 -2.650718148
SMOC1 0.274006329 0.601650928 2.7332722 0.006573155 0.03535256 -2.748215057
OBSCN 0.273740163 2.482955584 2.583380889 0.010168167 0.048001343 -3.128115866
EID2 0.273564635 4.429460792 2.917984767 0.003739076 0.023808767 -2.251849901
C12orf76 0.273504031 2.473081502 4.246565108 2.75E-05 0.000717959 2.210069112
ACSS3 0.273483386 1.79413837 2.620589277 0.009140962 0.044610101 -3.035733173
KIAA0355 0.273334582 3.726624613 2.732058204 0.006596933 0.035399603 -2.751374558
AP5S1 0.273252607 3.540615046 4.0774267 5.58E-05 0.001202521 1.557295154
UBXN11 0.273252313 4.52338908 2.899129374 0.003966044 0.024869248 -2.303939091
FAM117B 0.273098666 3.693102678 3.044770772 0.00249595 0.018018509 -1.893250898
THNSL1 0.273090652 3.868673788 3.034595556 0.002579548 0.018434781 -1.922565297
ASPG 0.273001408 1.013412681 2.726500665 0.006706781 0.035832995 -2.765821203
CSRNP3 0.272923168 1.32745851 3.328860986 0.000959973

0.009248456 -1.037367345

FAM217B 0.272884799 3.36273241 3.357873501 0.000867358 0.008598115-0.945916797

CAPN10 0.272866951 2.700474297 3.64796118 0.000302424 0.004013633 0.00922103

DUOXA1 0.272862237 0.9107011162.756974856 0.006124141 0.033562453 -2.686257828

SLC35E2A 0.272792605 1.912214392 2.748099351 0.006288921 0.034199048 -2.709517895

KMT2B 0.272457683 4.582994683 2.667872158 0.00797027 0.040370392 -2.916503682

PLEKHA8 0.272436179 1.817690214 3.612509035 0.000345299 0.004414872 -0.111460425

RNFT1 0.272237546 2.987652965 3.316389713 0.001002543 0.009495985 -1.076448946

ANKRD27 0.272229207 3.976121576 2.886009042 0.004131314 0.025549079 -2.339994729

PSMB1 0.272151794 7.042200715 3.656351475 0.000293037 0.003927716 0.037942252

SMOX 0.272022096 3.563387018 2.774483955 0.005810473 0.032424586 -2.640160954

CCDC92 0.271974676 4.198466774 3.795213183 0.000172406 0.0027211510.522152758

CDYL 0.271901508 4.405821546 3.612365567 0.000345483 0.004414872 -0.111946574

TPD52L2 0.27177857 6.53713848 3.871352544 0.000128015 0.002209805 0.79471596

ZNF780A 0.271731372 2.035649879 3.273970583 0.001160829 0.010444881 -1.208345956

RHOQ 0.271590086 4.969764577 3.533257143 0.000462641 0.005471205 -0.377272878

ACVR2B 0.27156726 2.251957348 3.23056907 0.00134654 0.011550542-1.341641988

DPP10 0.271490219 0.567937524 2.792243159 0.00550725 0.031305792 -2.593120122
ELP5 0.271363239 5.201032713 3.831402978 0.000149748 0.002449544 0.651083051
CCDC192 0.271308361 0.759693939 5.048652295 7.01E-07 5.25E-05 5.626444111
CBX1 0.2711427525.751626772 3.076657993 0.002249826 0.016703848 -1.800781888
PFKFB2 0.271033624 2.195634755 3.519374902 0.000486701 0.005680983 -0.423268649
PRPF6 0.270734201 7.008580279 3.67608599 0.000272027 0.003710667 0.105737672
C17orf75 0.270472322 2.818993843 3.844164676 0.000142452 0.00237499 0.696816852
GCDH 0.270437637 4.297107218 3.015601555 0.002742513 0.019240406 -1.977036855
TMEM199 0.270401717 2.872879594 4.383682157 1.52E-05 0.000468764 2.756835041
RB1CC1 0.270267063 4.304018607 3.746677781 0.000207908 0.00307874 0.351012807
DDHD1 0.270049254 1.599722444 4.489572369 9.56E-06 0.000339823 3.18974585
ZBTB40 0.26976647 3.105175693 2.916405474 0.003757621 0.023886977 -2.256225152
UBN1 0.269721773 4.361390573 2.907862344 0.003859405 0.024370591 -2.27985376
HHIPL20.269599793 0.528234528 2.839536497 0.004768421 0.028185429 -2.466448319
PYGO1 0.269460724 1.1116838792.866822216 0.004384313 0.026585236 -2.392440452
SMARCAD1 0.269430505 3.803986961 3.377314266 0.000810029 0.008139537 -0.884220608
SLC43A2 0.269265796 3.482308474 2.709820082 0.007046468

0.037116071-2.809012364

RAB29 0.269159948 3.904139564 2.8301329 0.004907725 0.028756673 -2.491797235

INTS1 0.269104392 5.473002322 2.639622265 0.008652345 0.042845925 -2.987985747

MTHFR0.269091537 2.973925326 2.800286724 0.005374691 0.030749237 -2.571719675

PCMT1 0.269020566 5.614806642 3.714877978 0.000234793 0.003334921 0.239988132

USP35 0.26895678 2.8411983372.653198236 0.008318281 0.041676417 -2.953725116

MLNR 0.268943533 0.438970994 4.396036871 1.44E-05 0.000454783 2.806867603

GPER1 0.268887627 1.535885513 2.889712653 0.004084037 0.025395142 -2.329832742

USP30 0.268863868 3.552677544 3.459257537 0.000605052 0.006640791 -0.620502596

RSPH3 0.268764388 3.280098229 3.609700463 0.000348929 0.004451867 -0.120974177

MAP4K4 0.268725237 4.24603155 2.824440775 0.004993833 0.029040341 -2.507102215

NECAB3 0.26859036 5.258346138 2.889614338 0.004085285 0.025395142 -2.33010266

JADE3 0.268584486 3.586914248 2.923109313 0.003679473 0.023539991 -2.237637382

TUBG2 0.268565792 3.688377467 3.330796783 0.000953518 0.009198574 -1.03128872

ARID1A 0.26855723 5.672789499 2.865265663 0.004405444 0.026633956 -2.396680522

UTP14C 0.268441004 3.706856763 3.331455064 0.000951332 0.009188445 -1.029220886

BORA 0.268183532 2.633368296 2.939509401 0.003494509 0.022677164 -2.191993299

FAM184A 0.268084534 0.593740658 4.686849102 3.91E-06
0.000180258 4.020791771
GPATCH1 0.267951568 3.225595806 3.377720274 0.000808871
0.008132945 -0.882928558
DDX52 0.267900053 3.050394217 3.484759936 0.000551892
0.006226293 -0.537221612
ATAD1 0.267765281 4.620656006 3.075043674 0.002261729
0.016776824 -1.805485178
PDZD7 0.267747782 0.963575663 3.055223931 0.002412666
0.017564532 -1.863038748
PIGQ 0.267558829 4.616970014 3.174430782 0.001627607
0.013207038 -1.511565448
CNTD1 0.267485208 0.972467873 4.634152633 4.98E-06
0.000214291 3.79569927
CCNYL1 0.267356551 3.458182071 3.50045988 0.000521372
0.005960958 -0.48566748
CASC3 0.267294421 5.365844277 2.9051128750.003892693
0.024504278 -2.287444183
LUC7L 0.26720484 3.721508986 2.988565317 0.002990799
0.020364122 -2.054011415
PDSS2 0.267193168 3.553026491 3.448386834 0.000629135
0.006808127 -0.655828236
EPB41 0.266731846 3.751414815 2.656742341 0.008233
0.041325932 -2.94475332
SLC17A5 0.266540837 4.030671967 2.8127211630.005175456
0.029849741 -2.538521092
ICA1 0.266473979 3.58599511 2.717240012 0.006893496
0.036542071 -2.789831285
RNASET2 0.2663841175.2334119052.845701886 0.004679049
0.027828903 -2.449784894
KBTBD7 0.266354468 2.974580905 3.735191496 0.000217265
0.00317804 0.310809037
PLEKHA5 0.266339154 2.70601569 3.013357356 0.002762379

0.019337851 -1.983451367
TIAM2 0.266332921 1.488064834 4.261682171 2.58E-05
0.000685357 2.269581135
DIABLO 0.266272912 2.668172003 3.847624718 0.000140533
0.002358441 0.709240646
USP5 0.266159841 6.393363181 3.31115127 0.001020942
0.009597214 -1.092823676
TTI1 0.266151351 4.819572186 3.36633443 0.000841959
0.008382281 -0.919106717
CNR1 0.2660407110.522338212 3.584017773 0.000383826
0.004769231 -0.207652501
CASTOR1 0.265979145 1.8896874113.366843214 0.000840454
0.008382281 -0.917492519
ZFYVE16 0.26596282 2.775035245 4.025152538 6.91E-05
0.001406854 1.360432459
LYPLA2 0.265885041 6.705321763 3.334017952 0.000942866
0.009128476 -1.021166519
ALMS1 0.265706286 2.627903736 3.020940933 0.002695773
0.018978337 -1.961757261
ZMYM2 0.265698449 3.715235547 3.512944035 0.000498239
0.005769868 -0.444518666
MED24 0.26549953 4.793365149 3.185960908 0.001565796 0.01289132
-1.476894785
RAD52 0.265289513 3.131992435 3.049585496 0.002457267
0.017802805 -1.879347394
FANCE 0.265197512 3.647817014 2.937504447 0.003516657
0.022784329 -2.197586466
CTNS 0.265132244 3.873463144 3.355982164 0.000873133
0.008627549 -0.9519012
PRPH 0.265031855 0.599938783 3.665707672 0.000282893
0.003820231 0.070042085
SLC38A9 0.26499276 2.135470019 3.778958889 0.000183603
0.002836544 0.464612259

SERTAD3 0.264678436 5.018372625 3.095092741 0.002117926 0.016018611-1.746906642
ACOT11 0.264514191 2.027870676 2.806422398 0.005275527 0.030329592 -2.555355691
RIT1 0.264366631 4.031245794 3.718324552 0.000231728 0.003308819 0.25197907
SEMA3G 0.26420329 1.616262201 2.636830623 0.008722513 0.043101045 -2.995009846
CD164 0.264180408 6.757273554 3.2192741 0.001399194 0.011907498-1.376056356
ZBED6 0.264109151 1.475310525 3.109346023 0.002020867 0.015434854 -1.70504258
CCDC24 0.26409784 4.192130156 3.289761032 0.001099377 0.0100581 -1.159434211
CPOX 0.263983157 3.955237615 3.723698391 0.000227024 0.003276358 0.270695667
PEAR1 0.263868402 1.413926013 2.85323295 0.004571946 0.027324826 -2.429383547
PGBD1 0.263868126 2.3843551142.807410691 0.00525971 0.030260168 -2.552716685
PCOLCE2 0.263859362 1.259996264 2.581513251 0.010222346 0.048174311-3.13271939
COL4A6 0.263837455 0.834230318 2.969888198 0.003174168 0.021184689-2.106801691
ZSWIM3 0.263806958 2.595645803 3.643839971 0.000307138 0.004066185 -0.004864051
CYP39A1 0.263506448 2.304401327 2.753457591 0.006188969 0.033836381 -2.695484136
DNAJC16 0.263376598 3.185203802 3.472204658 0.000577492 0.006434346 -0.578293754
SH2B2 0.263302018 4.325034465 2.7011850790.007228338 0.03773477 -2.83127112
MANEA 0.263152934 3.417260673 2.885241824 0.004141169

0.025590465 -2.342098271

NXPE3 0.263150589 1.676888876 3.355004503 0.000876132 0.008632193 -0.95499339

SPR -0.263292052 5.681448478 -2.778401401 0.005742315 0.032230117-2.629809075

NUDT7 -0.263765213 1.87397965 -3.327698664 0.000963868 0.009248456 -1.041015575

ATP6V0E2 -0.263774292 5.515979741 -2.826821914 0.004957647 0.028933913 -2.500703395

PGBD5 -0.263824989 1.21305103 -2.574158545 0.010438217 0.048919316 -3.150816747

CBR1 -0.26450637 5.431098031 -3.225533339 0.001369784 0.011706479-1.356999279

ITGA2 -0.264583699 1.749423028 -2.682365629 0.007639446 0.039173047 -2.879546347

HGH1 -0.264893956 5.713527918 -2.968079132 0.003192465 0.021264561 -2.111898226

MAX -0.265075697 5.175933421 -4.074323056 5.65E-05 0.0012131191.545542281

STX1B -0.265282841 0.953421982 -3.597601173 0.00036498 0.004595061 -0.161880636

ILDR1 -0.265606819 4.040794409 -2.80135312 0.005357336 0.030701727 -2.568878034

IL17C -0.265607237 0.59034504 -3.074741378 0.002263964 0.01677802 -1.806365652

DENND3 -0.265752183 2.133637195 -3.002481534 0.002860538 0.019759943 -2.014473031

COX8A -0.266370783 10.06887354 -3.45420178 0.000616143 0.006726023 -0.636944814

SELENOW -0.266632024 6.19530498 -2.97253713 0.00314755 0.021082074 -2.099333734

CMPK1 -0.267086729 5.959076266 -3.794884495 0.000172626 0.002721967 0.520986939

APRT -0.267136239 7.0480596 -3.321221482 0.000985846 0.009391588 -1.06132385
ACYP1 -0.267159381 3.562572111-3.162890822 0.001691721 0.013575727 -1.546146718
TATDN1 -0.268332488 4.383174948 -3.236578124 0.001319281 0.011395266-1.323286817
C2CD2L -0.268478247 2.891613558 -3.307283541 0.001034728 0.00970425 -1.104898102
TNFSF15 -0.268589469 1.405516676 -3.285163453 0.001116951 0.01016312 -1.173698316
SLC25A5 -0.268796442 8.965141559 -3.154308924 0.001740905 0.013887751 -1.57178649
DMAC1 -0.269060674 6.061464949 -3.11751333 0.001967114 0.015151234 -1.68097184
FRMD3 -0.269102197 1.646557572 -2.667156569 0.007986933 0.040442129 -2.918323402
XRCC3 -0.269217213 3.061919905 -3.267006734 0.001188931 0.010620939 -1.229846425
PRDX4 -0.269379356 7.241432386 -3.228460249 0.001356229 0.011621331-1.348075936
TMEM109 -0.26946223 6.792905346 -3.908930976 0.000110325 0.002001222 0.931073566
BAG1 -0.269711911 4.508971501 -3.350864532 0.000888938 0.008726477 -0.968078092
SYT3 -0.269768688 0.735729079 -3.388854569 0.000777689 0.007942832 -0.847438881
CLTRN -0.269929635 1.813255502 -2.952395998 0.003355175 0.022020296 -2.155957139
SLC25A23 -0.270804573 6.152915359 -2.650950788 0.008372771 0.04187162 -2.959408501
PTPRE -0.271189413 2.04093436 -3.536732424 0.000456795 0.005425865 -0.365731867
SNX7 -0.272114982 4.513756961 -2.675149113 0.007802592

0.039753455 -2.897972004
ITPK1 -0.272215321 5.290049782 -3.583495691 0.000384568
0.004771125-0.209408542
SYAP1 -0.272540515 4.809089909 -3.589329714 0.00037635
0.004701615 -0.189772078
SLIRP -0.272546062 5.538173871 -3.784449492 0.000179746
0.002801228 0.484023607
SORD -0.272578521 3.755996339 -2.994050803 0.002938818
0.020154384 -2.038447054
LRRC45 -0.272589425 3.897984482 -3.034562803 0.002579821
0.018434781 -1.922659506
MRPS25 -0.272901868 4.347191959 -3.861282375 0.000133193
0.00227059 0.75838091
ELF4 -0.273035874 5.683628673 -2.612731277 0.009349806
0.045342074 -3.055349306
ENGASE -0.273121325 2.945883985 -2.856649767 0.004524094
0.027157991 -2.420110541
MKRN2OS -0.273252212 1.99146133 -2.600121166 0.00969389
0.046453719 -3.08670974
REEP5 -0.273260747 6.22393165 -3.981530848 8.25E-05 0.001606097
1.197930675
CHRAC1 -0.273277627 6.184458345 -3.303444959 0.001048581
0.009778732 -1.11686841
ZNF586-0.273579783 3.020084791 -2.973266152 0.00314026
0.021045205 -2.097277342
SGCB -0.273708767 4.28340371 -2.866593495 0.004387412
0.026590734 -2.393063628
YBEY -0.274267292 3.987522351 -2.913478514 0.003792214
0.024049566 -2.264327989
ARHGAP20-0.274291294 1.240533317 -2.775748938 0.005788384
0.032380526 -2.636819756
DDT -0.274302778 4.980300101 -2.932367555 0.003573987
0.023045021 -2.21190012

TXLNG -0.274714821 3.961497832 -3.429231562 0.000673765 0.00713362 -0.717822364
TNIP3 -0.274797856 0.611387669 -3.484204893 0.000553001 0.006234456 -0.539040255
PTAR1 -0.27493347 2.893414905 -3.899505125 0.00011453 0.002045906 0.89675713
NUP205 -0.275278044 4.842735231 -3.439584324 0.00064929 0.006933472 -0.684356815
BID -0.275451813 4.526938542 -3.145006841 0.001795705 0.014192564 -1.599503508
NANS -0.275692587 4.10554982 -4.38670716 1.50E-05 0.000465268 2.769073632
CLTB -0.275712539 6.475306672 -3.708140687 0.000240894 0.003391809 0.216578217
PGRMC1 -0.275881953 7.850652372 -3.292102187 0.001090528 0.010001449 -1.152163505
PEX11G -0.276017304 2.608740198 -3.252148203 0.001251012 0.010981484 -1.275577061
INAFM1 -0.276126874 4.975560394 -3.147744102 0.001779416 0.014104552 -1.59135545
MTAP -0.276810138 2.835926757 -3.583677814 0.000384309 0.004771125 -0.208795992
ALG1L2 -0.277654502 1.95266384 -3.012547307 0.002769583 0.019371512 -1.985765586
ARPC1B -0.277834341 7.009427087 -2.891762908 0.004058078 0.025279777 -2.324201894
RLN1 -0.278447481 0.882708878 -3.290956378 0.001094851 0.01003379 -1.155722547
PSMG1 -0.278479317 5.147162811 -3.783503446 0.000180405 0.002801228 0.480677123
S100A13 -0.278560888 6.702589282 -2.625837613 0.009003815 0.044155877 -3.022600041
CASP7 -0.279460209 4.186758781 -3.286848824 0.001110479

0.010121308	-1.168471591
TRIP10	-0.27946583	5.265408078	-3.59104358	0.000373967	0.004686323	-0.183997809
FAM111A	-0.279643101	3.83097092	-3.578563881	0.000391647	0.004840382	-0.225985102
UHRF2	-0.27964884	3.117082369	-3.514750222	0.000494972	0.005744247	-0.438554003
OMA1	-0.280126887	3.738109766	-3.996935303	7.75E-05	0.001529437	1.255131274
ZNF367	-0.280606324	2.844548416	-2.84411851	0.004701854	0.027914349	-2.454067639
MSRA	-0.280796715	2.414407721	-3.677367685	0.000270713	0.003706112	0.110152472
DIS3L	-0.281026176	4.075199979	-3.376493869	0.000812375	0.008148316	-0.886830928
MAST4	-0.281047787	2.654581419	-2.739373097	0.006454826	0.03485712	-2.732316723
DAPK2	-0.281195547	1.346589736	-3.118830502	0.00195857	0.015095637	-1.677084265
AKT1	-0.281571863	5.246855857	-3.238336009	0.001311404	0.01135147	-1.317911124
ZC3H3	-0.281766363	5.839209192	-3.301636088	0.001055168	0.009810638	-1.122504696
MPP1	-0.281852507	3.02399909	-3.067067548	0.002321395	0.01710179	-1.828689108
INAFM2	-0.281950124	4.75186056	-3.62808917	0.000325797	0.004233534	-0.058559438
SLC35A4	-0.28342926	6.389865932	-4.063226149	5.92E-05	0.001259646	1.503587242
NUB1	-0.283673719	5.375657154	-3.833758728	0.000148375	0.002436963	0.659514756
MRPL13	-0.283835739	5.001086312	-3.335041861	0.000939503	0.009101367	-1.017947062

SH3GL2	-0.283846835	0.607860917	-3.159781049	0.001709393	0.013703948	-1.555445265
SEC14L2	-0.284311702	2.236578536	-2.920207683	0.003713114	0.023696554	-2.24568774
EMC2	-0.284322273	4.352752569	-3.773044039	0.000187846	0.002880873	0.443730148
TRAPPC9	-0.284564192	4.72785875	-3.307789821	0.001032914	0.009692853	-1.103318331
SHB	-0.284579012	3.987229239	-3.461000697	0.000601271	0.006621756	-0.614828319
APOBEC3C	-0.28469896	5.597556783	-2.803862264	0.0053167	0.030512087	-2.562187796
CLPP	-0.28486453	5.988214935	-3.995174456	7.81E-05	0.001536623	1.248582585
SYK	-0.285266208	5.183993077	-2.569833895	0.010567045	0.049280386	-3.161434969
GNG12	-0.28539569	5.733229111	-3.167136199	0.001667868	0.013444329	-1.533438635
POC1B	-0.285690295	3.067451295	-3.08617649	0.002180806	0.016326275	-1.773002339
ALDOA	-0.285986083	9.471248194	-3.90854963	0.000110492	0.002001723	0.929683736
EBAG9	-0.286068641	4.828603392	-3.264645122	0.001198603	0.010677863	-1.237127971
C5	-0.286093632	1.078161854	-3.664307313	0.00028439	0.003834046	0.065232804
CRYZ	-0.286159789	4.402755205	-3.150452371	0.001763434	0.01400532	-1.583287091
LRRC14	-0.28625314	4.193724383	-3.148127558	0.001777145	0.014093457	-1.590213473
RNF145	-0.286637607	4.893405637	-3.626751117	0.000327431	0.004247934	-0.063110981
C17orf99	-0.28680048	0.370410952	-5.767577473	1.70E-08	3.17E-06	

9.119297001
RMDN3 -0.286941903 4.031918187 -4.165359138 3.88E-05 0.000916578 1.893658358
CLDN23 -0.287015605 3.002916292 -2.886078141 0.004130427 0.025549079 -2.339805248
RNF125 -0.287072726 1.992755921 -3.03339681 0.002589564 0.018471767 -1.926012701
HSP90AA1 -0.287196248 8.871744534 -3.696702793 0.000251597 0.003511932 0.176925329
TLR5 -0.28721684 2.609353978 -2.679165522 0.007711409 0.039454516 -2.887722944
KLF9 -0.287419344 3.593763549 -2.742187802 0.006400886 0.034669518 -2.72497042
C9orf40 -0.287962103 3.265213893 -3.315958683 0.001004045 0.009503133 -1.077797211
NFIC -0.288019359 6.075692963 -3.208645639 0.001450475 0.012221762 -1.408336081
SLC66A3 -0.288244726 5.695007601 -3.417558808 0.000702393 0.007350135 -0.755441529
C2CD4C -0.28839186 0.554809723 -2.927000675 0.003634794 0.023353338 -2.226829119
LMF2 -0.288512041 5.805079564 -3.28831408 0.00110488 0.010087331 -1.163925441
BCL6 -0.288670865 5.508806856 -2.69304001 0.007403755 0.038402676 -2.852204446
MRRF -0.288909501 2.994127842 -4.188174192 3.52E-05 0.000857239 1.981995899
MICOS13 -0.29010849 5.816027742 -3.51764246 0.000489784 0.005708736 -0.42899686
TRAPPC2 -0.290964817 3.055394148 -4.285044159 2.33E-05 0.000638039 2.361926818
PDHA1 -0.291015054 5.475111424 -3.548507108 0.000437498 0.005250622 -0.326550875

COQ2 -0.291086703 3.546683427 -4.181335437 3.62E-05 0.000871319 1.955471029
ARSH -0.291224362 0.649643609 -4.100098181 5.08E-05 0.0011298281.643394636
GRHPR -0.291706915 5.059360935 -4.240258898 2.83E-05 0.00073156 2.185299661
KCTD9 -0.292018826 3.378781236 -3.638646817 0.000313176 0.004115811-0.022591677
TMEM63C -0.292080754 1.939234187 -2.633391255 0.008809664 0.043412351 -3.003653872
NCR3 -0.292137919 0.789172601 -3.865159455 0.000131176 0.002245677 0.772359836
HPGDS -0.29219111 1.551254094 -3.102884226 0.00206435 0.015713793 -1.724044447
TRIM56 -0.292364357 4.476120462 -3.022577944 0.00268159 0.018919653 -1.957067516
RBM47 -0.292520051 5.126096494 -3.172200377 0.001639821 0.013284436 -1.518258483
DDB2 -0.292630799 3.468934726 -3.88453637 0.000121523 0.002132319 0.842417163
STXBP2 -0.292823562 4.858225278 -3.450224798 0.000625001 0.006789809 -0.649862857
ABHD14B -0.292834059 6.050081983 -3.049827713 0.002455335 0.017802805 -1.878647391
GSAP -0.292849645 2.168561652 -3.644016888 0.000306934 0.004066185 -0.004259702
CINP -0.292911792 3.945867427 -4.788468576 2.44E-06 0.000129764 4.461198101
ANXA13 -0.29294609 0.955314903 -2.616765354 0.009242063 0.045006103 -3.045286013
TTPA -0.293167123 0.722365433 -3.60232313 0.000358635 0.004547022 -0.145931377
WDR76 -0.29317315 2.832955579 -2.984602685 0.003028873

0.020541083 -2.065237981
PCED1B -0.29346522 2.936551773 -2.634190222 0.00878935
0.043338647 -3.001646828
GPD2 -0.294293232 4.853225092 -3.132232718 0.001873555
0.01463562 -1.637439797
TAPBP -0.295217304 7.613496844 -3.092334991 0.002137194
0.01610411 -1.754985528
PCBD1 -0.295426457 7.900571512 -4.09962348 5.09E-05 0.001130494
1.641587411
ZG16B -0.295701436 1.048298622 -3.57158214 0.000401876
0.004921694 -0.24941546
PEA15 -0.295882908 9.034594905 -3.618766806 0.000337338
0.004358986 -0.09023813
FAM91A1 -0.295932298 4.763956902 -3.598163194 0.00036422
0.004589055 -0.159983328
TNFAIP8 -0.296336215 2.368285871 -3.26021583 0.001216941
0.010776397 -1.250771405
AGFG2 -0.296386647 4.303770952 -3.074471366 0.002265963
0.016780569 -1.807152023
ZFP41 -0.296414267 2.699466765 -3.269394023 0.001179227
0.010562864 -1.222480671
ZNF7 -0.296661303 3.330045138 -4.059663562 6.00E-05
0.001269248 1.490139978
BTN3A3 -0.297205575 4.181851833 -2.774790035 0.005805121
0.032413438 -2.639352639
LAT2 -0.297963231 2.601327176 -2.823572602 0.005007086
0.029096494 -2.509433968
ESRP1 -0.298679533 6.413357926 -2.835787276 0.004823525
0.028397013 -2.476564585
ANKRD6 -0.299546973 3.258884237 -2.683235386 0.007619992
0.039135313 -2.87732241
MGST2 -0.299630281 5.418629626 -3.769962339 0.000190093
0.002901244 0.432862311

NSDHL -0.29995574 5.341504086 -3.959852759 9.00E-05 0.001702195 1.1177769
ZDHHC24 -0.300077657 3.805914235 -4.682806706 3.98E-06 0.000182091 4.003444977
LGALS3BP -0.300078627 9.773455997 -3.174694658 0.001626167 0.013207038 -1.51077331
DTX3L -0.300258528 5.789205784 -2.846362601 0.004669562 0.027794029 -2.447997105
CXorf38 -0.300824147 3.721902504 -3.554539348 0.00042791 0.005160836 -0.306431212
CHCHD4 -0.301130619 4.388644579 -4.455826294 1.11E-05 0.000377293 3.050777945
SLC35F2 -0.301228237 4.832351125-3.041300302 0.002524179 0.018181731 -1.903259656
SLC41A2 -0.301464806 2.81236977 -3.129796539 0.001888751 0.014710139 -1.644658113
DGAT1 -0.301568156 5.727793486 -3.136948316 0.001844461 0.014471234 -1.623452537
IL7 -0.301706532 0.892593604 -4.280952375 2.38E-05 0.000645954 2.345719831
HADH -0.301725415 5.701044008 -3.523129869 0.00048008 0.005640327 -0.410844049
IRAK1 -0.301901144 6.54502212 -2.778329084 0.005743567 0.032230117-2.630000299
OCIAD2 -0.30270786 5.810388229 -3.49474988 0.000532284 0.006055764 -0.504442532
RPL8 -0.302891291 11.44014252-3.254887432 0.001239346 0.010914609 -1.267161206
YWHAH -0.303224603 7.226236035 -3.792452578 0.000174261 0.00273974 0.512364104
MYADM -0.303791091 6.675684587 -2.606617438 0.00951524 0.045814748 -3.070572221
TLR1 -0.303865795 2.192453808 -2.829301656 0.004920215

0.028797441	-2.49403412
SDHA	-0.303932271	5.263401361	-3.764424466	0.000194194
0.002931448	0.413353213
ARL6IP5	-0.304388516	7.07153389	-3.51394242	0.000496431
0.005753049	-0.441222003
PARP12	-0.304619393	4.813043255	-3.014419716	0.002752959
0.019285221	-1.980415427
ESRRA	-0.304638835	4.926184442	-3.515411256	0.000493782
0.005734678	-0.436370322
MACROD1	-0.30512101	4.999953725	-3.009410354	0.00279764
0.019491911	-1.994721929
RAB17	-0.305380027	3.379658474	-2.891346528	0.004063337
0.025293064	-2.325345755
HSF1	-0.305497093	6.863244758	-3.69093138	0.000257165
0.003574242	0.156960067
EEF1D	-0.305800389	5.478890602	-3.55441718	0.000428102
0.005160836	-0.306839003
FAM81A	-0.30627821	1.211404782	-3.789504843	0.000176263
0.002755147	0.501919171
JRKL	-0.306579851	3.451492174	-3.452825291	0.000619196
0.00674569	-0.641417505
GON7	-0.306811954	5.272681616	-3.765717575	0.000193229
0.002927022	0.417906271
KIAA1210	-0.307092366	0.67085757	-3.219306101	0.001399042
0.011907498	-1.375959013
SHARPIN	-0.30759806	6.837648935	-3.917524623	0.000106618
0.001950084	0.962426487
DMC1	-0.308187405	0.88592636	-3.994835252	7.82E-05	0.001536863
1.247321368
NMI	-0.30845944	5.111722698	-3.637068233	0.000315034	0.004126813
-0.027975762
ZRSR2	-0.308536918	3.701283377	-4.474034321	1.02E-05
0.000353889	3.125643048

DENND4C -0.30854791 3.290456636 -3.676404627 0.0002717 0.00370933 0.106835085
OGFRL1 -0.309965114 2.911869521 -3.795404941 0.000172278 0.002721151 0.522832946
PTPN18 -0.310015001 5.422771871 -3.98593395 8.10E-05 0.001581618 1.214259876
PLS1 -0.310535886 3.656816908 -2.905484021 0.003888185 0.024485423 -2.286419965
NUDT22 -0.311551641 4.307374892 -4.543520076 7.51E-06 0.000289233 3.413848044
ETHE1 -0.311849826 5.632936883 -3.259968028 0.001217974 0.010779207 -1.251534186
EIF1AX -0.311948609 5.808067204 -3.968965647 8.68E-05 0.001663461 1.151422597
SETD3 -0.312050974 4.631915937 -4.551646135 7.24E-06 0.000282897 3.447810882
RAB9A -0.312165818 5.120666541 -4.040453151 6.50E-05 0.001336591 1.417814175
SERPINB8 -0.313399537 2.843394662 -3.150017124 0.001765994 0.014013339 -1.584584204
IFNAR2 -0.313655816 3.675196382 -3.799224767 0.000169743 0.002699391 0.536388894
PARP3 -0.313998831 4.683088806 -3.414182912 0.000710881 0.007410205 -0.766299033
WDR91 -0.314024876 4.2254709 -3.6214263 0.000334007 0.004326313 -0.081208519
NDUFB1 -0.314081378 5.630872752 -3.888840342 0.000119472 0.002110444 0.85802189
LEAP2 -0.314410962 1.027818577 -4.459751938 1.09E-05 0.000372362 3.066895747
TLCD4 -0.314486258 2.001341209 -2.86726421 0.004378329 0.026558909 -2.391236054
DUSP23 -0.314586271 7.760531092 -3.557037198 0.000423998

0.005142012	-0.298090675
MYBPH	-0.314875272	1.267688554	-2.585341293	0.010111573
0.047845871	-3.123280234
ACE2	-0.31499678 0.692412322	-4.491455645	9.48E-06	0.000337732
3.197528863
RMI1	-0.315810055	3.741366844	-3.426147924	0.00068122
0.007194262	-0.727772043
TRAPPC6A -0.316164876	5.889645842	-3.303096348	0.001049847
0.009778732	-1.117954877
ADCK2 -0.316173909	5.228081264	-3.869448846	0.000128979
0.002217444	0.78784039
GADD45B	-0.31642615 5.184292489	-2.577211295	0.010348125
0.048637991	-3.143311038
KRTCAP3	-0.316515281	6.344858621	-2.897276268	0.003989015
0.024945607	-2.309041026
TSNARE1	-0.31661507 3.849533302	-3.260578102	0.001215431
0.01077543 -1.249656164
SAMD12	-0.31674658 2.850325782	-3.305780177	0.001040133
0.009738004	-1.109587779
CSF2RA	-0.317905254	2.1694422	-2.741068688	0.006422283
0.034737445	-2.727892142
KCTD13	-0.318474488	3.610679018	-4.586858861	6.18E-06
0.000251653	3.595606114
ATAD2	-0.318853455	4.327293059	-3.031220769	0.002607837
0.018553031	-1.932267346
WARS	-0.319431051	5.966322808	-2.837893525	0.004792498
0.028282758	-2.47088301
TPRG1	-0.319514171	1.646704692	-2.568337236	0.010611959
0.049432847	-3.165105677
C8orf33 -0.319517297	6.42687078 -3.189250751	0.001548562
0.012801549	-1.46698057
TEX264-0.319656286	4.814448115-4.480900398	9.93E-06	0.000347834
3.153944825

DCTPP1 -0.320096078 6.781257649 -3.754111282 0.000202056 0.003018339 0.377092019
MRPL36 -0.320998083 5.775114906-3.817487052 0.000158105 0.002562952 0.601372529
DCUN1D3 -0.321364572 2.835662354 -3.6025317 0.000358357 0.004547022 -0.145226447
CISD1 -0.321898938 4.625206257 -5.050819449 6.93E-07 5.23E-05 5.636374935
RAB27B -0.32219689 1.257581683 -3.462083088 0.000598934 0.006609522 -0.611303607
LXN -0.322221684 5.138448979 -3.036091722 0.002567097 0.018384457 -1.918260744
EXPH5 -0.322409627 1.999214914 -3.85200078 0.00013814 0.002330377 0.724968278
GPR84 -0.323747463 1.976833426 -2.609846134 0.009427551 0.045555233 -3.062537333
CHRNA5 -0.32517936 1.333447769 -3.347907843 0.00089819 0.008811955-0.977413654
PPIF -0.325223452 6.786948103 -3.676522857 0.000271578 0.00370933 0.1072423
MTHFD1 -0.325368414 4.30799629 -4.198599132 3.37E-05 0.000831297 2.022505754
SPINT1 -0.325461488 7.822673176 -2.851288905 0.004599378 0.027451248 -2.43465482
NINJ1 -0.325490909 5.98704889 -4.002283654 7.59E-05 0.001500571 1.275038274
VPS28 -0.325727721 6.909741079 -4.09303992 5.23E-05 0.001146667 1.616542886
ZNF83 -0.325921586 3.787442776 -3.614058185 0.000343312 0.004399516 -0.106209893
SLC1A1 -0.326088052 1.846775902 -3.27804516 0.001144674 0.010362919 -1.195745952
GRINA -0.326135311 8.88008571 -3.66204031 0.000286829 0.003857289

0.0574508

LDLRAD4 -0.326223235 1.831937317 -3.764355169 0.000194246 0.002931448 0.413109257

TSTD1 -0.326436266 6.969674488 -3.552595646 0.000430978 0.005187767 -0.312917628

PGM2L1 -0.326522934 2.812537373 -2.738360664 0.006474329 0.034902262 -2.734957374

GLRX5 -0.326534079 6.11107809 -4.852286096 1.80E-06 0.000101862 4.742019316

KLRB1 -0.326639551 1.48368323 -3.183913629 0.00157661 0.012967156 -1.48305955

SLC37A1 -0.327414111 3.355587393 -3.4304128 0.00067093 0.007112909-0.714008756

RIMBP2 -0.327761492 1.098903888 -2.637037444 0.008717297 0.043088432 -2.994489705

PPP1R16A -0.32812763 5.612040947 -3.804294551 0.000166434 0.002665905 0.55440016

SLC22A18 -0.32845723 4.148411709-3.069093391 0.002306105 0.017035693 -1.822800996

RNF19A -0.328977566 4.64568337 -3.466050003 0.000590443 0.006538121 -0.598376894

AMY1B -0.329206577 0.629505408 -2.662432521 0.008097721 0.040849684 -2.930324718

NKX3-1-0.329550818 1.536129089 -3.057825916 0.002392336 0.017496934 -1.855503109

TRIB1 -0.330160373 5.487640937 -2.870493164 0.004334843 0.026371747 -2.382432035

ETFB -0.331606213 3.687374061 -5.163233171 3.98E-07 3.87E-05 6.156549996

WDYHV1 -0.331654998 4.422480318 -4.221176722 3.06E-05 0.000770992 2.110551703

C8orf82 -0.331770435 6.106447287 -3.78395253 0.000180092 0.002801228 0.482265589

IL18BP -0.332007535 3.228813142 -3.616302982 0.000340451 0.004376904 -0.098597895
ACSL4 -0.332137993 4.734999384 -3.633150135 0.000319689 0.004174356 -0.041329868
FAAH2 -0.332192157 3.669607687 -3.424791221 0.000684524 0.007205097 -0.732146932
TMEM187 -0.332712688 4.256882896 -3.680683081 0.00026734 0.003673209 0.121578981
PDE6B -0.332853219 3.32892197 -3.037528787 0.002555189 0.018339773 -1.914124333
PUDP -0.332873238 4.083166414 -3.74447867 0.000209669 0.003094917 0.343306747
HBEGF -0.333161882 3.334077981 -2.85109734 0.004602089 0.027453021 -2.435174063
PFKP -0.333918876 6.278600816 -2.931774552 0.00358066 0.023078849 -2.213550952
MDFIC -0.334221631 2.894204558 -2.743485774 0.006376149 0.034568813 -2.721580312
SAA4 -0.335009385 0.490199608 -3.914184207 0.000108045 0.00197285 0.95023186
KLF6 -0.33540386 5.871232706 -2.971571909 0.003157225 0.021097723 -2.102055653
SUCLG2 -0.33570626 5.649536913 -4.555856078 7.10E-06 0.000278837 3.46542757
PARP9 -0.336487887 4.820002485 -2.962251019 0.003252068 0.021526989 -2.128297199
ELF3 -0.337355371 6.732220769 -2.745212571 0.006343374 0.03442573 -2.717067796
ZBTB42 -0.337365697 4.518882592 -3.108911042 0.002023767 0.015443633 -1.706322881
ATP2C2-0.337493518 1.152903986 -3.616447623 0.000340268 0.004376904 -0.098107272
OSTF1 -0.338588833 5.55368912 -4.091241343 5.27E-05 0.001151341

1.609707292

FAM153B -0.338654983 0.85827351 -3.668797905 0.000279615 0.003791799 0.080660968

CARD6 -0.338740279 2.615877855 -3.0659319 0.002330007 0.017149229 -1.831988254

KHDRBS3 -0.339405771 2.611876738 -2.703737417 0.007174145 0.037524514 -2.824698965

MYORG -0.339701616 3.810393786 -2.619901395 0.009159076 0.044671549 -3.037452615

TFRC -0.339839779 5.623484922 -2.623661923 0.009060444 0.044310718 -3.02804743

PPA1 -0.339890471 6.777537425 -3.744197619 0.000209895 0.00309543 0.342322197

SDC4 -0.340425276 7.786960461 -3.436208529 0.000657178 0.006994621 -0.695279526

ENDOU -0.340492863 0.954578268 -3.183024296 0.001581329 0.012972992 -1.48573634

HTATIP2 -0.340702303 4.485752265 -3.174392117 0.001627818 0.013207038 -1.511681513

PUM3 -0.340869597 4.763409736 -4.251474805 2.69E-05 0.000705394 2.229376386

STAP2 -0.341083068 4.597406033 -3.332890063 0.000946583 0.009158982 -1.024711843

BRCC3 -0.341341286 4.638255072 -4.01400206 7.23E-05 0.001453804 1.318740182

BACE1 -0.34137051 4.765815326 -3.199259259 0.001497204 0.012500826 -1.436759685

ACER2 -0.341845906 2.242164389 -2.78269076 0.005668518 0.031897492 -2.618458372

MAML2 -0.34192706 4.125938689 -3.192058658 0.001533991 0.01272273 -1.458511065

GNG7 -0.342674916 2.315706641 -2.948445284 0.003397342 0.022197981 -2.167020901

GLB1L2 -0.343605619 3.699767658 -2.686510848 0.007547129 0.038859788 -2.868940959
METRN -0.343952756 4.872296473 -2.651107808 0.008368954 0.041865471 -2.959011576
IL17RE -0.344365091 2.929448652 -3.456118165 0.000611917 0.006702498 -0.630715037
CPLX1 -0.344508656 3.353620109 -2.614466875 0.009303314 0.045225065 -3.051021564
CLN6 -0.34457553 4.885080381 -4.360694919 1.68E-05 0.000501898 2.664080765
BLNK -0.344739148 2.335691653 -3.424293558 0.00068574 0.007213201 -0.733751314
TCTA -0.34507197 5.612992834 -4.225963421 3.00E-05 0.000762643 2.129273318
DDX60 -0.346174602 3.307849426 -2.766895881 0.00594458 0.032901613 -2.660172694
KNDC1 -0.34618918 0.749416245 -3.360692854 0.000858816 0.008529118-0.936990168
EXOSC4 -0.346479074 6.305215505 -3.900418719 0.000114116 0.002042871 0.900079897
WWC3 -0.346585108 3.798600969 -3.499135417 0.000523884 0.005977207 -0.490024997
NXF3 -0.346846167 0.767167742 -2.828189401 0.004936973 0.028834058 -2.497026227
CYP21A2 -0.347875562 0.919931699 -3.422272776 0.000690698 0.007251205 -0.740263727
PDZD4 -0.34793923 1.336271673 -2.663610811 0.00806996 0.040722335 -2.927333218
FTH1 -0.348167315 11.29572541-3.39524859 0.000760292 0.007789745 -0.827008945
TRMT9B -0.348316644 0.394478504 -6.348700895 6.39E-10 3.78E-07 12.22228192
SH2D3A -0.349799848 3.439399998 -3.510110624 0.000503403

0.00581303 -0.45386982

CKMT1A -0.350007876 1.858177493 -2.732262015 0.006592936 0.035399603 -2.750844219

GRAMD1B -0.350065477 2.130805819 -3.011676635 0.002777344 0.019400635 -1.988252337

FAS -0.35023045 3.719751184-3.293723707 0.001084437 0.009980815 -1.147124851

DIAPH1 -0.350478742 5.936042565 -3.888905594 0.000119441 0.0021104440.858258593

LPCAT2 -0.351366823 3.190572289 -3.983256035 8.19E-05 0.001596879 1.204326679

BOP1 -0.35140648 6.100229824 -3.744805875 0.000209406 0.003093857 0.344453065

PIK3R3 -0.351408567 3.946192905 -2.739242631 0.006457336 0.03485712 -2.73265706

GPAA1 -0.351466708 7.597682118-3.741549303 0.000212038 0.003121333 0.333048238

ADCK5 -0.351929119 4.118905058-3.903794873 0.0001125980.002026261 0.912365265

RTN2 -0.353461314 3.395608953 -2.934067145 0.003554925 0.02296788 -2.207166951

ASS1 -0.353492529 9.002038108 -3.028281167 0.002632711 0.018672508 -1.94070994

EFR3A -0.35349739 5.235416219 -3.671062685 0.000277236 0.003765849 0.08844864

LMF1 -0.353691657 2.195911405-4.627095142 5.14E-06 0.000219765 3.765724463

CX3CL1 -0.354394297 3.834952921 -2.657186948 0.008222358 0.04128532 -2.943626997

ACSL1 -0.354937479 3.611734664-3.24247721 0.00129302 0.01122838 -1.305236266

PTPN3 -0.355006669 2.957321909 -3.80247359 0.000167615 0.002674241 0.547928338

ADK	-0.355197504	4.382358602	-4.43585444	1.21E-05	0.000402821	2.96897473
FAM120A	-0.355463475	6.222238392	-4.500258004	9.11E-06	0.000328395	3.233944999
TCIRG1	-0.355789744	5.535239627	-2.984705789	0.003027877	0.020541083	-2.064946058
RLN2	-0.356289151	1.534810279	-3.129667521	0.001889559	0.014710139	-1.64504024
KRT18	-0.356486084	9.304886249	-3.464195342	0.000594399	0.006574173	-0.604422274
ALDH3B1	-0.356553145	4.221239392	-2.797156065	0.005425937	0.030974173	-2.58005601
COX6C	-0.35714554	6.835621771	-3.846581472	0.000141109	0.002363497	0.705493632
HACD4	-0.357442366	2.667386062	-4.555393929	7.12E-06	0.000278837	3.463492976
NMRK1	-0.357615987	3.241012351	-4.495334563	9.32E-06	0.000334899	3.213568444
SLC16A2	-0.357986217	3.389408589	-2.733573793	0.00656726	0.0353326	-2.747429936
IL18	-0.358935696	3.853433073	-2.790468998	0.005536884	0.031419049	-2.597832466
ATAD3C	-0.359190812	2.868857404	-2.884078334	0.004156156	0.025664994	-2.345287284
FHL2	-0.359273773	4.706877902	-3.024447921	0.002665472	0.018871778	-1.951707409
PIEZO1	-0.359790221	3.680427553	-2.596607345	0.009791768	0.046784175	-3.095422333
TNFRSF21	-0.360036592	5.831834482	-2.976679729	0.003106331	0.020878367	-2.087642083
TTC39B	-0.360077168	2.09527972	-4.113291642	4.81E-05	0.001078702	1.693699406
MANEAL	-0.360540459	2.970402117	-2.599883128	0.009700493		

0.046459047	-3.087300317
CD99	-0.360602495	6.717323646	-3.807834509	0.000164159	0.002642753	0.56698959
POGLUT3	-0.360638795	4.076118054	-3.459962777	0.000603519	0.006629992	-0.618207244
CKMT1B	-0.361070363	2.072252719	-2.650260613	0.008389569	0.04192187	-2.961152897
SPATS2L	-0.36135757	4.785822145	-3.770920429	0.000189391	0.002893271	0.436240207
ZHX1	-0.362681734	4.184072697	-4.325756525	1.96E-05	0.000562154	2.523942964
CASP10	-0.362748066	2.948961308	-3.202523784	0.001480796	0.012401536	-1.426883045
FCER1G	-0.363347936	7.311958854	-2.596353101	0.009798884	0.046804353	-3.096052297
TOP1MT	-0.363537881	4.241497864	-3.931111256	0.000100998	0.001869496	1.012124602
SYNGR2	-0.364433821	7.459204516	-3.938989792	9.79E-05	0.001824094	1.04101552
NDRG1	-0.36481232	6.595914087	-2.634307006	0.008786384	0.043337231	-3.00135341
ODF3B	-0.36544733	4.204413801	-2.630249626	0.008889952	0.043741378	-3.011540117
IFI27L1	-0.365948321	3.570407518	-4.020437805	7.05E-05	0.001424033	1.342790912
HCCS	-0.366528879	4.770481378	-4.681920782	4.00E-06	0.00018232	3.999645056
ST6GALNAC4	-0.366600139	5.316493865	-4.311201115	2.09E-05	0.000589157	2.465860707
TMEM61	-0.366746201	2.634182869	-2.787844583	0.005580985	0.031547581	-2.604797908
STYK1	-0.366810277	1.551409154	-3.807546061	0.000164343	0.002642889	0.565963357

TIGD5 -0.367948345 4.124666217 -3.920033889 0.000105558 0.001936191 0.971593168
GNAQ -0.368197118 4.913316118-4.689202184 3.87E-06 0.000178823 4.030895442
SFMBT2 -0.368359808 1.369460155 -4.147246013 4.18E-05 0.000960666 1.823837978
IL24 -0.36845527 0.97108159 -5.453167806 9.09E-08 1.24E-05 7.543359221
TNFSF13B -0.369260372 3.345685581 -2.74850107 0.006281377 0.034181004 -2.708466662
IQANK1 -0.369733763 4.525057435 -2.687825704 0.007518057 0.038734752 -2.865573662
FBXL6 -0.370082123 4.960481781 -3.721655972 0.000228801 0.003284424 0.263579161
LENG9 -0.370264804 3.152558702 -3.191845052 0.001535095 0.01272273 -1.459155613
SUSD6 -0.371078583 4.989495505 -3.489173859 0.000543147 0.006157691 -0.522749423
PRKX -0.373787119 5.027213043 -3.315361208 0.001006131 0.009507703 -1.079665846
APOL2 -0.374836651 5.027665334 -3.218251584 0.001404053 0.011932457-1.379166222
SLC25A1 -0.375334304 6.956345107 -4.676510493 4.10E-06 0.000186141 3.976452936
CYP3A5 -0.375352144 0.946567136 -3.361701474 0.00085578 0.008504179 -0.933794972
LRRC2 -0.376406686 0.753015852 -4.517626133 8.43E-06 0.00031007 3.305985072
CD82 -0.376640606 5.652097112-3.194599967 0.001520914 0.012637565 -1.450839634
TSHZ3 -0.376841385 2.216374815 -3.089579904 0.002156604 0.016212613 -1.763049796
CLDND2 -0.376848957 1.6429678 -4.051598879 6.21E-05

0.001297906 1.459738928
HINT2 -0.37692371 4.911549854 -4.386977865 1.50E-05 0.000465268
2.770169221
TNFRSF10A -0.377688228 2.742950463 -4.338841504 1.85E-05
0.000538888 2.576307996
PCDH1 -0.378076171 5.33559883 -3.153205455 0.001747324
0.013918368 -1.575078497
TICAM1 -0.378526151 4.705082539 -3.811947921 0.000161553
0.002608387 0.581631999
TMPRSS3 -0.378703087 5.148179168 -2.584901538 0.010124244
0.047870266 -3.124365266
STEAP3 -0.37882042 4.128953692 -2.764592981 0.00598583
0.033084648 -2.666235658
DPP7 -0.380081785 6.487394754 -3.104988136 0.002050098
0.015627249 -1.717861701
SLFN11 -0.380308352 3.43057478 -3.596462137 0.000366527
0.004607358 -0.16572502
GLOD5 -0.38091561 2.366840329 -2.940281497 0.003486014
0.022640201 -2.189838433
CCR1 -0.38378268 3.367850597 -2.859458338 0.004485101
0.027014134 -2.412480333
RNF213 -0.3839025 4.468604338 -3.145183052 0.001794652
0.014192564 -1.59897918
ZBP1 -0.38414682 1.541724771 -3.112238238 0.002001679 0.01536203
-1.696525467
GEMIN8 -0.385042844 3.961447006 -4.474730561 1.02E-05
0.000353668 3.12851116
LAP3 -0.385971508 6.199105571 -3.791020446 0.000175231
0.002747361 0.507288584
FCMR -0.386163847 2.386142508 -3.8730897 0.000127141
0.002202231 0.800992742
SYNE2 -0.386220258 2.763292356 -3.948717398 9.41E-05
0.001768707 1.076760195

PLGRKT -0.386488867 5.073633127 -4.7097719 3.52E-06 0.000167399 4.119408505
UBR7 -0.387325734 4.739002963 -4.841032787 1.90E-06 0.000105466 4.692263695
UNC93B1 -0.387537907 6.372473263 -3.961511299 8.94E-05 0.001695979 1.123895127
RHBDL2 -0.387723543 2.340552823 -3.556514393 0.000424814 0.005148049 -0.299836819
CORO1A -0.387845523 4.379451173-3.001429665 0.002870199 0.019809763 -2.017467672
PSME1 -0.388967375 8.394037408 -5.06790162 6.38E-07 5.03E-05 5.71478195
ATP8B1-0.389350969 3.926882198 -3.262521814 0.001207362 0.010744057 -1.24367052
LYRM1 -0.390244998 4.86816498 -4.330694132 1.92E-05 0.000554465 2.543686146
DHRSX-0.390439877 4.009222949 -4.532945482 7.87E-06 0.000296242 3.369732497
GLI4 -0.390617563 4.650934274 -4.339141992 1.85E-05 0.000538888 2.577512198
ZNF683-0.390910847 1.579146445 -2.852633988 0.004580382 0.027353791 -2.431007997
TLR3 -0.390979344 2.556041177-3.869933689 0.000128732 0.002216201 0.789591199
ST6GAL1 -0.391092494 5.803773323 -2.833010449 0.00486471 0.028577079 -2.484048872
FAT1 -0.392494058 3.492997064 -2.998202526 0.002900027 0.019947548 -2.026649031
STK17B -0.392874131 4.11323539 -3.746559052 0.000208002 0.00307874 0.350596653
TYMP -0.393638854 5.488382405 -2.591188409 0.009944446 0.047345864 -3.108836459
CMPK2-0.394046887 3.786793789 -2.588858298 0.010010749

0.047493811-3.114596158

AFAP1L1 -0.395396625 2.567456907 -2.941518964 0.003472439 0.022579227 -2.186383629

FAM189A2 -0.395812501 1.920039243 -3.276546094 0.001150593 0.010403572 -1.200383299

OSCAR -0.396209035 2.758852273 -3.298745222 0.001065776 0.009881821 -1.131506354

NIPA1 -0.397611054 1.918449955 -3.77276855 0.000188046 0.002880873 0.442758281

FRMD4B -0.397743319 3.638887751 -4.070458255 5.74E-05 0.001227653 1.53091846

HEXD -0.398375075 3.873065162 -4.470630644 1.04E-05 0.000357861 3.111627564

PML -0.398539087 5.332037301 -4.271493429 2.47E-05 0.000663786 2.308307751

MFSD3 -0.398870607 5.972606453 -3.591629816 0.000373156 0.004679775 -0.182022105

PCDH7 -0.398962883 3.226346255 -2.985507918 0.003020136 0.020503468 -2.062674607

CYP2J2 -0.399700035 2.367933366 -3.281988006 0.00112924 0.010246128 -1.183539256

SGMS1 -0.399740292 3.891223812 -4.403461111 1.40E-05 0.000445164 2.836994118

IL13RA1 -0.403685777 6.103480609 -4.02466706 6.93E-05 0.001407865 1.358615031

TPRN -0.40384209 4.374223943 -4.518126007 8.41E-06 0.00031007 3.308062128

SERPINB1 -0.403929511 5.872919896 -3.901083126 0.000113816 0.002042871 0.902496811

PYCR3 -0.404878117 5.021760533 -4.034447896 6.66E-05 0.001361429 1.395269047

KCND2 -0.405403898 2.48901897 -3.540492512 0.000450548 0.005376391 -0.353233134

PTPRR -0.406102384 0.826688601 -3.4370681 0.000655161 0.006982341 -0.692499255
MVP -0.406401062 5.720082784 -4.211979993 3.19E-05 0.000790494 2.074635573
ANKRD33B-0.406460936 2.745662726 -2.884025089 0.004156843 0.025664994 -2.345433193
AOPEP -0.407420389 3.558258871 -4.129587744 4.50E-05 0.0010209 1.75603694
HSPB1 -0.407663176 10.86198542 -4.307831476 2.12E-05 0.000595684 2.452439557
PLEKHF2 -0.408711482 4.737474896 -4.094414359 5.20E-05 0.0011440871.621768364
UBE2L6 -0.409185272 7.421701025 -3.587322417 0.000379159 0.004725757 -0.196531699
PPM1J -0.409325087 2.524253158 -4.115982729 4.76E-05 0.001069357 1.703978182
INSIG1 -0.409361555 4.6677974 -3.958810465 9.04E-05 0.00170469 1.11393316
TAPBPL -0.409778484 5.359962748 -3.550432651 0.000434416 0.005225262 -0.320131965
GBP5 -0.410351803 2.284248194 -2.680732765 0.00767609 0.039311105-2.88371965
PGA5 -0.411521771 1.433704557 -2.777295681 0.005761479 0.032252703 -2.632732364
CELF5 -0.411567371 1.360959361 -4.286884821 2.32E-05 0.000634392 2.369221979
PTK6 -0.411668691 2.097000716 -2.650218571 0.008390593 0.04192187 -2.961259143
CAPN2 -0.413195606 5.8871746 -4.188059397 3.52E-05 0.000857239 1.98155033
VSTM1 -0.413593724 0.873740137 -3.181009017 0.001592071 0.013042305 -1.491799488
HPRT1 -0.414384431 5.976936473 -5.011097581 8.42E-07 5.87E-05

5.454939376
EIF4EBP3 -0.414470768 5.162728617 -3.730893313 0.000220867 0.003219094 0.295794085
CEBPD -0.415625312 7.504596774 -3.492746774 0.000536163 0.006095603 -0.511022162
A2M -0.415640999 6.661116969 -3.30329382 0.00104913 0.009778732 -1.117339456
SLC26A2 -0.415690512 3.236592934 -3.504986335 0.000512871 0.005884554 -0.47076371
LONRF2 -0.415881496 2.592065719 -2.829553617 0.004916426 0.028786804 -2.493356158
SYCE1L -0.416364662 2.333523261 -3.822993441 0.000154747 0.002518606 0.621022546
B2M -0.417037957 10.11254707 -3.445395945 0.000635917 0.006858575 -0.665529237
CDT1 -0.417508424 4.636203019 -4.072767261 5.69E-05 0.001217868 1.539653866
IDNK -0.417736613 2.794895361 -4.954377685 1.11E-06 7.17E-05 5.198021071
SEMA3B -0.420130413 5.115531246 -3.304396408 0.001045131 0.009778732 -1.113902611
PYCARD -0.421202192 4.910239822 -3.924281633 0.000103787 0.001916738 0.987123012
MYL5 -0.42135847 2.765980406 -5.134989954 4.58E-07 4.07E-05 6.024930121
FKBP5 -0.422242804 3.431663469 -2.971867647 0.003154258 0.021086603 -2.101221763
RHPN1 -0.422325766 5.151226567 -3.557753625 0.000422882 0.005132327 -0.295697455
XG -0.42281242 1.456303558 -3.296435989 0.00107432 0.009931629 -1.138691577
HLA-DOB -0.423303287 2.294634198 -3.397786331 0.000753489 0.007739645 -0.818890443

OAS1 -0.425113298 5.231933975 -2.639460616 0.008656394 0.04285286 -2.988392669

NPAS2 -0.42547943 2.957433961 -2.886309539 0.004127459 0.025549079 -2.339170688

RAB27A -0.425670241 3.17899279 -4.846000367 1.86E-06 0.000103726 4.71421498

FUT3 -0.425867009 1.773948935 -3.356153983 0.000872607 0.008627549 -0.951357674

GPR27 -0.426706262 4.627358935 -2.893111906 0.00404108 0.025212721 -2.320494909

TM4SF1 -0.427178446 7.946729917 -3.302359225 0.00105253 0.009792718 -1.120251812

SAMD9 -0.427980886 3.66232852 -3.218195551 0.00140432 0.011932457 -1.379336613

PAQR4 -0.428510467 4.222409414 -3.225616969 0.001369395 0.011706479 -1.35674442

OLR1 -0.4287569 3.033414741 -2.768027351 0.005924407 0.032801182 -2.65719204

MCUB -0.429032631 4.034072501 -3.648809292 0.000301463 0.004013021 0.012121461

DDAH1 -0.429097573 4.595289494 -4.341451306 1.83E-05 0.000535723 2.586769235

WT1 -0.4291263 6.905898651 -2.776867557 0.005768915 0.032282777 -2.633863935

DDX58 -0.429128468 4.643477594 -3.325422853 0.000971538 0.009280156 -1.048155291

PSME2 -0.429638674 6.676537778 -5.213704161 3.09E-07 3.13E-05 6.393302197

PTK2B -0.42983092 3.21068804 -4.835403269 1.95E-06 0.000107913 4.667411228

GSTK1 -0.430578041 6.846616632 -5.912703318 7.68E-09 1.83E-06 9.871436234

FAM110C -0.431299202 3.743181096 -3.395643576 0.00075923

0.00778526 -0.825745716
LRP8 -0.431537078 2.918432547 -3.807815045 0.000164172
0.002642753 0.566920341
SLC19A3 -0.431551057 1.735949253 -3.268566243 0.001182583
0.010578131 -1.225035283
GRAMD2B -0.431720986 4.189724891 -3.887636168 0.000120043
0.00211552 0.853654369
PRAG1 -0.432256416 3.911691572-3.783357227 0.000180507
0.002801228 0.480159965
LIF -0.432272744 3.245113848-2.785253899 0.005624831 0.031717852
-2.611667668
MILR1 -0.434033414 2.723986101 -3.432545693 0.000665838
0.007063557 -0.707119633
TSTA3 -0.434578826 6.681051755 -4.92374673 1.28E-06 8.01E-05
5.060334571
PLAAT3 -0.434713551 4.849118997-3.446053623 0.00063442
0.006851564 -0.663396721
MTM1 -0.435358973 3.792817294 -4.494706045 9.34E-06
0.000335093 3.210968639
MSN -0.435838466 7.318116357-4.298731137 2.20E-05 0.000614108
2.416240499
HRH1 -0.43808244 2.841400586 -3.474720754 0.000572275
0.006389412 -0.570073936
RIPK2 -0.438424151 4.707107104 -4.777514795 2.57E-06
0.000134387 4.41332576
SIAE -0.438637942 3.557323715 -4.091780578 5.26E-05
0.0011503361.611756397
PFKFB3 -0.438697017 4.818553716 -3.339679607 0.000924412
0.009014545 -1.003353055
CDC42EP5 -0.43964282 5.372107858 -2.636199467 0.008738448
0.043153423 -2.996596918
NRP2 -0.440091973 3.674629202 -2.856349855 0.004528276
0.027173012 -2.420924905

POU2F3 -0.440136854 2.851211294-2.590526329 0.009963245
0.047391577 -3.110473533
CYC1 -0.441261939 8.513173028 -4.379780746 1.55E-05
0.000475438 2.741061862
FAM83H -0.441901602 6.608007894 -3.673232873 0.000274974
0.003741407 0.095915226
PLSCR1 -0.442268759 6.025818739 -3.775405055 0.000186141
0.002865266 0.452061986
CDC42EP1 -0.442328587 7.011393489-3.718489862 0.000231582
0.003308819 0.252554458
FAM178B -0.444101348 1.934772834 -3.388086336 0.000779804
0.007947678 -0.849891079
BHLHE40 -0.44436876 6.280619326 -3.458645819 0.000606384
0.006650898 -0.622493209
UBA7 -0.444490521 5.276954957 -3.862942232 0.000132326
0.002258191 0.764363995
CD44 -0.445530203 4.13505471 -3.46022045 0.00060296 0.006629992 -
0.617368484
SGMS2 -0.445605718 3.359280026 -4.594402323 5.97E-06
0.000246896 3.627399272
DPYD -0.445960053 2.368493654 -3.767308433 0.000192048
0.002920067 0.423509692
MROH1 -0.446390308 4.683740291 -3.922054218 0.000104712
0.001927136 0.978977611
STEAP2 -0.446702496 3.063394187 -3.774389182 0.000186873
0.002873793 0.448476461
BSPRY -0.447516728 4.363448734 -4.005197906 7.50E-05
0.001491842 1.285895599
ITGB4 -0.447594224 6.495680574 -2.612789881 0.009348233
0.045342074 -3.05520322
RNF183-0.447849128 2.291177974-3.318335267 0.000995788
0.009454146 -1.070361184
RDH5 -0.448298378 1.824486854 -4.032906709 6.70E-05

0.001366554 1.389488004
S100A16 -0.448399135 8.12944209 -3.581223675 0.000387814
0.004796681 -0.217047773
SAA2 -0.44854185 1.453052467 -2.80716312 0.005263668 0.030272174
-2.553377849
TACR1 -0.449217392 1.33577337 -2.872649495 0.004306021
0.026261877 -2.376547346
LYN -0.449245752 4.272449213 -4.475246564 1.02E-05
0.000353668 3.130637059
SLC43A3 -0.449766587 4.598421265 -3.975284377 8.46E-05
0.00163916 1.174793547
S100A11 -0.45261174 11.23434861-4.273546457 2.45E-05 0.000660178
2.316421553
TRIM22 -0.452741129 4.291700827 -3.300953102 0.001057665
0.009828208 -1.124632065
CHCHD10 -0.454803294 7.982717538 -3.586288227 0.000380614
0.004740238 -0.200012985
VNN1 -0.456006636 1.699352635 -3.260549723 0.001215549
0.01077543 -1.249743531
RHOV -0.456134092 2.892368413 -3.132851198 0.001869715
0.01461268 -1.635606416
ITGB2 -0.456515964 4.523783288 -2.834793415 0.004838229
0.028473217 -2.47924411
LYSMD2 -0.456655677 3.737106582 -4.655740683 4.51E-06
0.000198954 3.887639999
TNFRSF14 -0.457632134 3.737797891 -4.152435406 4.09E-05
0.000952261 1.843813222
VSIR -0.458778667 4.549522116-3.719610213 0.000230594
0.003301374 0.256454635
CSF1 -0.458885965 5.587992431 -2.972313902 0.003149785
0.021082074 -2.099963311
SLC38A5 -0.46107495 2.385130054 -2.865556939 0.004401482
0.026633956 -2.395887249

SLC23A1 -0.461129243 1.219226909 -3.951483332 9.31E-05 0.001751244 1.086938537
PPP2R2B -0.46180082 1.230755641 -3.381288089 0.000798754 0.008086511 -0.871568361
SHROOM1 -0.462120687 3.770212542 -4.367085565 1.64E-05 0.000493495 2.689823271
STK32B -0.462799619 1.459381183 -3.394231719 0.000763034 0.00780795 -0.830260426
SLC15A2 -0.463912345 2.976301545 -3.166327474 0.001672388 0.013460642 -1.535860709
CHAF1B -0.464006608 3.084689492 -5.050247245 6.95E-07 5.23E-05 5.633752495
SLC16A9 -0.465279354 3.438337592 -2.909881941 0.003835119 0.024274082 -2.274273921
RASGRP1 -0.466123203 1.818799287 -4.771777232 2.64E-06 0.00013646 4.388288834
RIN1 -0.46662439 2.486202645 -4.725744102 3.27E-06 0.000159204 4.188373979
PRRG4 -0.467314303 3.72568608 -4.041393912 6.47E-05 0.001335201 1.421348786
SLC15A3 -0.46877183 4.325550312 -3.191996738 0.001534311 0.01272273 -1.45869791
GADD45A -0.468907119 5.574611698 -4.640986509 4.83E-06 0.000209373 3.824762919
TAP2 -0.468965015 3.780503821 -4.013112103 7.26E-05 0.001455805 1.315417136
CYP24A1 -0.470885323 0.773475811 -3.679494554 0.000268545 0.003684917 0.117481626
PMAIP1 -0.472059095 3.469477269 -4.100471155 5.08E-05 0.00112964 1.644814715
PTGER2 -0.472394663 2.7314619 -2.853048852 0.004574538 0.027328977 -2.429882875
SERTAD4 -0.472406029 2.5559701 -3.469918627 0.000582271

0.006465331 -0.585757147
CARD16 -0.474016773 3.512866789 -4.783234743 2.50E-06
0.000131723 4.438312159
RBPMS -0.475111089 3.660630727 -3.857252137 0.00013532 0.00229854
0.74386345
ENDOD1 -0.47543202 4.7122938 -4.23437544 2.90E-05 0.000744106
2.162220697
TDO2 -0.475619803 1.862995409 -3.063583598 0.002347909
0.017242154 -1.838806582
ZP3 -0.476426998 3.765792272 -4.221232369 3.06E-05 0.000770992
2.110769236
FAM111B -0.476753984 2.68826979 -3.988390513 8.02E-05
0.001569793 1.223377377
EMB -0.476962312 3.27174001 -3.400534167 0.000746186 0.00768415
-0.810093412
TRIM47 -0.477518297 5.346694484 -4.225479345 3.01E-05
0.00076301 2.127379139
PINLYP -0.477847155 2.849376999 -2.841759376 0.004736021
0.028055421 -2.460444444
ARSD -0.477872806 4.615586698 -4.805817451 2.25E-06
0.000120802 4.537216665
INF2 -0.478002075 5.427802779 -4.157260644 4.01E-05
0.000934606 1.862407087
IBSP -0.480540811 1.726093947 -3.077607722 0.00224285
0.016669001 -1.798013779
PRR15 -0.481209048 4.798428236 -3.335124615 0.000939232
0.009101367 -1.01768682
ZBTB7C -0.481669875 4.536696557 -3.373265155 0.00082167
0.00823095 -0.897098209
FDFT1 -0.482249813 5.916167337 -5.122346372 4.88E-07 4.21E-05
5.966209801
TACC1 -0.482615446 3.368621937 -4.243668836 2.79E-05
0.000724516 2.198689039

OSMR -0.4829396 4.339858026 -3.740839472 0.000212615 0.003126991 0.330563563
ERICH5 -0.482982821 2.689603524 -3.488538658 0.000544397 0.00616323 -0.524833149
COMTD1 -0.483012242 5.418705381 -4.241281952 2.81E-05 0.000729555 2.189315736
GSDMD -0.483567511 4.992318637 -5.189419333 3.49E-07 3.44E-05 6.279138488
SIRPA -0.485544967 4.613735527 -3.787864621 0.000177386 0.002767356 0.49611049
TMEM71 -0.486021602 1.106621371 -6.14197687 2.11E-09 7.95E-07 11.09080037
LGALS9 -0.486056401 5.31710345 -3.857100858 0.0001354 0.00229854 0.743318792
TNF -0.486409399 3.073011781 -2.640963488 0.008618814 0.042719108 -2.98460853
ASPHD1 -0.487624604 3.504304578 -3.061469959 0.002364129 0.017345524 -1.844939321
UBASH3B -0.488847721 1.804317763 -5.055030909 6.79E-07 5.18E-05 5.655684209
SELL -0.489169497 2.615421625 -3.596125533 0.000366985 0.004609535 -0.166860883
RAC2 -0.489610159 4.576023146 -3.658735017 0.000290421 0.003899109 0.046112635
TUBA4A -0.489861994 4.961481685 -3.529335048 0.000469323 0.005542124 -0.390284999
CISH -0.491608923 3.226847901 -4.593867252 5.98E-06 0.000246896 3.6251426
ETV7 -0.494660794 2.856878609 -3.413001772 0.000713874 0.007427043 -0.770095424
TMEM30B -0.495304538 4.861775141 -4.489080877 9.58E-06 0.000339823 3.187715141
IL15RA -0.495327295 3.183723249 -3.807152279 0.000164595

0.00264431 0.564562482
MYO5C -0.496111309 3.283136291 -5.072181766 6.24E-07 5.00E-05 5.734463686
TRIM14 -0.496673978 4.439702506 -4.37204463 1.60E-05 0.000486355 2.709822485
BACE2 -0.49710116 5.277577966 -4.439738234 1.19E-05 0.000400032 2.984856695
WWC1 -0.497323993 4.538327694 -4.886926369 1.53E-06 9.07E-05 4.895813306
LMTK3 -0.498328802 2.522058154 -3.589536023 0.000376062 0.004701615 -0.189077129
KIFC2 -0.49886658 4.538096124 -3.896839656 0.0001157470.002060017 0.887066825
NEURL1B -0.499365285 4.027242153 -4.257867612 2.62E-05 0.000693178 2.25454617
SLCO4A1 -0.501127546 1.674979072 -4.157300954 4.01E-05 0.000934606 1.862562503
VWA5A-0.502730325 3.386975773 -4.092882792 5.24E-05 0.0011466671.615945605
DHCR24 -0.502749816 8.480658102 -3.885681368 0.000120974 0.002127303 0.846566981
NT5E -0.504007904 3.414548677 -2.821434787 0.005039857 0.029234426 -2.515172823
EHF -0.505505985 5.147271278 -3.252404658 0.001249916 0.010977815 -1.274789425
CA12 -0.506169706 3.093186289 -2.815471647 0.0051323 0.029643111-2.531158597
TMC4 -0.506592275 6.733356038 -4.426534663 1.26E-05 0.000414761 2.93091422
ST6GALNAC1 -0.507854323 3.928984401 -2.597025534 0.009780073 0.046742103 -3.094386019
ASMTL-0.508964773 4.409280326 -5.924349229 7.20E-09 1.79E-06 9.932459649

PRPS2 -0.50985581 5.513207844 -5.856370471 1.05E-08 2.23E-06 9.577651675
SAMD9L -0.510056731 3.4048751 -3.835099491 0.000147599 0.002426682 0.66431575
GPR12 -0.510600104 1.061833997 -3.268505257 0.001182831 0.010578131 -1.225223467
AK4 -0.511182429 4.093833407 -4.150548728 4.12E-05 0.000955665 1.836548315
TAP1 -0.512121229 5.695709621 -3.434603647 0.000660959 0.007025623 -0.700468757
CLEC11A -0.513584054 5.982049947 -3.457818219 0.00060819 0.006666192 -0.625185805
PSMB8 -0.514621286 6.850656784 -4.253711696 2.67E-05 0.0007021172.238179594
LDLR -0.515086665 3.845170185 -3.727064112 0.000224124 0.00324566 0.282430907
IFIH1 -0.515297041 4.218784168 -3.810857677 0.00016224 0.002616866 0.577749666
MAL2 -0.515601631 8.211957394-4.409322251 1.36E-05 0.00043691 2.860809994
EPHX2 -0.515841203 3.364431335 -4.975839811 9.99E-07 6.65E-05 5.294936682
PIP -0.517188031 0.656442339 -3.968590244 8.69E-05 0.001664004 1.150035173
ARHGDIB -0.517451621 6.426316063 -4.626306163 5.16E-06 0.000219765 3.762376003
GNLY -0.518013727 2.202489525 -3.066097642 0.002328749 0.017148154 -1.831506834
VDR -0.518020479 3.383282734 -3.966613617 8.76E-05 0.001671351 1.142731888
CLPSL1 -0.520179651 1.666736555 -2.99778767 0.002903882 0.019965575 -2.027828633
SLC40A1 -0.520971184 6.439982989 -3.05690755 0.002399494

0.017533428	-1.858163497
SUSD3	-0.521208701	6.137245274	-3.425765023	0.000682151
0.007194262	-0.729006929
TNFSF10	-0.521626953	6.294453202	-2.794141199	0.005475705
0.031181261-2.588075572
P2RY6	-0.52389352 2.789257334	-4.191010856	3.48E-05	0.000854374
1.993009741
IFITM2	-0.52392084 8.415355912	-3.904646763	0.0001122180.002023923
0.915466721
NUDT8	-0.524612279	4.107907287	-4.991672438	9.25E-07	6.21E-05
5.366664989
MELTF	-0.525487595	3.260574806	-4.115726051	4.77E-05
0.001069357	1.702997523
MST1R	-0.526328377	2.657960691	-3.783230396	0.000180596
0.002801228	0.479711395
HSD3B7	-0.526590207	4.586969404	-5.264263818	2.40E-07	2.58E-
05	6.632450504
MT2A	-0.526734572	8.169403522	-3.762338032	0.000195761
0.002948397	0.406009888
REEP6	-0.527272503	2.844038816	-3.883230771	0.000122152
0.0021387110.837686645
C15orf48	-0.530174231	4.004671447	-3.088941925	0.002161122
0.016231491	-1.764916219
MX1	-0.531915482	5.534699176	-2.851238228	0.004600095
0.027451248	-2.434792185
SMARCA2	-0.532029607	4.203188719	-4.316520201	2.04E-05
0.000578843	2.48706567
EIF4E3	-0.532840722	3.167386388	-5.594889436	4.31E-08	6.62E-06
8.244560298
GAMT	-0.533846513	3.760224324	-3.713977532	0.0002356
0.003340517	0.236857093
APOL6	-0.534613975	4.020583279	-3.779359682	0.000183319
0.002836544	0.466028332

GALNT14	-0.536449407	2.870004871	-3.436458965	0.00065659	0.006992961	-0.694469562
TMC5	-0.53708217	2.079832184	-3.816039529	0.000158999	0.002569723	0.59621125
TRPM4	-0.537423944	3.874276689	-4.950796041	1.13E-06	7.24E-05	5.181883117
ARHGAP26	-0.537966492	3.123391268	-5.134994608	4.58E-07	4.07E-05	6.024951762
CDCA7	-0.538526044	3.657953345	-3.738316847	0.00021468	0.003143529	0.321736944
CXCL8	-0.539045394	3.720635339	-2.714583836	0.006947909	0.036745104	-2.796703484
CXCL3	-0.539509744	1.79433122	-4.013051987	7.26E-05	0.001455805	1.315192689
PLEKHA6	-0.539660186	3.558205627	-4.093237083	5.23E-05	0.001146667	1.617292384
KCNN4	-0.540095482	3.209195629	-3.253434417	0.001245522	0.010951113	-1.271626198
TNFRSF11B	-0.540153528	3.16775694	-2.89279769	0.004045033	0.025227658	-2.321358507
FZD4	-0.540254421	4.088342394	-4.58888987	6.12E-06	0.000250382	3.604161569
NRGN	-0.540586827	4.559255156	-3.011935043	0.002775038	0.019395439	-1.987514362
NRBP2	-0.540646423	5.841825418	-4.006342293	7.46E-05	0.001488599	1.290161097
FAM3B	-0.541189735	2.912699056	-3.077577513	0.002243072	0.016669001	-1.798101838
CDKN2C	-0.542832567	4.345342548	-3.250718369	0.001257142	0.011023331	-1.279967349
CHI3L2	-0.547207923	1.149554104	-4.139780397	4.31E-05	0.000985426	1.795140787
DPF3	-0.547874795	1.14797682	-5.852885903	1.07E-08	2.23E-06	

9.559555162
ZC3H12A -0.548336455 4.185828574 -4.426298941 1.26E-05 0.000414761 2.929952499
METTL7B -0.54888081 4.925404012 -3.44328463 0.000640745 0.006884883 -0.672372583
TUNAR -0.550109736 1.1351787 -2.926016075 0.003646052 0.023395189 -2.229565142
SPTSSB -0.550342449 1.232707153 -4.254777734 2.66E-05 0.000700075 2.242376425
PSMB10 -0.550529951 5.106139904 -5.110726625 5.16E-07 4.33E-05 5.912354603
TBC1D2 -0.551556119 4.060557732 -4.64438553 4.75E-06 0.000207255 3.839232673
SLC1A3 -0.551772458 4.2150907 -4.743502026 3.01E-06 0.000150165 4.265291638
CASP1 -0.55352281 3.361230585 -4.742316023 3.03E-06 0.000150165 4.260146594
LGALS2 -0.553795963 2.097123446 -4.741592238 3.04E-06 0.000150165 4.257007271
MYC -0.553895381 6.769087599 -3.901644039 0.000113563 0.002041357 0.904537544
LYNX1 -0.554291429 3.047432303 -2.724338789 0.006749956 0.036004218 -2.771433308
IGFL1 -0.554515851 1.342412543 -3.239498022 0.001306221 0.011330857 -1.314356126
SMKR1 -0.554558048 3.892994521 -4.387242502 1.50E-05 0.000465268 2.771240316
ST3GAL1 -0.556184613 4.748933318 -3.981162522 8.26E-05 0.001606551 1.196565461
SLC34A2 -0.558524254 8.375496337 -2.727470772 0.006687488 0.035765352 -2.763301475
HLA-DPB1 -0.559195151 7.280010629 -3.482903832 0.000555608 0.006255136 -0.543302221

CFH	-0.55925416	3.5819953	-3.632517378	0.000320447	0.004180794	-0.043485249
OXGR1	-0.563725796	2.352917841	-3.412469881	0.000715225	0.007428779	-0.77180461
GABRE	-0.563825792	2.88872943	-3.14326601	0.001806135	0.014248585	-1.604681986
MGLL	-0.564873536	3.471475865	-4.406872322	1.38E-05	0.000439425	2.850851613
EPHX4	-0.565412392	3.373147694	-4.046058642	6.35E-05	0.001313349	1.438886109
HPSE	-0.565705502	2.969065653	-4.731284373	3.18E-06	0.000156103	4.212344116
C19orf81	-0.568069166	3.542843792	-4.310459145	2.09E-05	0.000590012	2.462904657
B4GALT1	-0.571130886	6.794413328	-5.067087967	6.40E-07	5.03E-05	5.711042095
ACKR2	-0.571347737	2.12815305	-3.223717725	0.001378255	0.011766446	-1.362530751
CHST1	-0.574209138	4.222888415	-3.195130091	0.001518198	0.012621488	-1.449238623
STS	-0.574361882	2.821901074	-5.704039275	2.40E-08	4.14E-06	8.794874021
PALMD	-0.574606997	2.954123934	-4.684614879	3.95E-06	0.000181476	4.011202604
LY6E	-0.574803272	10.15712916	-4.218814862	3.10E-05	0.000775763	2.101321126
MICB	-0.575880898	3.139557937	-3.973167896	8.53E-05	0.001649242	1.166961575
LGALS3	-0.576181692	7.437443683	-5.157513017	4.09E-07	3.88E-05	6.129842556
GPT	-0.585571812	3.218891727	-3.654545162	0.000295035	0.003947947	0.031753805
RFTN1	-0.586167236	3.987880323	-5.682987588	2.69E-08	4.54E-06	

8.688044634

WNT7B -0.586259416 2.414905897 -3.875529296 0.000125923 0.002185822 0.809811988

HLA-DMA -0.58976376 6.58376151 -3.840393058 0.000144572 0.002403788 0.683286015

PRIMA1 -0.590090606 4.529717621 -4.112192177 4.84E-05 0.001082127 1.689501691

C2orf88 -0.590184827 4.367444294 -2.727911778 0.006678735 0.035730347 -2.762155733

ALOX5AP -0.590419743 4.887896993 -3.247656974 0.001270361 0.011091146 -1.289361212

FBP1 -0.590703496 4.164760083 -4.080955874 5.50E-05 0.001188351 1.570669327

LRRC26 -0.590706697 1.682475292 -3.39962185 0.000748603 0.00769924 -0.813014878

RTN4RL1 -0.59134379 1.30164156 -3.969556693 8.66E-05 0.001662194 1.153607245

PNPLA4 -0.592479323 4.311108617 -5.924360264 7.20E-09 1.79E-06 9.93251752

HLA-DPA1 -0.598158415 6.736916806 -3.16757401 0.001665426 0.013438034 -1.532127175

BBOX1 -0.598279734 3.513898932 -3.161214244 0.001701228 0.013645247 -1.551160934

ANXA1 -0.599020214 6.774425775 -4.23917065 2.84E-05 0.000733774 2.18102862

NIPAL2 -0.600040118 3.65927169 -5.327252297 1.74E-07 2.01E-05 6.933146895

PKHD1L1 -0.601860755 1.465557073 -3.264355679 0.001199793 0.010682586 -1.238020069

BCL3 -0.602185571 6.391094818 -4.874452049 1.62E-06 9.39E-05 4.840320196

PROM2 -0.602818706 4.88378548 -3.170103354 0.001651381 0.013338405 -1.524547203

SYBU	-0.605116489	2.879978076	-3.923334189	0.000104179	0.001921796	0.983657801
MUC1	-0.606585476	8.206369447	-3.72686461	0.000224295	0.00324566	0.281735029
STEAP1	-0.607217497	4.231009021	-4.539184864	7.66E-06	0.000292829	3.395751143
TMOD1	-0.609406616	5.036348954	-3.560176508	0.000419129	0.005094418	-0.287600508
SUSD2	-0.613266005	4.678567006	-2.988926995	0.002987346	0.020364122	-2.052986036
GJA5	-0.616043255	2.844407274	-3.709461138	0.000239687	0.003377746	0.221163259
CDCP1	-0.617354058	4.49441555	-4.897370627	1.46E-06	8.79E-05	4.942370874
RASL11A	-0.619163504	3.836886383	-3.892123066	0.00011793	0.002094258	0.869934592
EDN1	-0.621296369	3.58748656	-3.942549009	9.65E-05	0.001804548	1.054084704
SAT1	-0.623211888	7.85729613	-5.256699917	2.49E-07	2.65E-05	6.596547348
FAT2	-0.623511314	2.094602085	-4.256111262	2.64E-05	0.000697245	2.247627656
PCDH19	-0.624199977	2.889199123	-3.790770129	0.000175401	0.002747361	0.506401637
TC2N	-0.625454727	4.542103309	-5.99428857	4.87E-09	1.49E-06	10.30100259
RASSF5	-0.625983413	2.882045787	-4.999909174	8.89E-07	6.02E-05	5.404059115
THEM6	-0.627931703	6.238193135	-5.586210093	4.52E-08	6.76E-06	8.201182584
CFB	-0.629920103	5.24631425	-3.794107384	0.000173147	0.002726058	0.518230986
QPCT	-0.63185315	3.328676482	-3.772498608	0.000188242		

0.0028811470.441806047
STXBP6 -0.633106364 3.274365143 -3.673524891 0.000274671 0.003740429 0.096920233
ATP10A -0.633328578 2.050935666 -5.116165883 5.03E-07 4.28E-05 5.937551331
FOXC1 -0.633464058 3.647847682 -3.20374029 0.001474725 0.012368116-1.423200145
COLCA2 -0.633973562 2.316372868 -4.846201689 1.86E-06 0.000103726 4.715105016
SFN -0.634621185 5.6319302 -3.507148106 0.000508856 0.005855107 -0.463639537
HLA-DRB1 -0.634768562 9.172620014 -3.574702113 0.000397274 0.004876408 -0.238950259
IRF1 -0.637426091 4.757210155 -5.391809008 1.25E-07 1.62E-05 7.24449006
SLC27A6 -0.63813277 2.455118326-4.481981546 9.89E-06 0.000347752 3.15840482
APOL3 -0.638563957 2.943329812 -4.752780259 2.88E-06 0.000145262 4.305581004
KLK11 -0.639434728 5.72278488 -2.842870539 0.004719901 0.02798636 -2.457441568
CCL2 -0.643630374 5.549796072 -4.251493714 2.69E-05 0.000705394 2.229450786
GBP2 -0.645111645 4.143403056 -4.579298963 6.39E-06 0.000257173 3.563790259
APOL1 -0.645441325 6.096361234 -3.772830078 0.000188001 0.002880873 0.442975335
BIRC3 -0.645568097 2.801618435 -4.39177361 1.47E-05 0.000459476 2.789588507
IFI16 -0.647974765 5.908505866 -4.888788815 1.52E-06 9.04E-05 4.904109202
S100A6 -0.650560383 10.28136371 -4.062430879 5.94E-05 0.0012621 1.5005845

IL4I1 -0.651272901 5.91948419 -3.535520921 0.000458825 0.005445981 -0.36975633
SH3RF2 -0.654389668 2.258421485 -5.273298716 2.29E-07 2.49E-05 6.675393834
DEPTOR -0.654602535 5.596723543 -4.438092484 1.20E-05 0.00040128 2.978125226
BCL2L15 -0.657035152 1.065160418 -5.781285335 1.58E-08 2.98E-06 9.189679626
GBP1 -0.658406343 5.410447218 -4.657866632 4.47E-06 0.000198809 3.896714609
CXCL10 -0.658652694 6.382042549 -2.76229311 0.006027285 0.033242544 -2.672285823
CYP4X1 -0.660252121 4.033345299 -3.816338272 0.000158814 0.002569301 0.5972763
PSMB9 -0.663781664 5.344013352 -4.338840637 1.85E-05 0.000538888 2.57630452
NPR1 -0.663842663 6.314551114-4.164847334 3.88E-05 0.000916578 1.891681725
TNFSF14 -0.669207295 1.548784917 -5.039525672 7.33E-07 5.40E-05 5.584662545
C4B -0.669381863 3.216582337 -3.582553665 0.000385911 0.004780451 -0.212576475
C9orf152 -0.669552371 1.957412181 -4.182133798 3.61E-05 0.000869696 1.958565536
HLA-DRA -0.672902763 10.26281691 -3.79868875 0.000170097 0.002700514 0.534485897
ALOX5 -0.673771167 3.959635765 -4.529340101 8.00E-06 0.000298984 3.354712351
KLK10 -0.675321711 6.692770129 -3.150989203 0.001760282 0.013987153 -1.581687004
PTN -0.679393392 4.825786382 -2.988651308 0.002989978 0.020364122 -2.053767637
NAPRT -0.680091725 5.360921071 -4.940563413 1.18E-06 7.48E-05

5.135833599
THSD4 -0.684359542 4.694074689 -3.112083912 0.002002698 0.015362568 -1.696980123
MLPH -0.687632214 3.551021065 -4.299154107 2.20E-05 0.000614052 2.417921447
SPDEF -0.688663363 4.310561215 -2.879612882 0.004214137 0.02590285 -2.357515253
HRCT1 -0.688842275 2.93932626 -3.71123833 0.000238071 0.003363761 0.227336643
CRISP3 -0.693442184 1.553299628 -3.009038882 0.00280098 0.019493101 -1.995781933
SQOR -0.695299003 3.884585723 -6.313263437 7.86E-10 4.11E-07 12.0261901
IFI27 -0.697986934 8.047066863 -3.332142436 0.000949054 0.009171923 -1.027061258
TFAP2C -0.702255889 4.790849515 -4.294005508 2.25E-05 0.000623468 2.397470253
PIGR -0.702942342 2.038953661 -2.664860332 0.008040613 0.040599574 -2.924159483
KRT14 -0.70456318 2.845312533 -2.879860767 0.004210899 0.025902631 -2.356836932
TMEM173 -0.704961892 5.486142039 -5.364359948 1.44E-07 1.78E-05 7.111719345
MPZL2 -0.705411934 5.257422539 -6.117533951 2.43E-09 8.73E-07 10.95900967
AQP9 -0.708859648 2.778525563 -3.882935544 0.000122294 0.002138892 0.836617167
SOX2 -0.709688269 1.532513814 -2.908327445 0.0038538 0.024344698 -2.278569084
CPNE7 -0.710055517 1.672335287 -5.276992001 2.25E-07 2.46E-05 6.692966339
GLIPR2 -0.712466057 4.922194205 -5.969421999 5.60E-09 1.65E-06 10.16956365

ST6GALNAC5 -0.716295047 4.125256095 -3.407918608 0.000726887 0.0075199 -0.786419553
CCDC160 -0.716397039 1.947928186 -5.116282324 5.02E-07 4.28E-05 5.938090982
FBXO32 -0.720952704 4.68001527 -4.443101436 1.17E-05 0.000395044 2.998619862
HUNK -0.721140283 3.232606661 -5.856883434 1.05E-08 2.23E-06 9.58031641
CD74 -0.722697425 11.05686892-4.574737154 6.52E-06 0.00026169 3.544614443
ERP27 -0.729596058 4.583683999 -3.214180712 0.001423556 0.012057739 -1.391538069
IFITM1 -0.730358634 9.702549254 -4.616085119 5.41E-06 0.000227854 3.719043234
CXCL2 -0.734037782 3.002509584 -3.871134816 0.000128124 0.002209805 0.793929434
CIITA -0.734142722 3.001489164 -4.917137181 1.32E-06 8.17E-05 5.030722276
TMEM238 -0.734211876 6.815979108 -5.987394444 5.06E-09 1.52E-06 10.26451711
C4A -0.734584902 3.073401435 -3.844418223 0.0001423110.00237499 0.6977269
GBP4 -0.736499936 4.028509788 -4.323679288 1.98E-05 0.000565212 2.515643123
KLK7 -0.738274761 7.938792523 -3.65350507 0.00029619 0.00395687 0.028191726
CXCL11 -0.740090485 3.641804623 -3.478710164 0.000564092 0.006319861 -0.557029554
GSDMC -0.742004653 1.451845125 -5.628224208 3.61E-08 5.74E-06 8.411685503
RTP4 -0.745699481 5.032620134 -4.771492805 2.64E-06 0.00013646 4.387048372
TTC9 -0.750772036 3.445647983 -5.35534399 1.51E-07 1.83E-05

7.068234992

SAA1	-0.756282373	4.22210602	-2.923323183	0.003677005	0.023539991	-2.237043713
VTCN1	-0.758028663	5.961802664	-3.054819301	0.002415842	0.017573513	-1.864210057
PARP10	-0.760968765	5.623846109	-6.195845011	1.55E-09	6.78E-07	11.38274241
ADRA2C	-0.761143824	5.357030081	-4.286776678	2.32E-05	0.000634392	2.368793295
AC007906.2	-0.762923892	5.68059203	-3.849053357	0.000139747	0.002352582	0.714373386
IL3RA	-0.763761779	2.855183437	-7.413007001	8.54E-13	1.82E-09	18.50378386
CEACAM1	-0.767596753	2.488281836	-5.327322325	1.74E-07	2.01E-05	6.933482897
SMARCD3	-0.773607632	5.289481585	-6.08138067	2.98E-09	1.03E-06	10.76485989
GBP3	-0.779301865	4.066522708	-4.923275321	1.29E-06	8.01E-05	5.058221401
KLK6	-0.780393489	7.68808839	-3.421299642	0.000693097	0.007271675	-0.743398581
CCL20	-0.79847376	3.121802627	-3.727885512	0.000223422	0.003238816	0.285296366
NXNL2	-0.802013572	3.697639994	-4.414914761	1.33E-05	0.000428419	2.883560816
SLC28A3	-0.808646872	2.4622787	-5.627738749	3.62E-08	5.74E-06	8.40924567
SCARA3	-0.812433308	6.823271594	-5.40929406	1.14E-07	1.50E-05	7.329364949
CBR3	-0.815475541	2.771914979	-6.02032512	4.21E-09	1.31E-06	10.43910363
DAPL1	-0.821234352	5.961674114	-3.021509304	0.002690841	0.018970542	-1.960129254

FUT2 -0.824525809 2.898520069 -5.871679633 9.63E-09 2.11E-06 9.65726233
PRSS16 -0.826348906 3.280832651 -6.050419549 3.55E-09 1.15E-06 10.5993356
CLU -0.837698229 9.375696676 -4.173943537 3.74E-05 0.000890823 1.926844919
TNFAIP2 -0.83902233 7.288161052 -5.303163758 1.97E-07 2.21E-05 6.817791675
HLA-DRB5 -0.845681866 6.83846658 -3.515981734 0.000492757 0.005726884 -0.434485478
CXCL1 -0.84576719 3.870922773 -3.637790345 0.000314183 0.004119001 -0.025513119
ASRGL1 -0.849302488 5.000714075 -5.125037417 4.81E-07 4.18E-05 5.978697303
OVGP1 -0.849368728 2.852649825 -2.89765636 0.003984294 0.024925716 -2.307994816
SERPINA1 -0.853039061 5.120767174 -4.694834717 3.77E-06 0.000175238 4.055098635
KLK8 -0.854396504 5.99736741 -5.764449146 1.73E-08 3.18E-06 9.103254105
ACSL5 -0.85576762 4.412970933 -6.061104396 3.34E-09 1.10E-06 10.65638133
GMNC -0.857799108 2.415468849 -3.76447463 0.000194157 0.002931448 0.413529815
C3 -0.861891445 8.574586754 -3.858358654 0.000134733 0.002292014 0.747847885
SLC5A1 -0.871680837 2.437042432 -4.439130522 1.20E-05 0.000400275 2.982370761
GLYATL2 -0.888171918 0.971876756 -4.963289145 1.06E-06 6.93E-05 5.238217896
MT1E -0.895321407 6.955953397 -4.020016247 7.06E-05 0.001424033 1.34121445
EVA1C -0.903613313 4.235559027 -6.632313468 1.18E-10 8.67E-08

						13.82283456
ITGB6	-0.906497117	2.866552504	-5.598876373	4.22E-08	6.57E-06	8.264505177
NTN4	-0.923219316	4.321820624	-5.930659716	6.95E-09	1.79E-06	9.965567123
KRT23	-0.928842973	4.811931586	-4.330025589	1.92E-05	0.000554465	2.541011769
HP	-0.929051868	3.006342852	-3.335329968	0.000938559	0.009101367	-1.017041003
SECTM1	-0.93163499	4.739679103	-5.241003586	2.69E-07	2.83E-05	6.522183476
PAEP	-0.939942905	3.065678516	-3.366983211	0.00084004	0.008382281	-0.917048313
NNMT	-0.966836845	5.425184005	-4.084943567	5.41E-05	0.001176855	1.585793789
RSPO1	-0.972775064	3.699815797	-3.638033227	0.000313897	0.004118592	-0.024684708
HTR3A	-1.018407842	3.816319706	-3.859622023	0.000134065	0.002283059	0.75239841
UNC5A	-1.038738385	3.337989889	-4.736955047	3.10E-06	0.000152971	4.236904089
CYP4B1	-1.052357464	4.656783845	-4.128256307	4.52E-05	0.001023693	1.750935386
LCN2	-1.071824183	8.095931816	-4.566646068	6.77E-06	0.000268955	3.510644913
PLAAT4	-1.109654761	6.89930228	-6.432964923	3.89E-10	2.42E-07	12.6920554
CLDN10	-1.140115507	4.307792902	-4.651900641	4.59E-06	0.000201879	3.871258079
GJB1	-1.179961992	4.688586973	-5.962734345	5.81E-09	1.66E-06	10.1342905
KLK5	-1.197626681	6.804921105	-4.075188752	5.63E-05	0.001210415	1.548819675

CXCL17	-1.260615539	5.558095338	-4.213613523	3.16E-05	0.000786264	2.081009843
IDO1	-1.277259167	3.549287801	-6.344761801	6.54E-10	3.78E-07	12.20044189
LRG1	-1.287617184	3.608511654	-6.808177055	4.03E-11	3.43E-08	14.8426762
MMP7	-1.41977025	6.528544475	-5.208747992	3.17E-07	3.17E-05	6.369965929
DEFB1	-1.458073156	6.421806045	-5.161868219	4.01E-07	3.87E-05	6.15017471
PLEKHS1	-1.466783752	2.310539278	-7.192668803	3.57E-12	5.75E-09	17.1430731
PDZK1IP1	-1.560450278	6.257572674	-7.159665649	4.41E-12	5.75E-09	16.9418859

Supplementary table 7. Univariate cox analysis

	p.value	HR	Low 95%CI	High 95%CI
ARPC1B	0.043134227	1.288243721	1.007850935	1.646644188
DMC1	0.02716877	0.712947107	0.528062687	0.96256295
TCIRG1	0.021306452	1.259771245	1.034952574	1.53342639
TMPRSS3	0.027496473	0.837984371	0.716116619	0.980591411
HLA-DOB	0.029567603	0.813277085	0.675122482	0.979703142
NCCRP1	0.009129443	1.171034227	1.039972226	1.318613255
NPY	0.034412749	1.119884101	1.008344389	1.243761965
TMEM59L	0.019664484	1.181719664	1.027038214	1.359697569
TCF15	0.004955872	1.343675215	1.093467722	1.651135236
ACSS3	0.013985789	1.310703846	1.056317349	1.626352701
